# Benchmark dataset of the effect of grain size on strength in the single-phase FCC CrCoNi medium entropy alloy

**DOI:** 10.1016/j.dib.2019.104592

**Published:** 2019-10-01

**Authors:** M. Schneider, E.P. George, T.J. Manescau, T. Záležák, J. Hunfeld, A. Dlouhý, G. Eggeler, G. Laplanche

**Affiliations:** a Institute for Materials, Ruhr-University Bochum, Universitätsstr. 150, 44801 Bochum, Germany; b Materials Science and Technology Division, Oak Ridge National Laboratory, Oak Ridge, TN 37831-6115, USA; c Materials Science and Engineering Department, University of Tennessee, Knoxville, TN 37996-2200, USA; d Université de Toulouse Ensiacet, 4 Allée Emile Monso, 31030 Toulouse, France; e Institute of Physics of Materials, Academy of Sciences of the Czech Republic, 616 62 Brno, Czech Republic

**Keywords:** NiCoCr, Medium- and high-entropy alloys, Compression-test data, Density and average thickness of annealing twins, Hall-Petch parameters

## Abstract

This data article presents the microstructural data as well as the mechanical properties of the CrCoNi medium-entropy alloy (MEA). The data presented in this article are related to the research article entitled “Analysis of strengthening due to grain boundaries and annealing twin boundaries in the CrCoNi medium-entropy alloy”, see Ref. Schneider et al., 2019. This article can be referred to for the analysis and interpretation of the data, as well as their comparison to other datasets in literature. Microstructural data available in the present paper are backscattered electron micrographs for sixteen different grain sizes. Also available are pdf reports of grain size analysis (annealing twin boundaries were neglected) and crystallite sizes (including annealing twin boundaries) as well as data describing the number of annealing twin boundaries per grain (*n*), corresponding Taylor factors (*M*) and average annealing twin thicknesses (*t*). Additionally, raw data of stress-strain curves at five different temperatures [77 K, 293 K, 473 K, 673 K and 873 K] are given for all sixteen grain sizes, which may be used for further research, e.g. data mining, machine learning and other analytical methods. Mechanical data such as yield stresses (*σ*_*0.2%*_), Hall-Petch parameters (*σ*_*0*_ and *k*_*y*_) and critical boundary strengths (*τ*_*c*_) are provided along with a 1D discrete dislocation dynamics (1-D DDD) simulation results concerning the different boundary strengths.

**Table:** Specifications Table

Subject area	*Materials Science*
More specific subject area	*High- and medium-entropy alloys (HEAs and MEAs)*
Type of data	*Tables (raw stress-strain curve data), images (scanning electron microscopy), pdf-files (assessment of grain sizes)*
How data was acquired	*SEM: Quanta FEI 650 ESEM; Tensile testing machine: Zwick Roell XForce Z100*
Data format	*Raw (stress-strain curves, images), analyzed (grain/crystallite sizes)*
Experimental factors	*Metallographic samples were prepared by grinding and polishing*
Experimental features	*Backscatter electron images were obtained using a SEM of type Quanta FEI 650 ESEM with acceleration voltages between 15 kV and 30 kV and a working distance of 10 mm. Tensile tests were performed at five different temperatures with a strain rate of 10*^*−3*^ *s*^*−1*^*. Assessment of grain and crystallite sizes was carried out using the Heyn lineal intercept method.*
Data source location	*Institute for Materials, Ruhr-University Bochum, Universitätsstr. 150, 44801 Bochum, Germany*
Data accessibility	*Data are with the article*
Related research article	*Schneider, M.; George, E.P.; Manescau, T.J.; Záležák, T.; Hunfeld, J.; Dlouhý, A.; Eggeler, G.; Laplanche, G. Analysis of strengthening due to grain boundaries and annealing twin boundaries in the CrCoNi medium-entropy alloy, Int. J. Plasticity, in press.*https://doi.org/10.1016/j.ijplas.2019.08.009 [1]

**Table:** 

**Value of the Data** • The data compilation contains up-to-date microstructural data and mechanical properties of the ternary CrCoNi medium-entropy alloy. • These datasets constitute benchmark data which can be used for machine learning, i.e. a machine could be trained to determine a mean grain size, the number of annealing twin boundaries per grain, etc. This would help to speed up the analysis of microstructures. • The numerical values can be used to advance machine learning in terms of Hall-Petch plots. Here, the automated evaluation of stress-strain curves and corresponding grain/crystallite size is of importance.

## 1 Data

The data presented in this section summarize microstructural data (e.g. mean grain size (*d*), mean crystallite size (*c*), number of annealing twin boundaries per grain (*n*), Taylor factors (*M*) and average annealing twin thickness (*t*)) obtained for the equiatomic CrCoNi alloy after heat treatments at temperatures between 1073 K and 1473 K for durations ranging from 15 min to three weeks (see Table 1). Different methods were used to assess the grain size, namely the Heyn lineal intercept method using BSE micrographs and electron backscatter diffraction (EBSD), see Ref. [1]. These results are compared in Table 2. Mechanical properties of samples tested in compression having the same grain sizes as listed in Table 1 and resulting Hall-Petch parameters are given in Table 3, Table 4, respectively. Results of 1-D discrete dislocation dynamics (1-D DDD) simulations, conducted to shed light on the relative contributions of grain boundaries and annealing twin boundaries to the materials overall strength are presented in Table 5. Additionally, raw tensile stress-strain curves obtained at temperatures between 77 K and 873 K, BSE micrographs of recrystallized microstructures as well as pdf reports of grain and crystallite size distributions are given in attached files.

**Table 1:** Mean grain size (*d*), crystallite size (*c*), number of annealing twin boundaries per grain (*n*), Taylor factors (*M*) and average thickness of annealing twins (*t*) after heat treatments at different temperatures (*T*) and times. The parameter *d* counts only the grain-boundary intersections whereas *c* is determined by counting intersection with both grain and annealing twin boundaries.

*T* (K)	*time* (min)	*d* (μm)	*c* (μm)	*n* (−)	Magnification	*M*	*t* (μm)
1073	15	3.2 ± 0.4	1.3 ± 0.1	1.5 ± 0.12	500	2.99	0.4 ± 0.01
1073	120	4.2 ± 0.1	2.0 ± 0.1	1.1 ± 0.06	400	3.04	0.6 ± 0.02
1073	180	5.3 ± 0.4	2.3 ± 0.1	1.0 ± 0.15	400	2.99	0.8 ± 0.03
1173	10	4.5 ± 0.6	2.2 ± 0.1	1.3 ± 0.07	400	3.07	0.7 ± 0.02
1173	15	5.7 ± 0.5	2.5 ± 0.2	1.2 ± 0.04	400	3.10	0.9 ± 0.03
1173	20	5.4 ± 0.4	2.9 ± 0.1	0.9 ± 0.04	400	3.04	1.0 ± 0.03
1173	30	7.1 ± 0.4	3.2 ± 0.2	1.3 ± 0.04	300	3.05	1.1 ± 0.03
1173	45	8.4 ± 0.1	4.0 ± 0.1	1.1 ± 0.01	300	3.06	1.5 ± 0.05
1173	60	8.7 ± 0.5	4.8 ± 0.4	0.8 ± 0.03	300	3.08	1.5 ± 0.04
1173	120	13 ± 2	5.7 ± 0.4	1.2 ± 0.15	300	3.09	1.8 ± 0.05
1173	180	16 ± 1	6.9 ± 0.4	1.4 ± 0.06	200	3.09	2.4 ± 0.07
1273	60	33 ± 2	15.8 ± 0.8	1.1 ± 0.01	75	3.08	5.9 ± 0.18
1273	180	42 ± 1	18.5 ± 0.3	1.2 ± 0.01	75	3.10	7.5 ± 0.23
1373	30	61 ± 5	23 ± 1	1.7 ± 0.1	65	3.08	8.7 ± 0.26
1473	30	115 ± 8	42 ± 2	1.8 ± 0.01	70	3.29	13 ± 0.39
1473	30240	174 ± 10	50 ± 3	2.5 ± 0.03	75	3.26	22 ± 0.65

**Table 2:** Comparison of the mean grain size (excluding twin boundaries) obtained by the linear intercept method (*d*_*LIM*_) with that determined by EBSD (*d*_*EBSD*_).

*d*_*LIM*_ (μm)	*d*_*EBSD*_ (μm)
3.2 ± 0.4	2.0 ± 0.4
4.2 ± 0.1	3.4 ± 0.1
4.5 ± 0.6	4.0 ± 0.6
5.3 ± 0.4	4.0 ± 0.4
5.7 ± 0.5	4.6 ± 0.5
5.4 ± 0.4	6.4 ± 0.4
7.1 ± 0.4	5.8 ± 0.4
8.4 ± 0.1	7.4 ± 0.1
8.7 ± 0.5	8.4 ± 0.5
13 ± 2	13.2 ± 1.7
16 ± 1	17.5 ± 1.3
33 ± 2	33 ± 1.6
42 ± 1	37 ± 0.7
61 ± 5	61 ± 3
115 ± 8	126 ± 5
174 ± 10	173 ± 10

**Table 3:** Yield stresses *σ*_*0.2%*_ for sixteen grain (*d*) and crystallite (*c*) sizes obtained at five different temperatures.

*d* (μm)	*c* (μm)	*σ*_*0.2*%_ (MPa)				
		77 K	293 K	473 K	673 K	873 K
3.2 ± 0.4	1.3 ± 0.1	833 ± 17	682 ± 14	592 ± 12	504 ± 10	346 ± 7
4.2 ± 0.1	2.0 ± 0.1	748 ± 15	545 ± 11	465 ± 9	421 ± 8	278 ± 6
5.3 ± 0.4	2.3 ± 0.1	749 ± 15	563 ± 11	458 ± 9	409 ± 8	281 ± 6
4.5 ± 0.6	2.2 ± 0.1	719 ± 14	530 ± 11	429 ± 9	400 ± 8	305 ± 6
5.7 ± 0.5	2.5 ± 0.2	714 ± 14	507 ± 10	419 ± 8	387 ± 8	283 ± 6
5.4 ± 0.4	2.9 ± 0.1	685 ± 13	505 ± 10	411 ± 8	353 ± 7	281 ± 6
7.1 ± 0.4	3.2 ± 0.2	650 ± 13	440 ± 9	364 ± 7	317 ± 6	258 ± 5
8.4 ± 0.1	4.0 ± 0.1	–	424 ± 9	357 ± 7	303 ± 6	238 ± 5
8.7 ± 0.5	4.8 ± 0.4	634 ± 13	415 ± 8	325 ± 7	290 ± 6	219 ± 4
13 ± 2	5.7 ± 0.4	578 ± 12	378 ± 8	296 ± 6	258 ± 5	188 ± 4
16 ± 1	6.9 ± 0.4	566 ± 11	360 ± 7	279 ± 5	232 ± 5	189 ± 4
33 ± 2	15.8 ± 0.8	484 ± 10	290 ± 6	221 ± 4	184 ± 4	197 ± 4
42 ± 1	18.5 ± 0.3	475 ± 10	275 ± 6	200 ± 4	167 ± 3	142 ± 3
61 ± 5	23 ± 1	447 ± 9	263 ± 5	181 ± 4	141 ± 3	122 ± 2
115 ± 8	42 ± 2	438 ± 9	241 ± 5	169 ± 3	134 ± 3	112 ± 2
174 ± 10	50 ± 3	454 ± 9	234 ± 4	157 ± 3	114 ± 2	–

**Table 4:** Hall-Petch parameters *σ*_*0*_ and *k*_*y*_ and the critical boundary strength *τ*_*c*_ for five different temperatures.

*T (K)*	*σ*_*0*_ (MPa)		*k*_*y*_ (MPa μm^1/2^)		*τ*_*c*_ (GPa)	
	*d*	*c*	*d*	*c*	*d*	*c*
77	340 ± 6	330 ± 7	842 ± 25	601 ± 17	2.5 ± 0.3	1.3 ± 0.1
293	150 ± 4	135 ± 4	815 ± 17	598 ± 12	2.5 ± 0.2	1.3 ± 0.1
473	80 ± 3	68 ± 3	775 ± 14	565 ± 10	2.4 ± 0.2	1.3 ± 0.1
673	50 ± 2	35 ± 3	746 ± 13	545 ± 9	2.4 ± 0.2	1.3 ± 0.1
873	50 ± 10^a^	30 ± 10^a^	600 ± 60^a^	470 ± 50^a^	1.7 ± 0.4^a^	1.0 ± 0.3^a^

a based on the three largest grain/crystallite sizes only.

**Table 5:** Critical resolved shear stresses *τ*_*0.2%*_ at room temperature for seven different crystallite sizes (*c*) obtained by compression experiments and 1-D discrete dislocation dynamic (1-D DDD) simulations. For the simulations eight different combinations of grain boundary strength and annealing twin boundary strength (*τ*_*g*_/*τ*_*t*_) were considered.

*c* (*μm*)	*τ*_*0.2%*_ (MPa)	*τ*_*0.2%*_ (MPa)							
	Experiment	0.8/0.8	1.0/0.7	1.3/0.6	1.65/0.5	2.2/0.3	2.8/0.0	0.4/0.9	0.0/0.94
1.3 ± 0.1	*223* ± 7	*247*	*251*	*251*	*247*	*251*	*247*	*249*	*251*
2.0 ± 0.1	178 ± 5	221	224	218	223	219	215	215	215
2.9 ± 0.1	165 ± 5	169	165	167	167	163	163	163	164
4.0 ± 0.1	139 ± 4	155	151	151	153	153	149	153	153
6.9 ± 0.4	118 ± 4	121	119	119	121	119	117	119	119
18.5 ± 0.3	90 ± 3	97	97	97	97	97	95	97	95
42 ± 2	73 ± 2	73	73	73	73	73	71	73	73

Please note, that due to the high resolution (4096 pixels × 3775 pixels) of the BSE micrographs, the upload limit (500 MB) of “Data in Brief” was exceeded. Therefore, out of four BSE micrographs per grain size, only one image is given in the attached files. However, to make all high quality BSE images available for machine learning, all BSE micrographs can be downloaded from https://ruhr-uni-bochum.sciebo.de/s/kyYFnQ1UonJc7Wx. The BSE micrographs can also be sent on request via a link or per e-mail.

## 2 Experimental design, materials, and methods

Backscatter electron (BSE) micrographs were taken using a scanning electron microscope (SEM) of type Quanta FEI 650 ESEM with an accelerating voltage of 15–30 kV and a working distance of ∼10 mm. Mean grain sizes (*d*) and mean crystallite sizes (*c*) were determined using the Heyn linear intercept method. For each micrograph, four equidistant and parallel lines of identical length were used for the assessment. Following the procedure in ASTM E-112, each line intersected at least 50 grains, resulting in 500–1000 intercepts per micrograph. Four backscatter electron (BSE) micrographs, taken at locations spaced 1 mm apart between the center and the outer surface of the annealed rods were used per material state. In the related article, mean grain and crystallite sizes are calculated as the average of four independent measurements and the error bars correspond to the mean deviation from this mean value, similar to the procedure reported in Ref. [2]. The heat treatments described in section 2.1 in Ref. [1] yielded 16 different grain sizes (*d*), as listed in Table 1. Also listed in the table are the crystallite sizes (*c*), which include both annealing twin boundaries and grain boundaries, as well as the number of annealing twin boundaries per grain (*n*), associated Taylor factors (*M*) and the average thickness of annealing twins (*t*). Their detailed size distributions are provided in the supplementary material. In Table 2 the grain sizes shown in Table 1 were remeasured using EBSD (for further details see Ref. [1]) and compared to those assessed using the Heyn lineal intercept method.

Compression tests were performed in a Zwick Roell XForce Z100 machine at temperatures ranging from 77 K to 873 K and at an engineering strain rate of 10^−3^ s^−1^. Plastic strains of ∼16–22% were applied. The resulting raw data, which are given as Excel sheets, are named in such a way that all relevant information can be seen directly in the following order: alloy composition, heat treatment (temperature and time) and temperature at which the compression test was conducted (e.g. CrCoNi_1173 K_30min_873 K). These names are also provided in the first row of the Excel file. From the second to the fifth row, information such as diameter and height (= gauge length) of the sample measured prior to deformation, and the cross-head speed are given. Additionally, for each measured point, different parameters were recorded: time (first column), force (second column), cross-head displacement (third and fourth column) and the temperature (fifth column). For further details on the experimental methods the reader is referred to section 2 of the related article [1]. The yield stresses obtained in compression at five different temperatures [77 K, 293 K, 473 K, 673 K and 873 K] are summarized in Table 3 for all sixteen grain sizes (shown in Table 1). From these data, Hall-Petch parameters such as the intrinsic lattice strength (*σ*_*0*_) and the Hall-Petch slope (*k*_*y*_) as well as the critical boundary strength (*τ*_*c*_) were calculated for five temperatures and the results are listed in Table 4. Additional 1-D DDD simulations were performed to study the relative contributions of grain and annealing twin boundaries to the overall strength of the CrCoNi alloy and compare it to the experimental data. These results are, for eight different combinations of grain boundary strength and annealing twin boundary strength (*τ*_*g*_/*τ*_*t*_) listed in Table 5.
