# Supplementary material for: Benchmark dataset of the effect of grain size on strength in the single-phase FCC CrCoNi medium entropy alloy
Source: Data Brief. 2019 Oct 1;27:104592. doi: 10.1016/j.dib.2019.104592 (PMC6812030; doi:10.1016/j.dib.2019.104592)
Supplement: Multimedia component 1 [file mmc1.zip › CrCoNi_1073K_120min/CrCoNi_1073K_120min_c=2.0μm.pdf]

# Analysebericht

10.11.2017 12:27:24

powered by imagic.ch

## 1. cumulative Result 1

| | |
|---|---|
| Anzahl Bilder | 4 |
| Korngröße (ASTM) | 14,7 |
| Korngröße (G643) | 14,6 |
| Kornstreckung | 96,2 % |
| Mittlere Sehnenlänge | 2 µm |

## 2. Single Result 1 (CrCoNi - ASTM E 112_CrCoNi_homogenized_8.1mmSW_800°C_120min_00186)

| | |
|---|---|
| Mittlere Sehnenlänge | 1,9 µm |
| Korngröße (ASTM) | 14,8 |
| Korngröße (G643) | 14,7 |
| Kornstreckung | 87,7 % |

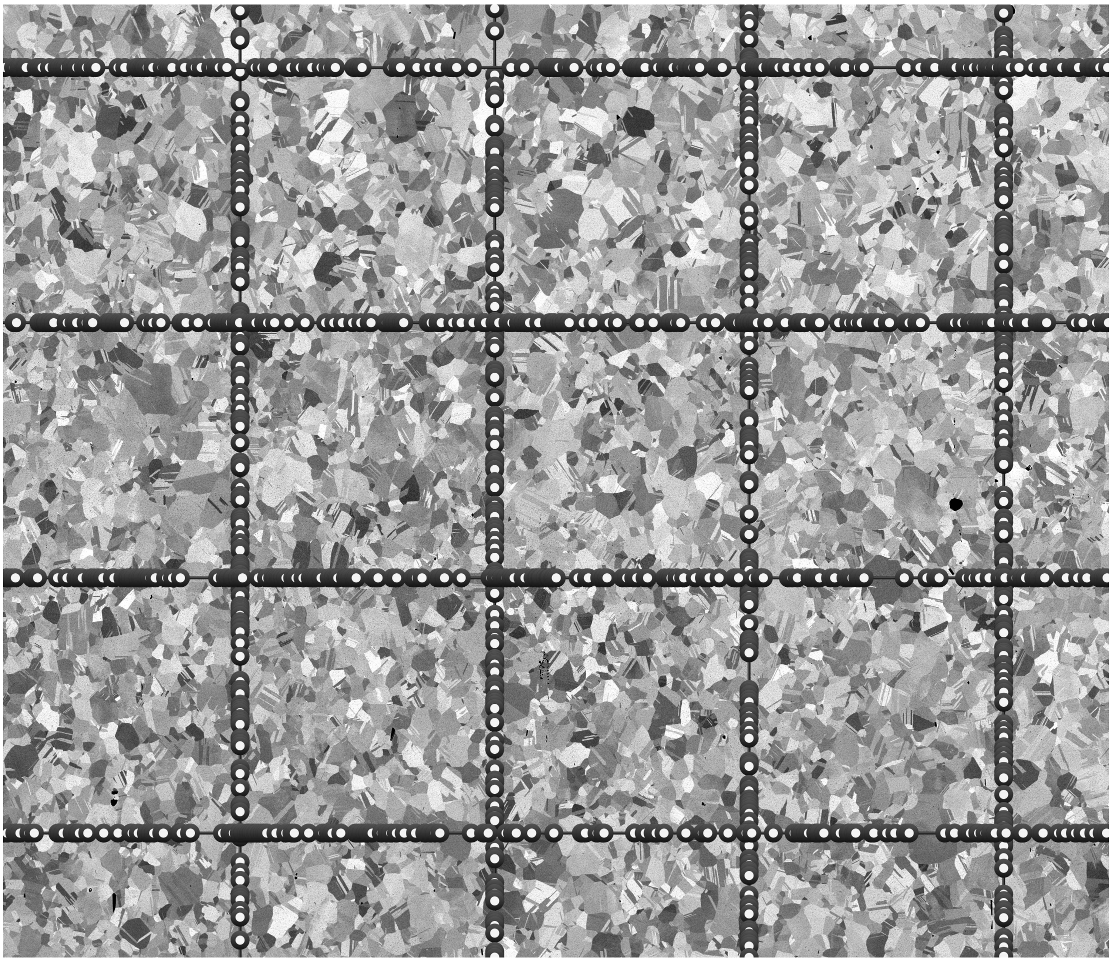

### 2.1. Statistische Analyse

| Statistische Daten | Länge |
|---|---|
| Anzahl Objekte | 1227 |
| Minimum | 0,1 µm |
| Maximum | 12,6 µm |
| Mittelwert | 1,9 µm |
| Standardabweichung | 1,9 µm |
| Schiefe | 0,0 |
| Standardabweichung (n-1) | 1,9 µm |
| Varianz | 3,6 µm² |
| Varianz (n-1) | 3,6 µm² |
| Summe | 2'363,7 µm |

| Statistische Daten | Länge |
|---|---|
| Quadratsumme | 9'017,2 µm² |
| Kubiksumme | 50'638,7 µm³ |

2.1.1. Chord Lenght Distribution

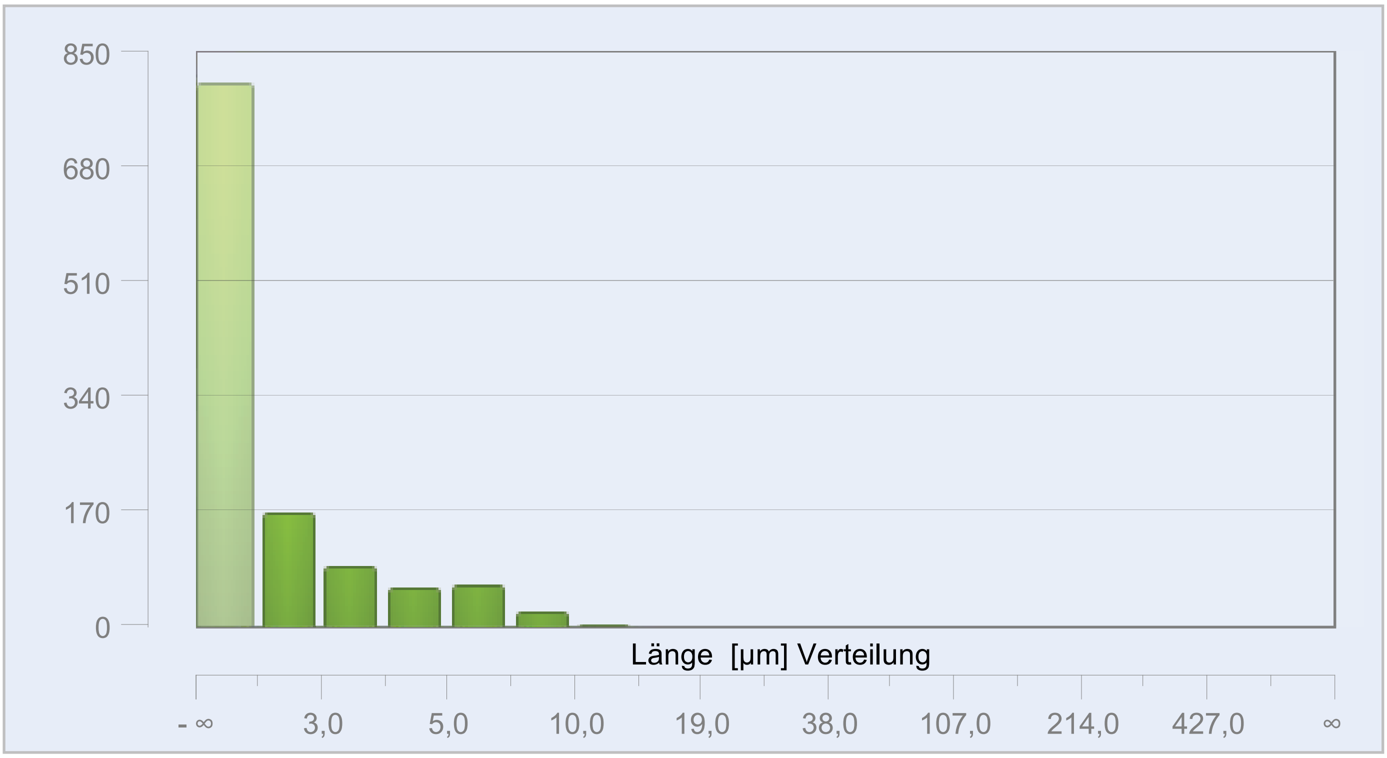


| Start | Ende | Absolute Häufigkeit | Absolute Häufigkeit (kumuliert) | Relative Häufigkeit [%] | Relative Häufigkeit (kumuliert) [%] |
|---|---|---|---|---|---|
| | 2,0 µm | 802 | 802 | 65 | 65 |
| 2,0 µm | 3,0 µm | 171 | 973 | 14 | 79 |
| 3,0 µm | 4,0 µm | 93 | 1066 | 8 | 87 |
| 4,0 µm | 5,0 µm | 62 | 1128 | 5 | 92 |
| 5,0 µm | 7,0 µm | 66 | 1194 | 5 | 97 |
| 7,0 µm | 10,0 µm | 26 | 1220 | 2 | 99 |
| 10,0 µm | 13,0 µm | 7 | 1227 | 1 | 100 |
| 13,0 µm | 19,0 µm | 0 | 1227 | 0 | 100 |
| 19,0 µm | 27,0 µm | 0 | 1227 | 0 | 100 |
| 27,0 µm | 38,0 µm | 0 | 1227 | 0 | 100 |
| 38,0 µm | 75,0 µm | 0 | 1227 | 0 | 100 |
| 75,0 µm | 107,0 µm | 0 | 1227 | 0 | 100 |
| 107,0 µm | 151,0 µm | 0 | 1227 | 0 | 100 |
| 151,0 µm | 214,0 µm | 0 | 1227 | 0 | 100 |
| 214,0 µm | 302,0 µm | 0 | 1227 | 0 | 100 |
| 302,0 µm | 427,0 µm | 0 | 1227 | 0 | 100 |
| 427,0 µm | 600,0 µm | 0 | 1227 | 0 | 100 |
| 600,0 µm | | 0 | 1227 | 0 | 100 |

3. Single Result 2 (CrCoNi - ASTM E 112_CrCoNi_homogenized_8.1mmSW_800°C_120min_00187)

| | |
|---|---|
| Mittlere Sehnenlänge | 2 µm |
| Korngröße (ASTM) | 14,6 |
| Korngröße (G643) | 14,6 |
| Kornstreckung | 98,5 % |

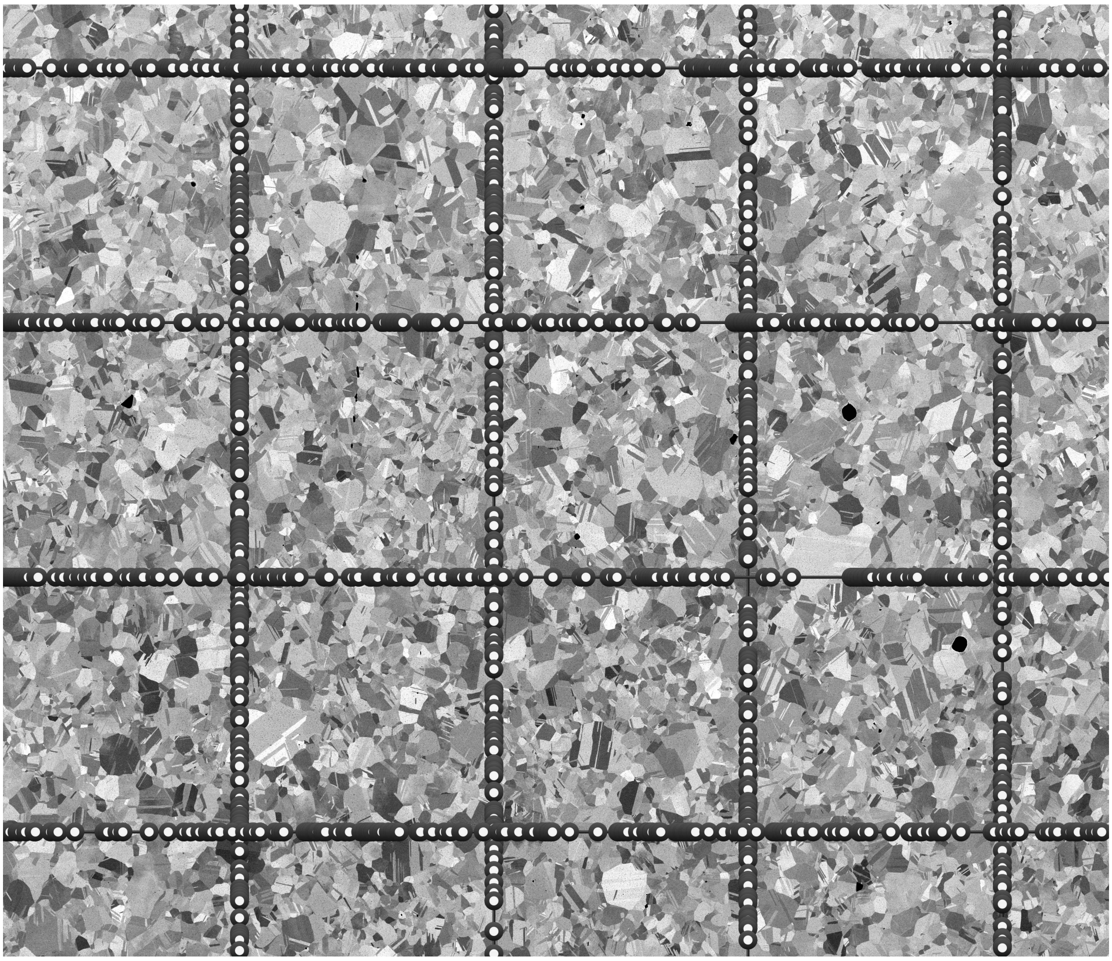

### 3.1. Statistische Analyse

| Statistische Daten | Länge |
|---|---|
| Anzahl Objekte | 1175 |
| Minimum | 0,2 µm |
| Maximum | 17,1 µm |
| Mittelwert | 2,0 µm |
| Standardabweichung | 1,9 µm |
| Schiefe | 0,0 |
| Standardabweichung (n-1) | 1,9 µm |
| Varianz | 3,5 µm² |
| Varianz (n-1) | 3,5 µm² |
| Summe | 2'364,0 µm |
| Quadratsumme | 8'841,4 µm² |
| Kubiksumme | 50'658,4 µm³ |

#### 3.1.1. Chord Lenght Distribution

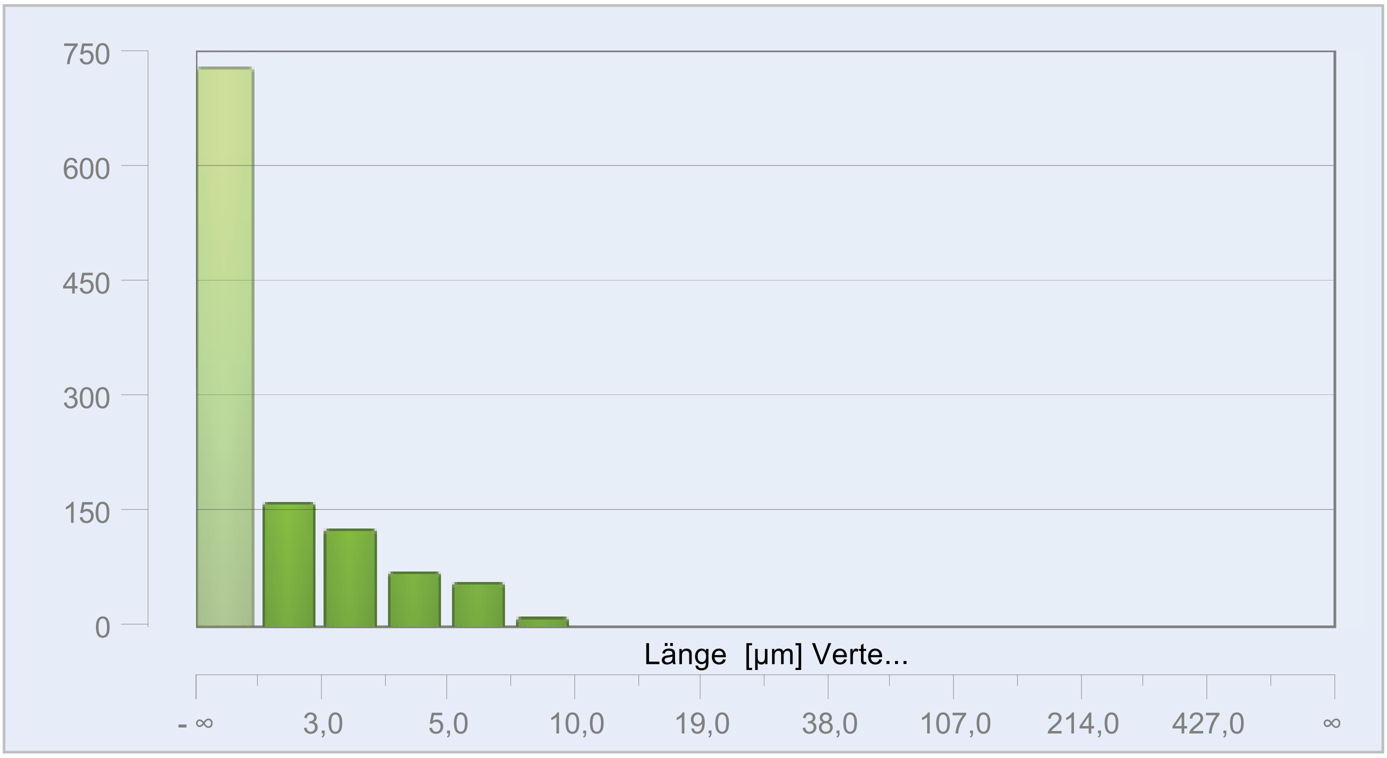


| Start | Ende | Absolute Häufigkeit | Absolute Häufigkeit (kumuliert) | Relative Häufigkeit [%] | Relative Häufigkeit (kumuliert) [%] |
|---|---|---|---|---|---|
| | 2,0 µm | 727 | 727 | 62 | 62 |
| 2,0 µm | 3,0 µm | 165 | 892 | 14 | 76 |
| 3,0 µm | 4,0 µm | 130 | 1022 | 11 | 87 |
| 4,0 µm | 5,0 µm | 73 | 1095 | 6 | 93 |
| 5,0 µm | 7,0 µm | 59 | 1154 | 5 | 98 |
| 7,0 µm | 10,0 µm | 15 | 1169 | 1 | 99 |
| 10,0 µm | 13,0 µm | 3 | 1172 | 0 | 100 |
| 13,0 µm | 19,0 µm | 3 | 1175 | 0 | 100 |
| 19,0 µm | 27,0 µm | 0 | 1175 | 0 | 100 |
| 27,0 µm | 38,0 µm | 0 | 1175 | 0 | 100 |
| 38,0 µm | 75,0 µm | 0 | 1175 | 0 | 100 |
| 75,0 µm | 107,0 µm | 0 | 1175 | 0 | 100 |
| 107,0 µm | 151,0 µm | 0 | 1175 | 0 | 100 |
| 151,0 µm | 214,0 µm | 0 | 1175 | 0 | 100 |
| 214,0 µm | 302,0 µm | 0 | 1175 | 0 | 100 |
| 302,0 µm | 427,0 µm | 0 | 1175 | 0 | 100 |
| 427,0 µm | 600,0 µm | 0 | 1175 | 0 | 100 |
| 600,0 µm | | 0 | 1175 | 0 | 100 |

## 4. Single Result 3 (CrCoNi - ASTM E 112_CrCoNi_homogenized_8.1mmSW_800°C_120min_00188)

| | |
|---|---|
| Mittlere Sehnenlänge | 1,9 µm |
| Korngröße (ASTM) | 14,8 |
| Korngröße (G643) | 14,8 |
| Kornstreckung | 96,2 % |

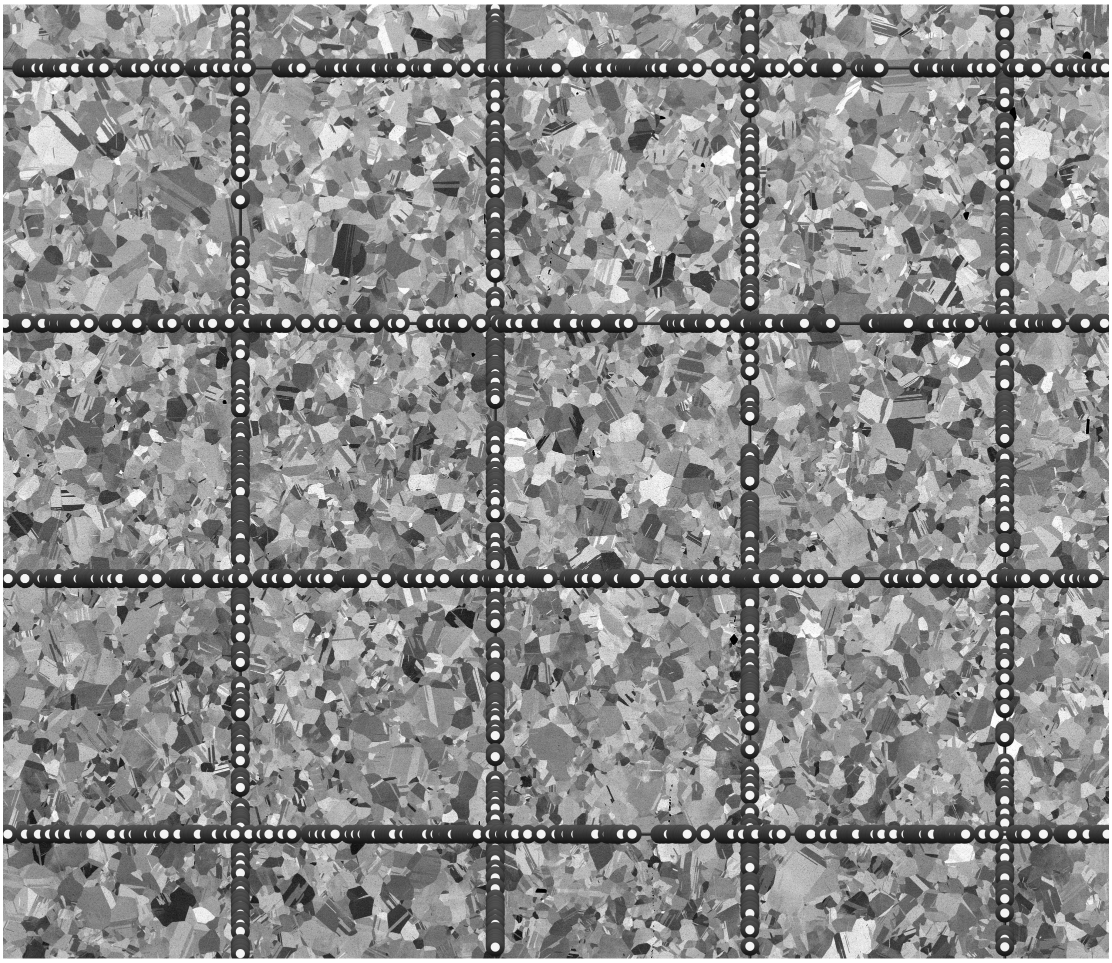

### 4.1. Statistische Analyse

| Statistische Daten | Länge |
|---:|---:|
| Anzahl Objekte | 1261 |
| Minimum | 0,1 µm |
| Maximum | 12,9 µm |
| Mittelwert | 1,9 µm |
| Standardabweichung | 1,7 µm |
| Schiefe | 0,0 |
| Standardabweichung (n-1) | 1,7 µm |
| Varianz | 2,9 µm² |
| Varianz (n-1) | 2,9 µm² |
| Summe | 2'365,0 µm |
| Quadratsumme | 8'105,2 µm² |
| Kubiksumme | 40'725,4 µm³ |

#### 4.1.1. Chord Lenght Distribution

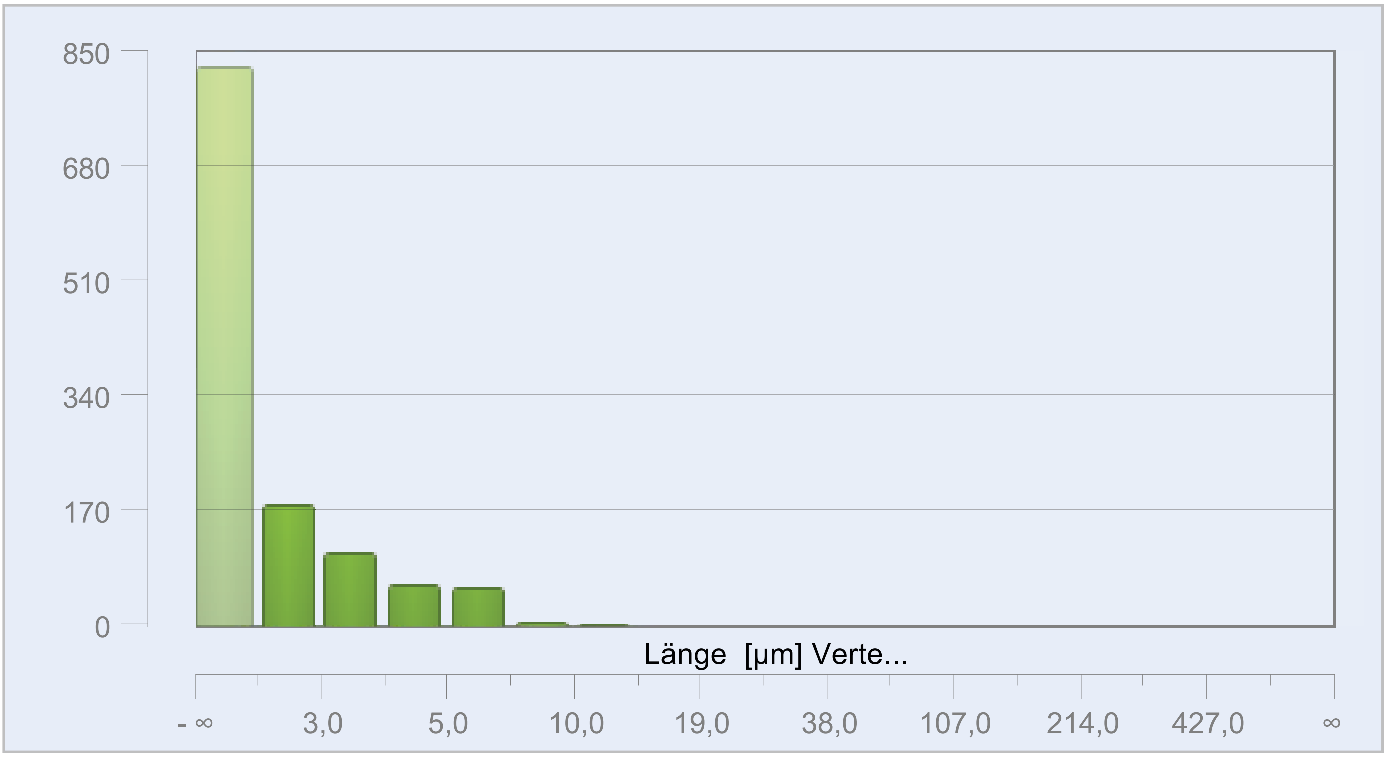


| Start | Ende | Absolute Häufigkeit | Absolute Häufigkeit (kumuliert) | Relative Häufigkeit [%] | Relative Häufigkeit (kumuliert) [%] |
|---|---|---|---|---|---|
| | 2,0 µm | 823 | 823 | 65 | 65 |
| 2,0 µm | 3,0 µm | 183 | 1006 | 15 | 80 |
| 3,0 µm | 4,0 µm | 112 | 1118 | 9 | 89 |
| 4,0 µm | 5,0 µm | 66 | 1184 | 5 | 94 |
| 5,0 µm | 7,0 µm | 62 | 1246 | 5 | 99 |
| 7,0 µm | 10,0 µm | 10 | 1256 | 1 | 100 |
| 10,0 µm | 13,0 µm | 5 | 1261 | 0 | 100 |
| 13,0 µm | 19,0 µm | 0 | 1261 | 0 | 100 |
| 19,0 µm | 27,0 µm | 0 | 1261 | 0 | 100 |
| 27,0 µm | 38,0 µm | 0 | 1261 | 0 | 100 |
| 38,0 µm | 75,0 µm | 0 | 1261 | 0 | 100 |
| 75,0 µm | 107,0 µm | 0 | 1261 | 0 | 100 |
| 107,0 µm | 151,0 µm | 0 | 1261 | 0 | 100 |
| 151,0 µm | 214,0 µm | 0 | 1261 | 0 | 100 |
| 214,0 µm | 302,0 µm | 0 | 1261 | 0 | 100 |
| 302,0 µm | 427,0 µm | 0 | 1261 | 0 | 100 |
| 427,0 µm | 600,0 µm | 0 | 1261 | 0 | 100 |
| 600,0 µm | | 0 | 1261 | 0 | 100 |

5. Single Result 4 (CrCoNi - ASTM E 112_CrCoNi_homogenized_8.1mmSW_800°C_120min_00189)

| | |
|---|---|
| Mittlere Sehnenlänge | 2,1 µm |
| Korngröße (ASTM) | 14,5 |
| Korngröße (G643) | 14,5 |
| Kornstreckung | 95,4 % |

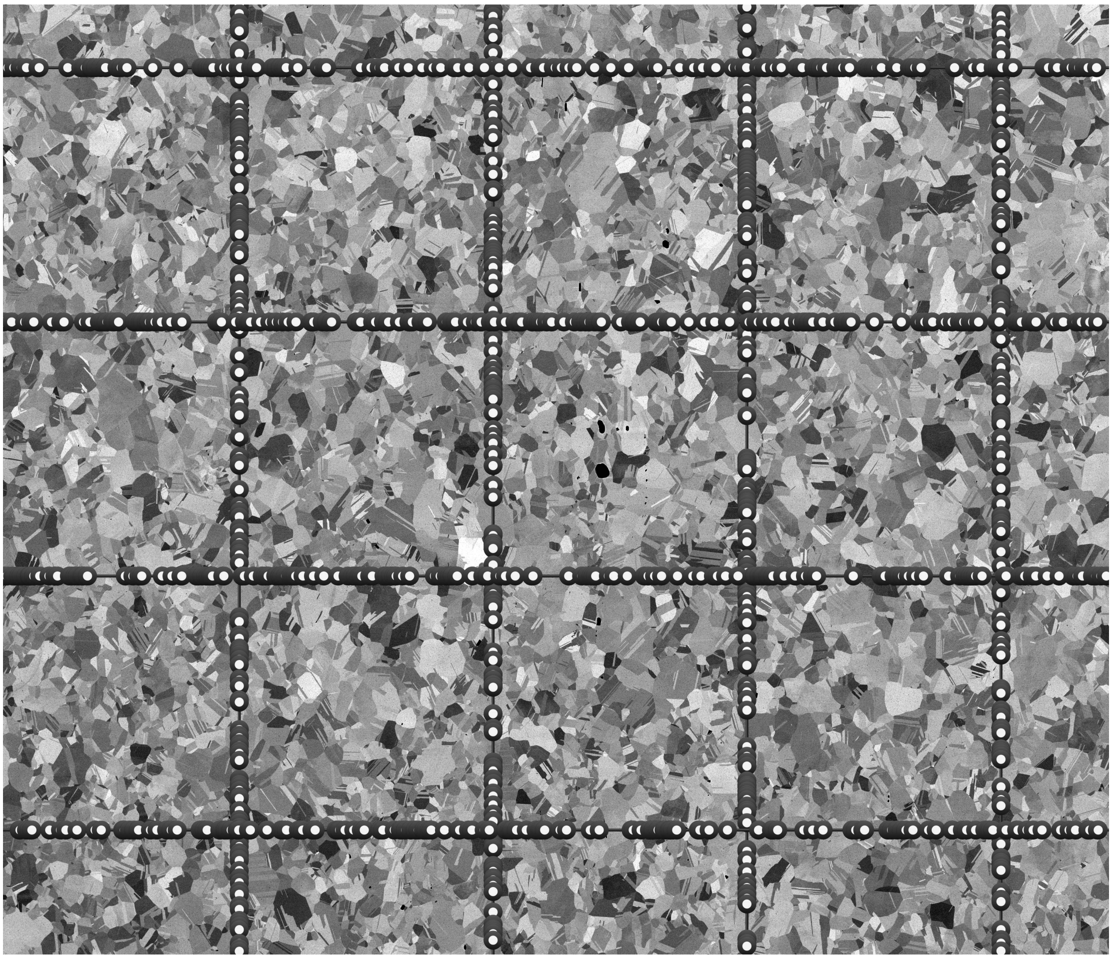

## 5.1. Statistische Analyse

| Statistische Daten | Länge |
|---:|---:|
| Anzahl Objekte | 1137 |
| Minimum | 0,2 µm |
| Maximum | 15,5 µm |
| Mittelwert | 2,1 µm |
| Standardabweichung | 2,0 µm |
| Schiefe | 0,0 |
| Standardabweichung (n-1) | 2,0 µm |
| Varianz | 3,9 µm² |
| Varianz (n-1) | 3,9 µm² |
| Summe | 2'360,6 µm |
| Quadratsumme | 9'318,1 µm² |
| Kubiksumme | 54'231,7 µm³ |

### 5.1.1. Chord Lenght Distribution

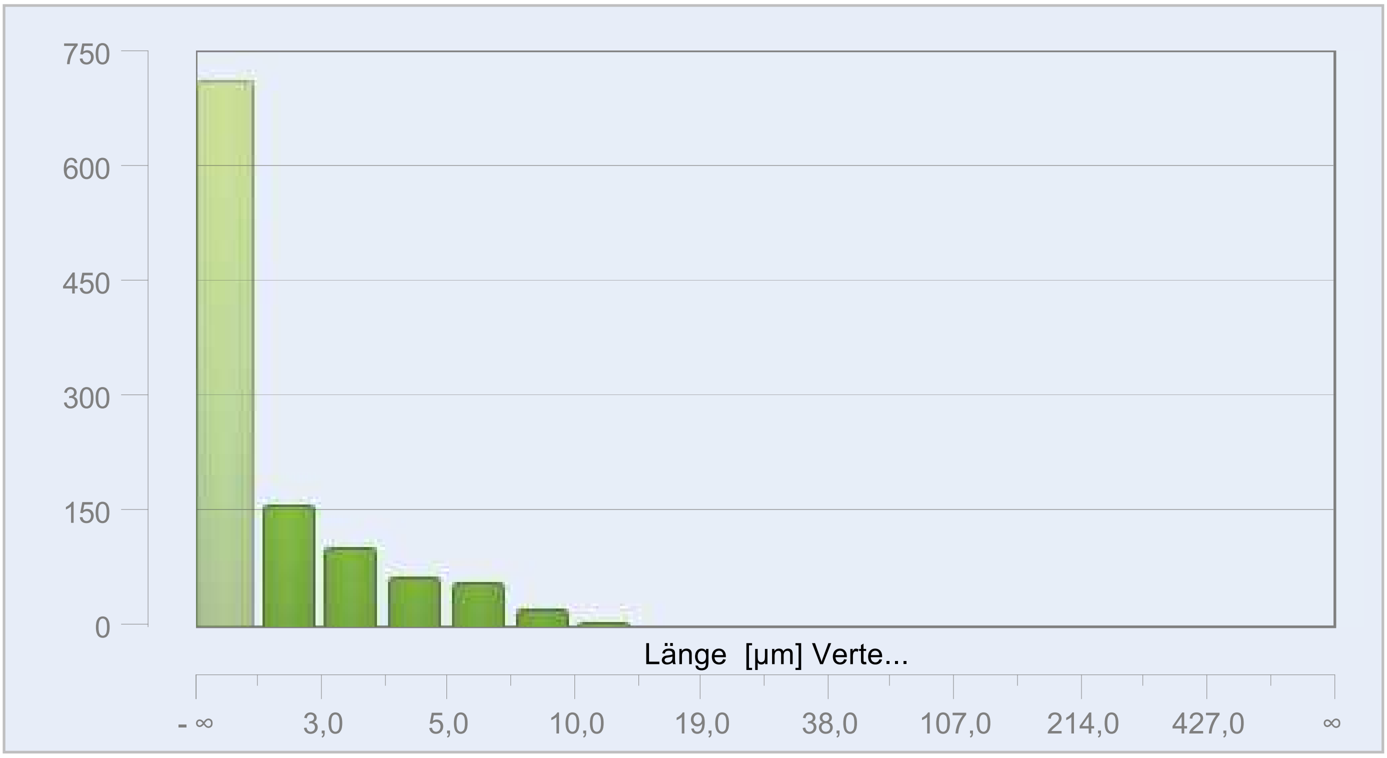


| Start | Ende | Absolute Häufigkeit | Absolute Häufigkeit (kumuliert) | Relative Häufigkeit [%] | Relative Häufigkeit (kumuliert) [%] |
|---|---|---|---|---|---|
| | 2,0 µm | 709 | 709 | 62 | 62 |
| 2,0 µm | 3,0 µm | 162 | 871 | 14 | 77 |
| 3,0 µm | 4,0 µm | 104 | 975 | 9 | 86 |
| 4,0 µm | 5,0 µm | 68 | 1043 | 6 | 92 |
| 5,0 µm | 7,0 µm | 60 | 1103 | 5 | 97 |
| 7,0 µm | 10,0 µm | 26 | 1129 | 2 | 99 |
| 10,0 µm | 13,0 µm | 7 | 1136 | 1 | 100 |
| 13,0 µm | 19,0 µm | 1 | 1137 | 0 | 100 |
| 19,0 µm | 27,0 µm | 0 | 1137 | 0 | 100 |
| 27,0 µm | 38,0 µm | 0 | 1137 | 0 | 100 |
| 38,0 µm | 75,0 µm | 0 | 1137 | 0 | 100 |
| 75,0 µm | 107,0 µm | 0 | 1137 | 0 | 100 |
| 107,0 µm | 151,0 µm | 0 | 1137 | 0 | 100 |
| 151,0 µm | 214,0 µm | 0 | 1137 | 0 | 100 |
| 214,0 µm | 302,0 µm | 0 | 1137 | 0 | 100 |
| 302,0 µm | 427,0 µm | 0 | 1137 | 0 | 100 |
| 427,0 µm | 600,0 µm | 0 | 1137 | 0 | 100 |
| 600,0 µm | | 0 | 1137 | 0 | 100 |
