# Supplementary material for: Benchmark dataset of the effect of grain size on strength in the single-phase FCC CrCoNi medium entropy alloy
Source: Data Brief. 2019 Oct 1;27:104592. doi: 10.1016/j.dib.2019.104592 (PMC6812030; doi:10.1016/j.dib.2019.104592)
Supplement: Multimedia component 1 [file mmc1.zip › CrCoNi_1073K_120min/CrCoNi_1073K_120min_d=4.2μm.pdf]

Analysebericht

Aug 28, 2017 4:34:23 PM

powered by imagic.ch

1. cumulative Result 1

Number of images	4
Grain size (ASTM)	12.5
Grain size (G643)	12.5
Grain stretching	96.4 %
Mean chord length	4.2 µm

2. Single Result 1 (CrCoNi Twins grain size_ASTM 800C 120min_00175)

Mean chord length	4.2 µm
Grain size (ASTM)	12.5
Grain size (G643)	12.4
Grain stretching	91.8 %

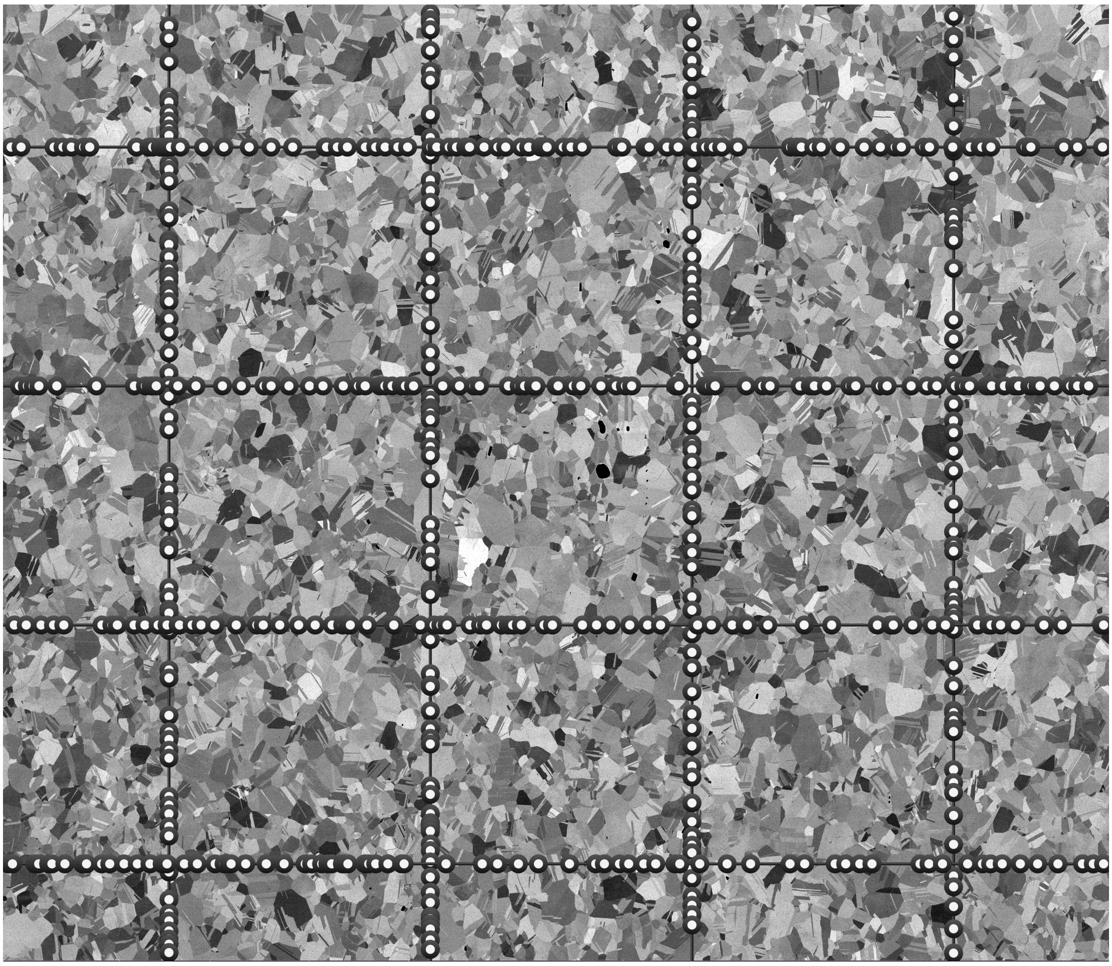

2.1. Statistical Analysis

Statistical Data	Length
Object Count	558
Minimum	0.2 µm
Maximum	15.1 µm
Average	4.2 µm
Standard deviation	2.8 µm
Skewness	0.0
Standard deviation (n-1)	2.8 µm
Variance	7.9 µm²
Variance (n-1)	7.9 µm²
Sum	2'365.9 µm

Statistical Data	Length
Sum of squares	14'437.3 µm²
Sum of cubes	113'124.0 µm³

2.1.1. Chord Lenght Distribution

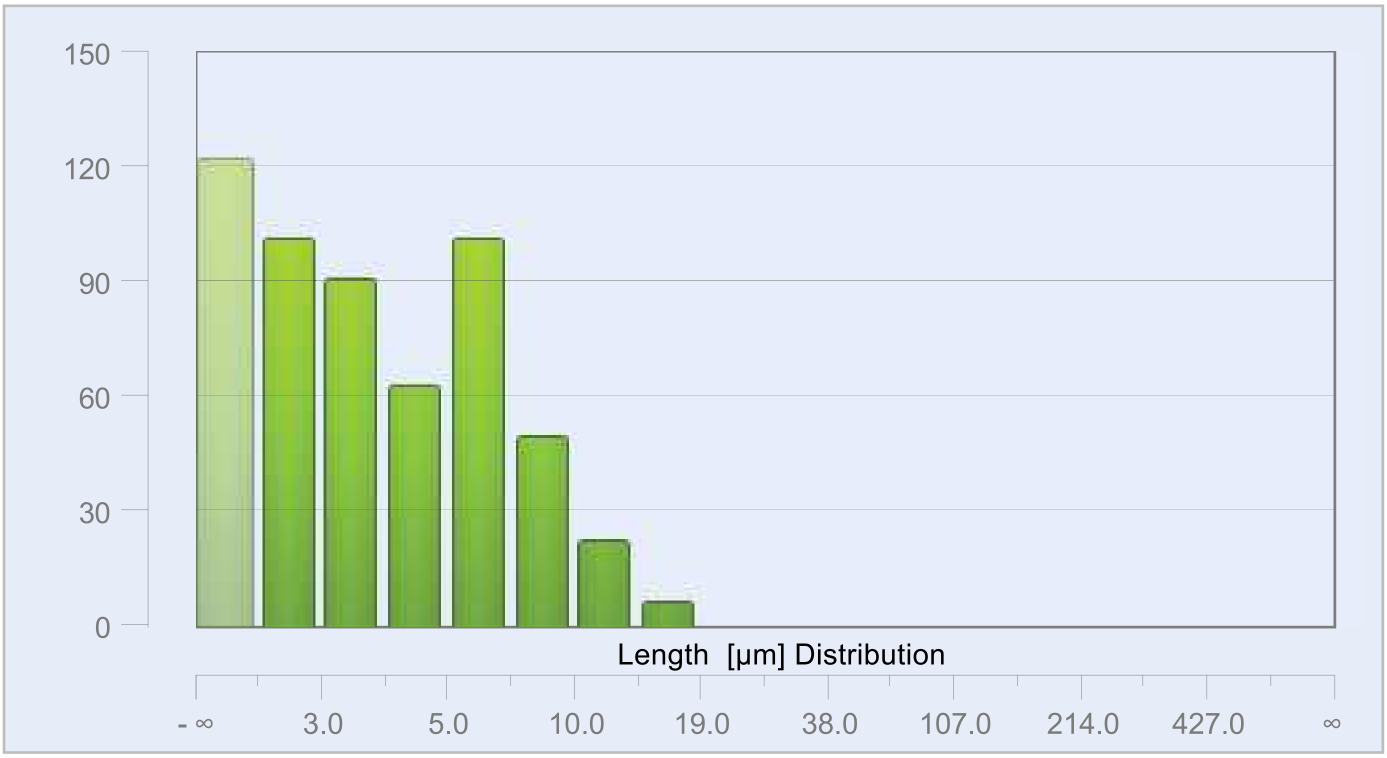

Start	End	Absolute Frequency	Absolute Frequency (accumulated)	Relative Frequency [%]	Relative Frequency (accumulated) [%]
	2.0 µm	122	122	22	22
2.0 µm	3.0 µm	101	223	18	40
3.0 µm	4.0 µm	91	314	16	56
4.0 µm	5.0 µm	63	377	11	68
5.0 µm	7.0 µm	101	478	18	86
7.0 µm	10.0 µm	50	528	9	95
10.0 µm	13.0 µm	23	551	4	99
13.0 µm	19.0 µm	7	558	1	100
19.0 µm	27.0 µm	0	558	0	100
27.0 µm	38.0 µm	0	558	0	100
38.0 µm	75.0 µm	0	558	0	100
75.0 µm	107.0 µm	0	558	0	100
107.0 µm	151.0 µm	0	558	0	100
151.0 µm	214.0 µm	0	558	0	100
214.0 µm	302.0 µm	0	558	0	100
302.0 µm	427.0 µm	0	558	0	100
427.0 µm	600.0 µm	0	558	0	100
600.0 µm		0	558	0	100

3. Single Result 2 (CrCoNi Twins grain size_ASTM 800C 120min_00176)

Mean chord length	4.3 µm
Grain size (ASTM)	12.5
Grain size (G643)	12.4
Grain stretching	92.8 %

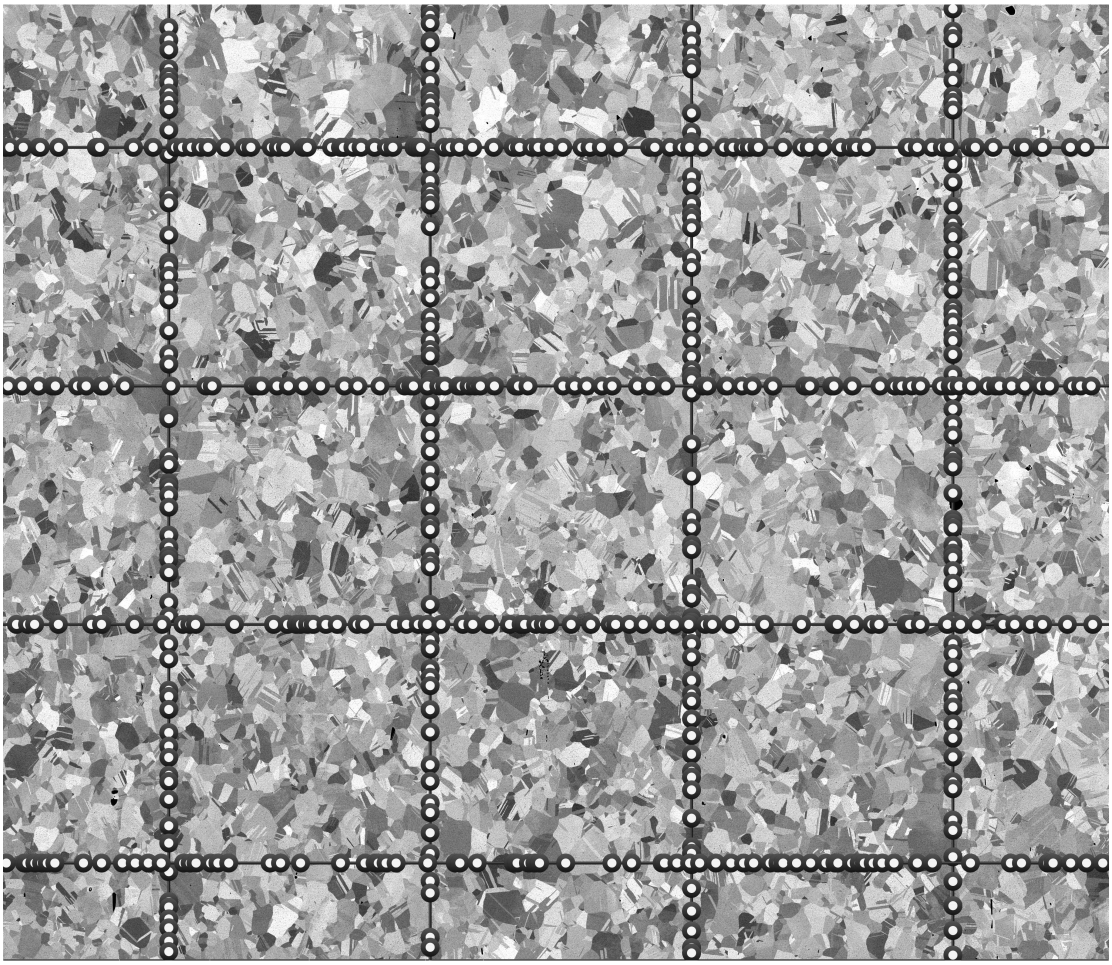

3.1. Statistical Analysis

Statistical Data	Length
Object Count	555
Minimum	0.4 μm
Maximum	14.6 μm
Average	4.3 μm
Standard deviation	2.7 μm
Skewness	0.0
Standard deviation (n-1)	2.7 μm
Variance	7.5 μm²
Variance (n-1)	7.5 μm²
Sum	2'365.6 μm
Sum of squares	14'237.8 μm²
Sum of cubes	108'911.6 μm³

3.1.1. Chord Lenght Distribution

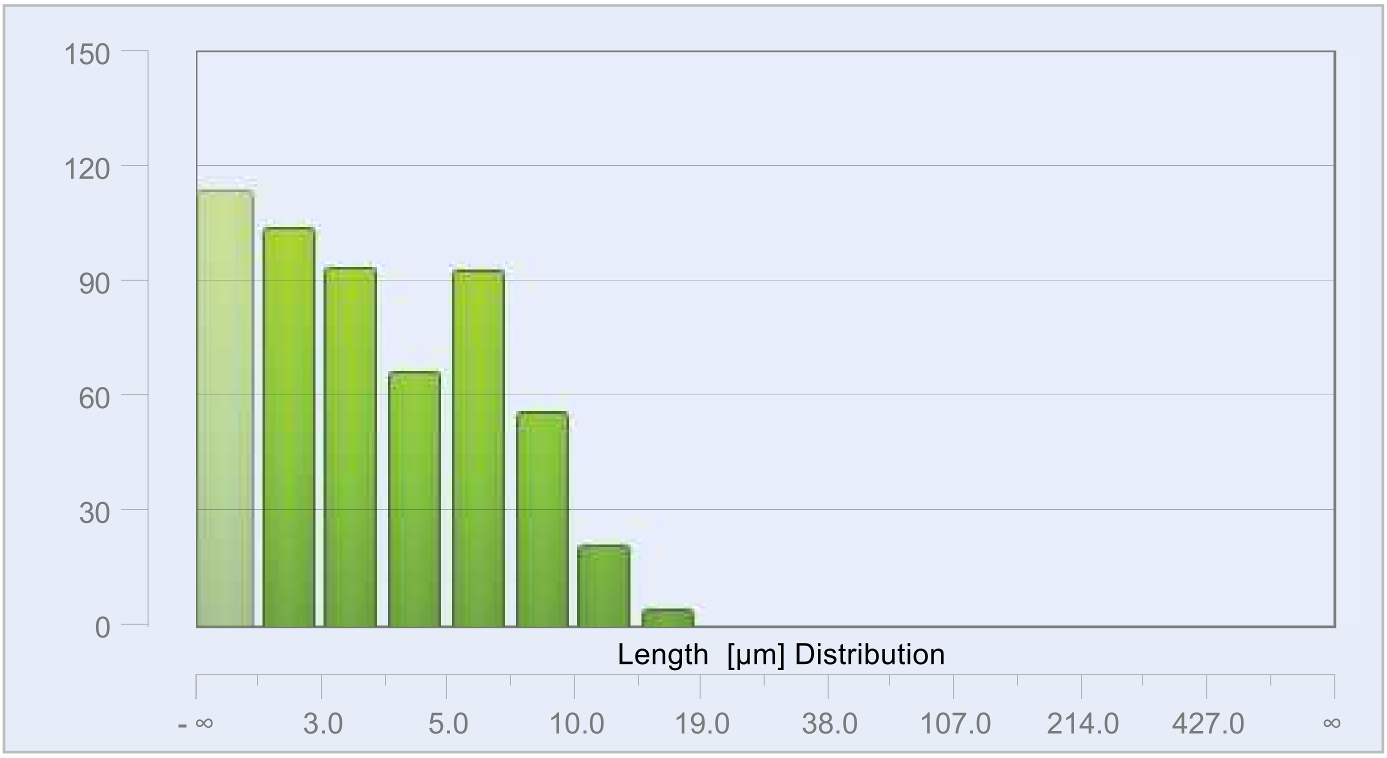

Start	End	Absolute Frequency	Absolute Frequency (accumulated)	Relative Frequency [%]	Relative Frequency (accumulated) [%]
	2.0 µm	114	114	21	21
2.0 µm	3.0 µm	104	218	19	39
3.0 µm	4.0 µm	94	312	17	56
4.0 µm	5.0 µm	67	379	12	68
5.0 µm	7.0 µm	93	472	17	85
7.0 µm	10.0 µm	56	528	10	95
10.0 µm	13.0 µm	22	550	4	99
13.0 µm	19.0 µm	5	555	1	100
19.0 µm	27.0 µm	0	555	0	100
27.0 µm	38.0 µm	0	555	0	100
38.0 µm	75.0 µm	0	555	0	100
75.0 µm	107.0 µm	0	555	0	100
107.0 µm	151.0 µm	0	555	0	100
151.0 µm	214.0 µm	0	555	0	100
214.0 µm	302.0 µm	0	555	0	100
302.0 µm	427.0 µm	0	555	0	100
427.0 µm	600.0 µm	0	555	0	100
600.0 µm		0	555	0	100

4. Single Result 3 (CrCoNi Twins grain size_ASTM 800C 120min_00177)

Mean chord length	4.2 µm
Grain size (ASTM)	12.5
Grain size (G643)	12.5
Grain stretching	93.4 %

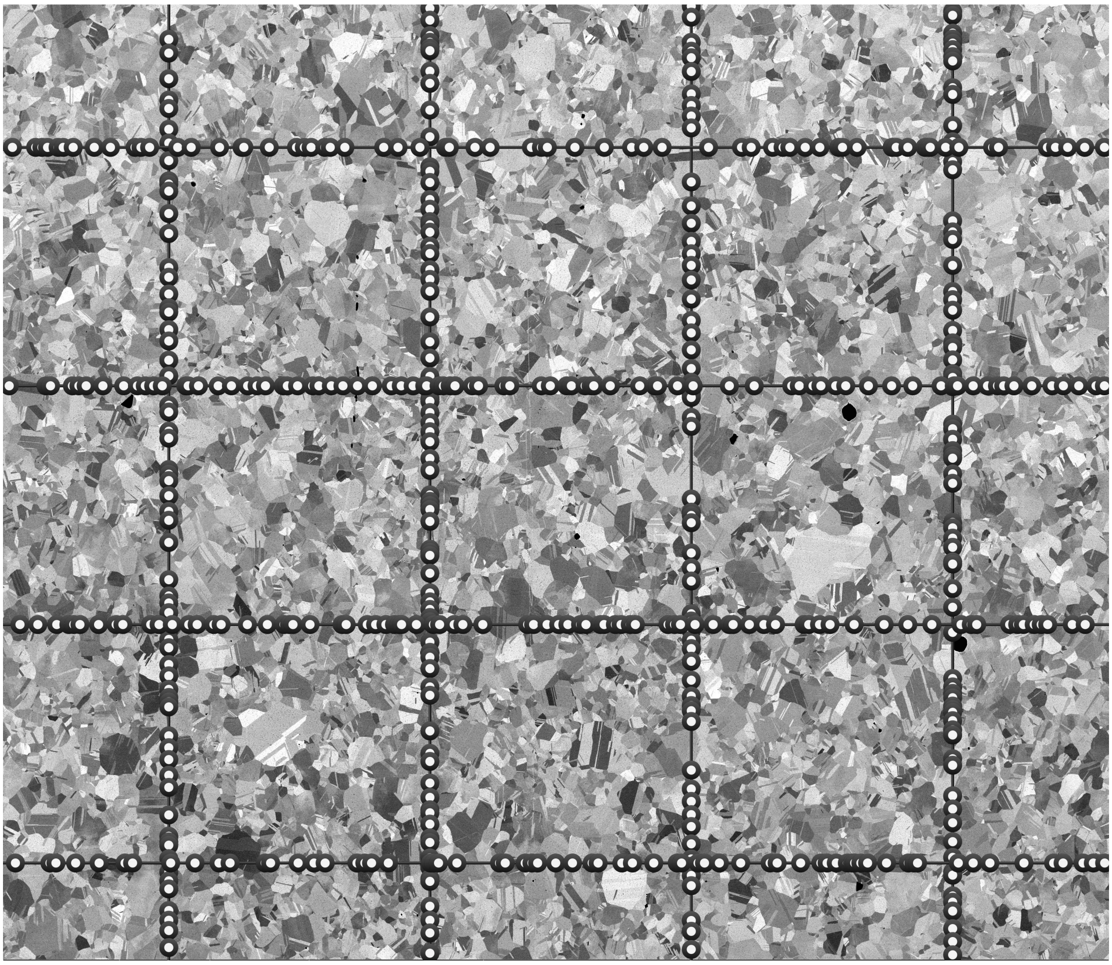

4.1. Statistical Analysis

Statistical Data	Length
Object Count	564
Minimum	0.5 µm
Maximum	20.8 µm
Average	4.2 µm
Standard deviation	2.8 µm
Skewness	0.0
Standard deviation (n-1)	2.8 µm
Variance	7.7 µm²
Variance (n-1)	7.7 µm²
Sum	2'365.3 µm
Sum of squares	14'258.4 µm²
Sum of cubes	113'509.5 µm³

4.1.1. Chord Lenght Distribution

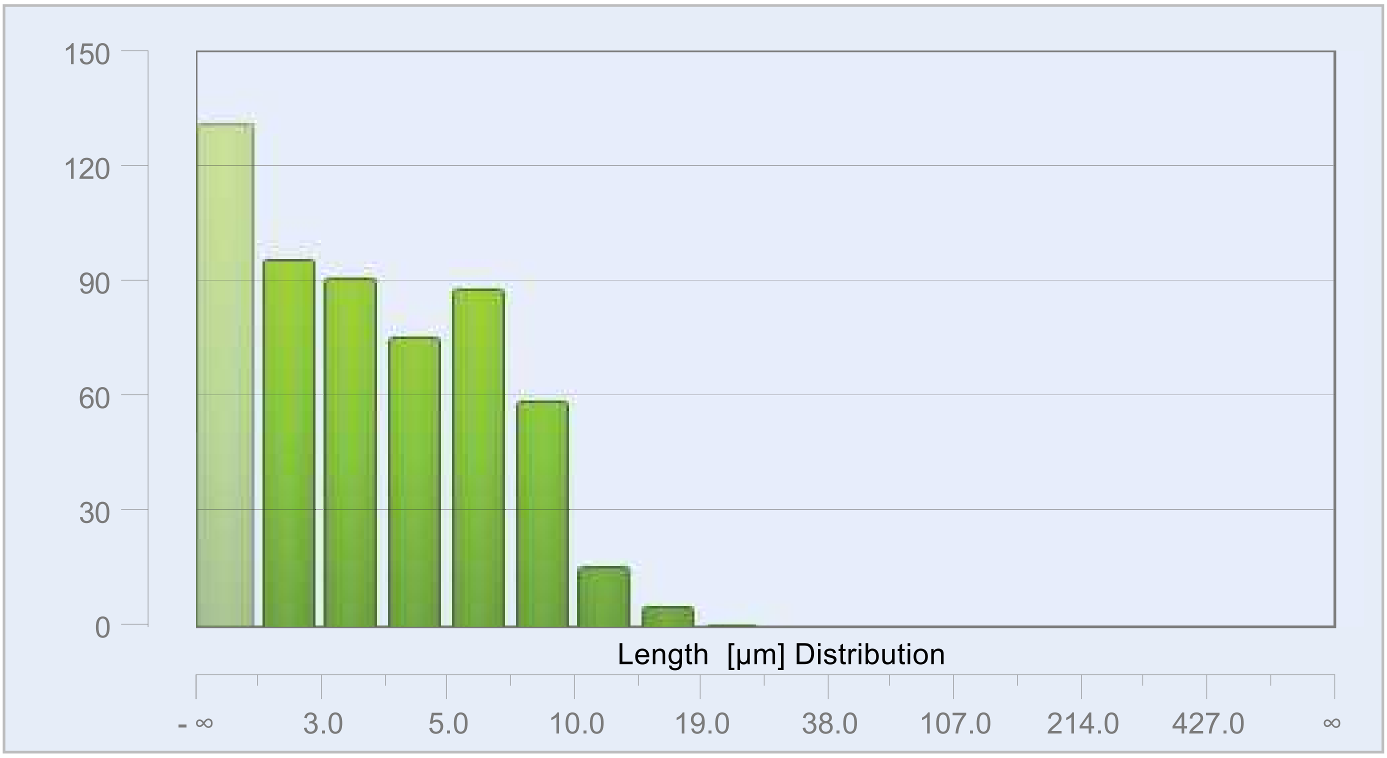

Start	End	Absolute Frequency	Absolute Frequency (accumulated)	Relative Frequency [%]	Relative Frequency (accumulated) [%]
	2.0 µm	131	131	23	23
2.0 µm	3.0 µm	96	227	17	40
3.0 µm	4.0 µm	91	318	16	56
4.0 µm	5.0 µm	76	394	13	70
5.0 µm	7.0 µm	88	482	16	85
7.0 µm	10.0 µm	59	541	10	96
10.0 µm	13.0 µm	16	557	3	99
13.0 µm	19.0 µm	6	563	1	100
19.0 µm	27.0 µm	1	564	0	100
27.0 µm	38.0 µm	0	564	0	100
38.0 µm	75.0 µm	0	564	0	100
75.0 µm	107.0 µm	0	564	0	100
107.0 µm	151.0 µm	0	564	0	100
151.0 µm	214.0 µm	0	564	0	100
214.0 µm	302.0 µm	0	564	0	100
302.0 µm	427.0 µm	0	564	0	100
427.0 µm	600.0 µm	0	564	0	100
600.0 µm		0	564	0	100

5. Single Result 4 (CrCoNi Twins grain size_ASTM 800C 120min_00178)

Mean chord length	4.1 µm
Grain size (ASTM)	12.6
Grain size (G643)	12.5
Grain stretching	94.5 %

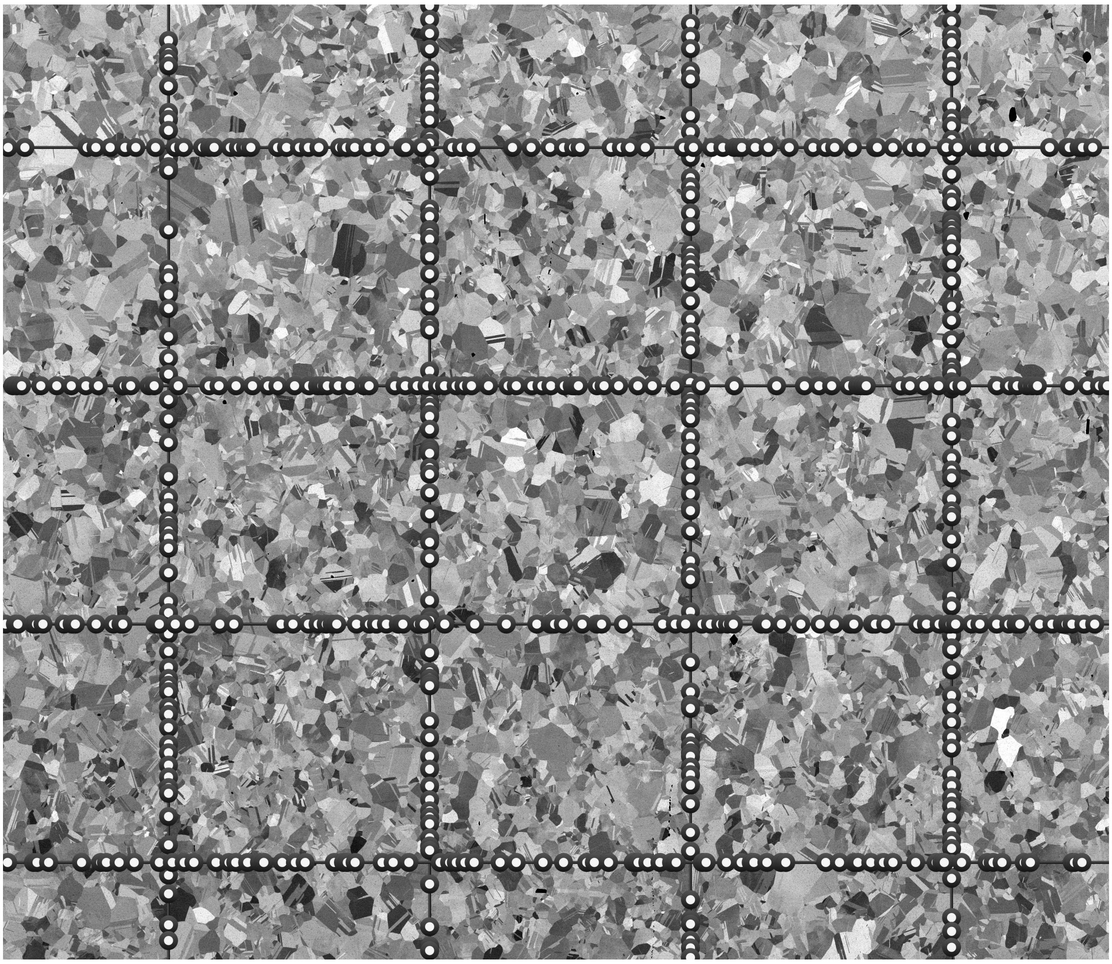

5.1. Statistical Analysis

Statistical Data	Length
Object Count	581
Minimum	0.3 µm
Maximum	17.9 µm
Average	4.1 µm
Standard deviation	2.6 µm
Skewness	0.0
Standard deviation (n-1)	2.6 µm
Variance	7.0 µm²
Variance (n-1)	7.0 µm²
Sum	2'365.9 µm
Sum of squares	13'697.3 µm²
Sum of cubes	103'955.6 µm³

5.1.1. Chord Lenght Distribution

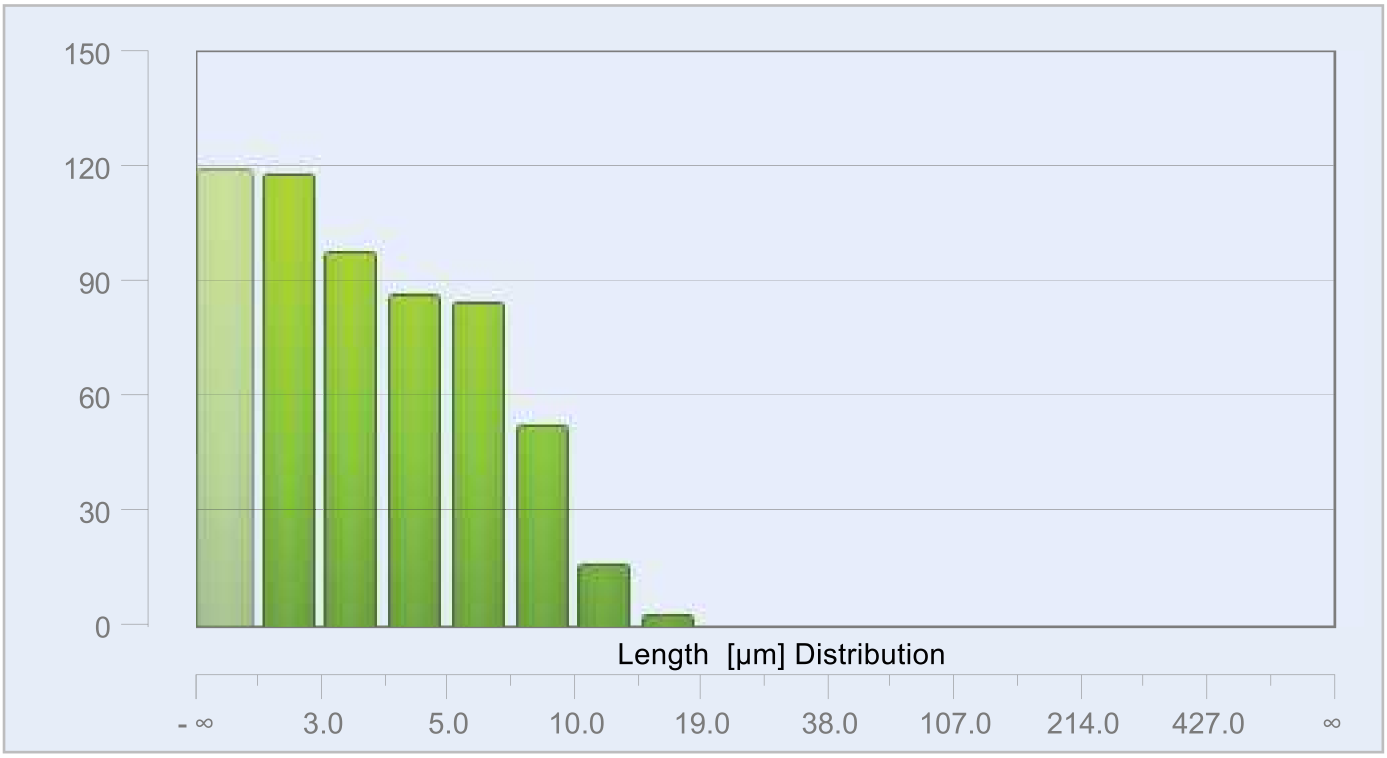

Start	End	Absolute Frequency	Absolute Frequency (accumulated)	Relative Frequency [%]	Relative Frequency (accumulated) [%]
	2.0 µm	119	119	20	20
2.0 µm	3.0 µm	118	237	20	41
3.0 µm	4.0 µm	98	335	17	58
4.0 µm	5.0 µm	87	422	15	73
5.0 µm	7.0 µm	85	507	15	87
7.0 µm	10.0 µm	53	560	9	96
10.0 µm	13.0 µm	17	577	3	99
13.0 µm	19.0 µm	4	581	1	100
19.0 µm	27.0 µm	0	581	0	100
27.0 µm	38.0 µm	0	581	0	100
38.0 µm	75.0 µm	0	581	0	100
75.0 µm	107.0 µm	0	581	0	100
107.0 µm	151.0 µm	0	581	0	100
151.0 µm	214.0 µm	0	581	0	100
214.0 µm	302.0 µm	0	581	0	100
302.0 µm	427.0 µm	0	581	0	100
427.0 µm	600.0 µm	0	581	0	100
600.0 µm		0	581	0	100
