# Supplementary material for: Benchmark dataset of the effect of grain size on strength in the single-phase FCC CrCoNi medium entropy alloy
Source: Data Brief. 2019 Oct 1;27:104592. doi: 10.1016/j.dib.2019.104592 (PMC6812030; doi:10.1016/j.dib.2019.104592)
Supplement: Multimedia component 1 [file mmc1.zip › CrCoNi_1073K_15min/CrCoNi_1073K_15min_c=1.3μm.pdf]

Analysebericht

09.11.2017 20:55:16

powered by imagic.ch

1. cumulative Result 1

Anzahl Bilder	4
Korngröße (ASTM)	16
Korngröße (G643)	15,9
Kornstreckung	85,7 %
Mittlere Sehnenlänge	1,3 µm

2. Single Result 1 (CrCoNi - ASTM E 112_CrCoNi_homogenized_8.1mmSW_800°C_15min_00162)

Mittlere Sehnenlänge	1,4 µm
Korngröße (ASTM)	15,7
Korngröße (G643)	15,6
Kornstreckung	95 %

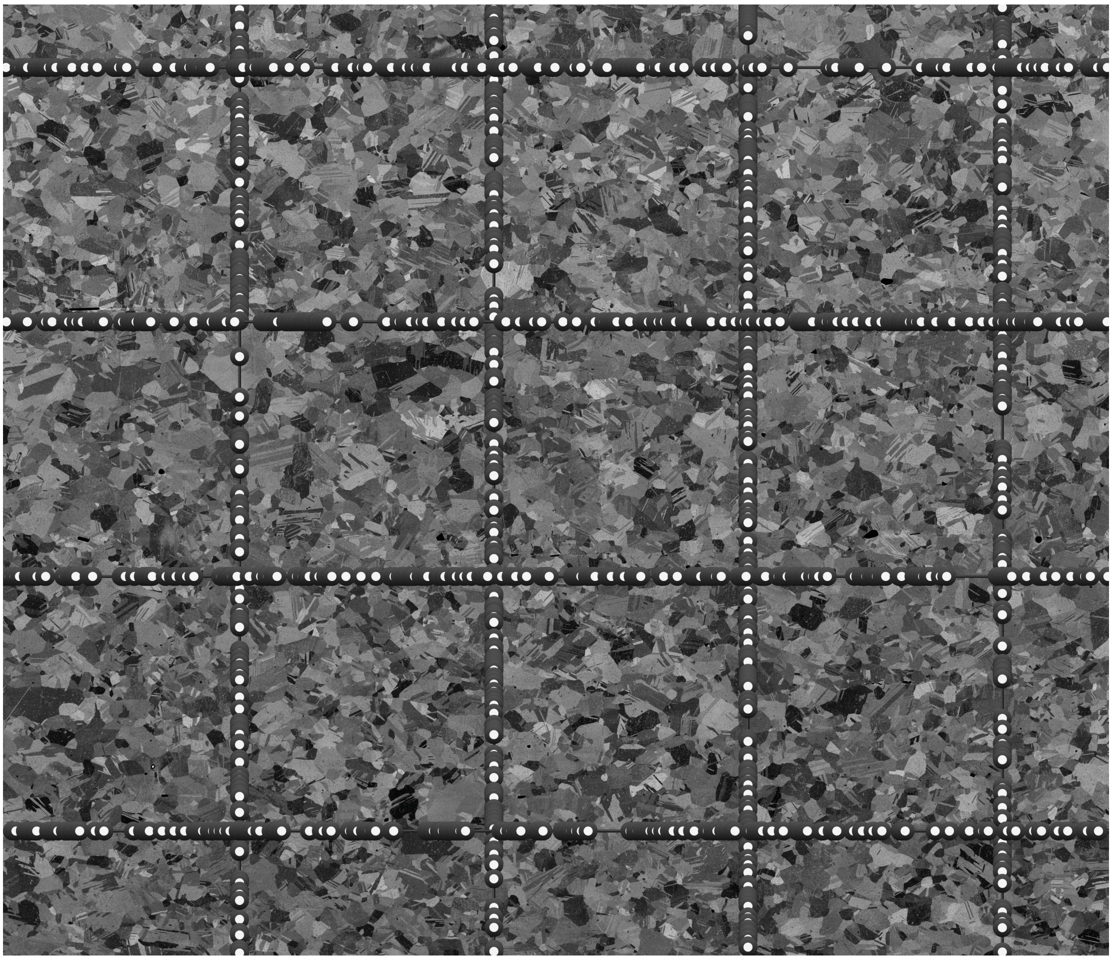

2.1. Statistische Analyse

Statistische Daten	Länge
Anzahl Objekte	1347
Minimum	0,1 µm
Maximum	11,6 µm
Mittelwert	1,4 µm
Standardabweichung	1,5 µm
Schiefe	0,0
Standardabweichung (n-1)	1,5 µm
Varianz	2,1 µm²
Varianz (n-1)	2,1 µm²
Summe	1'886,8 µm

Statistische Daten	Länge
Quadratsumme	5'501,8 µm²
Kubiksumme	25'385,3 µm³

2.1.1. Chord Lenght Distribution

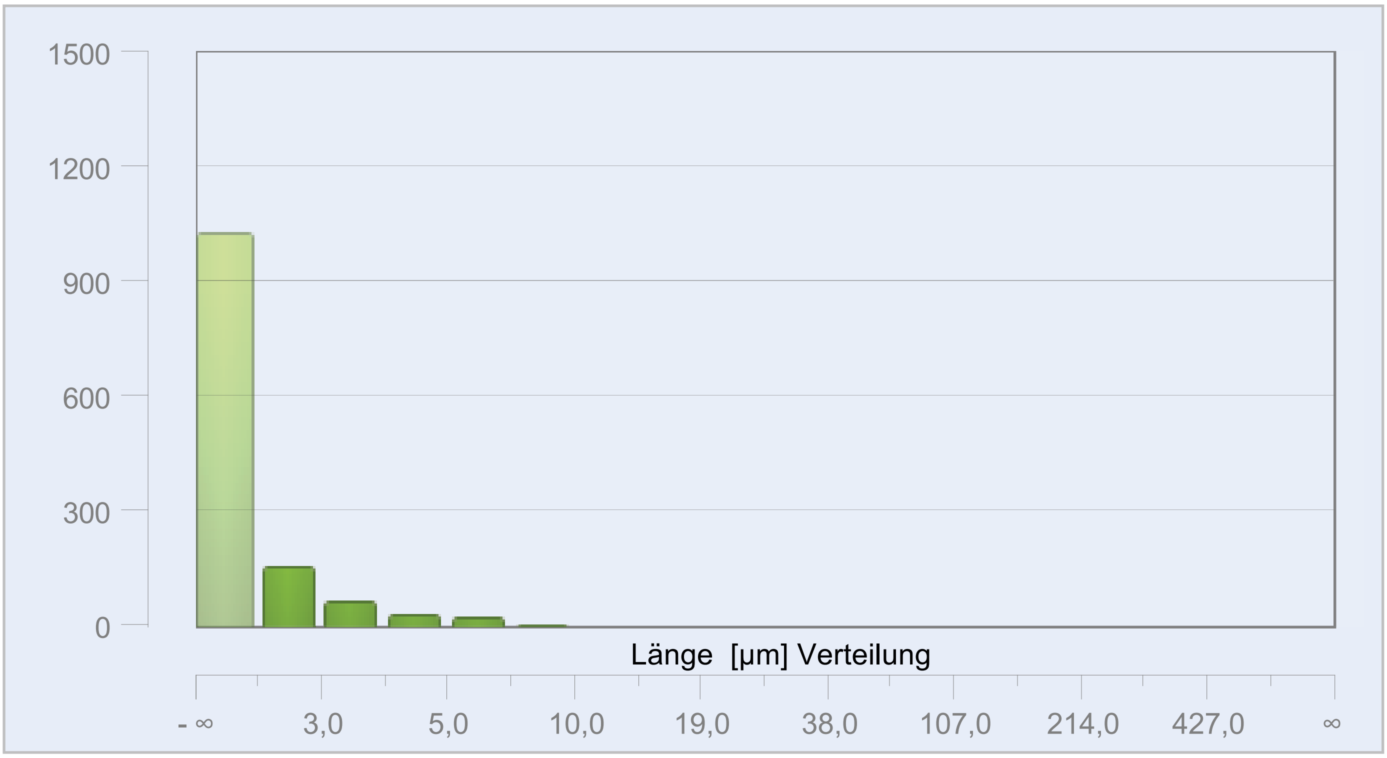

Start	Ende	Absolute Häufigkeit	Absolute Häufigkeit (kumuliert)	Relative Häufigkeit [%]	Relative Häufigkeit (kumuliert) [%]
	2,0 µm	1028	1028	76	76
2,0 µm	3,0 µm	165	1193	12	89
3,0 µm	4,0 µm	75	1268	6	94
4,0 µm	5,0 µm	35	1303	3	97
5,0 µm	7,0 µm	31	1334	2	99
7,0 µm	10,0 µm	12	1346	1	100
10,0 µm	13,0 µm	1	1347	0	100
13,0 µm	19,0 µm	0	1347	0	100
19,0 µm	27,0 µm	0	1347	0	100
27,0 µm	38,0 µm	0	1347	0	100
38,0 µm	75,0 µm	0	1347	0	100
75,0 µm	107,0 µm	0	1347	0	100
107,0 µm	151,0 µm	0	1347	0	100
151,0 µm	214,0 µm	0	1347	0	100
214,0 µm	302,0 µm	0	1347	0	100
302,0 µm	427,0 µm	0	1347	0	100
427,0 µm	600,0 µm	0	1347	0	100
600,0 µm		0	1347	0	100

3. Single Result 2 (CrCoNi - ASTM E 112_CrCoNi_homogenized_8.1mmSW_800°C_15min_00163)

Mittlere Sehnenlänge	1,2 µm
Korngröße (ASTM)	16
Korngröße (G643)	16
Kornstreckung	84,9 %

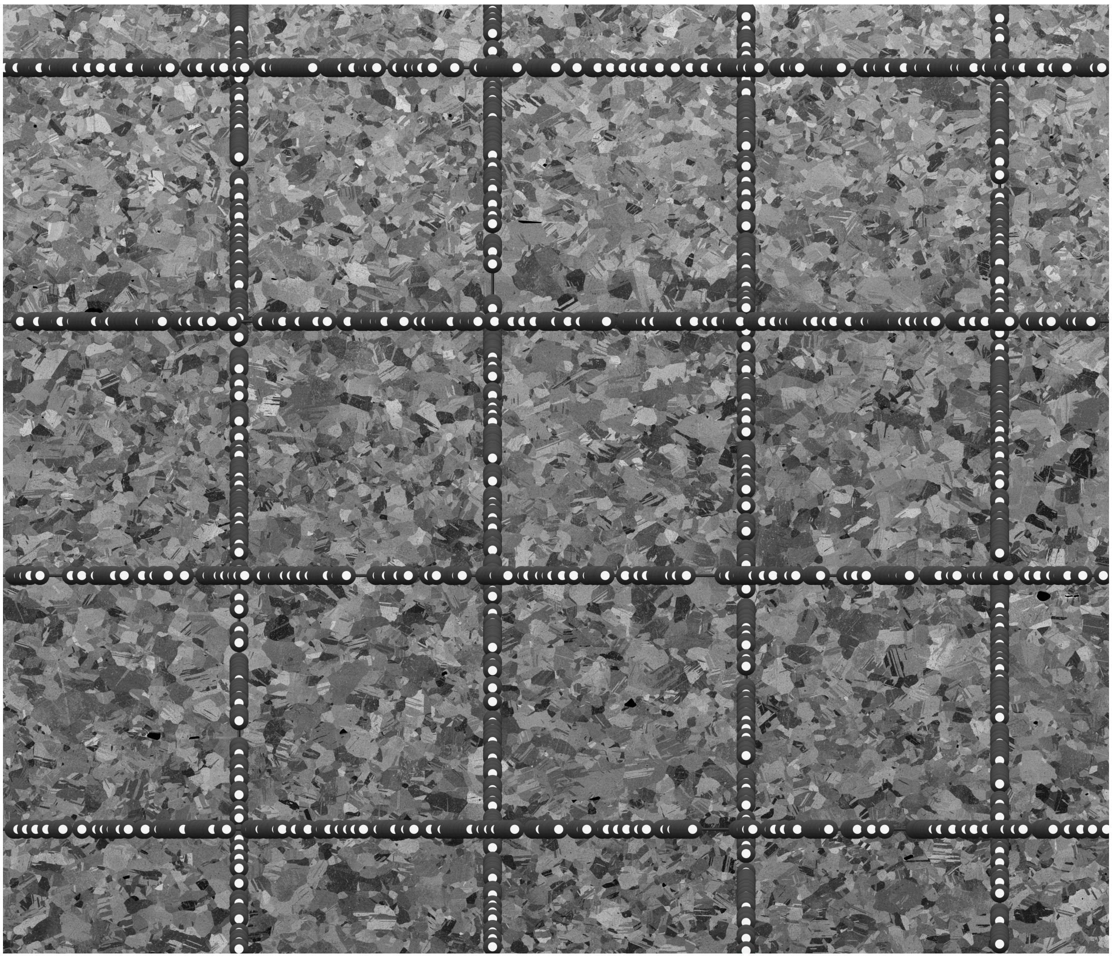

3.1. Statistische Analyse

Statistische Daten	Länge
Anzahl Objekte	1524
Minimum	0,1 µm
Maximum	10,4 µm
Mittelwert	1,2 µm
Standardabweichung	1,2 µm
Schiefe	0,0
Standardabweichung (n-1)	1,2 µm
Varianz	1,3 µm²
Varianz (n-1)	1,3 µm²
Summe	1'891,6 µm
Quadratsumme	4'394,5 µm²
Kubiksumme	15'529,1 µm³

3.1.1. Chord Lenght Distribution

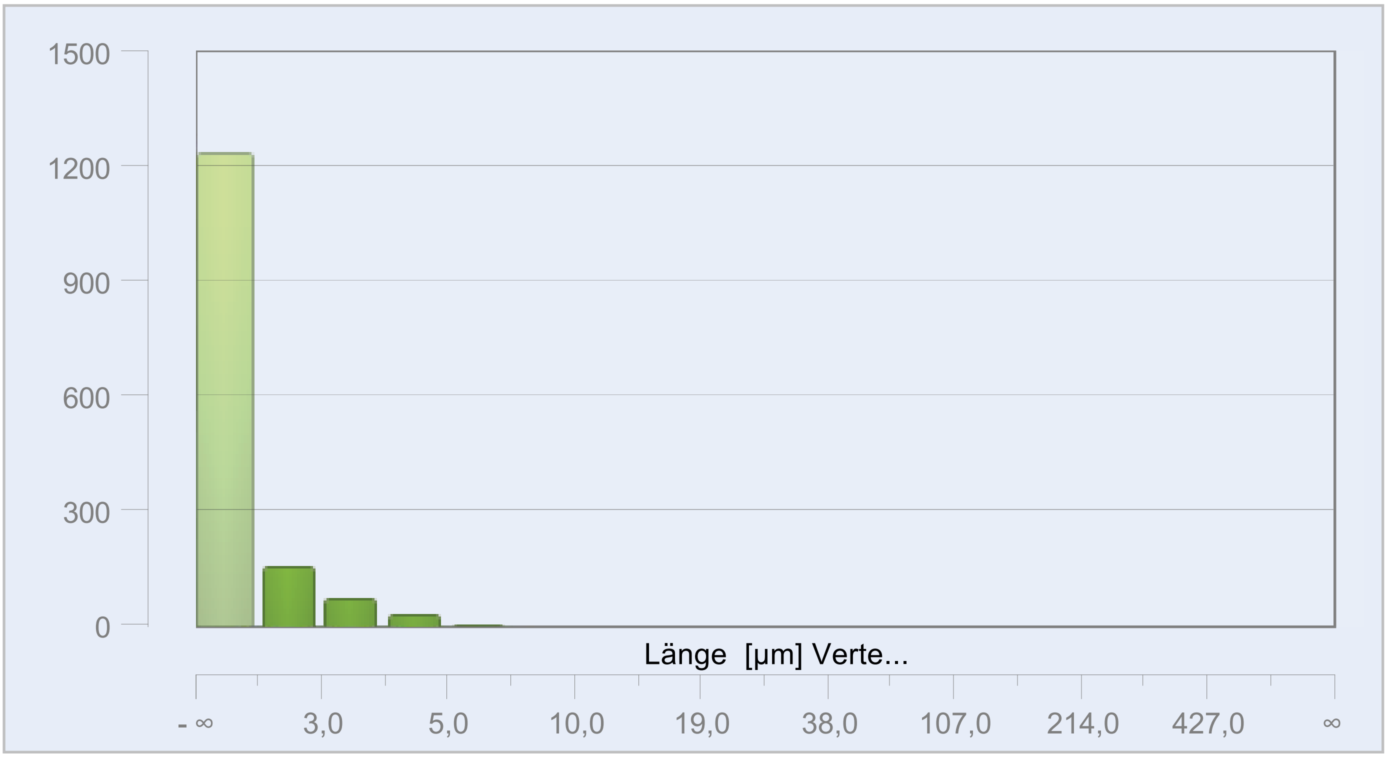

Start	Ende	Absolute Häufigkeit	Absolute Häufigkeit (kumuliert)	Relative Häufigkeit [%]	Relative Häufigkeit (kumuliert) [%]
	2,0 µm	1235	1235	81	81
2,0 µm	3,0 µm	159	1394	10	91
3,0 µm	4,0 µm	78	1472	5	97
4,0 µm	5,0 µm	35	1507	2	99
5,0 µm	7,0 µm	12	1519	1	100
7,0 µm	10,0 µm	4	1523	0	100
10,0 µm	13,0 µm	1	1524	0	100
13,0 µm	19,0 µm	0	1524	0	100
19,0 µm	27,0 µm	0	1524	0	100
27,0 µm	38,0 µm	0	1524	0	100
38,0 µm	75,0 µm	0	1524	0	100
75,0 µm	107,0 µm	0	1524	0	100
107,0 µm	151,0 µm	0	1524	0	100
151,0 µm	214,0 µm	0	1524	0	100
214,0 µm	302,0 µm	0	1524	0	100
302,0 µm	427,0 µm	0	1524	0	100
427,0 µm	600,0 µm	0	1524	0	100
600,0 µm		0	1524	0	100

4. Single Result 3 (CrCoNi - ASTM E 112_CrCoNi_homogenized_8.1mmSW_800°C_15min_00164)

Mittlere Sehnenlänge	1,2 µm
Korngröße (ASTM)	16,1
Korngröße (G643)	16
Kornstreckung	82,9 %

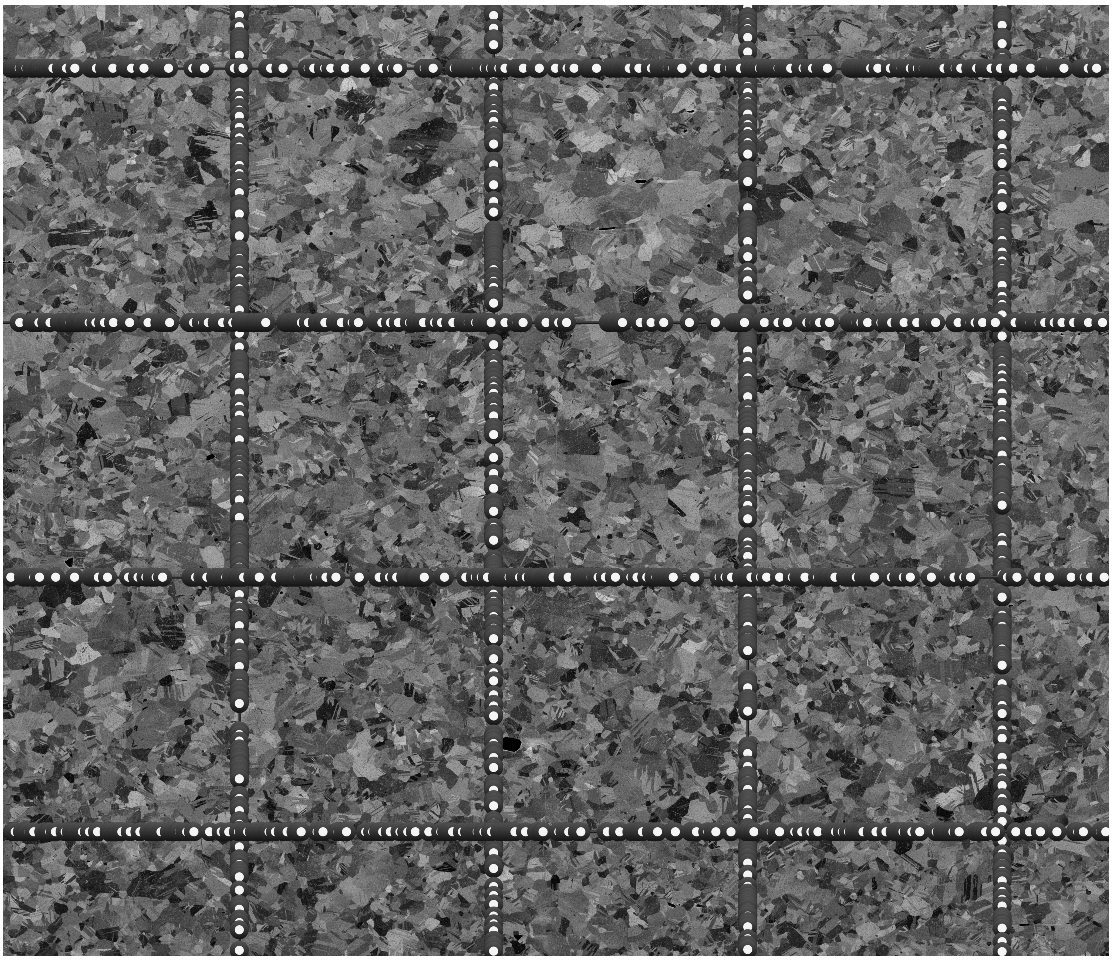

4.1. Statistische Analyse

Statistische Daten	Länge
Anzahl Objekte	1557
Minimum	0,1 µm
Maximum	9,6 µm
Mittelwert	1,2 µm
Standardabweichung	1,2 µm
Schiefe	0,0
Standardabweichung (n-1)	1,2 µm
Varianz	1,4 µm²
Varianz (n-1)	1,4 µm²
Summe	1'892,7 µm
Quadratsumme	4'448,7 µm²
Kubiksumme	15'609,3 µm³

4.1.1. Chord Lenght Distribution

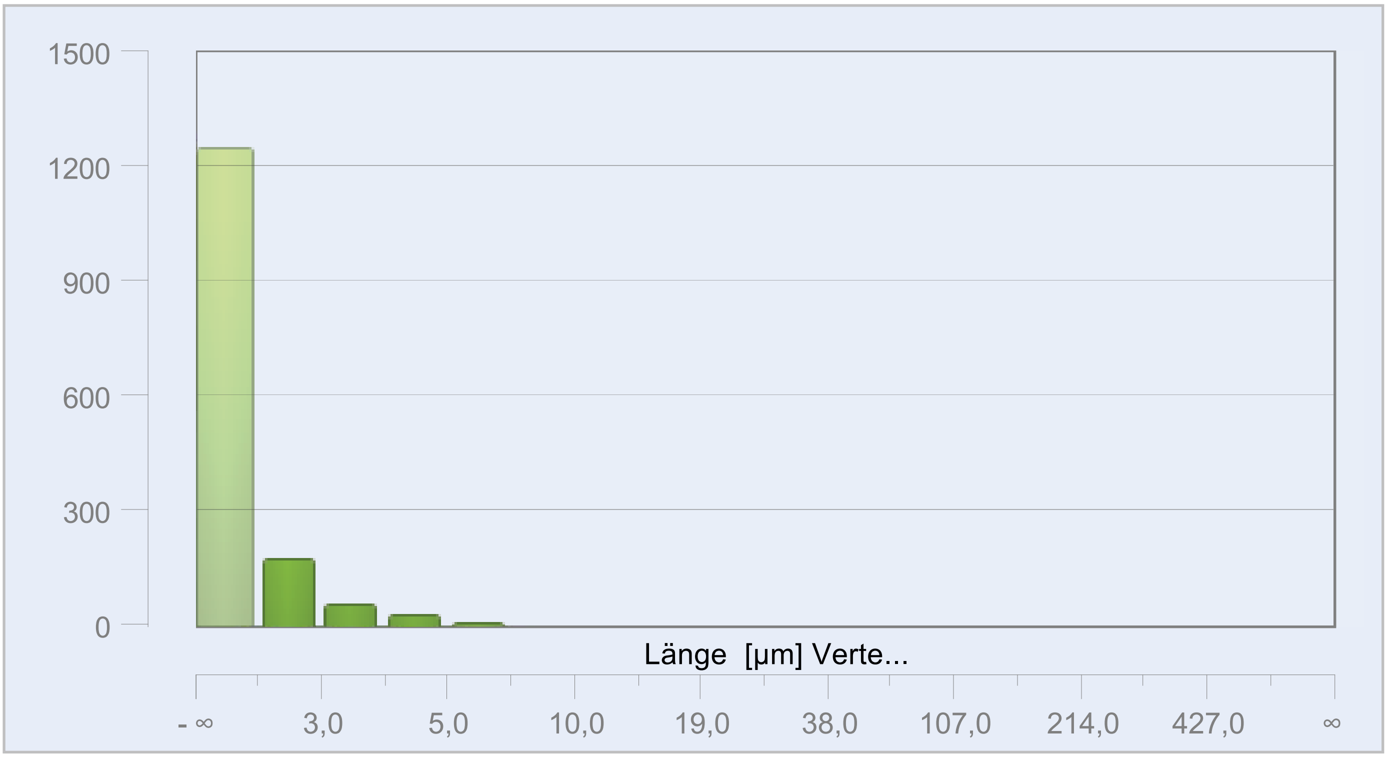

Start	Ende	Absolute Häufigkeit	Absolute Häufigkeit (kumuliert)	Relative Häufigkeit [%]	Relative Häufigkeit (kumuliert) [%]
	2,0 µm	1251	1251	80	80
2,0 µm	3,0 µm	181	1432	12	92
3,0 µm	4,0 µm	68	1500	4	96
4,0 µm	5,0 µm	37	1537	2	99
5,0 µm	7,0 µm	17	1554	1	100
7,0 µm	10,0 µm	3	1557	0	100
10,0 µm	13,0 µm	0	1557	0	100
13,0 µm	19,0 µm	0	1557	0	100
19,0 µm	27,0 µm	0	1557	0	100
27,0 µm	38,0 µm	0	1557	0	100
38,0 µm	75,0 µm	0	1557	0	100
75,0 µm	107,0 µm	0	1557	0	100
107,0 µm	151,0 µm	0	1557	0	100
151,0 µm	214,0 µm	0	1557	0	100
214,0 µm	302,0 µm	0	1557	0	100
302,0 µm	427,0 µm	0	1557	0	100
427,0 µm	600,0 µm	0	1557	0	100
600,0 µm		0	1557	0	100

5. Single Result 4 (CrCoNi - ASTM E 112_CrCoNi_homogenized_8.1mmSW_800°C_15min_00165)

Mittlere Sehnenlänge	1,2 µm
Korngröße (ASTM)	16
Korngröße (G643)	16
Kornstreckung	81,5 %

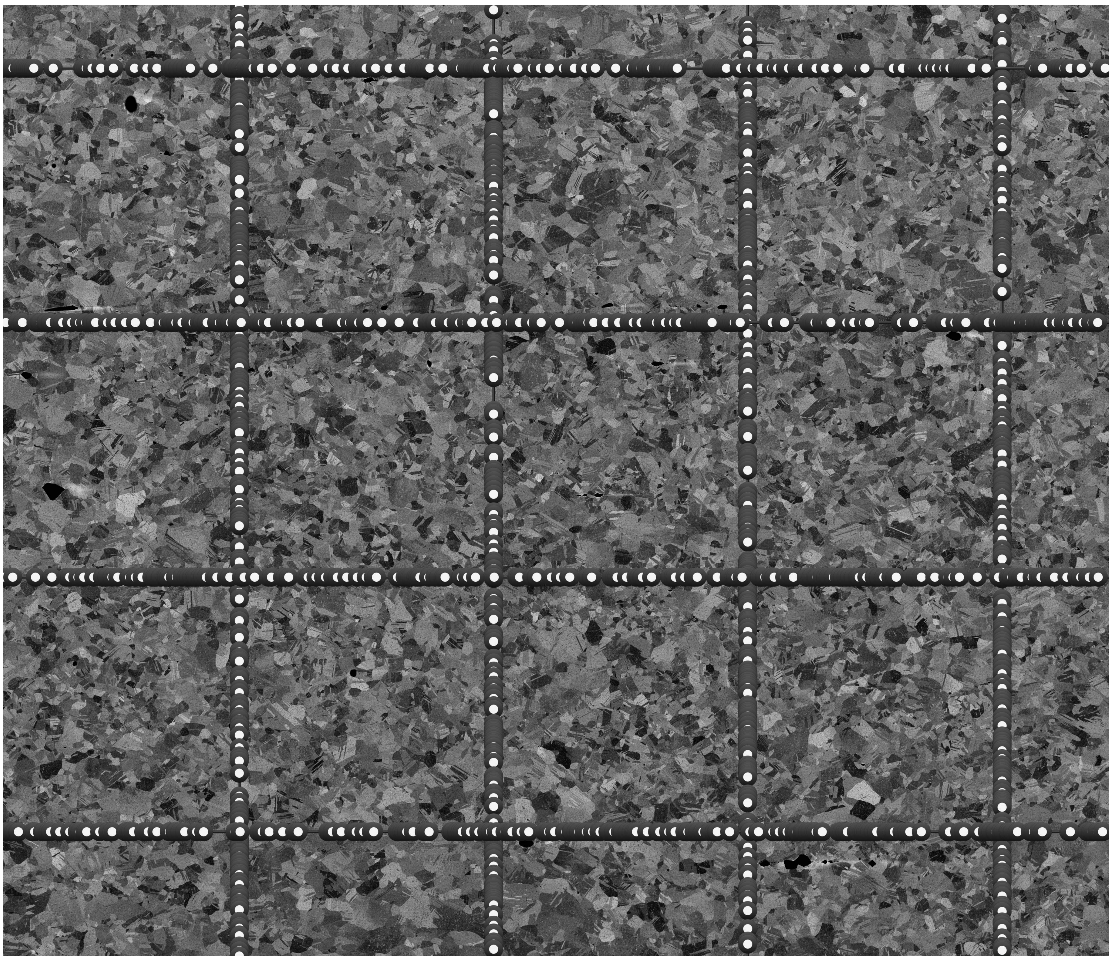

5.1. Statistische Analyse

Statistische Daten	Länge
Anzahl Objekte	1527
Minimum	0,1 µm
Maximum	11,0 µm
Mittelwert	1,2 µm
Standardabweichung	1,2 µm
Schiefe	0,0
Standardabweichung (n-1)	1,2 µm
Varianz	1,4 µm²
Varianz (n-1)	1,4 µm²
Summe	1'892,7 µm
Quadratsumme	4'449,1 µm²
Kubiksumme	16'208,0 µm³

5.1.1. Chord Lenght Distribution

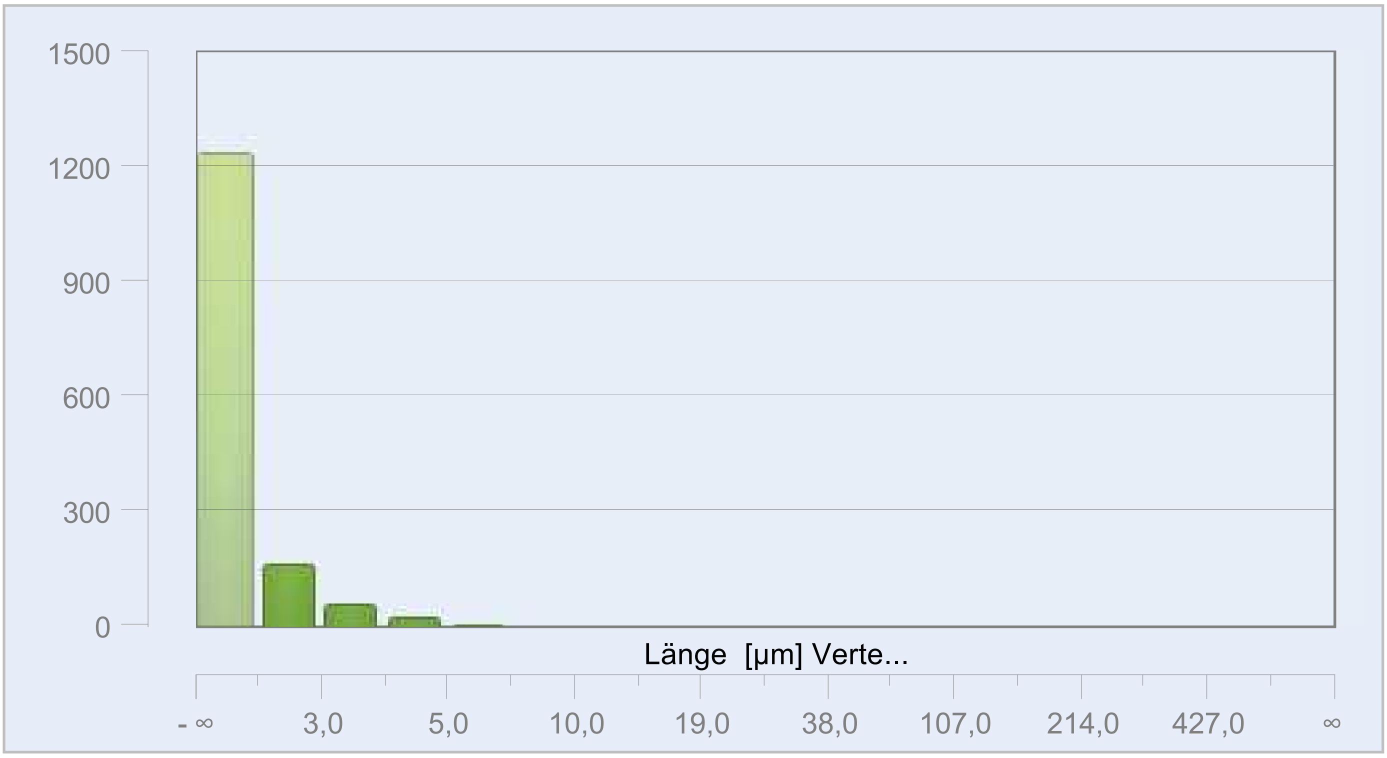

Start	Ende	Absolute Häufigkeit	Absolute Häufigkeit (kumuliert)	Relative Häufigkeit [%]	Relative Häufigkeit (kumuliert) [%]
	2,0 µm	1235	1235	81	81
2,0 µm	3,0 µm	172	1407	11	92
3,0 µm	4,0 µm	69	1476	5	97
4,0 µm	5,0 µm	31	1507	2	99
5,0 µm	7,0 µm	13	1520	1	100
7,0 µm	10,0 µm	6	1526	0	100
10,0 µm	13,0 µm	1	1527	0	100
13,0 µm	19,0 µm	0	1527	0	100
19,0 µm	27,0 µm	0	1527	0	100
27,0 µm	38,0 µm	0	1527	0	100
38,0 µm	75,0 µm	0	1527	0	100
75,0 µm	107,0 µm	0	1527	0	100
107,0 µm	151,0 µm	0	1527	0	100
151,0 µm	214,0 µm	0	1527	0	100
214,0 µm	302,0 µm	0	1527	0	100
302,0 µm	427,0 µm	0	1527	0	100
427,0 µm	600,0 µm	0	1527	0	100
600,0 µm		0	1527	0	100
