# Supplementary material for: Benchmark dataset of the effect of grain size on strength in the single-phase FCC CrCoNi medium entropy alloy
Source: Data Brief. 2019 Oct 1;27:104592. doi: 10.1016/j.dib.2019.104592 (PMC6812030; doi:10.1016/j.dib.2019.104592)
Supplement: Multimedia component 1 [file mmc1.zip › CrCoNi_1073K_15min/CrCoNi_1073K_15min_d=3.2μm.pdf]

Analysebericht

Aug 30, 2017 5:00:02 PM

powered by imagic.ch

1. cumulative Result 1

Number of images	3
Grain size (ASTM)	13.3
Grain size (G643)	13.3
Grain stretching	82.9 %
Mean chord length	3.1 µm

2. Single Result 1 (CrCoNi Twins grain size_ASTM 800C 15min_00215)

Mean chord length	3.1 µm
Grain size (ASTM)	13.4
Grain size (G643)	13.3
Grain stretching	83.7 %

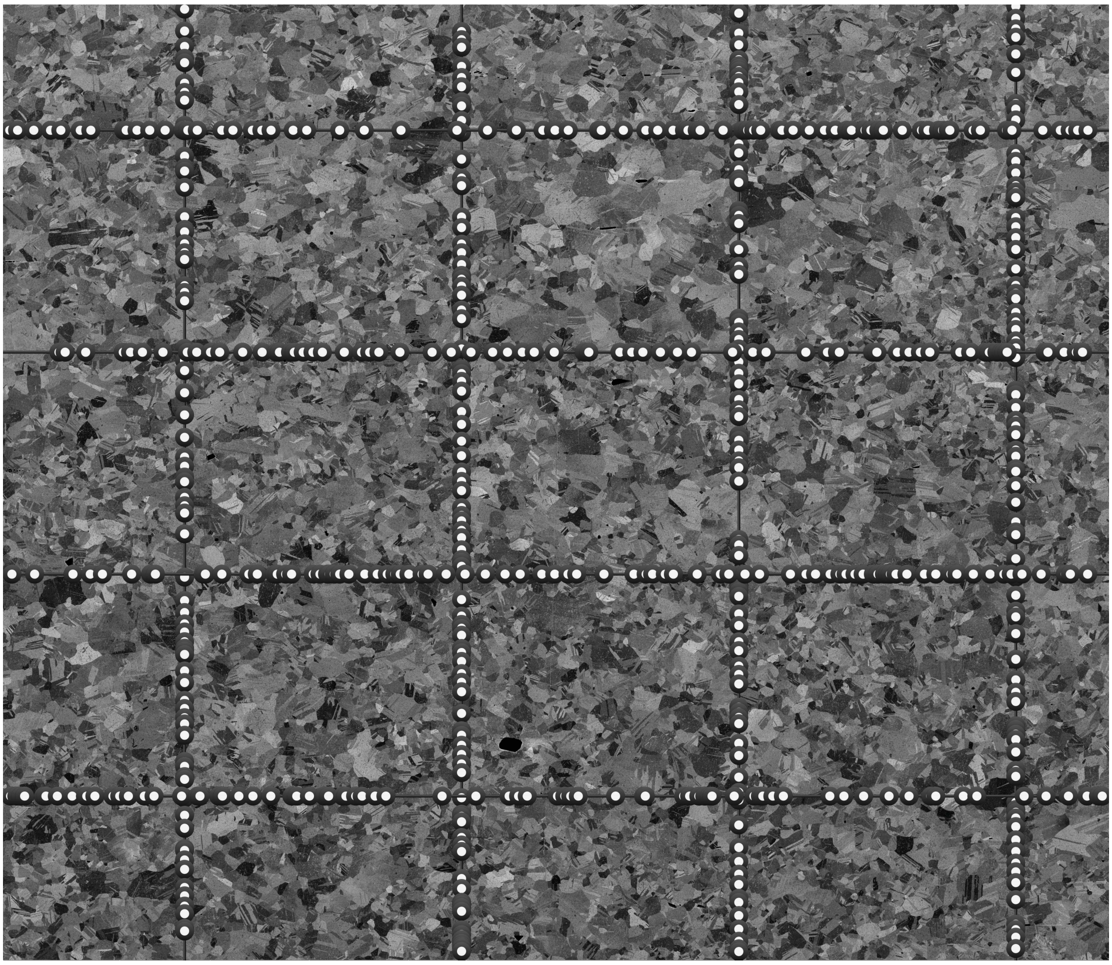

2.1. Statistical Analysis

Statistical Data	Length
Object Count	610
Minimum	0.3 µm
Maximum	13.4 µm
Average	3.1 µm
Standard deviation	2.1 µm
Skewness	0.0
Standard deviation (n-1)	2.1 µm
Variance	4.5 µm²
Variance (n-1)	4.5 µm²
Sum	1'894.7 µm

Statistical Data	Length
Sum of squares	8'635.4 µm²
Sum of cubes	52'985.6 µm³

2.1.1. Chord Lenght Distribution

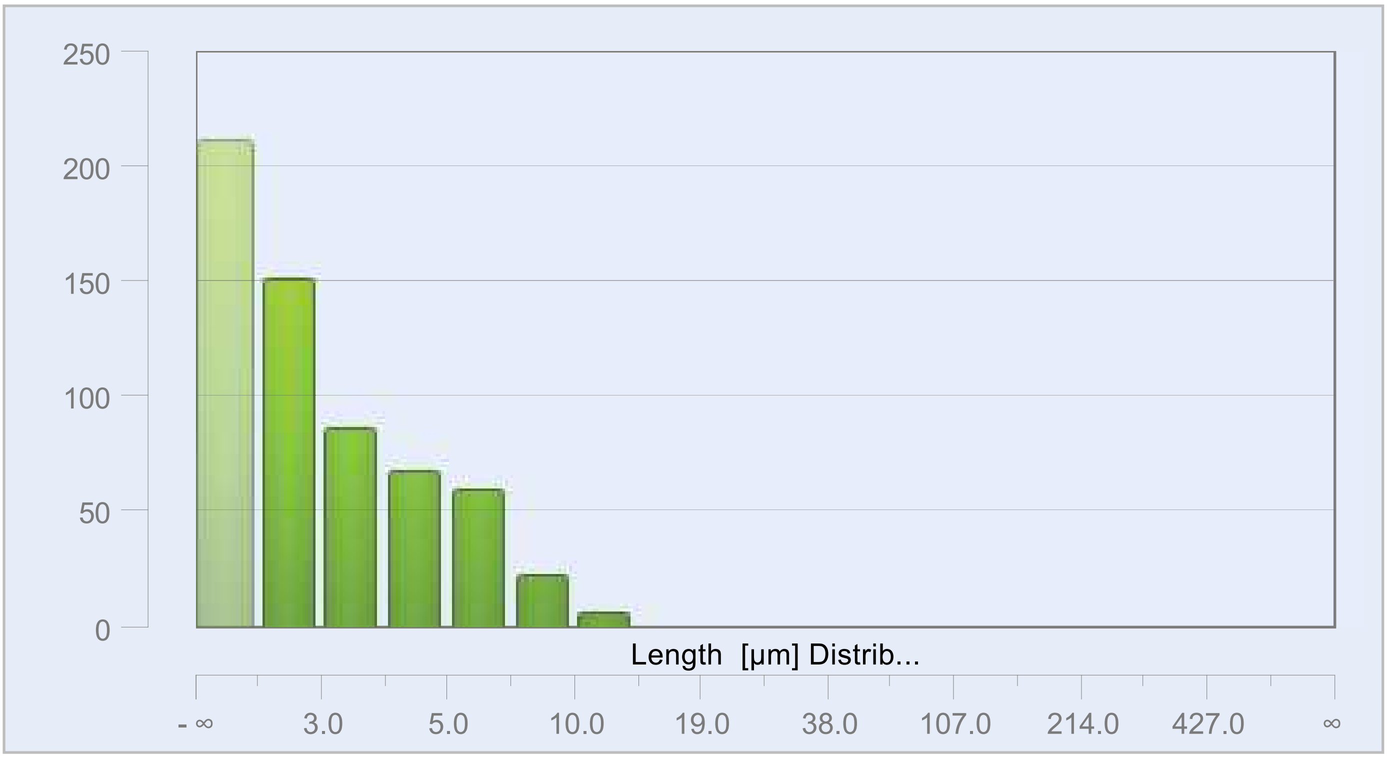

Start	End	Absolute Frequency	Absolute Frequency (accumulated)	Relative Frequency [%]	Relative Frequency (accumulated) [%]
	2.0 µm	211	211	35	35
2.0 µm	3.0 µm	151	362	25	59
3.0 µm	4.0 µm	87	449	14	74
4.0 µm	5.0 µm	68	517	11	85
5.0 µm	7.0 µm	60	577	10	95
7.0 µm	10.0 µm	24	601	4	99
10.0 µm	13.0 µm	8	609	1	100
13.0 µm	19.0 µm	1	610	0	100
19.0 µm	27.0 µm	0	610	0	100
27.0 µm	38.0 µm	0	610	0	100
38.0 µm	75.0 µm	0	610	0	100
75.0 µm	107.0 µm	0	610	0	100
107.0 µm	151.0 µm	0	610	0	100
151.0 µm	214.0 µm	0	610	0	100
214.0 µm	302.0 µm	0	610	0	100
302.0 µm	427.0 µm	0	610	0	100
427.0 µm	600.0 µm	0	610	0	100
600.0 µm		0	610	0	100

3. Single Result 2 (CrCoNi Twins grain size_ASTM 800C 15min_00216)

Mean chord length	2.8 µm
Grain size (ASTM)	13.7
Grain size (G643)	13.6
Grain stretching	87.2 %

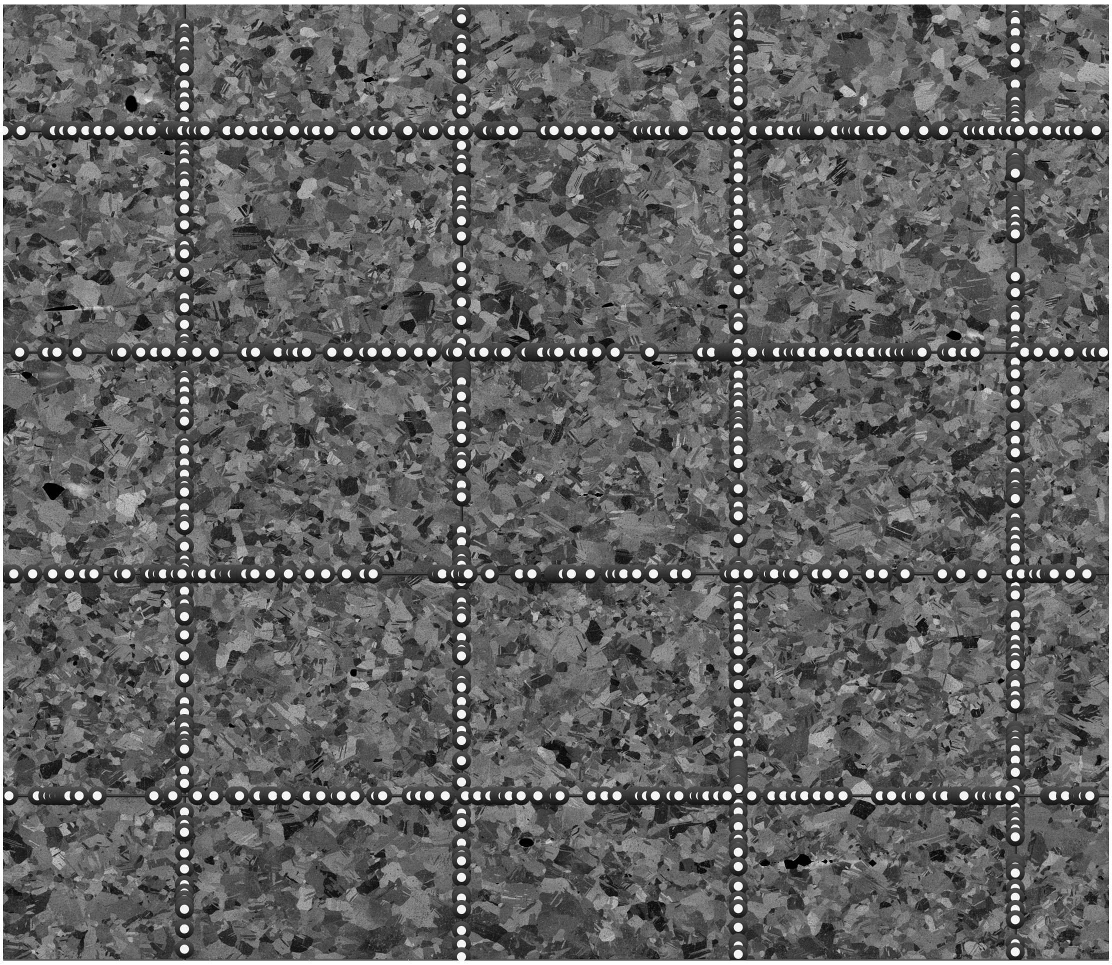

3.1. Statistical Analysis

Statistical Data	Length
Object Count	677
Minimum	0.2 µm
Maximum	14.6 µm
Average	2.8 µm
Standard deviation	1.8 µm
Skewness	0.0
Standard deviation (n-1)	1.8 µm
Variance	3.1 µm²
Variance (n-1)	3.1 µm²
Sum	1'895.7 µm
Sum of squares	7'387.8 µm²
Sum of cubes	39'269.6 µm³

3.1.1. Chord Lenght Distribution

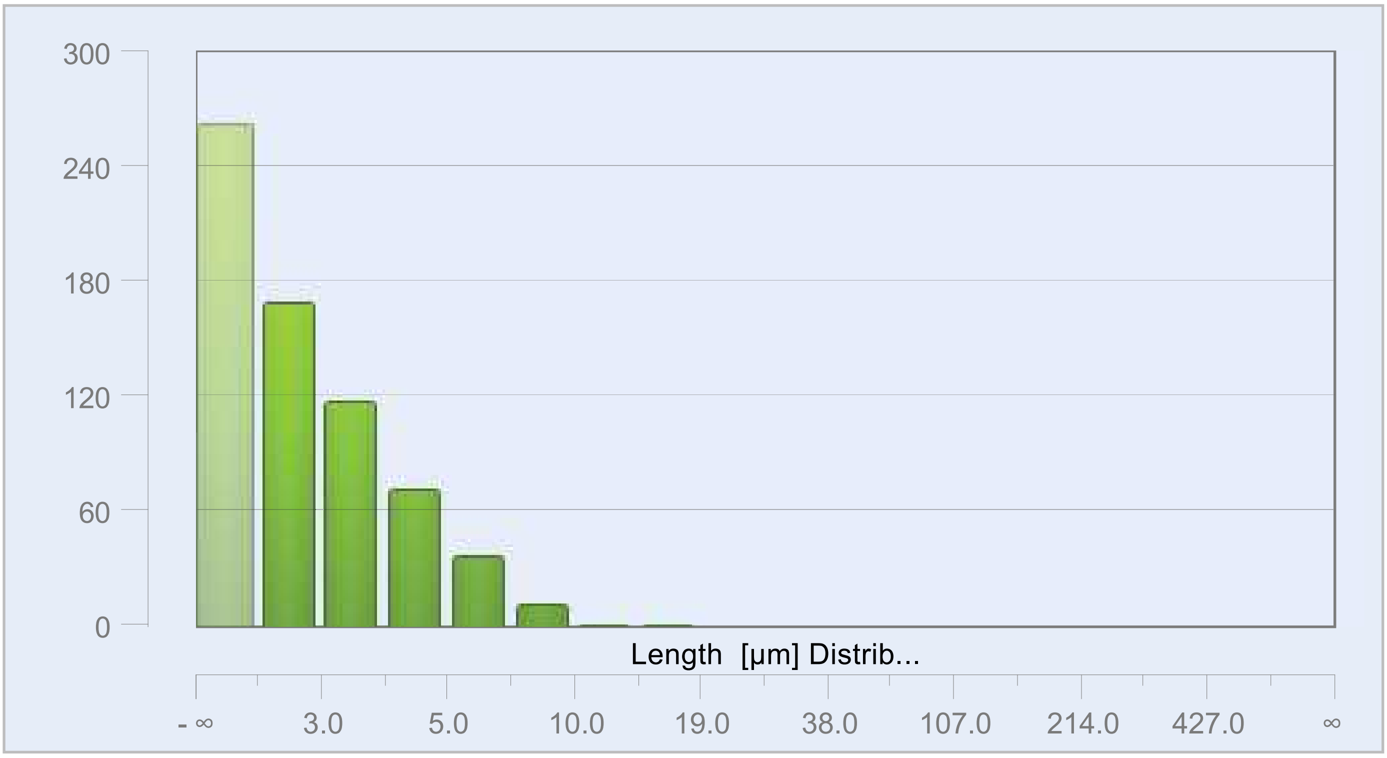

Start	End	Absolute Frequency	Absolute Frequency (accumulated)	Relative Frequency [%]	Relative Frequency (accumulated) [%]
	2.0 µm	262	262	39	39
2.0 µm	3.0 µm	169	431	25	64
3.0 µm	4.0 µm	118	549	17	81
4.0 µm	5.0 µm	73	622	11	92
5.0 µm	7.0 µm	38	660	6	97
7.0 µm	10.0 µm	13	673	2	99
10.0 µm	13.0 µm	2	675	0	100
13.0 µm	19.0 µm	2	677	0	100
19.0 µm	27.0 µm	0	677	0	100
27.0 µm	38.0 µm	0	677	0	100
38.0 µm	75.0 µm	0	677	0	100
75.0 µm	107.0 µm	0	677	0	100
107.0 µm	151.0 µm	0	677	0	100
151.0 µm	214.0 µm	0	677	0	100
214.0 µm	302.0 µm	0	677	0	100
302.0 µm	427.0 µm	0	677	0	100
427.0 µm	600.0 µm	0	677	0	100
600.0 µm		0	677	0	100

4. Single Result 3 (CrCoNi Twins grain size_ASTM 800C 15min_00217)

Mean chord length	3.6 µm
Grain size (ASTM)	12.9
Grain size (G643)	12.9
Grain stretching	76.9 %

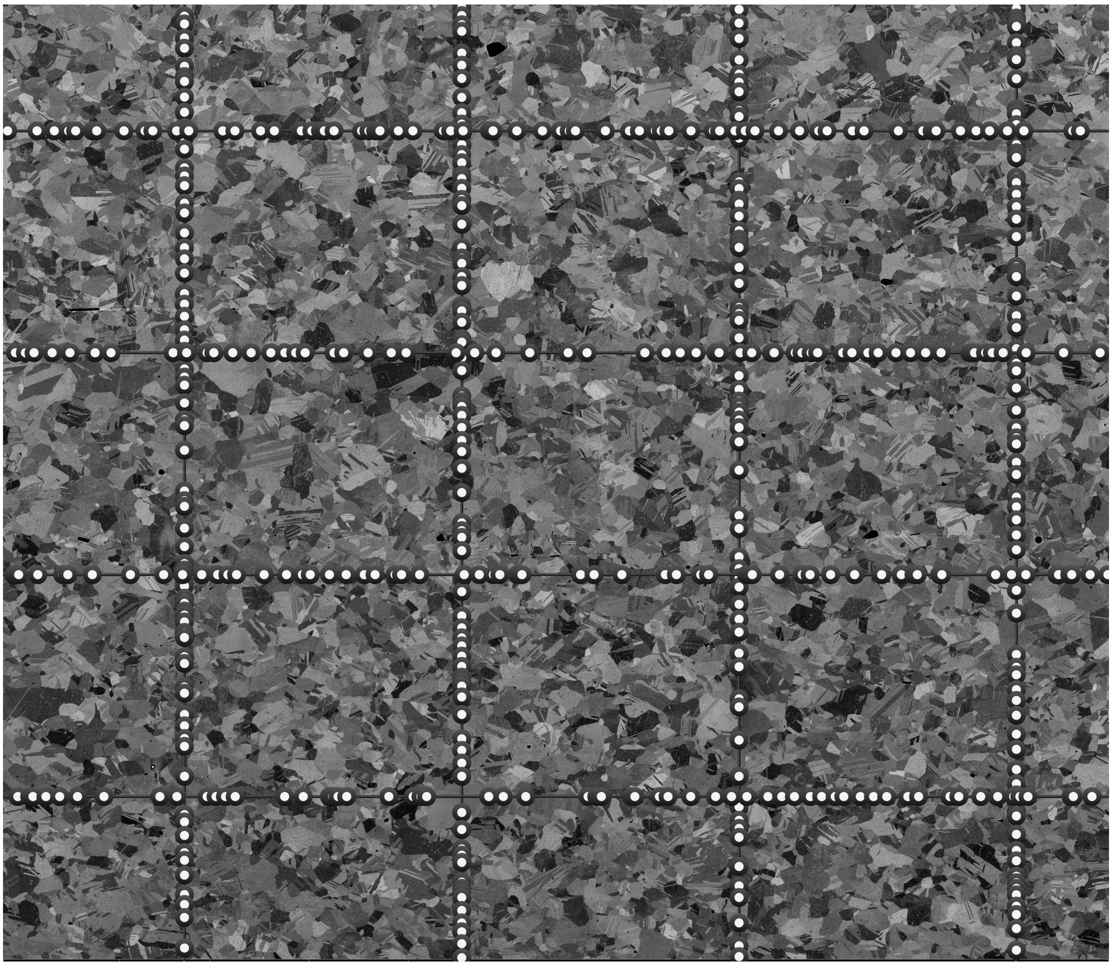

4.1. Statistical Analysis

Statistical Data	Length
Object Count	525
Minimum	0.2 µm
Maximum	14.3 µm
Average	3.6 µm
Standard deviation	2.3 µm
Skewness	0.0
Standard deviation (n-1)	2.3 µm
Variance	5.5 µm²
Variance (n-1)	5.5 µm²
Sum	1'896.7 µm
Sum of squares	9'739.5 µm²
Sum of cubes	67'231.3 µm³

4.1.1. Chord Lenght Distribution

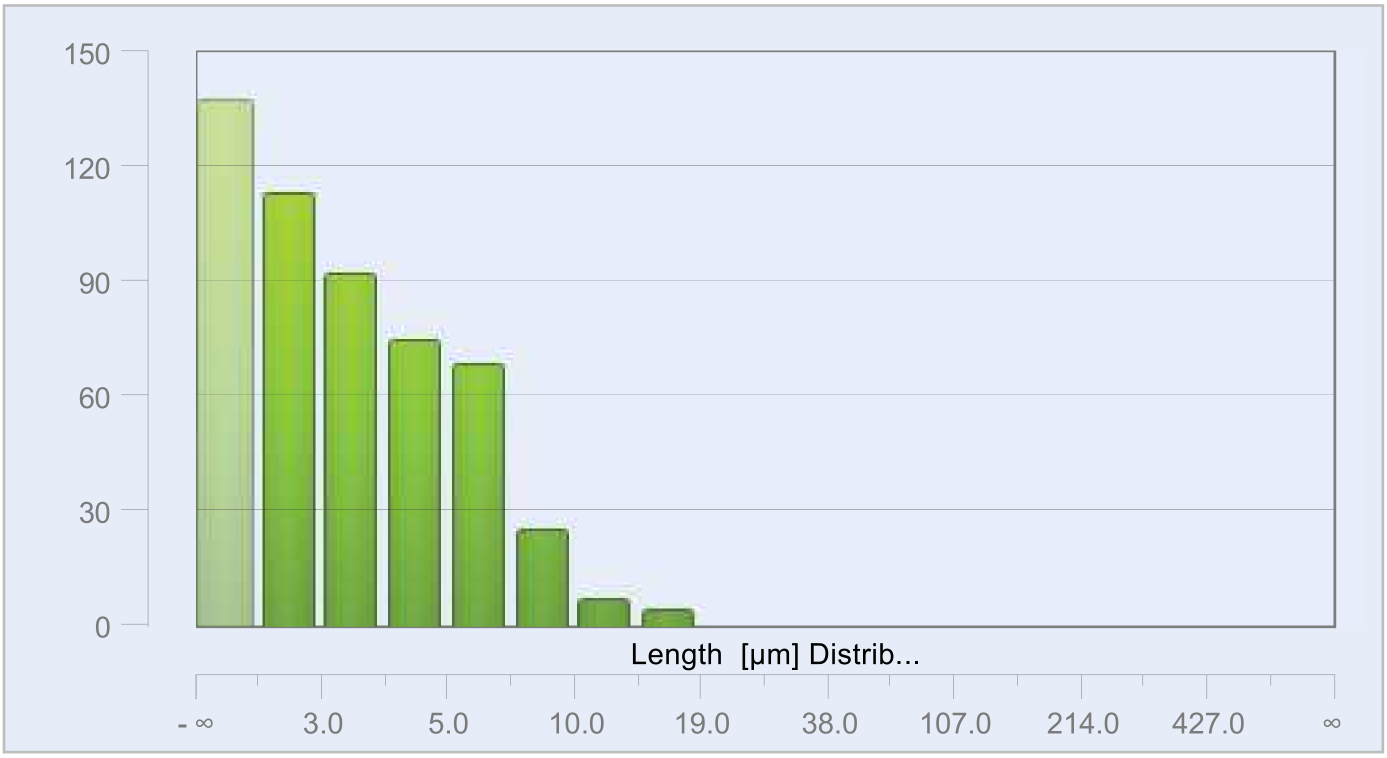

Start	End	Absolute Frequency	Absolute Frequency (accumulated)	Relative Frequency [%]	Relative Frequency (accumulated) [%]
	2.0 µm	137	137	26	26
2.0 µm	3.0 µm	113	250	22	48
3.0 µm	4.0 µm	92	342	18	65
4.0 µm	5.0 µm	75	417	14	79
5.0 µm	7.0 µm	69	486	13	93
7.0 µm	10.0 µm	26	512	5	98
10.0 µm	13.0 µm	8	520	2	99
13.0 µm	19.0 µm	5	525	1	100
19.0 µm	27.0 µm	0	525	0	100
27.0 µm	38.0 µm	0	525	0	100
38.0 µm	75.0 µm	0	525	0	100
75.0 µm	107.0 µm	0	525	0	100
107.0 µm	151.0 µm	0	525	0	100
151.0 µm	214.0 µm	0	525	0	100
214.0 µm	302.0 µm	0	525	0	100
302.0 µm	427.0 µm	0	525	0	100
427.0 µm	600.0 µm	0	525	0	100
600.0 µm		0	525	0	100
