# Supplementary material for: Benchmark dataset of the effect of grain size on strength in the single-phase FCC CrCoNi medium entropy alloy
Source: Data Brief. 2019 Oct 1;27:104592. doi: 10.1016/j.dib.2019.104592 (PMC6812030; doi:10.1016/j.dib.2019.104592)
Supplement: Multimedia component 1 [file mmc1.zip › CrCoNi_1073K_180min/CrCoNi_1073K_180min_c=2.3μm.pdf]

Analysebericht

11.11.2017 18:36:29

powered by imagic.ch

1. cumulative Result 1

Anzahl Bilder	4
Korngröße (ASTM)	14,2
Korngröße (G643)	14,2
Kornstreckung	90,1 %
Mittlere Sehnenlänge	2,3 µm

2. Single Result 1 (CrCoNi - ASTM E 112_CrCoNi_homogenized_8.1mmSW_800°C_180min_00174)

Mittlere Sehnenlänge	2,2 µm
Korngröße (ASTM)	14,4
Korngröße (G643)	14,4
Kornstreckung	97,2 %

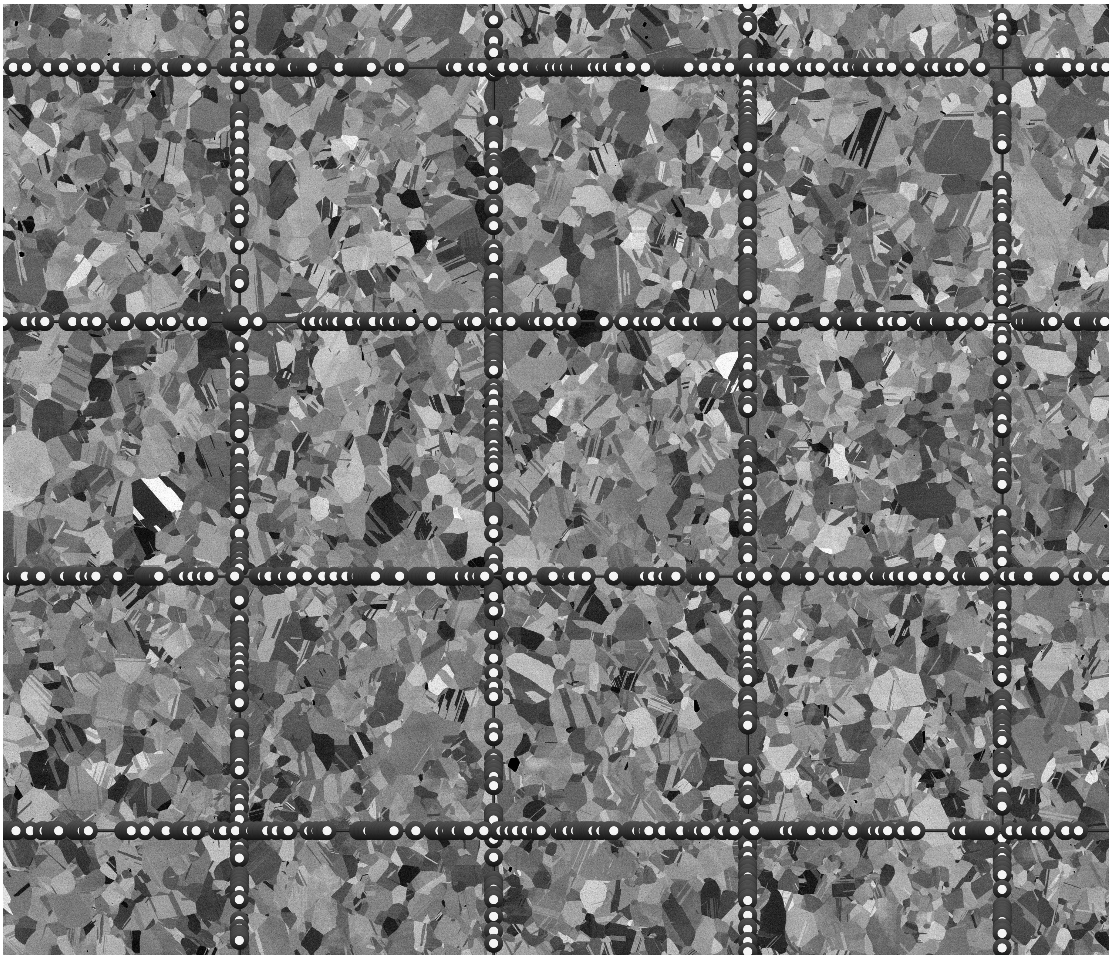

2.1. Statistische Analyse

Statistische Daten	Länge
Anzahl Objekte	1086
Minimum	0,1 µm
Maximum	14,3 µm
Mittelwert	2,2 µm
Standardabweichung	2,1 µm
Schiefe	0,0
Standardabweichung (n-1)	2,1 µm
Varianz	4,5 µm²
Varianz (n-1)	4,5 µm²
Summe	2'362,2 µm

Statistische Daten	Länge
Quadratsumme	10'016,8 µm²
Kubiksumme	63'389,5 µm³

2.1.1. Chord Lenght Distribution

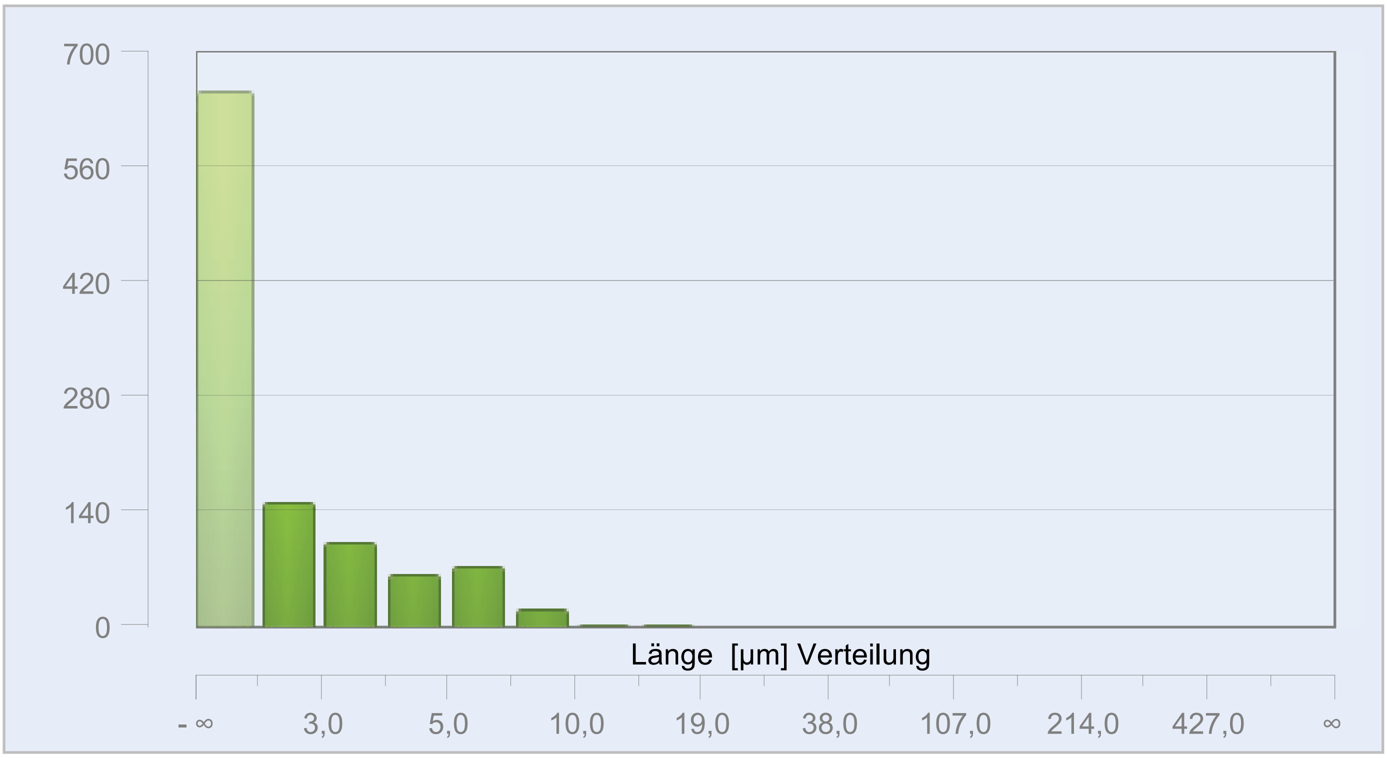

Start	Ende	Absolute Häufigkeit	Absolute Häufigkeit (kumuliert)	Relative Häufigkeit [%]	Relative Häufigkeit (kumuliert) [%]
	2,0 µm	651	651	60	60
2,0 µm	3,0 µm	154	805	14	74
3,0 µm	4,0 µm	105	910	10	84
4,0 µm	5,0 µm	66	976	6	90
5,0 µm	7,0 µm	75	1051	7	97
7,0 µm	10,0 µm	24	1075	2	99
10,0 µm	13,0 µm	6	1081	1	100
13,0 µm	19,0 µm	5	1086	0	100
19,0 µm	27,0 µm	0	1086	0	100
27,0 µm	38,0 µm	0	1086	0	100
38,0 µm	75,0 µm	0	1086	0	100
75,0 µm	107,0 µm	0	1086	0	100
107,0 µm	151,0 µm	0	1086	0	100
151,0 µm	214,0 µm	0	1086	0	100
214,0 µm	302,0 µm	0	1086	0	100
302,0 µm	427,0 µm	0	1086	0	100
427,0 µm	600,0 µm	0	1086	0	100
600,0 µm		0	1086	0	100

3. Single Result 2 (CrCoNi - ASTM E 112_CrCoNi_homogenized_8.1mmSW_800°C_180min_00175)

Mittlere Sehnenlänge	2,3 µm
Korngröße (ASTM)	14,2
Korngröße (G643)	14,2
Kornstreckung	83,1 %

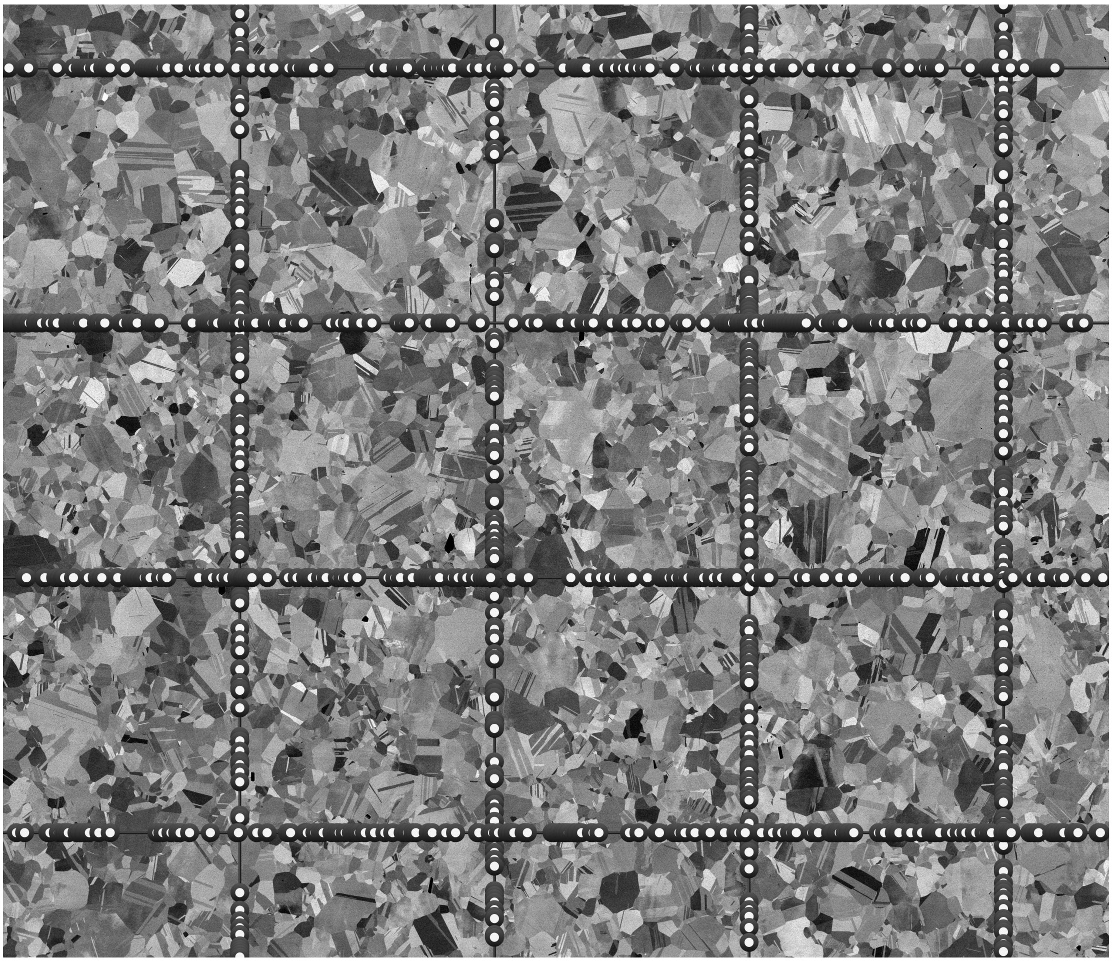

3.1. Statistische Analyse

Statistische Daten	Länge
Anzahl Objekte	1026
Minimum	0,1 µm
Maximum	21,5 µm
Mittelwert	2,3 µm
Standardabweichung	2,2 µm
Schiefe	0,0
Standardabweichung (n-1)	2,2 µm
Varianz	4,9 µm²
Varianz (n-1)	4,9 µm²
Summe	2'363,4 µm
Quadratsumme	10'513,4 µm²
Kubiksumme	76'292,2 µm³

3.1.1. Chord Lenght Distribution

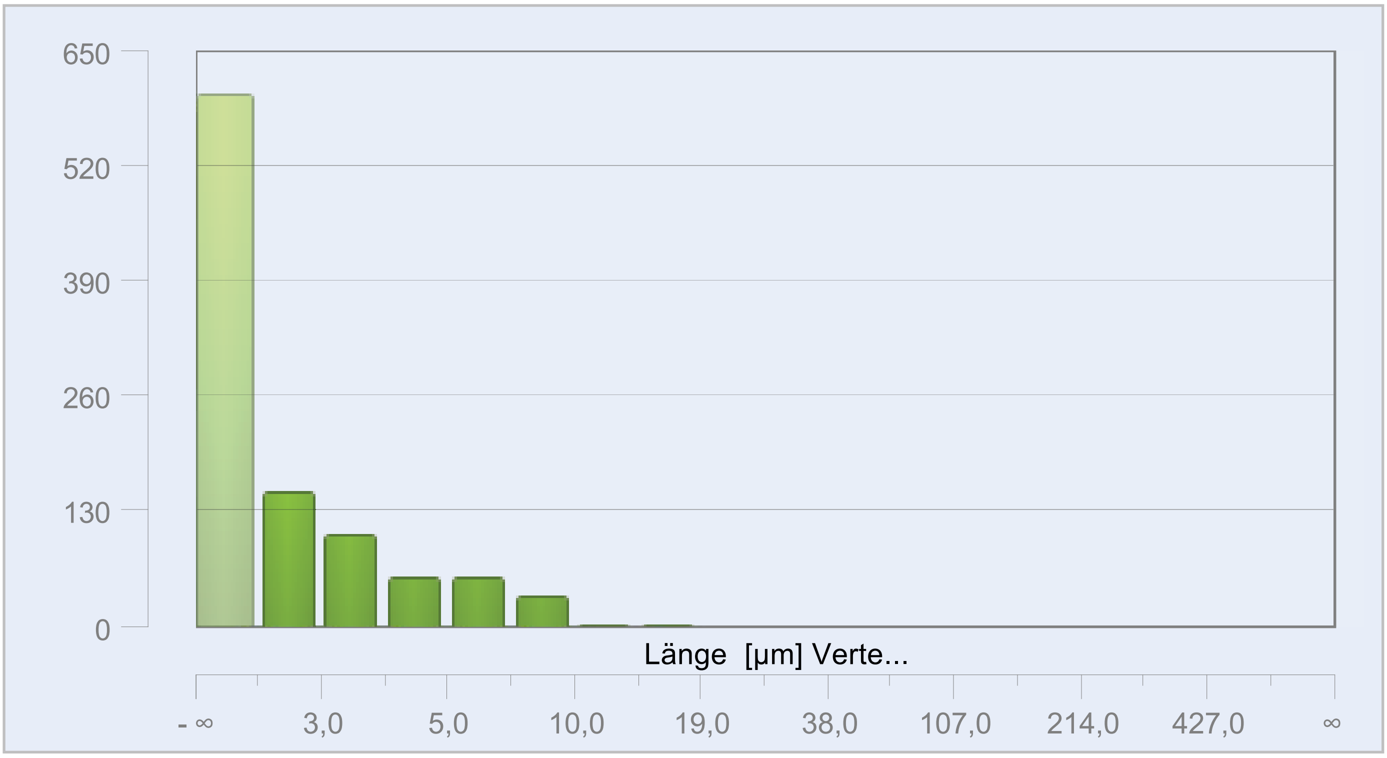

Start	Ende	Absolute Häufigkeit	Absolute Häufigkeit (kumuliert)	Relative Häufigkeit [%]	Relative Häufigkeit (kumuliert) [%]
	2,0 µm	602	602	59	59
2,0 µm	3,0 µm	153	755	15	74
3,0 µm	4,0 µm	106	861	10	84
4,0 µm	5,0 µm	59	920	6	90
5,0 µm	7,0 µm	59	979	6	95
7,0 µm	10,0 µm	37	1016	4	99
10,0 µm	13,0 µm	5	1021	0	100
13,0 µm	19,0 µm	4	1025	0	100
19,0 µm	27,0 µm	1	1026	0	100
27,0 µm	38,0 µm	0	1026	0	100
38,0 µm	75,0 µm	0	1026	0	100
75,0 µm	107,0 µm	0	1026	0	100
107,0 µm	151,0 µm	0	1026	0	100
151,0 µm	214,0 µm	0	1026	0	100
214,0 µm	302,0 µm	0	1026	0	100
302,0 µm	427,0 µm	0	1026	0	100
427,0 µm	600,0 µm	0	1026	0	100
600,0 µm		0	1026	0	100

4. Single Result 3 (CrCoNi - ASTM E 112_CrCoNi_homogenized_8.1mmSW_800°C_180min_00176)

Mittlere Sehnenlänge	2,4 µm
Korngröße (ASTM)	14,1
Korngröße (G643)	14
Kornstreckung	85,8 %

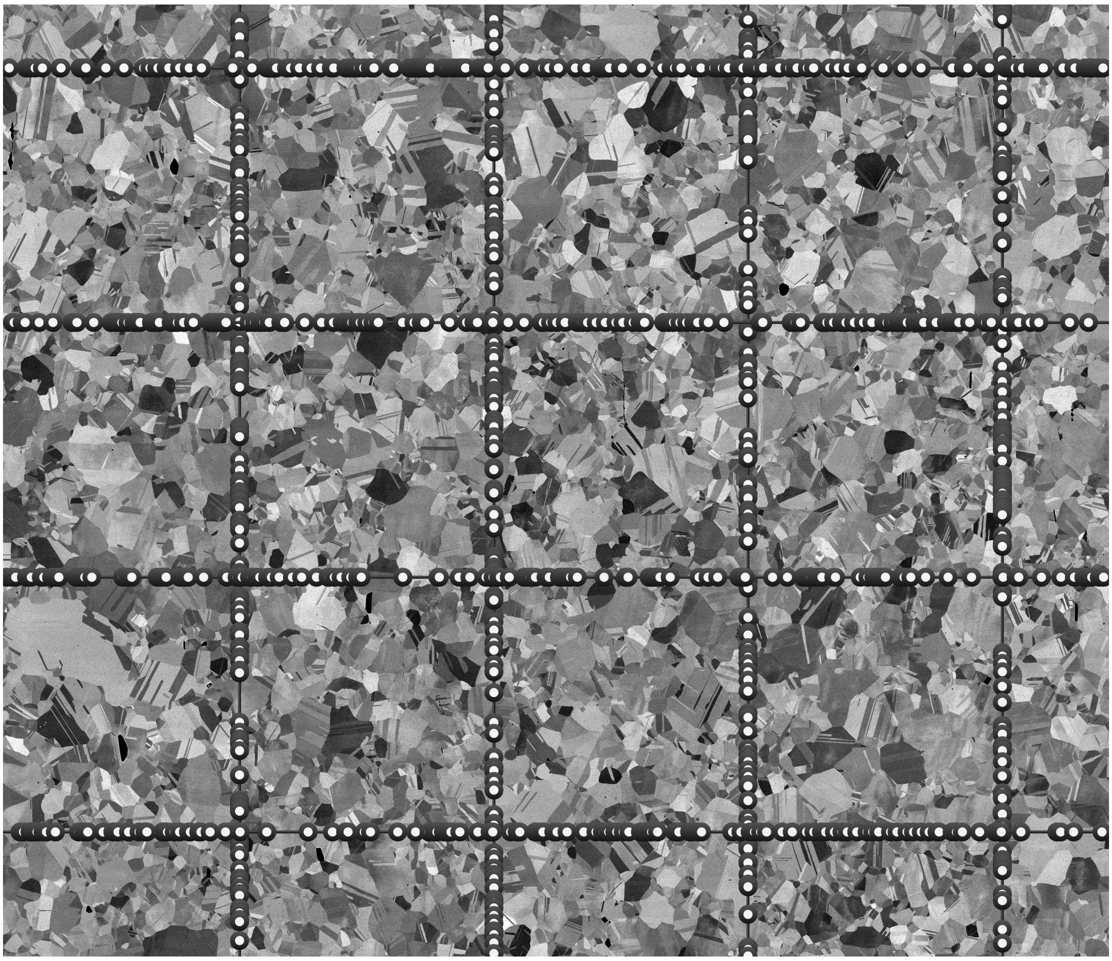

4.1. Statistische Analyse

Statistische Daten	Länge
Anzahl Objekte	970
Minimum	0,1 µm
Maximum	16,0 µm
Mittelwert	2,4 µm
Standardabweichung	2,3 µm
Schiefe	0,0
Standardabweichung (n-1)	2,3 µm
Varianz	5,2 µm²
Varianz (n-1)	5,2 µm²
Summe	2'362,2 µm
Quadratsumme	10'750,0 µm²
Kubiksumme	71'524,6 µm³

4.1.1. Chord Lenght Distribution

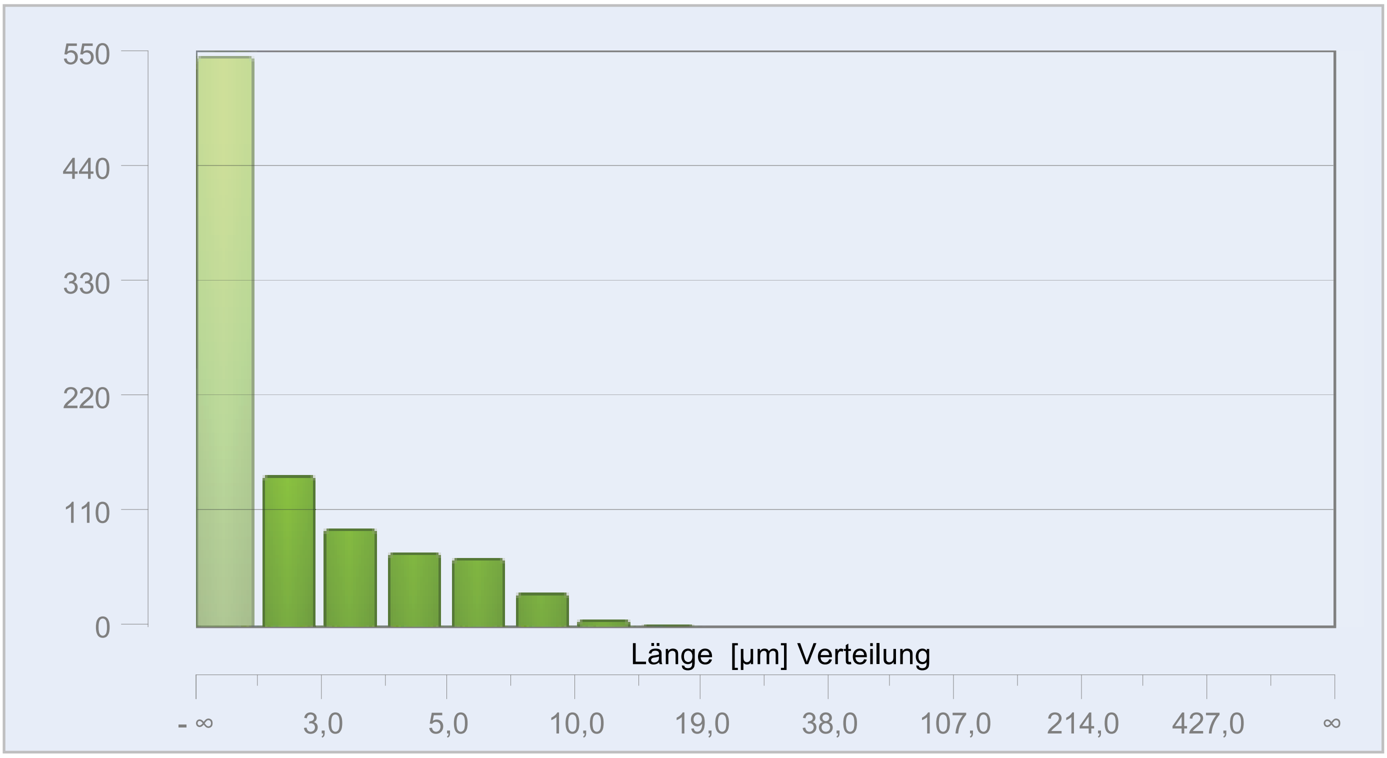

Start	Ende	Absolute Häufigkeit	Absolute Häufigkeit (kumuliert)	Relative Häufigkeit [%]	Relative Häufigkeit (kumuliert) [%]
	2,0 µm	544	544	56	56
2,0 µm	3,0 µm	145	689	15	71
3,0 µm	4,0 µm	94	783	10	81
4,0 µm	5,0 µm	73	856	8	88
5,0 µm	7,0 µm	66	922	7	95
7,0 µm	10,0 µm	35	957	4	99
10,0 µm	13,0 µm	10	967	1	100
13,0 µm	19,0 µm	3	970	0	100
19,0 µm	27,0 µm	0	970	0	100
27,0 µm	38,0 µm	0	970	0	100
38,0 µm	75,0 µm	0	970	0	100
75,0 µm	107,0 µm	0	970	0	100
107,0 µm	151,0 µm	0	970	0	100
151,0 µm	214,0 µm	0	970	0	100
214,0 µm	302,0 µm	0	970	0	100
302,0 µm	427,0 µm	0	970	0	100
427,0 µm	600,0 µm	0	970	0	100
600,0 µm		0	970	0	100

5. Single Result 4 (CrCoNi - ASTM E 112_CrCoNi_homogenized_8.1mmSW_800°C_180min_00177)

Mittlere Sehnenlänge	2,3 µm
Korngröße (ASTM)	14,2
Korngröße (G643)	14,1
Kornstreckung	88,8 %

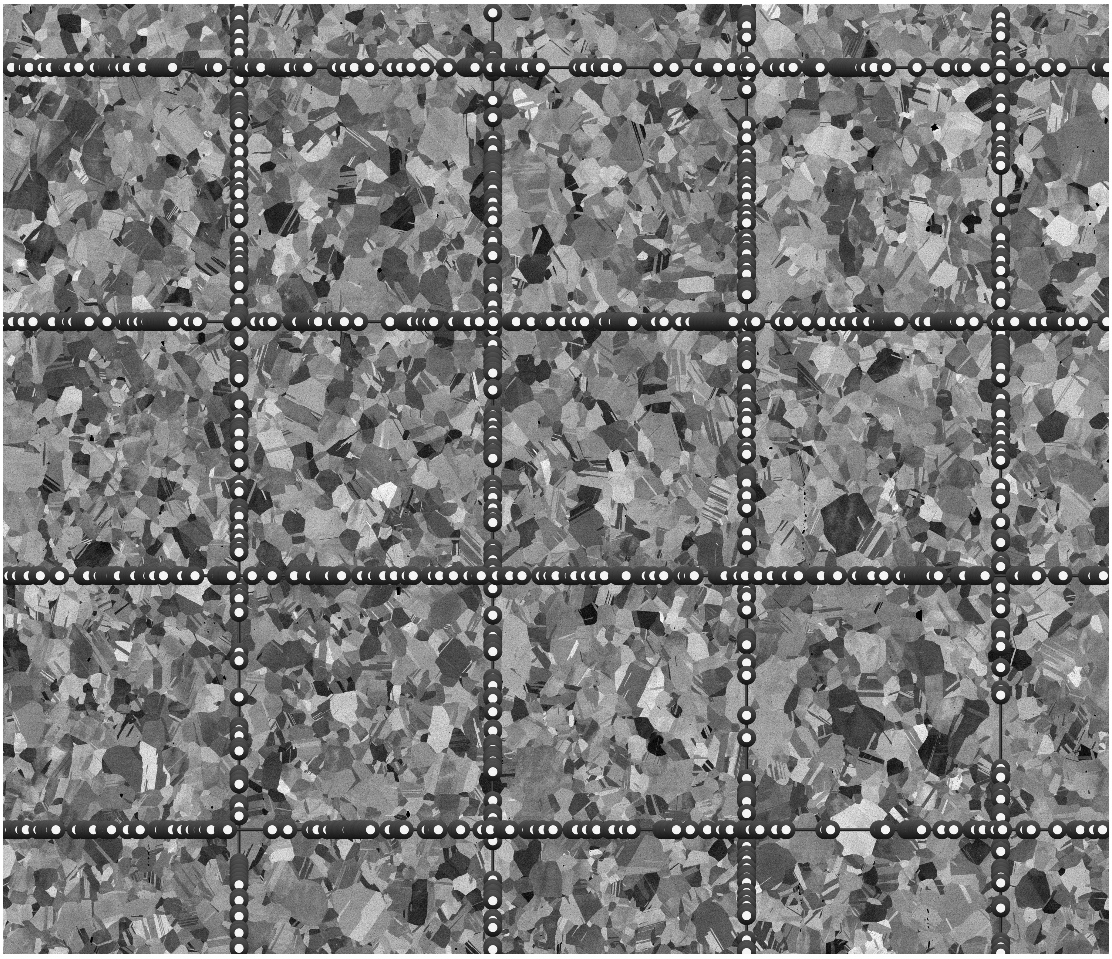

5.1. Statistische Analyse

Statistische Daten	Länge
Anzahl Objekte	1005
Minimum	0,2 µm
Maximum	21,1 µm
Mittelwert	2,3 µm
Standardabweichung	2,3 µm
Schiefe	0,0
Standardabweichung (n-1)	2,3 µm
Varianz	5,1 µm²
Varianz (n-1)	5,1 µm²
Summe	2'360,6 µm
Quadratsumme	10'700,5 µm²
Kubiksumme	75'318,0 µm³

5.1.1. Chord Lenght Distribution

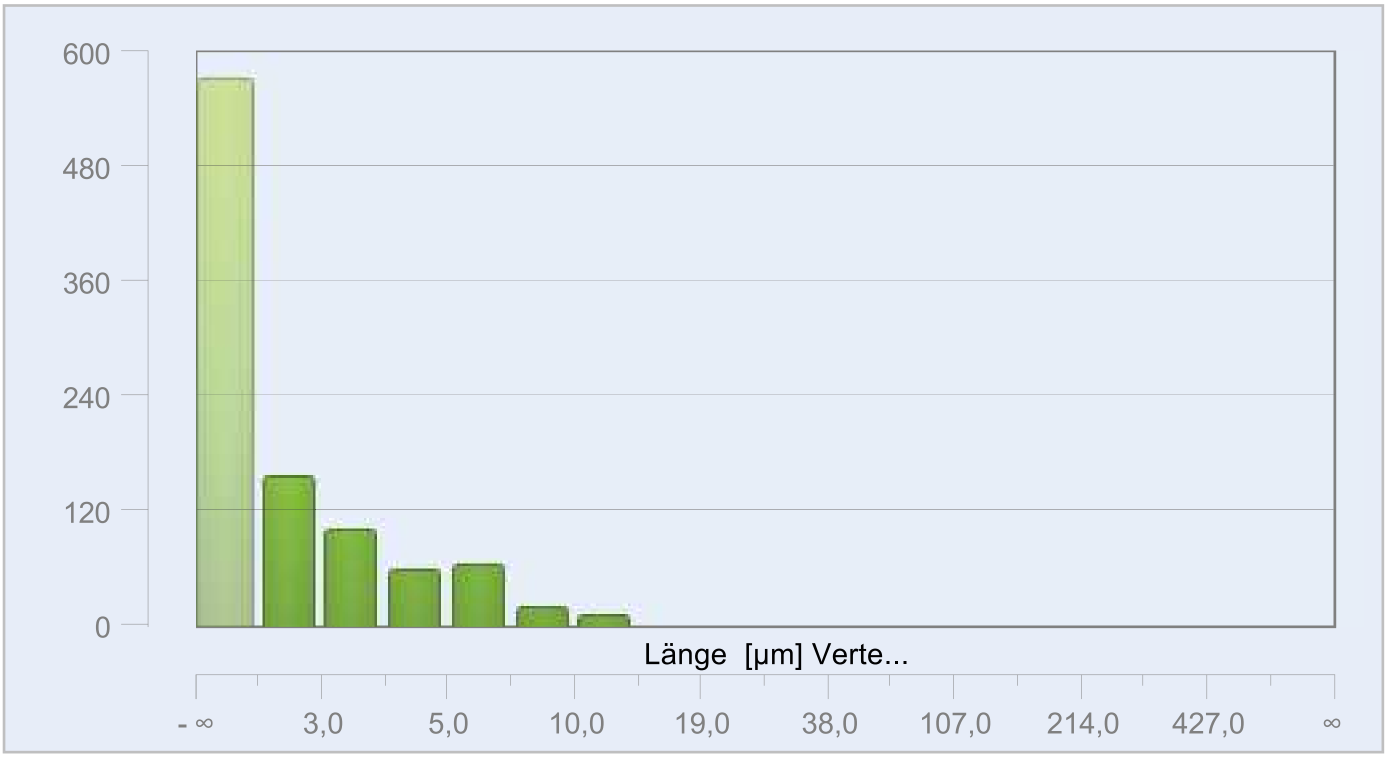

Start	Ende	Absolute Häufigkeit	Absolute Häufigkeit (kumuliert)	Relative Häufigkeit [%]	Relative Häufigkeit (kumuliert) [%]
	2,0 µm	570	570	57	57
2,0 µm	3,0 µm	159	729	16	73
3,0 µm	4,0 µm	105	834	10	83
4,0 µm	5,0 µm	62	896	6	89
5,0 µm	7,0 µm	67	963	7	96
7,0 µm	10,0 µm	24	987	2	98
10,0 µm	13,0 µm	15	1002	1	100
13,0 µm	19,0 µm	2	1004	0	100
19,0 µm	27,0 µm	1	1005	0	100
27,0 µm	38,0 µm	0	1005	0	100
38,0 µm	75,0 µm	0	1005	0	100
75,0 µm	107,0 µm	0	1005	0	100
107,0 µm	151,0 µm	0	1005	0	100
151,0 µm	214,0 µm	0	1005	0	100
214,0 µm	302,0 µm	0	1005	0	100
302,0 µm	427,0 µm	0	1005	0	100
427,0 µm	600,0 µm	0	1005	0	100
600,0 µm		0	1005	0	100
