# Supplementary material for: Benchmark dataset of the effect of grain size on strength in the single-phase FCC CrCoNi medium entropy alloy
Source: Data Brief. 2019 Oct 1;27:104592. doi: 10.1016/j.dib.2019.104592 (PMC6812030; doi:10.1016/j.dib.2019.104592)
Supplement: Multimedia component 1 [file mmc1.zip › CrCoNi_1073K_180min/CrCoNi_1073K_180min_d=5.3μm.pdf]

Analysebericht

Aug 30, 2017 3:41:54 PM

powered by imagic.ch

1. cumulative Result 1

Number of images	4
Grain size (ASTM)	11.8
Grain size (G643)	11.8
Grain stretching	93.9 %
Mean chord length	5.3 µm

2. Single Result 1 (CrCoNi Twins grain size_ASTM 800C 180min_00179)

Mean chord length	5.2 µm
Grain size (ASTM)	11.9
Grain size (G643)	11.8
Grain stretching	87.4 %

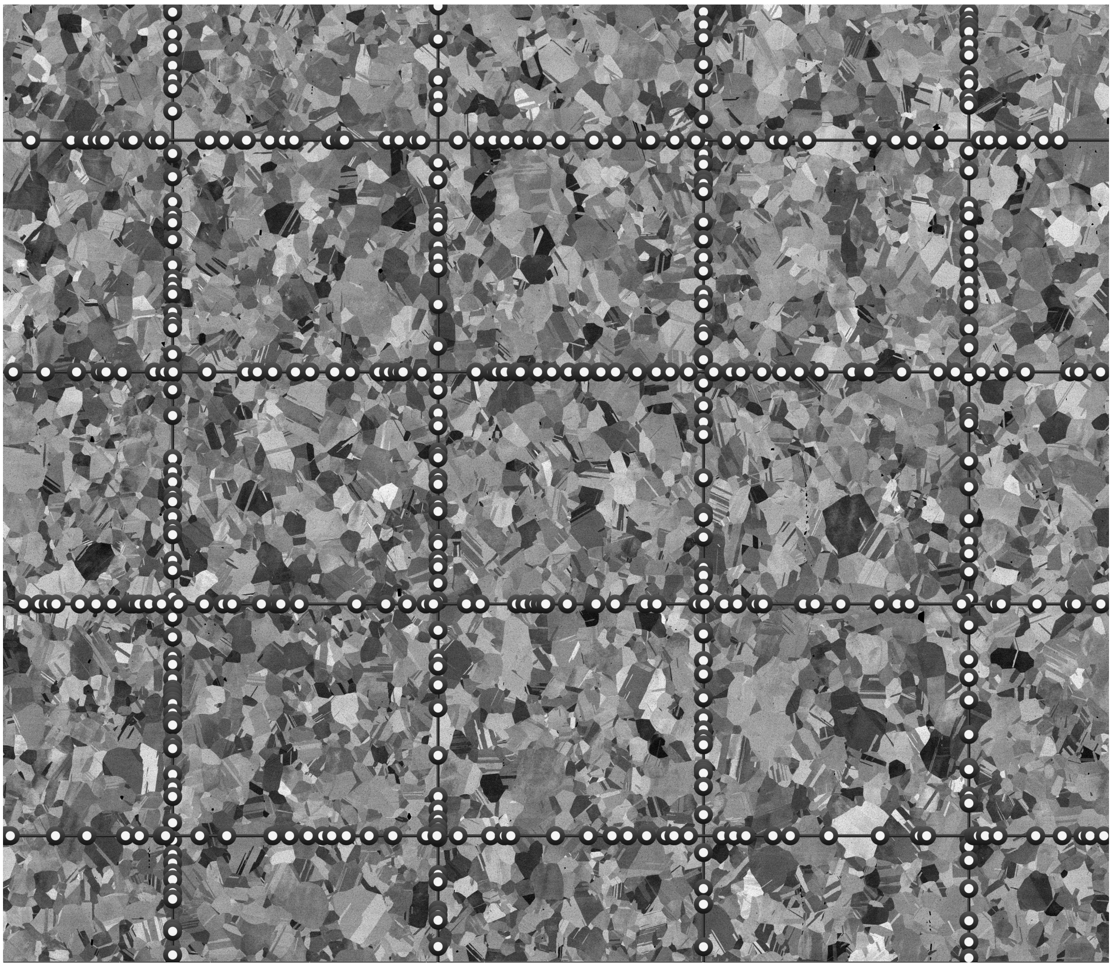

2.1. Statistical Analysis

Statistical Data	Length
Object Count	450
Minimum	0.5 µm
Maximum	18.6 µm
Average	5.2 µm
Standard deviation	3.3 µm
Skewness	0.0
Standard deviation (n-1)	3.3 µm
Variance	11.1 µm²
Variance (n-1)	11.1 µm²
Sum	2'359.2 µm

Statistical Data	Length
Sum of squares	17'356.4 µm²
Sum of cubes	158'422.8 µm³

2.1.1. Chord Lenght Distribution

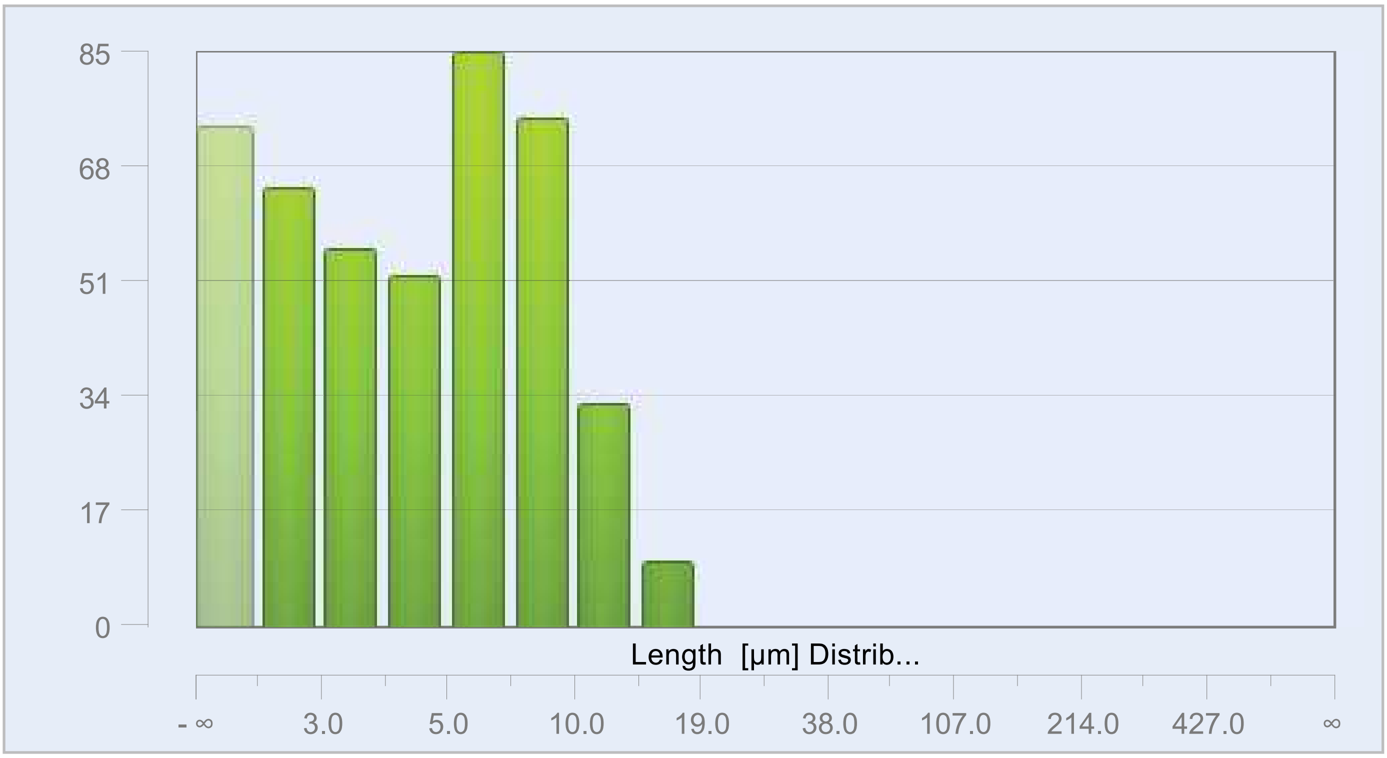

Start	End	Absolute Frequency	Absolute Frequency (accumulated)	Relative Frequency [%]	Relative Frequency (accumulated) [%]
	2.0 µm	74	74	16	16
2.0 µm	3.0 µm	65	139	14	31
3.0 µm	4.0 µm	56	195	12	43
4.0 µm	5.0 µm	52	247	12	55
5.0 µm	7.0 µm	85	332	19	74
7.0 µm	10.0 µm	75	407	17	90
10.0 µm	13.0 µm	33	440	7	98
13.0 µm	19.0 µm	10	450	2	100
19.0 µm	27.0 µm	0	450	0	100
27.0 µm	38.0 µm	0	450	0	100
38.0 µm	75.0 µm	0	450	0	100
75.0 µm	107.0 µm	0	450	0	100
107.0 µm	151.0 µm	0	450	0	100
151.0 µm	214.0 µm	0	450	0	100
214.0 µm	302.0 µm	0	450	0	100
302.0 µm	427.0 µm	0	450	0	100
427.0 µm	600.0 µm	0	450	0	100
600.0 µm		0	450	0	100

3. Single Result 2 (CrCoNi Twins grain size_ASTM 800C 180min_00180)

Mean chord length	5 µm
Grain size (ASTM)	12
Grain size (G643)	11.9
Grain stretching	92.8 %

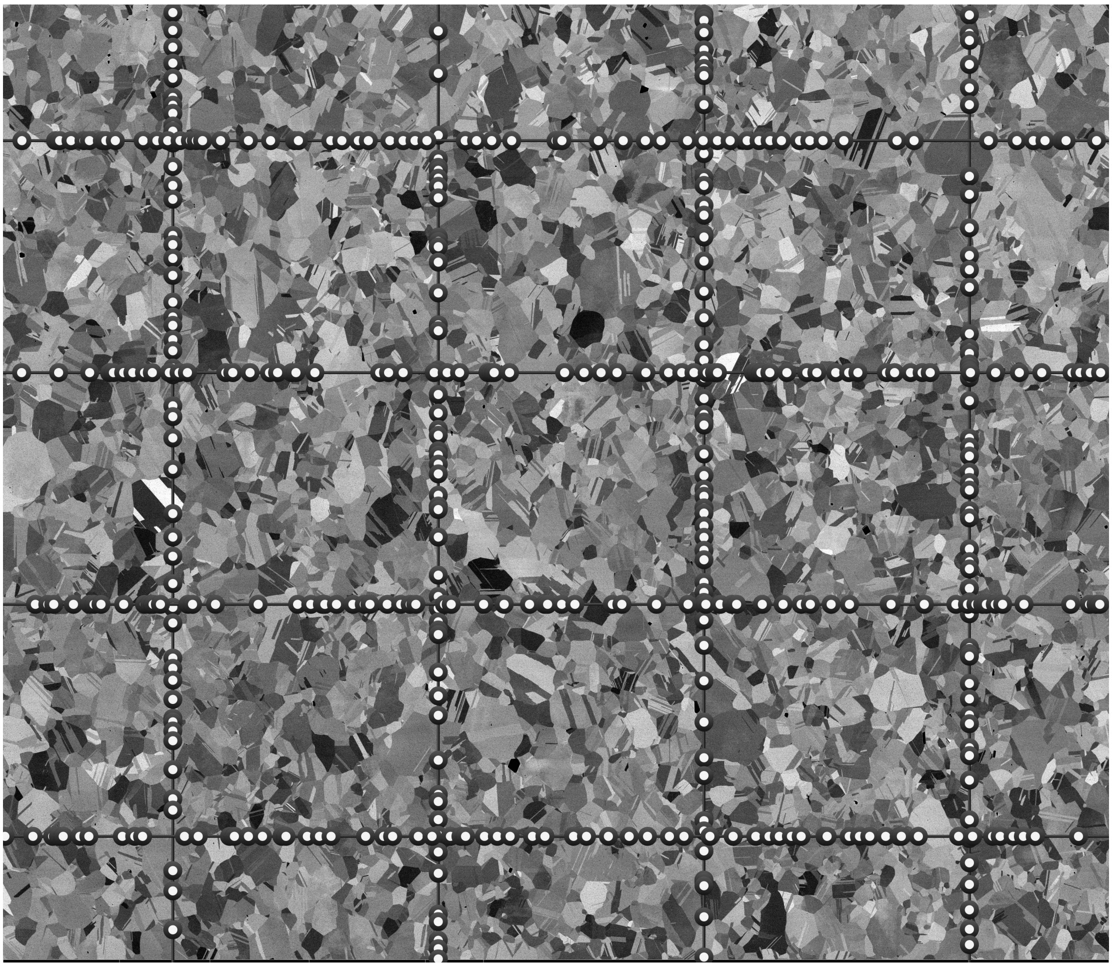

3.1. Statistical Analysis

Statistical Data	Length
Object Count	469
Minimum	0.5 µm
Maximum	21.4 µm
Average	5.0 µm
Standard deviation	3.4 µm
Skewness	0.0
Standard deviation (n-1)	3.4 µm
Variance	11.2 µm²
Variance (n-1)	11.3 µm²
Sum	2'368.1 µm
Sum of squares	17'225.5 µm²
Sum of cubes	164'528.3 µm³

3.1.1. Chord Lenght Distribution

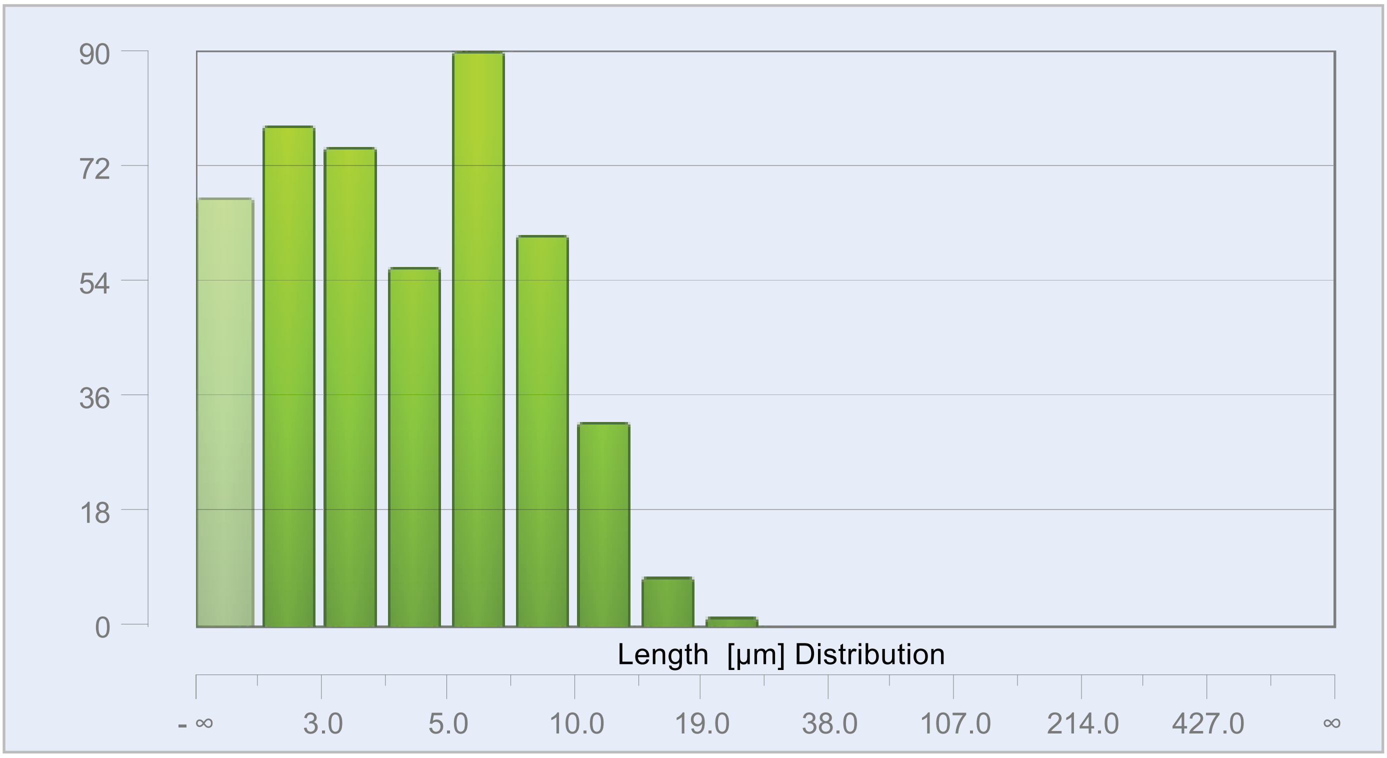

Start	End	Absolute Frequency	Absolute Frequency (accumulated)	Relative Frequency [%]	Relative Frequency (accumulated) [%]
	2.0 µm	67	67	14	14
2.0 µm	3.0 µm	78	145	17	31
3.0 µm	4.0 µm	75	220	16	47
4.0 µm	5.0 µm	56	276	12	59
5.0 µm	7.0 µm	90	366	19	78
7.0 µm	10.0 µm	61	427	13	91
10.0 µm	13.0 µm	32	459	7	98
13.0 µm	19.0 µm	8	467	2	100
19.0 µm	27.0 µm	2	469	0	100
27.0 µm	38.0 µm	0	469	0	100
38.0 µm	75.0 µm	0	469	0	100
75.0 µm	107.0 µm	0	469	0	100
107.0 µm	151.0 µm	0	469	0	100
151.0 µm	214.0 µm	0	469	0	100
214.0 µm	302.0 µm	0	469	0	100
302.0 µm	427.0 µm	0	469	0	100
427.0 µm	600.0 µm	0	469	0	100
600.0 µm		0	469	0	100

4. Single Result 3 (CrCoNi Twins grain size_ASTM 800C 180min_00181)

Mean chord length	5.1 µm
Grain size (ASTM)	11.9
Grain size (G643)	11.9
Grain stretching	97.2 %

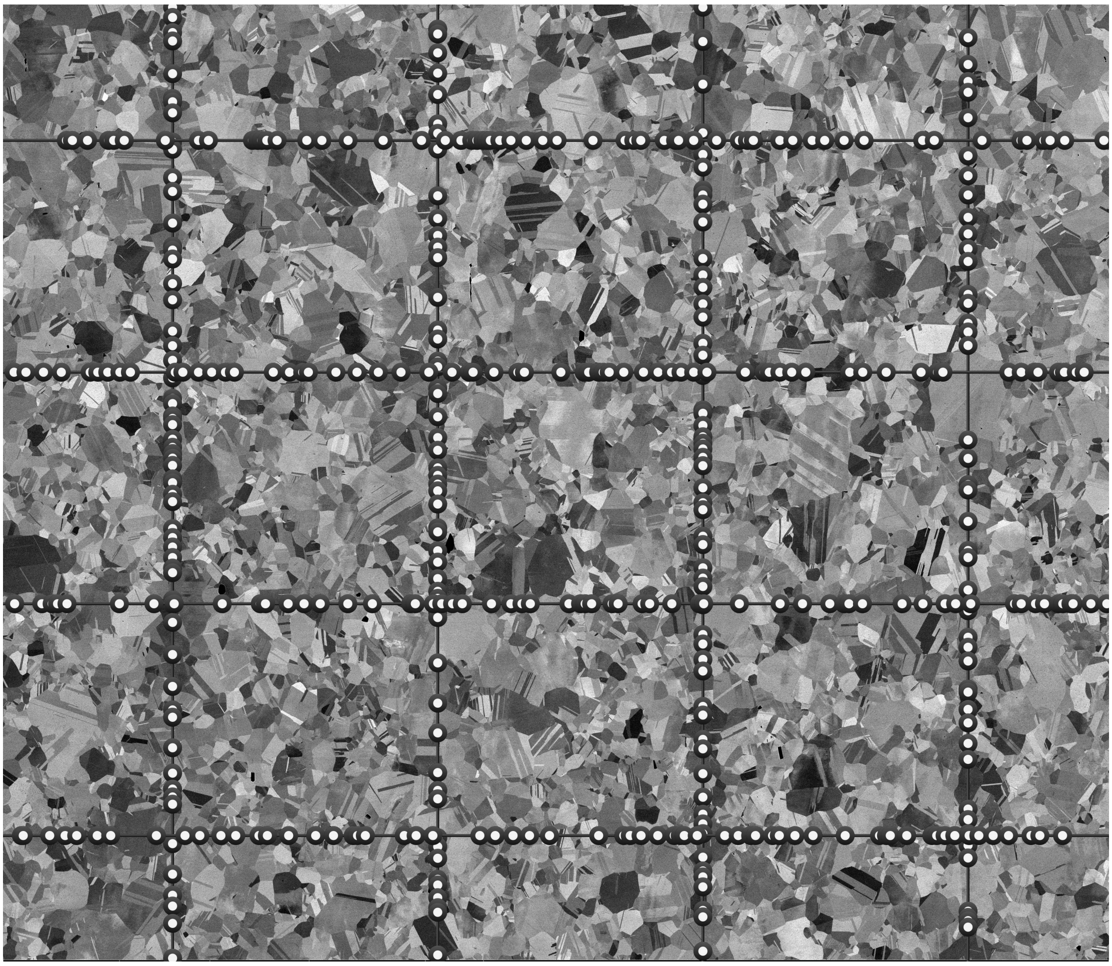

4.1. Statistical Analysis

Statistical Data	Length
Object Count	463
Minimum	0.5 µm
Maximum	27.1 µm
Average	5.1 µm
Standard deviation	3.5 µm
Skewness	0.0
Standard deviation (n-1)	3.5 µm
Variance	12.3 µm²
Variance (n-1)	12.3 µm²
Sum	2'366.5 µm
Sum of squares	17'778.9 µm²
Sum of cubes	178'578.3 µm³

4.1.1. Chord Lenght Distribution

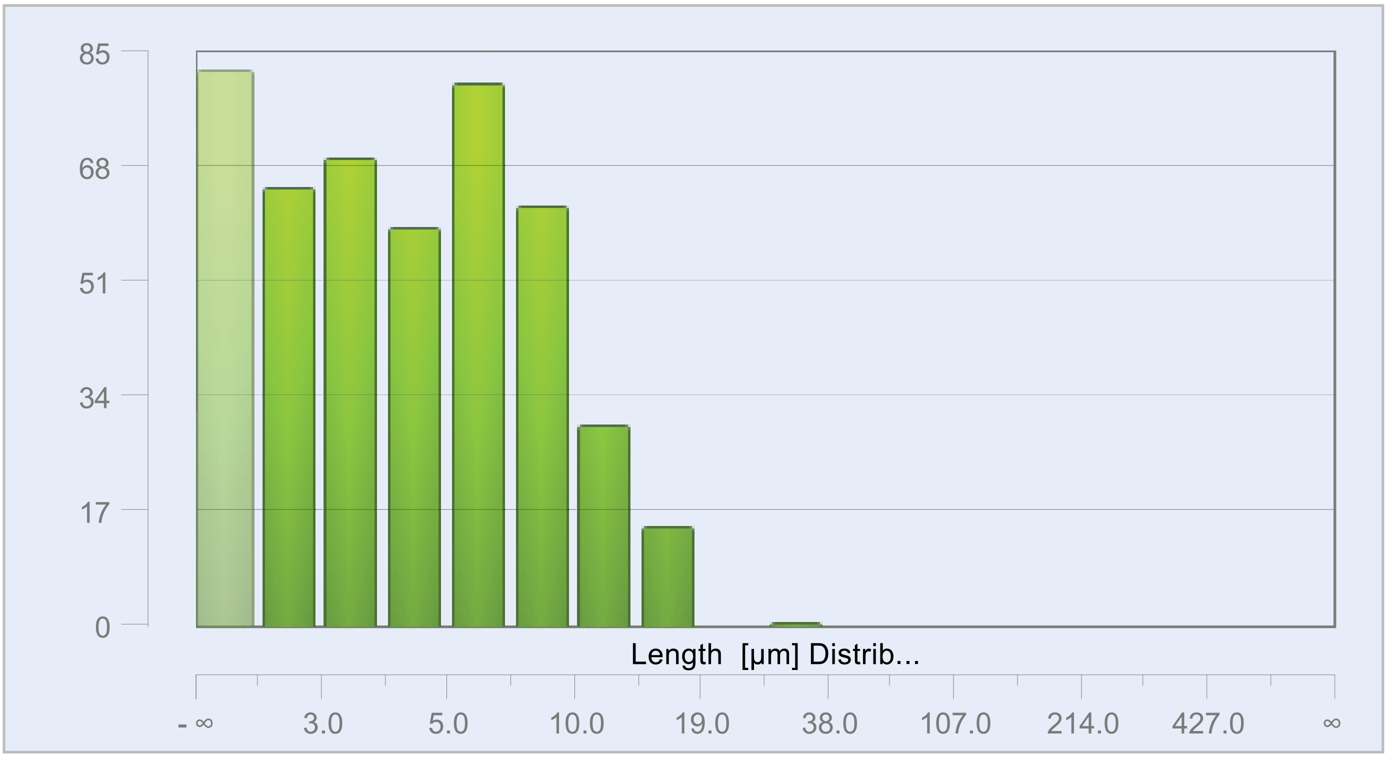

Start	End	Absolute Frequency	Absolute Frequency (accumulated)	Relative Frequency [%]	Relative Frequency (accumulated) [%]
	2.0 µm	82	82	18	18
2.0 µm	3.0 µm	65	147	14	32
3.0 µm	4.0 µm	69	216	15	47
4.0 µm	5.0 µm	59	275	13	59
5.0 µm	7.0 µm	80	355	17	77
7.0 µm	10.0 µm	62	417	13	90
10.0 µm	13.0 µm	30	447	6	97
13.0 µm	19.0 µm	15	462	3	100
19.0 µm	27.0 µm	0	462	0	100
27.0 µm	38.0 µm	1	463	0	100
38.0 µm	75.0 µm	0	463	0	100
75.0 µm	107.0 µm	0	463	0	100
107.0 µm	151.0 µm	0	463	0	100
151.0 µm	214.0 µm	0	463	0	100
214.0 µm	302.0 µm	0	463	0	100
302.0 µm	427.0 µm	0	463	0	100
427.0 µm	600.0 µm	0	463	0	100
600.0 µm		0	463	0	100

5. Single Result 4 (CrCoNi Twins grain size_ASTM 800C 180min_00182)

Mean chord length	5.8 µm
Grain size (ASTM)	11.6
Grain size (G643)	11.5
Grain stretching	83.5 %

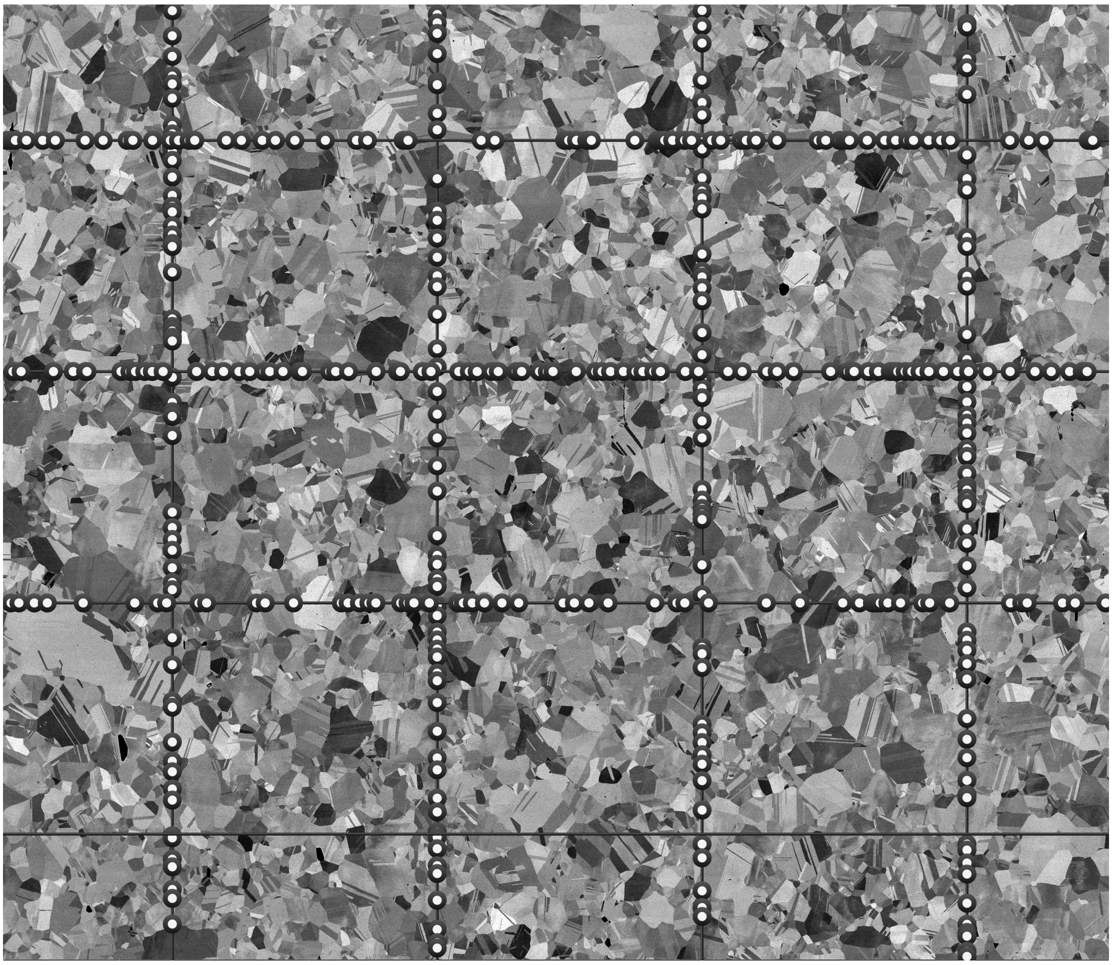

5.1. Statistical Analysis

Statistical Data	Length
Object Count	405
Minimum	0.4 µm
Maximum	317.2 µm
Average	5.8 µm
Standard deviation	15.9 µm
Skewness	0.0
Standard deviation (n-1)	15.9 µm
Variance	253.6 µm²
Variance (n-1)	254.2 µm²
Sum	2'365.3 µm
Sum of squares	116'528.3 µm²
Sum of cubes	32'083'202.4 µm³

5.1.1. Chord Lenght Distribution

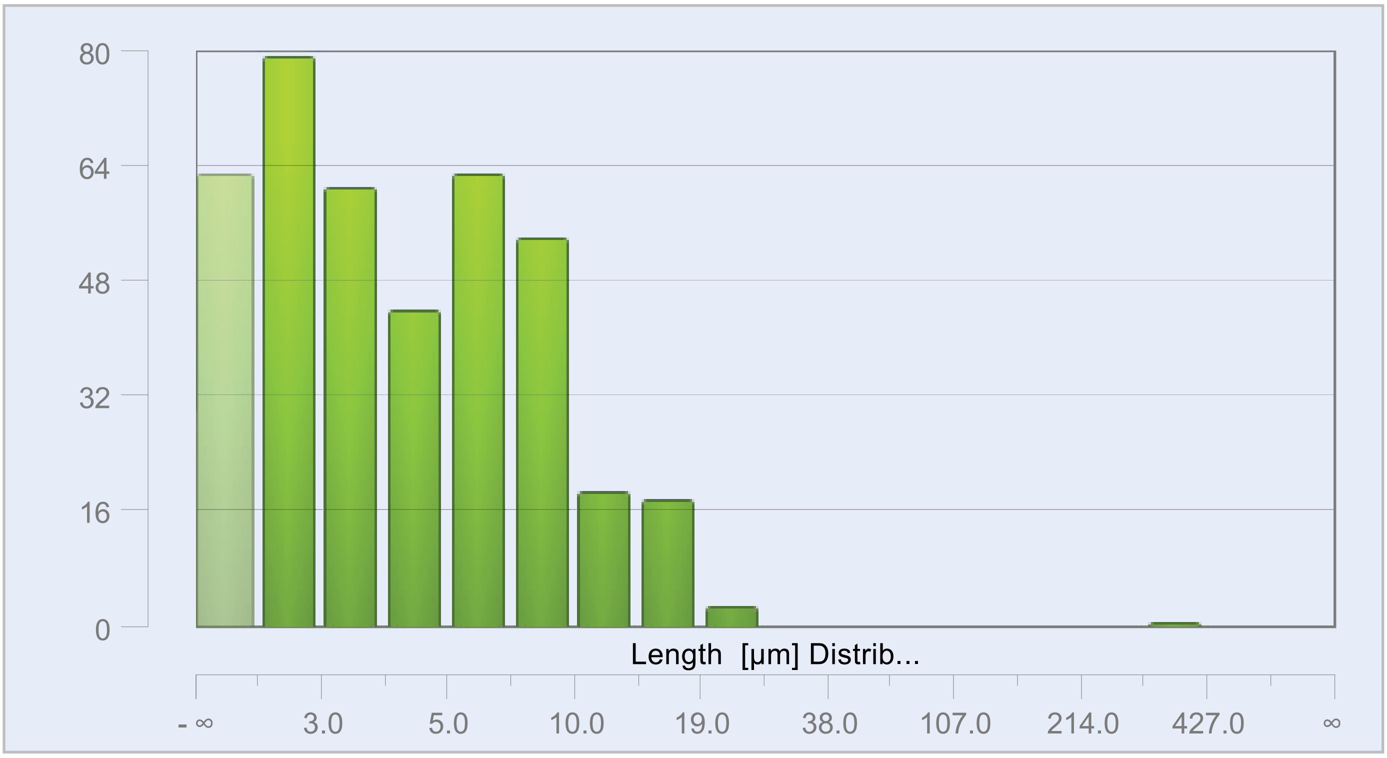

Start	End	Absolute Frequency	Absolute Frequency (accumulated)	Relative Frequency [%]	Relative Frequency (accumulated) [%]
	2.0 µm	63	63	16	16
2.0 µm	3.0 µm	79	142	20	35
3.0 µm	4.0 µm	61	203	15	50
4.0 µm	5.0 µm	44	247	11	61
5.0 µm	7.0 µm	63	310	16	77
7.0 µm	10.0 µm	54	364	13	90
10.0 µm	13.0 µm	19	383	5	95
13.0 µm	19.0 µm	18	401	4	99
19.0 µm	27.0 µm	3	404	1	100
27.0 µm	38.0 µm	0	404	0	100
38.0 µm	75.0 µm	0	404	0	100
75.0 µm	107.0 µm	0	404	0	100
107.0 µm	151.0 µm	0	404	0	100
151.0 µm	214.0 µm	0	404	0	100
214.0 µm	302.0 µm	0	404	0	100
302.0 µm	427.0 µm	1	405	0	100
427.0 µm	600.0 µm	0	405	0	100
600.0 µm		0	405	0	100
