# Supplementary material for: Benchmark dataset of the effect of grain size on strength in the single-phase FCC CrCoNi medium entropy alloy
Source: Data Brief. 2019 Oct 1;27:104592. doi: 10.1016/j.dib.2019.104592 (PMC6812030; doi:10.1016/j.dib.2019.104592)
Supplement: Multimedia component 1 [file mmc1.zip › CrCoNi_1173K_10min/CrCoNi_1173K_10min_c=2.2μm.pdf]

Analysebericht

13.11.2017 09:53:00

powered by imagic.ch

1. cumulative Result 1

Anzahl Bilder	4
Korngröße (ASTM)	14,4
Korngröße (G643)	14,3
Kornstreckung	92,1 %
Mittlere Sehnenlänge	2,2 µm

2. Single Result 1 (CrCoNi - ASTM E 112_CrCoNi_homogenized_8.1mmSW_900°C_10min_00181)

Mittlere Sehnenlänge	2,1 µm
Korngröße (ASTM)	14,5
Korngröße (G643)	14,5
Kornstreckung	97,4 %

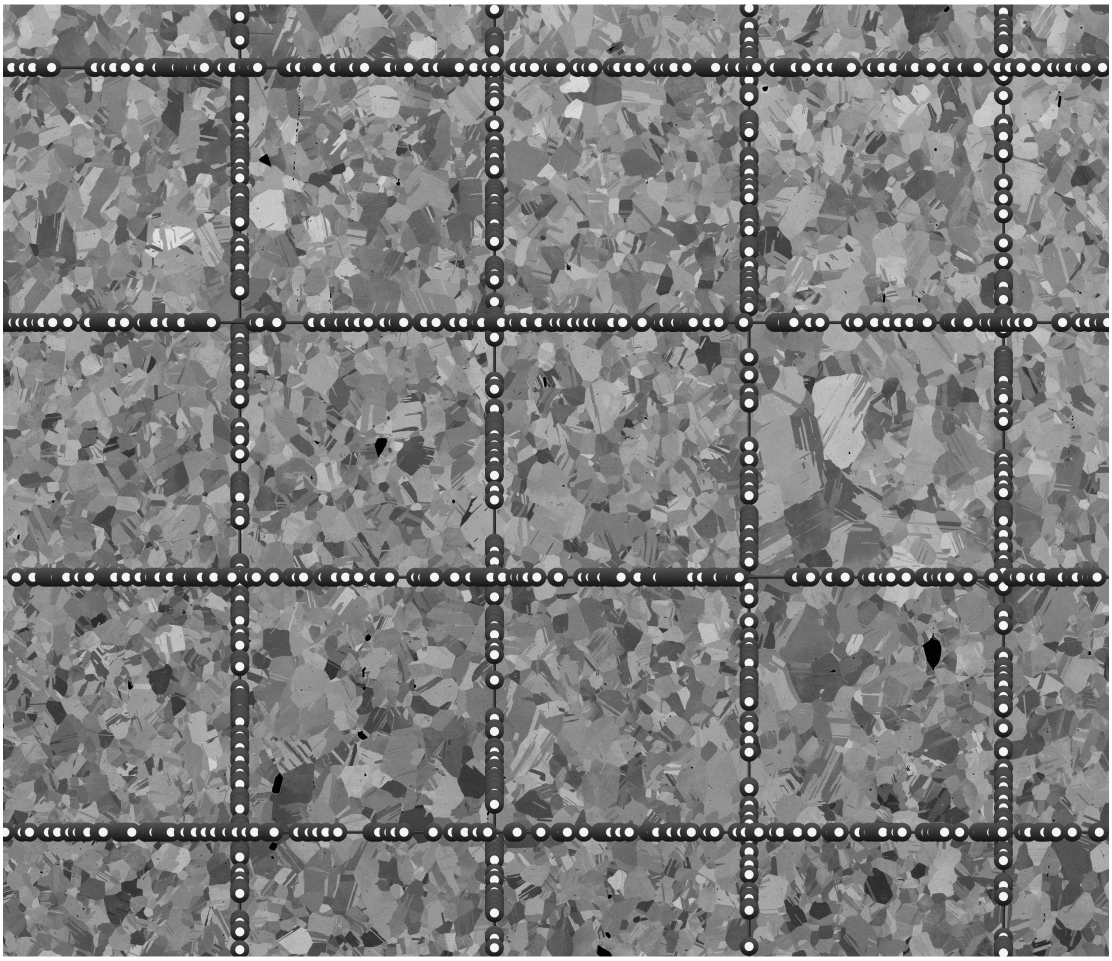

2.1. Statistische Analyse

Statistische Daten	Länge
Anzahl Objekte	1125
Minimum	0,2 µm
Maximum	15,6 µm
Mittelwert	2,1 µm
Standardabweichung	2,1 µm
Schiefe	0,0
Standardabweichung (n-1)	2,1 µm
Varianz	4,4 µm²
Varianz (n-1)	4,4 µm²
Summe	2'363,4 µm

Statistische Daten	Länge
Quadratsumme	9'955,5 µm²
Kubiksumme	65'123,9 µm³

2.1.1. Chord Lenght Distribution

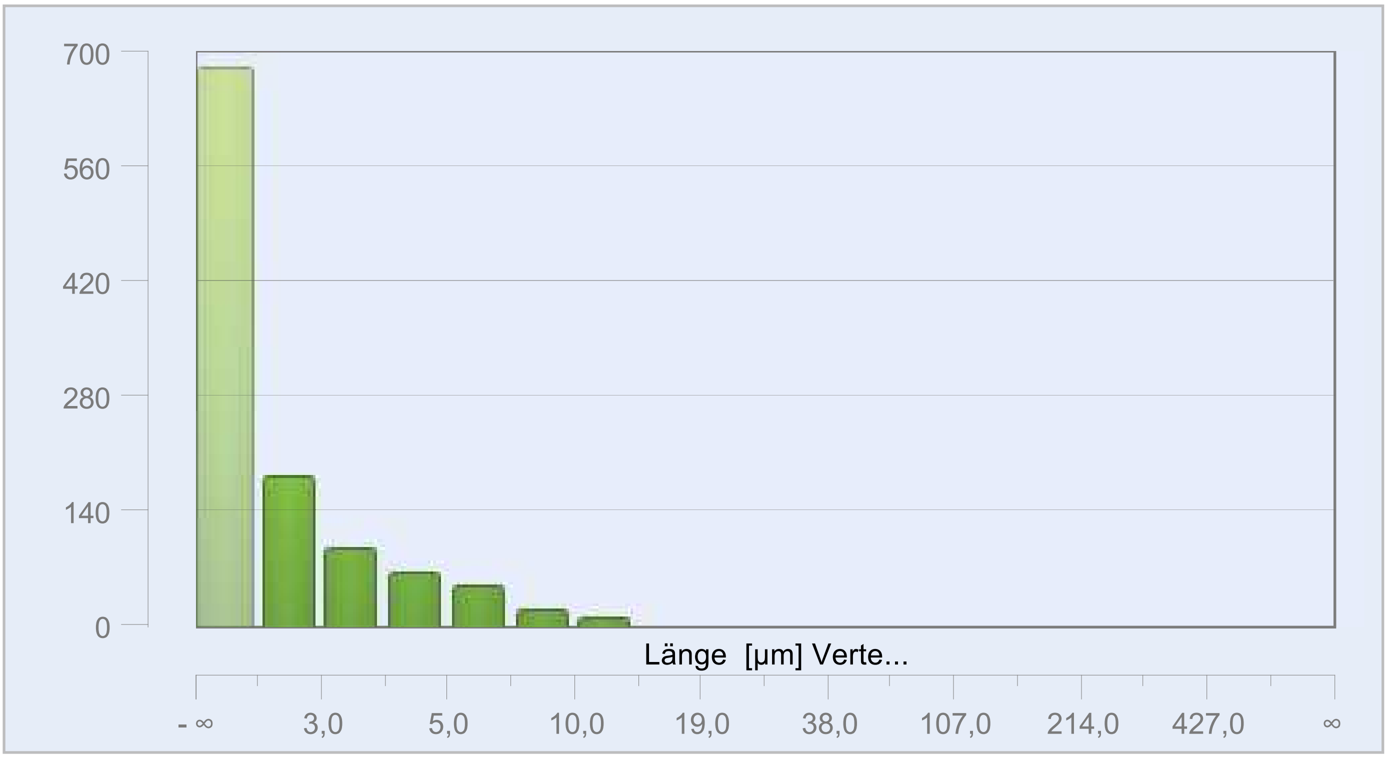

Start	Ende	Absolute Häufigkeit	Absolute Häufigkeit (kumuliert)	Relative Häufigkeit [%]	Relative Häufigkeit (kumuliert) [%]
	2,0 µm	680	680	60	60
2,0 µm	3,0 µm	186	866	17	77
3,0 µm	4,0 µm	97	963	9	86
4,0 µm	5,0 µm	69	1032	6	92
5,0 µm	7,0 µm	53	1085	5	96
7,0 µm	10,0 µm	25	1110	2	99
10,0 µm	13,0 µm	13	1123	1	100
13,0 µm	19,0 µm	2	1125	0	100
19,0 µm	27,0 µm	0	1125	0	100
27,0 µm	38,0 µm	0	1125	0	100
38,0 µm	75,0 µm	0	1125	0	100
75,0 µm	107,0 µm	0	1125	0	100
107,0 µm	151,0 µm	0	1125	0	100
151,0 µm	214,0 µm	0	1125	0	100
214,0 µm	302,0 µm	0	1125	0	100
302,0 µm	427,0 µm	0	1125	0	100
427,0 µm	600,0 µm	0	1125	0	100
600,0 µm		0	1125	0	100

3. Single Result 2 (CrCoNi - ASTM E 112_CrCoNi_homogenized_8.1mmSW_900°C_10min_00182)

Mittlere Sehnenlänge	2,1 µm
Korngröße (ASTM)	14,5
Korngröße (G643)	14,5
Kornstreckung	84 %

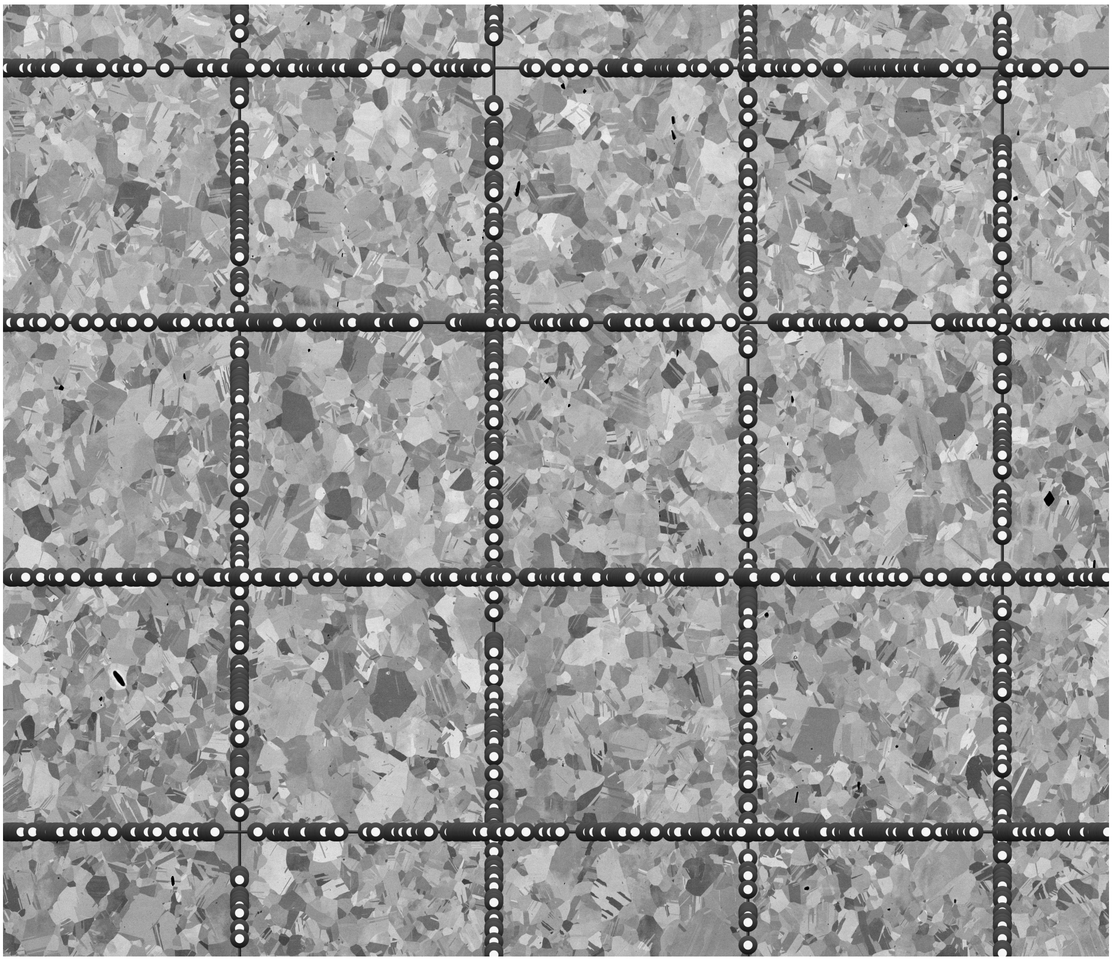

3.1. Statistische Analyse

Statistische Daten	Länge
Anzahl Objekte	1135
Minimum	0,2 µm
Maximum	19,4 µm
Mittelwert	2,1 µm
Standardabweichung	2,1 µm
Schiefe	0,0
Standardabweichung (n-1)	2,1 µm
Varianz	4,2 µm²
Varianz (n-1)	4,2 µm²
Summe	2'362,2 µm
Quadratsumme	9'690,5 µm²
Kubiksumme	64'883,5 µm³

3.1.1. Chord Lenght Distribution

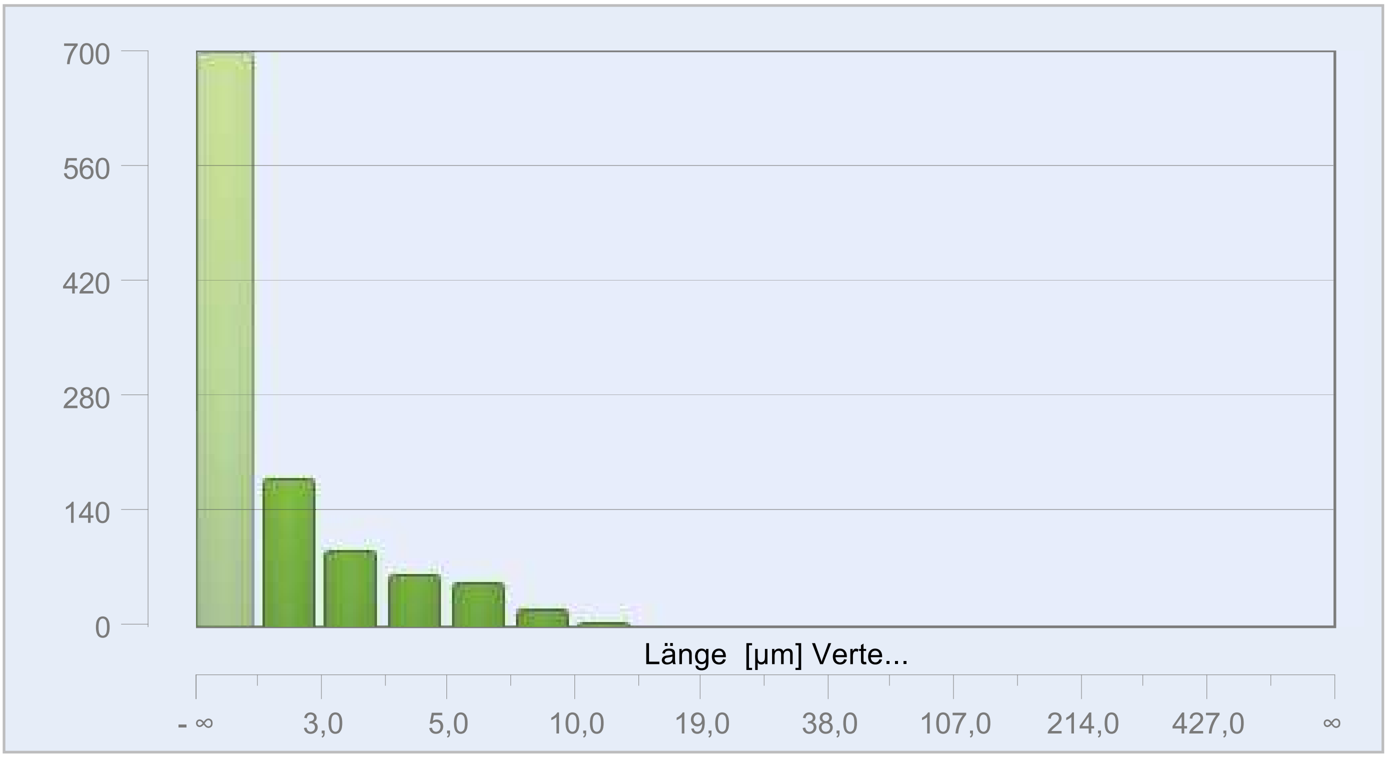

Start	Ende	Absolute Häufigkeit	Absolute Häufigkeit (kumuliert)	Relative Häufigkeit [%]	Relative Häufigkeit (kumuliert) [%]
	2,0 µm	698	698	61	61
2,0 µm	3,0 µm	181	879	16	77
3,0 µm	4,0 µm	96	975	8	86
4,0 µm	5,0 µm	67	1042	6	92
5,0 µm	7,0 µm	58	1100	5	97
7,0 µm	10,0 µm	24	1124	2	99
10,0 µm	13,0 µm	7	1131	1	100
13,0 µm	19,0 µm	3	1134	0	100
19,0 µm	27,0 µm	1	1135	0	100
27,0 µm	38,0 µm	0	1135	0	100
38,0 µm	75,0 µm	0	1135	0	100
75,0 µm	107,0 µm	0	1135	0	100
107,0 µm	151,0 µm	0	1135	0	100
151,0 µm	214,0 µm	0	1135	0	100
214,0 µm	302,0 µm	0	1135	0	100
302,0 µm	427,0 µm	0	1135	0	100
427,0 µm	600,0 µm	0	1135	0	100
600,0 µm		0	1135	0	100

4. Single Result 3 (CrCoNi - ASTM E 112_CrCoNi_homogenized_8.1mmSW_900°C_10min_00183)

Mittlere Sehnenlänge	2,2 µm
Korngröße (ASTM)	14,3
Korngröße (G643)	14,3
Kornstreckung	91,5 %

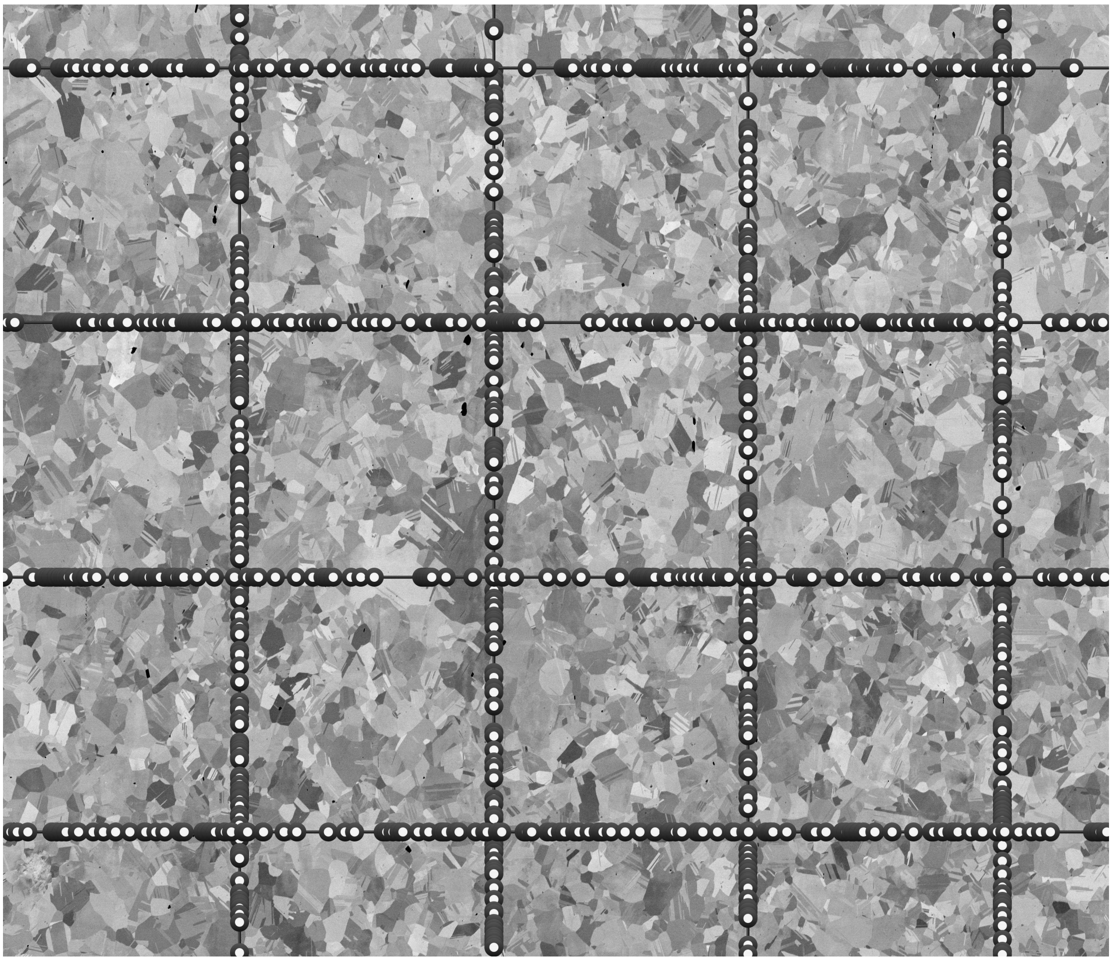

4.1. Statistische Analyse

Statistische Daten	Länge
Anzahl Objekte	1051
Minimum	0,1 µm
Maximum	15,7 µm
Mittelwert	2,2 µm
Standardabweichung	2,2 µm
Schiefe	0,0
Standardabweichung (n-1)	2,2 µm
Varianz	4,9 µm²
Varianz (n-1)	4,9 µm²
Summe	2'362,2 µm
Quadratsumme	10'478,4 µm²
Kubiksumme	71'192,9 µm³

4.1.1. Chord Lenght Distribution

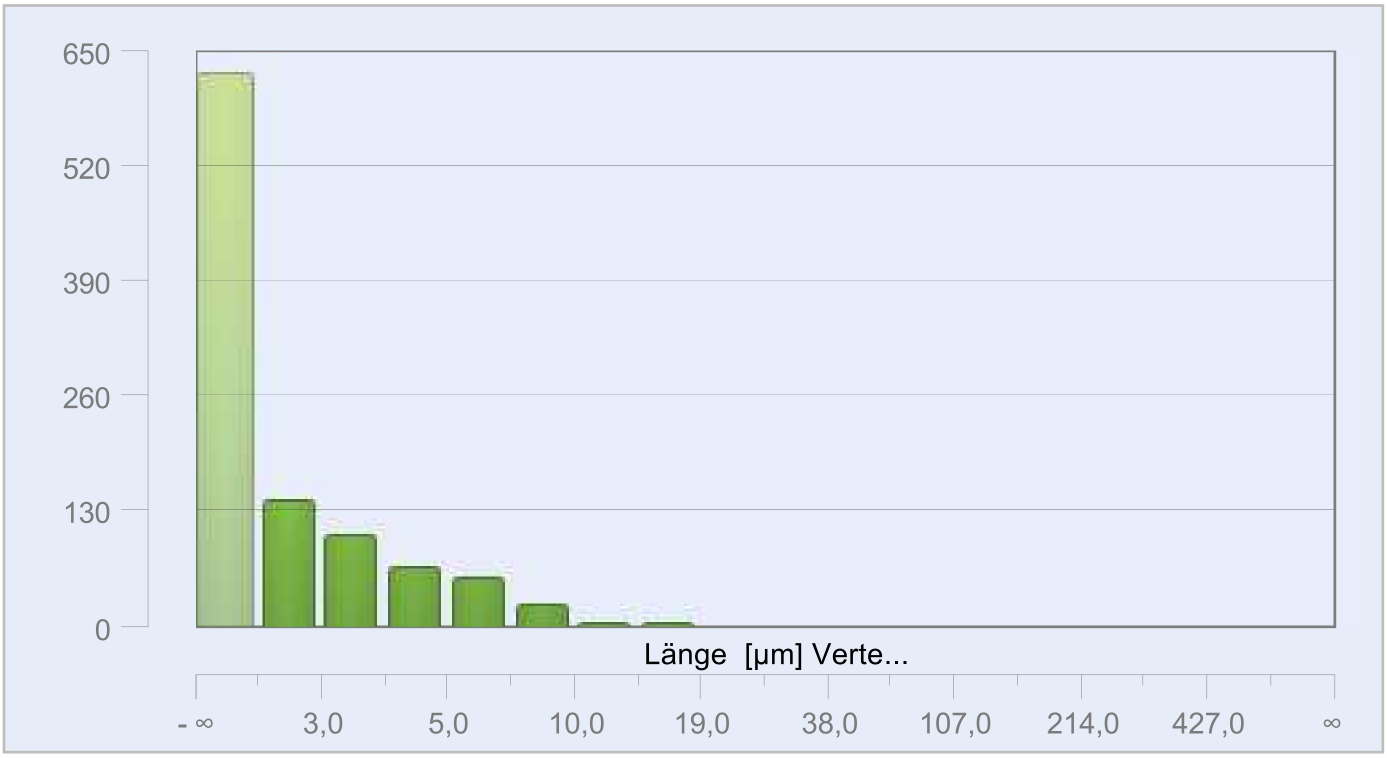

Start	Ende	Absolute Häufigkeit	Absolute Häufigkeit (kumuliert)	Relative Häufigkeit [%]	Relative Häufigkeit (kumuliert) [%]
	2,0 µm	626	626	60	60
2,0 µm	3,0 µm	145	771	14	73
3,0 µm	4,0 µm	107	878	10	84
4,0 µm	5,0 µm	71	949	7	90
5,0 µm	7,0 µm	59	1008	6	96
7,0 µm	10,0 µm	29	1037	3	99
10,0 µm	13,0 µm	8	1045	1	99
13,0 µm	19,0 µm	6	1051	1	100
19,0 µm	27,0 µm	0	1051	0	100
27,0 µm	38,0 µm	0	1051	0	100
38,0 µm	75,0 µm	0	1051	0	100
75,0 µm	107,0 µm	0	1051	0	100
107,0 µm	151,0 µm	0	1051	0	100
151,0 µm	214,0 µm	0	1051	0	100
214,0 µm	302,0 µm	0	1051	0	100
302,0 µm	427,0 µm	0	1051	0	100
427,0 µm	600,0 µm	0	1051	0	100
600,0 µm		0	1051	0	100

5. Single Result 4 (CrCoNi - ASTM E 112_CrCoNi_homogenized_8.1mmSW_900°C_10min_00184)

Mittlere Sehnenlänge	2,4 µm
Korngröße (ASTM)	14,2
Korngröße (G643)	14,1
Kornstreckung	91,1 %

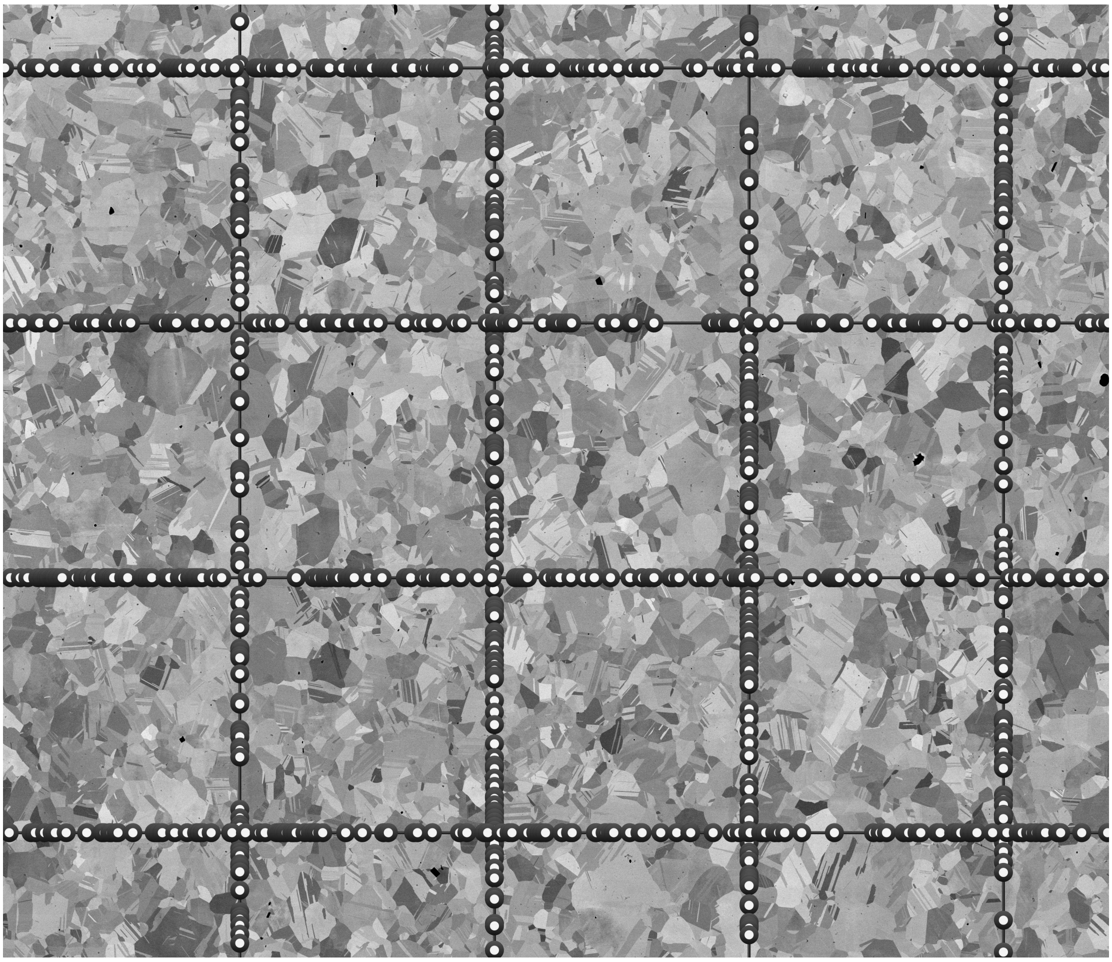

5.1. Statistische Analyse

Statistische Daten	Länge
Anzahl Objekte	1001
Minimum	0,2 µm
Maximum	16,5 µm
Mittelwert	2,4 µm
Standardabweichung	2,4 µm
Schiefe	0,0
Standardabweichung (n-1)	2,4 µm
Varianz	5,8 µm²
Varianz (n-1)	5,8 µm²
Summe	2'363,4 µm
Quadratsumme	11'420,6 µm²
Kubiksumme	81'391,0 µm³

5.1.1. Chord Lenght Distribution

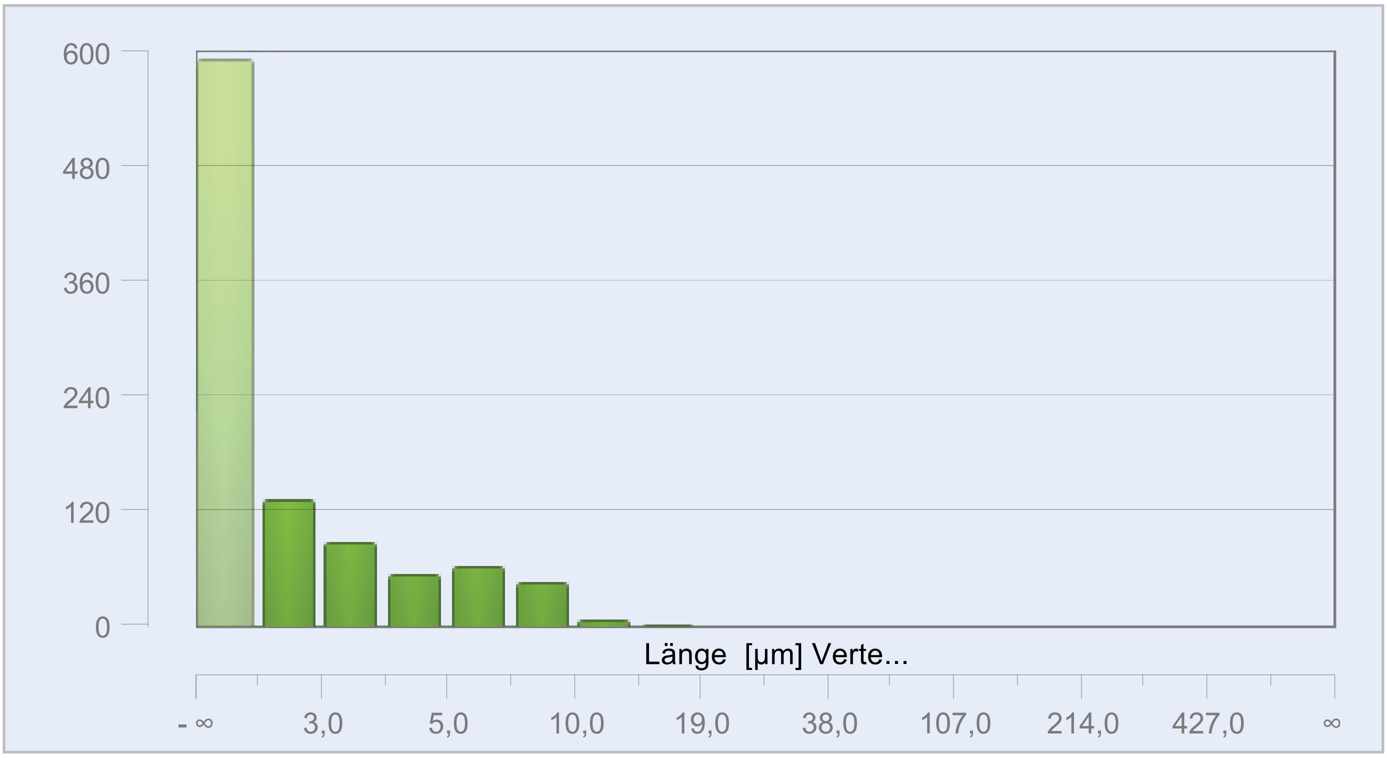

Start	Ende	Absolute Häufigkeit	Absolute Häufigkeit (kumuliert)	Relative Häufigkeit [%]	Relative Häufigkeit (kumuliert) [%]
	2,0 µm	591	591	59	59
2,0 µm	3,0 µm	134	725	13	72
3,0 µm	4,0 µm	90	815	9	81
4,0 µm	5,0 µm	57	872	6	87
5,0 µm	7,0 µm	66	938	7	94
7,0 µm	10,0 µm	48	986	5	99
10,0 µm	13,0 µm	10	996	1	100
13,0 µm	19,0 µm	5	1001	0	100
19,0 µm	27,0 µm	0	1001	0	100
27,0 µm	38,0 µm	0	1001	0	100
38,0 µm	75,0 µm	0	1001	0	100
75,0 µm	107,0 µm	0	1001	0	100
107,0 µm	151,0 µm	0	1001	0	100
151,0 µm	214,0 µm	0	1001	0	100
214,0 µm	302,0 µm	0	1001	0	100
302,0 µm	427,0 µm	0	1001	0	100
427,0 µm	600,0 µm	0	1001	0	100
600,0 µm		0	1001	0	100
