# Supplementary material for: Benchmark dataset of the effect of grain size on strength in the single-phase FCC CrCoNi medium entropy alloy
Source: Data Brief. 2019 Oct 1;27:104592. doi: 10.1016/j.dib.2019.104592 (PMC6812030; doi:10.1016/j.dib.2019.104592)
Supplement: Multimedia component 1 [file mmc1.zip › CrCoNi_1173K_10min/CrCoNi_1173K_10min_d=4.5μm.pdf]

Analysebericht

Aug 23, 2017 10:45:37 AM

powered by imagic.ch

1. cumulative Result 1

Number of images	4
Grain size (ASTM)	12.3
Grain size (G643)	12.3
Grain stretching	85.1 %
Mean chord length	4.5 µm

2. Single Result 1 (CrCoNi Twins grain size_ASTM 900C 10min_00099)

Mean chord length	4.1 µm
Grain size (ASTM)	12.5
Grain size (G643)	12.5
Grain stretching	83.8 %

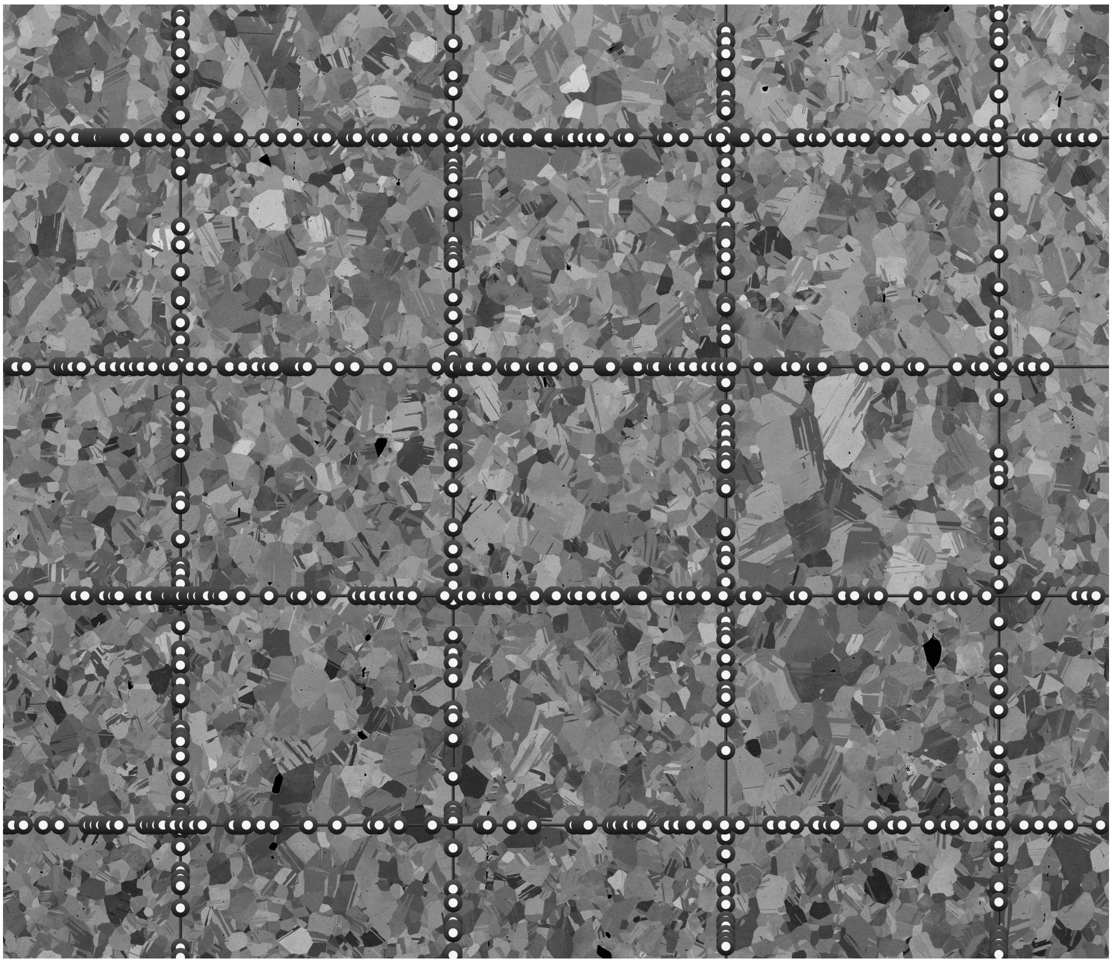

2.1. Statistical Analysis

Statistical Data	Length
Object Count	572
Minimum	0.2 µm
Maximum	24.7 µm
Average	4.1 µm
Standard deviation	3.1 µm
Skewness	0.0
Standard deviation (n-1)	3.1 µm
Variance	9.9 µm²
Variance (n-1)	9.9 µm²
Sum	2'365.3 µm

Statistical Data	Length
Sum of squares	15'428.1 μm²
Sum of cubes	138'104.4 μm³

2.1.1. Chord Lenght Distribution

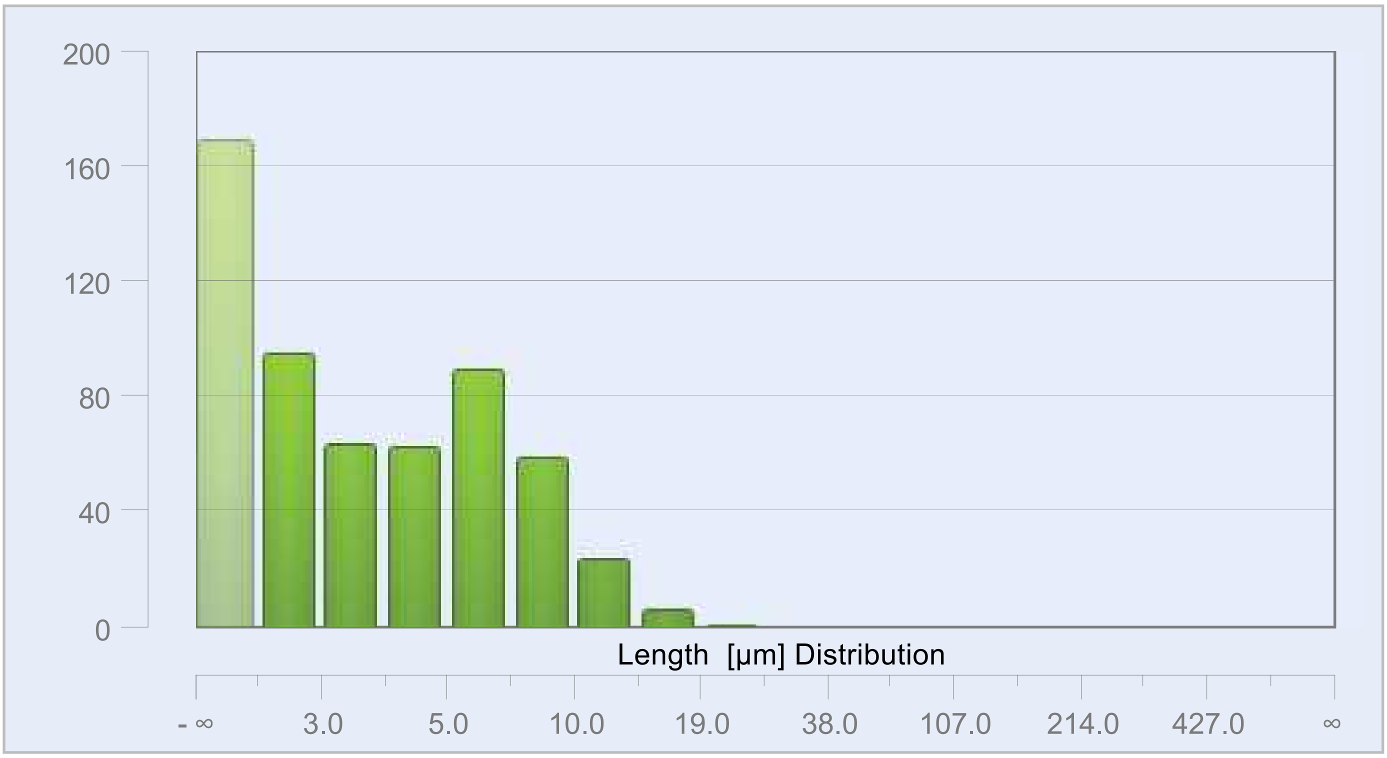

Start	End	Absolute Frequency	Absolute Frequency (accumulated)	Relative Frequency [%]	Relative Frequency (accumulated) [%]
	2.0 μm	169	169	30	30
2.0 μm	3.0 μm	95	264	17	46
3.0 μm	4.0 μm	64	328	11	57
4.0 μm	5.0 μm	63	391	11	68
5.0 μm	7.0 μm	90	481	16	84
7.0 μm	10.0 μm	59	540	10	94
10.0 μm	13.0 μm	24	564	4	99
13.0 μm	19.0 μm	7	571	1	100
19.0 μm	27.0 μm	1	572	0	100
27.0 μm	38.0 μm	0	572	0	100
38.0 μm	75.0 μm	0	572	0	100
75.0 μm	107.0 μm	0	572	0	100
107.0 μm	151.0 μm	0	572	0	100
151.0 μm	214.0 μm	0	572	0	100
214.0 μm	302.0 μm	0	572	0	100
302.0 μm	427.0 μm	0	572	0	100
427.0 μm	600.0 μm	0	572	0	100
600.0 μm		0	572	0	100

3. Single Result 2 (CrCoNi Twins grain size_ASTM 900C 10min_00100)

Mean chord length	4.1 μm
Grain size (ASTM)	12.6
Grain size (G643)	12.5
Grain stretching	86.3 %

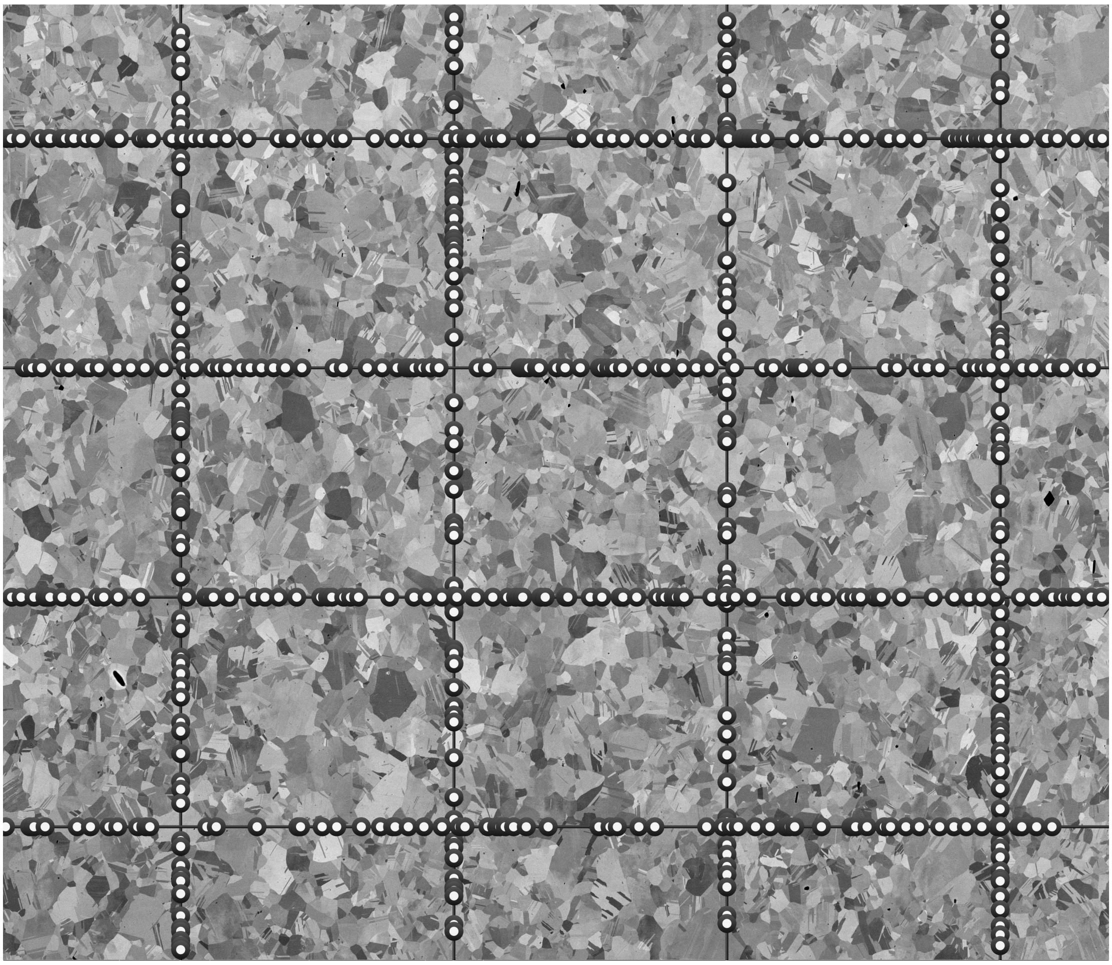

3.1. Statistical Analysis

Statistical Data	Length
Object Count	576
Minimum	0.4 µm
Maximum	18.8 µm
Average	4.1 µm
Standard deviation	3.0 µm
Skewness	0.0
Standard deviation (n-1)	3.0 µm
Variance	8.8 µm²
Variance (n-1)	8.8 µm²
Sum	2'366.8 µm
Sum of squares	14'776.7 µm²
Sum of cubes	124'072.8 µm³

3.1.1. Chord Lenght Distribution

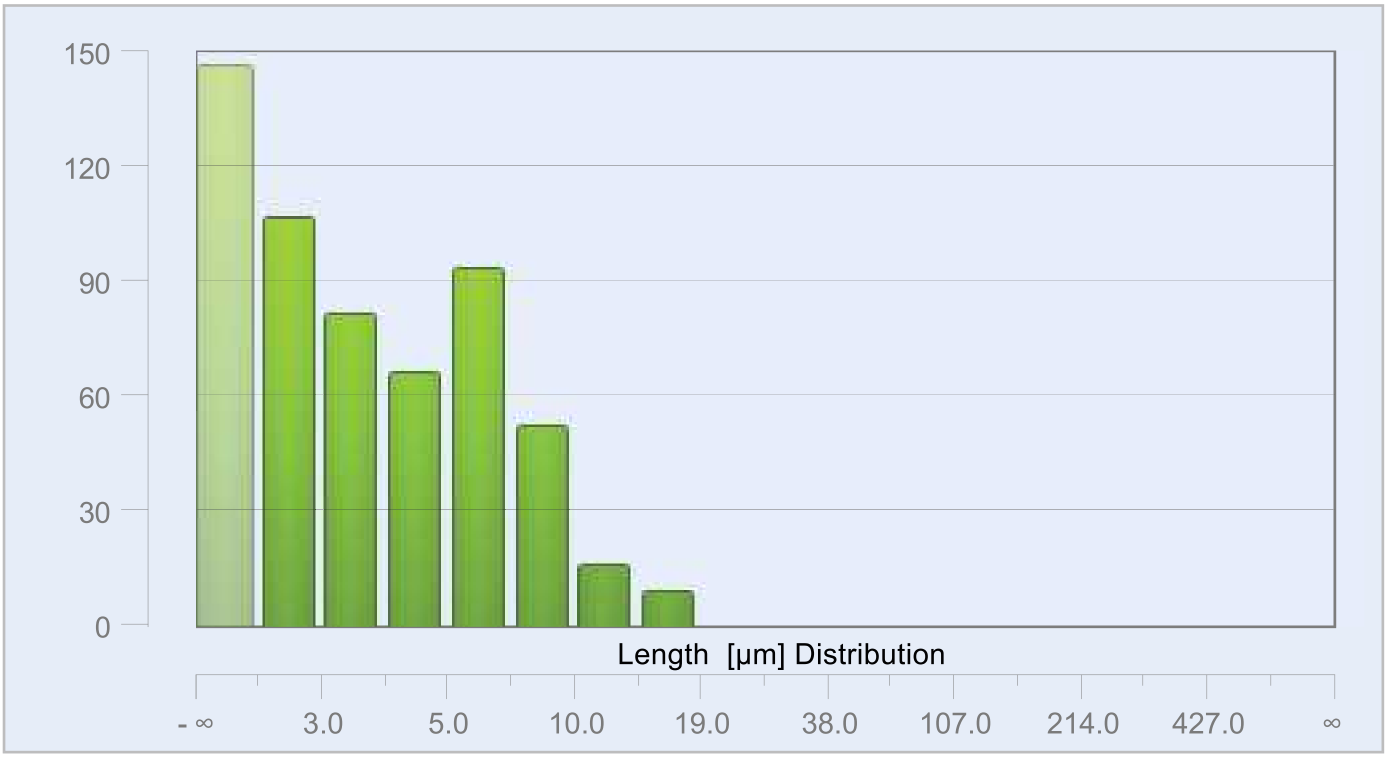

Start	End	Absolute Frequency	Absolute Frequency (accumulated)	Relative Frequency [%]	Relative Frequency (accumulated) [%]
	2.0 µm	146	146	25	25
2.0 µm	3.0 µm	107	253	19	44
3.0 µm	4.0 µm	82	335	14	58
4.0 µm	5.0 µm	67	402	12	70
5.0 µm	7.0 µm	94	496	16	86
7.0 µm	10.0 µm	53	549	9	95
10.0 µm	13.0 µm	17	566	3	98
13.0 µm	19.0 µm	10	576	2	100
19.0 µm	27.0 µm	0	576	0	100
27.0 µm	38.0 µm	0	576	0	100
38.0 µm	75.0 µm	0	576	0	100
75.0 µm	107.0 µm	0	576	0	100
107.0 µm	151.0 µm	0	576	0	100
151.0 µm	214.0 µm	0	576	0	100
214.0 µm	302.0 µm	0	576	0	100
302.0 µm	427.0 µm	0	576	0	100
427.0 µm	600.0 µm	0	576	0	100
600.0 µm		0	576	0	100

4. Single Result 3 (CrCoNi Twins grain size_ASTM 900C 10min_00102)

Mean chord length	5.4 µm
Grain size (ASTM)	11.8
Grain size (G643)	11.7
Grain stretching	86.5 %

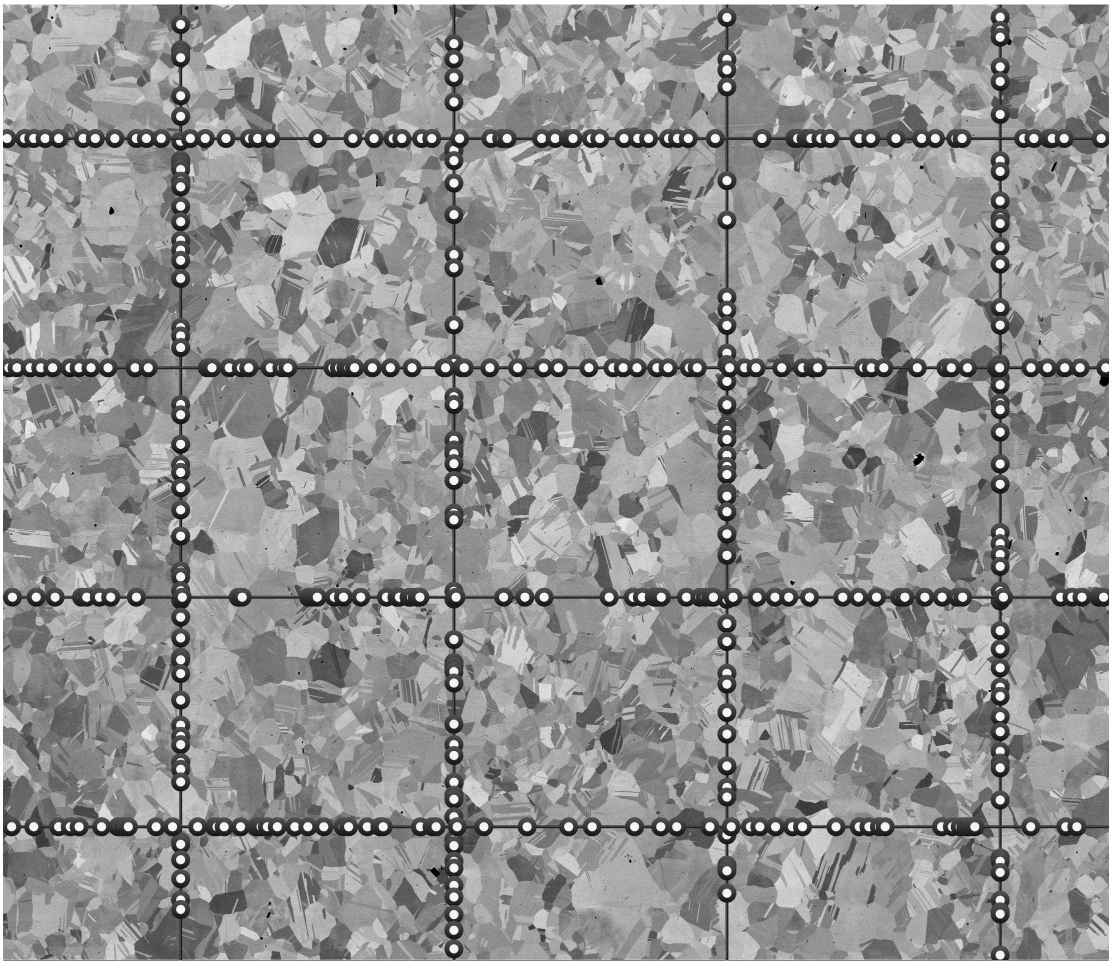

4.1. Statistical Analysis

Statistical Data	Length
Object Count	435
Minimum	0.5 µm
Maximum	26.9 µm
Average	5.4 µm
Standard deviation	4.0 µm
Skewness	0.0
Standard deviation (n-1)	4.0 µm
Variance	15.9 µm²
Variance (n-1)	16.0 µm²
Sum	2'363.2 µm
Sum of squares	19'774.0 µm²
Sum of cubes	226'991.6 µm³

4.1.1. Chord Lenght Distribution

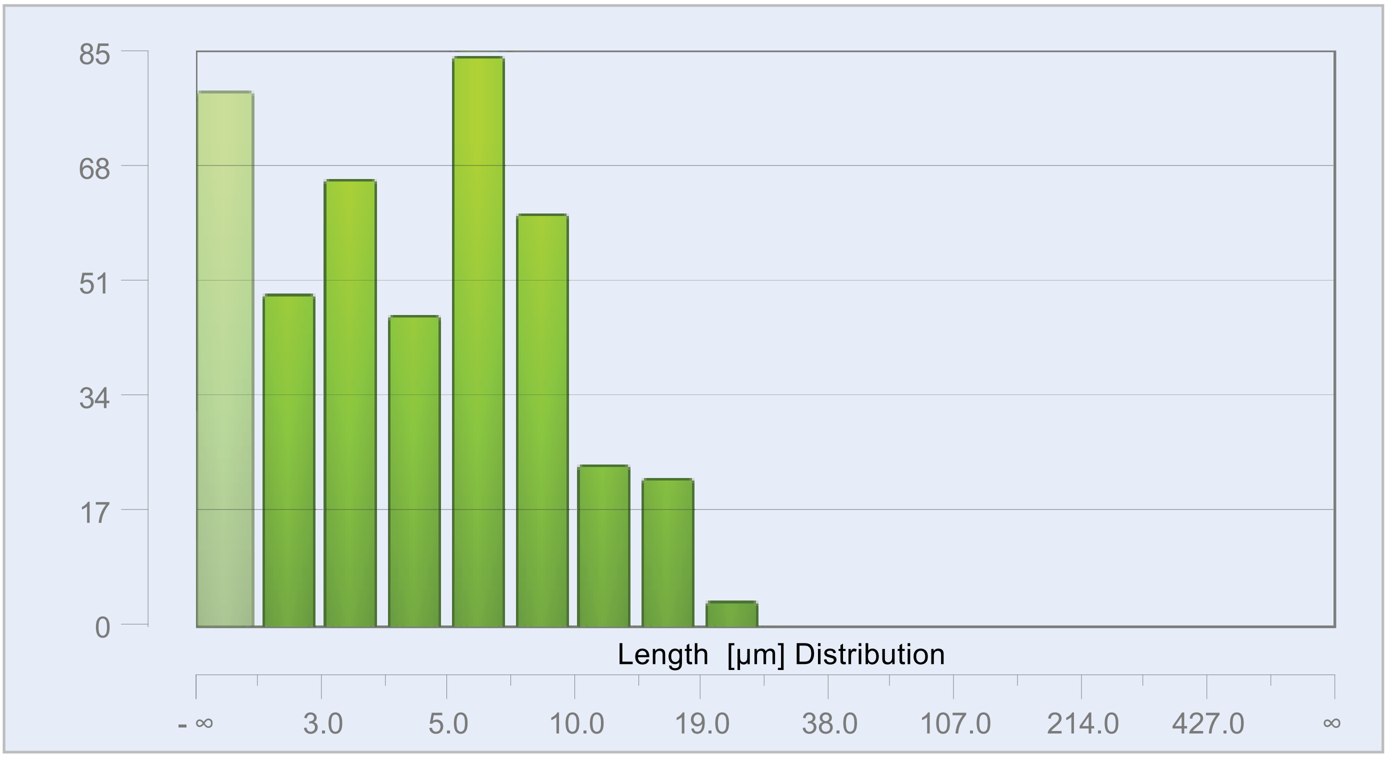

Start	End	Absolute Frequency	Absolute Frequency (accumulated)	Relative Frequency [%]	Relative Frequency (accumulated) [%]
	2.0 µm	79	79	18	18
2.0 µm	3.0 µm	49	128	11	29
3.0 µm	4.0 µm	66	194	15	45
4.0 µm	5.0 µm	46	240	11	55
5.0 µm	7.0 µm	84	324	19	74
7.0 µm	10.0 µm	61	385	14	89
10.0 µm	13.0 µm	24	409	6	94
13.0 µm	19.0 µm	22	431	5	99
19.0 µm	27.0 µm	4	435	1	100
27.0 µm	38.0 µm	0	435	0	100
38.0 µm	75.0 µm	0	435	0	100
75.0 µm	107.0 µm	0	435	0	100
107.0 µm	151.0 µm	0	435	0	100
151.0 µm	214.0 µm	0	435	0	100
214.0 µm	302.0 µm	0	435	0	100
302.0 µm	427.0 µm	0	435	0	100
427.0 µm	600.0 µm	0	435	0	100
600.0 µm		0	435	0	100

5. Single Result 4 (CrCoNi Twins grain size_ASTM 900C 10min_00103)

Mean chord length	4.4 µm
Grain size (ASTM)	12.4
Grain size (G643)	12.3
Grain stretching	84.2 %

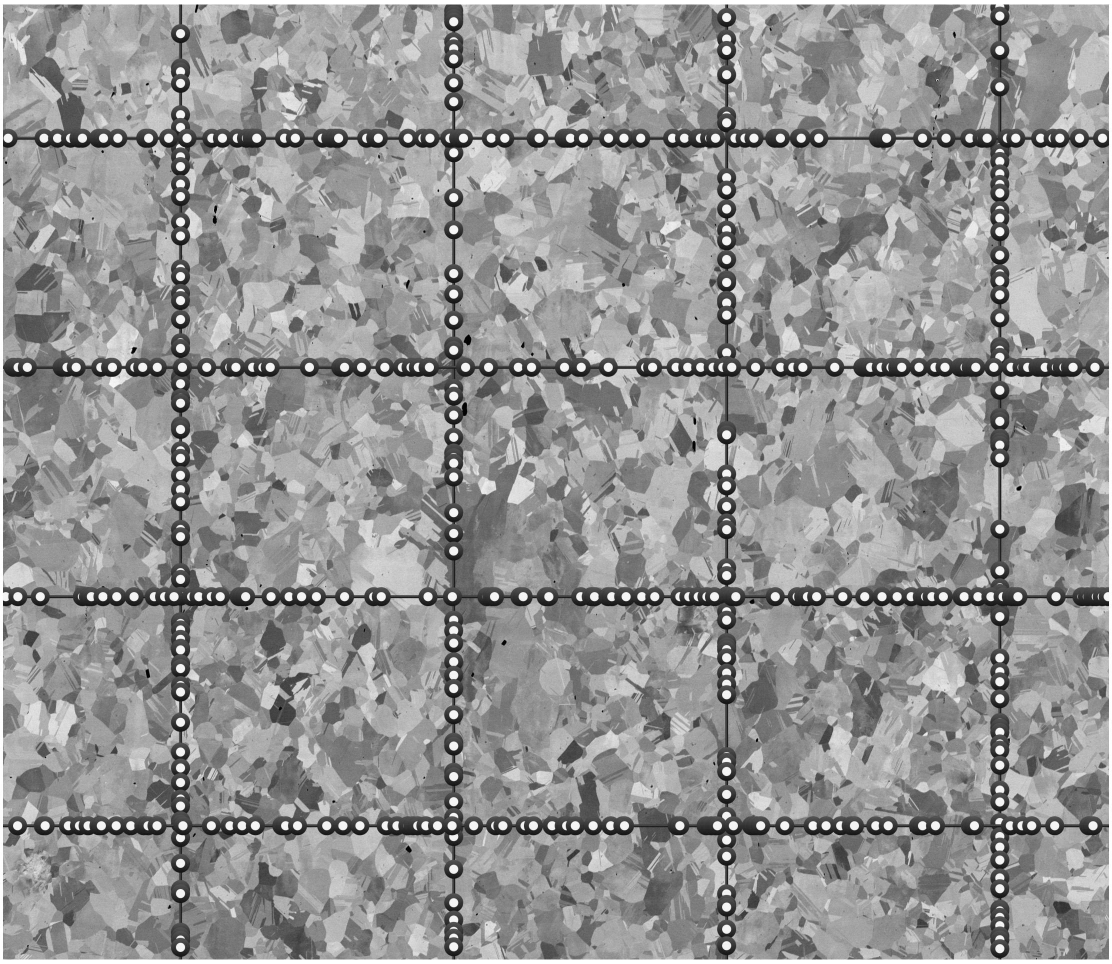

5.1. Statistical Analysis

Statistical Data	Length
Object Count	539
Minimum	0.2 µm
Maximum	20.4 µm
Average	4.4 µm
Standard deviation	3.3 µm
Skewness	0.0
Standard deviation (n-1)	3.3 µm
Variance	10.6 µm²
Variance (n-1)	10.6 µm²
Sum	2'364.7 µm
Sum of squares	16'095.7 µm²
Sum of cubes	148'105.1 µm³

5.1.1. Chord Lenght Distribution

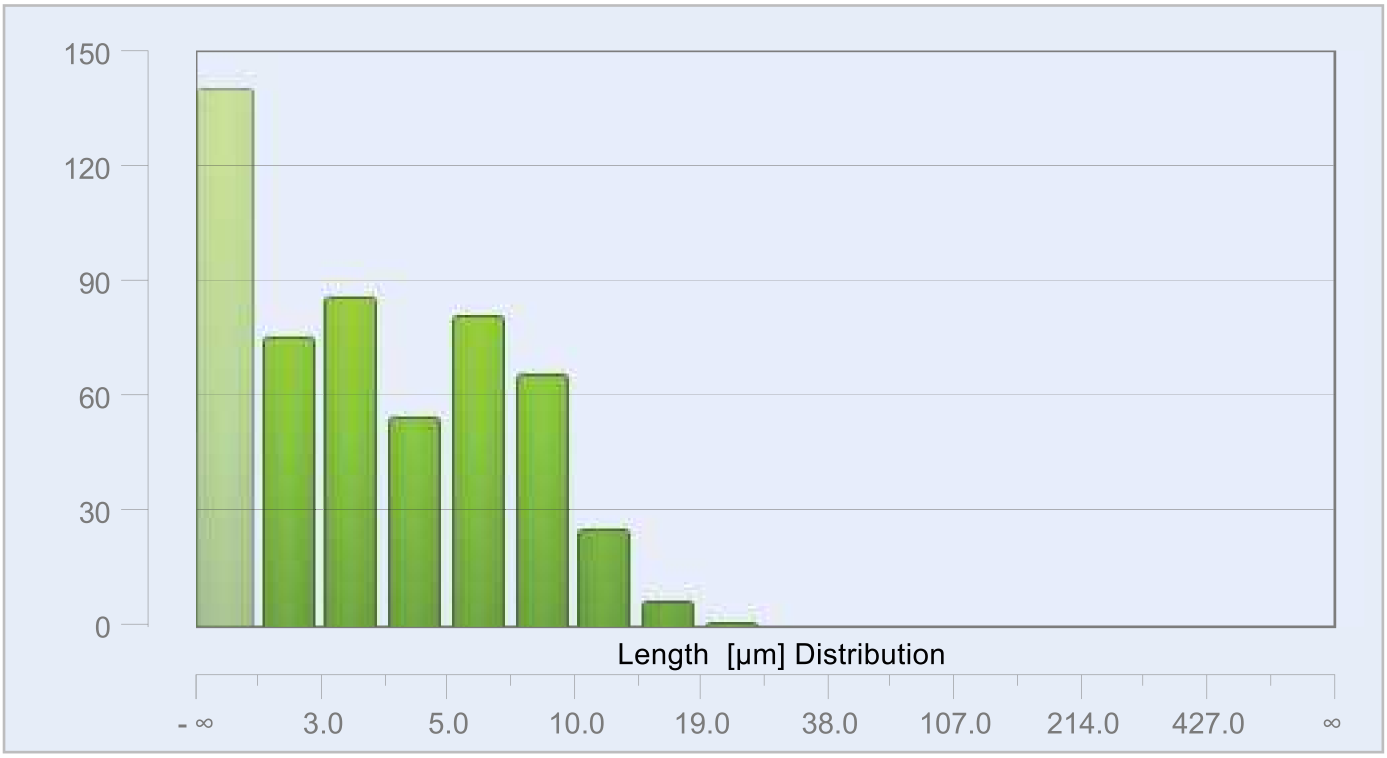

Start	End	Absolute Frequency	Absolute Frequency (accumulated)	Relative Frequency [%]	Relative Frequency (accumulated) [%]
	2.0 µm	140	140	26	26
2.0 µm	3.0 µm	76	216	14	40
3.0 µm	4.0 µm	86	302	16	56
4.0 µm	5.0 µm	55	357	10	66
5.0 µm	7.0 µm	81	438	15	81
7.0 µm	10.0 µm	66	504	12	94
10.0 µm	13.0 µm	26	530	5	98
13.0 µm	19.0 µm	7	537	1	100
19.0 µm	27.0 µm	2	539	0	100
27.0 µm	38.0 µm	0	539	0	100
38.0 µm	75.0 µm	0	539	0	100
75.0 µm	107.0 µm	0	539	0	100
107.0 µm	151.0 µm	0	539	0	100
151.0 µm	214.0 µm	0	539	0	100
214.0 µm	302.0 µm	0	539	0	100
302.0 µm	427.0 µm	0	539	0	100
427.0 µm	600.0 µm	0	539	0	100
600.0 µm		0	539	0	100
