# Supplementary material for: Benchmark dataset of the effect of grain size on strength in the single-phase FCC CrCoNi medium entropy alloy
Source: Data Brief. 2019 Oct 1;27:104592. doi: 10.1016/j.dib.2019.104592 (PMC6812030; doi:10.1016/j.dib.2019.104592)
Supplement: Multimedia component 1 [file mmc1.zip › CrCoNi_1173K_120min/CrCoNi_1173K_120min_c=5.7μm.pdf]

Analysebericht

05.11.2017 12:47:53

powered by imagic.ch

1. cumulative Result 1

Anzahl Bilder	4
Korngröße (ASTM)	11,6
Korngröße (G643)	11,6
Kornstreckung	92,2 %
Mittlere Sehnenlänge	5,7 µm

2. Single Result 1 (CrCoNi - ASTM E 112_CrCoNi_homogenized_8.1mmSW_900°C_120min_00146)

Mittlere Sehnenlänge	5,6 µm
Korngröße (ASTM)	11,7
Korngröße (G643)	11,6
Kornstreckung	83,9 %

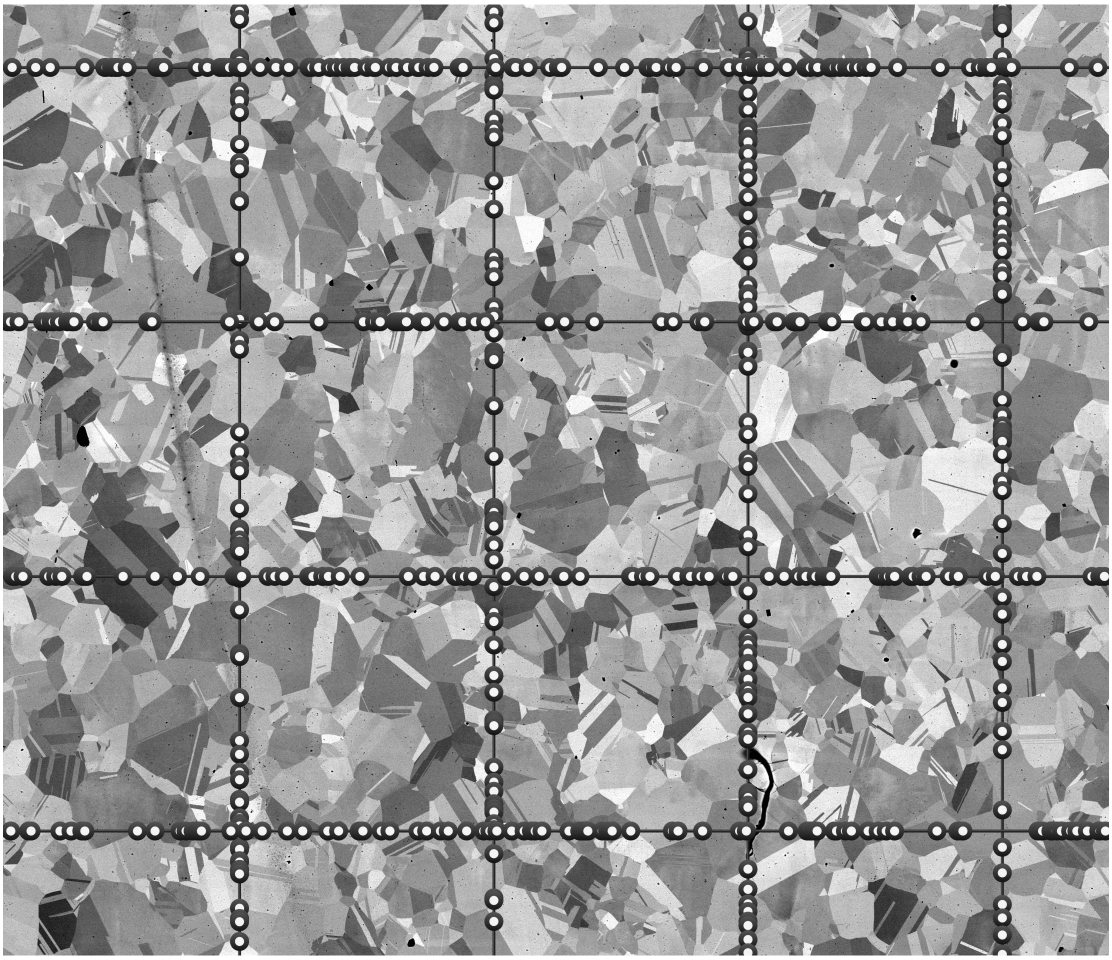

2.1. Statistische Analyse

Statistische Daten	Länge
Anzahl Objekte	558
Minimum	0,3 µm
Maximum	31,6 µm
Mittelwert	5,6 µm
Standardabweichung	5,3 µm
Schiefe	0,0
Standardabweichung (n-1)	5,3 µm
Varianz	28,4 µm²
Varianz (n-1)	28,4 µm²
Summe	3'147,9 µm

Statistische Daten	Länge
Quadratsumme	33'587,9 µm²
Kubiksumme	508'806,1 µm³

2.1.1. Chord Lenght Distribution

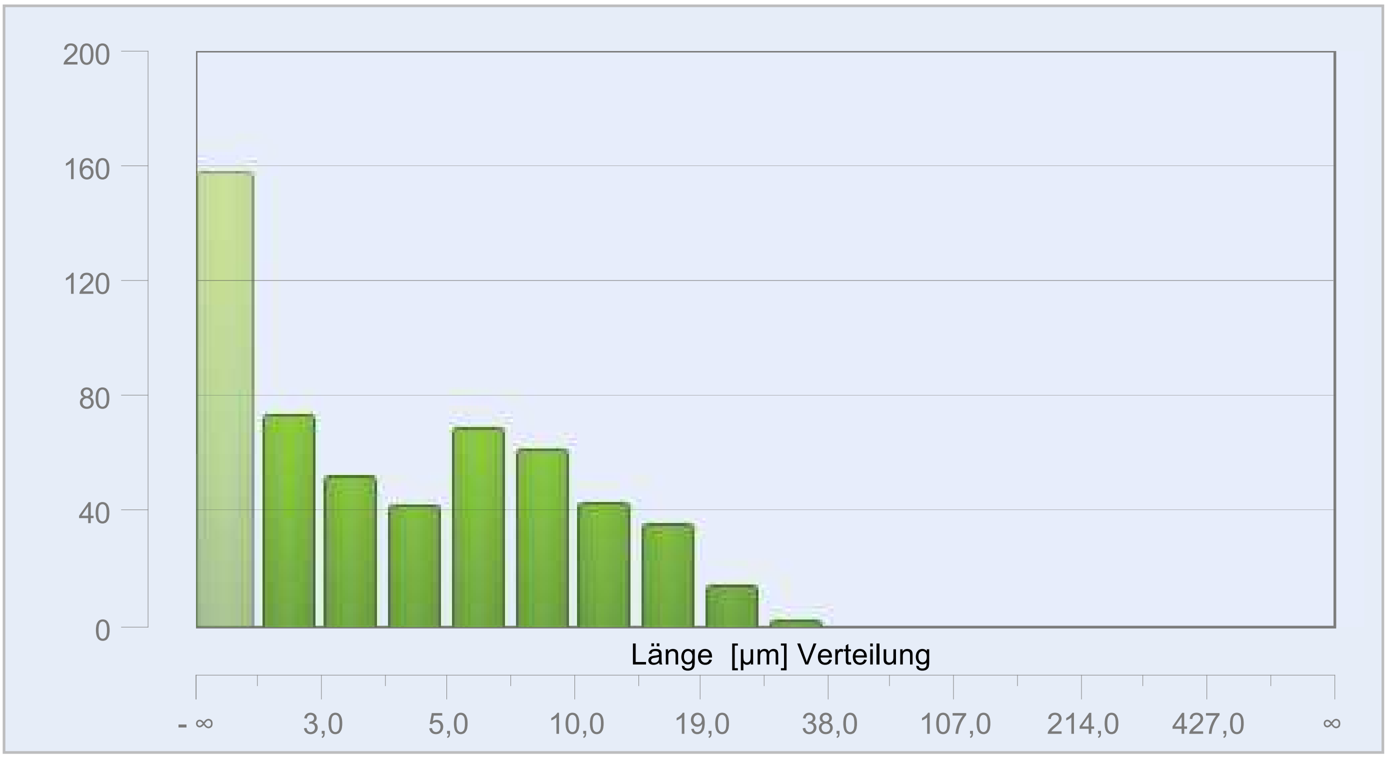

Start	Ende	Absolute Häufigkeit	Absolute Häufigkeit (kumuliert)	Relative Häufigkeit [%]	Relative Häufigkeit (kumuliert) [%]
	2,0 µm	158	158	28	28
2,0 µm	3,0 µm	74	232	13	42
3,0 µm	4,0 µm	53	285	9	51
4,0 µm	5,0 µm	43	328	8	59
5,0 µm	7,0 µm	70	398	13	71
7,0 µm	10,0 µm	62	460	11	82
10,0 µm	13,0 µm	44	504	8	90
13,0 µm	19,0 µm	36	540	6	97
19,0 µm	27,0 µm	15	555	3	99
27,0 µm	38,0 µm	3	558	1	100
38,0 µm	75,0 µm	0	558	0	100
75,0 µm	107,0 µm	0	558	0	100
107,0 µm	151,0 µm	0	558	0	100
151,0 µm	214,0 µm	0	558	0	100
214,0 µm	302,0 µm	0	558	0	100
302,0 µm	427,0 µm	0	558	0	100
427,0 µm	600,0 µm	0	558	0	100
600,0 µm		0	558	0	100

3. Single Result 2 (CrCoNi - ASTM E 112_CrCoNi_homogenized_8.1mmSW_900°C_120min_00147)

Mittlere Sehnenlänge	5,2 µm
Korngröße (ASTM)	11,9
Korngröße (G643)	11,9
Kornstreckung	91,2 %

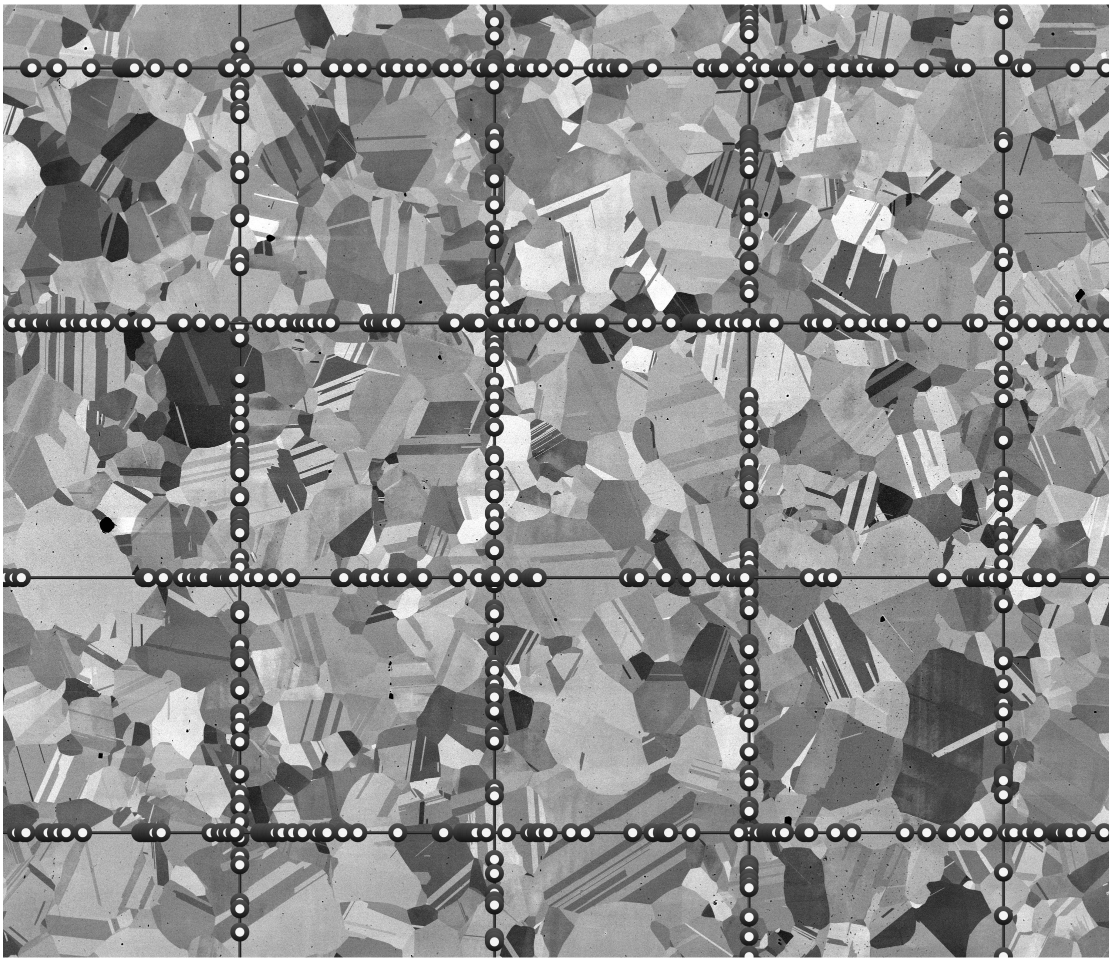

3.1. Statistische Analyse

Statistische Daten	Länge
Anzahl Objekte	609
Minimum	0,2 µm
Maximum	46,9 µm
Mittelwert	5,2 µm
Standardabweichung	5,6 µm
Schiefe	0,0
Standardabweichung (n-1)	5,6 µm
Varianz	31,4 µm²
Varianz (n-1)	31,5 µm²
Summe	3'149,6 µm
Quadratsumme	35'429,6 µm²
Kubiksumme	652'214,6 µm³

3.1.1. Chord Lenght Distribution

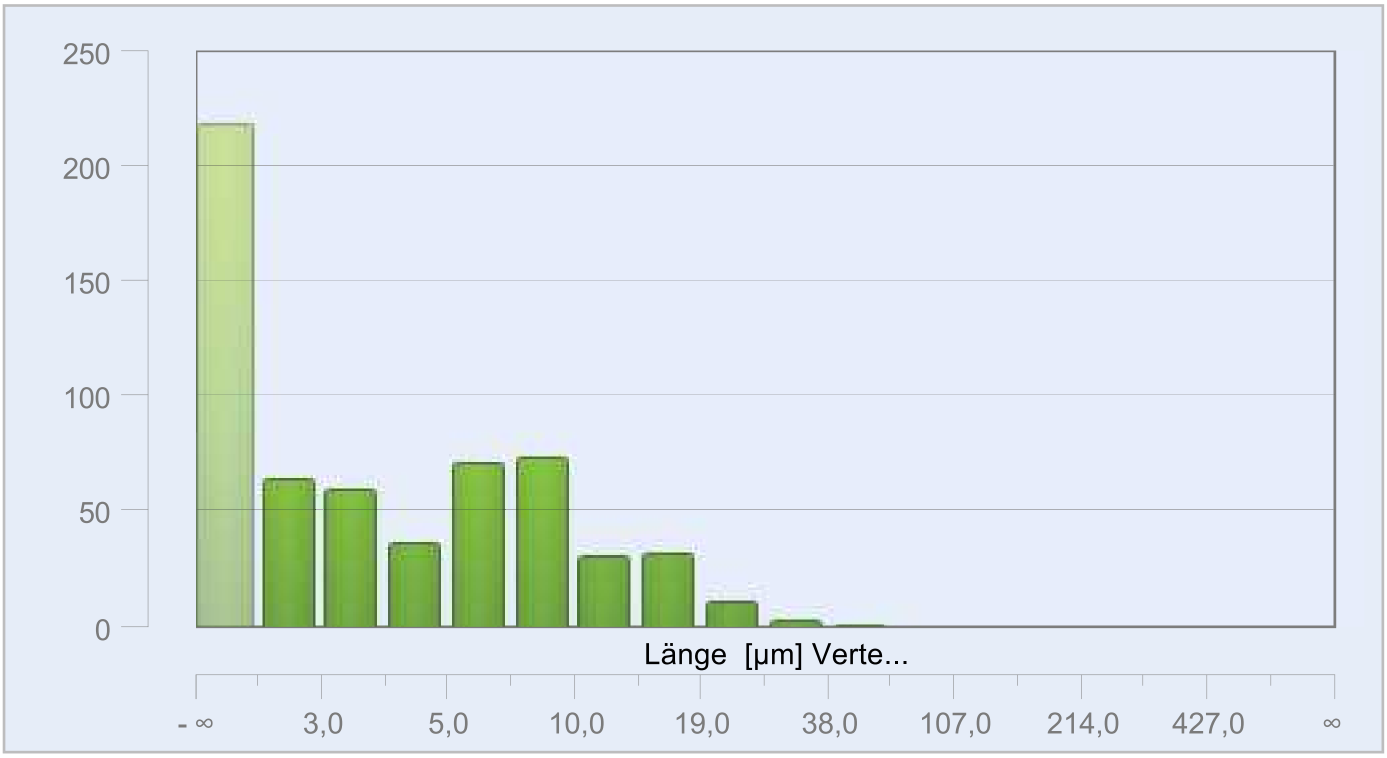

Start	Ende	Absolute Häufigkeit	Absolute Häufigkeit (kumuliert)	Relative Häufigkeit [%]	Relative Häufigkeit (kumuliert) [%]
	2,0 µm	218	218	36	36
2,0 µm	3,0 µm	65	283	11	46
3,0 µm	4,0 µm	60	343	10	56
4,0 µm	5,0 µm	37	380	6	62
5,0 µm	7,0 µm	72	452	12	74
7,0 µm	10,0 µm	74	526	12	86
10,0 µm	13,0 µm	32	558	5	92
13,0 µm	19,0 µm	33	591	5	97
19,0 µm	27,0 µm	12	603	2	99
27,0 µm	38,0 µm	4	607	1	100
38,0 µm	75,0 µm	2	609	0	100
75,0 µm	107,0 µm	0	609	0	100
107,0 µm	151,0 µm	0	609	0	100
151,0 µm	214,0 µm	0	609	0	100
214,0 µm	302,0 µm	0	609	0	100
302,0 µm	427,0 µm	0	609	0	100
427,0 µm	600,0 µm	0	609	0	100
600,0 µm		0	609	0	100

4. Single Result 3 (CrCoNi - ASTM E 112_CrCoNi_homogenized_8.1mmSW_900°C_120min_00148)

Mittlere Sehnenlänge	6,1 µm
Korngröße (ASTM)	11,4
Korngröße (G643)	11,4
Kornstreckung	99 %

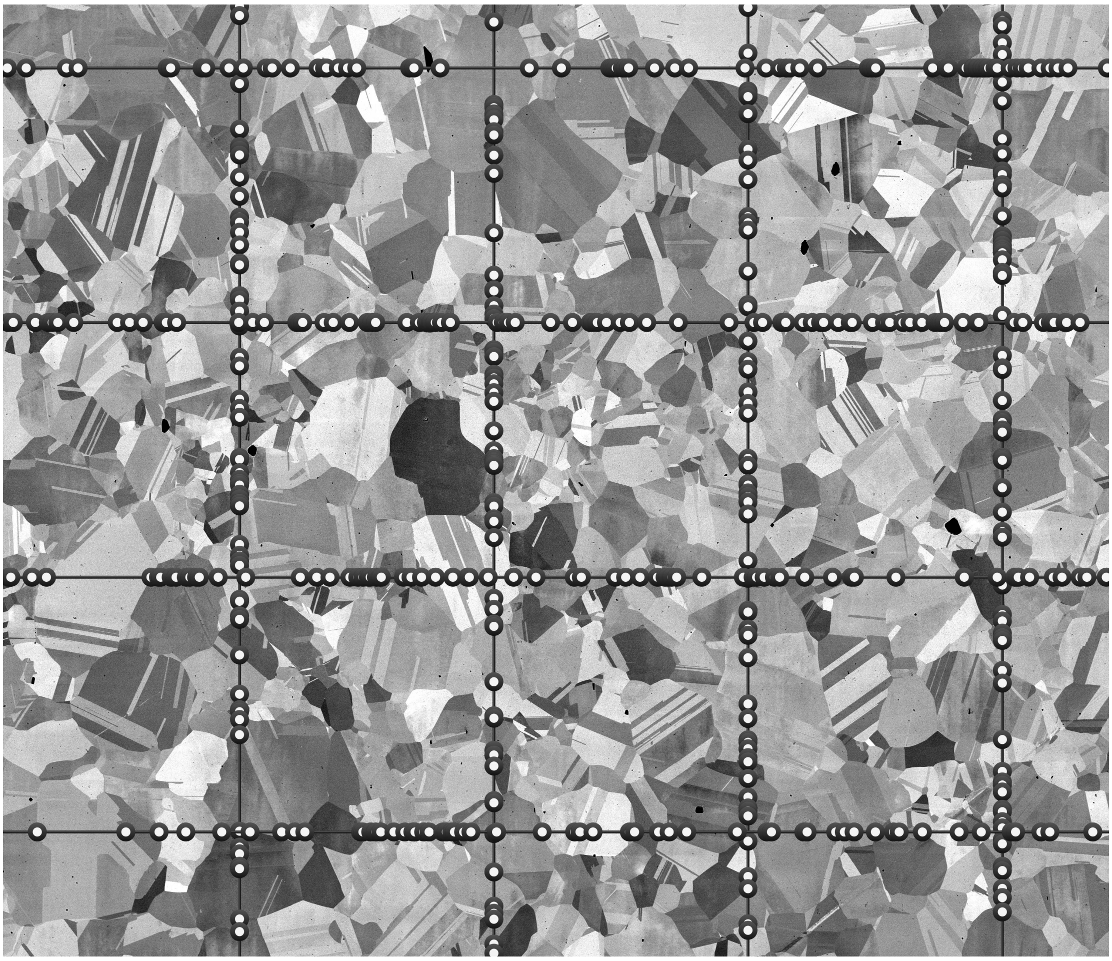

4.1. Statistische Analyse

Statistische Daten	Länge
Anzahl Objekte	513
Minimum	0,2 µm
Maximum	40,0 µm
Mittelwert	6,1 µm
Standardabweichung	6,1 µm
Schiefe	0,0
Standardabweichung (n-1)	6,1 µm
Varianz	37,0 µm²
Varianz (n-1)	37,1 µm²
Summe	3'147,9 µm
Quadratsumme	38'313,7 µm²
Kubiksumme	697'609,4 µm³

4.1.1. Chord Lenght Distribution

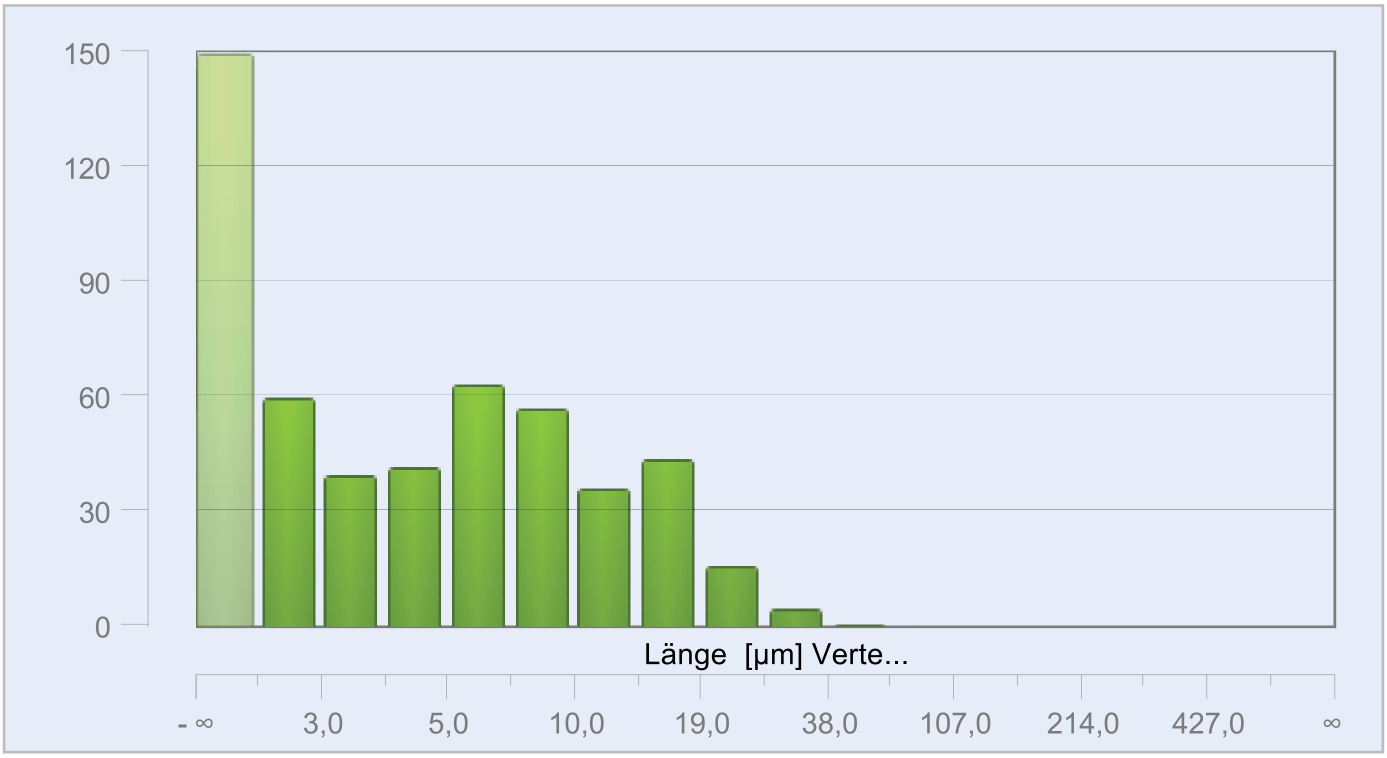

Start	Ende	Absolute Häufigkeit	Absolute Häufigkeit (kumuliert)	Relative Häufigkeit [%]	Relative Häufigkeit (kumuliert) [%]
	2,0 µm	149	149	29	29
2,0 µm	3,0 µm	60	209	12	41
3,0 µm	4,0 µm	40	249	8	49
4,0 µm	5,0 µm	42	291	8	57
5,0 µm	7,0 µm	63	354	12	69
7,0 µm	10,0 µm	57	411	11	80
10,0 µm	13,0 µm	36	447	7	87
13,0 µm	19,0 µm	44	491	9	96
19,0 µm	27,0 µm	16	507	3	99
27,0 µm	38,0 µm	5	512	1	100
38,0 µm	75,0 µm	1	513	0	100
75,0 µm	107,0 µm	0	513	0	100
107,0 µm	151,0 µm	0	513	0	100
151,0 µm	214,0 µm	0	513	0	100
214,0 µm	302,0 µm	0	513	0	100
302,0 µm	427,0 µm	0	513	0	100
427,0 µm	600,0 µm	0	513	0	100
600,0 µm		0	513	0	100

5. Single Result 4 (CrCoNi - ASTM E 112_CrCoNi_homogenized_8.1mmSW_900°C_120min_00149)

Mittlere Sehnenlänge	5,8 µm
Korngröße (ASTM)	11,6
Korngröße (G643)	11,5
Kornstreckung	95,8 %

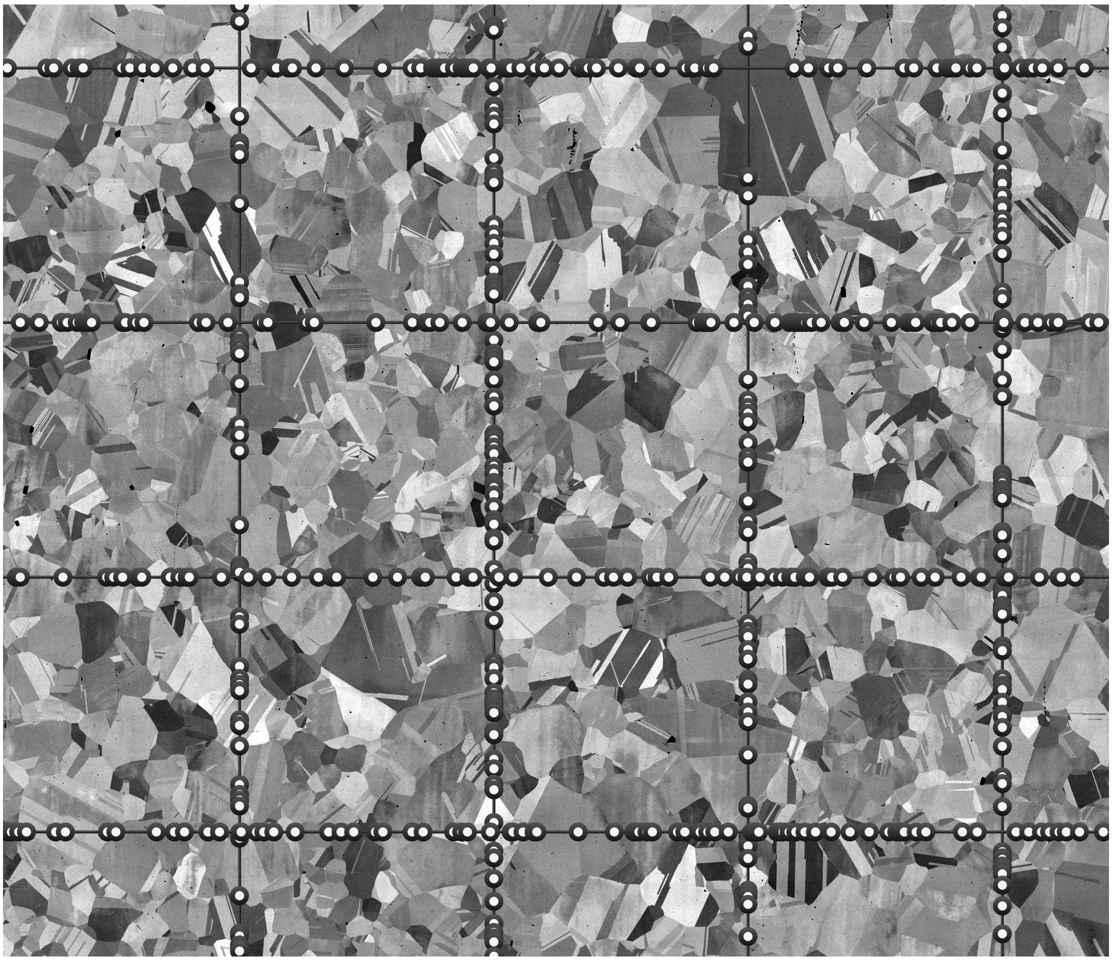

5.1. Statistische Analyse

Statistische Daten	Länge
Anzahl Objekte	540
Minimum	0,2 µm
Maximum	49,8 µm
Mittelwert	5,8 µm
Standardabweichung	5,5 µm
Schiefe	0,0
Standardabweichung (n-1)	5,5 µm
Varianz	30,7 µm²
Varianz (n-1)	30,8 µm²
Summe	3'147,9 µm
Quadratsumme	34'940,6 µm²
Kubiksumme	619'479,0 µm³

5.1.1. Chord Lenght Distribution

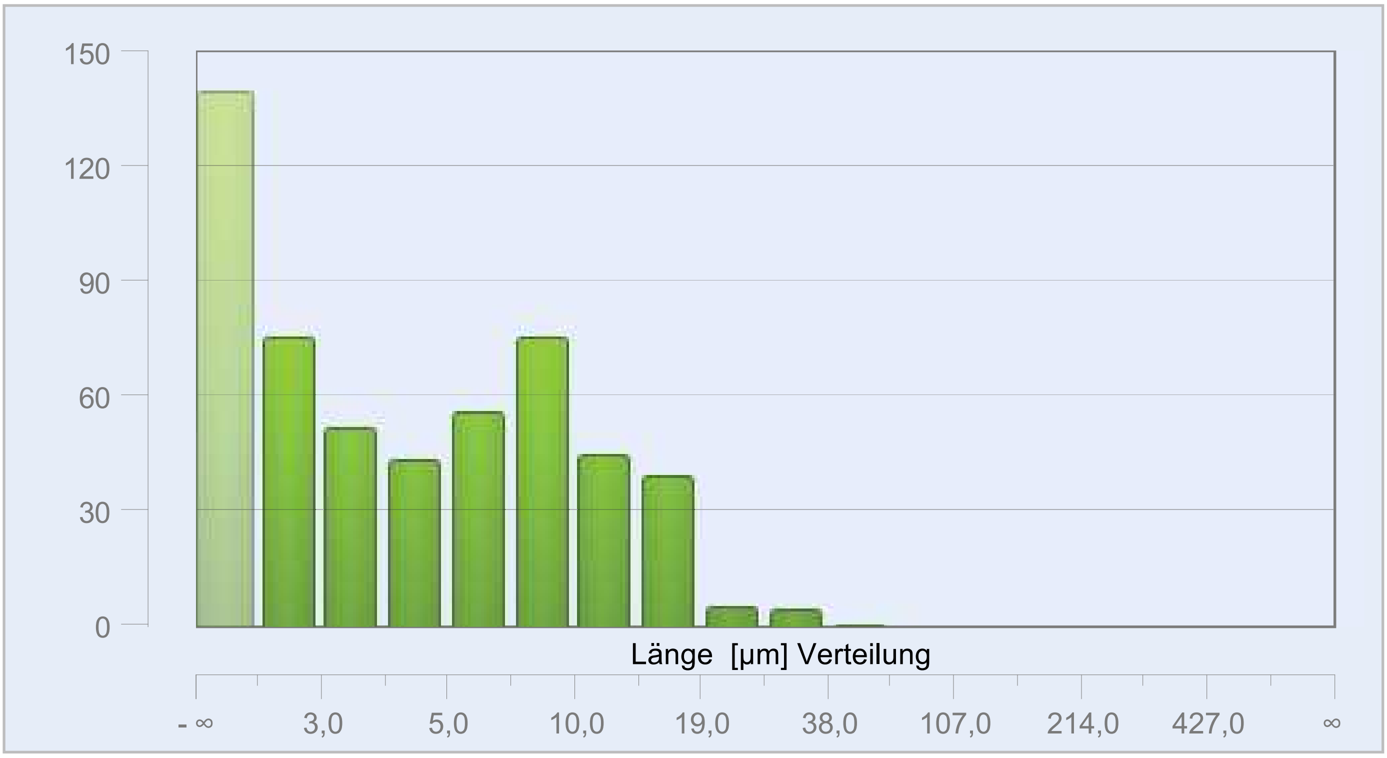

Start	Ende	Absolute Häufigkeit	Absolute Häufigkeit (kumuliert)	Relative Häufigkeit [%]	Relative Häufigkeit (kumuliert) [%]
	2,0 µm	139	139	26	26
2,0 µm	3,0 µm	76	215	14	40
3,0 µm	4,0 µm	52	267	10	49
4,0 µm	5,0 µm	44	311	8	58
5,0 µm	7,0 µm	56	367	10	68
7,0 µm	10,0 µm	76	443	14	82
10,0 µm	13,0 µm	45	488	8	90
13,0 µm	19,0 µm	40	528	7	98
19,0 µm	27,0 µm	6	534	1	99
27,0 µm	38,0 µm	5	539	1	100
38,0 µm	75,0 µm	1	540	0	100
75,0 µm	107,0 µm	0	540	0	100
107,0 µm	151,0 µm	0	540	0	100
151,0 µm	214,0 µm	0	540	0	100
214,0 µm	302,0 µm	0	540	0	100
302,0 µm	427,0 µm	0	540	0	100
427,0 µm	600,0 µm	0	540	0	100
600,0 µm		0	540	0	100
