# Supplementary material for: Benchmark dataset of the effect of grain size on strength in the single-phase FCC CrCoNi medium entropy alloy
Source: Data Brief. 2019 Oct 1;27:104592. doi: 10.1016/j.dib.2019.104592 (PMC6812030; doi:10.1016/j.dib.2019.104592)
Supplement: Multimedia component 1 [file mmc1.zip › CrCoNi_1173K_120min/CrCoNi_1173K_120min_d=13μm.pdf]

Analysebericht

Aug 24, 2017 10:23:24 AM

powered by imagic.ch

1. cumulative Result 1

Number of images	4
Grain size (ASTM)	9.4
Grain size (G643)	9.3
Grain stretching	99.6 %
Mean chord length	12.4 µm

2. Single Result 1 (CrCoNi Twins grain size_ASTM 900C 120min_00123)

Mean chord length	12.8 µm
Grain size (ASTM)	9.3
Grain size (G643)	9.2
Grain stretching	82.7 %

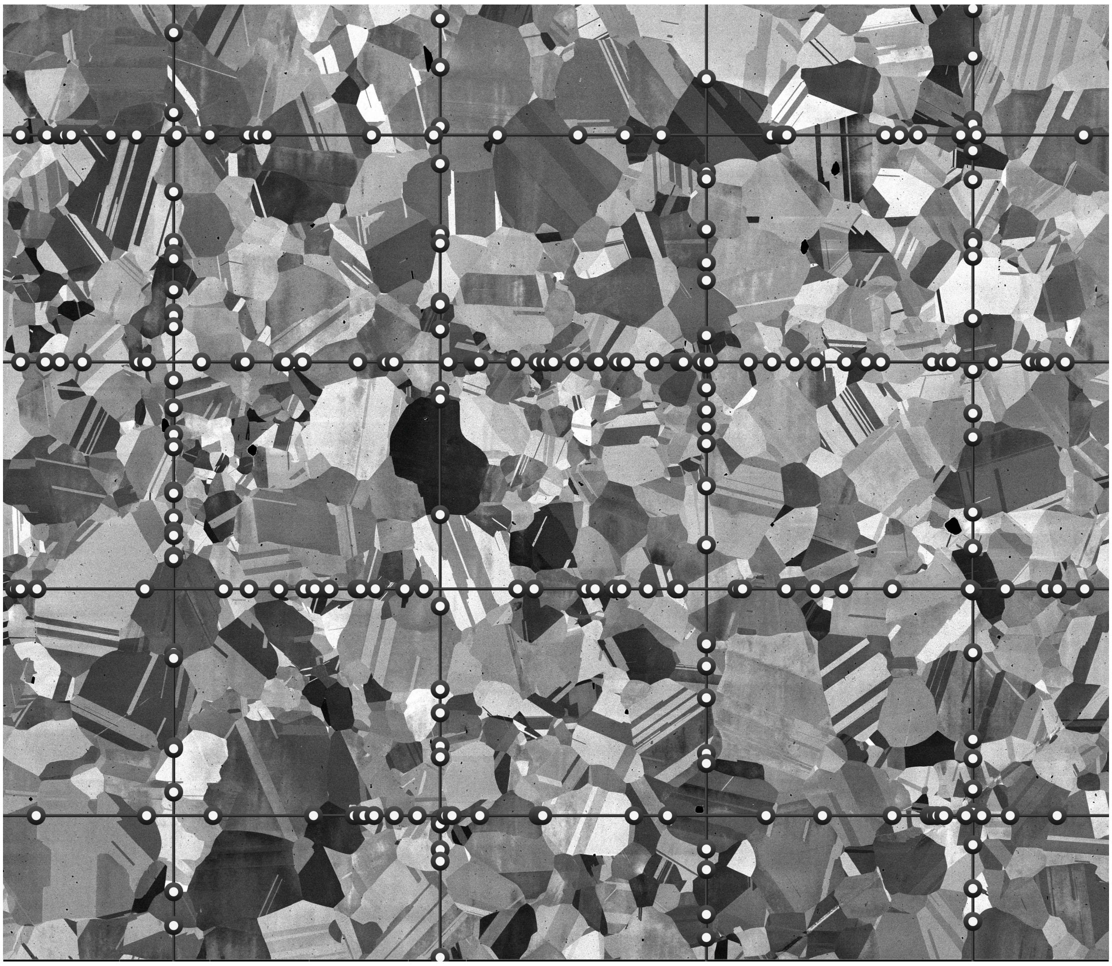

2.1. Statistical Analysis

Statistical Data	Length
Object Count	247
Minimum	0.6 µm
Maximum	44.3 µm
Average	12.8 µm
Standard deviation	10.4 µm
Skewness	0.0
Standard deviation (n-1)	10.4 µm
Variance	107.9 µm²
Variance (n-1)	108.3 µm²
Sum	3'161.1 µm

Statistical Data	Length
Sum of squares	67'102.3 µm²
Sum of cubes	1'849'445.2 µm³

2.1.1. Chord Lenght Distribution

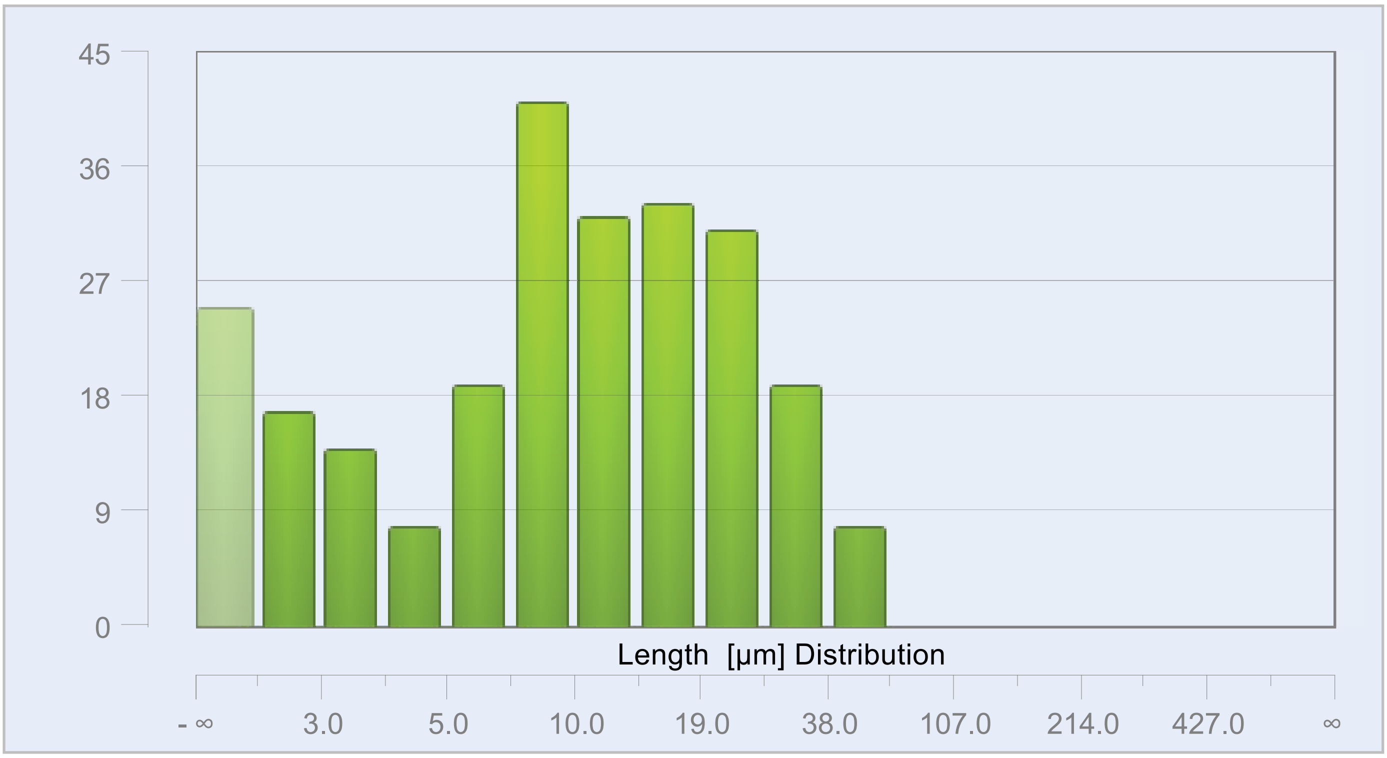

Start	End	Absolute Frequency	Absolute Frequency (accumulated)	Relative Frequency [%]	Relative Frequency (accumulated) [%]
	2.0 µm	25	25	10	10
2.0 µm	3.0 µm	17	42	7	17
3.0 µm	4.0 µm	14	56	6	23
4.0 µm	5.0 µm	8	64	3	26
5.0 µm	7.0 µm	19	83	8	34
7.0 µm	10.0 µm	41	124	17	50
10.0 µm	13.0 µm	32	156	13	63
13.0 µm	19.0 µm	33	189	13	77
19.0 µm	27.0 µm	31	220	13	89
27.0 µm	38.0 µm	19	239	8	97
38.0 µm	75.0 µm	8	247	3	100
75.0 µm	107.0 µm	0	247	0	100
107.0 µm	151.0 µm	0	247	0	100
151.0 µm	214.0 µm	0	247	0	100
214.0 µm	302.0 µm	0	247	0	100
302.0 µm	427.0 µm	0	247	0	100
427.0 µm	600.0 µm	0	247	0	100
600.0 µm		0	247	0	100

3. Single Result 2 (CrCoNi Twins grain size_ASTM 900C 120min_00124)

Mean chord length	14.9 µm
Grain size (ASTM)	8.8
Grain size (G643)	8.8
Grain stretching	95.1 %

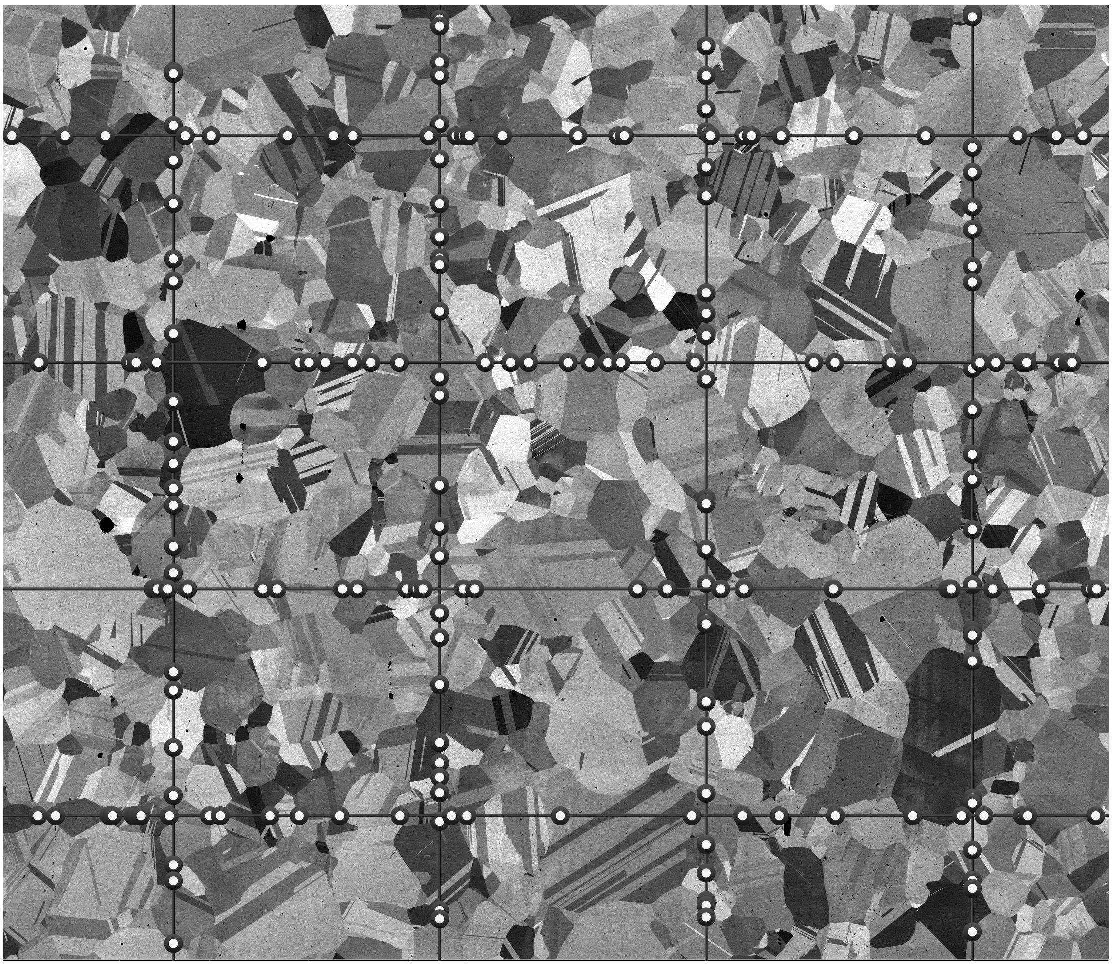

3.1. Statistical Analysis

Statistical Data	Length
Object Count	212
Minimum	0.7 µm
Maximum	62.4 µm
Average	14.9 µm
Standard deviation	11.5 µm
Skewness	0.0
Standard deviation (n-1)	11.6 µm
Variance	133.3 µm²
Variance (n-1)	133.9 µm²
Sum	3'159.0 µm
Sum of squares	75'336.5 µm²
Sum of cubes	2'421'910.6 µm³

3.1.1. Chord Lenght Distribution

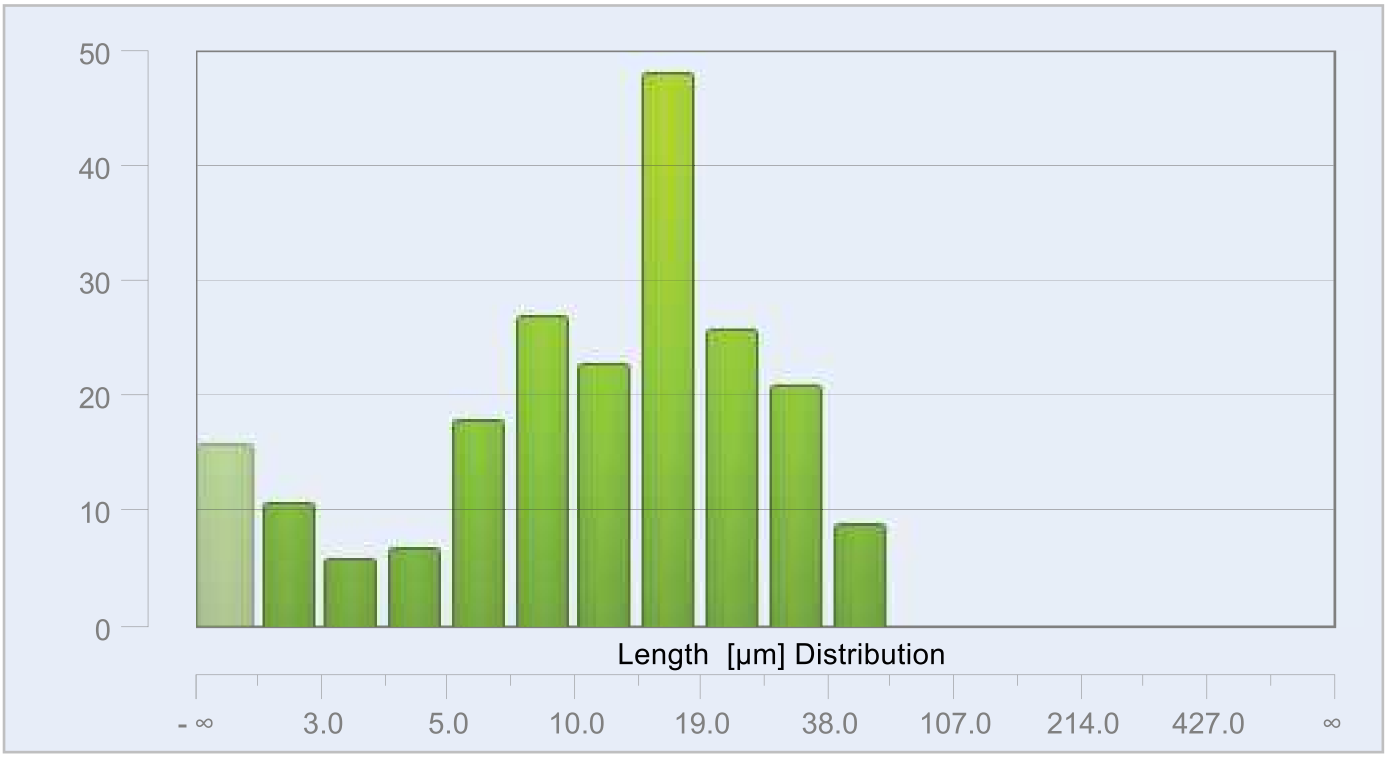

Start	End	Absolute Frequency	Absolute Frequency (accumulated)	Relative Frequency [%]	Relative Frequency (accumulated) [%]
	2.0 µm	16	16	8	8
2.0 µm	3.0 µm	11	27	5	13
3.0 µm	4.0 µm	6	33	3	16
4.0 µm	5.0 µm	7	40	3	19
5.0 µm	7.0 µm	18	58	8	27
7.0 µm	10.0 µm	27	85	13	40
10.0 µm	13.0 µm	23	108	11	51
13.0 µm	19.0 µm	48	156	23	74
19.0 µm	27.0 µm	26	182	12	86
27.0 µm	38.0 µm	21	203	10	96
38.0 µm	75.0 µm	9	212	4	100
75.0 µm	107.0 µm	0	212	0	100
107.0 µm	151.0 µm	0	212	0	100
151.0 µm	214.0 µm	0	212	0	100
214.0 µm	302.0 µm	0	212	0	100
302.0 µm	427.0 µm	0	212	0	100
427.0 µm	600.0 µm	0	212	0	100
600.0 µm		0	212	0	100

4. Single Result 3 (CrCoNi Twins grain size_ASTM 900C 120min_00125)

Mean chord length	11.4 µm
Grain size (ASTM)	9.6
Grain size (G643)	9.6
Grain stretching	93.7 %

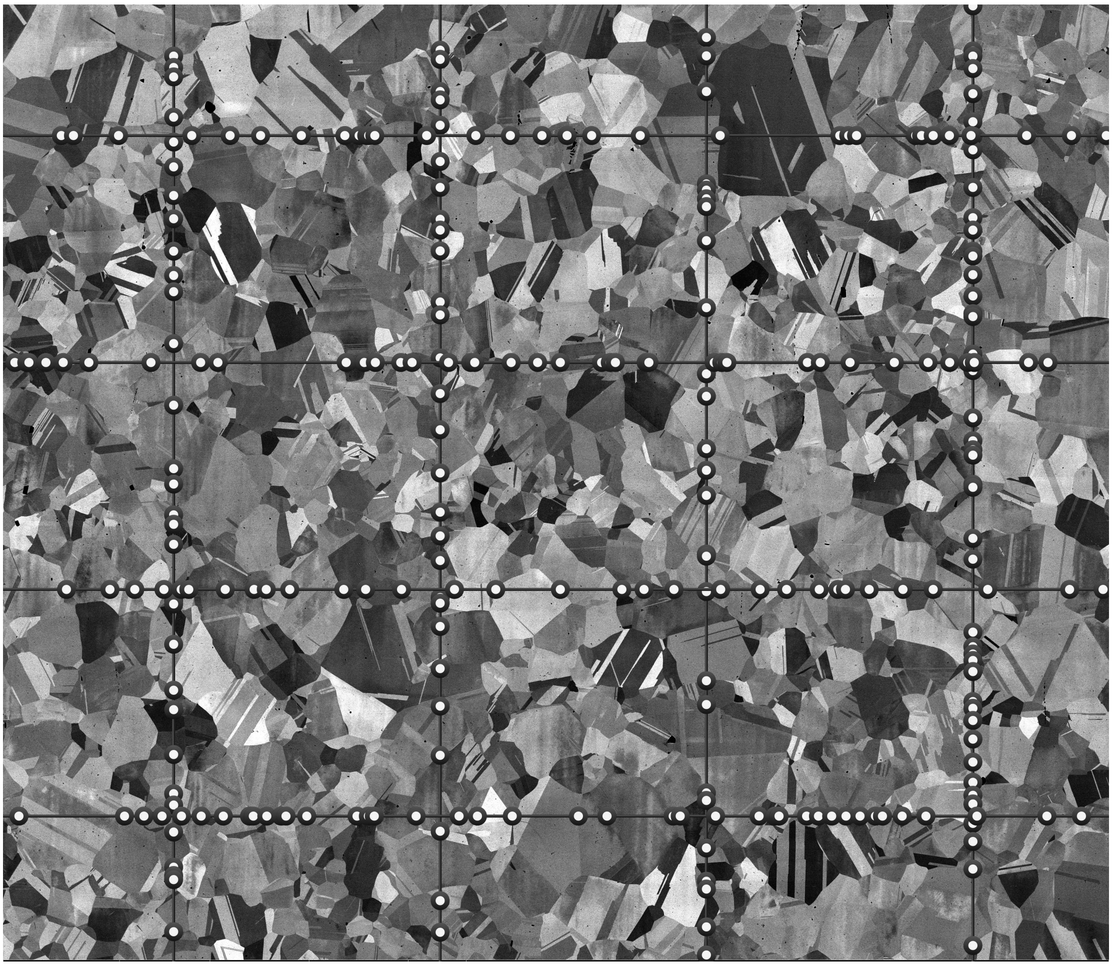

4.1. Statistical Analysis

Statistical Data	Length
Object Count	277
Minimum	0.5 µm
Maximum	49.0 µm
Average	11.4 µm
Standard deviation	8.2 µm
Skewness	0.0
Standard deviation (n-1)	8.2 µm
Variance	67.6 µm²
Variance (n-1)	67.8 µm²
Sum	3'152.9 µm
Sum of squares	54'601.2 µm²
Sum of cubes	1'248'746.7 µm³

4.1.1. Chord Lenght Distribution

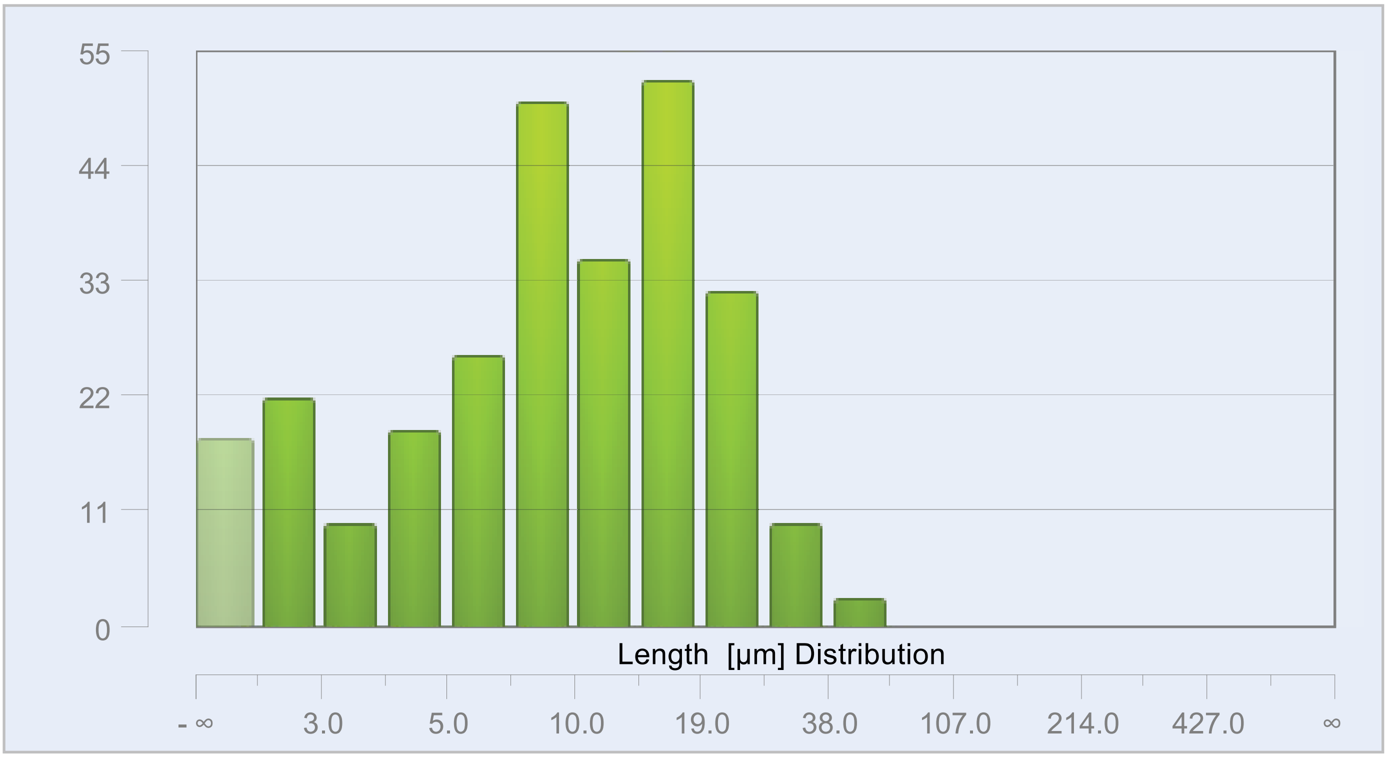

Start	End	Absolute Frequency	Absolute Frequency (accumulated)	Relative Frequency [%]	Relative Frequency (accumulated) [%]
	2.0 µm	18	18	6	6
2.0 µm	3.0 µm	22	40	8	14
3.0 µm	4.0 µm	10	50	4	18
4.0 µm	5.0 µm	19	69	7	25
5.0 µm	7.0 µm	26	95	9	34
7.0 µm	10.0 µm	50	145	18	52
10.0 µm	13.0 µm	35	180	13	65
13.0 µm	19.0 µm	52	232	19	84
19.0 µm	27.0 µm	32	264	12	95
27.0 µm	38.0 µm	10	274	4	99
38.0 µm	75.0 µm	3	277	1	100
75.0 µm	107.0 µm	0	277	0	100
107.0 µm	151.0 µm	0	277	0	100
151.0 µm	214.0 µm	0	277	0	100
214.0 µm	302.0 µm	0	277	0	100
302.0 µm	427.0 µm	0	277	0	100
427.0 µm	600.0 µm	0	277	0	100
600.0 µm		0	277	0	100

5. Single Result 4 (CrCoNi Twins grain size_ASTM 900C 120min_00126)

Mean chord length	11.2 µm
Grain size (ASTM)	9.7
Grain size (G643)	9.6
Grain stretching	95.3 %

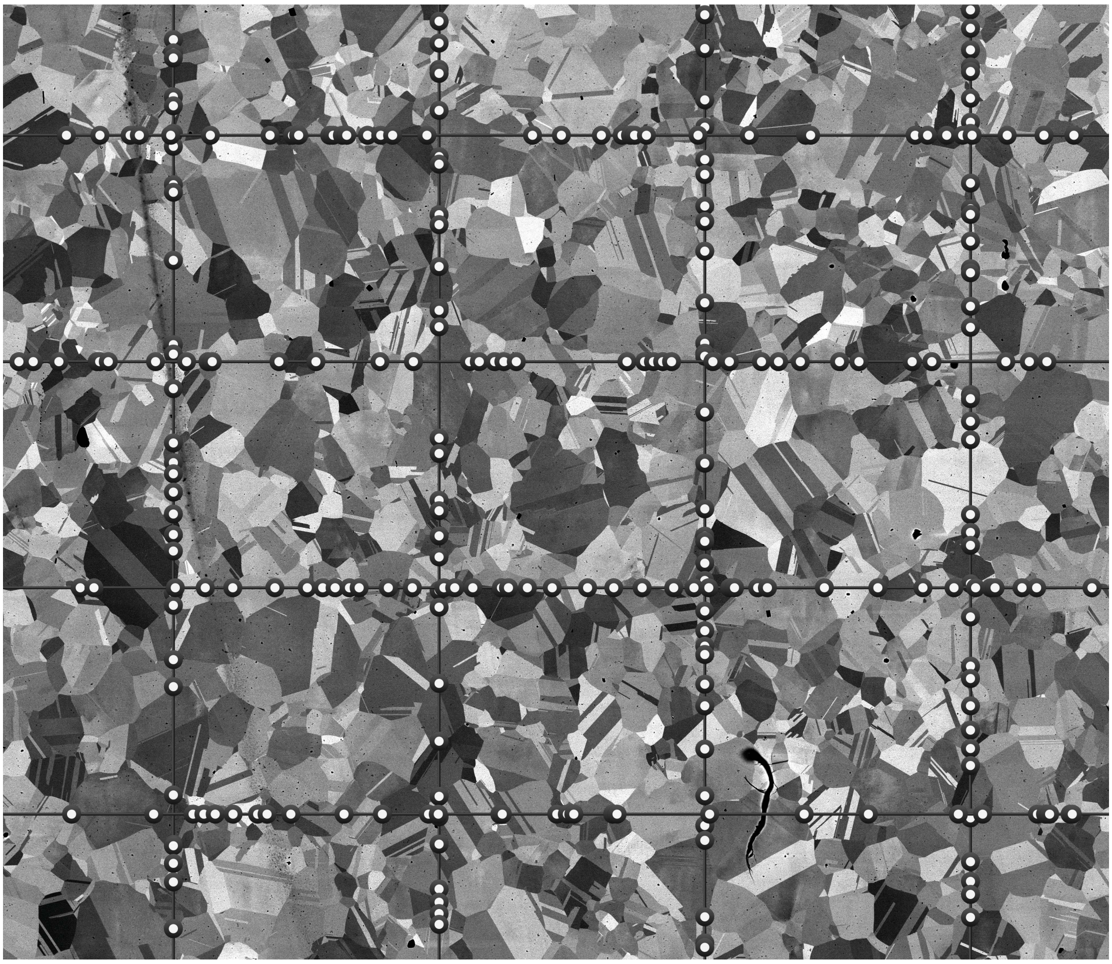

5.1. Statistical Analysis

Statistical Data	Length
Object Count	282
Minimum	0.5 µm
Maximum	42.5 µm
Average	11.2 µm
Standard deviation	8.4 µm
Skewness	0.0
Standard deviation (n-1)	8.5 µm
Variance	71.3 µm²
Variance (n-1)	71.5 µm²
Sum	3'147.6 µm
Sum of squares	55'227.9 µm²
Sum of cubes	1'297'841.8 µm³

5.1.1. Chord Lenght Distribution

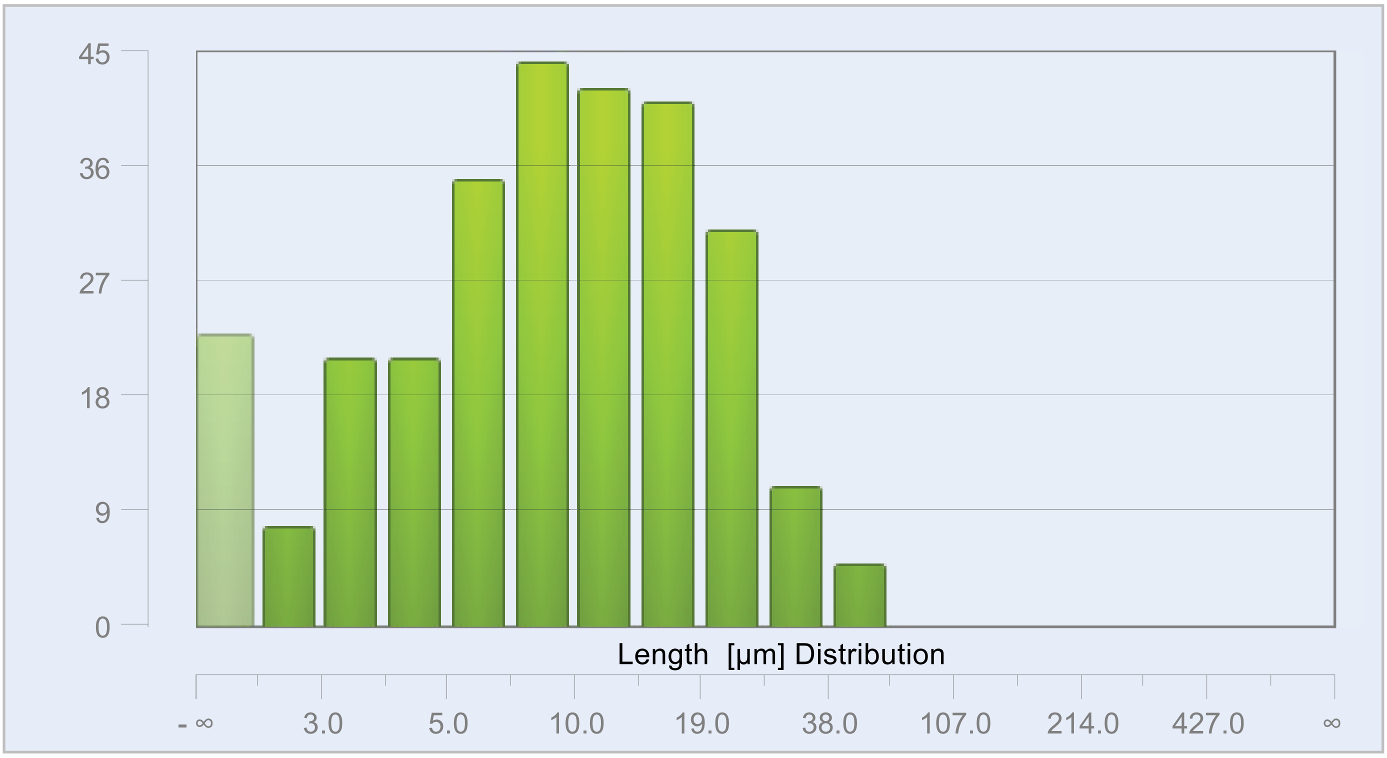

Start	End	Absolute Frequency	Absolute Frequency (accumulated)	Relative Frequency [%]	Relative Frequency (accumulated) [%]
	2.0 µm	23	23	8	8
2.0 µm	3.0 µm	8	31	3	11
3.0 µm	4.0 µm	21	52	7	18
4.0 µm	5.0 µm	21	73	7	26
5.0 µm	7.0 µm	35	108	12	38
7.0 µm	10.0 µm	44	152	16	54
10.0 µm	13.0 µm	42	194	15	69
13.0 µm	19.0 µm	41	235	15	83
19.0 µm	27.0 µm	31	266	11	94
27.0 µm	38.0 µm	11	277	4	98
38.0 µm	75.0 µm	5	282	2	100
75.0 µm	107.0 µm	0	282	0	100
107.0 µm	151.0 µm	0	282	0	100
151.0 µm	214.0 µm	0	282	0	100
214.0 µm	302.0 µm	0	282	0	100
302.0 µm	427.0 µm	0	282	0	100
427.0 µm	600.0 µm	0	282	0	100
600.0 µm		0	282	0	100
