# Supplementary material for: Benchmark dataset of the effect of grain size on strength in the single-phase FCC CrCoNi medium entropy alloy
Source: Data Brief. 2019 Oct 1;27:104592. doi: 10.1016/j.dib.2019.104592 (PMC6812030; doi:10.1016/j.dib.2019.104592)
Supplement: Multimedia component 1 [file mmc1.zip › CrCoNi_1173K_15min/CrCoNi_1173K_15min_c=2.5μm.pdf]

# Analysebericht

Nov 13, 2017 1:08:23 PM

powered by imagic.ch

## 1. cumulative Result 1

| | |
|---|---|
| Number of images | 4 |
| Grain size (ASTM) | 13.9 |
| Grain size (G643) | 13.8 |
| Grain stretching | 83.2 % |
| Mean chord length | 2.6 µm |

## 2. Single Result 1 (CrCoNi - ASTM E 112_CrCoNi_homogenized_8.1mmSW_900°C_15min_00166)

| | |
|---|---|
| Mean chord length | 2.6 µm |
| Grain size (ASTM) | 13.8 |
| Grain size (G643) | 13.8 |
| Grain stretching | 81.4 % |

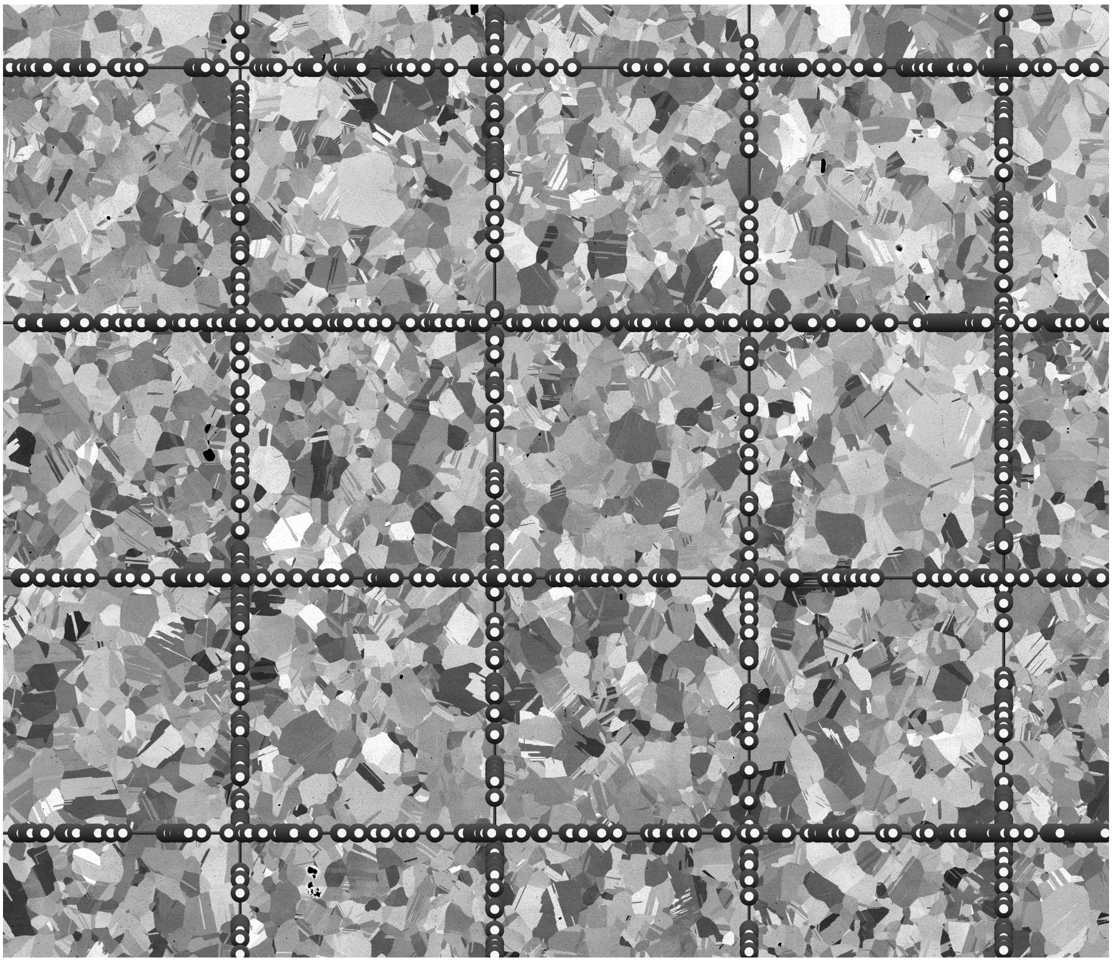

### 2.1. Statistical Analysis

| Statistical Data | Length |
|---|---|
| Object Count | 895 |
| Minimum | 0.2 µm |
| Maximum | 16.3 µm |
| Average | 2.6 µm |
| Standard deviation | 2.6 µm |
| Skewness | 0.0 |
| Standard deviation (n-1) | 2.6 µm |
| Variance | 6.6 µm² |
| Variance (n-1) | 6.6 µm² |
| Sum | 2'365.6 µm |

| Statistical Data | Length |
|---|---|
| Sum of squares | 12'193.5 µm² |
| Sum of cubes | 92'633.3 µm³ |

### 2.1.1. Chord Lenght Distribution

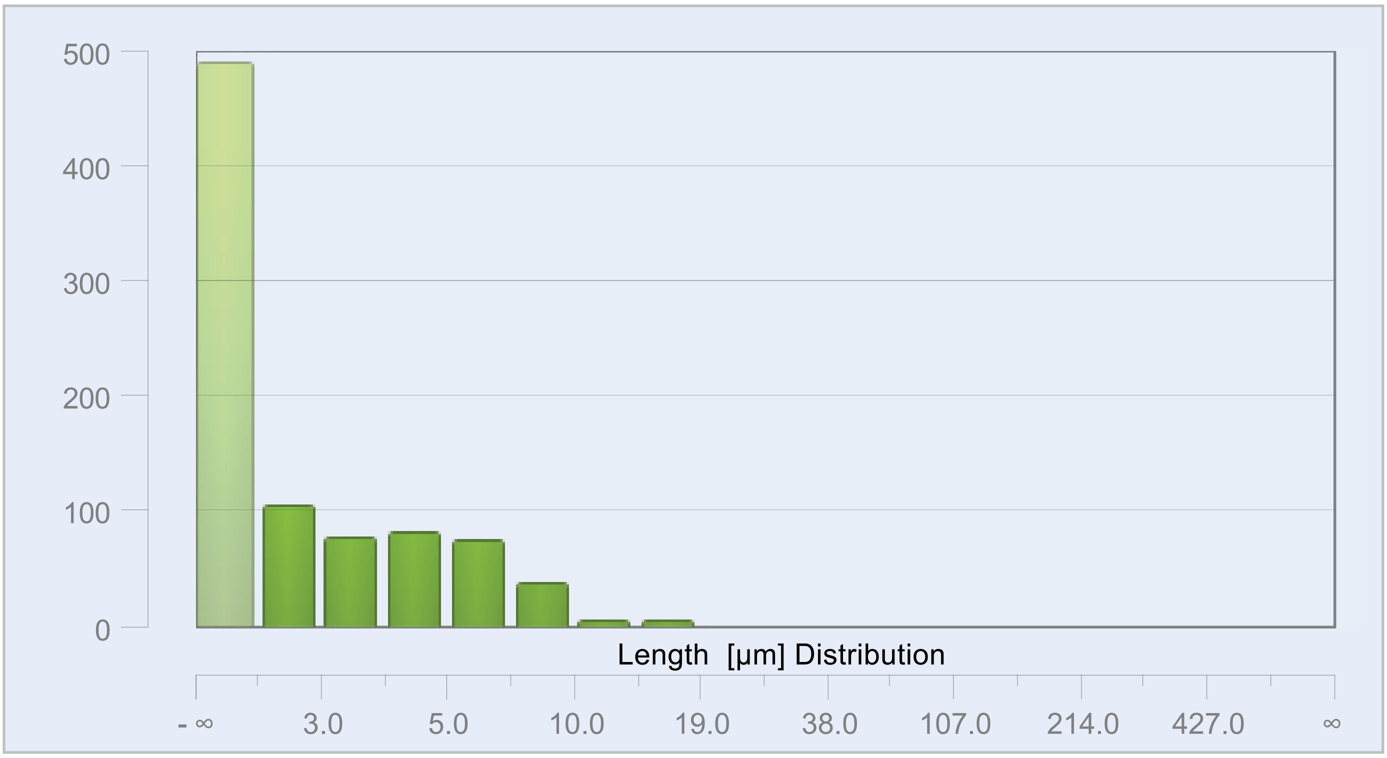


| Start | End | Absolute Frequency | Absolute Frequency (accumulated) | Relative Frequency [%] | Relative Frequency (accumulated) [%] |
|---|---|---|---|---|---|
| | 2.0 µm | 490 | 490 | 55 | 55 |
| 2.0 µm | 3.0 µm | 106 | 596 | 12 | 67 |
| 3.0 µm | 4.0 µm | 80 | 676 | 9 | 76 |
| 4.0 µm | 5.0 µm | 85 | 761 | 9 | 85 |
| 5.0 µm | 7.0 µm | 77 | 838 | 9 | 94 |
| 7.0 µm | 10.0 µm | 41 | 879 | 5 | 98 |
| 10.0 µm | 13.0 µm | 8 | 887 | 1 | 99 |
| 13.0 µm | 19.0 µm | 8 | 895 | 1 | 100 |
| 19.0 µm | 27.0 µm | 0 | 895 | 0 | 100 |
| 27.0 µm | 38.0 µm | 0 | 895 | 0 | 100 |
| 38.0 µm | 75.0 µm | 0 | 895 | 0 | 100 |
| 75.0 µm | 107.0 µm | 0 | 895 | 0 | 100 |
| 107.0 µm | 151.0 µm | 0 | 895 | 0 | 100 |
| 151.0 µm | 214.0 µm | 0 | 895 | 0 | 100 |
| 214.0 µm | 302.0 µm | 0 | 895 | 0 | 100 |
| 302.0 µm | 427.0 µm | 0 | 895 | 0 | 100 |
| 427.0 µm | 600.0 µm | 0 | 895 | 0 | 100 |
| 600.0 µm | | 0 | 895 | 0 | 100 |

## 3. Single Result 2 (CrCoNi - ASTM E 112_CrCoNi_homogenized_8.1mmSW_900°C_15min_00167)

| | |
|---|---|
| Mean chord length | 2.6 µm |
| Grain size (ASTM) | 13.9 |
| Grain size (G643) | 13.8 |
| Grain stretching | 85.2 % |

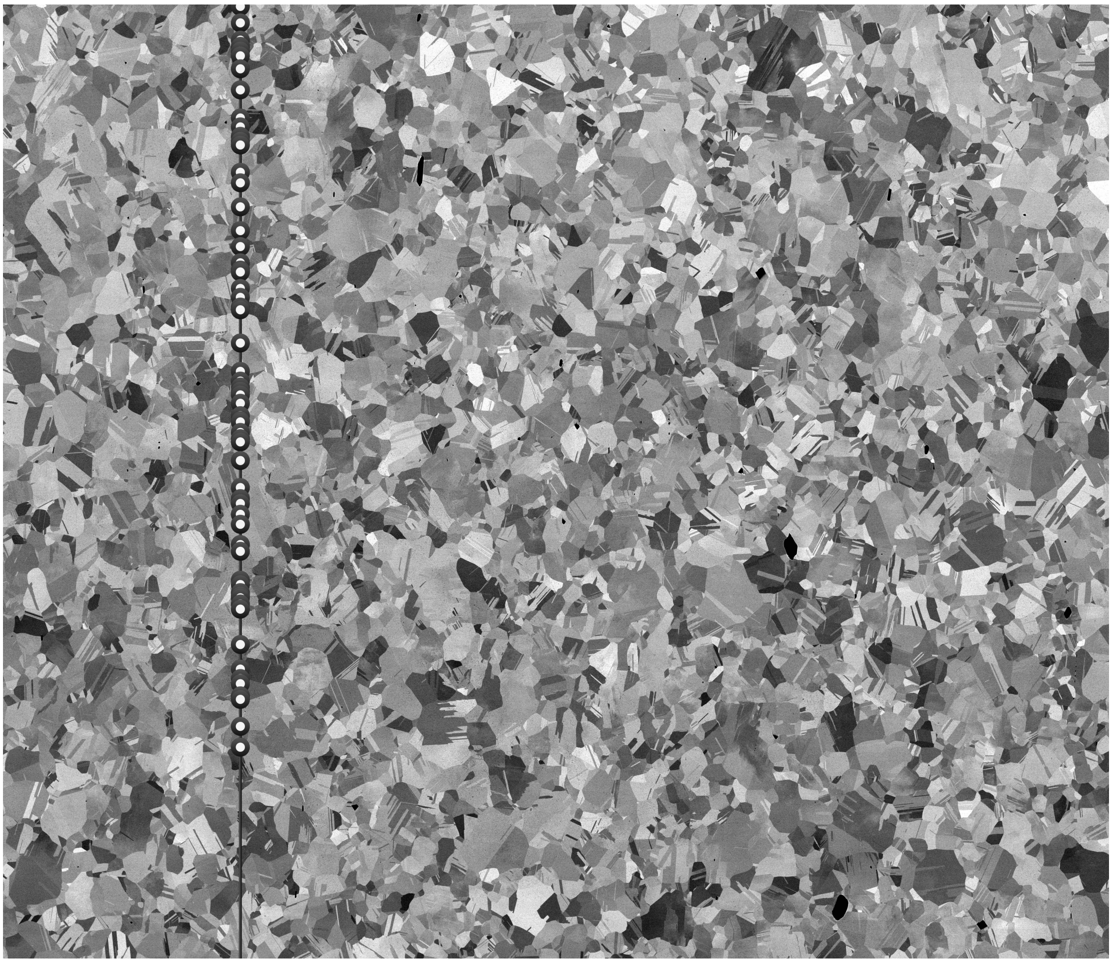

### 3.1. Statistical Analysis

| Statistical Data | Length |
|---|---|
| Object Count | 911 |
| Minimum | 0.2 µm |
| Maximum | 15.5 µm |
| Average | 2.6 µm |
| Standard deviation | 2.5 µm |
| Skewness | 0.0 |
| Standard deviation (n-1) | 2.5 µm |
| Variance | 6.1 µm² |
| Variance (n-1) | 6.1 µm² |
| Sum | 2'365.0 µm |
| Sum of squares | 11'734.5 µm² |
| Sum of cubes | 83'787.2 µm³ |

#### 3.1.1. Chord Lenght Distribution

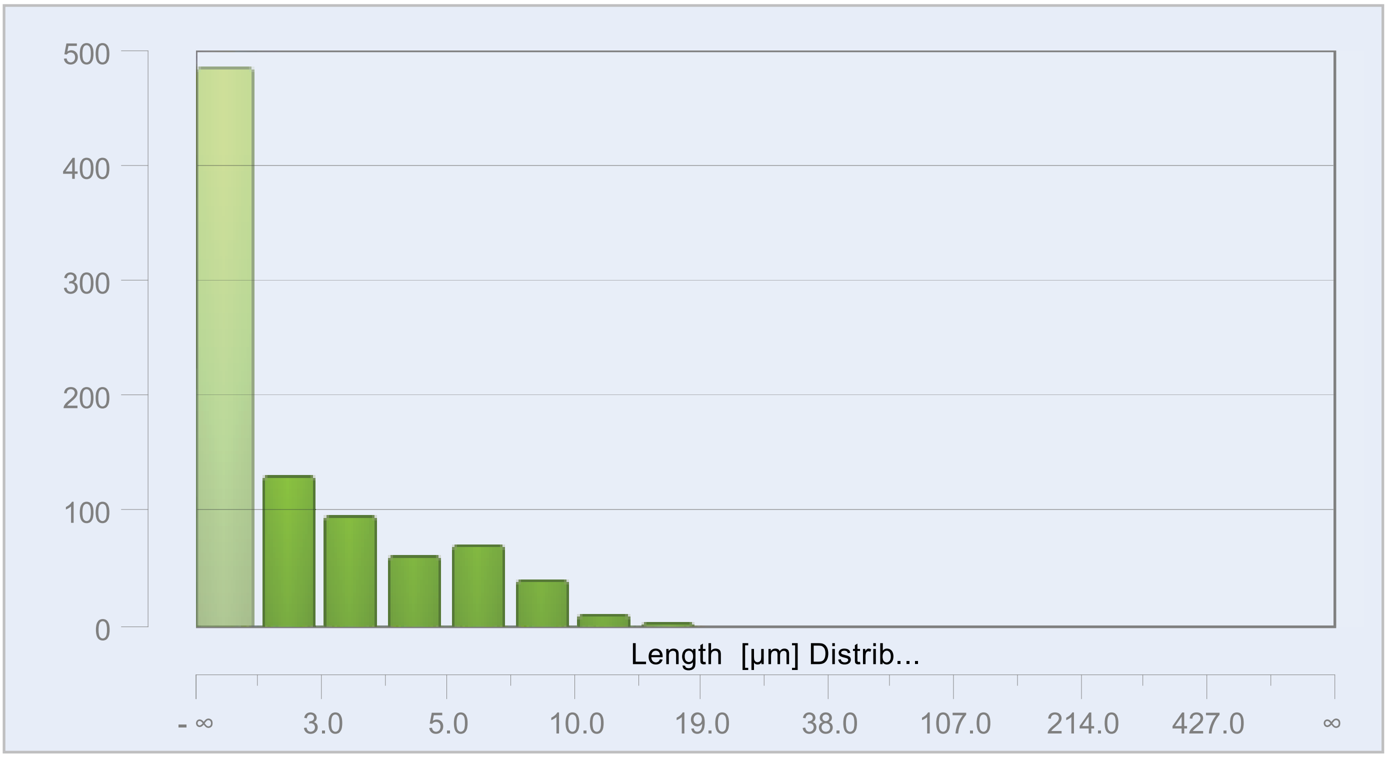


| Start | End | Absolute Frequency | Absolute Frequency (accumulated) | Relative Frequency [%] | Relative Frequency (accumulated) [%] |
|---|---|---|---|---|---|
| | 2.0 µm | 485 | 485 | 53 | 53 |
| 2.0 µm | 3.0 µm | 132 | 617 | 14 | 68 |
| 3.0 µm | 4.0 µm | 99 | 716 | 11 | 79 |
| 4.0 µm | 5.0 µm | 63 | 779 | 7 | 86 |
| 5.0 µm | 7.0 µm | 72 | 851 | 8 | 93 |
| 7.0 µm | 10.0 µm | 43 | 894 | 5 | 98 |
| 10.0 µm | 13.0 µm | 12 | 906 | 1 | 99 |
| 13.0 µm | 19.0 µm | 5 | 911 | 1 | 100 |
| 19.0 µm | 27.0 µm | 0 | 911 | 0 | 100 |
| 27.0 µm | 38.0 µm | 0 | 911 | 0 | 100 |
| 38.0 µm | 75.0 µm | 0 | 911 | 0 | 100 |
| 75.0 µm | 107.0 µm | 0 | 911 | 0 | 100 |
| 107.0 µm | 151.0 µm | 0 | 911 | 0 | 100 |
| 151.0 µm | 214.0 µm | 0 | 911 | 0 | 100 |
| 214.0 µm | 302.0 µm | 0 | 911 | 0 | 100 |
| 302.0 µm | 427.0 µm | 0 | 911 | 0 | 100 |
| 427.0 µm | 600.0 µm | 0 | 911 | 0 | 100 |
| 600.0 µm | | 0 | 911 | 0 | 100 |

4. Single Result 3 (CrCoNi - ASTM E 112_CrCoNi_homogenized_8.1mmSW_900°C_15min_00168)

| | |
|---|---|
| Mean chord length | 2.4 µm |
| Grain size (ASTM) | 14.1 |
| Grain size (G643) | 14 |
| Grain stretching | 79.9 % |

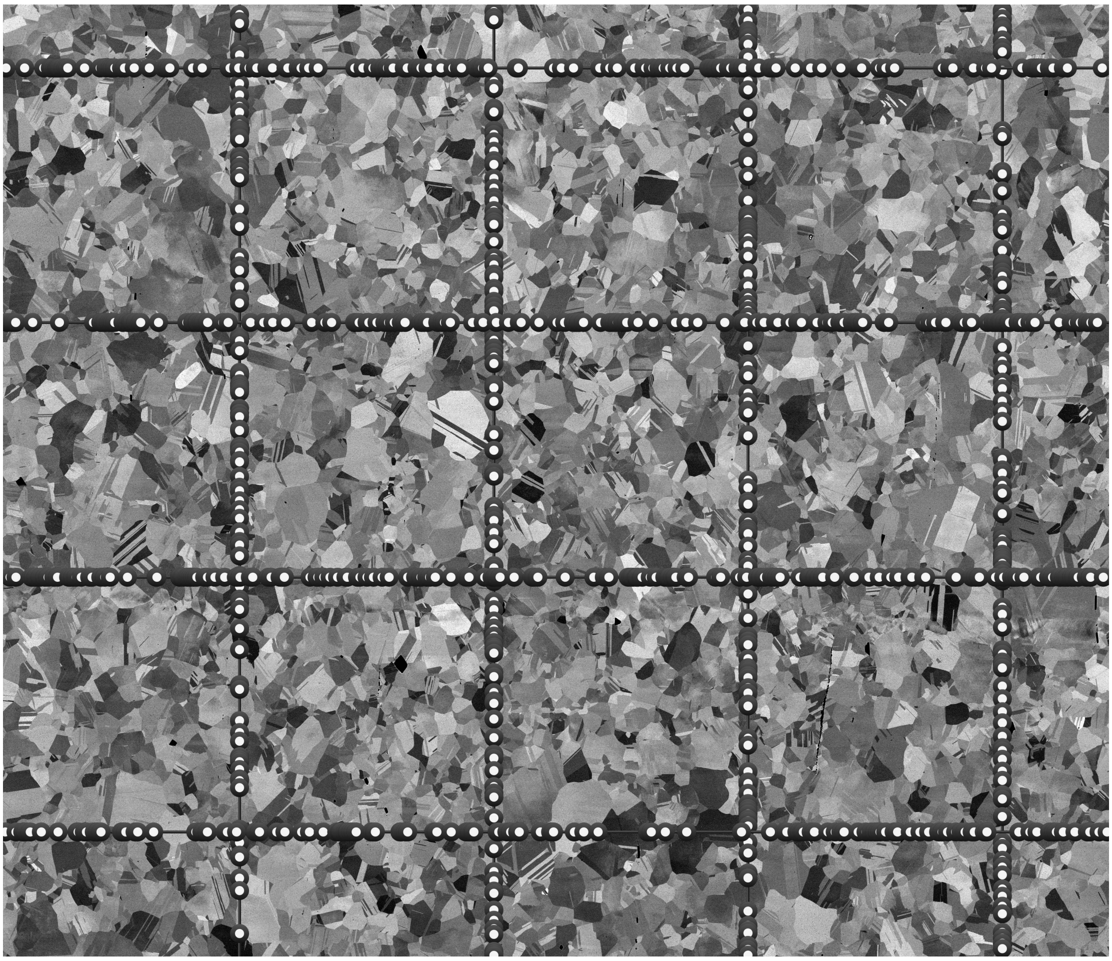

## 4.1. Statistical Analysis

| Statistical Data | Length |
| --- | --- |
| Object Count | 972 |
| Minimum | 0.2 µm |
| Maximum | 17.7 µm |
| Average | 2.4 µm |
| Standard deviation | 2.4 µm |
| Skewness | 0.0 |
| Standard deviation (n-1) | 2.4 µm |
| Variance | 5.7 µm² |
| Variance (n-1) | 5.7 µm² |
| Sum | 2'362.2 µm |
| Sum of squares | 11'277.7 µm² |
| Sum of cubes | 81'906.0 µm³ |

### 4.1.1. Chord Lenght Distribution

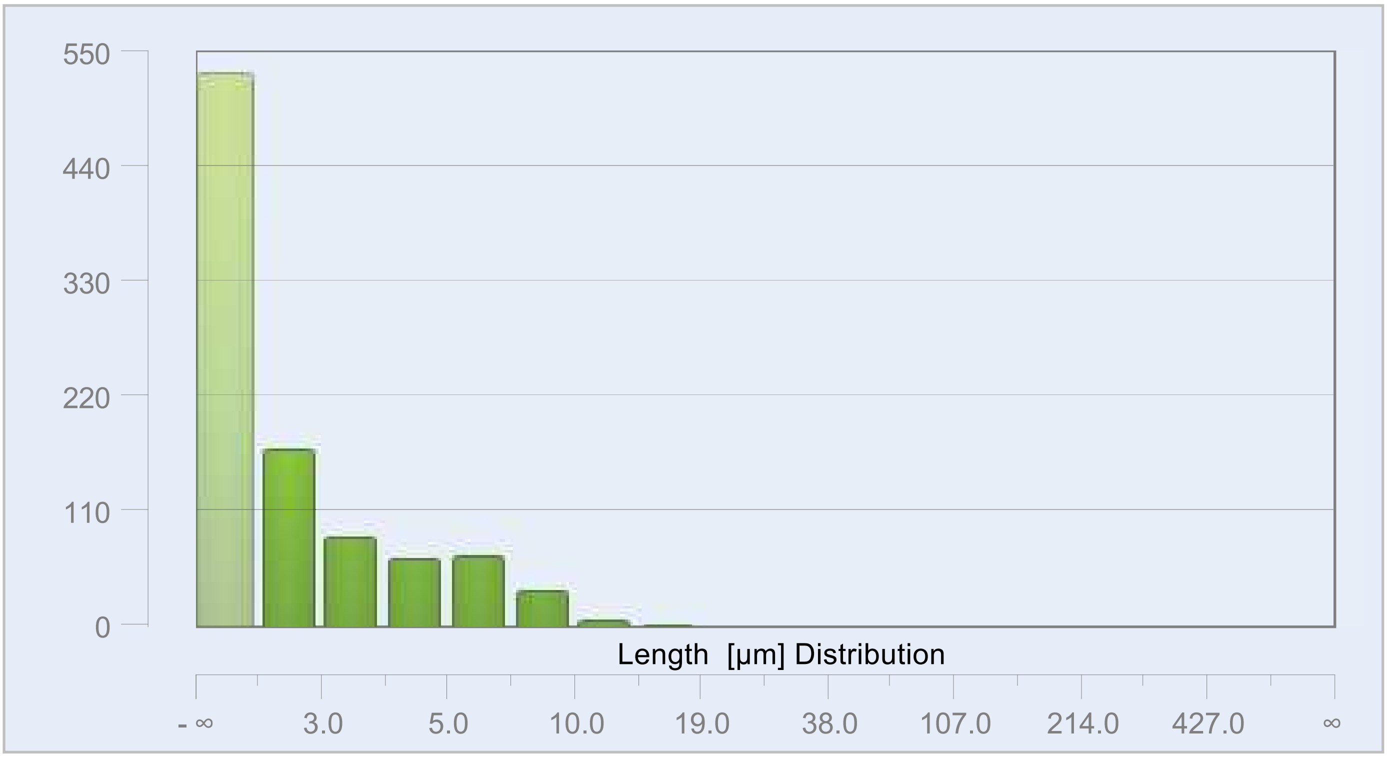


| Start | End | Absolute Frequency | Absolute Frequency (accumulated) | Relative Frequency [%] | Relative Frequency (accumulated) [%] |
|---|---|---|---|---|---|
| | 2.0 µm | 528 | 528 | 54 | 54 |
| 2.0 µm | 3.0 µm | 170 | 698 | 17 | 72 |
| 3.0 µm | 4.0 µm | 88 | 786 | 9 | 81 |
| 4.0 µm | 5.0 µm | 66 | 852 | 7 | 88 |
| 5.0 µm | 7.0 µm | 69 | 921 | 7 | 95 |
| 7.0 µm | 10.0 µm | 37 | 958 | 4 | 99 |
| 10.0 µm | 13.0 µm | 9 | 967 | 1 | 99 |
| 13.0 µm | 19.0 µm | 5 | 972 | 1 | 100 |
| 19.0 µm | 27.0 µm | 0 | 972 | 0 | 100 |
| 27.0 µm | 38.0 µm | 0 | 972 | 0 | 100 |
| 38.0 µm | 75.0 µm | 0 | 972 | 0 | 100 |
| 75.0 µm | 107.0 µm | 0 | 972 | 0 | 100 |
| 107.0 µm | 151.0 µm | 0 | 972 | 0 | 100 |
| 151.0 µm | 214.0 µm | 0 | 972 | 0 | 100 |
| 214.0 µm | 302.0 µm | 0 | 972 | 0 | 100 |
| 302.0 µm | 427.0 µm | 0 | 972 | 0 | 100 |
| 427.0 µm | 600.0 µm | 0 | 972 | 0 | 100 |
| 600.0 µm | | 0 | 972 | 0 | 100 |

## 5. Single Result 4 (CrCoNi - ASTM E 112_CrCoNi_homogenized_8.1mmSW_900°C_15min_00169)

| | |
|---|---|
| Mean chord length | 2.8 µm |
| Grain size (ASTM) | 13.7 |
| Grain size (G643) | 13.7 |
| Grain stretching | 86.6 % |

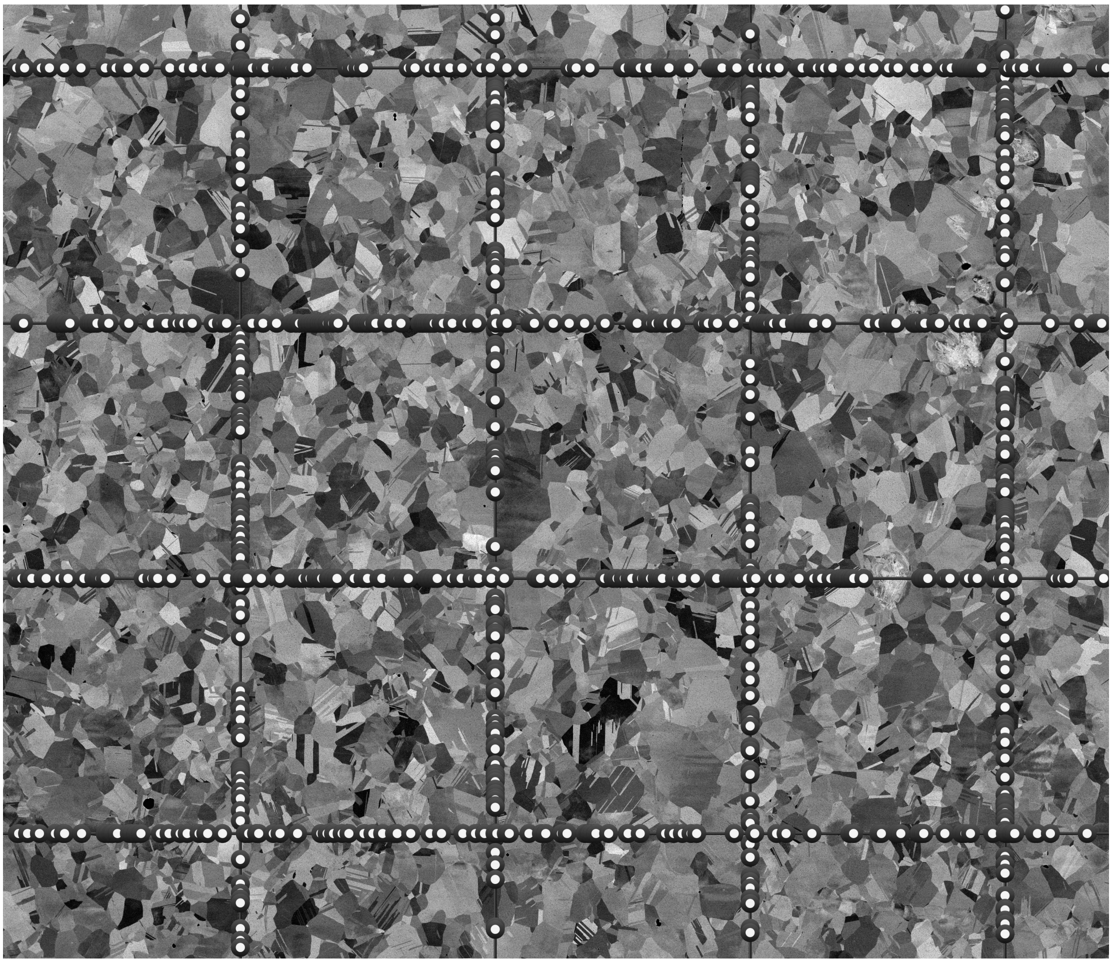

### 5.1. Statistical Analysis

| Statistical Data | Length |
|---|---|
| Object Count | 858 |
| Minimum | 0.2 µm |
| Maximum | 16.3 µm |
| Average | 2.8 µm |
| Standard deviation | 2.6 µm |
| Skewness | 0.0 |
| Standard deviation (n-1) | 2.6 µm |
| Variance | 6.8 µm² |
| Variance (n-1) | 6.8 µm² |
| Sum | 2'365.3 µm |
| Sum of squares | 12'345.4 µm² |
| Sum of cubes | 93'402.2 µm³ |

#### 5.1.1. Chord Lenght Distribution

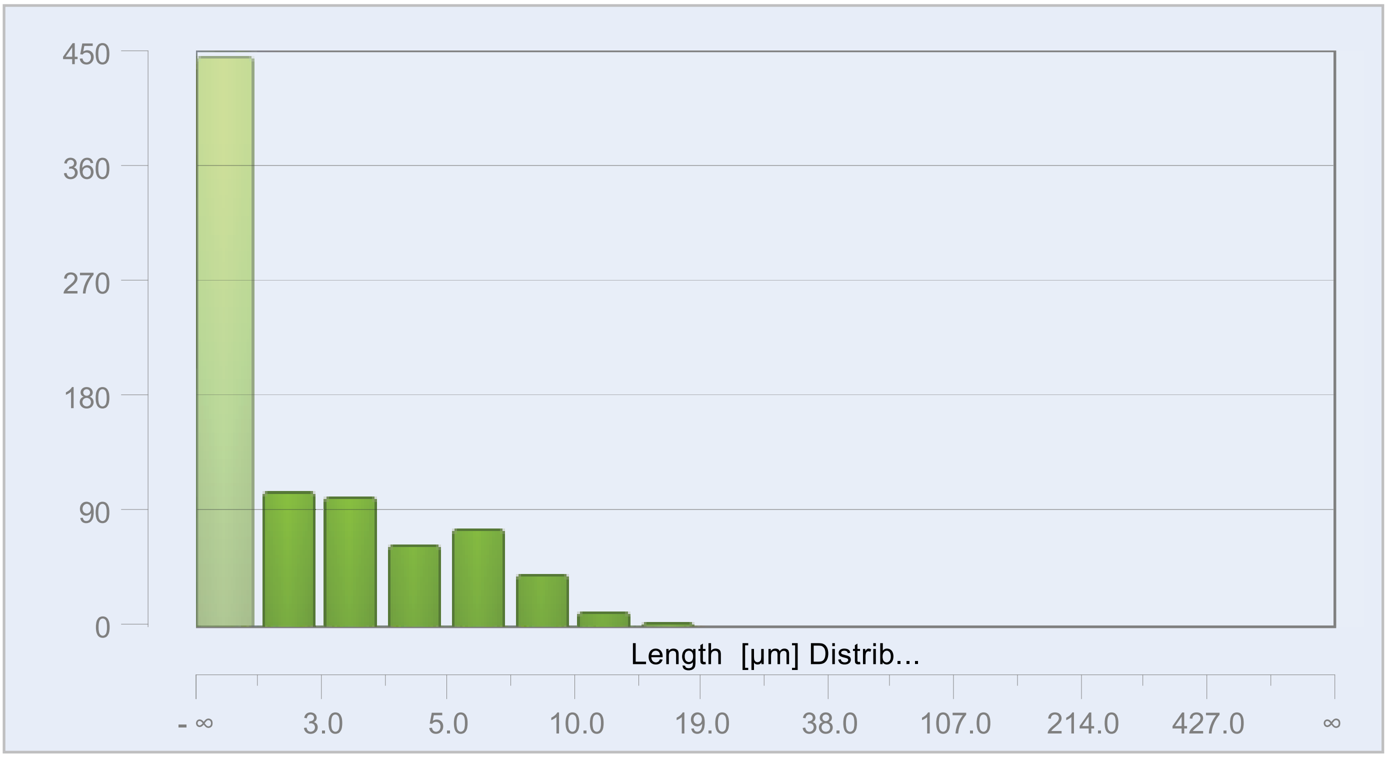


| Start | End | Absolute Frequency | Absolute Frequency (accumulated) | Relative Frequency [%] | Relative Frequency (accumulated) [%] |
|---|---|---|---|---|---|
| | 2.0 μm | 445 | 445 | 52 | 52 |
| 2.0 μm | 3.0 μm | 107 | 552 | 12 | 64 |
| 3.0 μm | 4.0 μm | 102 | 654 | 12 | 76 |
| 4.0 μm | 5.0 μm | 66 | 720 | 8 | 84 |
| 5.0 μm | 7.0 μm | 77 | 797 | 9 | 93 |
| 7.0 μm | 10.0 μm | 42 | 839 | 5 | 98 |
| 10.0 μm | 13.0 μm | 13 | 852 | 2 | 99 |
| 13.0 μm | 19.0 μm | 6 | 858 | 1 | 100 |
| 19.0 μm | 27.0 μm | 0 | 858 | 0 | 100 |
| 27.0 μm | 38.0 μm | 0 | 858 | 0 | 100 |
| 38.0 μm | 75.0 μm | 0 | 858 | 0 | 100 |
| 75.0 μm | 107.0 μm | 0 | 858 | 0 | 100 |
| 107.0 μm | 151.0 μm | 0 | 858 | 0 | 100 |
| 151.0 μm | 214.0 μm | 0 | 858 | 0 | 100 |
| 214.0 μm | 302.0 μm | 0 | 858 | 0 | 100 |
| 302.0 μm | 427.0 μm | 0 | 858 | 0 | 100 |
| 427.0 μm | 600.0 μm | 0 | 858 | 0 | 100 |
| 600.0 μm | | 0 | 858 | 0 | 100 |
