# Supplementary material for: Benchmark dataset of the effect of grain size on strength in the single-phase FCC CrCoNi medium entropy alloy
Source: Data Brief. 2019 Oct 1;27:104592. doi: 10.1016/j.dib.2019.104592 (PMC6812030; doi:10.1016/j.dib.2019.104592)
Supplement: Multimedia component 1 [file mmc1.zip › CrCoNi_1173K_15min/CrCoNi_1173K_15min_d=5.7μm.pdf]

Analysebericht

Aug 24, 2017 11:40:47 AM

powered by imagic.ch

1. cumulative Result 1

Number of images	4
Grain size (ASTM)	11.6
Grain size (G643)	11.6
Grain stretching	100 %
Mean chord length	5.7 µm

2. Single Result 1 (CrCoNi Twins grain size_900C 15min_00131)

Mean chord length	5.4 µm
Grain size (ASTM)	11.8
Grain size (G643)	11.7
Grain stretching	96.6 %

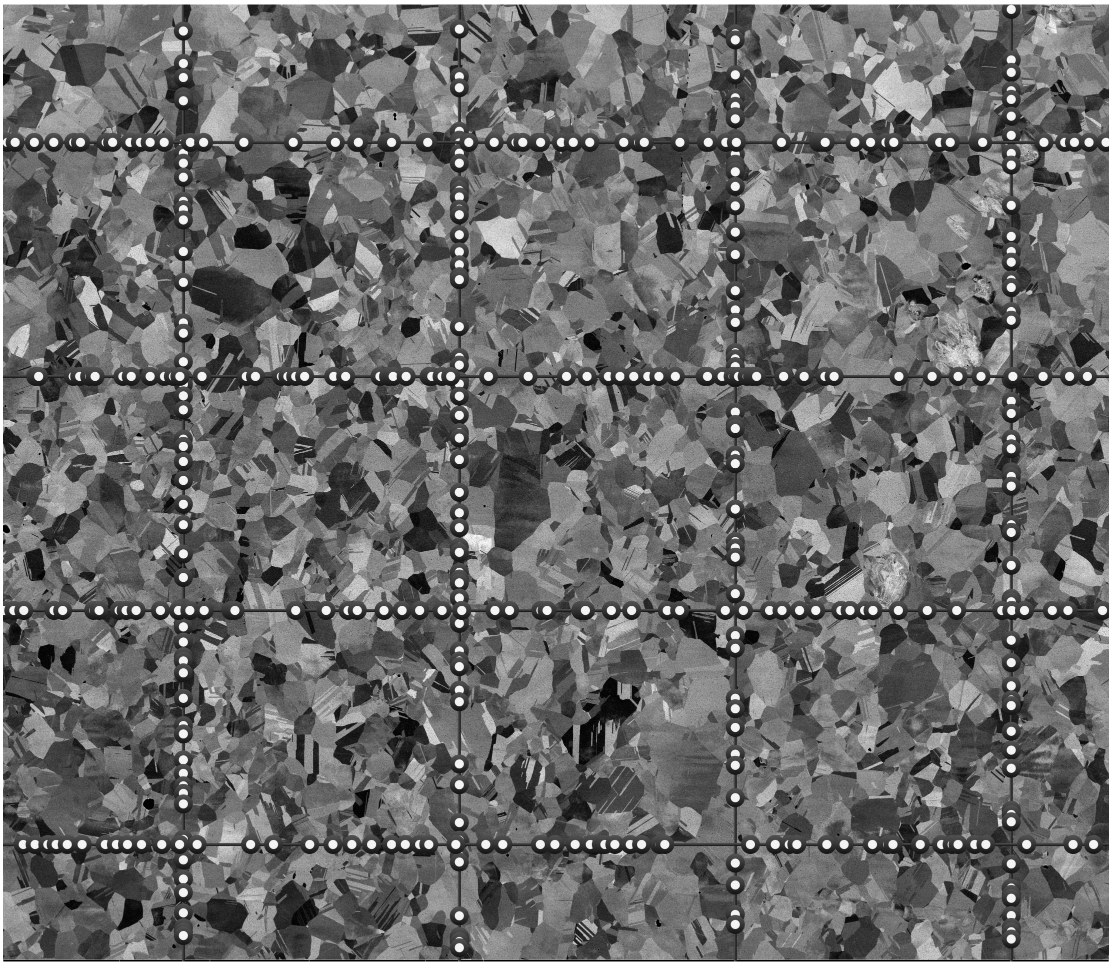

2.1. Statistical Analysis

Statistical Data	Length
Object Count	436
Minimum	0.2 µm
Maximum	24.6 µm
Average	5.4 µm
Standard deviation	3.7 µm
Skewness	0.0
Standard deviation (n-1)	3.7 µm
Variance	13.5 µm²
Variance (n-1)	13.5 µm²
Sum	2'368.1 µm

Statistical Data	Length
Sum of squares	18'755.3 µm²
Sum of cubes	193'108.7 µm³

2.1.1. Chord Lenght Distribution

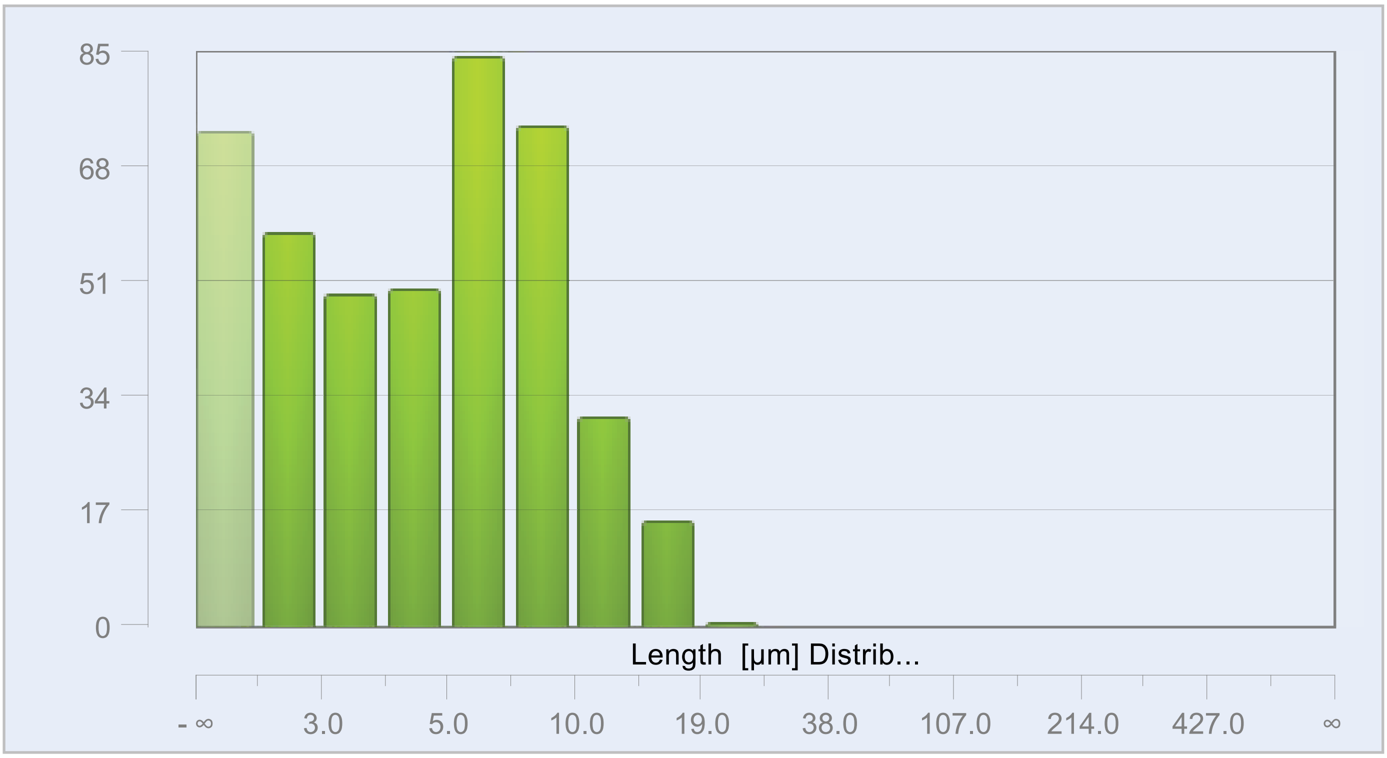

Start	End	Absolute Frequency	Absolute Frequency (accumulated)	Relative Frequency [%]	Relative Frequency (accumulated) [%]
	2.0 µm	73	73	17	17
2.0 µm	3.0 µm	58	131	13	30
3.0 µm	4.0 µm	49	180	11	41
4.0 µm	5.0 µm	50	230	11	53
5.0 µm	7.0 µm	84	314	19	72
7.0 µm	10.0 µm	74	388	17	89
10.0 µm	13.0 µm	31	419	7	96
13.0 µm	19.0 µm	16	435	4	100
19.0 µm	27.0 µm	1	436	0	100
27.0 µm	38.0 µm	0	436	0	100
38.0 µm	75.0 µm	0	436	0	100
75.0 µm	107.0 µm	0	436	0	100
107.0 µm	151.0 µm	0	436	0	100
151.0 µm	214.0 µm	0	436	0	100
214.0 µm	302.0 µm	0	436	0	100
302.0 µm	427.0 µm	0	436	0	100
427.0 µm	600.0 µm	0	436	0	100
600.0 µm		0	436	0	100

3. Single Result 2 (CrCoNi Twins grain size_900C 15min_00132)

Mean chord length	5.3 µm
Grain size (ASTM)	11.8
Grain size (G643)	11.8
Grain stretching	93.5 %

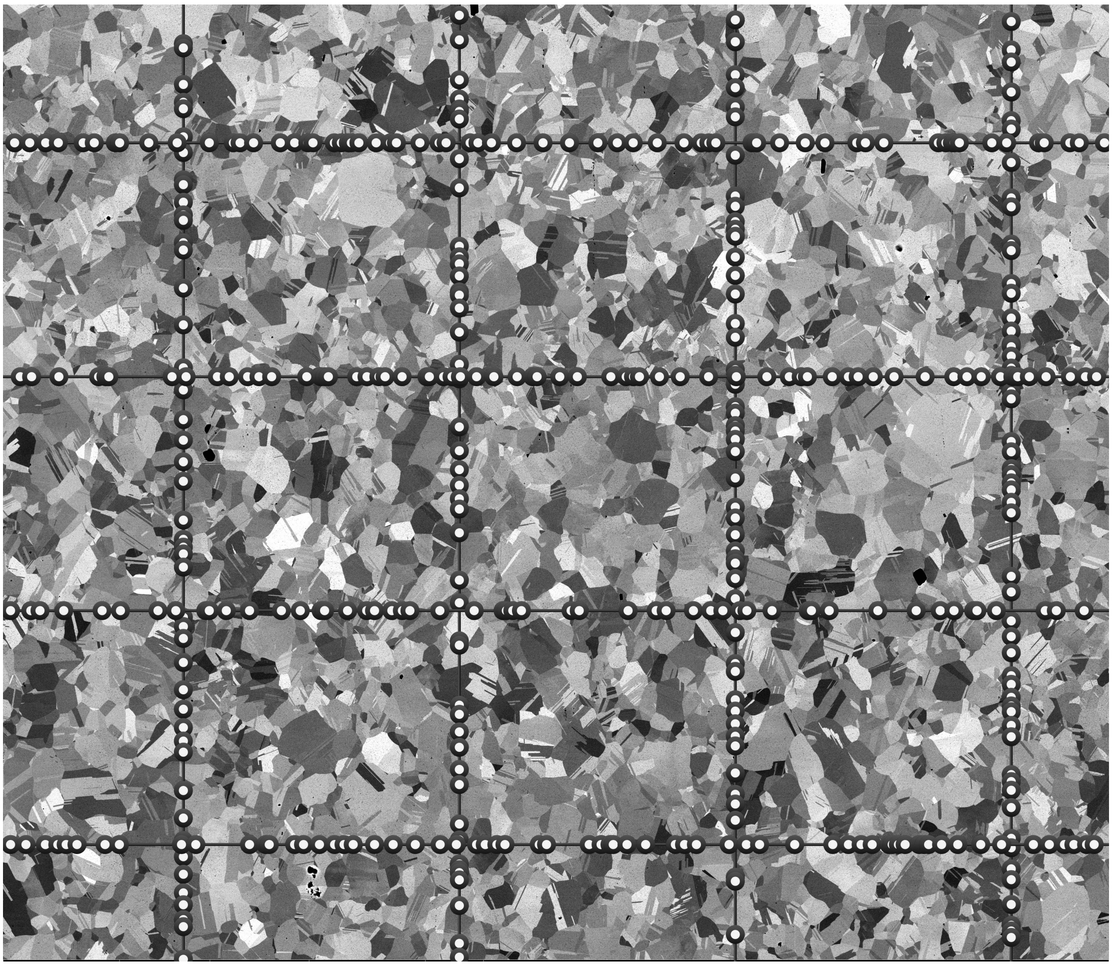

3.1. Statistical Analysis

Statistical Data	Length
Object Count	445
Minimum	0.5 µm
Maximum	18.2 µm
Average	5.3 µm
Standard deviation	3.4 µm
Skewness	0.0
Standard deviation (n-1)	3.4 µm
Variance	11.5 µm²
Variance (n-1)	11.6 µm²
Sum	2'368.1 µm
Sum of squares	17'735.4 µm²
Sum of cubes	169'298.2 µm³

3.1.1. Chord Lenght Distribution

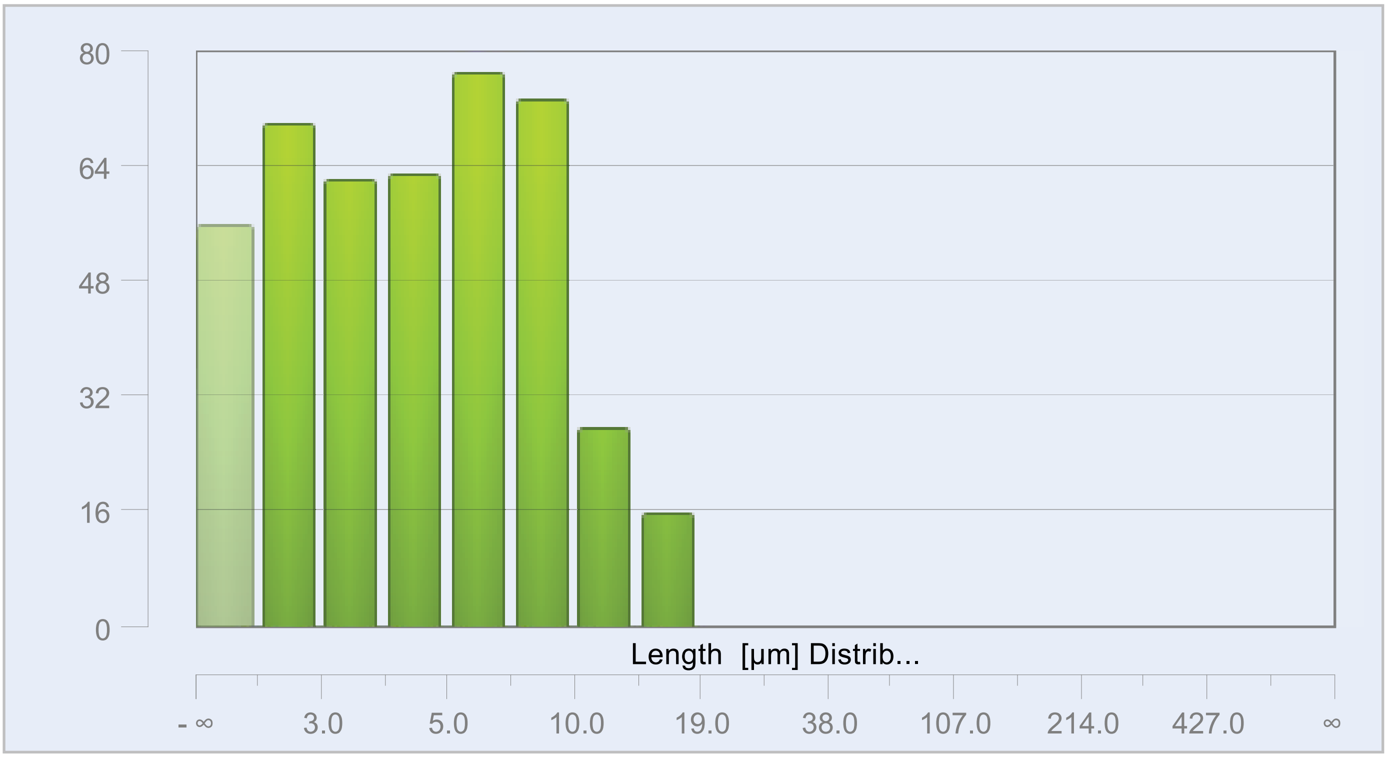

Start	End	Absolute Frequency	Absolute Frequency (accumulated)	Relative Frequency [%]	Relative Frequency (accumulated) [%]
	2.0 µm	56	56	13	13
2.0 µm	3.0 µm	70	126	16	28
3.0 µm	4.0 µm	62	188	14	42
4.0 µm	5.0 µm	63	251	14	56
5.0 µm	7.0 µm	77	328	17	74
7.0 µm	10.0 µm	73	401	16	90
10.0 µm	13.0 µm	28	429	6	96
13.0 µm	19.0 µm	16	445	4	100
19.0 µm	27.0 µm	0	445	0	100
27.0 µm	38.0 µm	0	445	0	100
38.0 µm	75.0 µm	0	445	0	100
75.0 µm	107.0 µm	0	445	0	100
107.0 µm	151.0 µm	0	445	0	100
151.0 µm	214.0 µm	0	445	0	100
214.0 µm	302.0 µm	0	445	0	100
302.0 µm	427.0 µm	0	445	0	100
427.0 µm	600.0 µm	0	445	0	100
600.0 µm		0	445	0	100

4. Single Result 3 (CrCoNi Twins grain size_900C 15min_00133)

Mean chord length	6.3 µm
Grain size (ASTM)	11.3
Grain size (G643)	11.3
Grain stretching	85.4 %

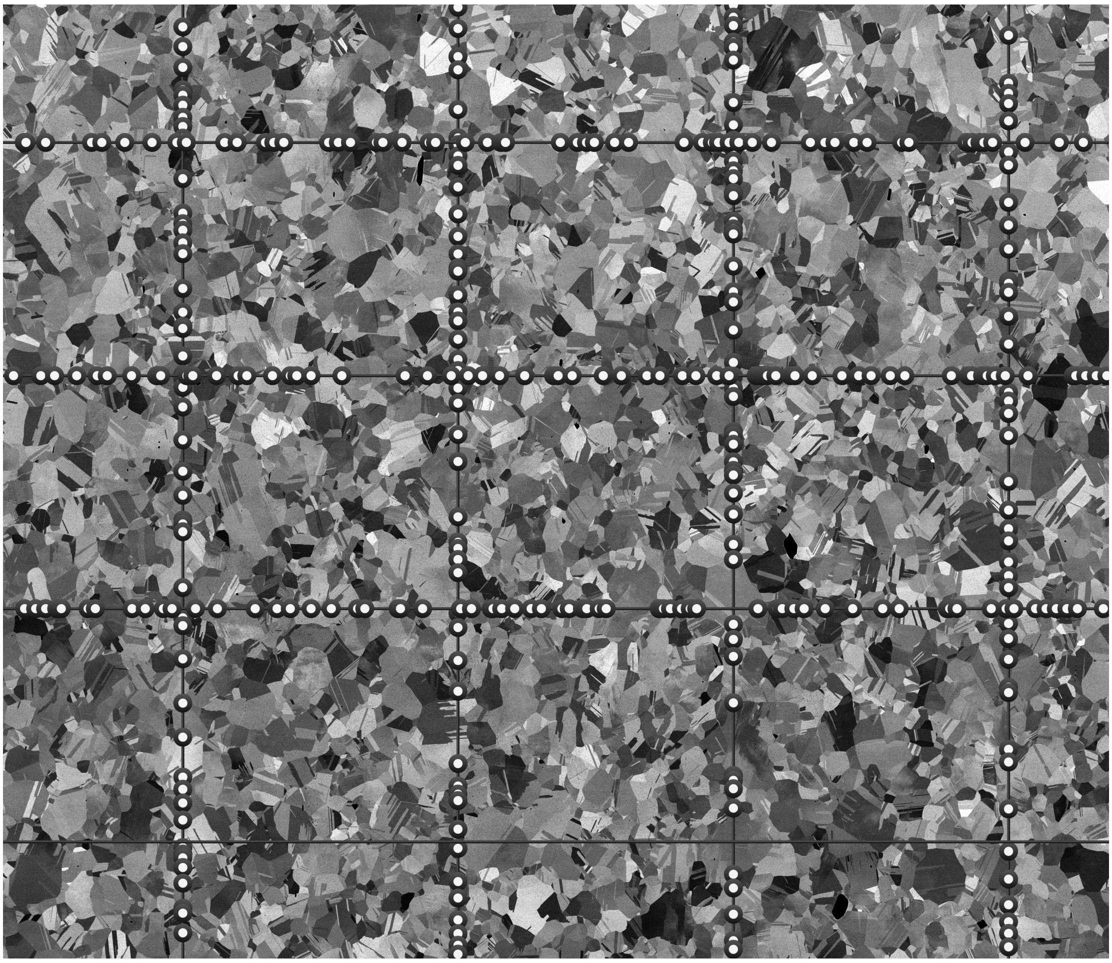

4.1. Statistical Analysis

Statistical Data	Length
Object Count	374
Minimum	0.2 µm
Maximum	317.7 µm
Average	6.3 µm
Standard deviation	16.6 µm
Skewness	0.0
Standard deviation (n-1)	16.6 µm
Variance	274.2 µm²
Variance (n-1)	274.9 µm²
Sum	2'367.1 µm
Sum of squares	117'523.5 µm²
Sum of cubes	32'240'226.3 µm³

4.1.1. Chord Lenght Distribution

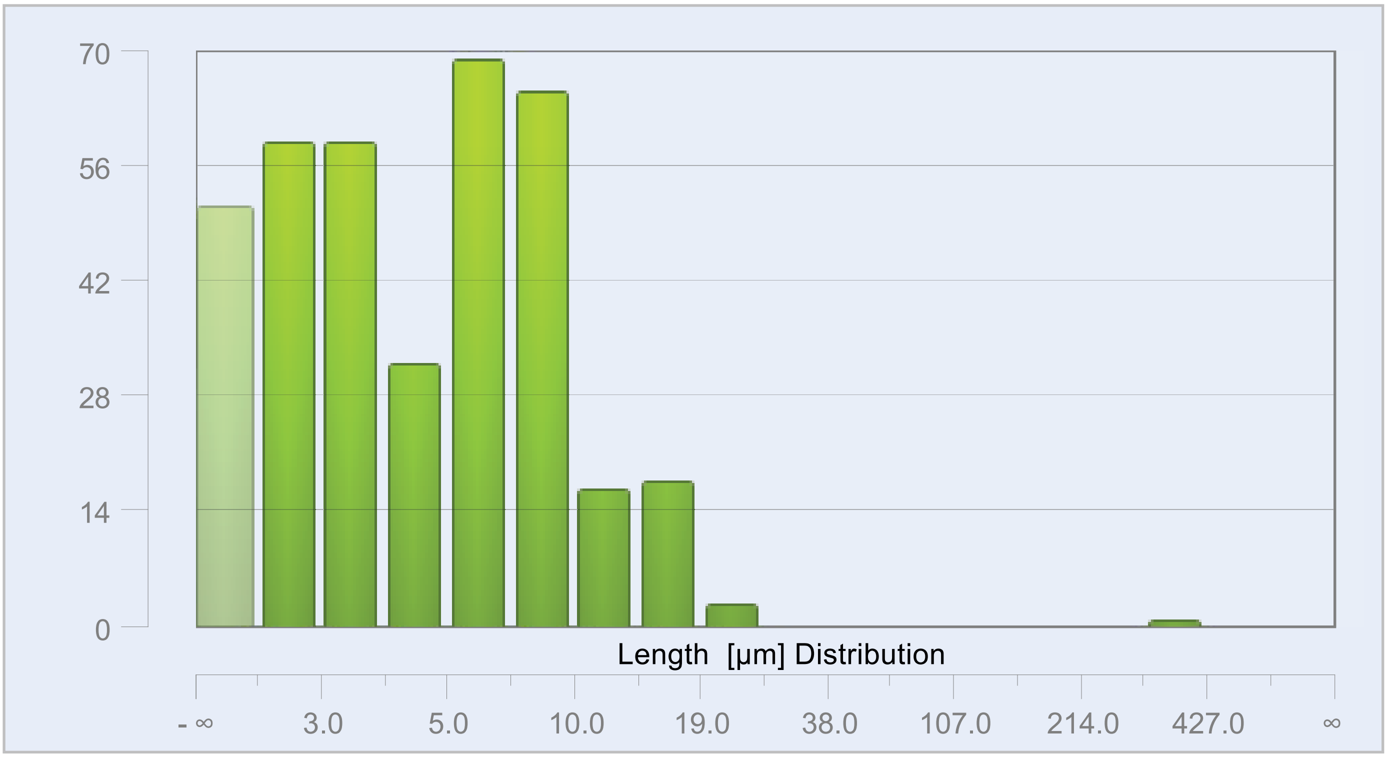

Start	End	Absolute Frequency	Absolute Frequency (accumulated)	Relative Frequency [%]	Relative Frequency (accumulated) [%]
	2.0 µm	51	51	14	14
2.0 µm	3.0 µm	59	110	16	29
3.0 µm	4.0 µm	59	169	16	45
4.0 µm	5.0 µm	32	201	9	54
5.0 µm	7.0 µm	69	270	18	72
7.0 µm	10.0 µm	65	335	17	90
10.0 µm	13.0 µm	17	352	5	94
13.0 µm	19.0 µm	18	370	5	99
19.0 µm	27.0 µm	3	373	1	100
27.0 µm	38.0 µm	0	373	0	100
38.0 µm	75.0 µm	0	373	0	100
75.0 µm	107.0 µm	0	373	0	100
107.0 µm	151.0 µm	0	373	0	100
151.0 µm	214.0 µm	0	373	0	100
214.0 µm	302.0 µm	0	373	0	100
302.0 µm	427.0 µm	1	374	0	100
427.0 µm	600.0 µm	0	374	0	100
600.0 µm		0	374	0	100

5. Single Result 4 (CrCoNi Twins grain size_900C 15min_00134)

Mean chord length	5.6 µm
Grain size (ASTM)	11.7
Grain size (G643)	11.6
Grain stretching	89.7 %

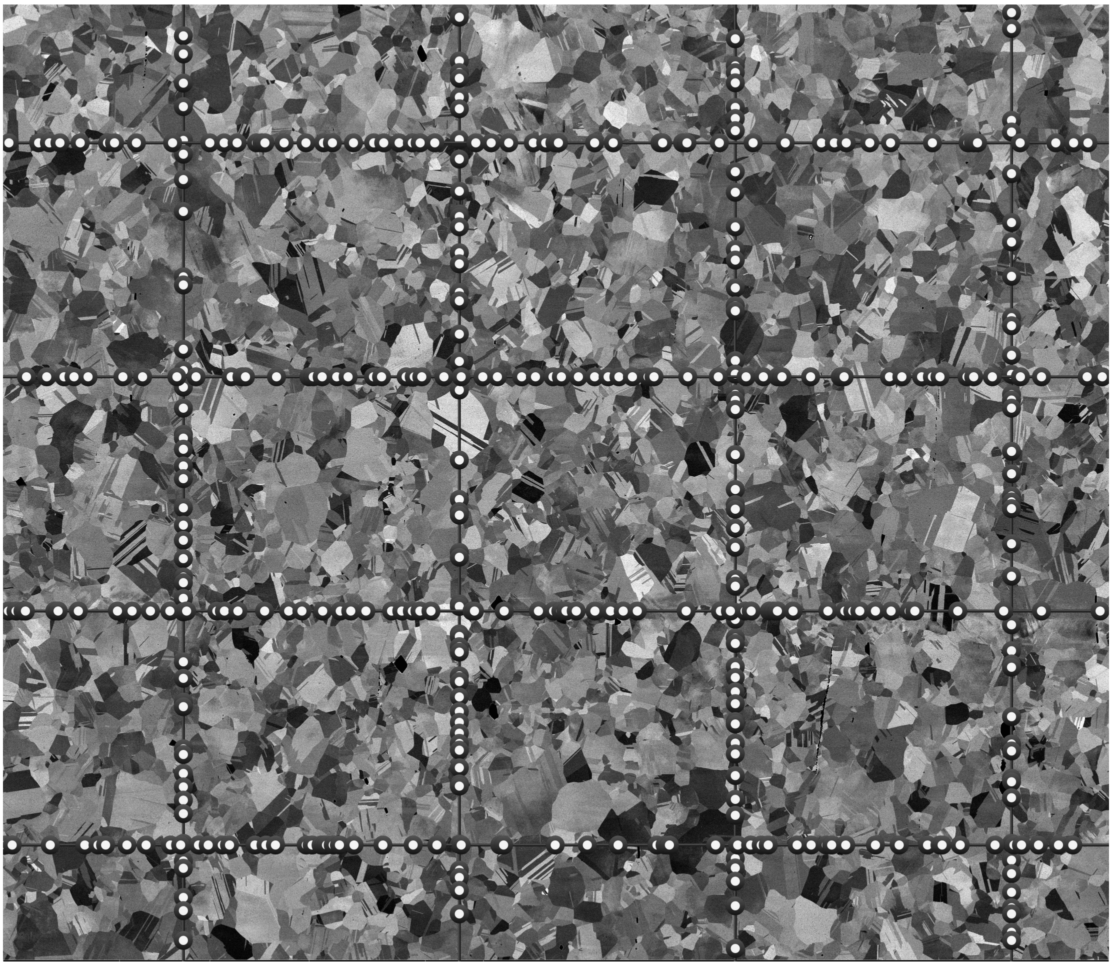

5.1. Statistical Analysis

Statistical Data	Length
Object Count	421
Minimum	0.6 µm
Maximum	26.4 µm
Average	5.6 µm
Standard deviation	3.7 µm
Skewness	0.0
Standard deviation (n-1)	3.7 µm
Variance	14.0 µm²
Variance (n-1)	14.0 µm²
Sum	2'368.4 µm
Sum of squares	19'222.0 µm²
Sum of cubes	202'227.8 µm³

5.1.1. Chord Lenght Distribution

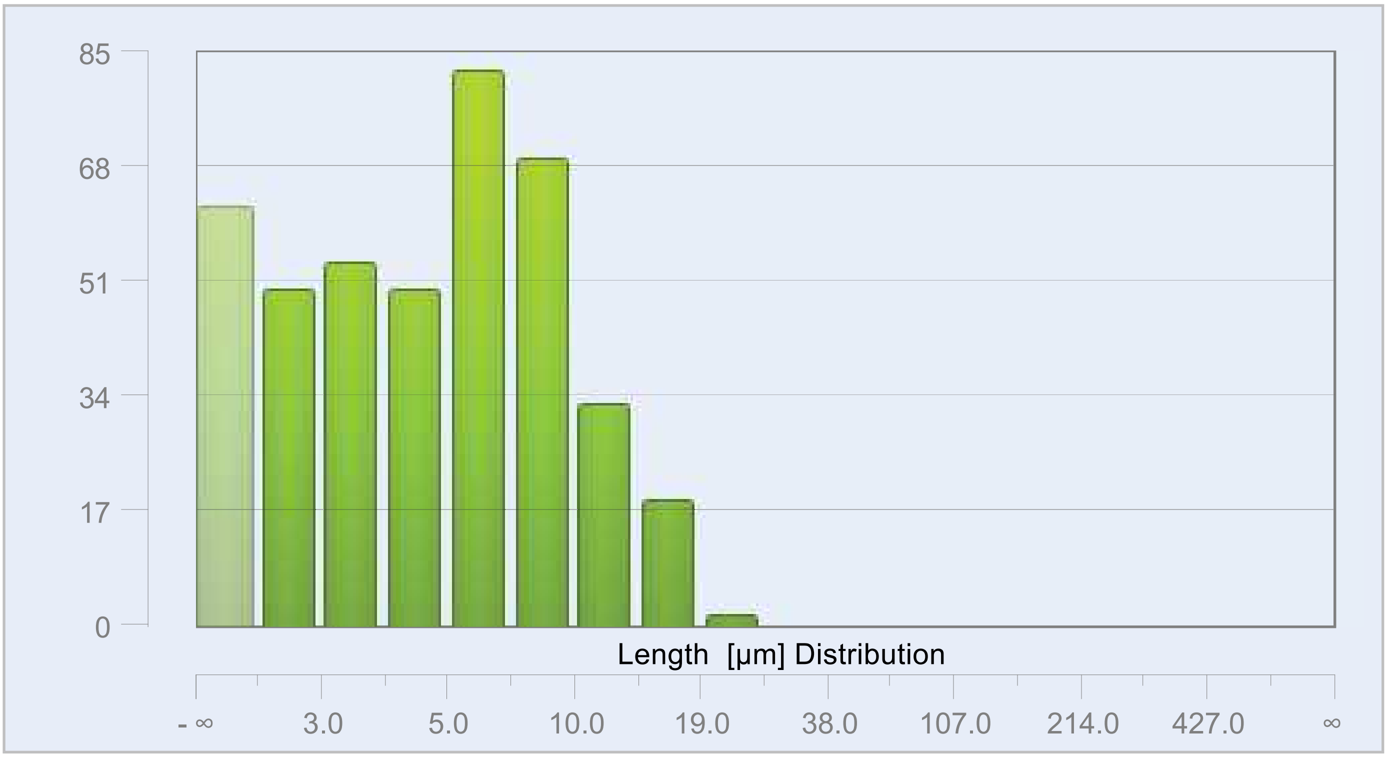

Start	End	Absolute Frequency	Absolute Frequency (accumulated)	Relative Frequency [%]	Relative Frequency (accumulated) [%]
	2.0 µm	62	62	15	15
2.0 µm	3.0 µm	50	112	12	27
3.0 µm	4.0 µm	54	166	13	39
4.0 µm	5.0 µm	50	216	12	51
5.0 µm	7.0 µm	82	298	19	71
7.0 µm	10.0 µm	69	367	16	87
10.0 µm	13.0 µm	33	400	8	95
13.0 µm	19.0 µm	19	419	5	100
19.0 µm	27.0 µm	2	421	0	100
27.0 µm	38.0 µm	0	421	0	100
38.0 µm	75.0 µm	0	421	0	100
75.0 µm	107.0 µm	0	421	0	100
107.0 µm	151.0 µm	0	421	0	100
151.0 µm	214.0 µm	0	421	0	100
214.0 µm	302.0 µm	0	421	0	100
302.0 µm	427.0 µm	0	421	0	100
427.0 µm	600.0 µm	0	421	0	100
600.0 µm		0	421	0	100
