# Supplementary material for: Benchmark dataset of the effect of grain size on strength in the single-phase FCC CrCoNi medium entropy alloy
Source: Data Brief. 2019 Oct 1;27:104592. doi: 10.1016/j.dib.2019.104592 (PMC6812030; doi:10.1016/j.dib.2019.104592)
Supplement: Multimedia component 1 [file mmc1.zip › CrCoNi_1173K_180min/CrCoNi_1173K_180min_c=6.9μm.pdf]

Analysebericht

03.11.2017 11:33:19

powered by imagic.ch

1. cumulative Result 1

Anzahl Bilder	4
Korngröße (ASTM)	11,1
Korngröße (G643)	11
Kornstreckung	94,3 %
Mittlere Sehnenlänge	6,9 µm

2. Single Result 1 (CrCoNi - ASTM E 112_CrCoNi_homogenized_8.1mmSW_900°C_180min_00142)

Mittlere Sehnenlänge	6,8 µm
Korngröße (ASTM)	11,1
Korngröße (G643)	11,1
Kornstreckung	97,2 %

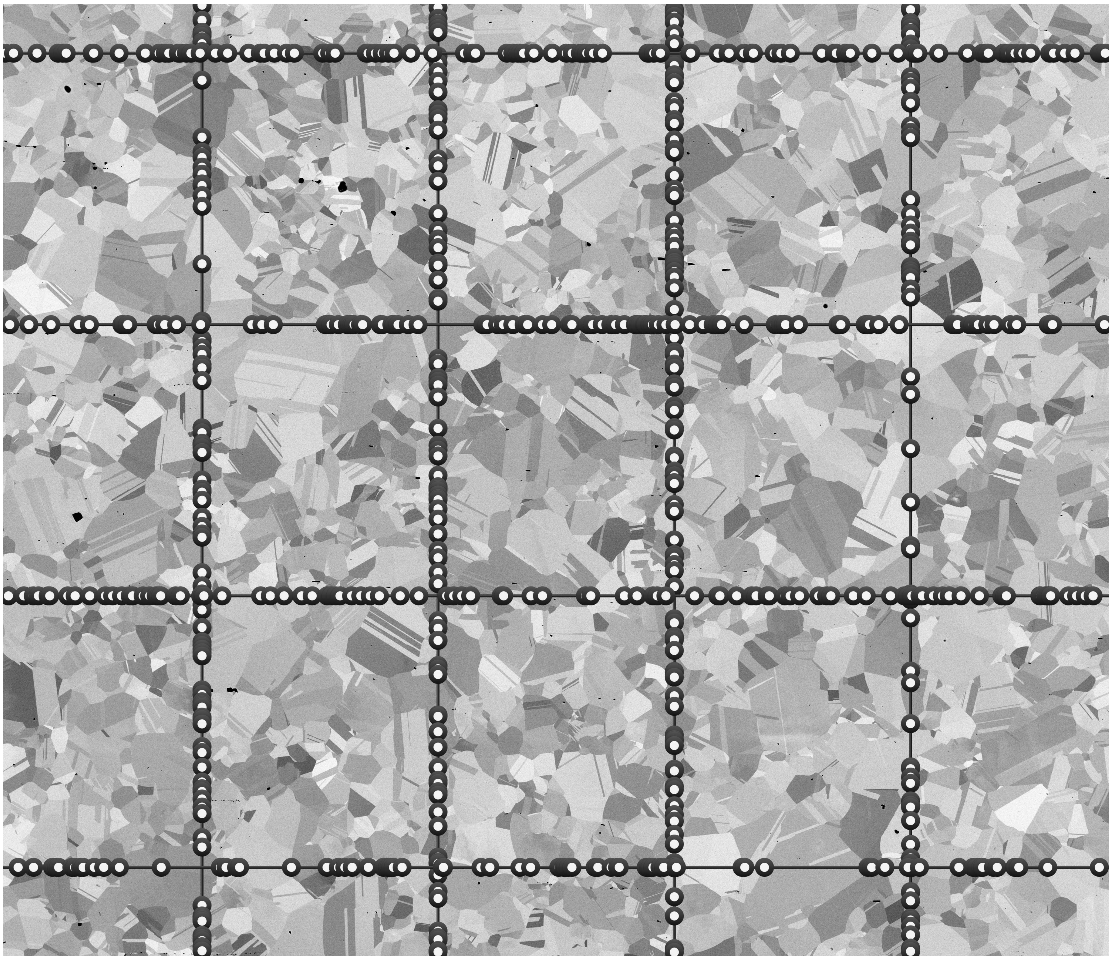

2.1. Statistische Analyse

Statistische Daten	Länge
Anzahl Objekte	698
Minimum	0,3 µm
Maximum	59,4 µm
Mittelwert	6,8 µm
Standardabweichung	7,1 µm
Schiefe	0,0
Standardabweichung (n-1)	7,1 µm
Varianz	50,8 µm²
Varianz (n-1)	50,9 µm²
Summe	4'726,8 µm

Statistische Daten	Länge
Quadratsumme	67'498,4 µm²
Kubiksumme	1'547'809,8 µm³

2.1.1. Chord Lenght Distribution

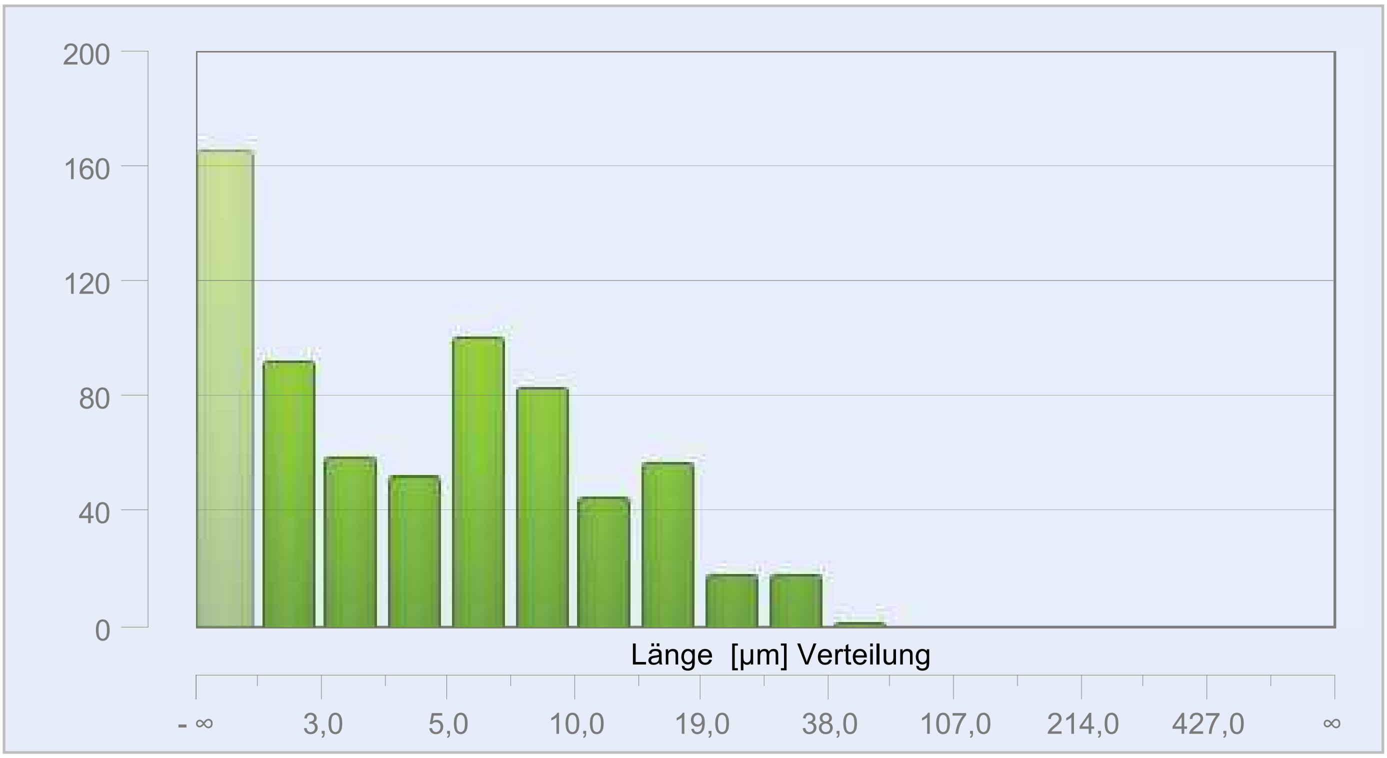

Start	Ende	Absolute Häufigkeit	Absolute Häufigkeit (kumuliert)	Relative Häufigkeit [%]	Relative Häufigkeit (kumuliert) [%]
	2,0 µm	165	165	24	24
2,0 µm	3,0 µm	93	258	13	37
3,0 µm	4,0 µm	59	317	8	45
4,0 µm	5,0 µm	53	370	8	53
5,0 µm	7,0 µm	101	471	14	67
7,0 µm	10,0 µm	83	554	12	79
10,0 µm	13,0 µm	46	600	7	86
13,0 µm	19,0 µm	58	658	8	94
19,0 µm	27,0 µm	19	677	3	97
27,0 µm	38,0 µm	19	696	3	100
38,0 µm	75,0 µm	2	698	0	100
75,0 µm	107,0 µm	0	698	0	100
107,0 µm	151,0 µm	0	698	0	100
151,0 µm	214,0 µm	0	698	0	100
214,0 µm	302,0 µm	0	698	0	100
302,0 µm	427,0 µm	0	698	0	100
427,0 µm	600,0 µm	0	698	0	100
600,0 µm		0	698	0	100

3. Single Result 2 (CrCoNi - ASTM E 112_CrCoNi_homogenized_8.1mmSW_900°C_180min_00143)

Mittlere Sehnenlänge	6,6 µm
Korngröße (ASTM)	11,2
Korngröße (G643)	11,1
Kornstreckung	83,7 %

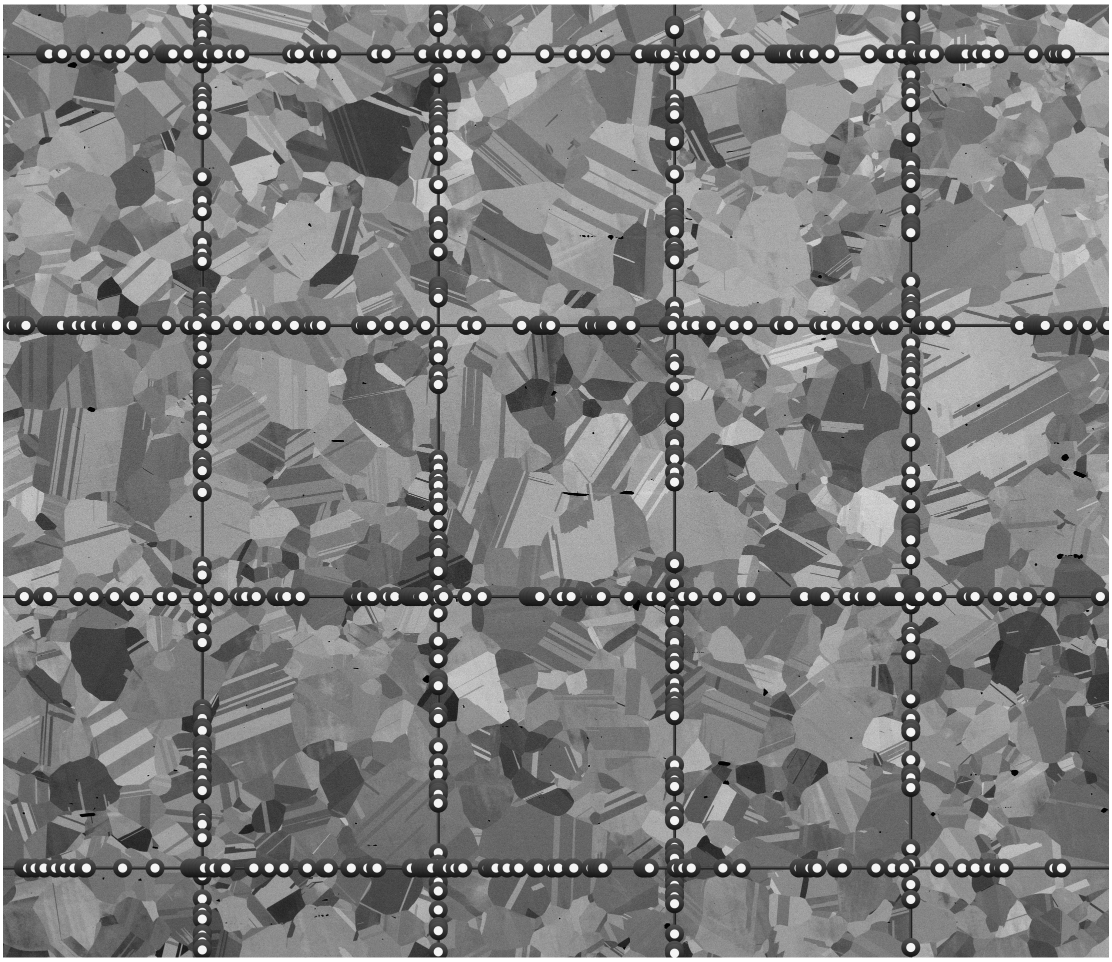

3.1. Statistische Analyse

Statistische Daten	Länge
Anzahl Objekte	712
Minimum	0,3 µm
Maximum	45,1 µm
Mittelwert	6,6 µm
Standardabweichung	7,0 µm
Schiefe	0,0
Standardabweichung (n-1)	7,0 µm
Varianz	48,7 µm²
Varianz (n-1)	48,8 µm²
Summe	4'726,8 µm
Quadratsumme	66'056,6 µm²
Kubiksumme	1'400'014,6 µm³

3.1.1. Chord Lenght Distribution

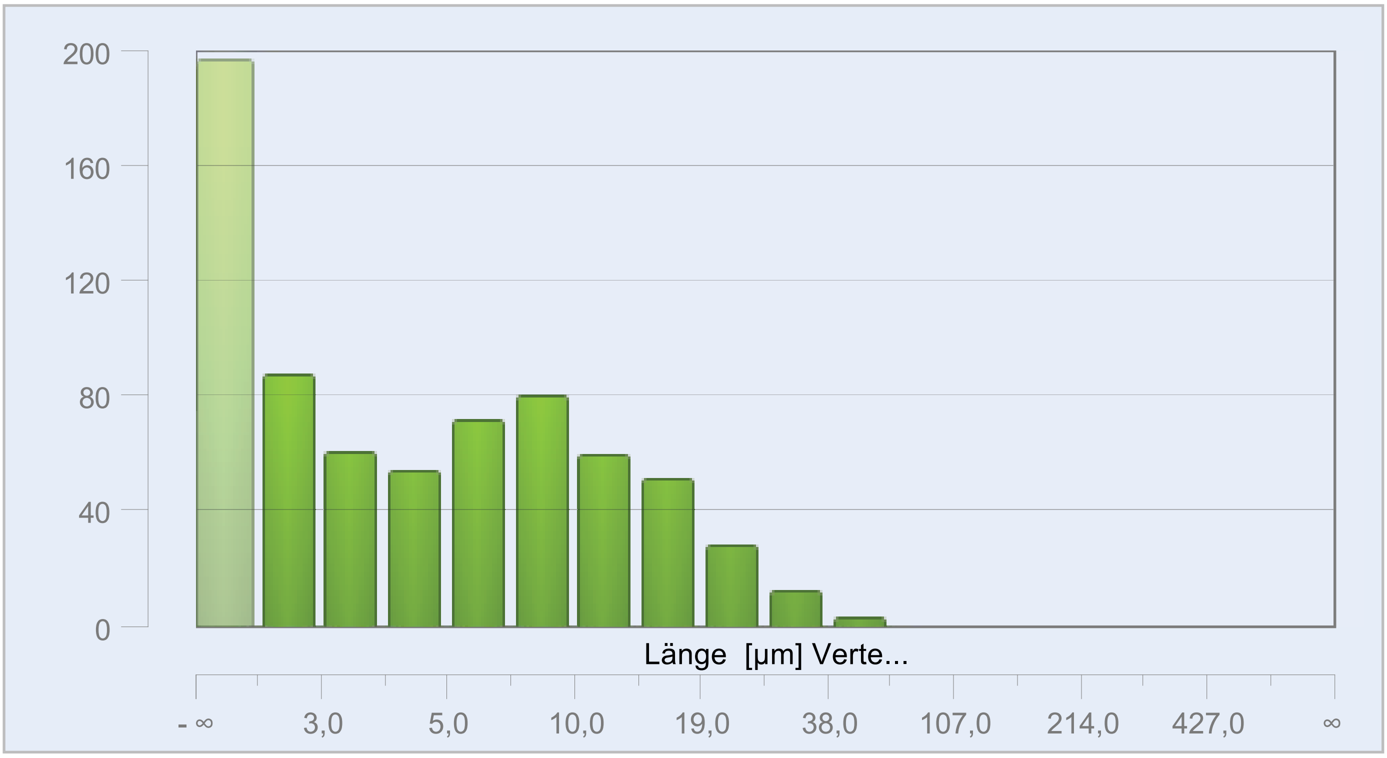

Start	Ende	Absolute Häufigkeit	Absolute Häufigkeit (kumuliert)	Relative Häufigkeit [%]	Relative Häufigkeit (kumuliert) [%]
	2,0 µm	197	197	28	28
2,0 µm	3,0 µm	88	285	12	40
3,0 µm	4,0 µm	61	346	9	49
4,0 µm	5,0 µm	55	401	8	56
5,0 µm	7,0 µm	72	473	10	66
7,0 µm	10,0 µm	81	554	11	78
10,0 µm	13,0 µm	60	614	8	86
13,0 µm	19,0 µm	52	666	7	94
19,0 µm	27,0 µm	29	695	4	98
27,0 µm	38,0 µm	13	708	2	99
38,0 µm	75,0 µm	4	712	1	100
75,0 µm	107,0 µm	0	712	0	100
107,0 µm	151,0 µm	0	712	0	100
151,0 µm	214,0 µm	0	712	0	100
214,0 µm	302,0 µm	0	712	0	100
302,0 µm	427,0 µm	0	712	0	100
427,0 µm	600,0 µm	0	712	0	100
600,0 µm		0	712	0	100

4. Single Result 3 (CrCoNi - ASTM E 112_CrCoNi_homogenized_8.1mmSW_900°C_180min_00144)

Mittlere Sehnenlänge	7,5 µm
Korngröße (ASTM)	10,8
Korngröße (G643)	10,8
Kornstreckung	99 %

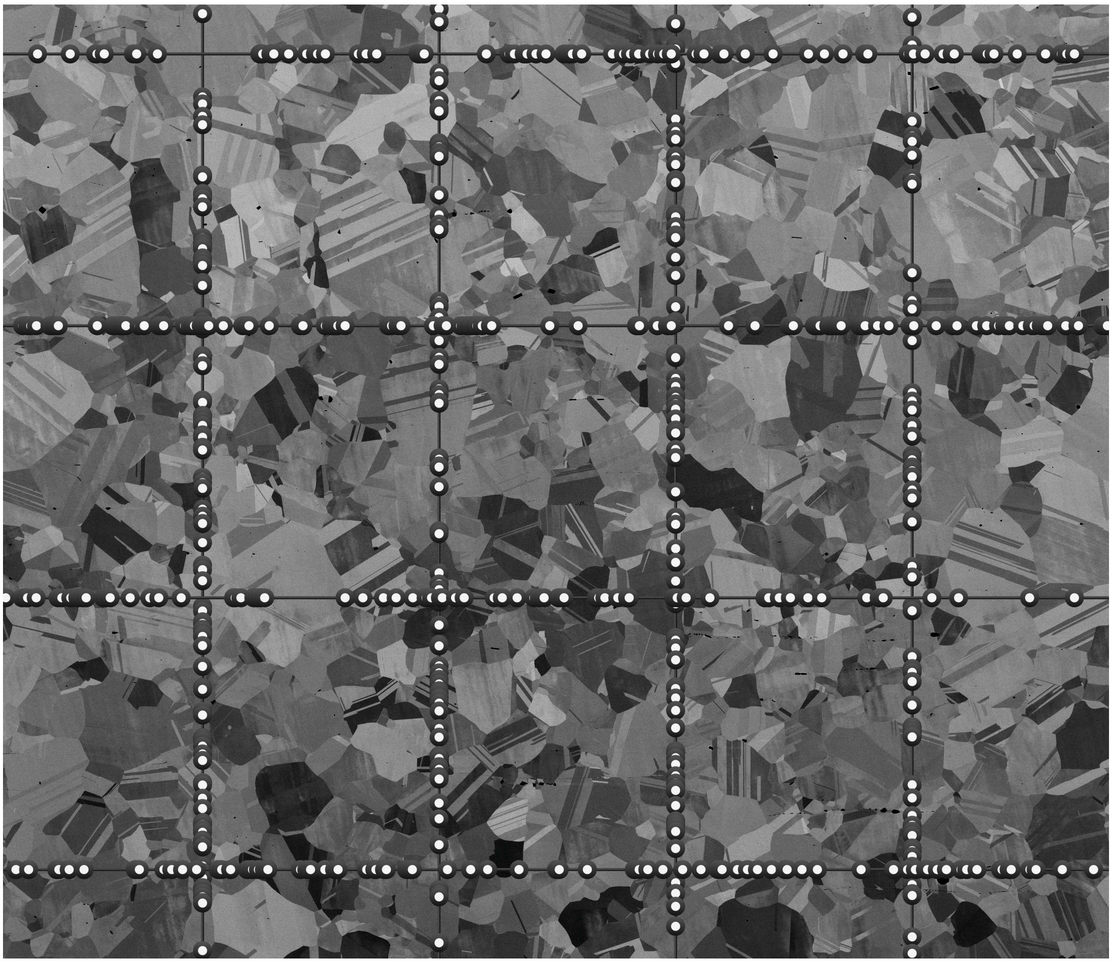

4.1. Statistische Analyse

Statistische Daten	Länge
Anzahl Objekte	627
Minimum	0,3 µm
Maximum	58,8 µm
Mittelwert	7,5 µm
Standardabweichung	8,1 µm
Schiefe	0,0
Standardabweichung (n-1)	8,1 µm
Varianz	65,2 µm²
Varianz (n-1)	65,3 µm²
Summe	4'729,9 µm
Quadratsumme	76'571,4 µm²
Kubiksumme	1'941'656,0 µm³

4.1.1. Chord Lenght Distribution

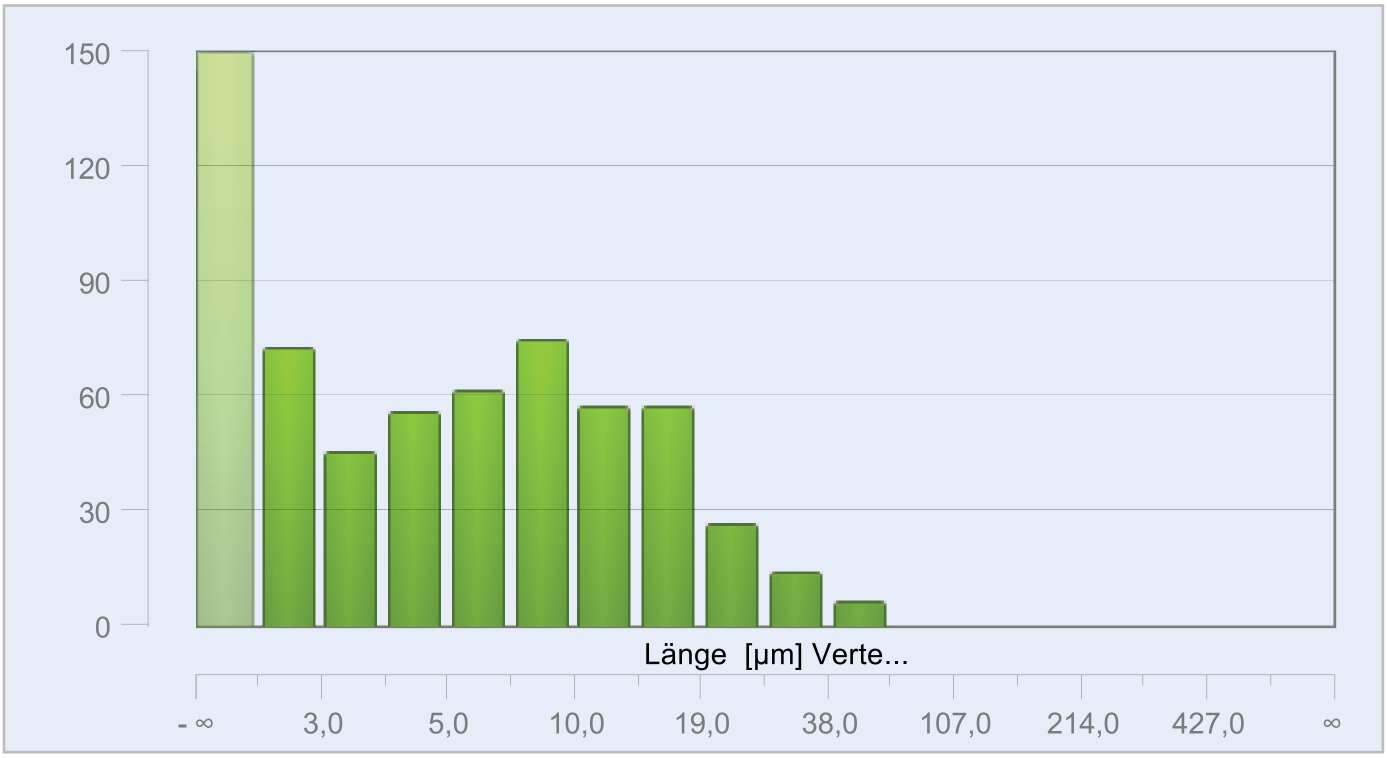

Start	Ende	Absolute Häufigkeit	Absolute Häufigkeit (kumuliert)	Relative Häufigkeit [%]	Relative Häufigkeit (kumuliert) [%]
	2,0 µm	150	150	24	24
2,0 µm	3,0 µm	73	223	12	36
3,0 µm	4,0 µm	46	269	7	43
4,0 µm	5,0 µm	56	325	9	52
5,0 µm	7,0 µm	62	387	10	62
7,0 µm	10,0 µm	75	462	12	74
10,0 µm	13,0 µm	58	520	9	83
13,0 µm	19,0 µm	58	578	9	92
19,0 µm	27,0 µm	27	605	4	96
27,0 µm	38,0 µm	15	620	2	99
38,0 µm	75,0 µm	7	627	1	100
75,0 µm	107,0 µm	0	627	0	100
107,0 µm	151,0 µm	0	627	0	100
151,0 µm	214,0 µm	0	627	0	100
214,0 µm	302,0 µm	0	627	0	100
302,0 µm	427,0 µm	0	627	0	100
427,0 µm	600,0 µm	0	627	0	100
600,0 µm		0	627	0	100

5. Single Result 4 (CrCoNi - ASTM E 112_CrCoNi_homogenized_8.1mmSW_900°C_180min_00145)

Mittlere Sehnenlänge	6,7 µm
Korngröße (ASTM)	11,1
Korngröße (G643)	11,1
Kornstreckung	98,8 %

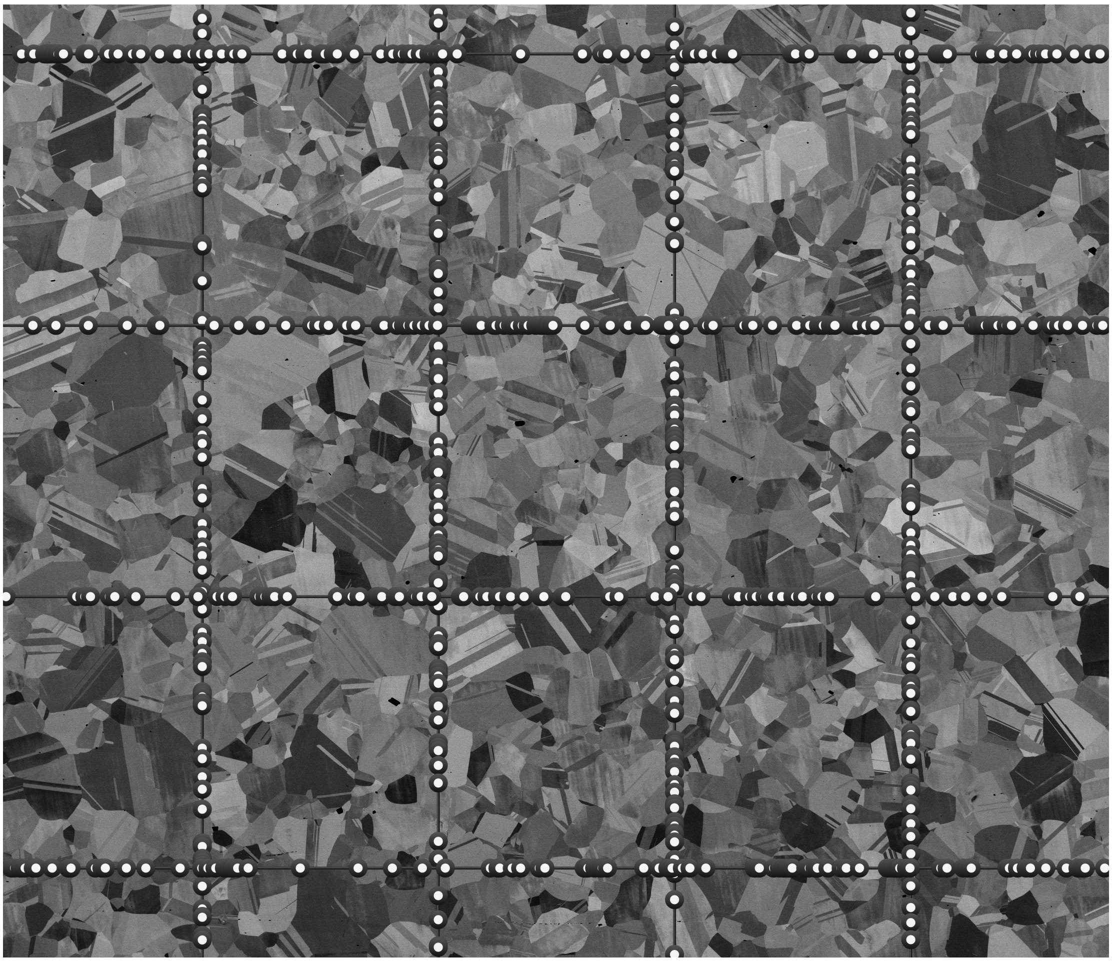

5.1. Statistische Analyse

Statistische Daten	Länge
Anzahl Objekte	702
Minimum	0,3 µm
Maximum	40,6 µm
Mittelwert	6,7 µm
Standardabweichung	6,5 µm
Schiefe	0,0
Standardabweichung (n-1)	6,5 µm
Varianz	41,9 µm²
Varianz (n-1)	42,0 µm²
Summe	4'726,8 µm
Quadratsumme	61'273,5 µm²
Kubiksumme	1'177'456,0 µm³

5.1.1. Chord Lenght Distribution

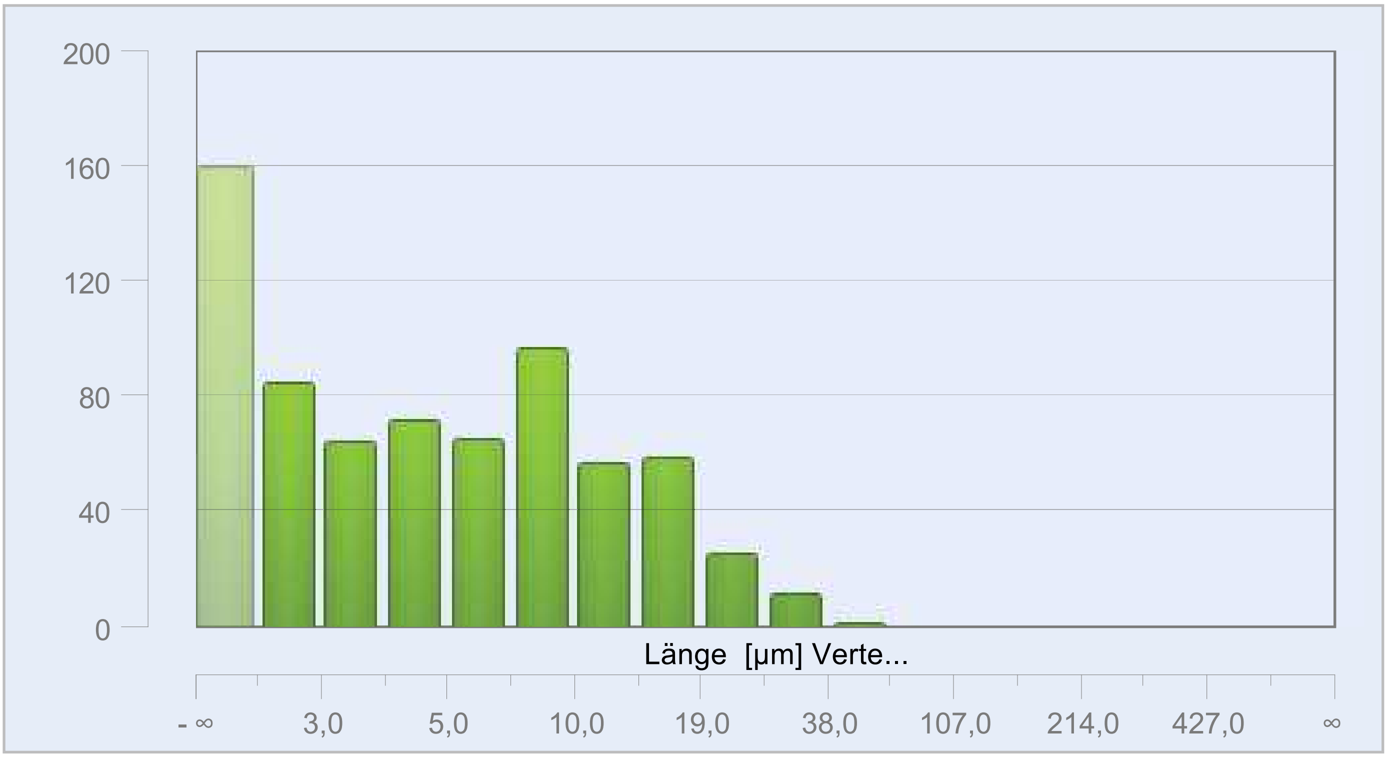

Start	Ende	Absolute Häufigkeit	Absolute Häufigkeit (kumuliert)	Relative Häufigkeit [%]	Relative Häufigkeit (kumuliert) [%]
	2,0 µm	160	160	23	23
2,0 µm	3,0 µm	85	245	12	35
3,0 µm	4,0 µm	65	310	9	44
4,0 µm	5,0 µm	72	382	10	54
5,0 µm	7,0 µm	66	448	9	64
7,0 µm	10,0 µm	97	545	14	78
10,0 µm	13,0 µm	58	603	8	86
13,0 µm	19,0 µm	59	662	8	94
19,0 µm	27,0 µm	26	688	4	98
27,0 µm	38,0 µm	12	700	2	100
38,0 µm	75,0 µm	2	702	0	100
75,0 µm	107,0 µm	0	702	0	100
107,0 µm	151,0 µm	0	702	0	100
151,0 µm	214,0 µm	0	702	0	100
214,0 µm	302,0 µm	0	702	0	100
302,0 µm	427,0 µm	0	702	0	100
427,0 µm	600,0 µm	0	702	0	100
600,0 µm		0	702	0	100
