# Supplementary material for: Benchmark dataset of the effect of grain size on strength in the single-phase FCC CrCoNi medium entropy alloy
Source: Data Brief. 2019 Oct 1;27:104592. doi: 10.1016/j.dib.2019.104592 (PMC6812030; doi:10.1016/j.dib.2019.104592)
Supplement: Multimedia component 1 [file mmc1.zip › CrCoNi_1173K_180min/CrCoNi_1173K_180min_d=16μm.pdf]

Analysebericht

Aug 29, 2017 3:50:33 PM

powered by imagic.ch

1. cumulative Result 1

Number of images	4
Grain size (ASTM)	8.6
Grain size (G643)	8.6
Grain stretching	99 %
Mean chord length	16.2 µm

2. Single Result 1 (CrCoNi Twins grain size_ASTM 900C 180min_00211)

Mean chord length	14.4 µm
Grain size (ASTM)	8.9
Grain size (G643)	8.9
Grain stretching	99.8 %

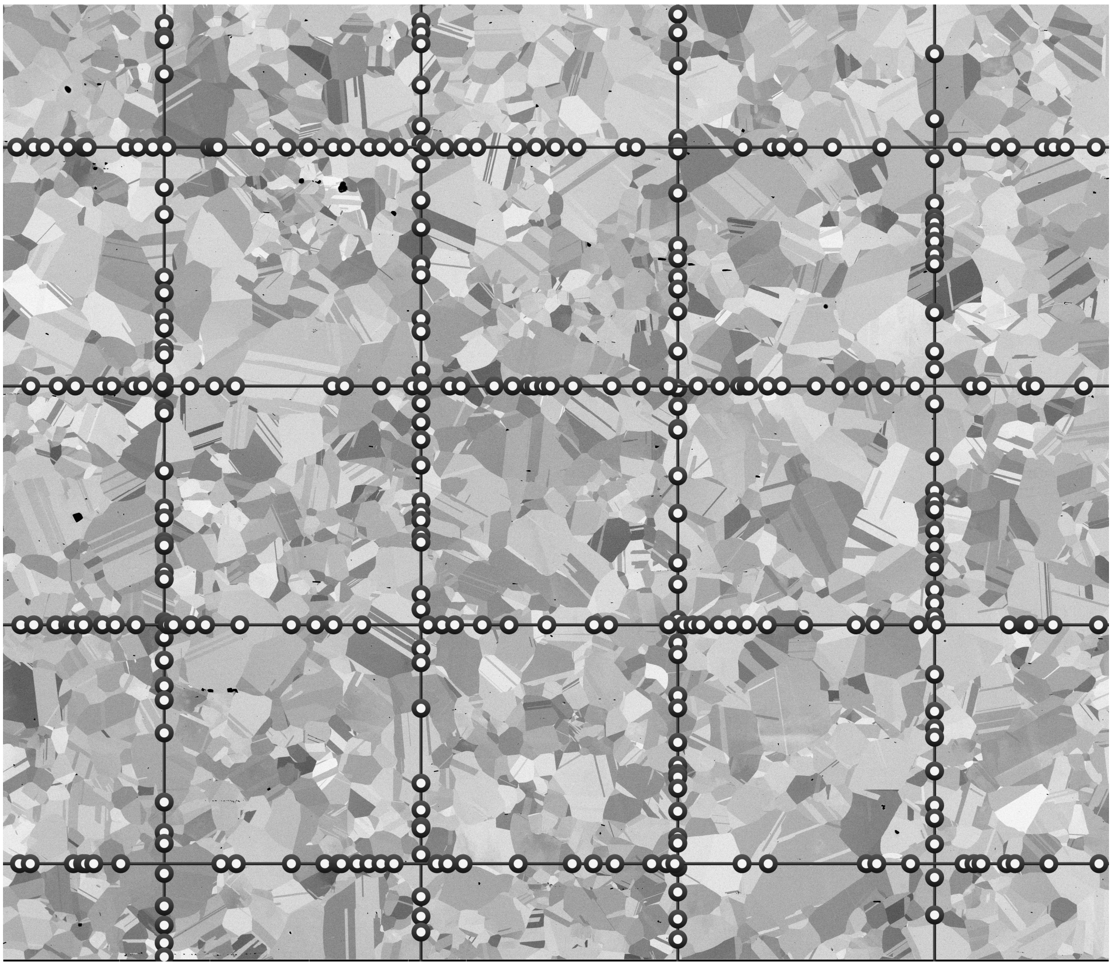

2.1. Statistical Analysis

Statistical Data	Length
Object Count	328
Minimum	0.9 µm
Maximum	61.3 µm
Average	14.4 µm
Standard deviation	10.1 µm
Skewness	0.0
Standard deviation (n-1)	10.1 µm
Variance	101.4 µm²
Variance (n-1)	101.7 µm²
Sum	4'739.2 µm

Statistical Data	Length
Sum of squares	101'736.6 µm²
Sum of cubes	2'953'338.1 µm³

2.1.1. Chord Lenght Distribution

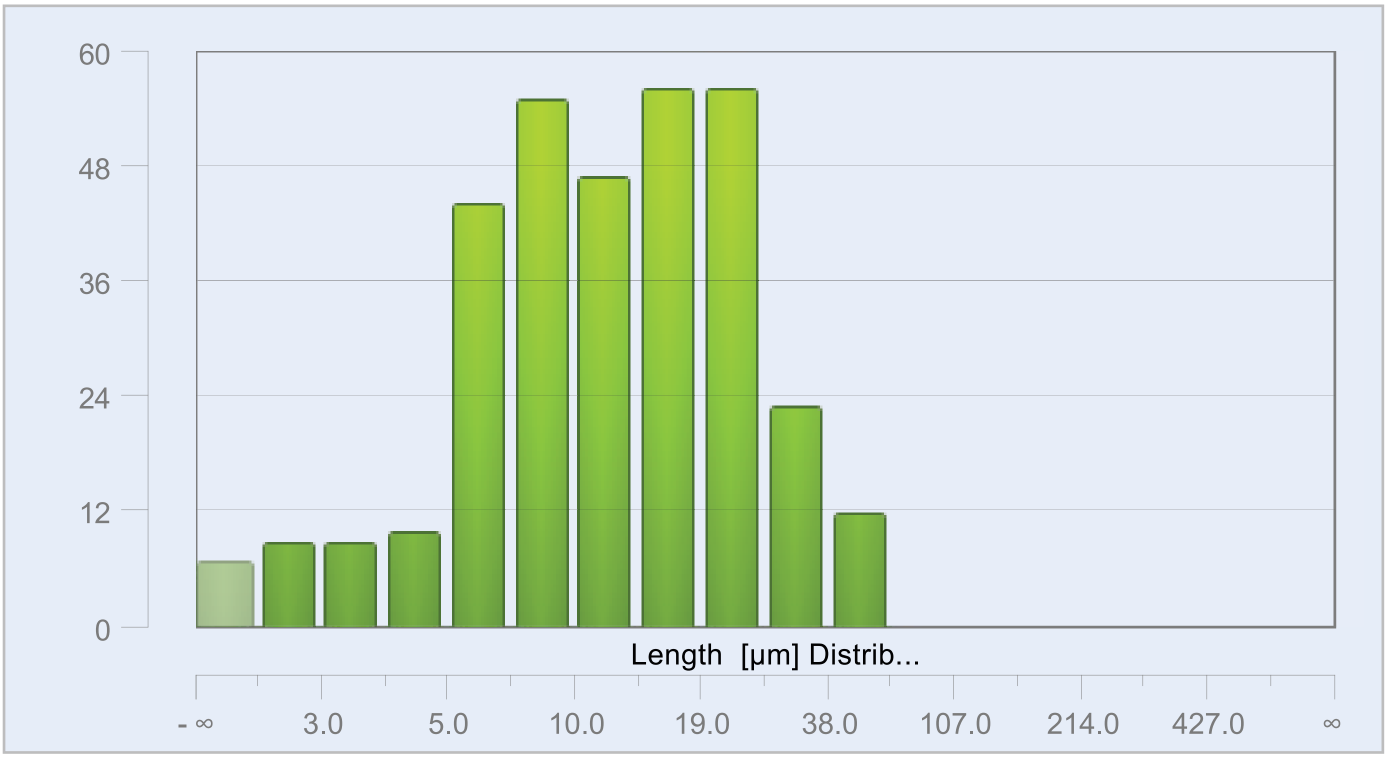

Start	End	Absolute Frequency	Absolute Frequency (accumulated)	Relative Frequency [%]	Relative Frequency (accumulated) [%]
	2.0 µm	7	7	2	2
2.0 µm	3.0 µm	9	16	3	5
3.0 µm	4.0 µm	9	25	3	8
4.0 µm	5.0 µm	10	35	3	11
5.0 µm	7.0 µm	44	79	13	24
7.0 µm	10.0 µm	55	134	17	41
10.0 µm	13.0 µm	47	181	14	55
13.0 µm	19.0 µm	56	237	17	72
19.0 µm	27.0 µm	56	293	17	89
27.0 µm	38.0 µm	23	316	7	96
38.0 µm	75.0 µm	12	328	4	100
75.0 µm	107.0 µm	0	328	0	100
107.0 µm	151.0 µm	0	328	0	100
151.0 µm	214.0 µm	0	328	0	100
214.0 µm	302.0 µm	0	328	0	100
302.0 µm	427.0 µm	0	328	0	100
427.0 µm	600.0 µm	0	328	0	100
600.0 µm		0	328	0	100

3. Single Result 2 (CrCoNi Twins grain size_ASTM 900C 180min_00212)

Mean chord length	16.9 µm
Grain size (ASTM)	8.5
Grain size (G643)	8.4
Grain stretching	84.6 %

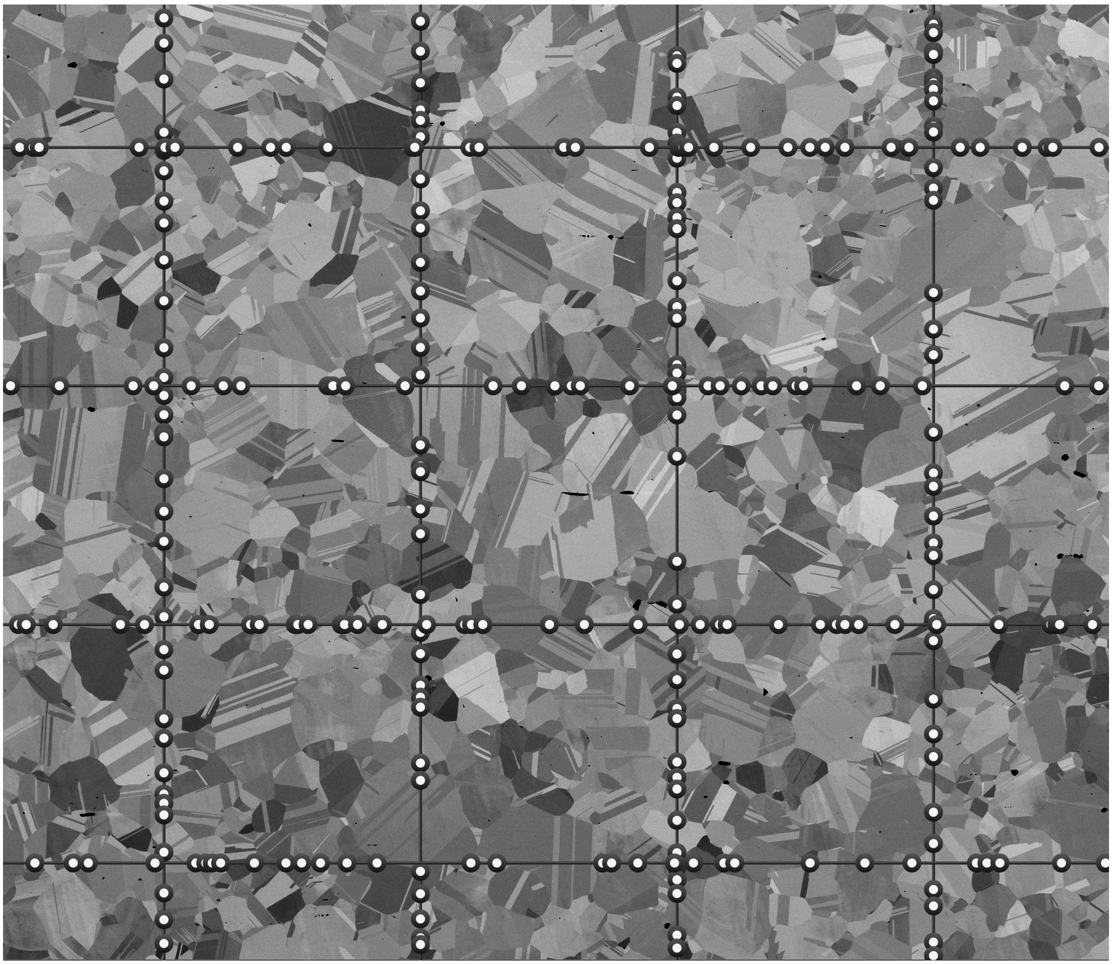

3.1. Statistical Analysis

Statistical Data	Length
Object Count	281
Minimum	1.2 µm
Maximum	81.7 µm
Average	16.9 µm
Standard deviation	12.4 µm
Skewness	0.0
Standard deviation (n-1)	12.5 µm
Variance	154.6 µm²
Variance (n-1)	155.1 µm²
Sum	4'736.7 µm
Sum of squares	123'276.8 µm²
Sum of cubes	4'392'756.8 µm³

3.1.1. Chord Lenght Distribution

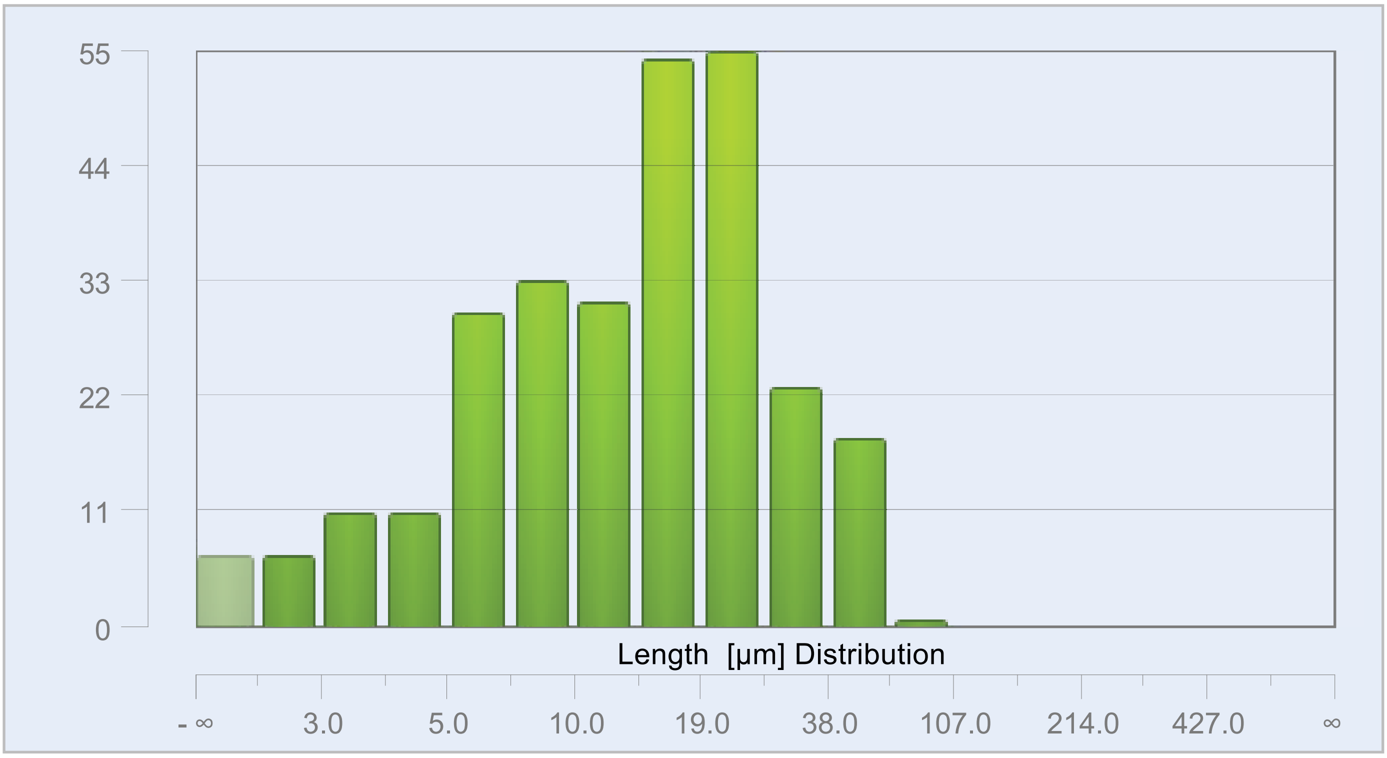

Start	End	Absolute Frequency	Absolute Frequency (accumulated)	Relative Frequency [%]	Relative Frequency (accumulated) [%]
	2.0 µm	7	7	2	2
2.0 µm	3.0 µm	7	14	2	5
3.0 µm	4.0 µm	11	25	4	9
4.0 µm	5.0 µm	11	36	4	13
5.0 µm	7.0 µm	30	66	11	23
7.0 µm	10.0 µm	33	99	12	35
10.0 µm	13.0 µm	31	130	11	46
13.0 µm	19.0 µm	54	184	19	65
19.0 µm	27.0 µm	55	239	20	85
27.0 µm	38.0 µm	23	262	8	93
38.0 µm	75.0 µm	18	280	6	100
75.0 µm	107.0 µm	1	281	0	100
107.0 µm	151.0 µm	0	281	0	100
151.0 µm	214.0 µm	0	281	0	100
214.0 µm	302.0 µm	0	281	0	100
302.0 µm	427.0 µm	0	281	0	100
427.0 µm	600.0 µm	0	281	0	100
600.0 µm		0	281	0	100

4. Single Result 3 (CrCoNi Twins grain size_ASTM 900C 180min_00213)

Mean chord length	17.5 µm
Grain size (ASTM)	8.4
Grain size (G643)	8.3
Grain stretching	87.3 %

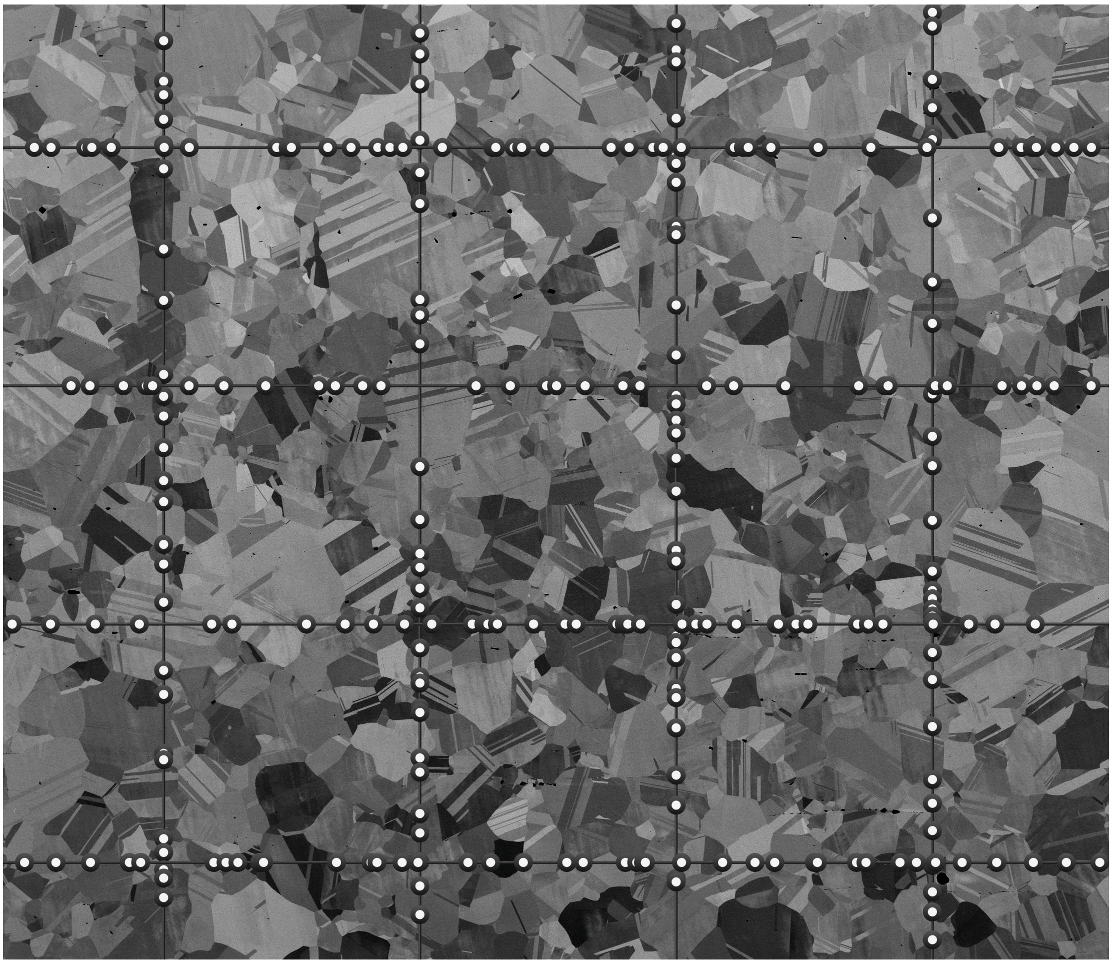

4.1. Statistical Analysis

Statistical Data	Length
Object Count	270
Minimum	1.1 µm
Maximum	70.4 µm
Average	17.5 µm
Standard deviation	11.8 µm
Skewness	0.0
Standard deviation (n-1)	11.8 µm
Variance	138.5 µm²
Variance (n-1)	139.0 µm²
Sum	4'734.2 µm
Sum of squares	120'414.2 µm²
Sum of cubes	3'906'023.7 µm³

4.1.1. Chord Lenght Distribution

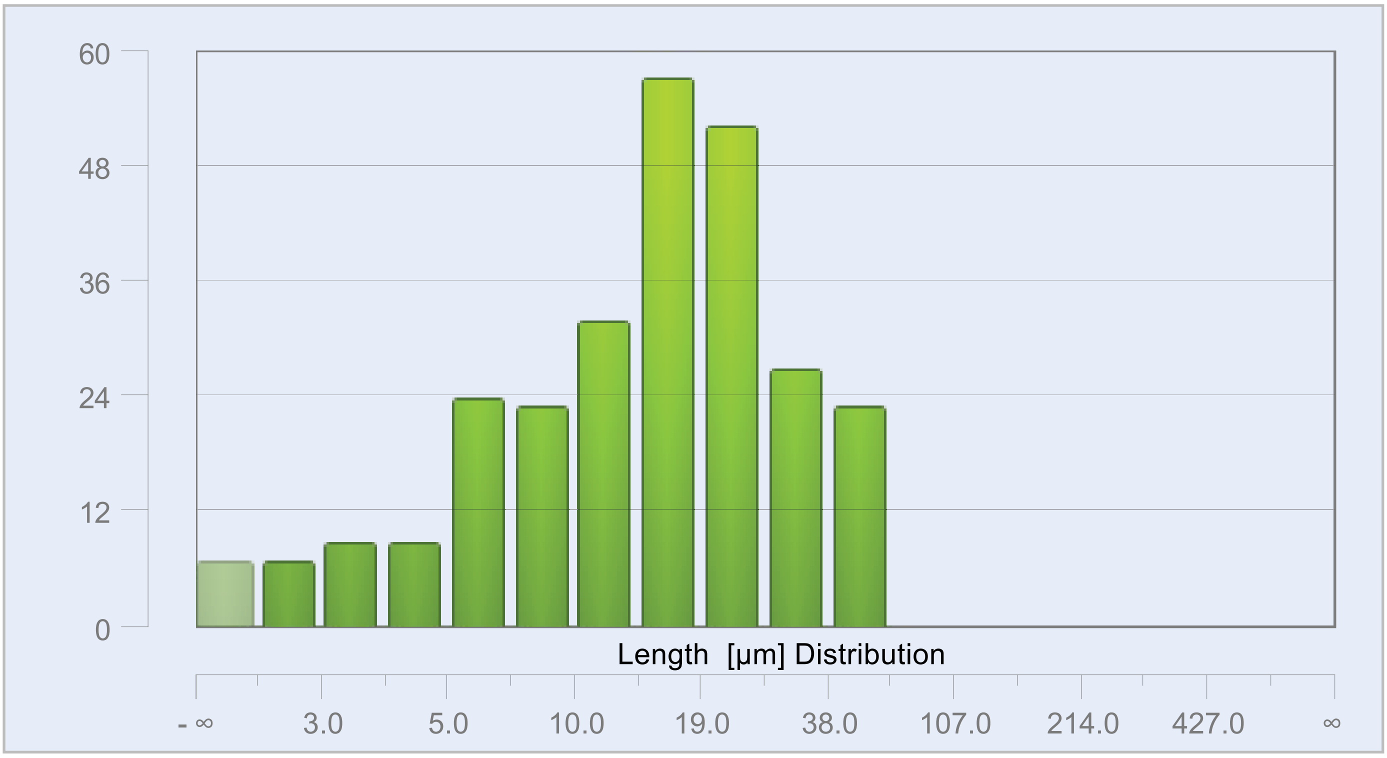

Start	End	Absolute Frequency	Absolute Frequency (accumulated)	Relative Frequency [%]	Relative Frequency (accumulated) [%]
	2.0 µm	7	7	3	3
2.0 µm	3.0 µm	7	14	3	5
3.0 µm	4.0 µm	9	23	3	9
4.0 µm	5.0 µm	9	32	3	12
5.0 µm	7.0 µm	24	56	9	21
7.0 µm	10.0 µm	23	79	9	29
10.0 µm	13.0 µm	32	111	12	41
13.0 µm	19.0 µm	57	168	21	62
19.0 µm	27.0 µm	52	220	19	81
27.0 µm	38.0 µm	27	247	10	91
38.0 µm	75.0 µm	23	270	9	100
75.0 µm	107.0 µm	0	270	0	100
107.0 µm	151.0 µm	0	270	0	100
151.0 µm	214.0 µm	0	270	0	100
214.0 µm	302.0 µm	0	270	0	100
302.0 µm	427.0 µm	0	270	0	100
427.0 µm	600.0 µm	0	270	0	100
600.0 µm		0	270	0	100

5. Single Result 4 (CrCoNi Twins grain size_ASTM 900C 180min_00214)

Mean chord length	16.4 µm
Grain size (ASTM)	8.6
Grain size (G643)	8.5
Grain stretching	92.6 %

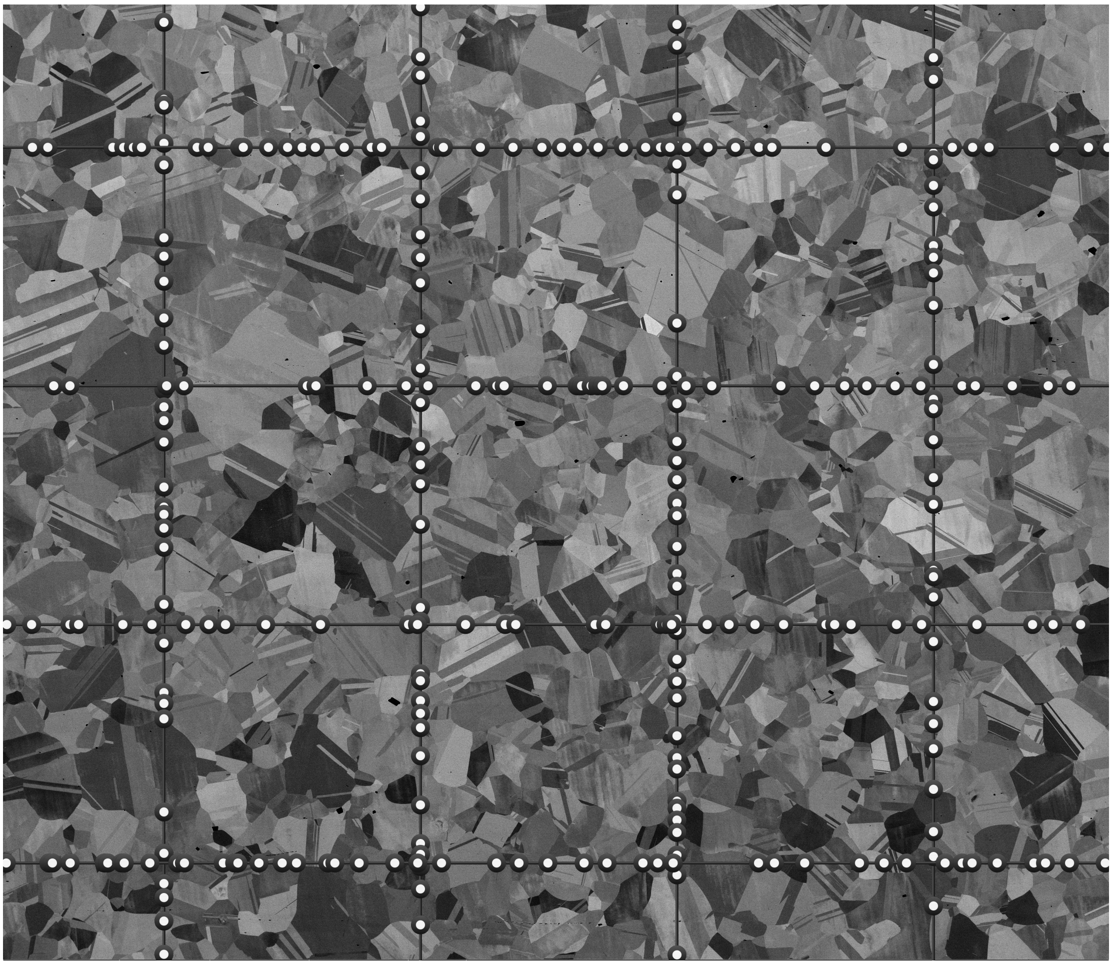

5.1. Statistical Analysis

Statistical Data	Length
Object Count	288
Minimum	0.5 µm
Maximum	73.7 µm
Average	16.4 µm
Standard deviation	11.6 µm
Skewness	0.0
Standard deviation (n-1)	11.6 µm
Variance	134.6 µm²
Variance (n-1)	135.1 µm²
Sum	4'736.7 µm
Sum of squares	116'666.0 µm²
Sum of cubes	3'889'926.1 µm³

5.1.1. Chord Lenght Distribution

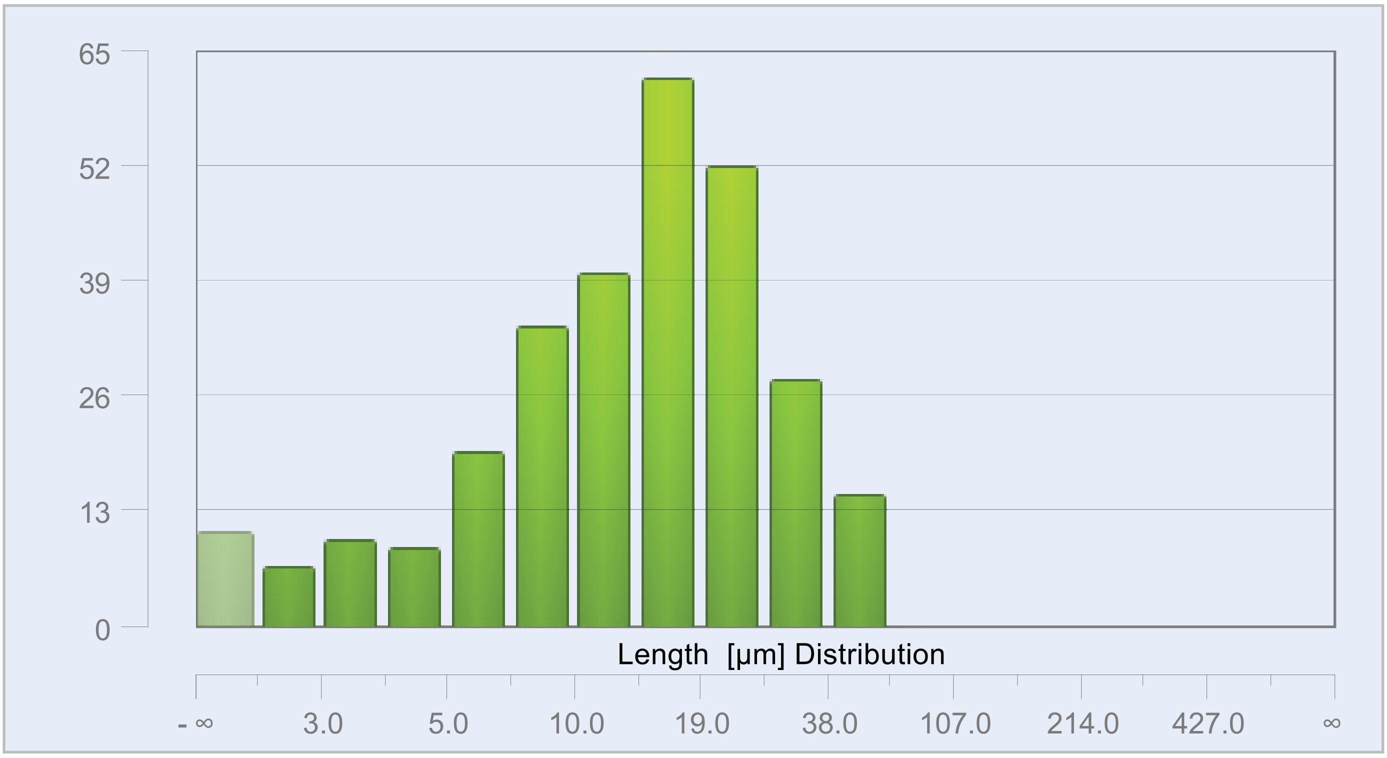

Start	End	Absolute Frequency	Absolute Frequency (accumulated)	Relative Frequency [%]	Relative Frequency (accumulated) [%]
	2.0 µm	11	11	4	4
2.0 µm	3.0 µm	7	18	2	6
3.0 µm	4.0 µm	10	28	3	10
4.0 µm	5.0 µm	9	37	3	13
5.0 µm	7.0 µm	20	57	7	20
7.0 µm	10.0 µm	34	91	12	32
10.0 µm	13.0 µm	40	131	14	45
13.0 µm	19.0 µm	62	193	22	67
19.0 µm	27.0 µm	52	245	18	85
27.0 µm	38.0 µm	28	273	10	95
38.0 µm	75.0 µm	15	288	5	100
75.0 µm	107.0 µm	0	288	0	100
107.0 µm	151.0 µm	0	288	0	100
151.0 µm	214.0 µm	0	288	0	100
214.0 µm	302.0 µm	0	288	0	100
302.0 µm	427.0 µm	0	288	0	100
427.0 µm	600.0 µm	0	288	0	100
600.0 µm		0	288	0	100
