# Supplementary material for: Benchmark dataset of the effect of grain size on strength in the single-phase FCC CrCoNi medium entropy alloy
Source: Data Brief. 2019 Oct 1;27:104592. doi: 10.1016/j.dib.2019.104592 (PMC6812030; doi:10.1016/j.dib.2019.104592)
Supplement: Multimedia component 1 [file mmc1.zip › CrCoNi_1173K_20min/CrCoNi_1173K_20min_c=2.9μm.pdf]

Analysebericht

Nov 8, 2017 3:58:24 PM

powered by imagic.ch

cumulative Result 1

Number of images	4
Grain size (ASTM)	13.6
Grain size (G643)	13.6
Grain stretching	99.4 %
Mean chord length	2.9 µm

Single Result 1 (CrCoNi - ASTM E 112_CrCoNi_homogenized_8.1mmSW_900°C_20min_00158)

Mean chord length	3 µm
Grain size (ASTM)	13.5
Grain size (G643)	13.5
Grain stretching	98 %

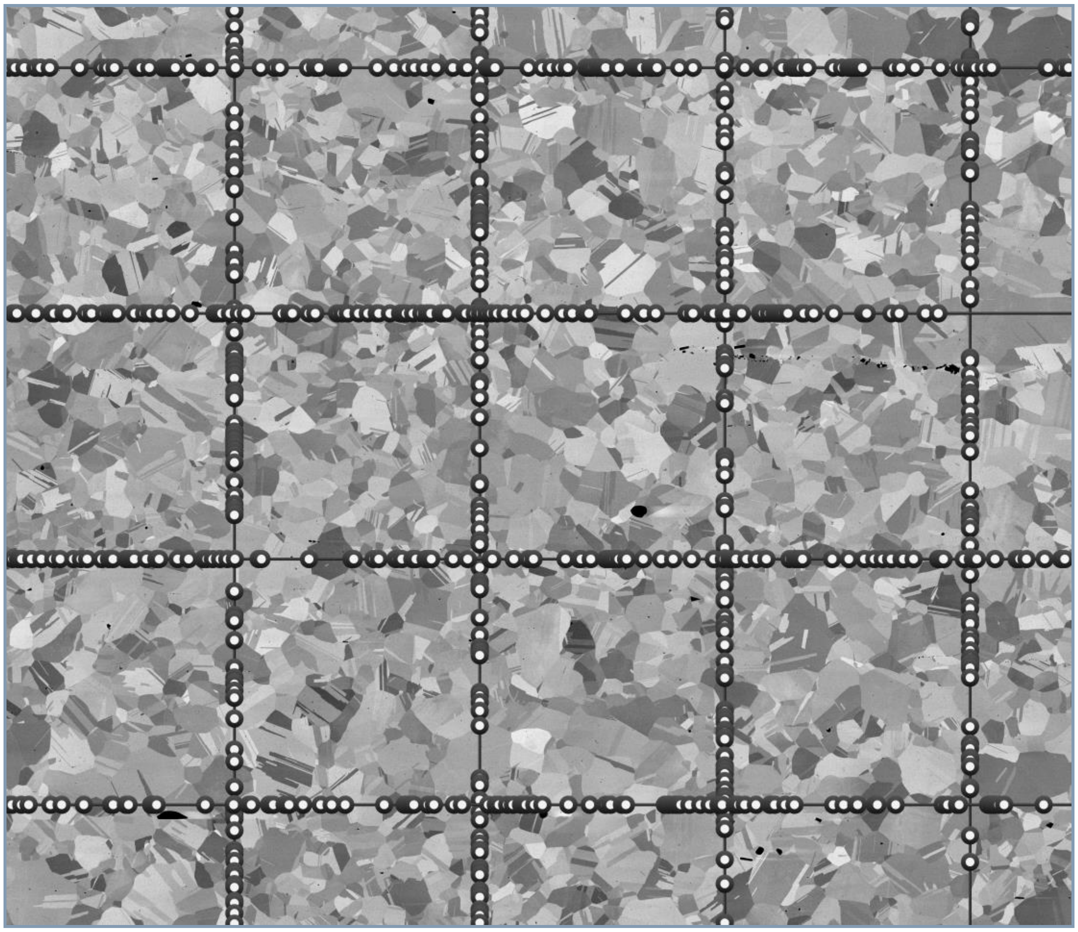

Statistical Analysis

Statistical Data	Length
Object Count	795
Minimum	0.2 µm
Maximum	39.6 µm

Average	3.0 µm
Standard deviation	3.2 µm
Skewness	0.0
Standard deviation (n-1)	3.2 µm
Variance	10.3 µm²
Variance (n-1)	10.3 µm²
Sum	2'365.6 µm
Sum of squares	15'240.2 µm²
Sum of cubes	183'166.2 µm³

Chord Lenght Distribution

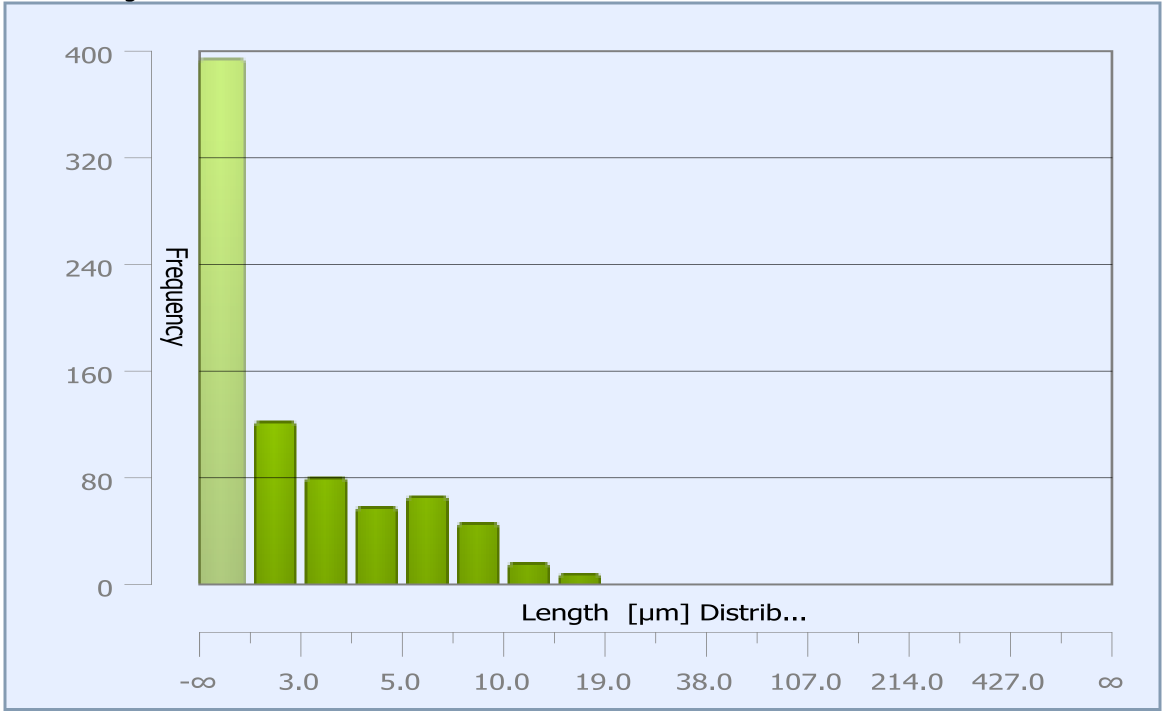

Start	End	Absolute Frequency	Absolute Frequency (accumulated)	Relative Frequency [%]	Relative Frequency (accumulated) [%]
	2.0 µm	393	393	49	49
2.0 µm	3.0 µm	122	515	15	65
3.0 µm	4.0 µm	80	595	10	75
4.0 µm	5.0 µm	58	653	7	82
5.0 µm	7.0 µm	67	720	8	91
7.0 µm	10.0 µm	47	767	6	96
10.0 µm	13.0 µm	17	784	2	99

13.0 µm	19.0 µm	9	793	1	100
19.0 µm	27.0 µm	1	794	0	100
27.0 µm	38.0 µm	0	794	0	100
38.0 µm	75.0 µm	1	795	0	100
75.0 µm	107.0 µm	0	795	0	100
107.0 µm	151.0 µm	0	795	0	100
151.0 µm	214.0 µm	0	795	0	100
214.0 µm	302.0 µm	0	795	0	100
302.0 µm	427.0 µm	0	795	0	100
427.0 µm	600.0 µm	0	795	0	100
600.0 µm		0	795	0	100

Single Result 2 (CrCoNi - ASTM E 112_CrCoNi_homogenized_8.1mmSW_900°C_20min_00159)

Mean chord length	2.9 µm
Grain size (ASTM)	13.6
Grain size (G643)	13.6
Grain stretching	95.9 %

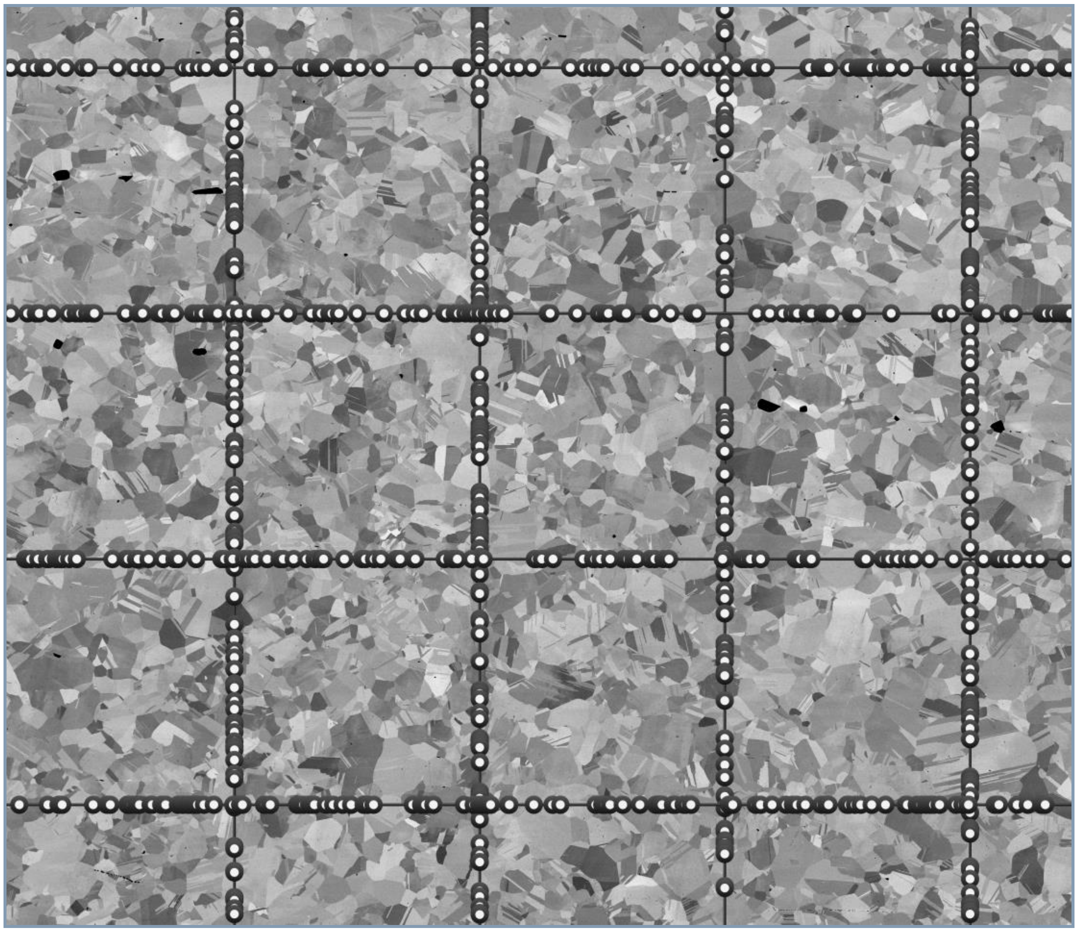

Statistical Analysis

Statistical Data	Length
Object Count	822
Minimum	0.2 µm
Maximum	19.3 µm
Average	2.9 µm
Standard deviation	3.0 µm
Skewness	0.0
Standard deviation (n-1)	3.0 µm
Variance	9.0 µm²
Variance (n-1)	9.0 µm²
Sum	2'363.7 µm
Sum of squares	14'155.4 µm²
Sum of cubes	126'474.1 µm³

Chord Lenght Distribution

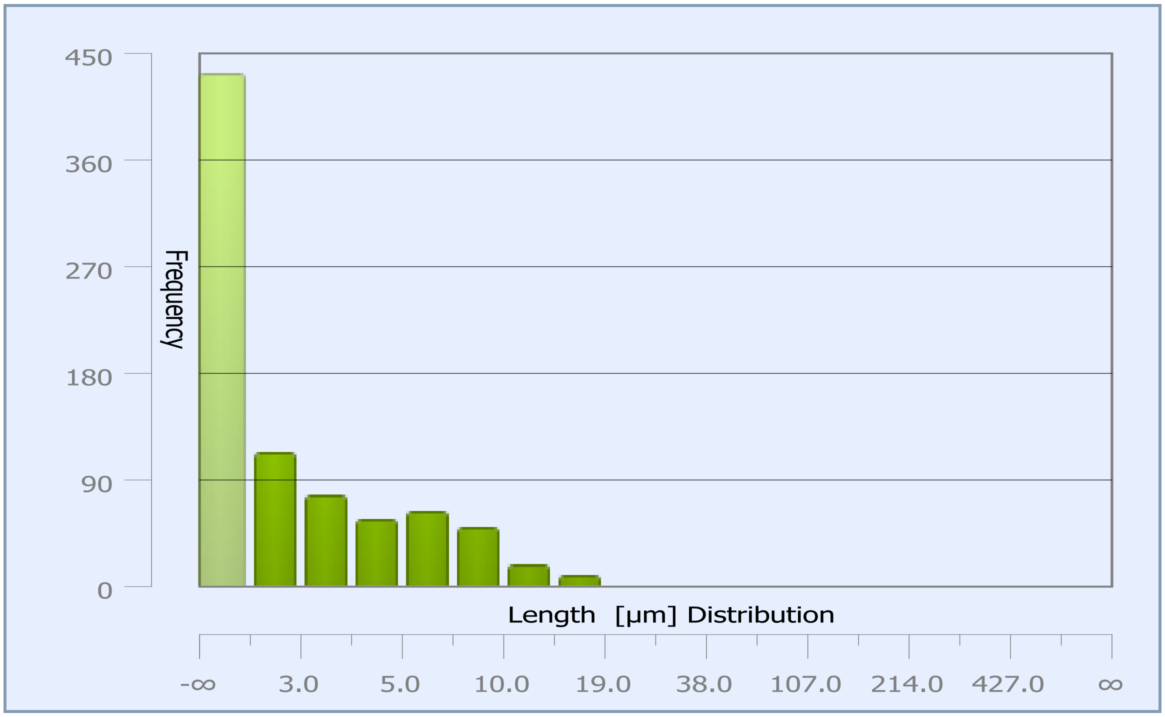

Start	End	Absolute Frequency	Absolute Frequency (accumulated)	Relative Frequency [%]	Relative Frequency (accumulated) [%]
	2.0 µm	430	430	52	52
2.0 µm	3.0 µm	113	543	14	66
3.0 µm	4.0 µm	77	620	9	75
4.0 µm	5.0 µm	58	678	7	82
5.0 µm	7.0 µm	64	742	8	90
7.0 µm	10.0 µm	50	792	6	96
10.0 µm	13.0 µm	20	812	2	99
13.0 µm	19.0 µm	9	821	1	100
19.0 µm	27.0 µm	1	822	0	100
27.0 µm	38.0 µm	0	822	0	100
38.0 µm	75.0 µm	0	822	0	100
75.0 µm	107.0 µm	0	822	0	100
107.0 µm	151.0 µm	0	822	0	100

151.0 µm	214.0 µm	0	822	0	100
214.0 µm	302.0 µm	0	822	0	100
302.0 µm	427.0 µm	0	822	0	100
427.0 µm	600.0 µm	0	822	0	100
600.0 µm		0	822	0	100

Single Result 3 (CrCoNi - ASTM E 112_CrCoNi_homogenized_8.1mmSW_900°C_20min_00160)

Mean chord length	2.7 µm
Grain size (ASTM)	13.8
Grain size (G643)	13.8
Grain stretching	95.6 %

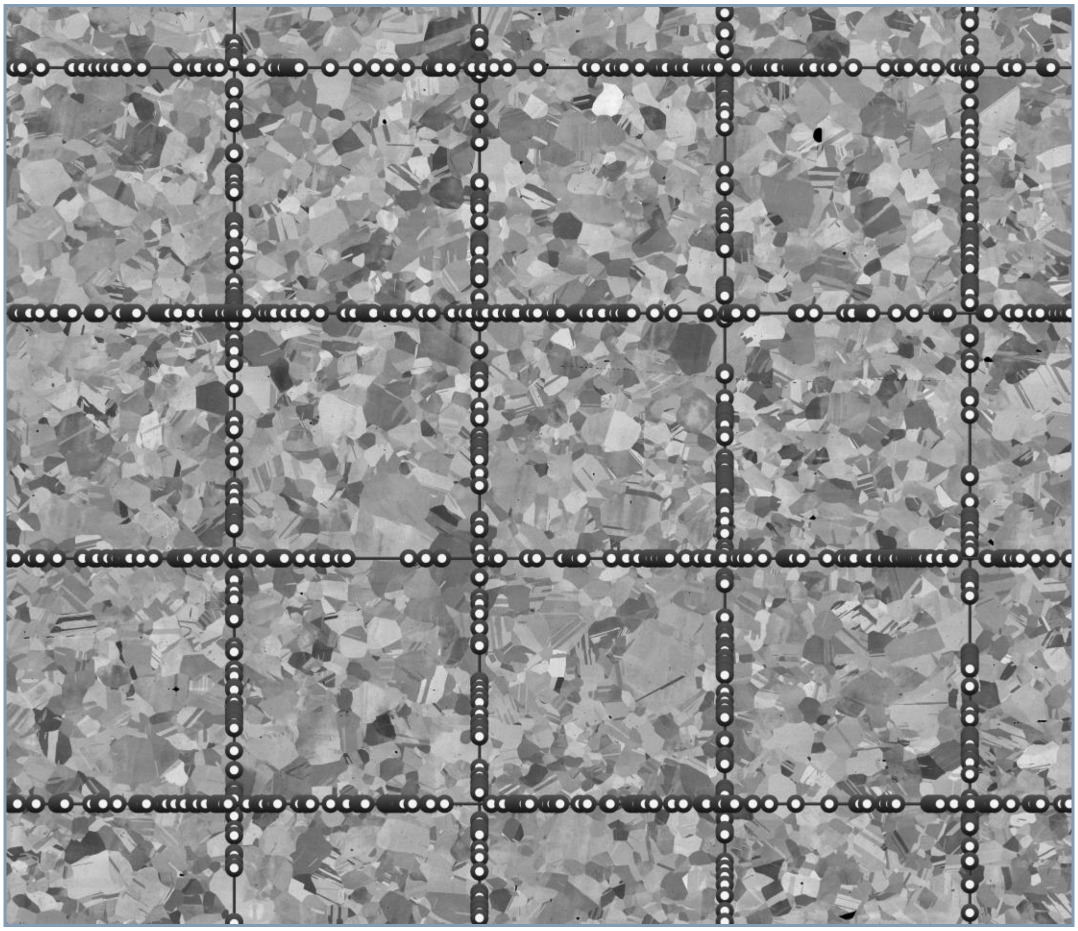

Statistical Analysis

Statistical Data	Length
Object Count	886

Minimum	0.1 µm
Maximum	18.5 µm
Average	2.7 µm
Standard deviation	2.7 µm
Skewness	0.0
Standard deviation (n-1)	2.7 µm
Variance	7.2 µm²
Variance (n-1)	7.2 µm²
Sum	2'361.6 µm
Sum of squares	12'677.1 µm²
Sum of cubes	102'137.3 µm³

Chord Lenght Distribution

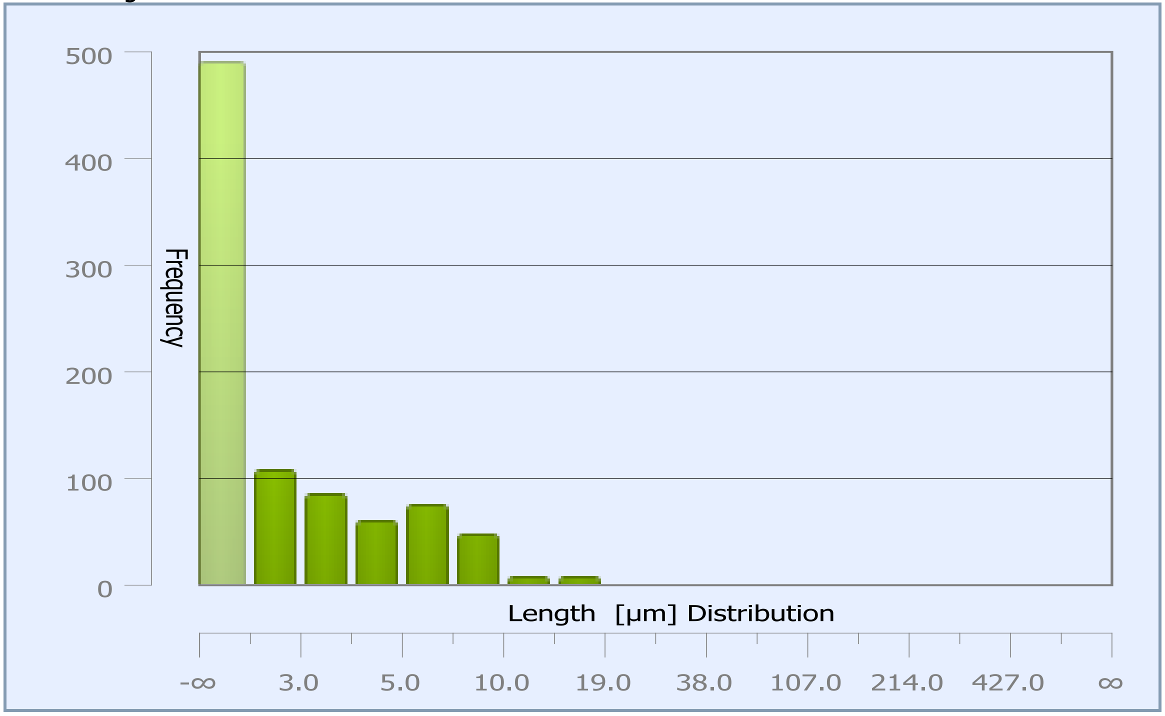

Start	End	Absolute Frequency	Absolute Frequency (accumulated)	Relative Frequency [%]	Relative Frequency (accumulated) [%]
	2.0 µm	489	489	55	55
2.0 µm	3.0 µm	108	597	12	67
3.0 µm	4.0 µm	86	683	10	77
4.0 µm	5.0 µm	62	745	7	84
5.0 µm	7.0 µm	76	821	9	93
7.0 µm	10.0 µm	48	869	5	98

10.0 µm	13.0 µm	9	878	1	99
13.0 µm	19.0 µm	8	886	1	100
19.0 µm	27.0 µm	0	886	0	100
27.0 µm	38.0 µm	0	886	0	100
38.0 µm	75.0 µm	0	886	0	100
75.0 µm	107.0 µm	0	886	0	100
107.0 µm	151.0 µm	0	886	0	100
151.0 µm	214.0 µm	0	886	0	100
214.0 µm	302.0 µm	0	886	0	100
302.0 µm	427.0 µm	0	886	0	100
427.0 µm	600.0 µm	0	886	0	100
600.0 µm		0	886	0	100

Single Result 4 (CrCoNi - ASTM E 112_CrCoNi_homogenized_8.1mmSW_900°C_20min_00161)

Mean chord length	3 µm
Grain size (ASTM)	13.5
Grain size (G643)	13.4
Grain stretching	86.9 %

Statistical Analysis

Statistical Data	Length
Object Count	785
Minimum	0.1 µm
Maximum	19.2 µm
Average	3.0 µm
Standard deviation	3.1 µm
Skewness	0.0
Standard deviation (n-1)	3.1 µm
Variance	9.4 µm²
Variance (n-1)	9.4 µm²
Sum	2'363.7 µm
Sum of squares	14'468.7 µm²
Sum of cubes	130'641.0 µm³

Chord Lenght Distribution

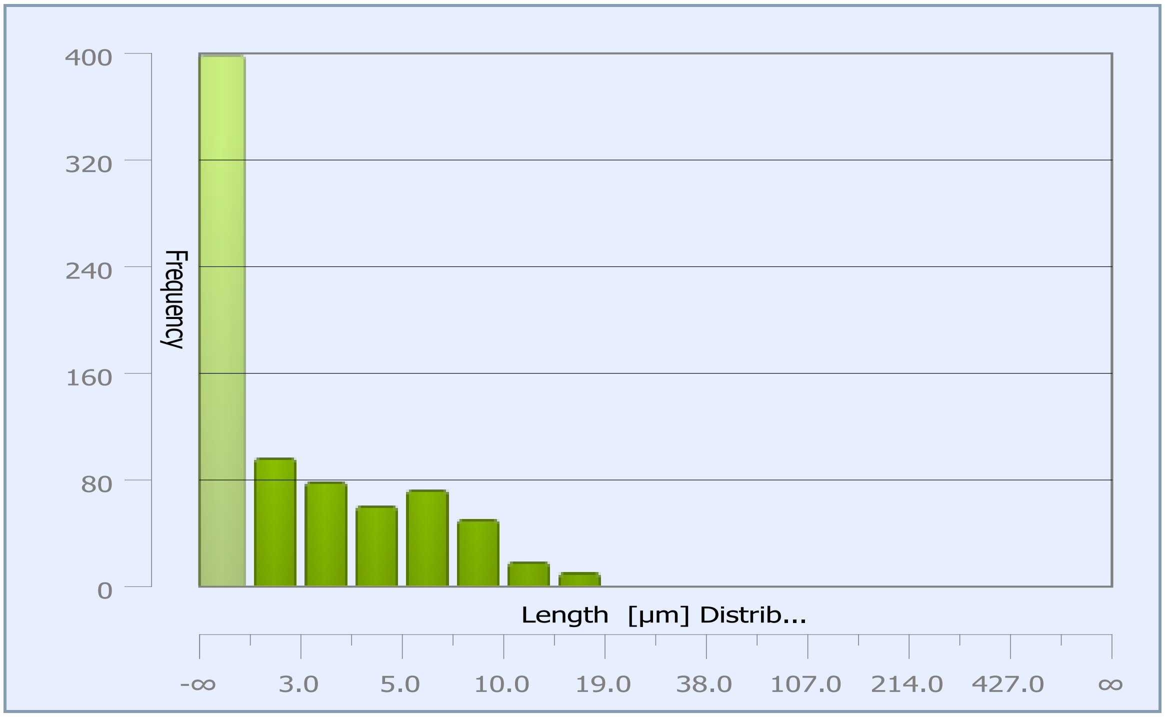

Start	End	Absolute Frequency	Absolute Frequency (accumulated)	Relative Frequency [%]	Relative Frequency (accumulated) [%]
	2.0 µm	398	398	51	51
2.0 µm	3.0 µm	97	495	12	63
3.0 µm	4.0 µm	79	574	10	73
4.0 µm	5.0 µm	60	634	8	81
5.0 µm	7.0 µm	72	706	9	90
7.0 µm	10.0 µm	50	756	6	96
10.0 µm	13.0 µm	18	774	2	99
13.0 µm	19.0 µm	10	784	1	100
19.0 µm	27.0 µm	1	785	0	100
27.0 µm	38.0 µm	0	785	0	100
38.0 µm	75.0 µm	0	785	0	100
75.0 µm	107.0 µm	0	785	0	100
107.0 µm	151.0 µm	0	785	0	100

151.0 µm	214.0 µm	0	785	0	100
214.0 µm	302.0 µm	0	785	0	100
302.0 µm	427.0 µm	0	785	0	100
427.0 µm	600.0 µm	0	785	0	100
600.0 µm		0	785	0	100
