# Supplementary material for: Benchmark dataset of the effect of grain size on strength in the single-phase FCC CrCoNi medium entropy alloy
Source: Data Brief. 2019 Oct 1;27:104592. doi: 10.1016/j.dib.2019.104592 (PMC6812030; doi:10.1016/j.dib.2019.104592)
Supplement: Multimedia component 1 [file mmc1.zip › CrCoNi_1173K_20min/CrCoNi_1173K_20min_d=5.4μm.pdf]

Analysebericht

Aug 23, 2017 12:48:14 PM

powered by imagic.ch

1. cumulative Result 1

Number of images	4
Grain size (ASTM)	11.8
Grain size (G643)	11.7
Grain stretching	95.2 %
Mean chord length	5.4 µm

2. Single Result 1 (CrCoNi Twins grain size_ASTM 900C 20min_00109)

Mean chord length	5.7 µm
Grain size (ASTM)	11.6
Grain size (G643)	11.6
Grain stretching	89.3 %

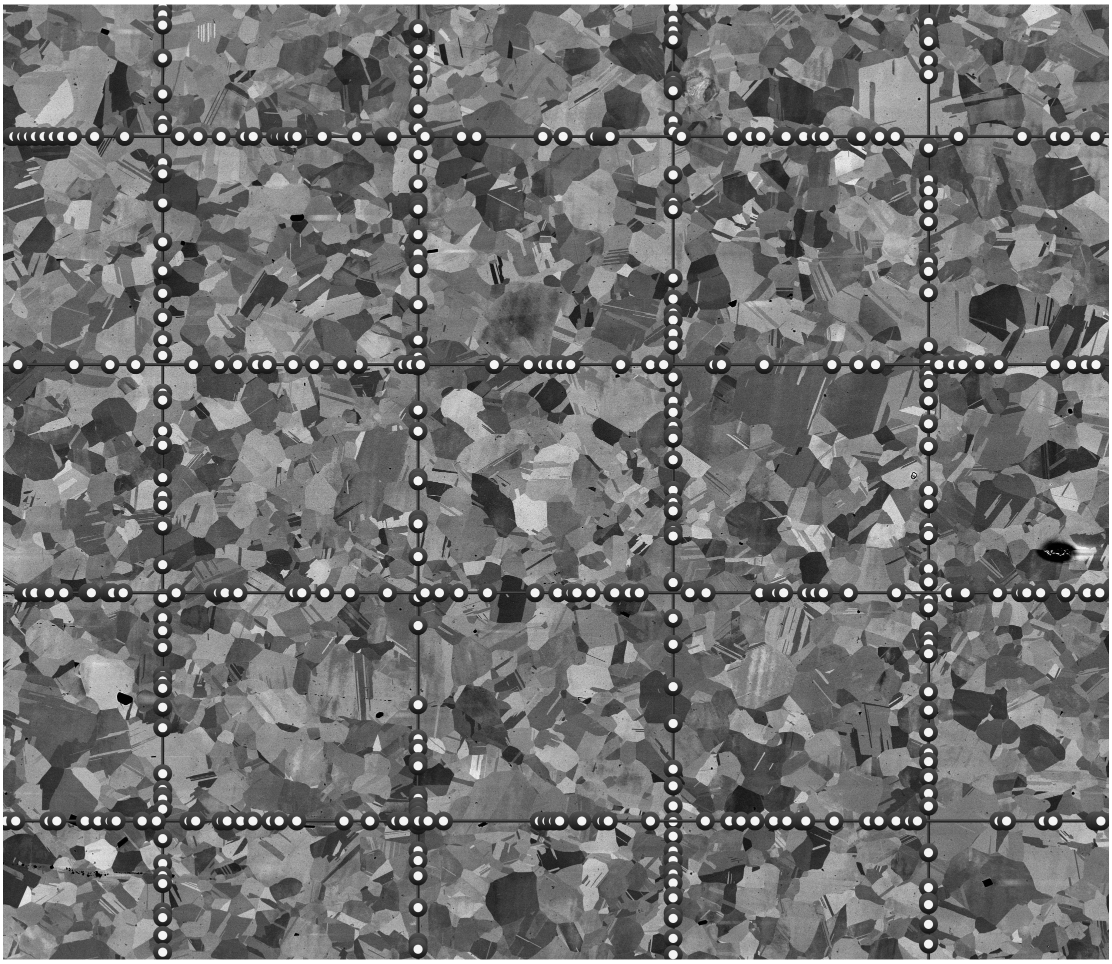

2.1. Statistical Analysis

Statistical Data	Length
Object Count	411
Minimum	0.2 µm
Maximum	27.5 µm
Average	5.7 µm
Standard deviation	4.5 µm
Skewness	0.0
Standard deviation (n-1)	4.5 µm
Variance	20.4 µm²
Variance (n-1)	20.5 µm²
Sum	2'362.5 µm

Statistical Data	Length
Sum of squares	21'974.2 µm²
Sum of cubes	279'717.2 µm³

2.1.1. Chord Lenght Distribution

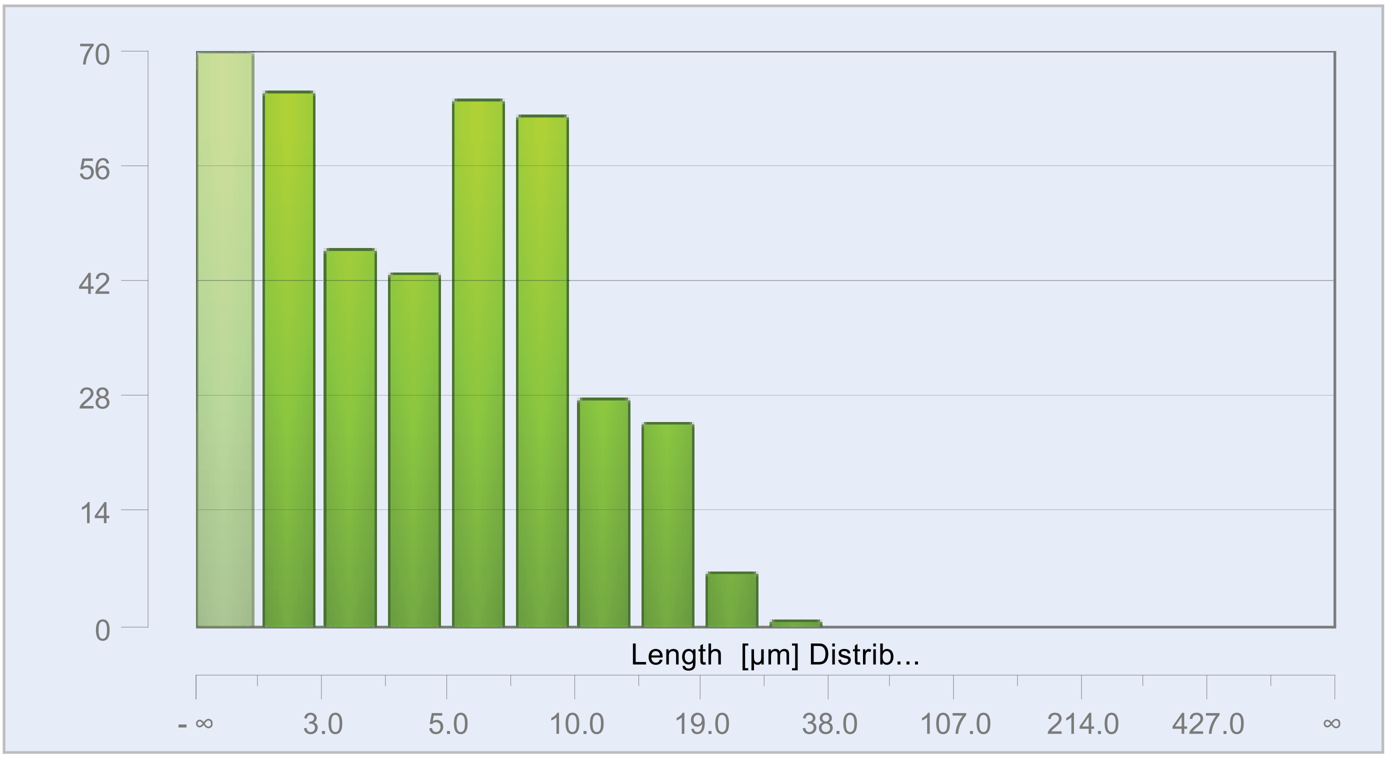

Start	End	Absolute Frequency	Absolute Frequency (accumulated)	Relative Frequency [%]	Relative Frequency (accumulated) [%]
	2.0 µm	70	70	17	17
2.0 µm	3.0 µm	65	135	16	33
3.0 µm	4.0 µm	46	181	11	44
4.0 µm	5.0 µm	43	224	10	55
5.0 µm	7.0 µm	64	288	16	70
7.0 µm	10.0 µm	62	350	15	85
10.0 µm	13.0 µm	28	378	7	92
13.0 µm	19.0 µm	25	403	6	98
19.0 µm	27.0 µm	7	410	2	100
27.0 µm	38.0 µm	1	411	0	100
38.0 µm	75.0 µm	0	411	0	100
75.0 µm	107.0 µm	0	411	0	100
107.0 µm	151.0 µm	0	411	0	100
151.0 µm	214.0 µm	0	411	0	100
214.0 µm	302.0 µm	0	411	0	100
302.0 µm	427.0 µm	0	411	0	100
427.0 µm	600.0 µm	0	411	0	100
600.0 µm		0	411	0	100

3. Single Result 2 (CrCoNi Twins grain size_ASTM 900C 20min_00110)

Mean chord length	5.8 µm
Grain size (ASTM)	11.6
Grain size (G643)	11.5
Grain stretching	94.3 %

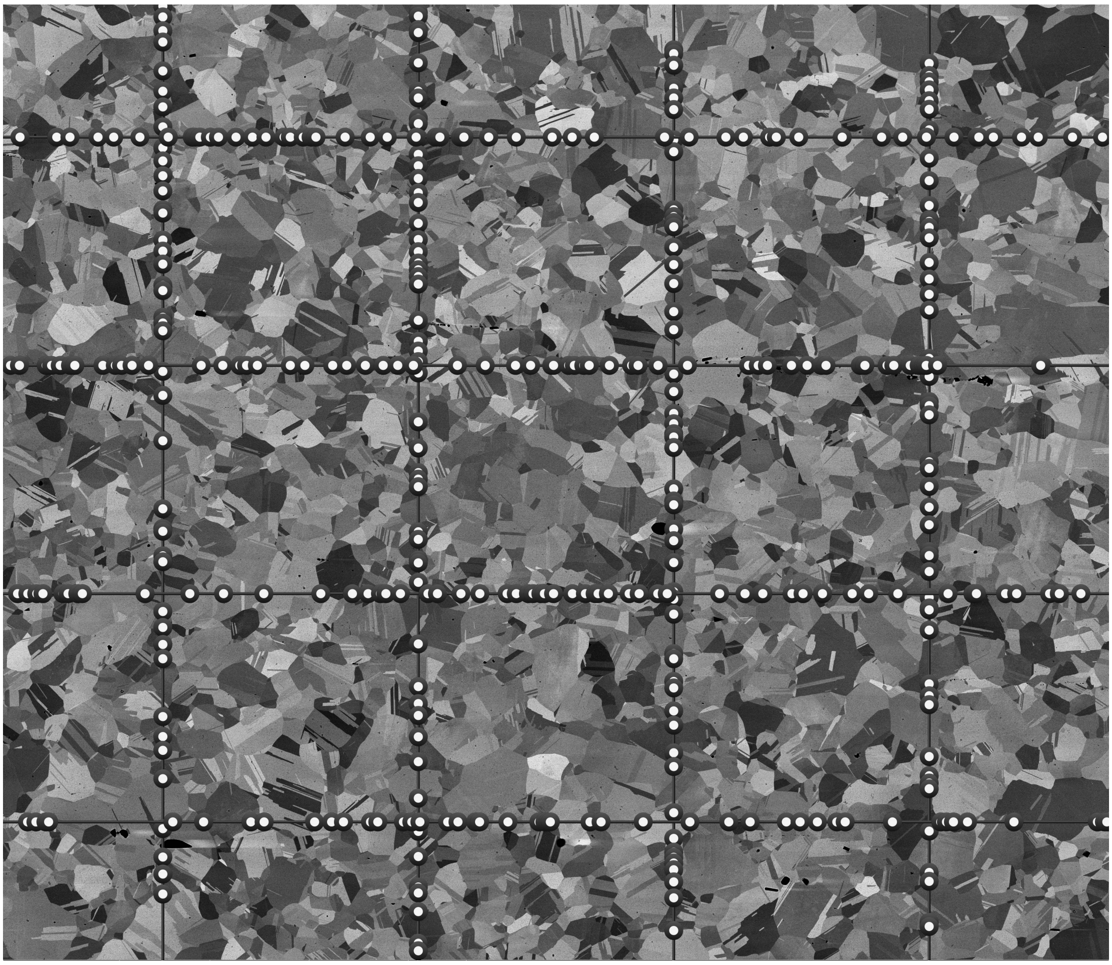

3.1. Statistical Analysis

Statistical Data	Length
Object Count	410
Minimum	0.5 µm
Maximum	35.6 µm
Average	5.8 µm
Standard deviation	4.5 µm
Skewness	0.0
Standard deviation (n-1)	4.5 µm
Variance	20.4 µm²
Variance (n-1)	20.4 µm²
Sum	2'363.2 µm
Sum of squares	21'972.3 µm²
Sum of cubes	297'064.4 µm³

3.1.1. Chord Lenght Distribution

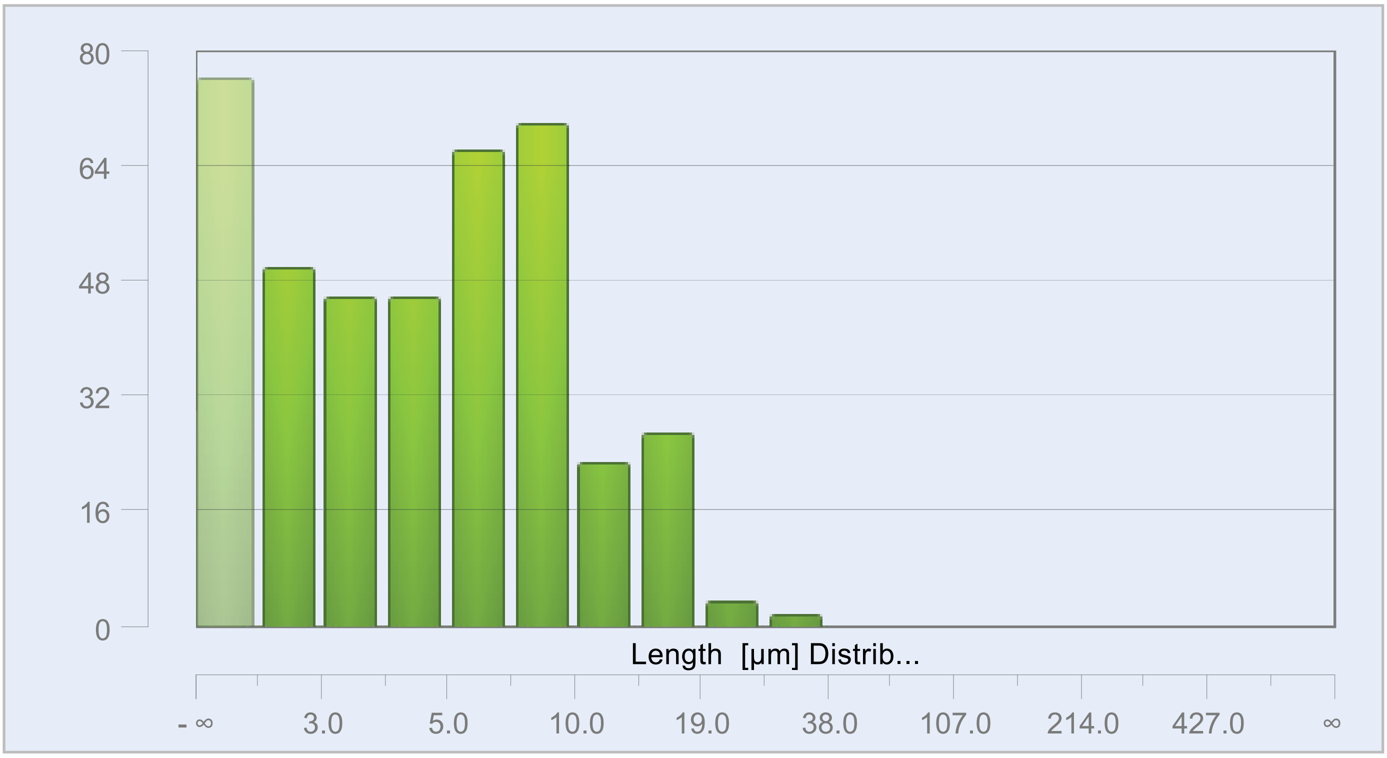

Start	End	Absolute Frequency	Absolute Frequency (accumulated)	Relative Frequency [%]	Relative Frequency (accumulated) [%]
	2.0 µm	76	76	19	19
2.0 µm	3.0 µm	50	126	12	31
3.0 µm	4.0 µm	46	172	11	42
4.0 µm	5.0 µm	46	218	11	53
5.0 µm	7.0 µm	66	284	16	69
7.0 µm	10.0 µm	70	354	17	86
10.0 µm	13.0 µm	23	377	6	92
13.0 µm	19.0 µm	27	404	7	99
19.0 µm	27.0 µm	4	408	1	100
27.0 µm	38.0 µm	2	410	0	100
38.0 µm	75.0 µm	0	410	0	100
75.0 µm	107.0 µm	0	410	0	100
107.0 µm	151.0 µm	0	410	0	100
151.0 µm	214.0 µm	0	410	0	100
214.0 µm	302.0 µm	0	410	0	100
302.0 µm	427.0 µm	0	410	0	100
427.0 µm	600.0 µm	0	410	0	100
600.0 µm		0	410	0	100

4. Single Result 3 (CrCoNi Twins grain size_ASTM 900C 20min_00111)

Mean chord length	5.1 µm
Grain size (ASTM)	11.9
Grain size (G643)	11.9
Grain stretching	94.6 %

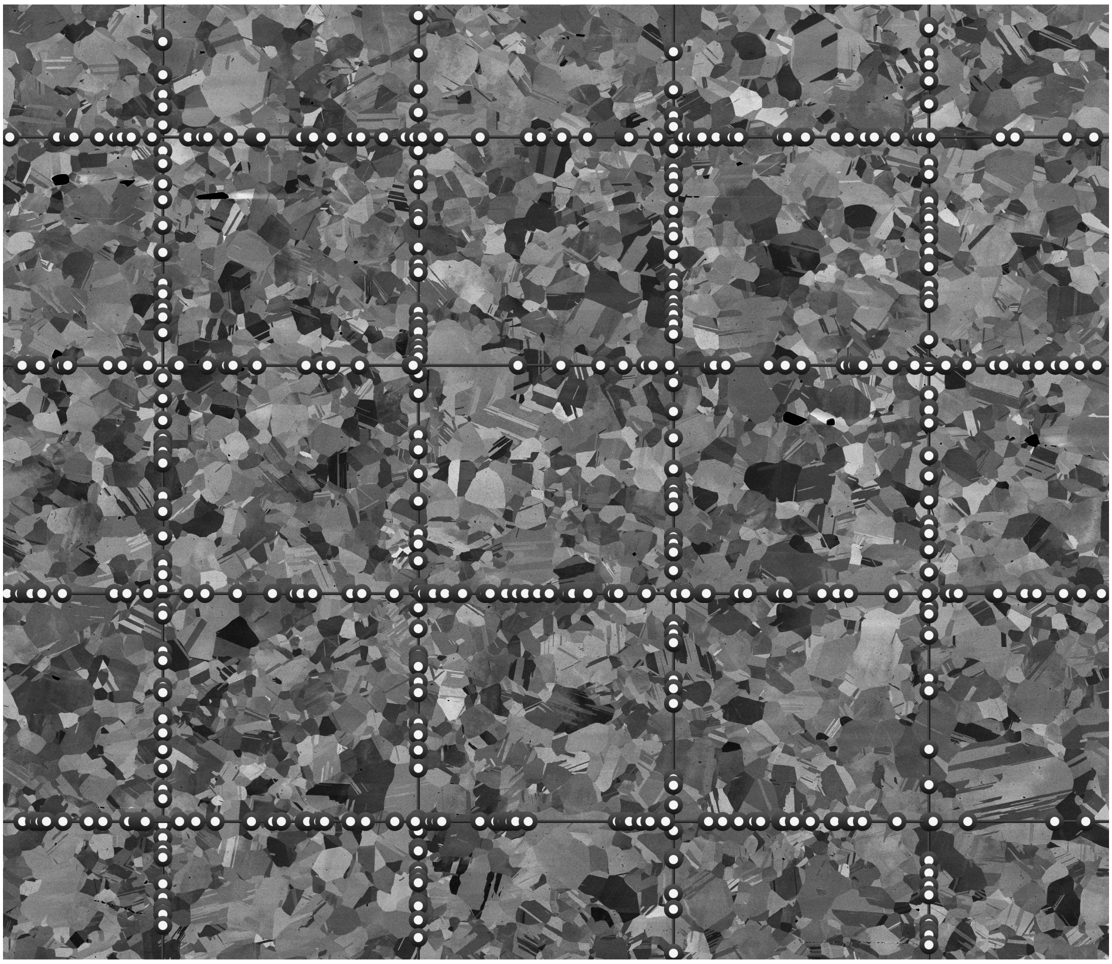

4.1. Statistical Analysis

Statistical Data	Length
Object Count	463
Minimum	0.5 μm
Maximum	30.0 μm
Average	5.1 μm
Standard deviation	3.9 μm
Skewness	0.0
Standard deviation (n-1)	3.9 μm
Variance	15.0 μm²
Variance (n-1)	15.1 μm²
Sum	2'362.8 μm
Sum of squares	19'024.7 μm²
Sum of cubes	219'581.5 μm³

4.1.1. Chord Lenght Distribution

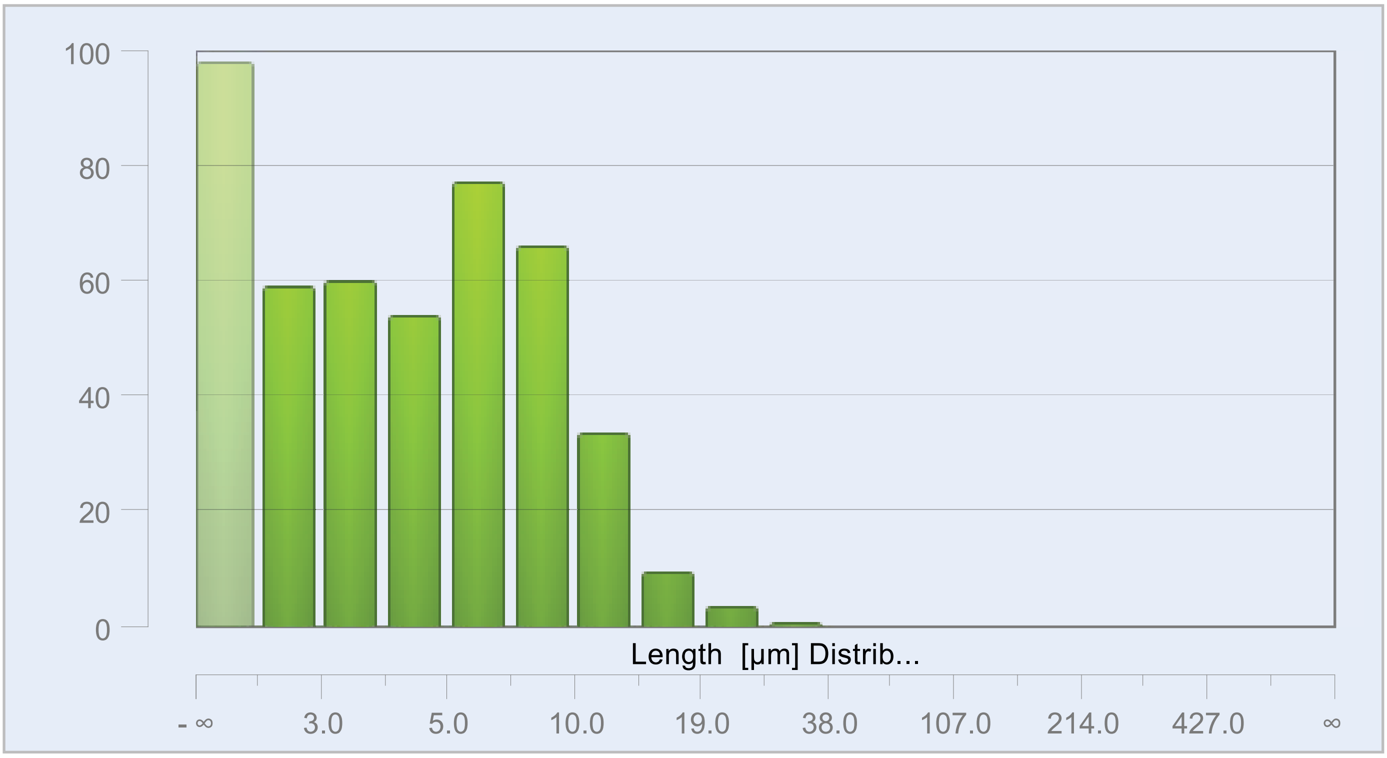

Start	End	Absolute Frequency	Absolute Frequency (accumulated)	Relative Frequency [%]	Relative Frequency (accumulated) [%]
	2.0 µm	98	98	21	21
2.0 µm	3.0 µm	59	157	13	34
3.0 µm	4.0 µm	60	217	13	47
4.0 µm	5.0 µm	54	271	12	59
5.0 µm	7.0 µm	77	348	17	75
7.0 µm	10.0 µm	66	414	14	89
10.0 µm	13.0 µm	34	448	7	97
13.0 µm	19.0 µm	10	458	2	99
19.0 µm	27.0 µm	4	462	1	100
27.0 µm	38.0 µm	1	463	0	100
38.0 µm	75.0 µm	0	463	0	100
75.0 µm	107.0 µm	0	463	0	100
107.0 µm	151.0 µm	0	463	0	100
151.0 µm	214.0 µm	0	463	0	100
214.0 µm	302.0 µm	0	463	0	100
302.0 µm	427.0 µm	0	463	0	100
427.0 µm	600.0 µm	0	463	0	100
600.0 µm		0	463	0	100

5. Single Result 4 (CrCoNi Twins grain size_ASTM 900C 20min_00113)

Mean chord length	5.1 µm
Grain size (ASTM)	12
Grain size (G643)	11.9
Grain stretching	97.9 %

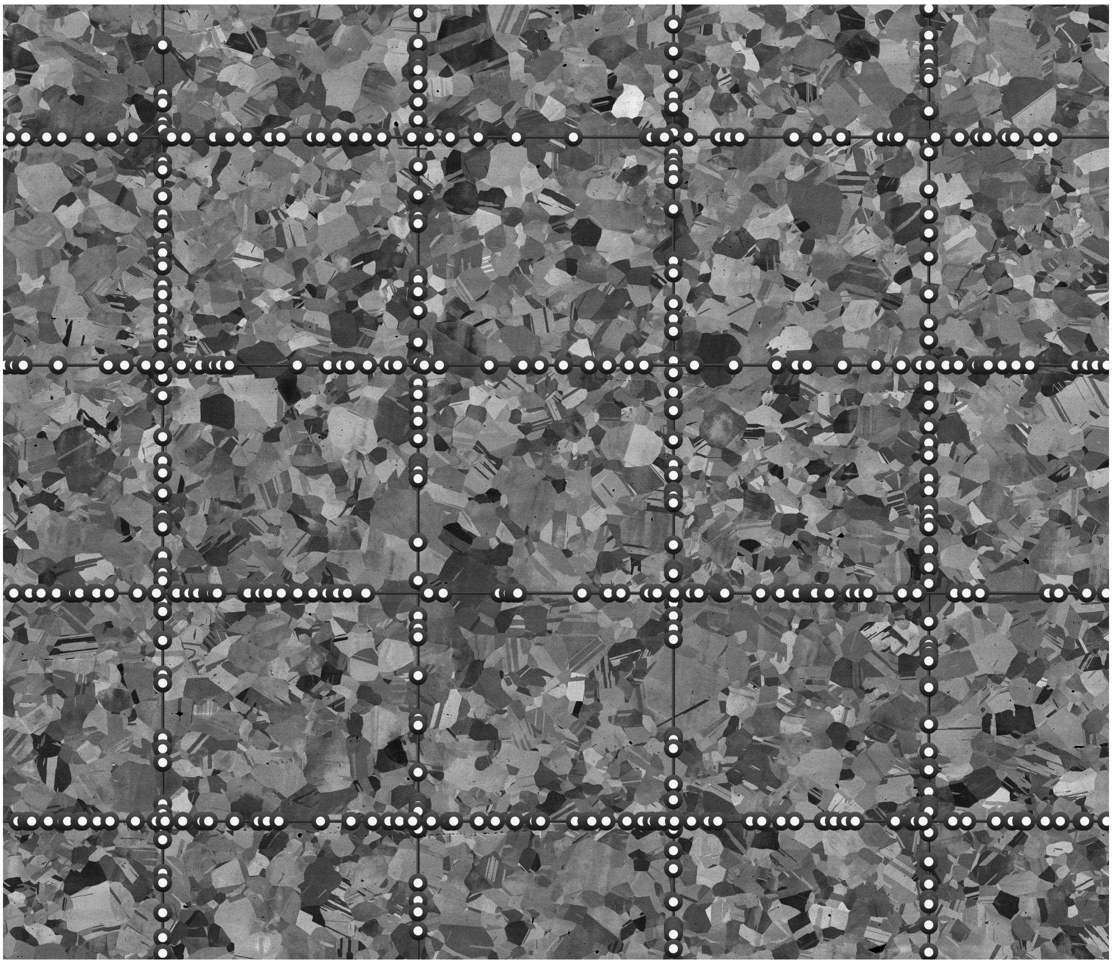

5.1. Statistical Analysis

Statistical Data	Length
Object Count	465
Minimum	0.4 µm
Maximum	27.7 µm
Average	5.1 µm
Standard deviation	3.8 µm
Skewness	0.0
Standard deviation (n-1)	3.8 µm
Variance	14.3 µm²
Variance (n-1)	14.4 µm²
Sum	2'362.8 µm
Sum of squares	18'676.1 µm²
Sum of cubes	206'072.0 µm³

5.1.1. Chord Lenght Distribution

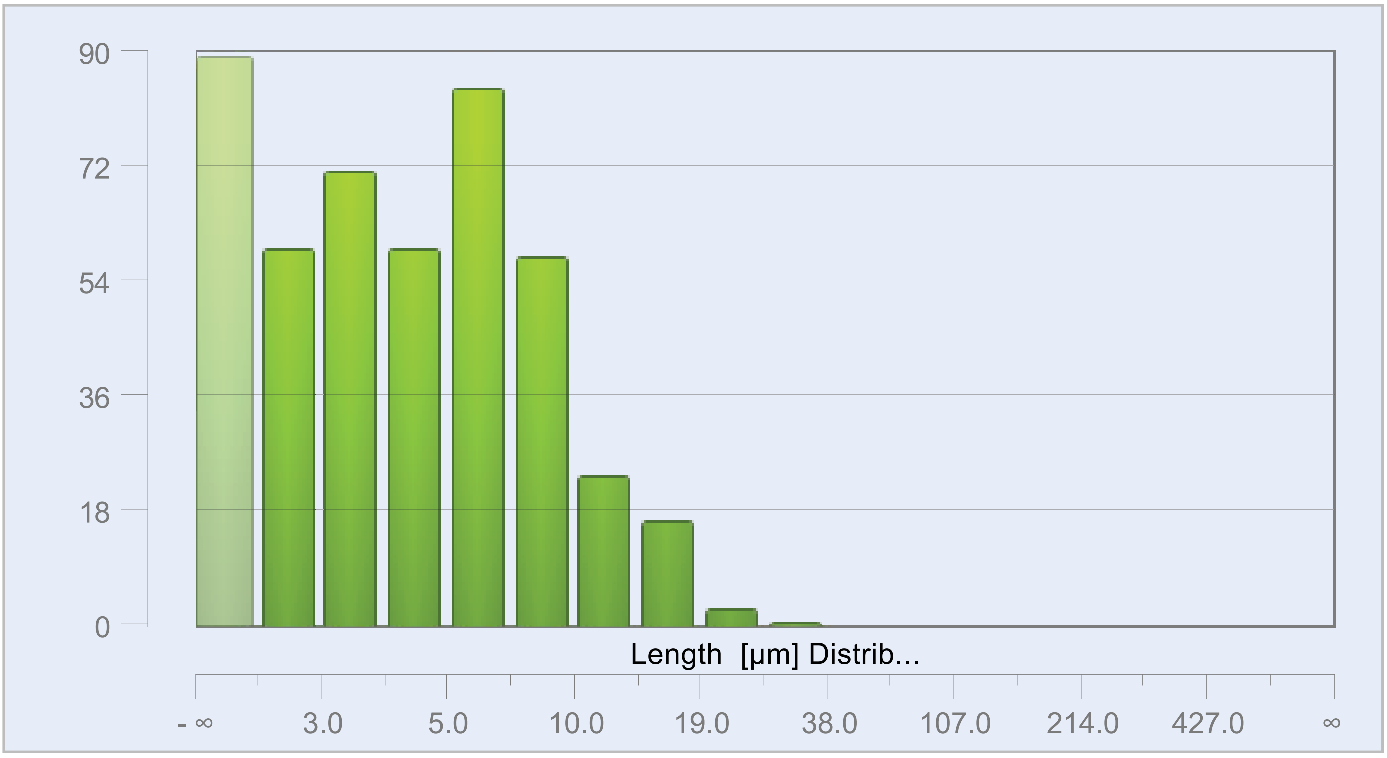

Start	End	Absolute Frequency	Absolute Frequency (accumulated)	Relative Frequency [%]	Relative Frequency (accumulated) [%]
	2.0 µm	89	89	19	19
2.0 µm	3.0 µm	59	148	13	32
3.0 µm	4.0 µm	71	219	15	47
4.0 µm	5.0 µm	59	278	13	60
5.0 µm	7.0 µm	84	362	18	78
7.0 µm	10.0 µm	58	420	12	90
10.0 µm	13.0 µm	24	444	5	95
13.0 µm	19.0 µm	17	461	4	99
19.0 µm	27.0 µm	3	464	1	100
27.0 µm	38.0 µm	1	465	0	100
38.0 µm	75.0 µm	0	465	0	100
75.0 µm	107.0 µm	0	465	0	100
107.0 µm	151.0 µm	0	465	0	100
151.0 µm	214.0 µm	0	465	0	100
214.0 µm	302.0 µm	0	465	0	100
302.0 µm	427.0 µm	0	465	0	100
427.0 µm	600.0 µm	0	465	0	100
600.0 µm		0	465	0	100
