# Supplementary material for: Benchmark dataset of the effect of grain size on strength in the single-phase FCC CrCoNi medium entropy alloy
Source: Data Brief. 2019 Oct 1;27:104592. doi: 10.1016/j.dib.2019.104592 (PMC6812030; doi:10.1016/j.dib.2019.104592)
Supplement: Multimedia component 1 [file mmc1.zip › CrCoNi_1173K_30min/CrCoNi_1173K_30min_c=3.2μm.pdf]

Analysebericht

08.11.2017 14:11:41

powered by imagic.ch

1. cumulative Result 1

Anzahl Bilder	4
Korngröße (ASTM)	13,4
Korngröße (G643)	13,3
Kornstreckung	87,3 %
Mittlere Sehnenlänge	3,1 µm

2. Single Result 1 (CrCoNi - ASTM E 112_CrCoNi_homogenized_8.1mmSW_900°C_30min_00170)

Mittlere Sehnenlänge	3,4 µm
Korngröße (ASTM)	13,1
Korngröße (G643)	13,1
Kornstreckung	86 %

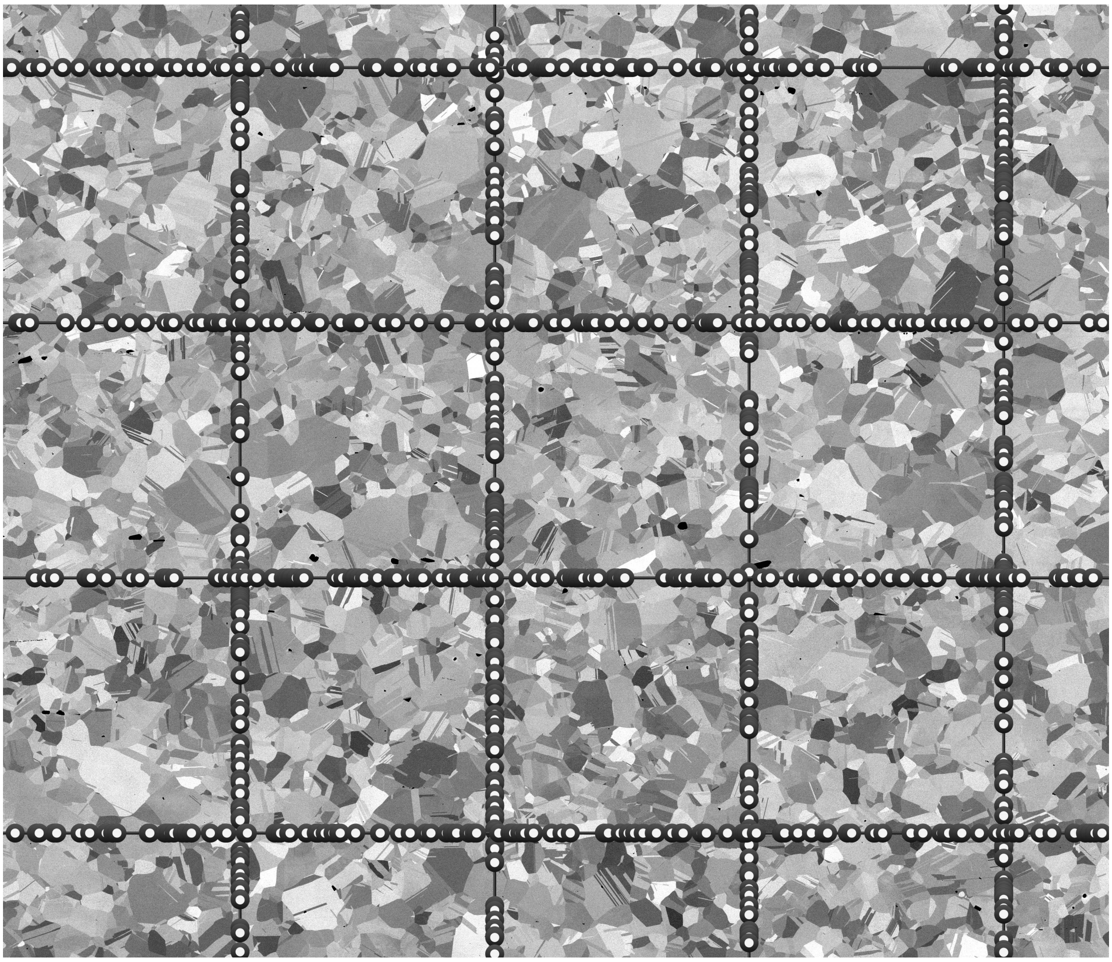

2.1. Statistische Analyse

Statistische Daten	Länge
Anzahl Objekte	923
Minimum	0,2 µm
Maximum	23,1 µm
Mittelwert	3,4 µm
Standardabweichung	3,3 µm
Schiefe	0,0
Standardabweichung (n-1)	3,3 µm
Varianz	10,6 µm²
Varianz (n-1)	10,6 µm²
Summe	3'148,4 µm

Statistische Daten	Länge
Quadratsumme	20'526,2 µm²
Kubiksumme	189'116,4 µm³

2.1.1. Chord Lenght Distribution

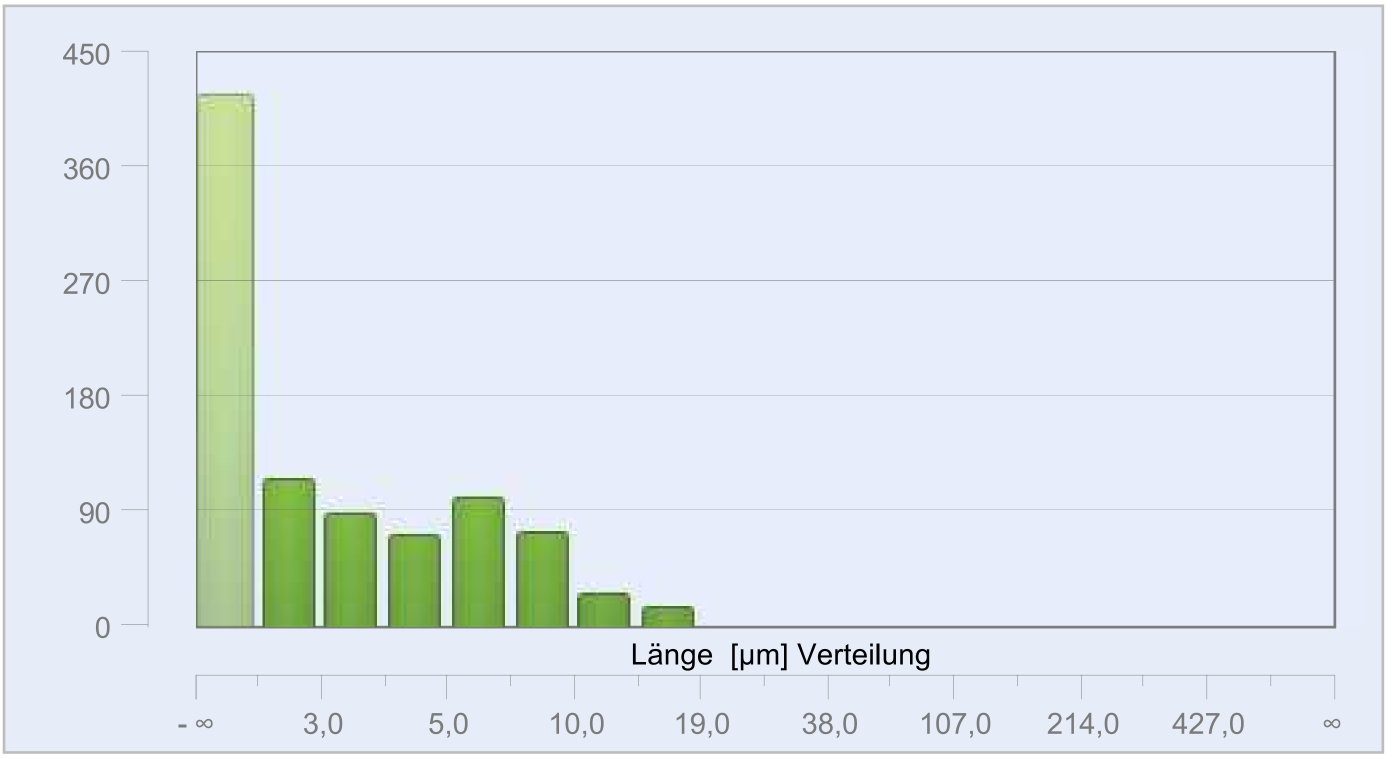

Start	Ende	Absolute Häufigkeit	Absolute Häufigkeit (kumuliert)	Relative Häufigkeit [%]	Relative Häufigkeit (kumuliert) [%]
	2,0 µm	416	416	45	45
2,0 µm	3,0 µm	118	534	13	58
3,0 µm	4,0 µm	91	625	10	68
4,0 µm	5,0 µm	74	699	8	76
5,0 µm	7,0 µm	103	802	11	87
7,0 µm	10,0 µm	75	877	8	95
10,0 µm	13,0 µm	27	904	3	98
13,0 µm	19,0 µm	18	922	2	100
19,0 µm	27,0 µm	1	923	0	100
27,0 µm	38,0 µm	0	923	0	100
38,0 µm	75,0 µm	0	923	0	100
75,0 µm	107,0 µm	0	923	0	100
107,0 µm	151,0 µm	0	923	0	100
151,0 µm	214,0 µm	0	923	0	100
214,0 µm	302,0 µm	0	923	0	100
302,0 µm	427,0 µm	0	923	0	100
427,0 µm	600,0 µm	0	923	0	100
600,0 µm		0	923	0	100

3. Single Result 2 (CrCoNi - ASTM E 112_CrCoNi_homogenized_8.1mmSW_900°C_30min_00171)

Mittlere Sehnenlänge	2,9 µm
Korngröße (ASTM)	13,6
Korngröße (G643)	13,6
Kornstreckung	86,1 %

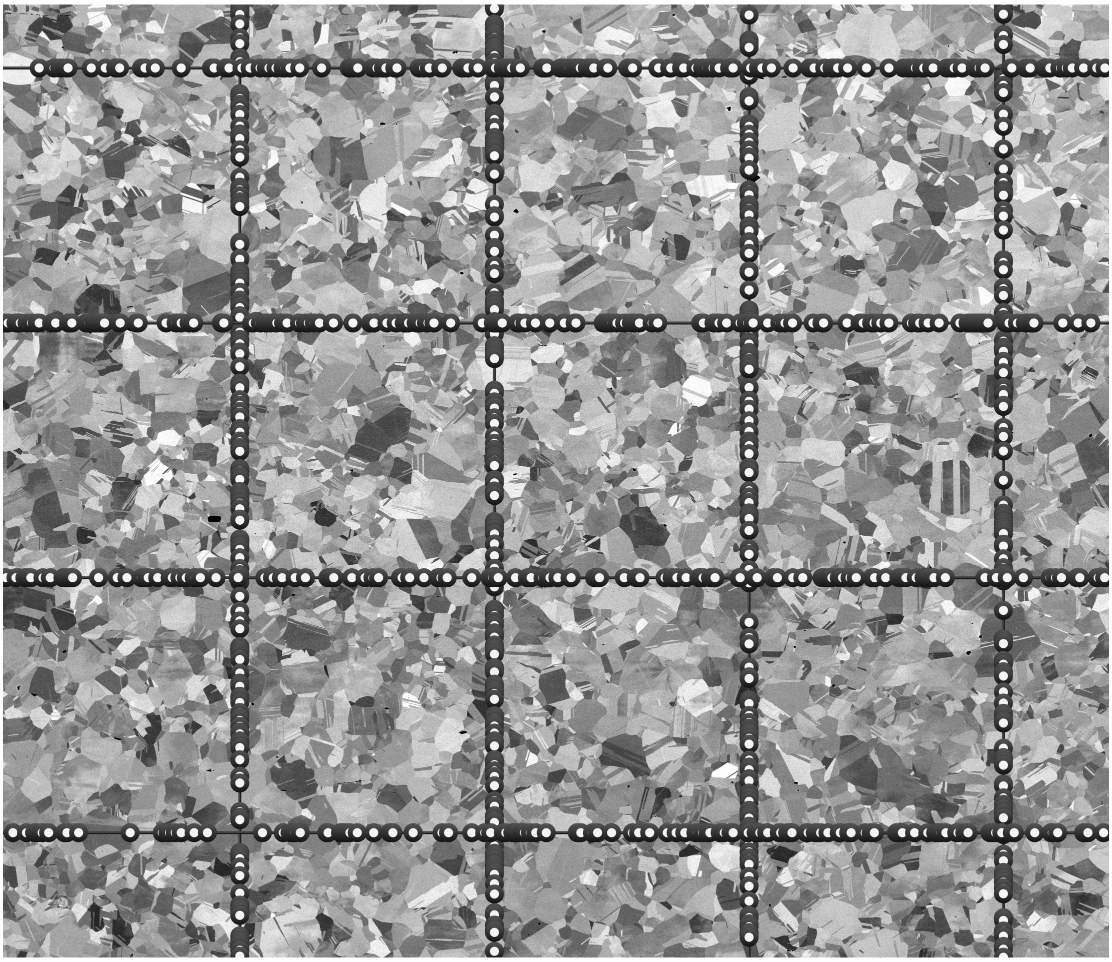

3.1. Statistische Analyse

Statistische Daten	Länge
Anzahl Objekte	1100
Minimum	0,2 µm
Maximum	21,1 µm
Mittelwert	2,9 µm
Standardabweichung	2,8 µm
Schiefe	0,0
Standardabweichung (n-1)	2,8 µm
Varianz	7,9 µm²
Varianz (n-1)	7,9 µm²
Summe	3'148,0 µm
Quadratsumme	17'662,1 µm²
Kubiksumme	144'236,1 µm³

3.1.1. Chord Lenght Distribution

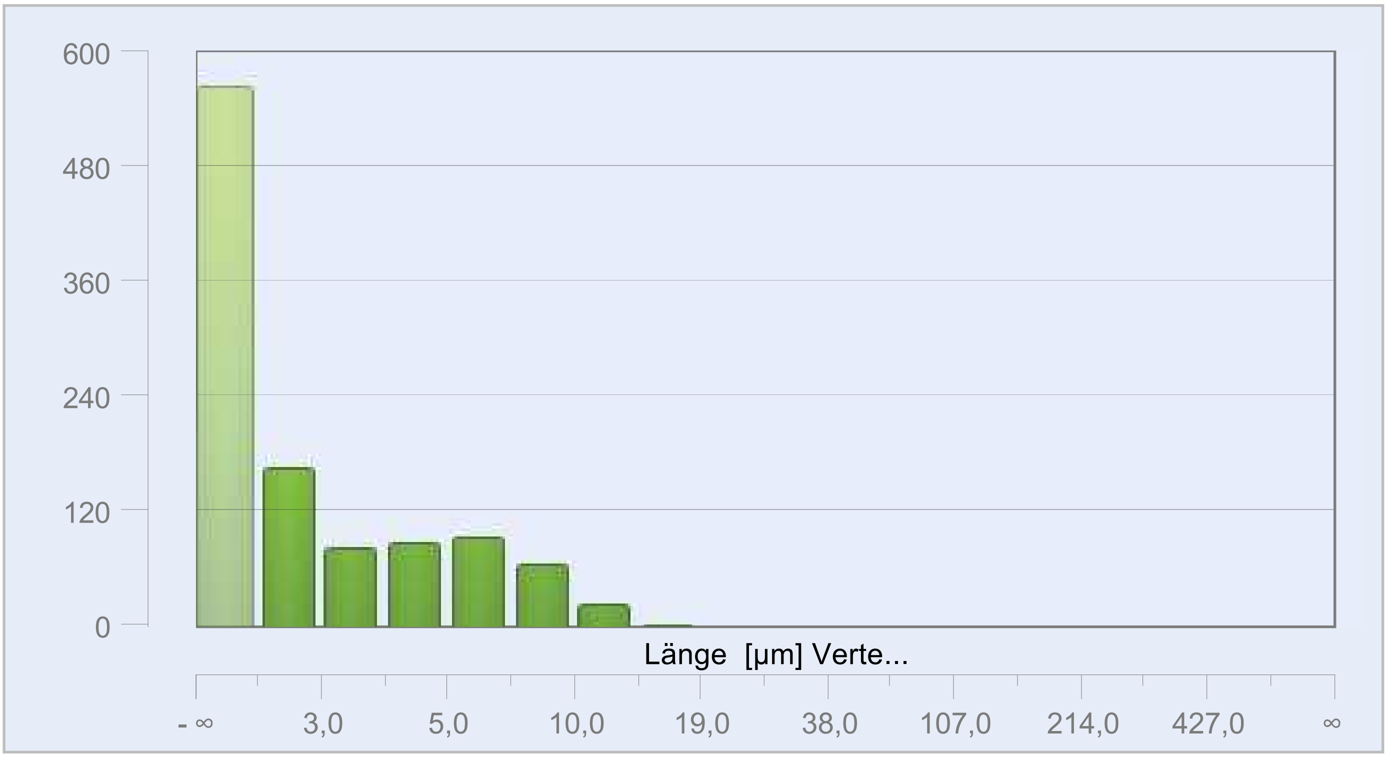

Start	Ende	Absolute Häufigkeit	Absolute Häufigkeit (kumuliert)	Relative Häufigkeit [%]	Relative Häufigkeit (kumuliert) [%]
	2,0 µm	564	564	51	51
2,0 µm	3,0 µm	166	730	15	66
3,0 µm	4,0 µm	84	814	8	74
4,0 µm	5,0 µm	91	905	8	82
5,0 µm	7,0 µm	96	1001	9	91
7,0 µm	10,0 µm	67	1068	6	97
10,0 µm	13,0 µm	25	1093	2	99
13,0 µm	19,0 µm	5	1098	0	100
19,0 µm	27,0 µm	2	1100	0	100
27,0 µm	38,0 µm	0	1100	0	100
38,0 µm	75,0 µm	0	1100	0	100
75,0 µm	107,0 µm	0	1100	0	100
107,0 µm	151,0 µm	0	1100	0	100
151,0 µm	214,0 µm	0	1100	0	100
214,0 µm	302,0 µm	0	1100	0	100
302,0 µm	427,0 µm	0	1100	0	100
427,0 µm	600,0 µm	0	1100	0	100
600,0 µm		0	1100	0	100

4. Single Result 3 (CrCoNi - ASTM E 112_CrCoNi_homogenized_8.1mmSW_900°C_30min_00172)

Mittlere Sehnenlänge	3 µm
Korngröße (ASTM)	13,5
Korngröße (G643)	13,5
Kornstreckung	89,5 %

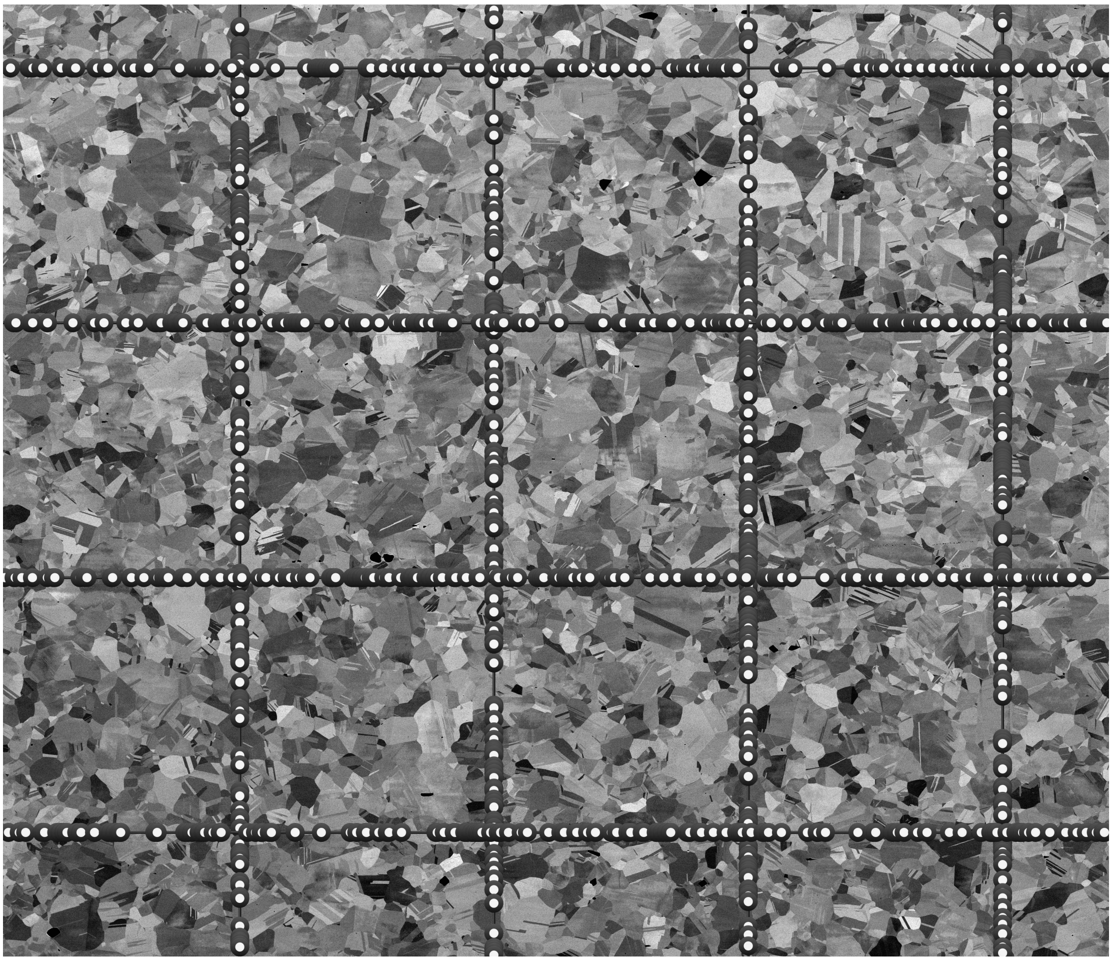

4.1. Statistische Analyse

Statistische Daten	Länge
Anzahl Objekte	1059
Minimum	0,2 µm
Maximum	17,1 µm
Mittelwert	3,0 µm
Standardabweichung	2,9 µm
Schiefe	0,0
Standardabweichung (n-1)	2,9 µm
Varianz	8,4 µm²
Varianz (n-1)	8,4 µm²
Summe	3'148,3 µm
Quadratsumme	18'273,9 µm²
Kubiksumme	151'560,1 µm³

4.1.1. Chord Lenght Distribution

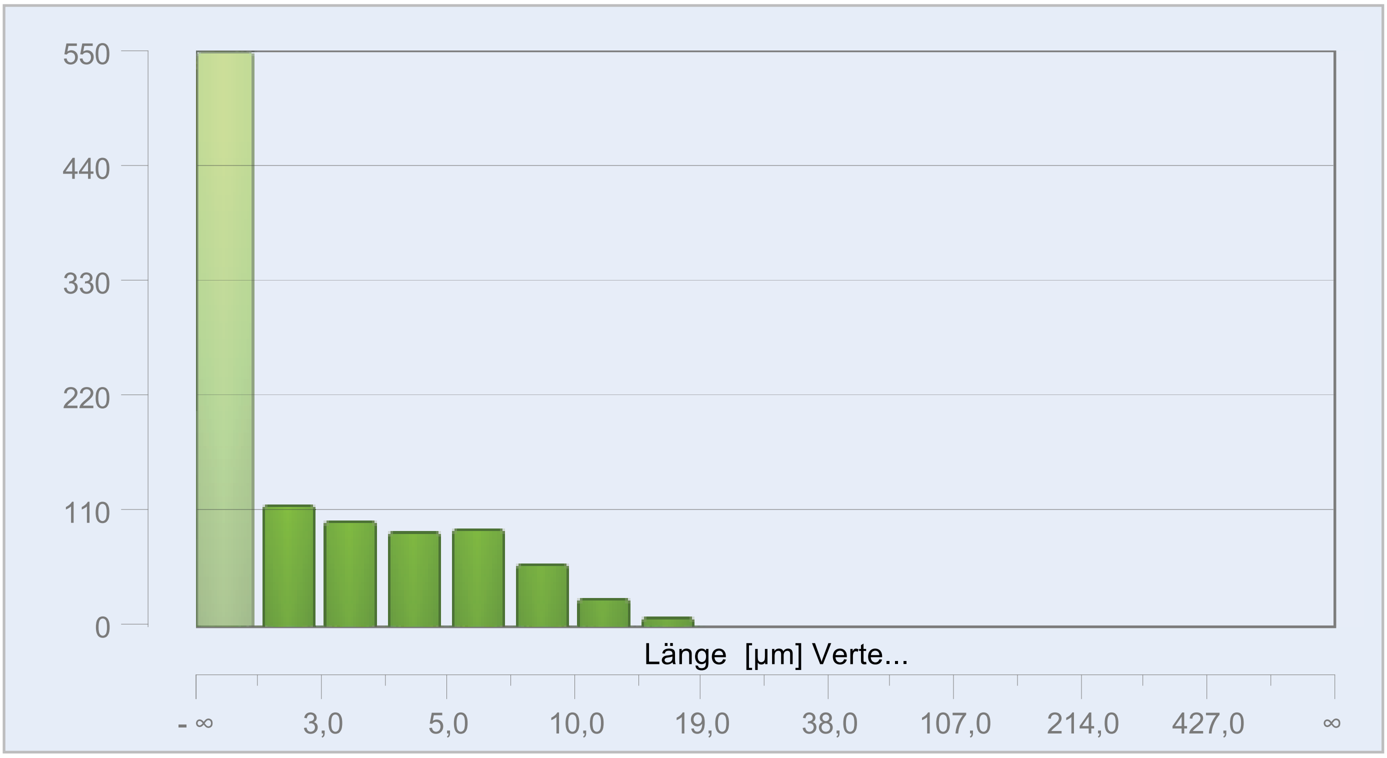

Start	Ende	Absolute Häufigkeit	Absolute Häufigkeit (kumuliert)	Relative Häufigkeit [%]	Relative Häufigkeit (kumuliert) [%]
	2,0 µm	549	549	52	52
2,0 µm	3,0 µm	117	666	11	63
3,0 µm	4,0 µm	103	769	10	73
4,0 µm	5,0 µm	93	862	9	81
5,0 µm	7,0 µm	94	956	9	90
7,0 µm	10,0 µm	63	1019	6	96
10,0 µm	13,0 µm	28	1047	3	99
13,0 µm	19,0 µm	12	1059	1	100
19,0 µm	27,0 µm	0	1059	0	100
27,0 µm	38,0 µm	0	1059	0	100
38,0 µm	75,0 µm	0	1059	0	100
75,0 µm	107,0 µm	0	1059	0	100
107,0 µm	151,0 µm	0	1059	0	100
151,0 µm	214,0 µm	0	1059	0	100
214,0 µm	302,0 µm	0	1059	0	100
302,0 µm	427,0 µm	0	1059	0	100
427,0 µm	600,0 µm	0	1059	0	100
600,0 µm		0	1059	0	100

5. Single Result 4 (CrCoNi - ASTM E 112_CrCoNi_homogenized_8.1mmSW_900°C_30min_00173)

Mittlere Sehnenlänge	3,3 µm
Korngröße (ASTM)	13,2
Korngröße (G643)	13,2
Kornstreckung	87,6 %

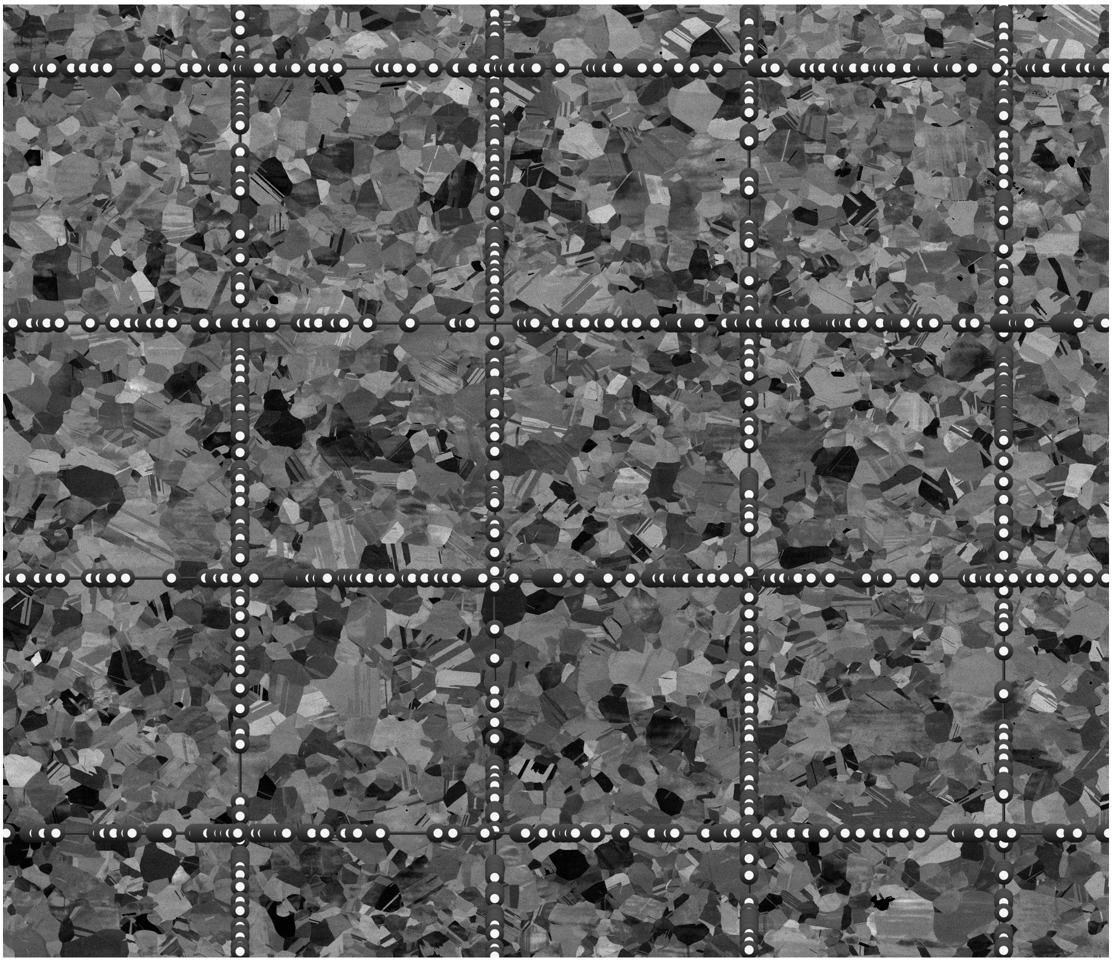

5.1. Statistische Analyse

Statistische Daten	Länge
Anzahl Objekte	968
Minimum	0,1 µm
Maximum	22,0 µm
Mittelwert	3,3 µm
Standardabweichung	3,3 µm
Schiefe	0,0
Standardabweichung (n-1)	3,3 µm
Varianz	10,8 µm²
Varianz (n-1)	10,8 µm²
Summe	3'150,0 µm
Quadratsumme	20'706,5 µm²
Kubiksumme	202'118,2 µm³

5.1.1. Chord Lenght Distribution

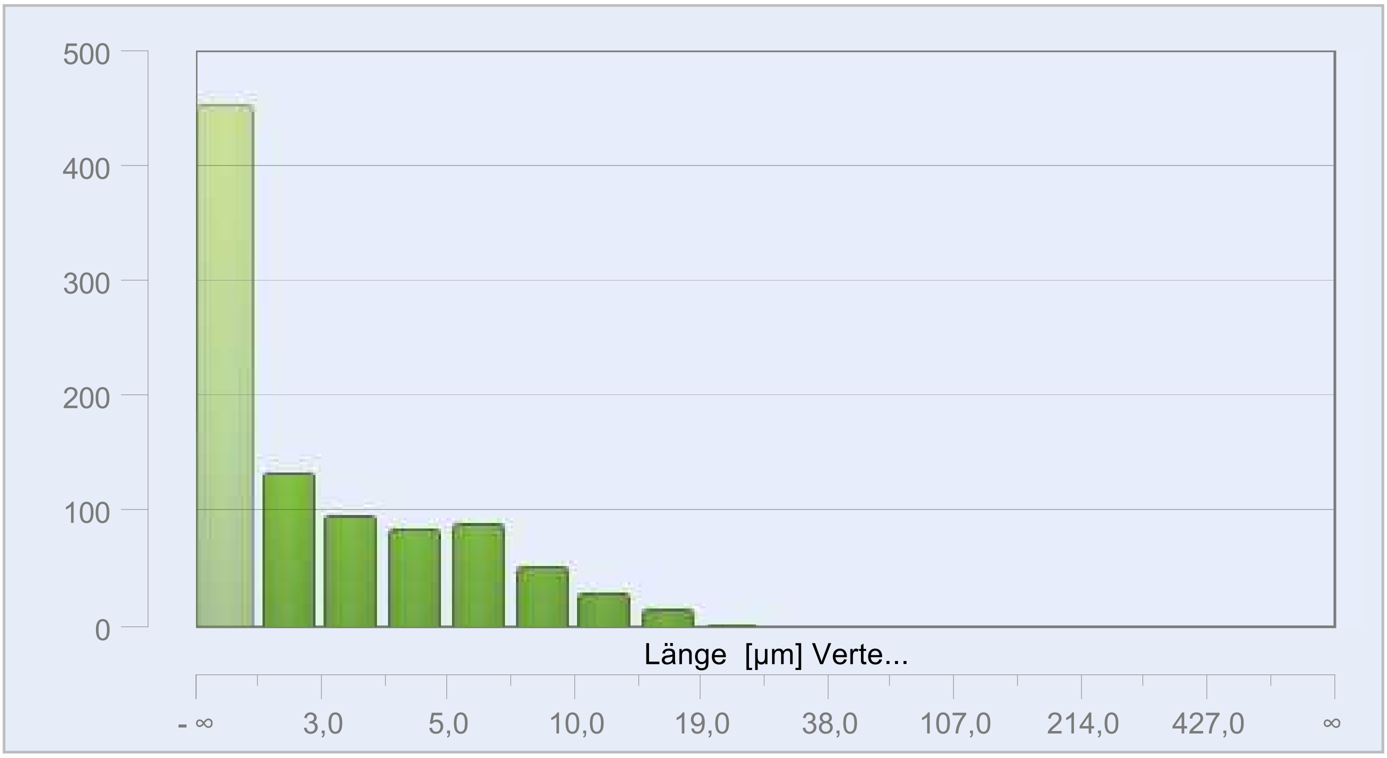

Start	Ende	Absolute Häufigkeit	Absolute Häufigkeit (kumuliert)	Relative Häufigkeit [%]	Relative Häufigkeit (kumuliert) [%]
	2,0 µm	452	452	47	47
2,0 µm	3,0 µm	134	586	14	61
3,0 µm	4,0 µm	98	684	10	71
4,0 µm	5,0 µm	86	770	9	80
5,0 µm	7,0 µm	91	861	9	89
7,0 µm	10,0 µm	55	916	6	95
10,0 µm	13,0 µm	32	948	3	98
13,0 µm	19,0 µm	17	965	2	100
19,0 µm	27,0 µm	3	968	0	100
27,0 µm	38,0 µm	0	968	0	100
38,0 µm	75,0 µm	0	968	0	100
75,0 µm	107,0 µm	0	968	0	100
107,0 µm	151,0 µm	0	968	0	100
151,0 µm	214,0 µm	0	968	0	100
214,0 µm	302,0 µm	0	968	0	100
302,0 µm	427,0 µm	0	968	0	100
427,0 µm	600,0 µm	0	968	0	100
600,0 µm		0	968	0	100
