# Supplementary material for: Benchmark dataset of the effect of grain size on strength in the single-phase FCC CrCoNi medium entropy alloy
Source: Data Brief. 2019 Oct 1;27:104592. doi: 10.1016/j.dib.2019.104592 (PMC6812030; doi:10.1016/j.dib.2019.104592)
Supplement: Multimedia component 1 [file mmc1.zip › CrCoNi_1173K_30min/CrCoNi_1173K_30min_d=7.1μm.pdf]

Analysebericht

Aug 24, 2017 3:08:15 PM

powered by imagic.ch

1. cumulative Result 1

Number of images	4
Grain size (ASTM)	11
Grain size (G643)	11
Grain stretching	96.4 %
Mean chord length	7.1 µm

2. Single Result 1 (CrCoNi Twins grain size_ASTM 900C 30min_00139)

Mean chord length	7.4 µm
Grain size (ASTM)	10.9
Grain size (G643)	10.8
Grain stretching	98.6 %

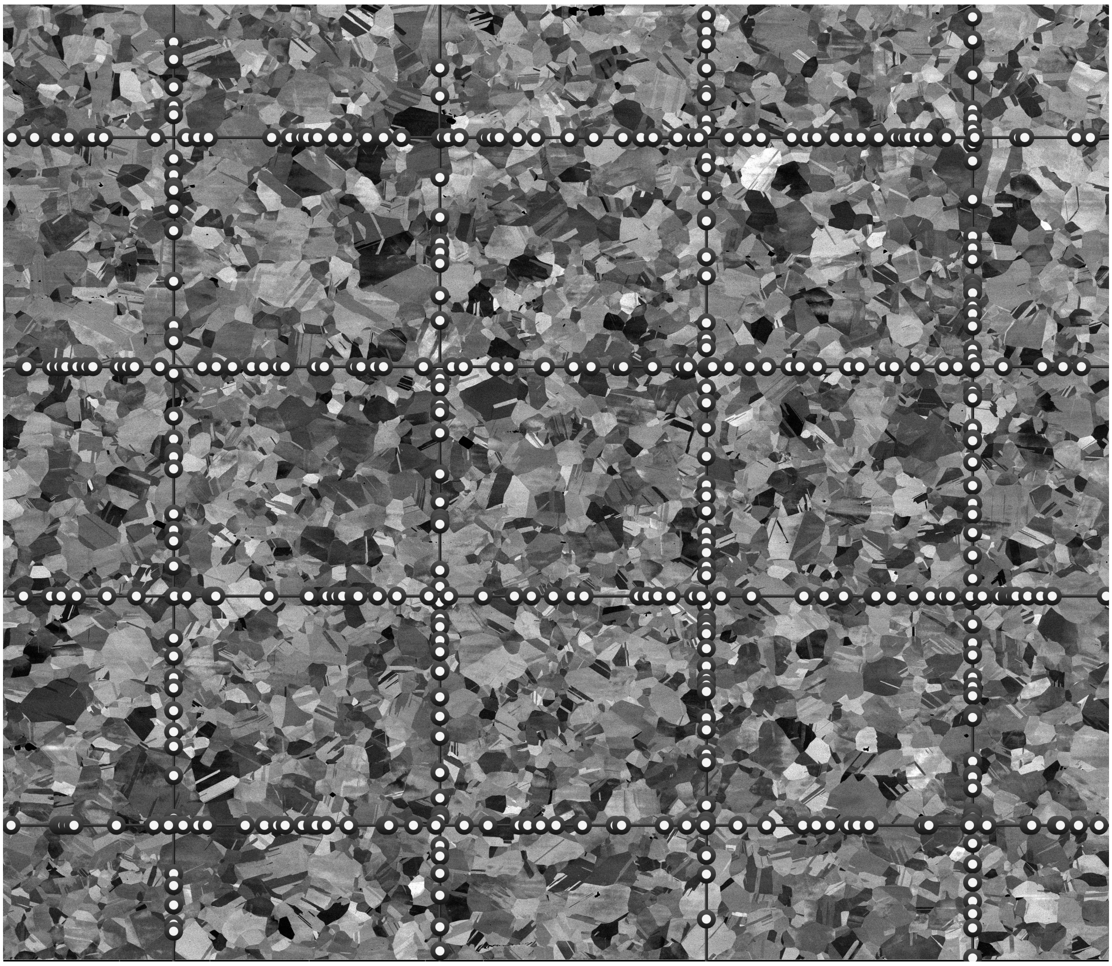

2.1. Statistical Analysis

Statistical Data	Length
Object Count	428
Minimum	0.7 µm
Maximum	24.5 µm
Average	7.4 µm
Standard deviation	4.6 µm
Skewness	0.0
Standard deviation (n-1)	4.6 µm
Variance	21.3 µm²
Variance (n-1)	21.3 µm²
Sum	3'159.0 µm

Statistical Data	Length
Sum of squares	32'420.4 µm²
Sum of cubes	415'755.9 µm³

2.1.1. Chord Lenght Distribution

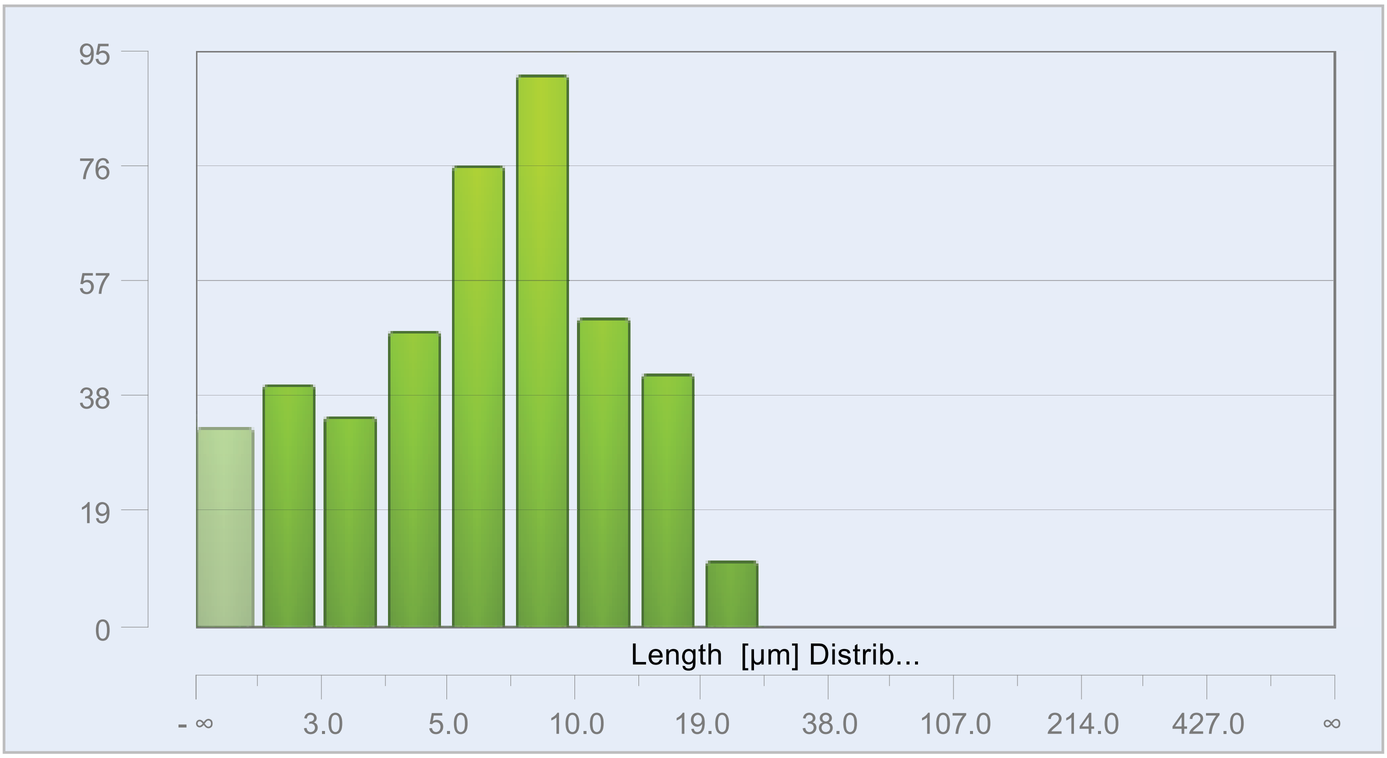

Start	End	Absolute Frequency	Absolute Frequency (accumulated)	Relative Frequency [%]	Relative Frequency (accumulated) [%]
	2.0 µm	33	33	8	8
2.0 µm	3.0 µm	40	73	9	17
3.0 µm	4.0 µm	35	108	8	25
4.0 µm	5.0 µm	49	157	11	37
5.0 µm	7.0 µm	76	233	18	54
7.0 µm	10.0 µm	91	324	21	76
10.0 µm	13.0 µm	51	375	12	88
13.0 µm	19.0 µm	42	417	10	97
19.0 µm	27.0 µm	11	428	3	100
27.0 µm	38.0 µm	0	428	0	100
38.0 µm	75.0 µm	0	428	0	100
75.0 µm	107.0 µm	0	428	0	100
107.0 µm	151.0 µm	0	428	0	100
151.0 µm	214.0 µm	0	428	0	100
214.0 µm	302.0 µm	0	428	0	100
302.0 µm	427.0 µm	0	428	0	100
427.0 µm	600.0 µm	0	428	0	100
600.0 µm		0	428	0	100

3. Single Result 2 (CrCoNi Twins grain size_ASTM 900C 30min_00140)

Mean chord length	7.5 µm
Grain size (ASTM)	10.8
Grain size (G643)	10.8
Grain stretching	97.4 %

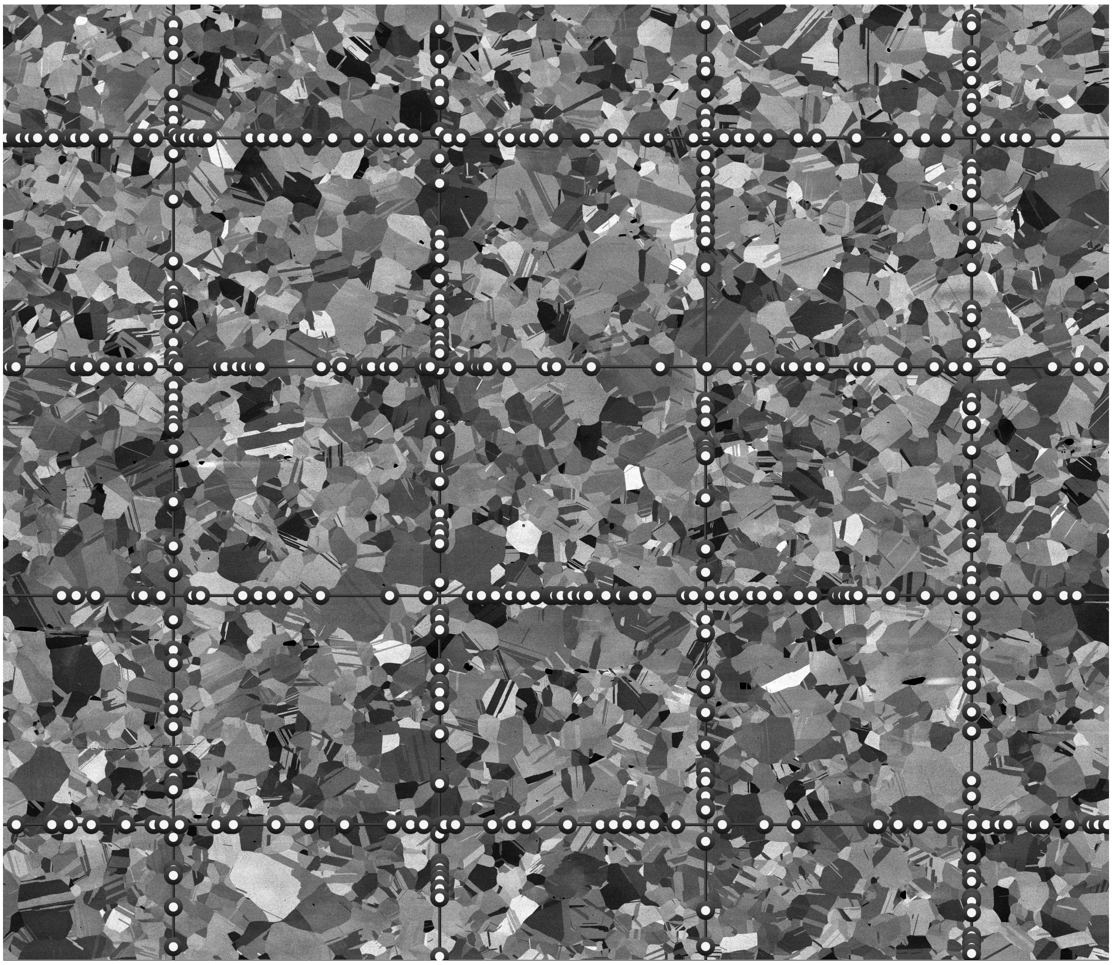

3.1. Statistical Analysis

Statistical Data	Length
Object Count	419
Minimum	0.4 µm
Maximum	37.8 µm
Average	7.5 µm
Standard deviation	5.4 µm
Skewness	0.0
Standard deviation (n-1)	5.4 µm
Variance	29.3 µm²
Variance (n-1)	29.4 µm²
Sum	3'159.0 µm
Sum of squares	36'106.2 µm²
Sum of cubes	554'793.9 µm³

3.1.1. Chord Lenght Distribution

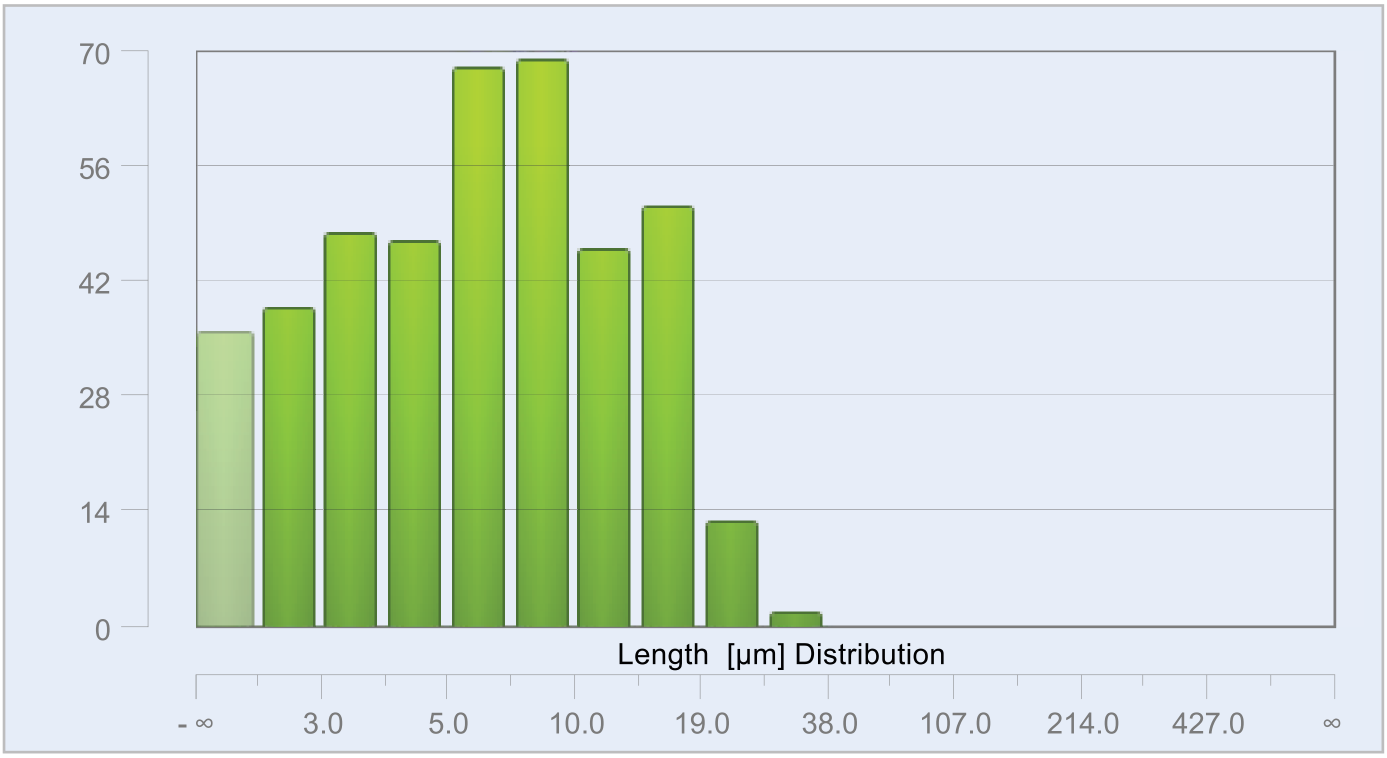

Start	End	Absolute Frequency	Absolute Frequency (accumulated)	Relative Frequency [%]	Relative Frequency (accumulated) [%]
	2.0 µm	36	36	9	9
2.0 µm	3.0 µm	39	75	9	18
3.0 µm	4.0 µm	48	123	11	29
4.0 µm	5.0 µm	47	170	11	41
5.0 µm	7.0 µm	68	238	16	57
7.0 µm	10.0 µm	69	307	16	73
10.0 µm	13.0 µm	46	353	11	84
13.0 µm	19.0 µm	51	404	12	96
19.0 µm	27.0 µm	13	417	3	100
27.0 µm	38.0 µm	2	419	0	100
38.0 µm	75.0 µm	0	419	0	100
75.0 µm	107.0 µm	0	419	0	100
107.0 µm	151.0 µm	0	419	0	100
151.0 µm	214.0 µm	0	419	0	100
214.0 µm	302.0 µm	0	419	0	100
302.0 µm	427.0 µm	0	419	0	100
427.0 µm	600.0 µm	0	419	0	100
600.0 µm		0	419	0	100

4. Single Result 3 (CrCoNi Twins grain size_ASTM 900C 30min_00141)

Mean chord length	6.5 µm
Grain size (ASTM)	11.3
Grain size (G643)	11.2
Grain stretching	92 %

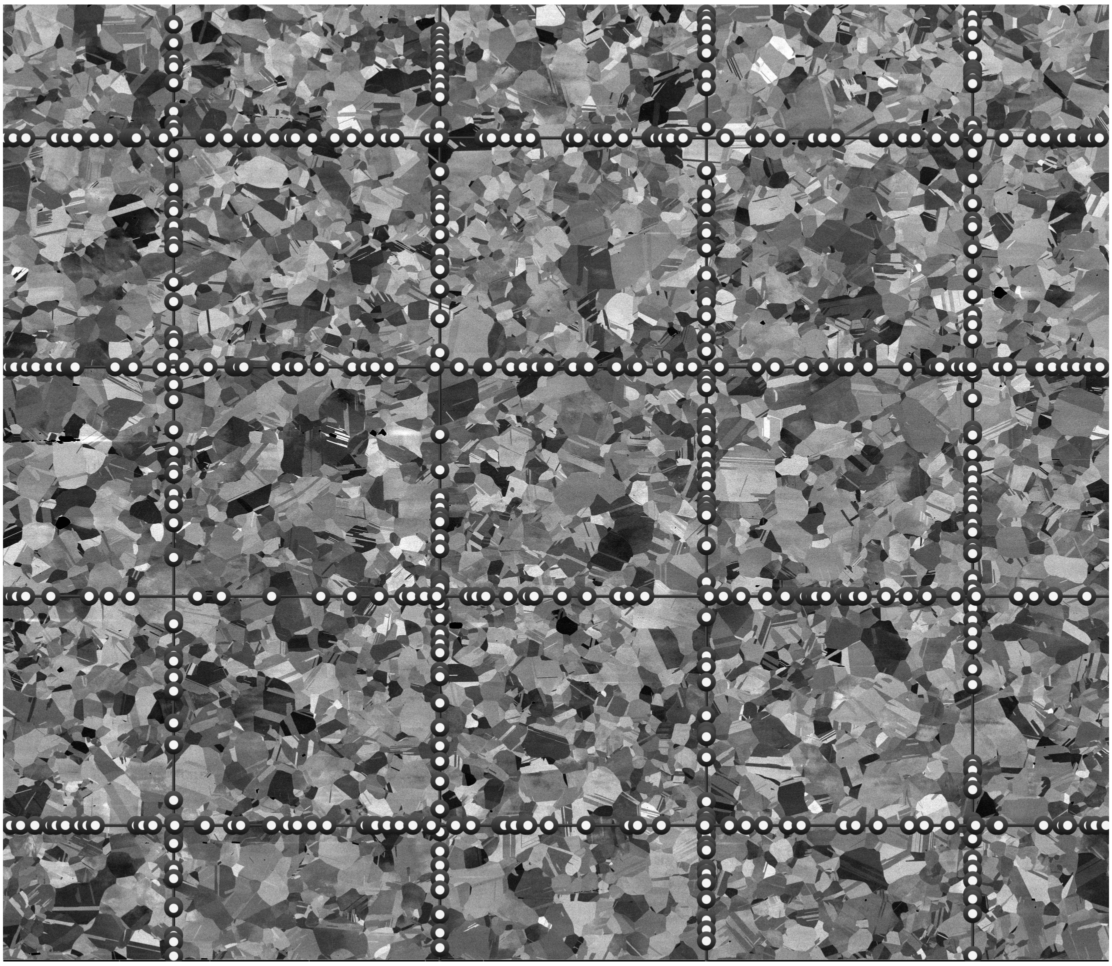

4.1. Statistical Analysis

Statistical Data	Length
Object Count	489
Minimum	0.5 µm
Maximum	31.3 µm
Average	6.5 µm
Standard deviation	4.5 µm
Skewness	0.0
Standard deviation (n-1)	4.5 µm
Variance	20.1 µm²
Variance (n-1)	20.2 µm²
Sum	3'159.0 µm
Sum of squares	30'256.2 µm²
Sum of cubes	394'819.5 µm³

4.1.1. Chord Lenght Distribution

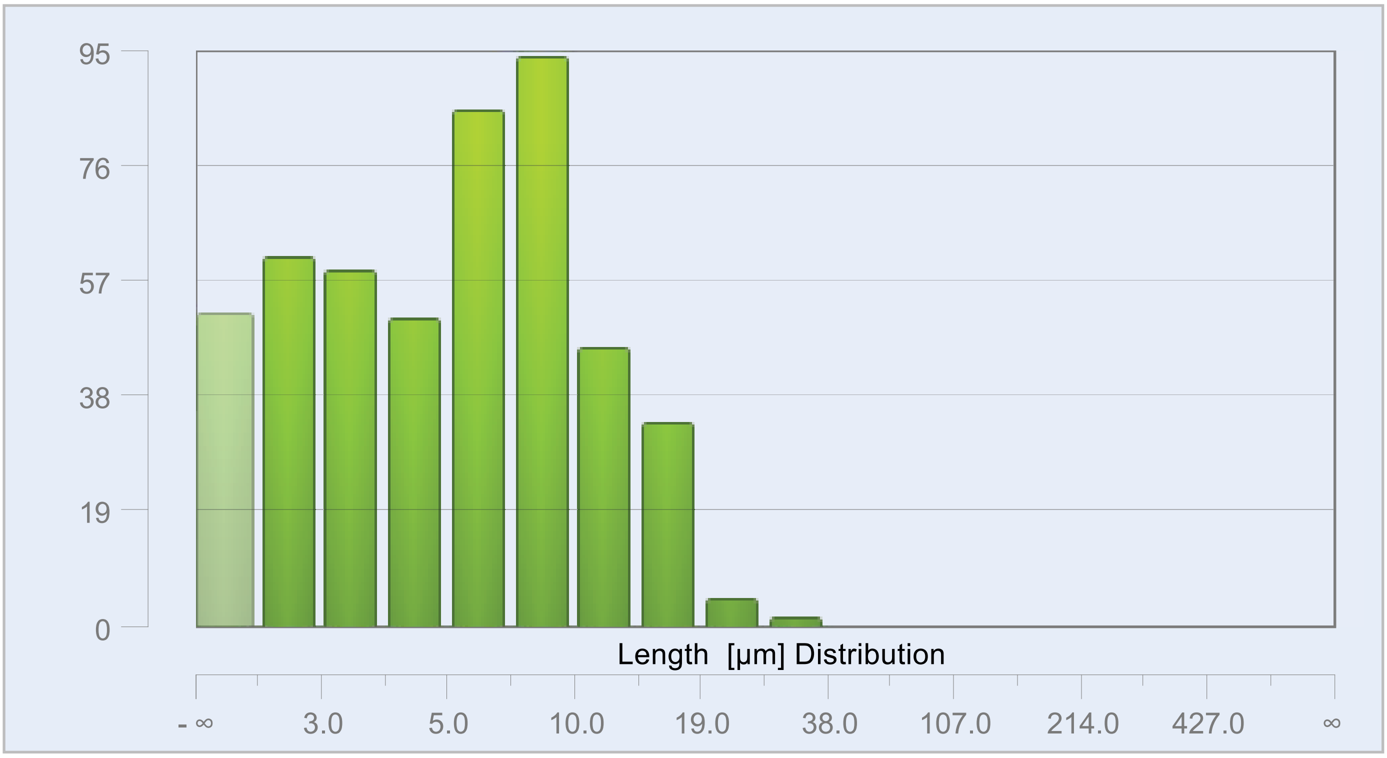

Start	End	Absolute Frequency	Absolute Frequency (accumulated)	Relative Frequency [%]	Relative Frequency (accumulated) [%]
	2.0 µm	52	52	11	11
2.0 µm	3.0 µm	61	113	12	23
3.0 µm	4.0 µm	59	172	12	35
4.0 µm	5.0 µm	51	223	10	46
5.0 µm	7.0 µm	85	308	17	63
7.0 µm	10.0 µm	94	402	19	82
10.0 µm	13.0 µm	46	448	9	92
13.0 µm	19.0 µm	34	482	7	99
19.0 µm	27.0 µm	5	487	1	100
27.0 µm	38.0 µm	2	489	0	100
38.0 µm	75.0 µm	0	489	0	100
75.0 µm	107.0 µm	0	489	0	100
107.0 µm	151.0 µm	0	489	0	100
151.0 µm	214.0 µm	0	489	0	100
214.0 µm	302.0 µm	0	489	0	100
302.0 µm	427.0 µm	0	489	0	100
427.0 µm	600.0 µm	0	489	0	100
600.0 µm		0	489	0	100

5. Single Result 4 (CrCoNi Twins grain size_ASTM 900C 30min_00142)

Mean chord length	6.9 µm
Grain size (ASTM)	11.1
Grain size (G643)	11
Grain stretching	95.9 %

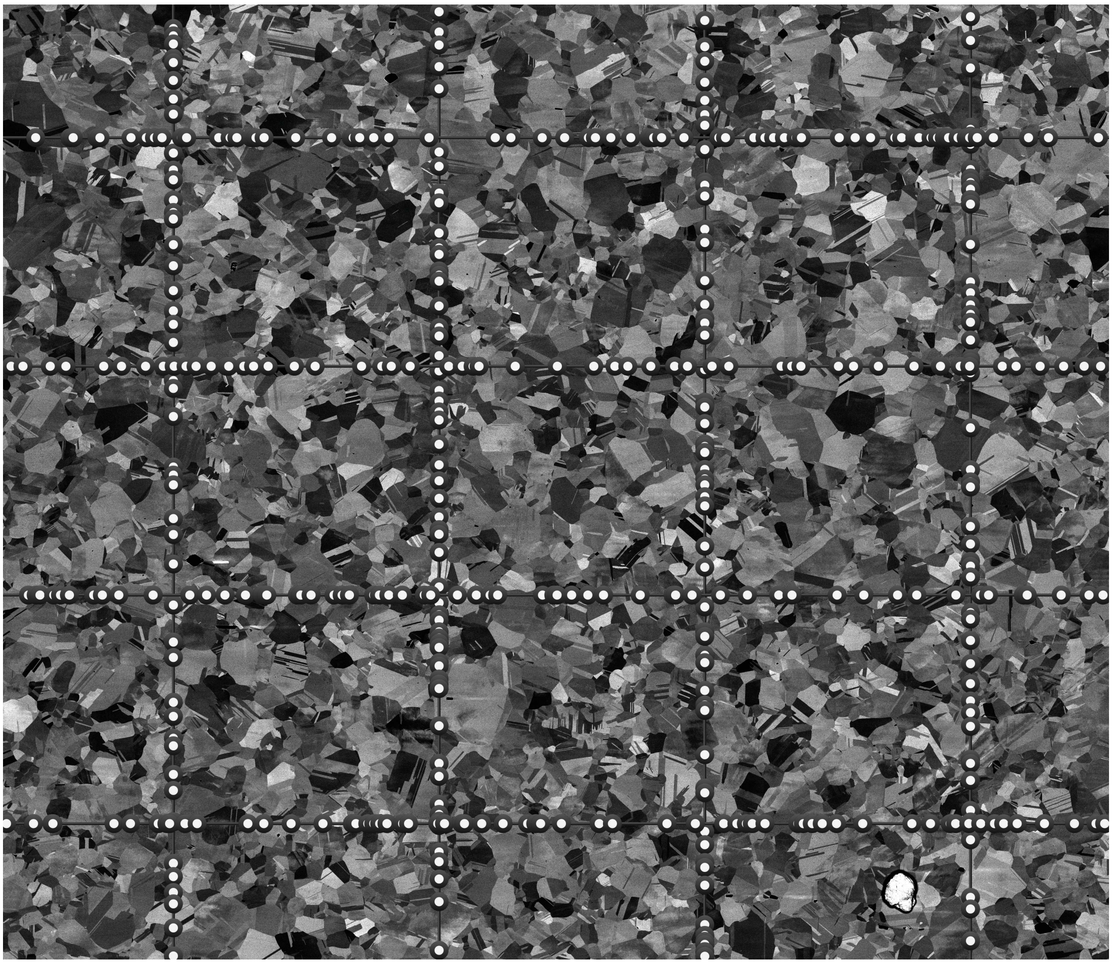

5.1. Statistical Analysis

Statistical Data	Length
Object Count	456
Minimum	0.4 µm
Maximum	25.3 µm
Average	6.9 µm
Standard deviation	4.7 µm
Skewness	0.0
Standard deviation (n-1)	4.7 µm
Variance	22.1 µm²
Variance (n-1)	22.1 µm²
Sum	3'157.4 µm
Sum of squares	31'923.7 µm²
Sum of cubes	411'698.6 µm³

5.1.1. Chord Lenght Distribution

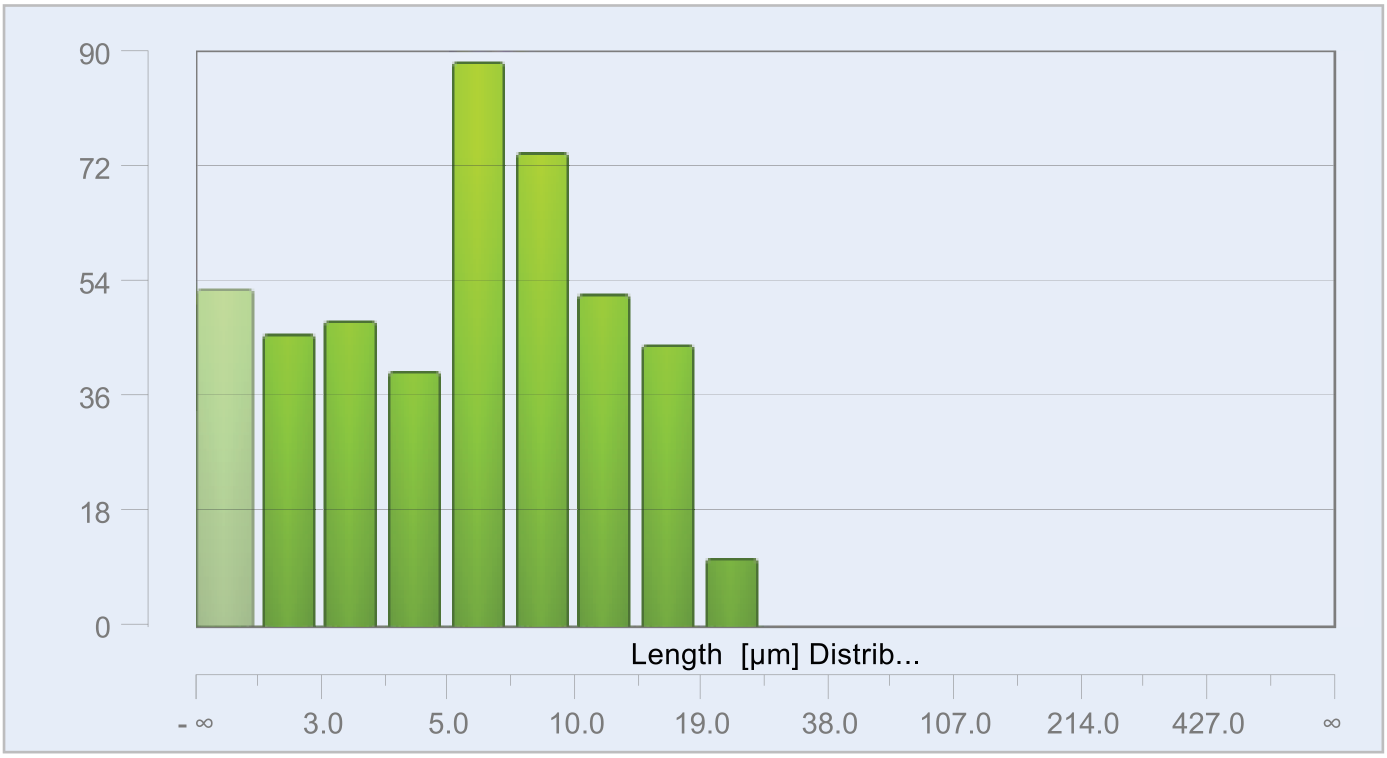

Start	End	Absolute Frequency	Absolute Frequency (accumulated)	Relative Frequency [%]	Relative Frequency (accumulated) [%]
	2.0 µm	53	53	12	12
2.0 µm	3.0 µm	46	99	10	22
3.0 µm	4.0 µm	48	147	11	32
4.0 µm	5.0 µm	40	187	9	41
5.0 µm	7.0 µm	88	275	19	60
7.0 µm	10.0 µm	74	349	16	77
10.0 µm	13.0 µm	52	401	11	88
13.0 µm	19.0 µm	44	445	10	98
19.0 µm	27.0 µm	11	456	2	100
27.0 µm	38.0 µm	0	456	0	100
38.0 µm	75.0 µm	0	456	0	100
75.0 µm	107.0 µm	0	456	0	100
107.0 µm	151.0 µm	0	456	0	100
151.0 µm	214.0 µm	0	456	0	100
214.0 µm	302.0 µm	0	456	0	100
302.0 µm	427.0 µm	0	456	0	100
427.0 µm	600.0 µm	0	456	0	100
600.0 µm		0	456	0	100
