# Supplementary material for: Benchmark dataset of the effect of grain size on strength in the single-phase FCC CrCoNi medium entropy alloy
Source: Data Brief. 2019 Oct 1;27:104592. doi: 10.1016/j.dib.2019.104592 (PMC6812030; doi:10.1016/j.dib.2019.104592)
Supplement: Multimedia component 1 [file mmc1.zip › CrCoNi_1173K_45min/CrCoNi_1173K_45min_c=4.0μm.pdf]

Analysebericht

Nov 7, 2017 3:08:56 PM

powered by imagic.ch

1. cumulative Result 1

Number of images	4
Grain size (ASTM)	12.6
Grain size (G643)	12.6
Grain stretching	93.8 %
Mean chord length	4 µm

2. Single Result 1 (CrCoNi - ASTM E 112_CrCoNi_homogenized_8.1mmSW_900°C_45min_00154)

Mean chord length	4.1 µm
Grain size (ASTM)	12.6
Grain size (G643)	12.5
Grain stretching	99 %

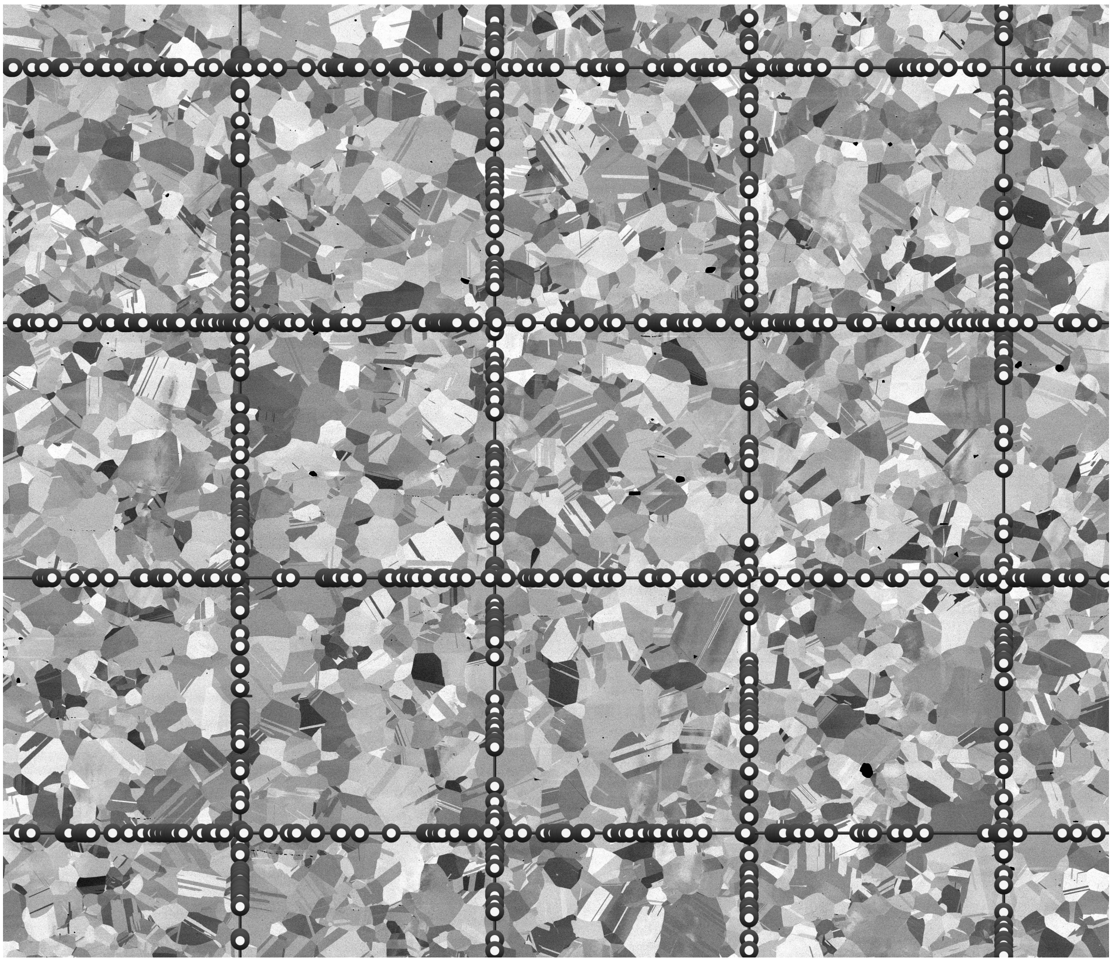

2.1. Statistical Analysis

Statistical Data	Length
Object Count	763
Minimum	0.2 µm
Maximum	23.8 µm
Average	4.1 µm
Standard deviation	3.8 µm
Skewness	0.0
Standard deviation (n-1)	3.8 µm
Variance	14.5 µm²
Variance (n-1)	14.5 µm²
Sum	3'150.0 µm

Statistical Data	Length
Sum of squares	24'065.1 µm²
Sum of cubes	262'980.1 µm³

2.1.1. Chord Lenght Distribution

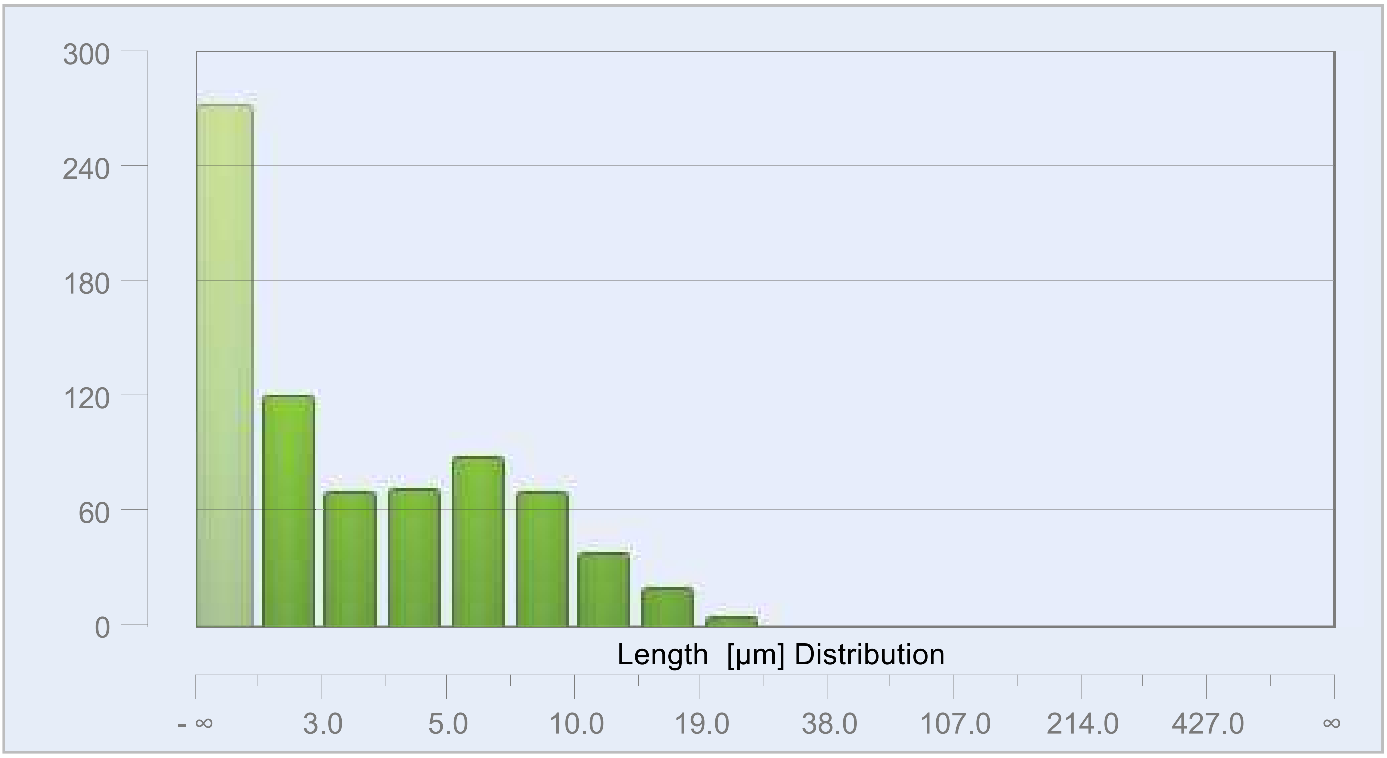

Start	End	Absolute Frequency	Absolute Frequency (accumulated)	Relative Frequency [%]	Relative Frequency (accumulated) [%]
	2.0 µm	272	272	36	36
2.0 µm	3.0 µm	121	393	16	52
3.0 µm	4.0 µm	71	464	9	61
4.0 µm	5.0 µm	73	537	10	70
5.0 µm	7.0 µm	89	626	12	82
7.0 µm	10.0 µm	71	697	9	91
10.0 µm	13.0 µm	39	736	5	96
13.0 µm	19.0 µm	21	757	3	99
19.0 µm	27.0 µm	6	763	1	100
27.0 µm	38.0 µm	0	763	0	100
38.0 µm	75.0 µm	0	763	0	100
75.0 µm	107.0 µm	0	763	0	100
107.0 µm	151.0 µm	0	763	0	100
151.0 µm	214.0 µm	0	763	0	100
214.0 µm	302.0 µm	0	763	0	100
302.0 µm	427.0 µm	0	763	0	100
427.0 µm	600.0 µm	0	763	0	100
600.0 µm		0	763	0	100

3. Single Result 2 (CrCoNi - ASTM E 112_CrCoNi_homogenized_8.1mmSW_900°C_45min_00155)

Mean chord length	3.9 µm
Grain size (ASTM)	12.7
Grain size (G643)	12.7
Grain stretching	94.3 %

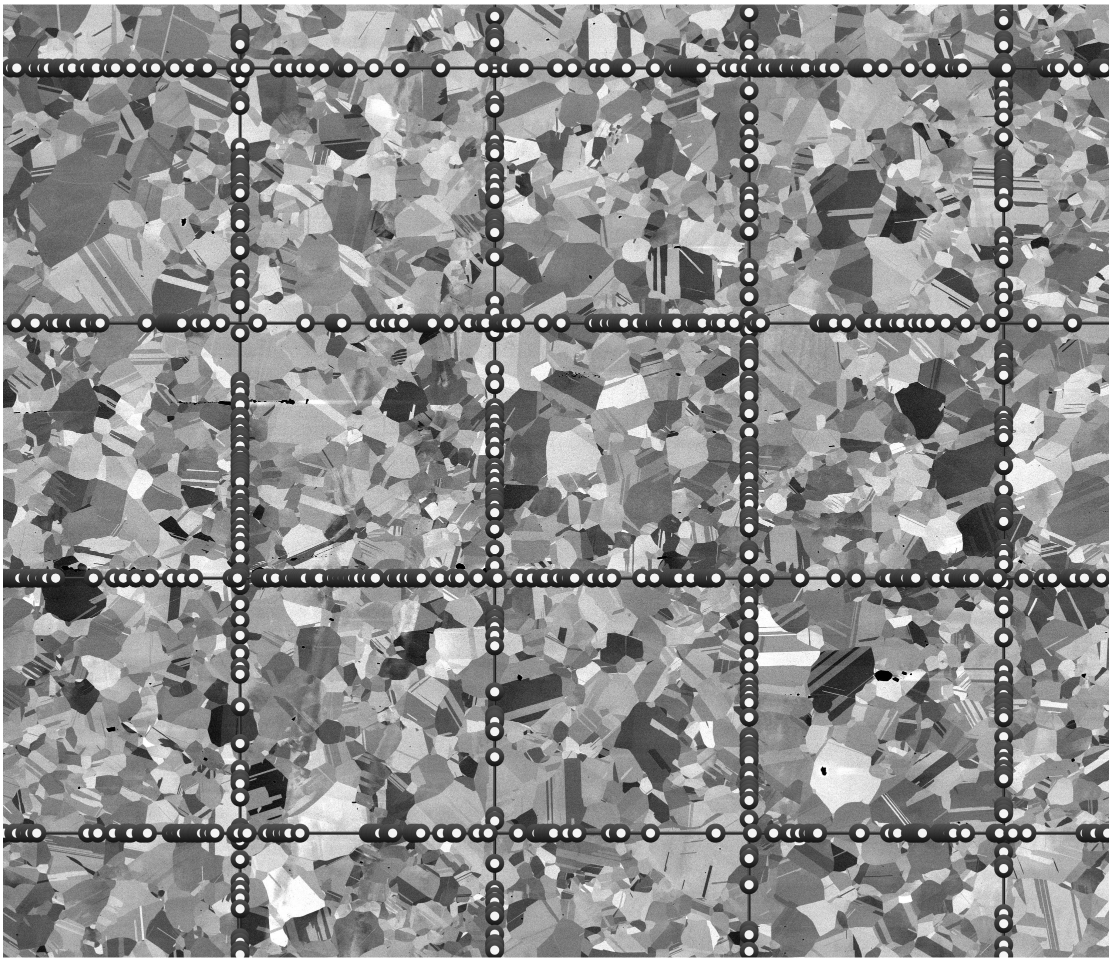

3.1. Statistical Analysis

Statistical Data	Length
Object Count	815
Minimum	0.2 µm
Maximum	26.1 µm
Average	3.9 µm
Standard deviation	4.0 µm
Skewness	0.0
Standard deviation (n-1)	4.0 µm
Variance	15.8 µm²
Variance (n-1)	15.8 µm²
Sum	3'150.0 µm
Sum of squares	25'052.6 µm²
Sum of cubes	298'850.6 µm³

3.1.1. Chord Lenght Distribution

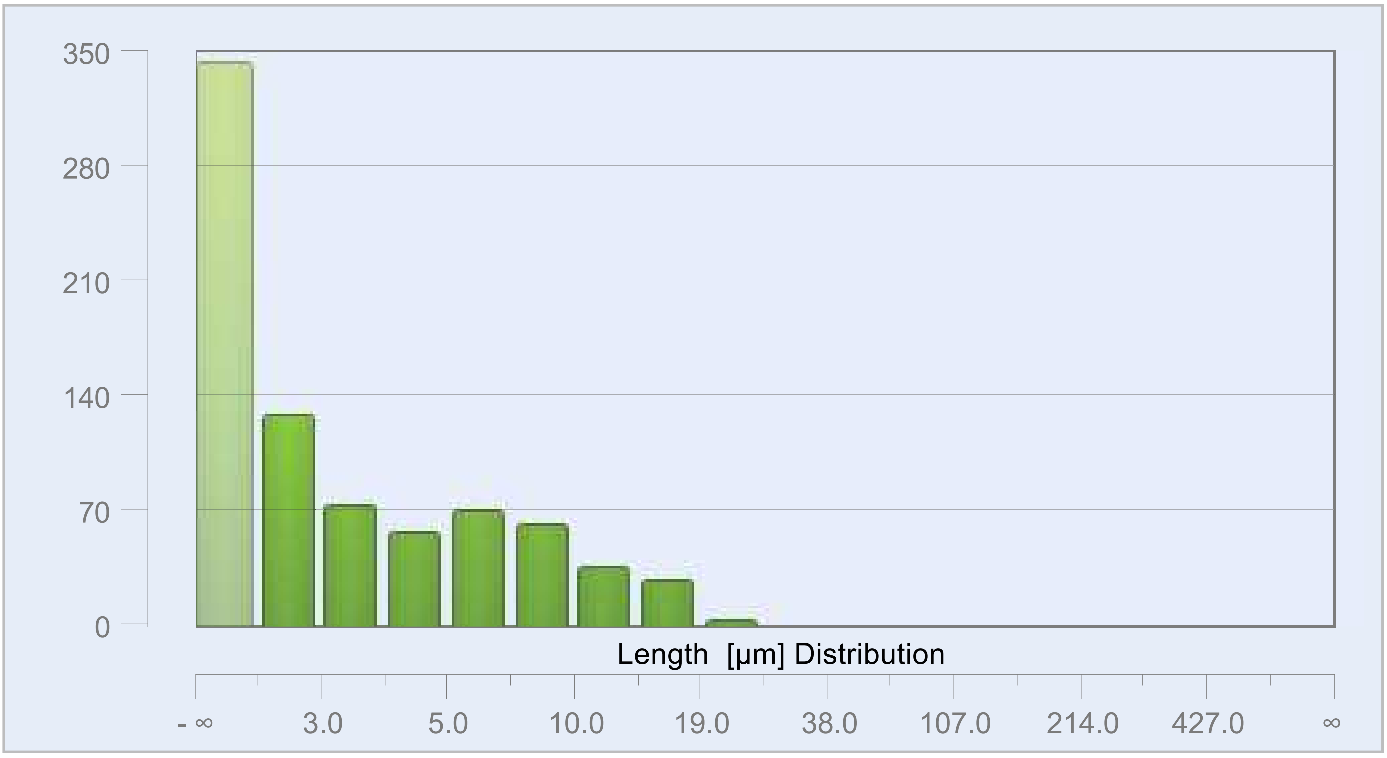

Start	End	Absolute Frequency	Absolute Frequency (accumulated)	Relative Frequency [%]	Relative Frequency (accumulated) [%]
	2.0 µm	343	343	42	42
2.0 µm	3.0 µm	130	473	16	58
3.0 µm	4.0 µm	75	548	9	67
4.0 µm	5.0 µm	59	607	7	74
5.0 µm	7.0 µm	71	678	9	83
7.0 µm	10.0 µm	63	741	8	91
10.0 µm	13.0 µm	38	779	5	96
13.0 µm	19.0 µm	30	809	4	99
19.0 µm	27.0 µm	6	815	1	100
27.0 µm	38.0 µm	0	815	0	100
38.0 µm	75.0 µm	0	815	0	100
75.0 µm	107.0 µm	0	815	0	100
107.0 µm	151.0 µm	0	815	0	100
151.0 µm	214.0 µm	0	815	0	100
214.0 µm	302.0 µm	0	815	0	100
302.0 µm	427.0 µm	0	815	0	100
427.0 µm	600.0 µm	0	815	0	100
600.0 µm		0	815	0	100

4. Single Result 3 (CrCoNi - ASTM E 112_CrCoNi_homogenized_8.1mmSW_900°C_45min_00156)

Mean chord length	4 µm
Grain size (ASTM)	12.6
Grain size (G643)	12.6
Grain stretching	85.9 %

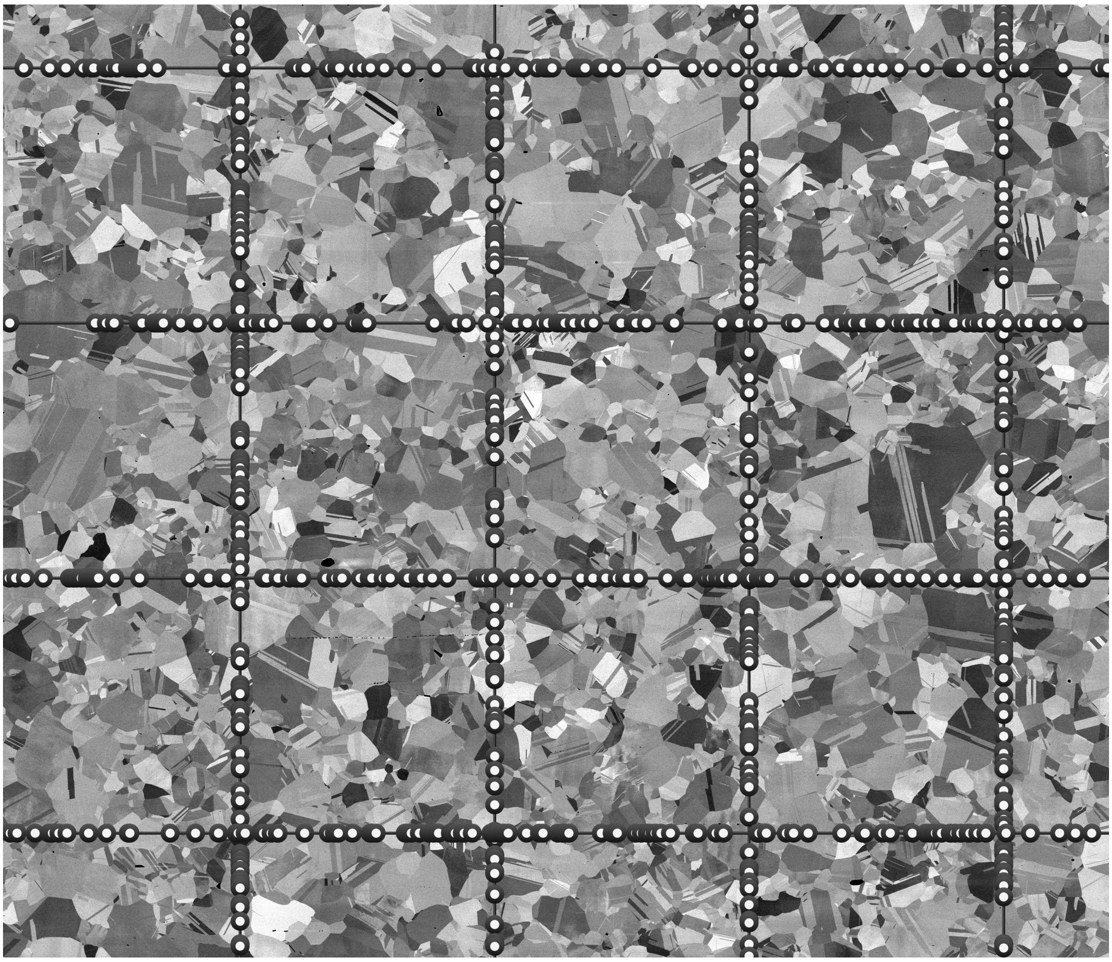

4.1. Statistical Analysis

Statistical Data	Length
Object Count	787
Minimum	0.2 µm
Maximum	32.7 µm
Average	4.0 µm
Standard deviation	3.9 µm
Skewness	0.0
Standard deviation (n-1)	3.9 µm
Variance	15.5 µm²
Variance (n-1)	15.5 µm²
Sum	3'150.0 µm
Sum of squares	24'828.3 µm²
Sum of cubes	298'927.1 µm³

4.1.1. Chord Lenght Distribution

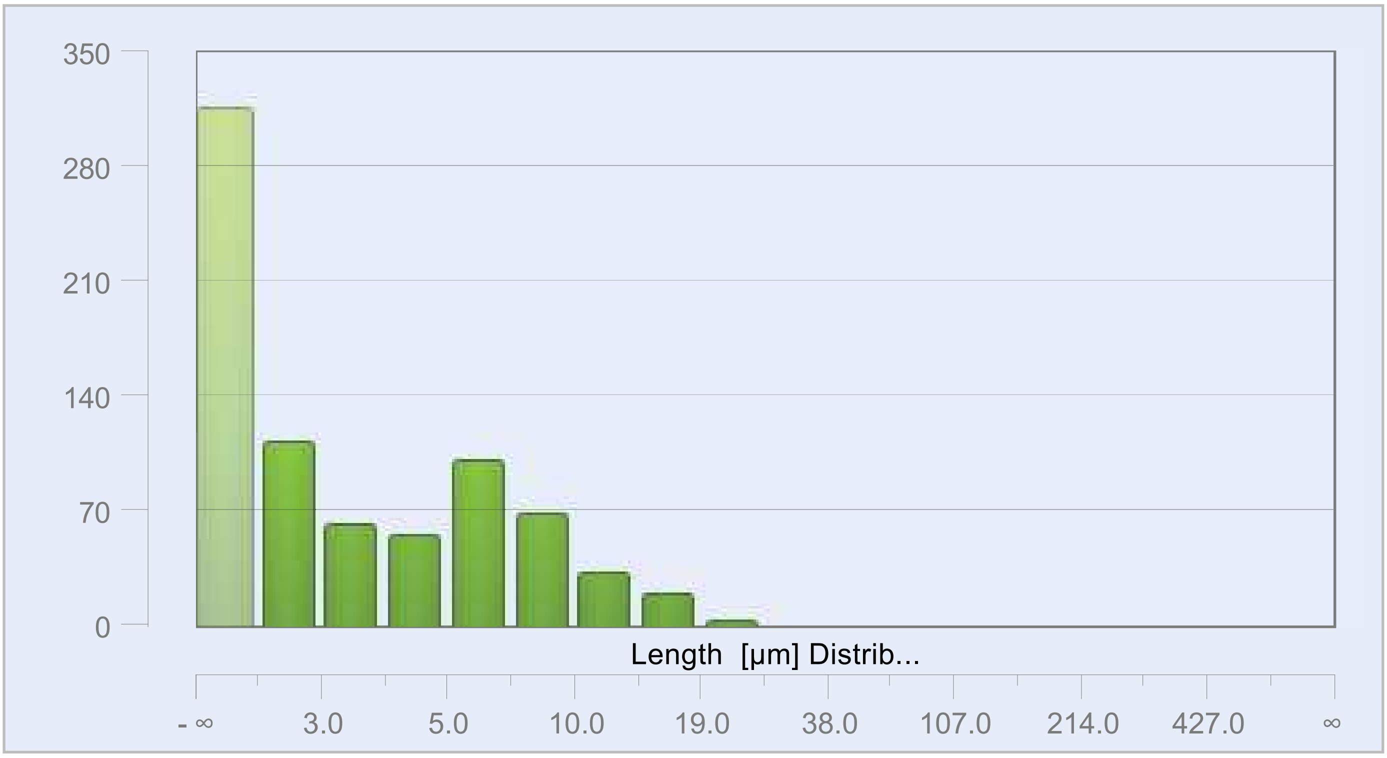

Start	End	Absolute Frequency	Absolute Frequency (accumulated)	Relative Frequency [%]	Relative Frequency (accumulated) [%]
	2.0 µm	316	316	40	40
2.0 µm	3.0 µm	113	429	14	55
3.0 µm	4.0 µm	64	493	8	63
4.0 µm	5.0 µm	58	551	7	70
5.0 µm	7.0 µm	103	654	13	83
7.0 µm	10.0 µm	70	724	9	92
10.0 µm	13.0 µm	35	759	4	96
13.0 µm	19.0 µm	21	780	3	99
19.0 µm	27.0 µm	6	786	1	100
27.0 µm	38.0 µm	1	787	0	100
38.0 µm	75.0 µm	0	787	0	100
75.0 µm	107.0 µm	0	787	0	100
107.0 µm	151.0 µm	0	787	0	100
151.0 µm	214.0 µm	0	787	0	100
214.0 µm	302.0 µm	0	787	0	100
302.0 µm	427.0 µm	0	787	0	100
427.0 µm	600.0 µm	0	787	0	100
600.0 µm		0	787	0	100

5. Single Result 4 (CrCoNi - ASTM E 112_CrCoNi_homogenized_8.1mmSW_900°C_45min_00157)

Mean chord length	4 µm
Grain size (ASTM)	12.6
Grain size (G643)	12.6
Grain stretching	96.4 %

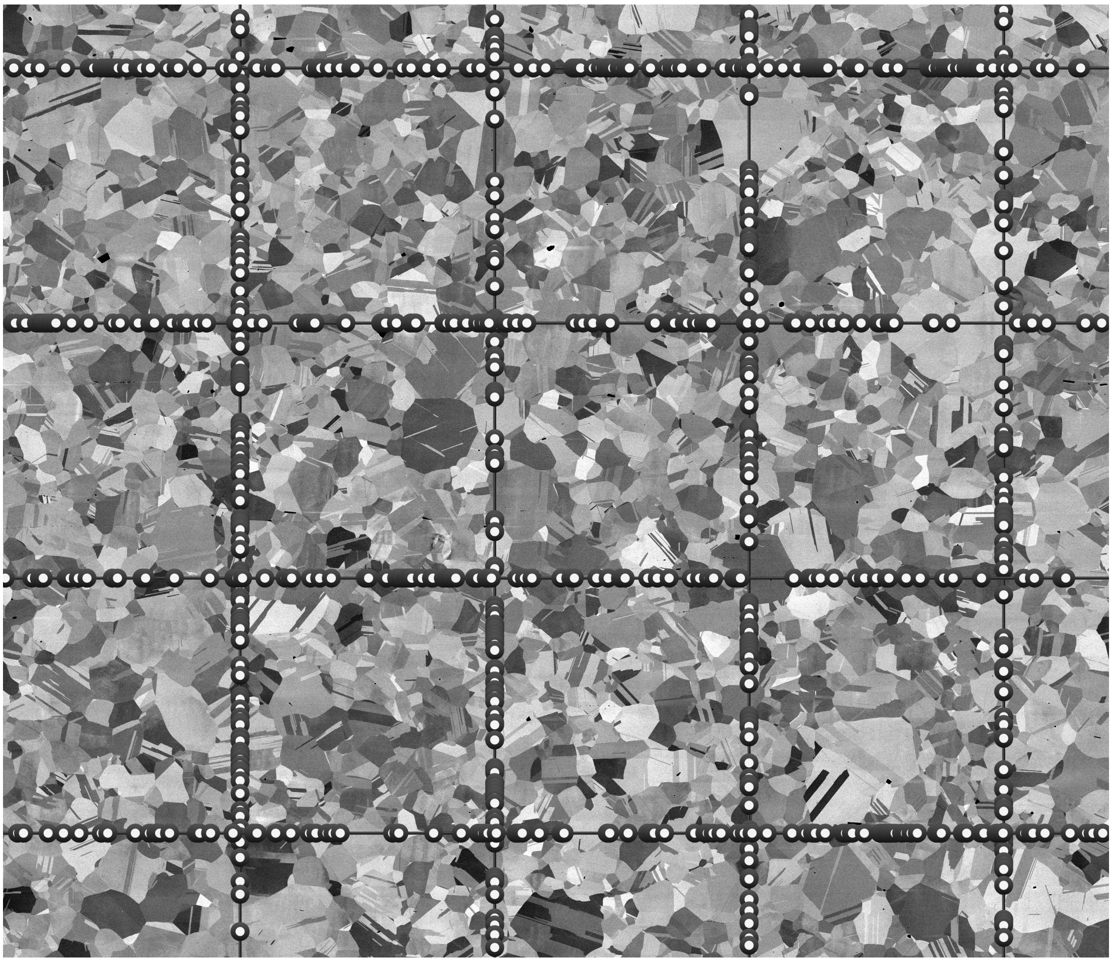

5.1. Statistical Analysis

Statistical Data	Length
Object Count	782
Minimum	0.2 µm
Maximum	27.9 µm
Average	4.0 µm
Standard deviation	4.0 µm
Skewness	0.0
Standard deviation (n-1)	4.0 µm
Variance	15.8 µm²
Variance (n-1)	15.8 µm²
Sum	3'150.0 µm
Sum of squares	25'046.9 µm²
Sum of cubes	294'037.9 µm³

5.1.1. Chord Lenght Distribution

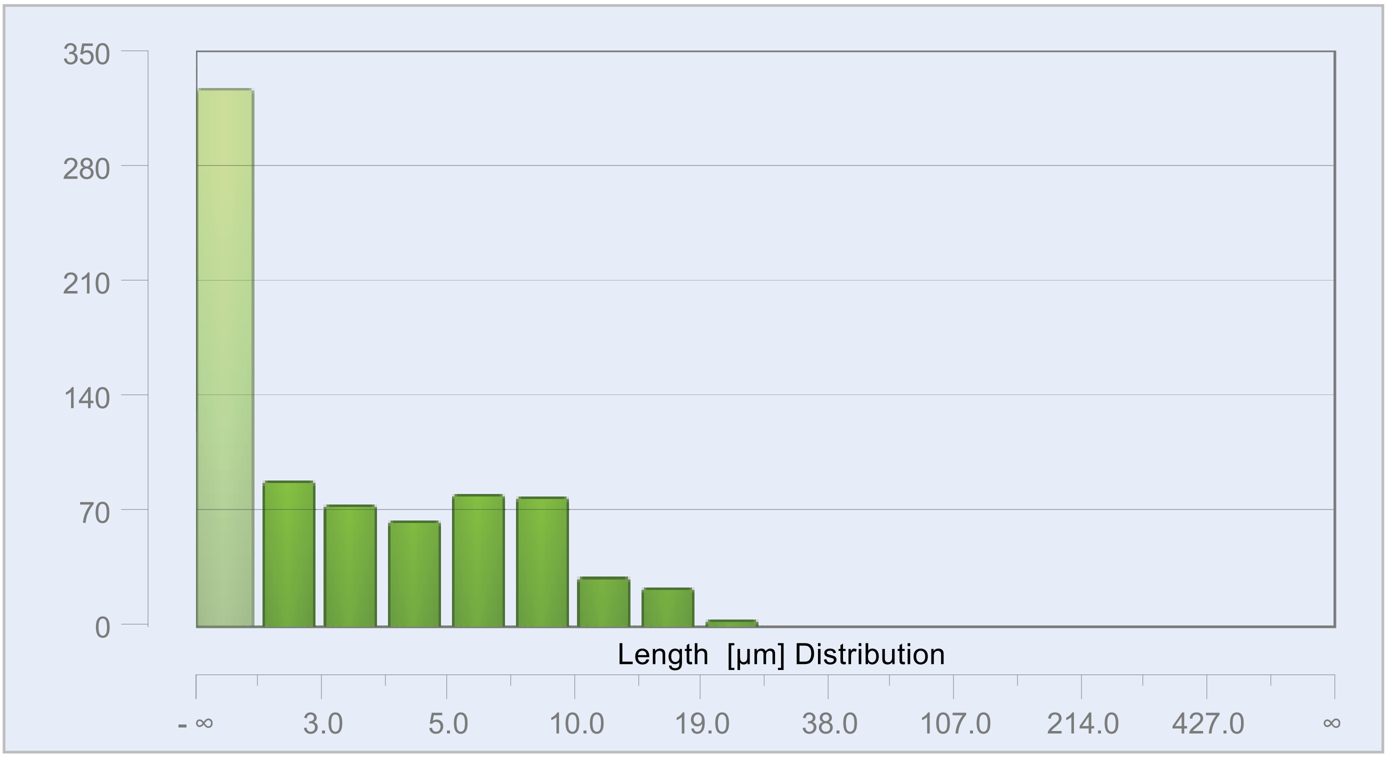

Start	End	Absolute Frequency	Absolute Frequency (accumulated)	Relative Frequency [%]	Relative Frequency (accumulated) [%]
	2.0 µm	327	327	42	42
2.0 µm	3.0 µm	89	416	11	53
3.0 µm	4.0 µm	75	491	10	63
4.0 µm	5.0 µm	65	556	8	71
5.0 µm	7.0 µm	82	638	10	82
7.0 µm	10.0 µm	80	718	10	92
10.0 µm	13.0 µm	32	750	4	96
13.0 µm	19.0 µm	25	775	3	99
19.0 µm	27.0 µm	6	781	1	100
27.0 µm	38.0 µm	1	782	0	100
38.0 µm	75.0 µm	0	782	0	100
75.0 µm	107.0 µm	0	782	0	100
107.0 µm	151.0 µm	0	782	0	100
151.0 µm	214.0 µm	0	782	0	100
214.0 µm	302.0 µm	0	782	0	100
302.0 µm	427.0 µm	0	782	0	100
427.0 µm	600.0 µm	0	782	0	100
600.0 µm		0	782	0	100
