# Supplementary material for: Benchmark dataset of the effect of grain size on strength in the single-phase FCC CrCoNi medium entropy alloy
Source: Data Brief. 2019 Oct 1;27:104592. doi: 10.1016/j.dib.2019.104592 (PMC6812030; doi:10.1016/j.dib.2019.104592)
Supplement: Multimedia component 1 [file mmc1.zip › CrCoNi_1173K_45min/CrCoNi_1173K_45min_d=8.4μm.pdf]

Analysebericht

Aug 24, 2017 4:35:58 PM

powered by imagic.ch

1. cumulative Result 1

Number of images	4
Grain size (ASTM)	10.5
Grain size (G643)	10.4
Grain stretching	92.4 %
Mean chord length	8.4 µm

2. Single Result 1 (CrCoNi Twins grain size_ASTM 900C 45min_00147)

Mean chord length	8.6 µm
Grain size (ASTM)	10.4
Grain size (G643)	10.4
Grain stretching	90.3 %

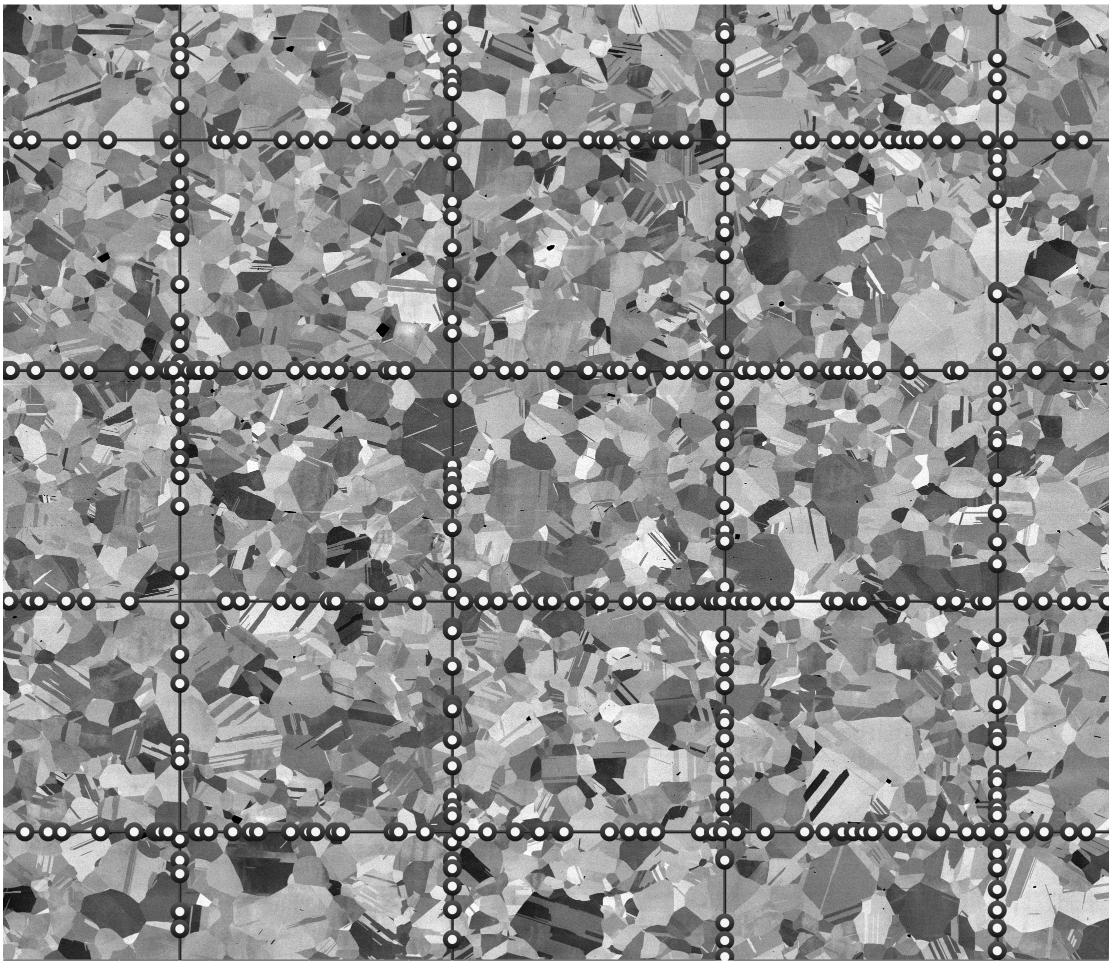

2.1. Statistical Analysis

Statistical Data	Length
Object Count	365
Minimum	0.4 µm
Maximum	36.9 µm
Average	8.6 µm
Standard deviation	6.0 µm
Skewness	0.0
Standard deviation (n-1)	6.0 µm
Variance	36.0 µm²
Variance (n-1)	36.1 µm²
Sum	3'156.2 µm

Statistical Data	Length
Sum of squares	40'415.9 µm²
Sum of cubes	692'272.2 µm³

2.1.1. Chord Lenght Distribution

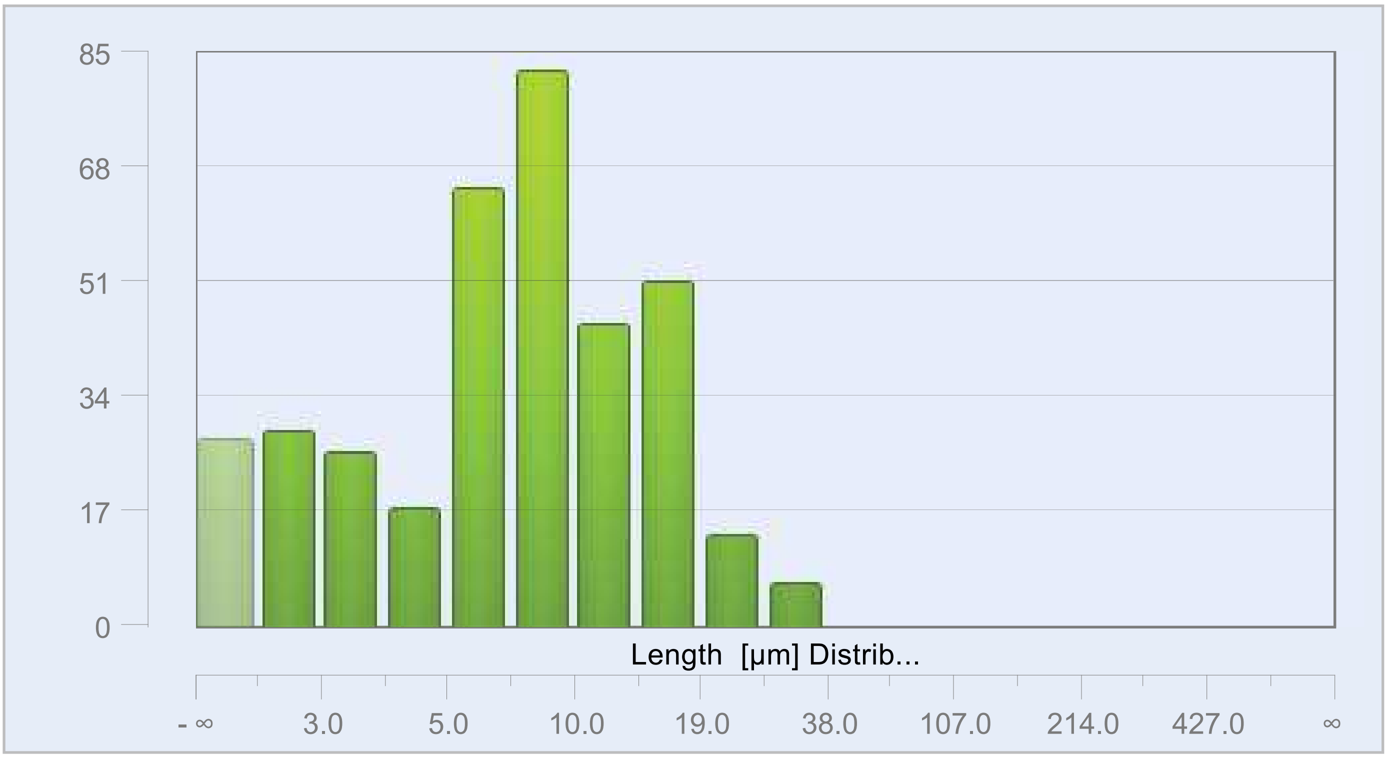

Start	End	Absolute Frequency	Absolute Frequency (accumulated)	Relative Frequency [%]	Relative Frequency (accumulated) [%]
	2.0 µm	28	28	8	8
2.0 µm	3.0 µm	29	57	8	16
3.0 µm	4.0 µm	26	83	7	23
4.0 µm	5.0 µm	18	101	5	28
5.0 µm	7.0 µm	65	166	18	45
7.0 µm	10.0 µm	82	248	22	68
10.0 µm	13.0 µm	45	293	12	80
13.0 µm	19.0 µm	51	344	14	94
19.0 µm	27.0 µm	14	358	4	98
27.0 µm	38.0 µm	7	365	2	100
38.0 µm	75.0 µm	0	365	0	100
75.0 µm	107.0 µm	0	365	0	100
107.0 µm	151.0 µm	0	365	0	100
151.0 µm	214.0 µm	0	365	0	100
214.0 µm	302.0 µm	0	365	0	100
302.0 µm	427.0 µm	0	365	0	100
427.0 µm	600.0 µm	0	365	0	100
600.0 µm		0	365	0	100

3. Single Result 2 (CrCoNi Twins grain size_ASTM 900C 45min_00148)

Mean chord length	8.4 µm
Grain size (ASTM)	10.5
Grain size (G643)	10.5
Grain stretching	81.2 %

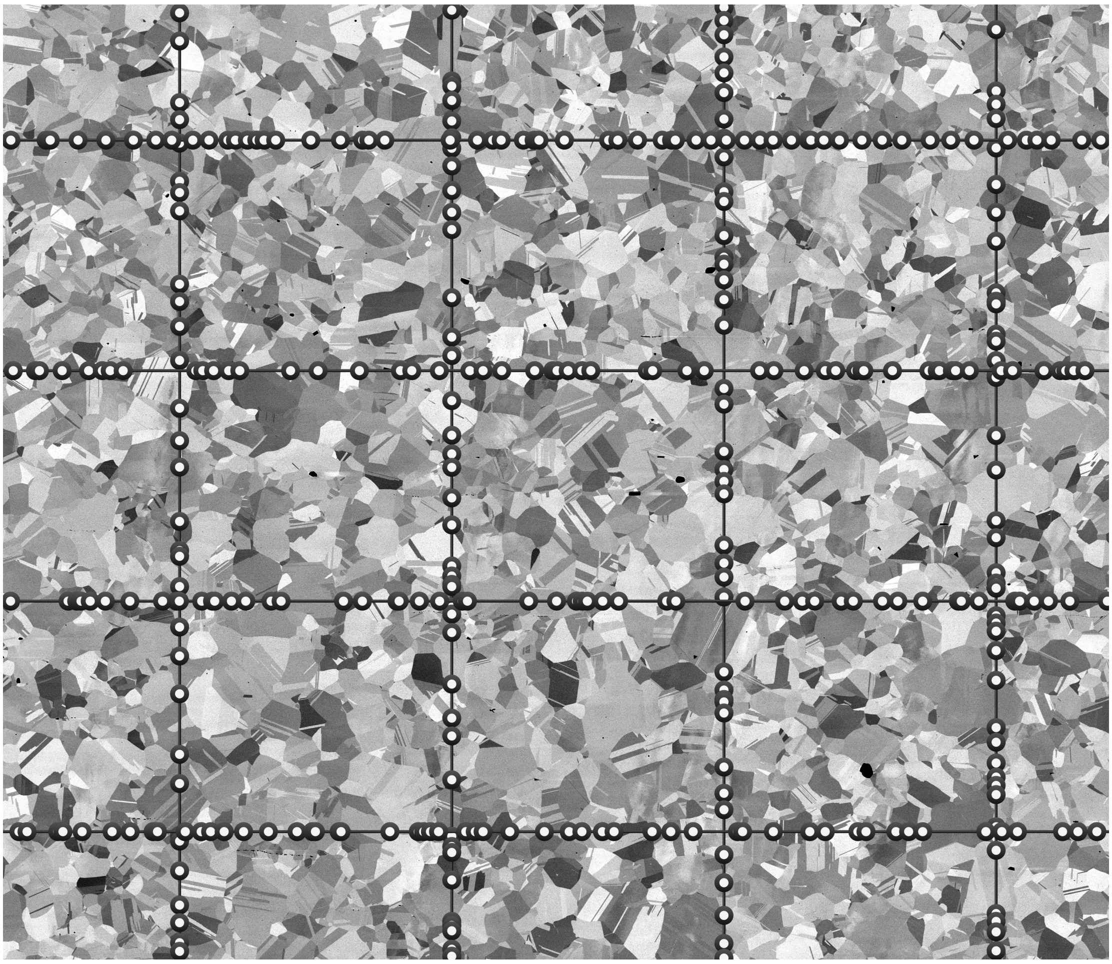

3.1. Statistical Analysis

Statistical Data	Length
Object Count	376
Minimum	0.5 µm
Maximum	28.6 µm
Average	8.4 µm
Standard deviation	5.8 µm
Skewness	0.0
Standard deviation (n-1)	5.9 µm
Variance	34.2 µm²
Variance (n-1)	34.3 µm²
Sum	3'156.2 µm
Sum of squares	39'344.1 µm²
Sum of cubes	637'334.8 µm³

3.1.1. Chord Lenght Distribution

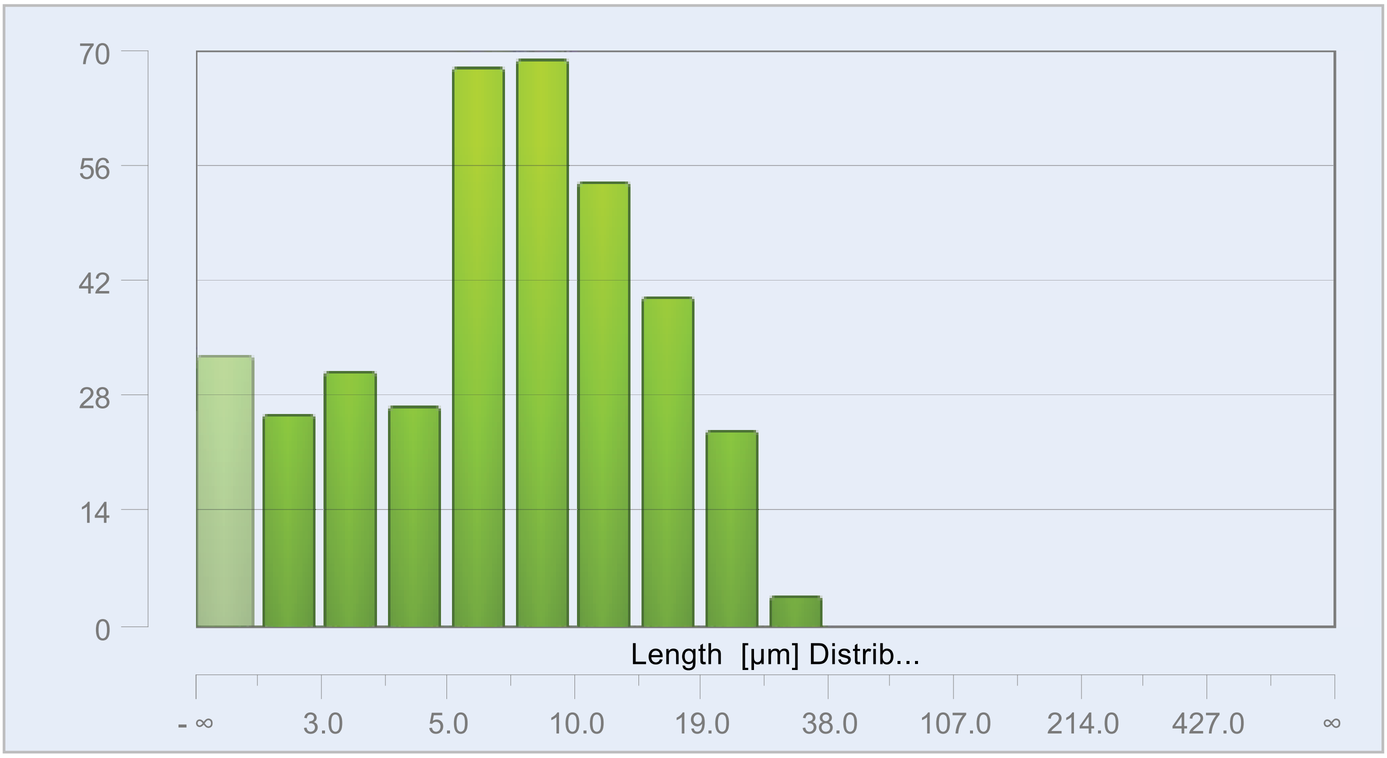

Start	End	Absolute Frequency	Absolute Frequency (accumulated)	Relative Frequency [%]	Relative Frequency (accumulated) [%]
	2.0 µm	33	33	9	9
2.0 µm	3.0 µm	26	59	7	16
3.0 µm	4.0 µm	31	90	8	24
4.0 µm	5.0 µm	27	117	7	31
5.0 µm	7.0 µm	68	185	18	49
7.0 µm	10.0 µm	69	254	18	68
10.0 µm	13.0 µm	54	308	14	82
13.0 µm	19.0 µm	40	348	11	93
19.0 µm	27.0 µm	24	372	6	99
27.0 µm	38.0 µm	4	376	1	100
38.0 µm	75.0 µm	0	376	0	100
75.0 µm	107.0 µm	0	376	0	100
107.0 µm	151.0 µm	0	376	0	100
151.0 µm	214.0 µm	0	376	0	100
214.0 µm	302.0 µm	0	376	0	100
302.0 µm	427.0 µm	0	376	0	100
427.0 µm	600.0 µm	0	376	0	100
600.0 µm		0	376	0	100

4. Single Result 3 (CrCoNi Twins grain size_ASTM 900C 45min_00149)

Mean chord length	8.4 µm
Grain size (ASTM)	10.5
Grain size (G643)	10.5
Grain stretching	94 %

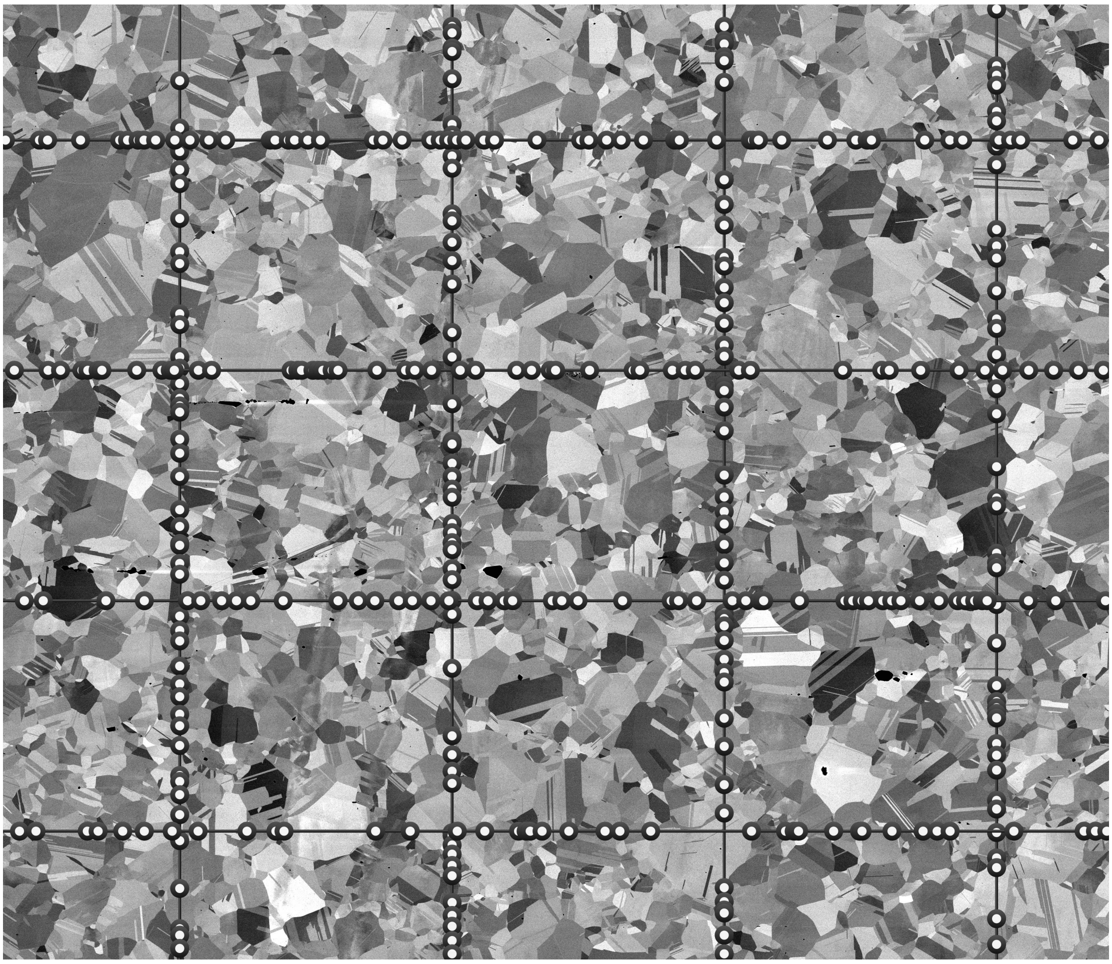

4.1. Statistical Analysis

Statistical Data	Length
Object Count	376
Minimum	0.7 µm
Maximum	38.6 µm
Average	8.4 µm
Standard deviation	6.3 µm
Skewness	0.0
Standard deviation (n-1)	6.4 µm
Variance	40.3 µm²
Variance (n-1)	40.4 µm²
Sum	3'154.5 µm
Sum of squares	41'601.6 µm²
Sum of cubes	763'660.4 µm³

4.1.1. Chord Lenght Distribution

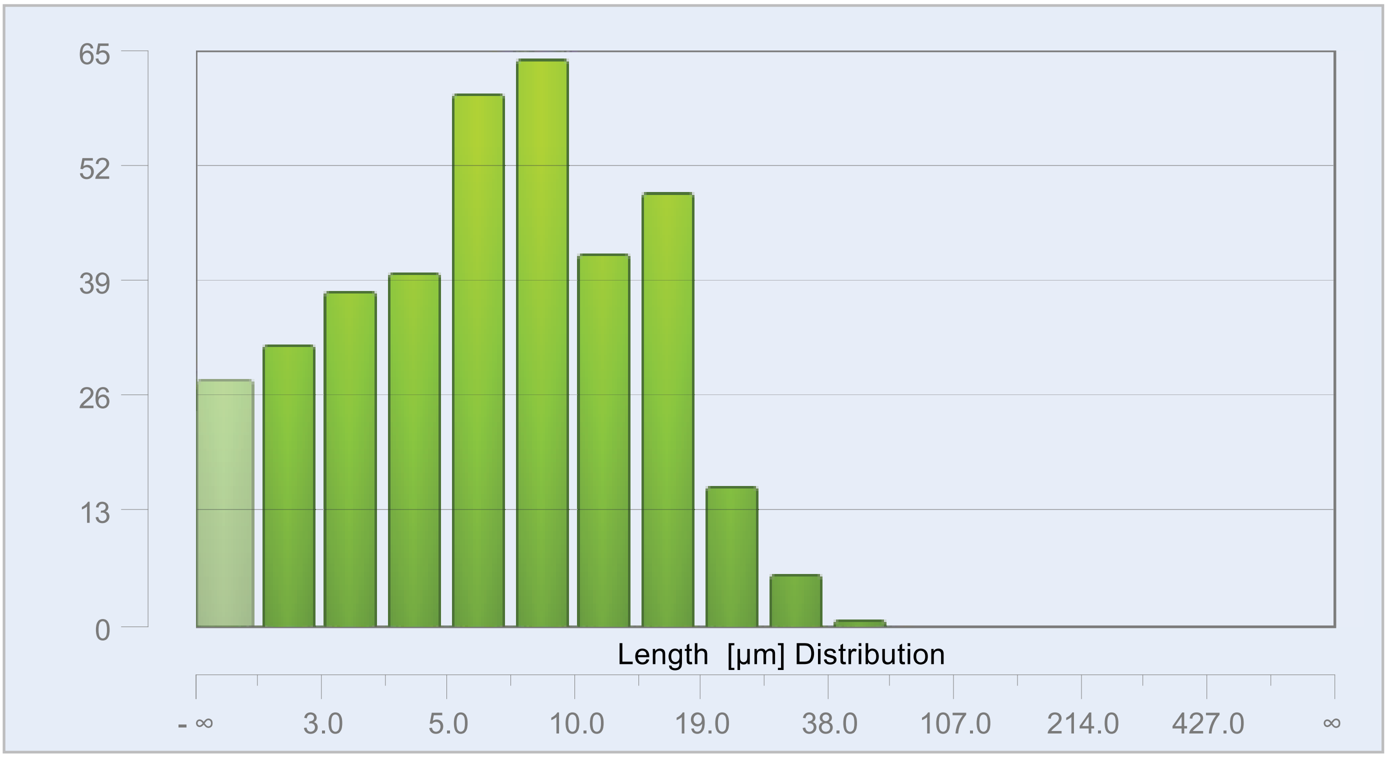

Start	End	Absolute Frequency	Absolute Frequency (accumulated)	Relative Frequency [%]	Relative Frequency (accumulated) [%]
	2.0 µm	28	28	7	7
2.0 µm	3.0 µm	32	60	9	16
3.0 µm	4.0 µm	38	98	10	26
4.0 µm	5.0 µm	40	138	11	37
5.0 µm	7.0 µm	60	198	16	53
7.0 µm	10.0 µm	64	262	17	70
10.0 µm	13.0 µm	42	304	11	81
13.0 µm	19.0 µm	49	353	13	94
19.0 µm	27.0 µm	16	369	4	98
27.0 µm	38.0 µm	6	375	2	100
38.0 µm	75.0 µm	1	376	0	100
75.0 µm	107.0 µm	0	376	0	100
107.0 µm	151.0 µm	0	376	0	100
151.0 µm	214.0 µm	0	376	0	100
214.0 µm	302.0 µm	0	376	0	100
302.0 µm	427.0 µm	0	376	0	100
427.0 µm	600.0 µm	0	376	0	100
600.0 µm		0	376	0	100

5. Single Result 4 (CrCoNi Twins grain size_ASTM 900C 45min_00150)

Mean chord length	8.3 µm
Grain size (ASTM)	10.5
Grain size (G643)	10.5
Grain stretching	93.1 %

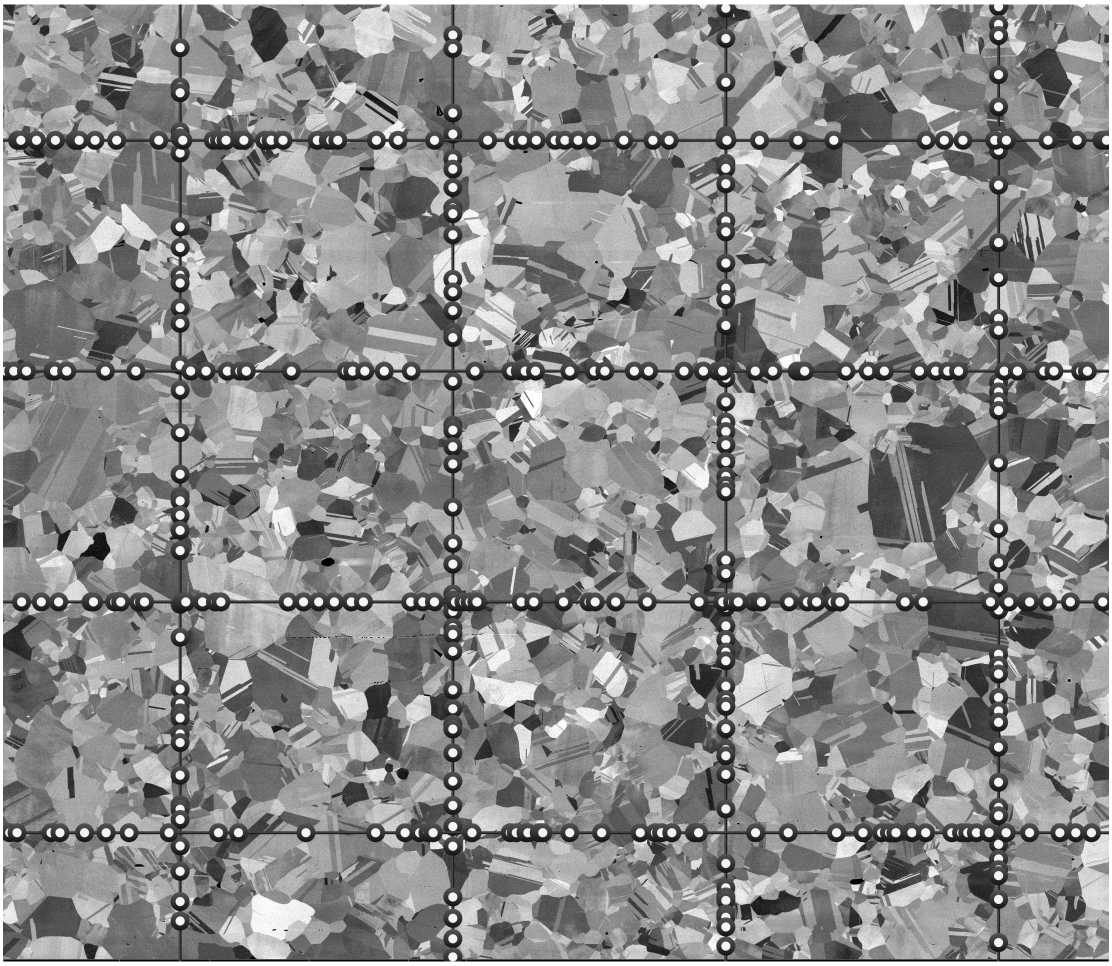

5.1. Statistical Analysis

Statistical Data	Length
Object Count	381
Minimum	0.6 µm
Maximum	35.4 µm
Average	8.3 µm
Standard deviation	5.9 µm
Skewness	0.0
Standard deviation (n-1)	5.9 µm
Variance	35.2 µm²
Variance (n-1)	35.3 µm²
Sum	3'159.9 µm
Sum of squares	39'607.4 µm²
Sum of cubes	647'061.8 µm³

5.1.1. Chord Lenght Distribution

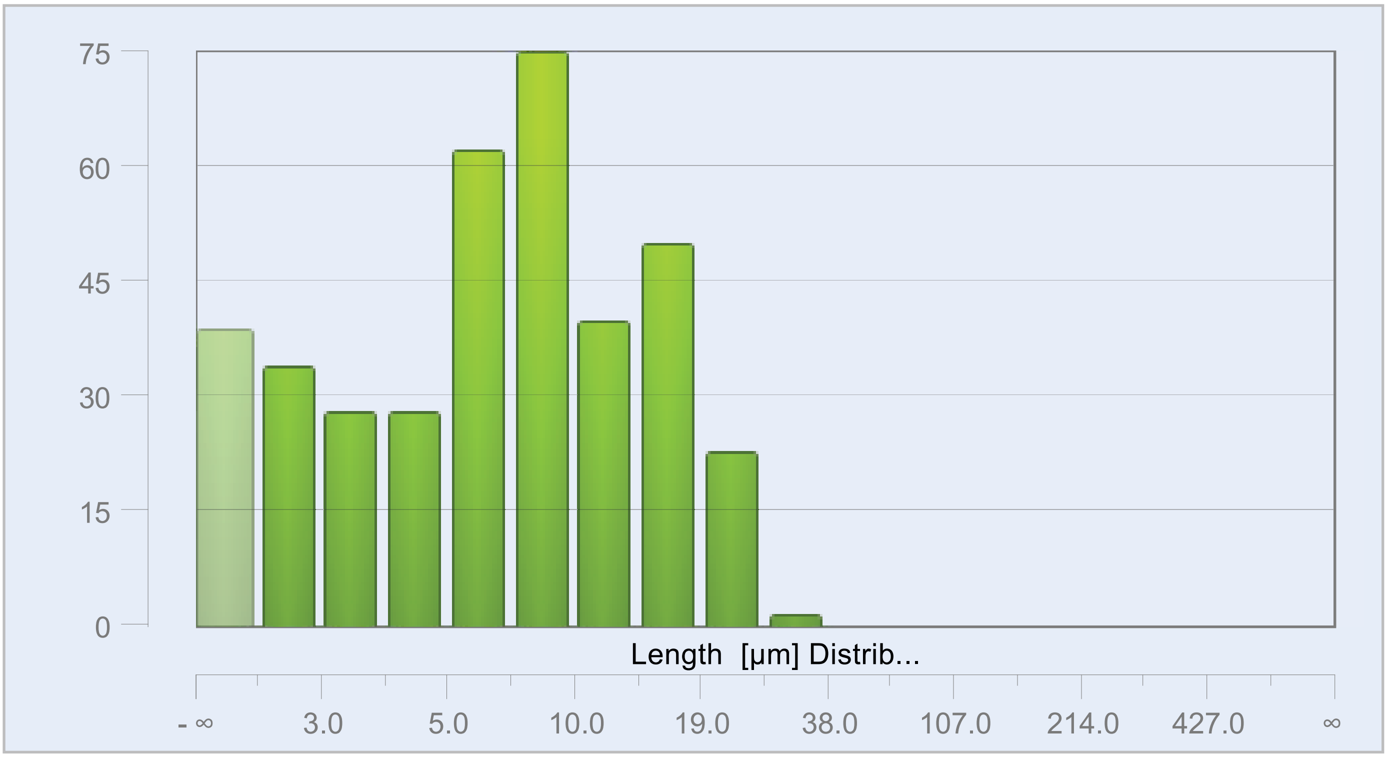

Start	End	Absolute Frequency	Absolute Frequency (accumulated)	Relative Frequency [%]	Relative Frequency (accumulated) [%]
	2.0 µm	39	39	10	10
2.0 µm	3.0 µm	34	73	9	19
3.0 µm	4.0 µm	28	101	7	27
4.0 µm	5.0 µm	28	129	7	34
5.0 µm	7.0 µm	62	191	16	50
7.0 µm	10.0 µm	75	266	20	70
10.0 µm	13.0 µm	40	306	10	80
13.0 µm	19.0 µm	50	356	13	93
19.0 µm	27.0 µm	23	379	6	99
27.0 µm	38.0 µm	2	381	1	100
38.0 µm	75.0 µm	0	381	0	100
75.0 µm	107.0 µm	0	381	0	100
107.0 µm	151.0 µm	0	381	0	100
151.0 µm	214.0 µm	0	381	0	100
214.0 µm	302.0 µm	0	381	0	100
302.0 µm	427.0 µm	0	381	0	100
427.0 µm	600.0 µm	0	381	0	100
600.0 µm		0	381	0	100
