# Supplementary material for: Benchmark dataset of the effect of grain size on strength in the single-phase FCC CrCoNi medium entropy alloy
Source: Data Brief. 2019 Oct 1;27:104592. doi: 10.1016/j.dib.2019.104592 (PMC6812030; doi:10.1016/j.dib.2019.104592)
Supplement: Multimedia component 1 [file mmc1.zip › CrCoNi_1173K_60min/CrCoNi_1173K_60min_c=4.8μm.pdf]

Analysebericht

06.11.2017 10:55:39

powered by imagic.ch

1. cumulative Result 1

Anzahl Bilder	4
Korngröße (ASTM)	12,1
Korngröße (G643)	12,1
Kornstreckung	90,2 %
Mittlere Sehnenlänge	4,8 µm

2. Single Result 1 (CrCoNi - ASTM E 112_CrCoNi_homogenized_8.1mmSW_900°C_60min_00150)

Mittlere Sehnenlänge	4,4 µm
Korngröße (ASTM)	12,4
Korngröße (G643)	12,3
Kornstreckung	96,1 %

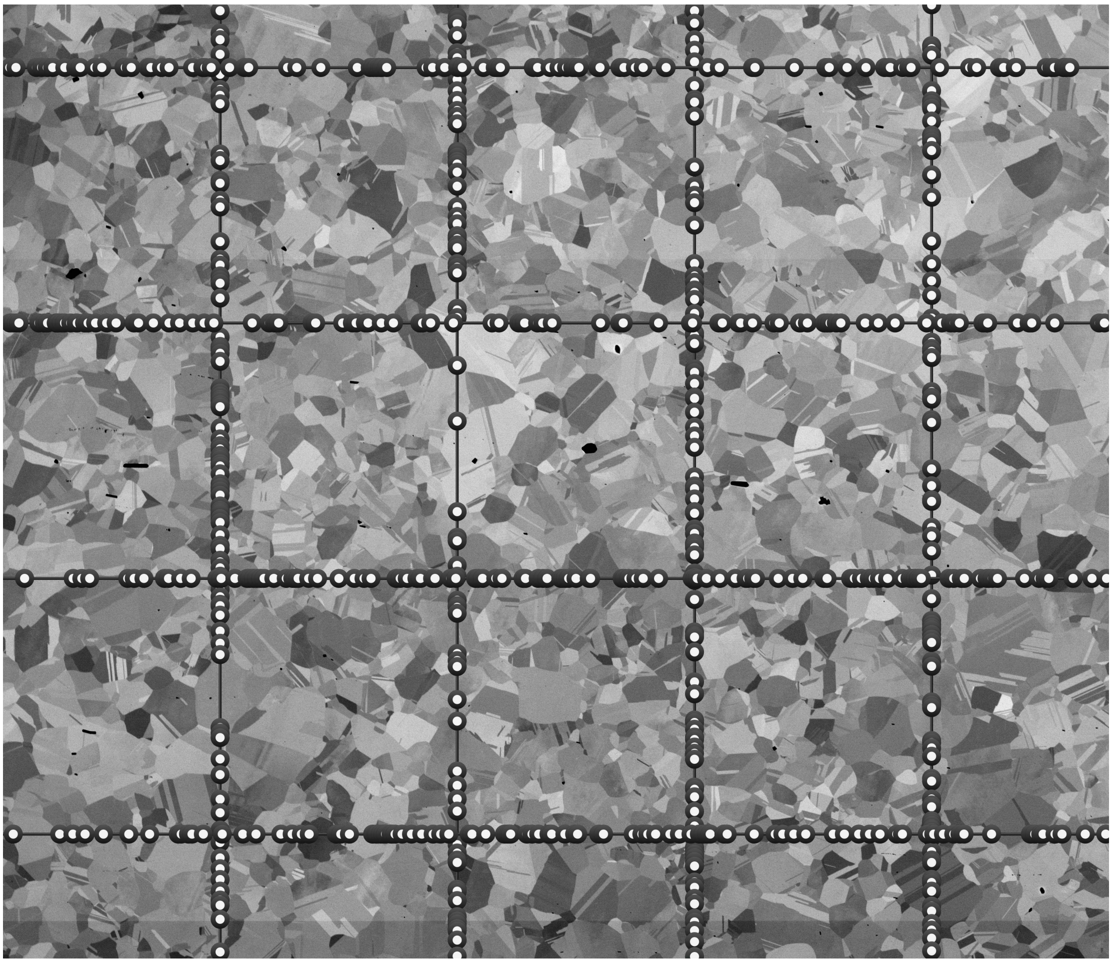

2.1. Statistische Analyse

Statistische Daten	Länge
Anzahl Objekte	715
Minimum	0,2 µm
Maximum	34,6 µm
Mittelwert	4,4 µm
Standardabweichung	4,1 µm
Schiefe	0,0
Standardabweichung (n-1)	4,1 µm
Varianz	16,8 µm²
Varianz (n-1)	16,8 µm²
Summe	3'154,9 µm

Statistische Daten	Länge
Quadratsumme	25'950,5 µm²
Kubiksumme	321'824,0 µm³

2.1.1. Chord Lenght Distribution

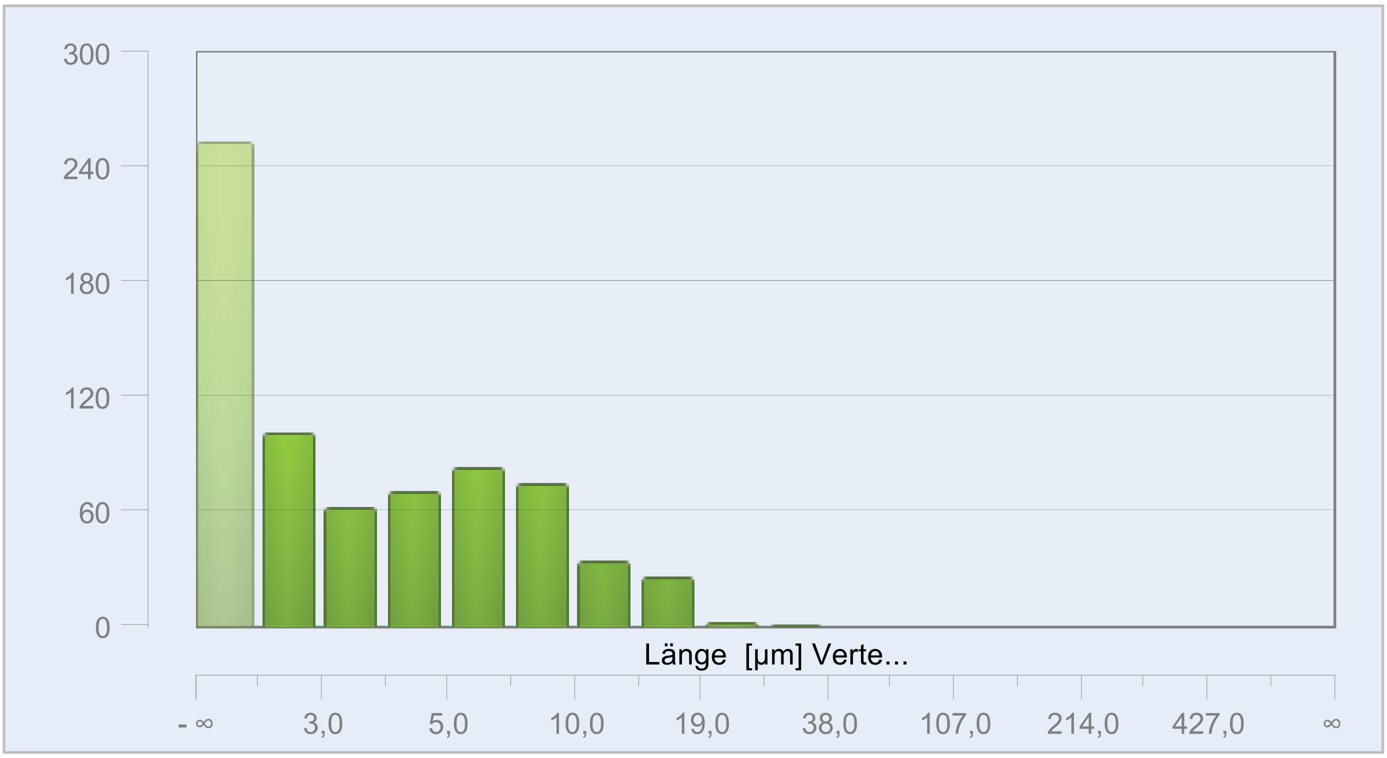

Start	Ende	Absolute Häufigkeit	Absolute Häufigkeit (kumuliert)	Relative Häufigkeit [%]	Relative Häufigkeit (kumuliert) [%]
	2,0 µm	252	252	35	35
2,0 µm	3,0 µm	102	354	14	50
3,0 µm	4,0 µm	63	417	9	58
4,0 µm	5,0 µm	71	488	10	68
5,0 µm	7,0 µm	83	571	12	80
7,0 µm	10,0 µm	76	647	11	90
10,0 µm	13,0 µm	35	682	5	95
13,0 µm	19,0 µm	27	709	4	99
19,0 µm	27,0 µm	4	713	1	100
27,0 µm	38,0 µm	2	715	0	100
38,0 µm	75,0 µm	0	715	0	100
75,0 µm	107,0 µm	0	715	0	100
107,0 µm	151,0 µm	0	715	0	100
151,0 µm	214,0 µm	0	715	0	100
214,0 µm	302,0 µm	0	715	0	100
302,0 µm	427,0 µm	0	715	0	100
427,0 µm	600,0 µm	0	715	0	100
600,0 µm		0	715	0	100

3. Single Result 2 (CrCoNi - ASTM E 112_CrCoNi_homogenized_8.1mmSW_900°C_60min_00151)

Mittlere Sehnenlänge	4,8 µm
Korngröße (ASTM)	12,1
Korngröße (G643)	12,1
Kornstreckung	90,5 %

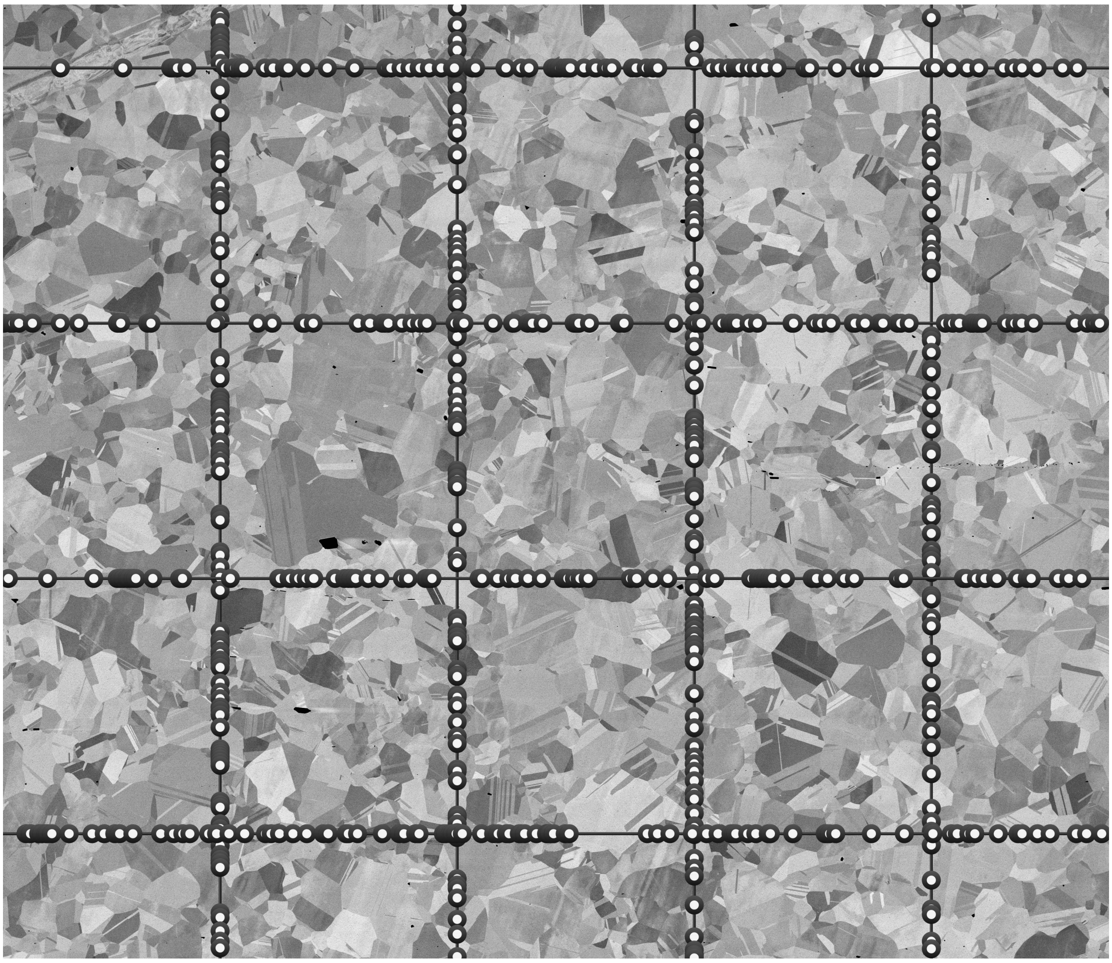

3.1. Statistische Analyse

Statistische Daten	Länge
Anzahl Objekte	656
Minimum	0,1 µm
Maximum	29,9 µm
Mittelwert	4,8 µm
Standardabweichung	4,6 µm
Schiefe	0,0
Standardabweichung (n-1)	4,6 µm
Varianz	21,2 µm²
Varianz (n-1)	21,2 µm²
Summe	3'152,9 µm
Quadratsumme	29'060,5 µm²
Kubiksumme	390'466,3 µm³

3.1.1. Chord Lenght Distribution

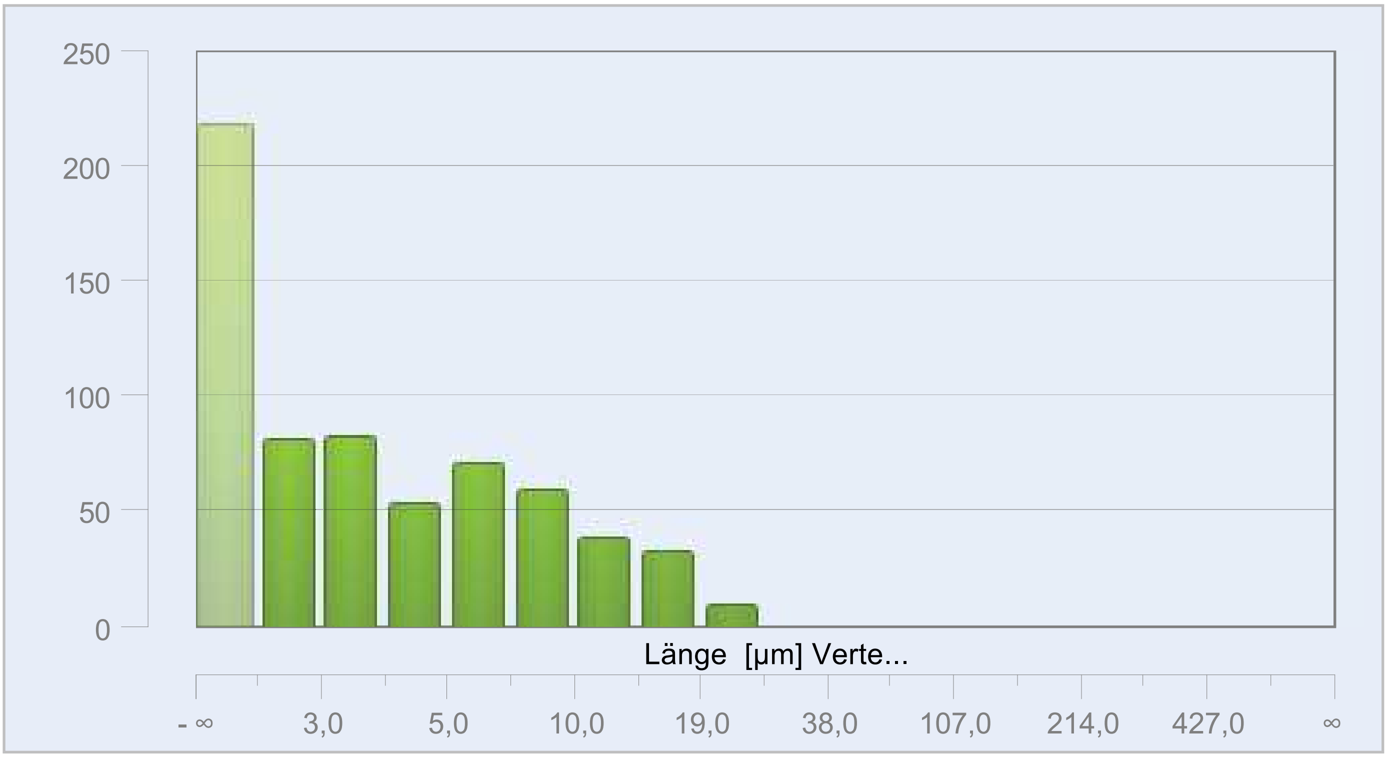

Start	Ende	Absolute Häufigkeit	Absolute Häufigkeit (kumuliert)	Relative Häufigkeit [%]	Relative Häufigkeit (kumuliert) [%]
	2,0 µm	218	218	33	33
2,0 µm	3,0 µm	82	300	12	46
3,0 µm	4,0 µm	83	383	13	58
4,0 µm	5,0 µm	55	438	8	67
5,0 µm	7,0 µm	72	510	11	78
7,0 µm	10,0 µm	60	570	9	87
10,0 µm	13,0 µm	40	610	6	93
13,0 µm	19,0 µm	34	644	5	98
19,0 µm	27,0 µm	11	655	2	100
27,0 µm	38,0 µm	1	656	0	100
38,0 µm	75,0 µm	0	656	0	100
75,0 µm	107,0 µm	0	656	0	100
107,0 µm	151,0 µm	0	656	0	100
151,0 µm	214,0 µm	0	656	0	100
214,0 µm	302,0 µm	0	656	0	100
302,0 µm	427,0 µm	0	656	0	100
427,0 µm	600,0 µm	0	656	0	100
600,0 µm		0	656	0	100

4. Single Result 3 (CrCoNi - ASTM E 112_CrCoNi_homogenized_8.1mmSW_900°C_60min_00152)

Mittlere Sehnenlänge	4,7 µm
Korngröße (ASTM)	12,2
Korngröße (G643)	12,1
Kornstreckung	85,5 %

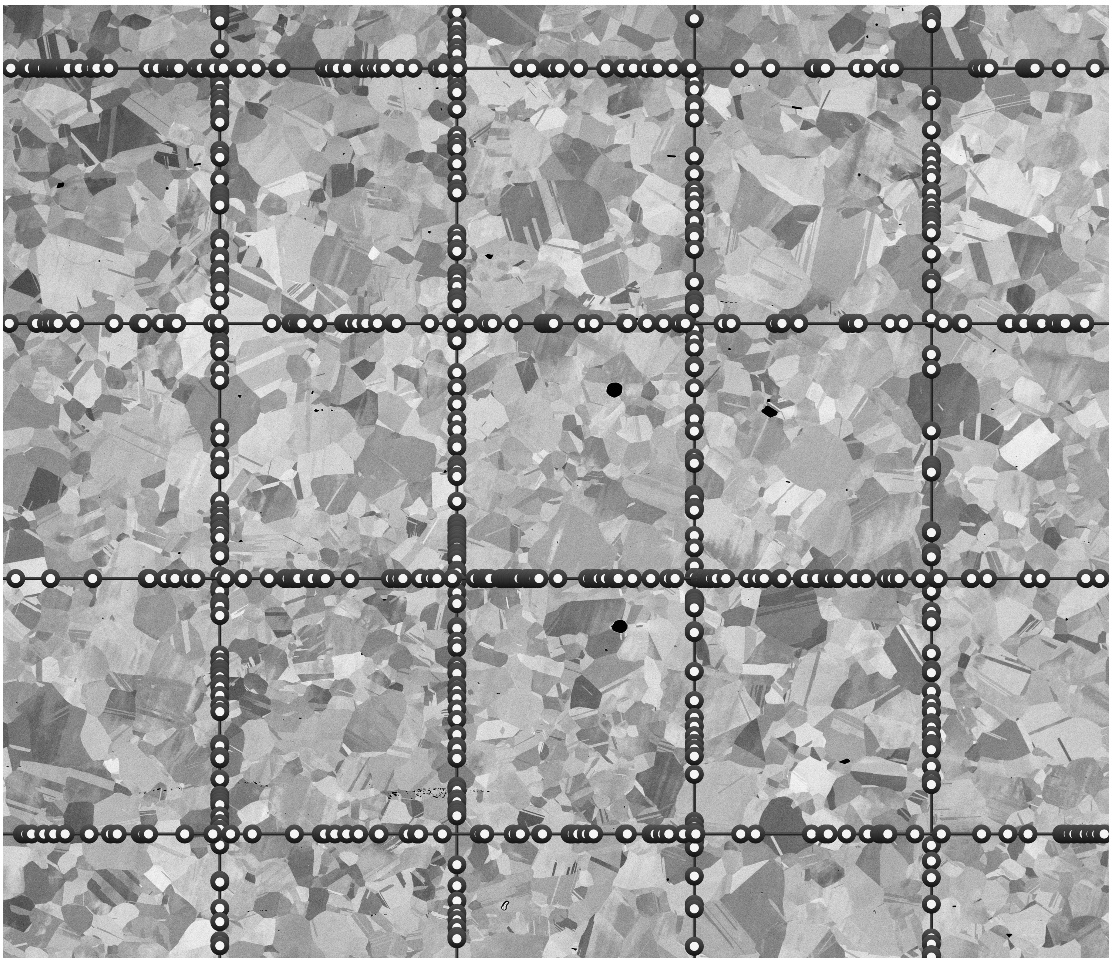

4.1. Statistische Analyse

Statistische Daten	Länge
Anzahl Objekte	669
Minimum	0,1 µm
Maximum	31,6 µm
Mittelwert	4,7 µm
Standardabweichung	4,6 µm
Schiefe	0,0
Standardabweichung (n-1)	4,6 µm
Varianz	21,4 µm²
Varianz (n-1)	21,4 µm²
Summe	3'153,3 µm
Quadratsumme	29'166,9 µm²
Kubiksumme	393'523,2 µm³

4.1.1. Chord Lenght Distribution

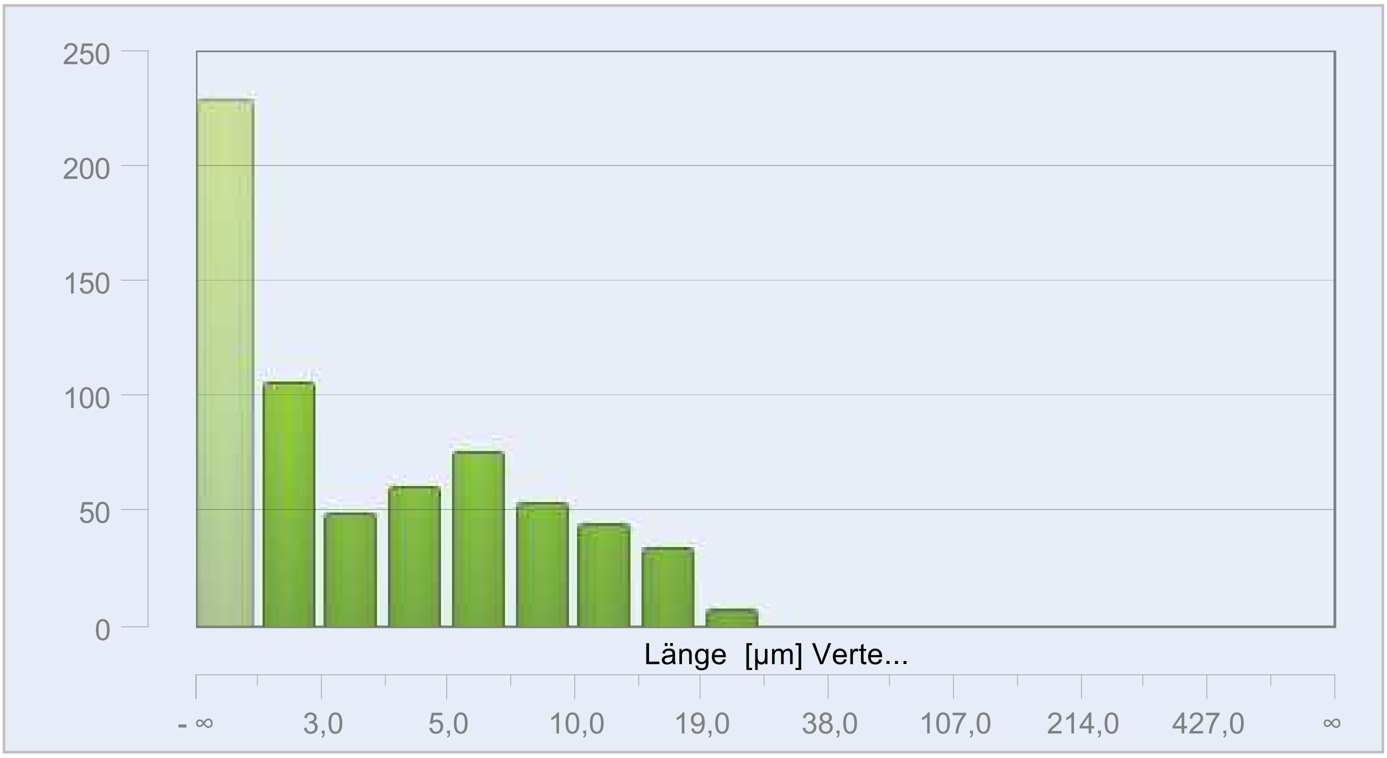

Start	Ende	Absolute Häufigkeit	Absolute Häufigkeit (kumuliert)	Relative Häufigkeit [%]	Relative Häufigkeit (kumuliert) [%]
	2,0 µm	229	229	34	34
2,0 µm	3,0 µm	106	335	16	50
3,0 µm	4,0 µm	50	385	7	58
4,0 µm	5,0 µm	62	447	9	67
5,0 µm	7,0 µm	77	524	12	78
7,0 µm	10,0 µm	55	579	8	87
10,0 µm	13,0 µm	45	624	7	93
13,0 µm	19,0 µm	35	659	5	99
19,0 µm	27,0 µm	9	668	1	100
27,0 µm	38,0 µm	1	669	0	100
38,0 µm	75,0 µm	0	669	0	100
75,0 µm	107,0 µm	0	669	0	100
107,0 µm	151,0 µm	0	669	0	100
151,0 µm	214,0 µm	0	669	0	100
214,0 µm	302,0 µm	0	669	0	100
302,0 µm	427,0 µm	0	669	0	100
427,0 µm	600,0 µm	0	669	0	100
600,0 µm		0	669	0	100

5. Single Result 4 (CrCoNi - ASTM E 112_CrCoNi_homogenized_8.1mmSW_900°C_60min_00153)

Mittlere Sehnenlänge	5,3 µm
Korngröße (ASTM)	11,8
Korngröße (G643)	11,8
Kornstreckung	80,6 %

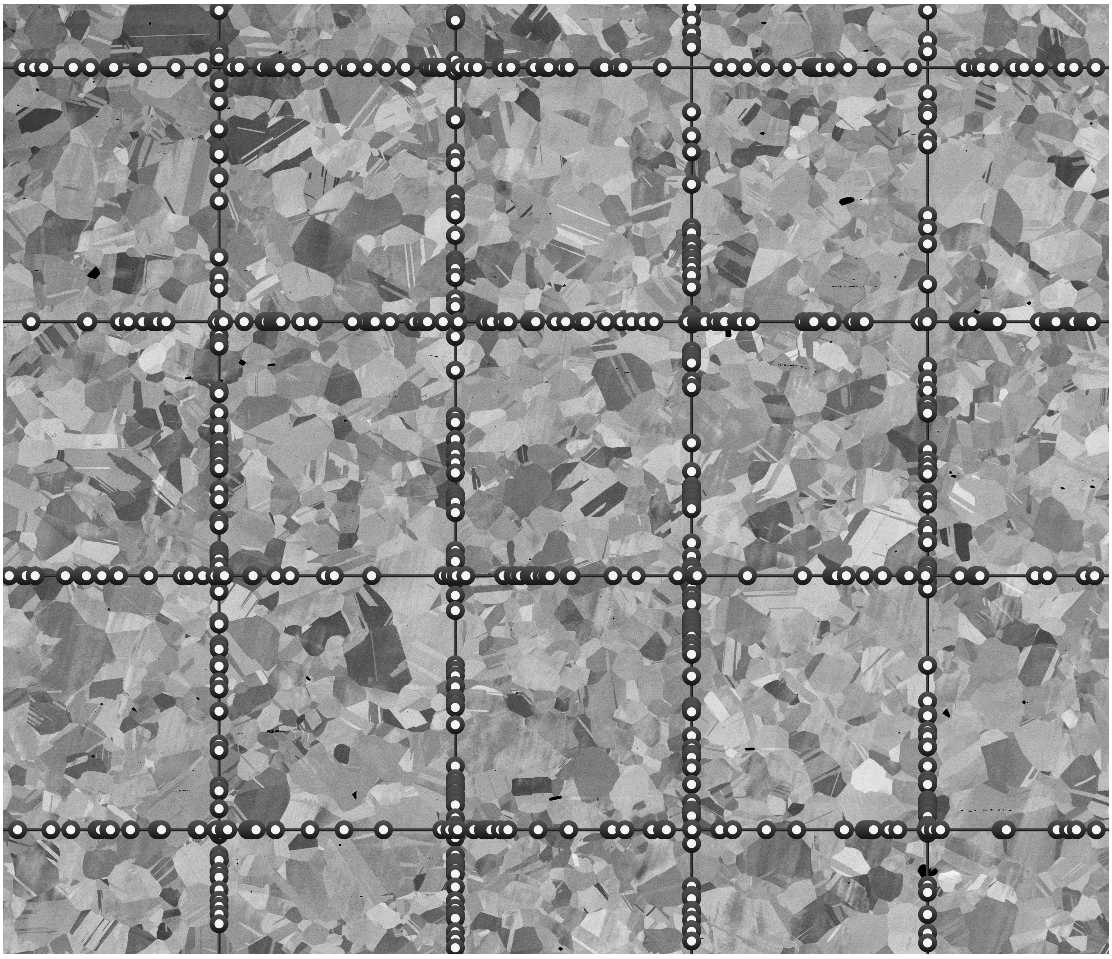

5.1. Statistische Analyse

Statistische Daten	Länge
Anzahl Objekte	595
Minimum	0,2 µm
Maximum	29,3 µm
Mittelwert	5,3 µm
Standardabweichung	5,1 µm
Schiefe	0,0
Standardabweichung (n-1)	5,1 µm
Varianz	26,1 µm²
Varianz (n-1)	26,1 µm²
Summe	3'149,1 µm
Quadratsumme	32'176,4 µm²
Kubiksumme	467'000,1 µm³

5.1.1. Chord Lenght Distribution

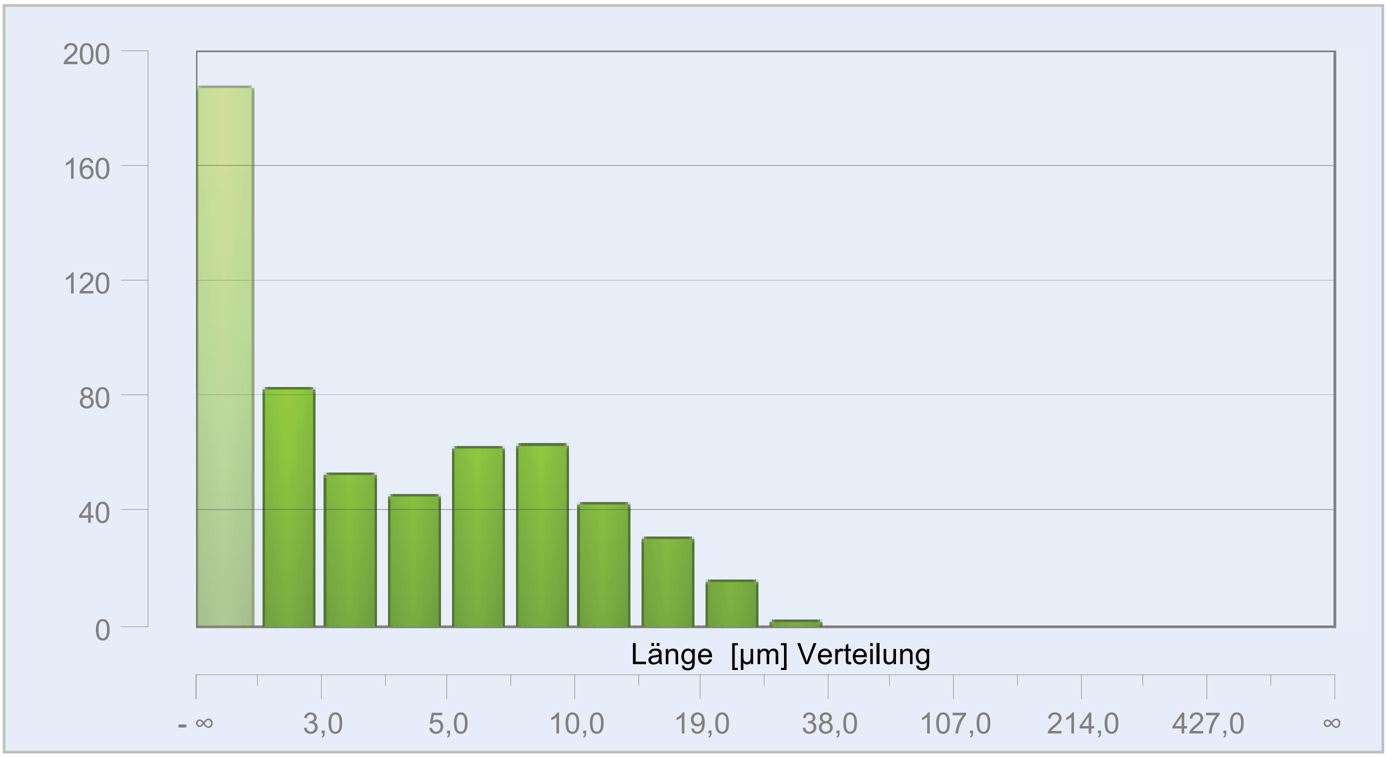

Start	Ende	Absolute Häufigkeit	Absolute Häufigkeit (kumuliert)	Relative Häufigkeit [%]	Relative Häufigkeit (kumuliert) [%]
	2,0 µm	188	188	32	32
2,0 µm	3,0 µm	83	271	14	46
3,0 µm	4,0 µm	54	325	9	55
4,0 µm	5,0 µm	47	372	8	63
5,0 µm	7,0 µm	63	435	11	73
7,0 µm	10,0 µm	64	499	11	84
10,0 µm	13,0 µm	44	543	7	91
13,0 µm	19,0 µm	32	575	5	97
19,0 µm	27,0 µm	17	592	3	99
27,0 µm	38,0 µm	3	595	1	100
38,0 µm	75,0 µm	0	595	0	100
75,0 µm	107,0 µm	0	595	0	100
107,0 µm	151,0 µm	0	595	0	100
151,0 µm	214,0 µm	0	595	0	100
214,0 µm	302,0 µm	0	595	0	100
302,0 µm	427,0 µm	0	595	0	100
427,0 µm	600,0 µm	0	595	0	100
600,0 µm		0	595	0	100
