# Supplementary material for: Benchmark dataset of the effect of grain size on strength in the single-phase FCC CrCoNi medium entropy alloy
Source: Data Brief. 2019 Oct 1;27:104592. doi: 10.1016/j.dib.2019.104592 (PMC6812030; doi:10.1016/j.dib.2019.104592)
Supplement: Multimedia component 1 [file mmc1.zip › CrCoNi_1173K_60min/CrCoNi_1173K_60min_d=8.7μm.pdf]

Analysebericht

Aug 29, 2017 3:02:38 PM

powered by imagic.ch

1. cumulative Result 1

Number of images	4
Grain size (ASTM)	10.4
Grain size (G643)	10.4
Grain stretching	95.2 %
Mean chord length	8.6 µm

2. Single Result 1 (CrCoNi Twins grain size_ASTM 900C 1h_00207)

Mean chord length	7.9 µm
Grain size (ASTM)	10.7
Grain size (G643)	10.7
Grain stretching	96.8 %

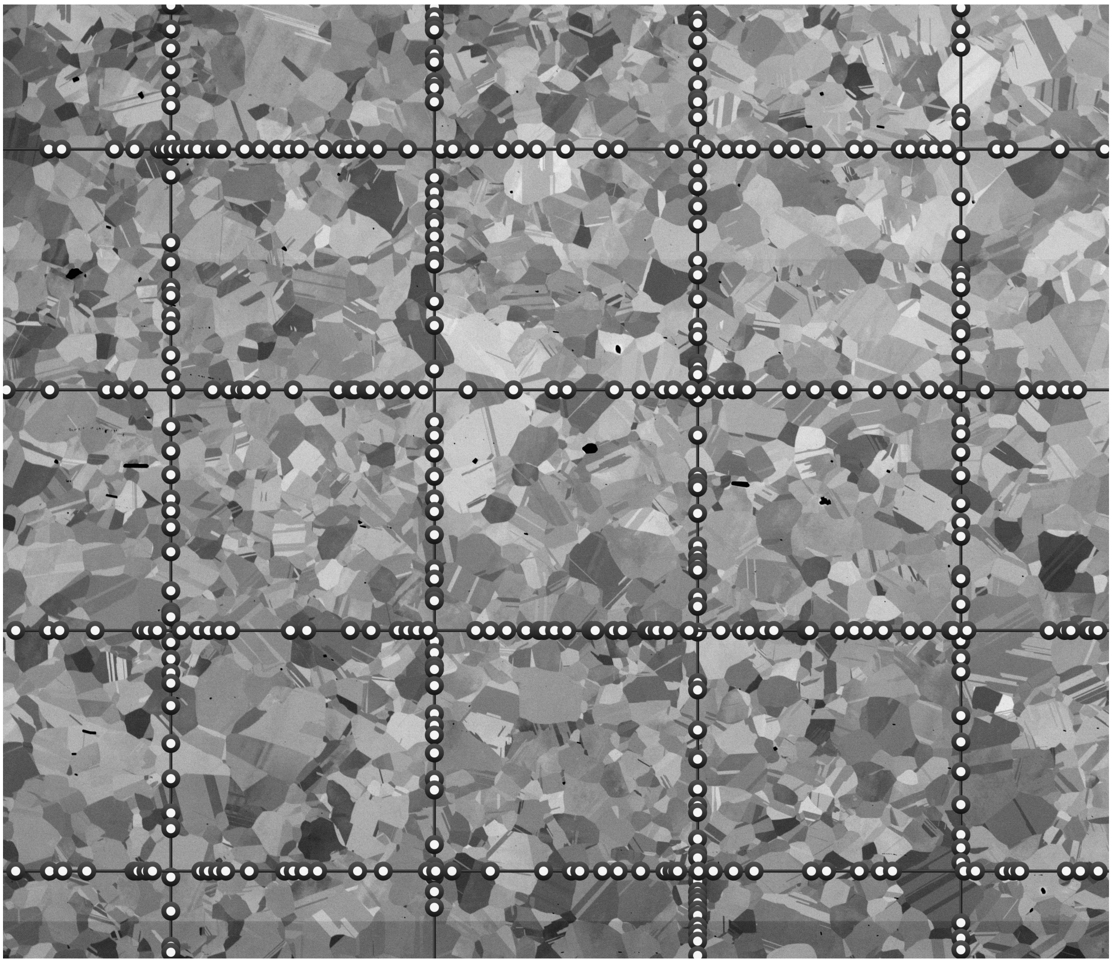

2.1. Statistical Analysis

Statistical Data	Length
Object Count	401
Minimum	0.4 µm
Maximum	31.3 µm
Average	7.9 µm
Standard deviation	5.4 µm
Skewness	0.0
Standard deviation (n-1)	5.4 µm
Variance	29.1 µm²
Variance (n-1)	29.1 µm²
Sum	3'149.6 µm

Statistical Data	Length
Sum of squares	36'393.0 µm²
Sum of cubes	547'445.2 µm³

2.1.1. Chord Lenght Distribution

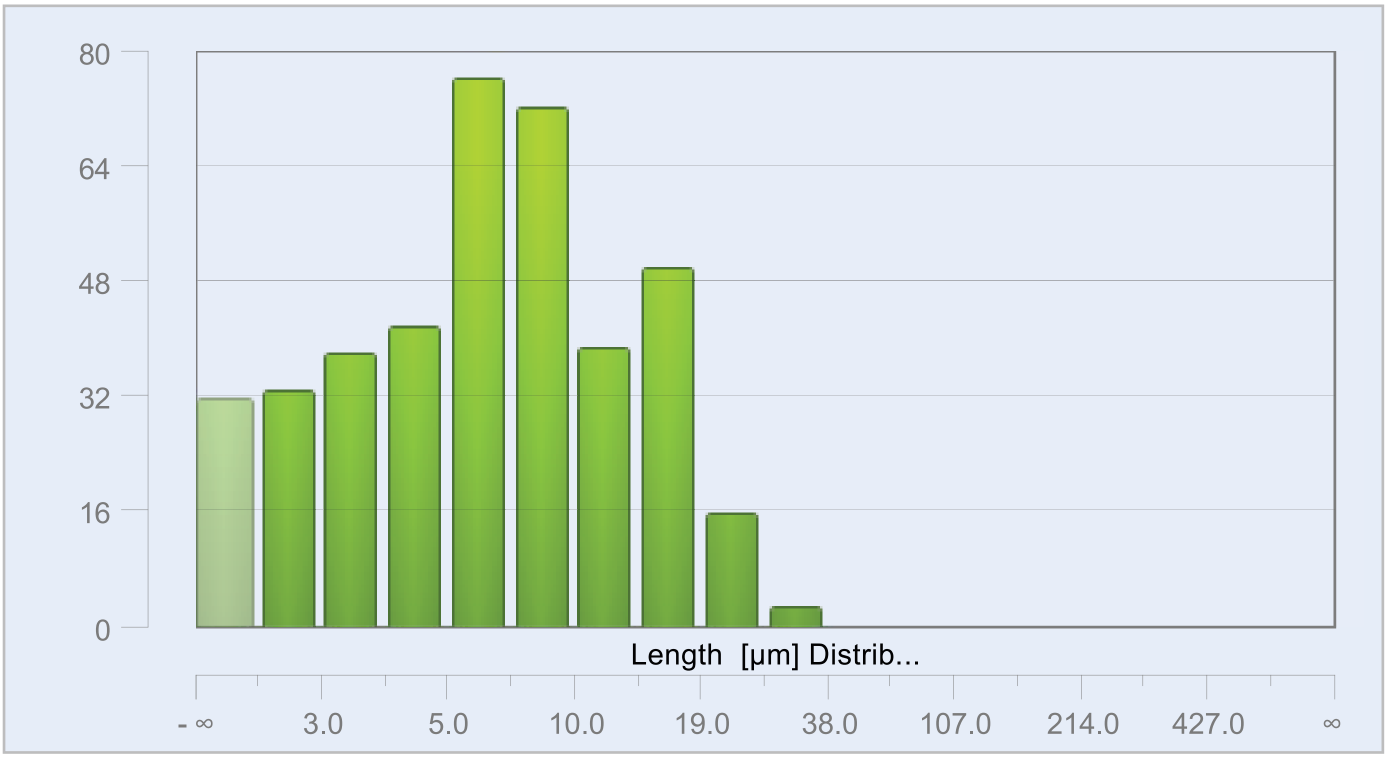

Start	End	Absolute Frequency	Absolute Frequency (accumulated)	Relative Frequency [%]	Relative Frequency (accumulated) [%]
	2.0 µm	32	32	8	8
2.0 µm	3.0 µm	33	65	8	16
3.0 µm	4.0 µm	38	103	9	26
4.0 µm	5.0 µm	42	145	10	36
5.0 µm	7.0 µm	76	221	19	55
7.0 µm	10.0 µm	72	293	18	73
10.0 µm	13.0 µm	39	332	10	83
13.0 µm	19.0 µm	50	382	12	95
19.0 µm	27.0 µm	16	398	4	99
27.0 µm	38.0 µm	3	401	1	100
38.0 µm	75.0 µm	0	401	0	100
75.0 µm	107.0 µm	0	401	0	100
107.0 µm	151.0 µm	0	401	0	100
151.0 µm	214.0 µm	0	401	0	100
214.0 µm	302.0 µm	0	401	0	100
302.0 µm	427.0 µm	0	401	0	100
427.0 µm	600.0 µm	0	401	0	100
600.0 µm		0	401	0	100

3. Single Result 2 (CrCoNi Twins grain size_ASTM 900C 1h_00208)

Mean chord length	8.8 µm
Grain size (ASTM)	10.4
Grain size (G643)	10.3
Grain stretching	94.7 %

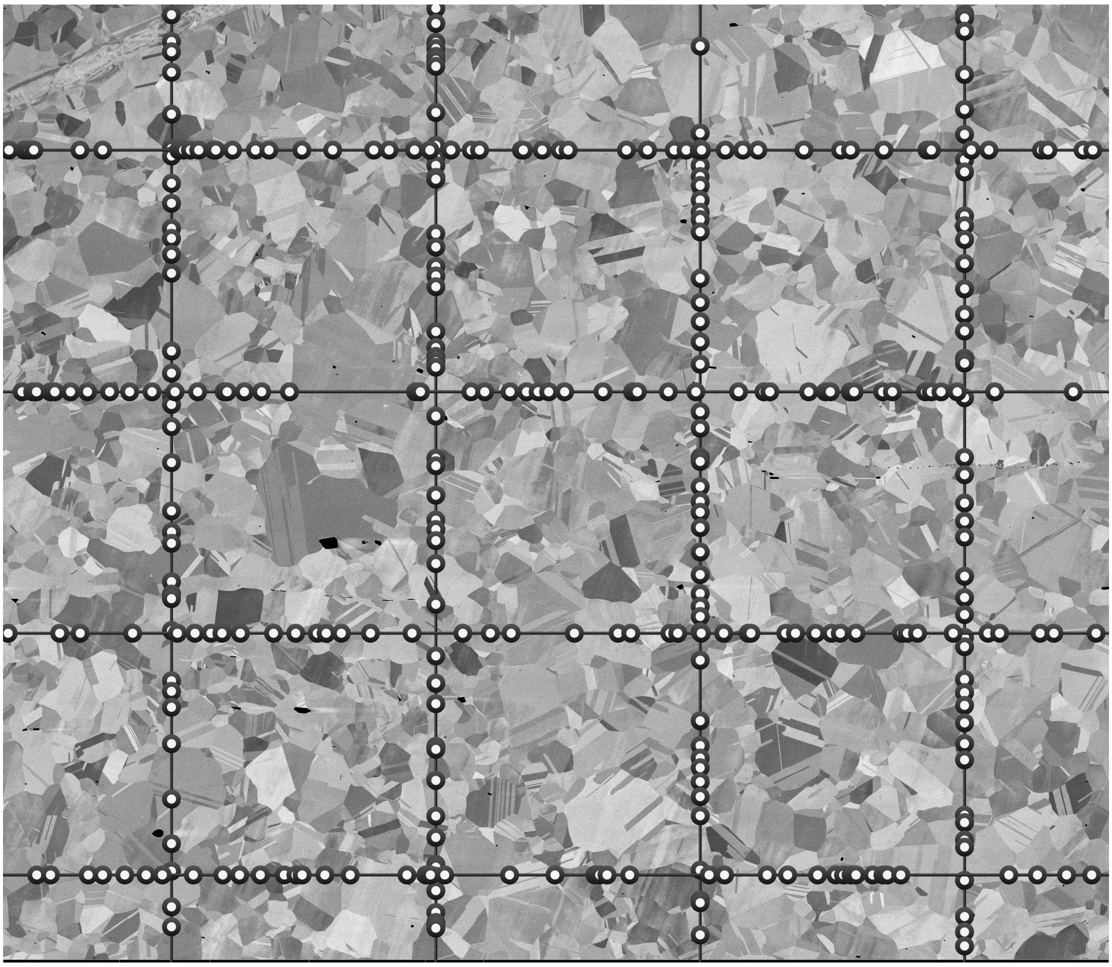

3.1. Statistical Analysis

Statistical Data	Length
Object Count	358
Minimum	0.7 µm
Maximum	48.2 µm
Average	8.8 µm
Standard deviation	6.4 µm
Skewness	0.0
Standard deviation (n-1)	6.5 µm
Variance	41.5 µm²
Variance (n-1)	41.6 µm²
Sum	3'158.3 µm
Sum of squares	42'714.7 µm²
Sum of cubes	813'360.3 µm³

3.1.1. Chord Lenght Distribution

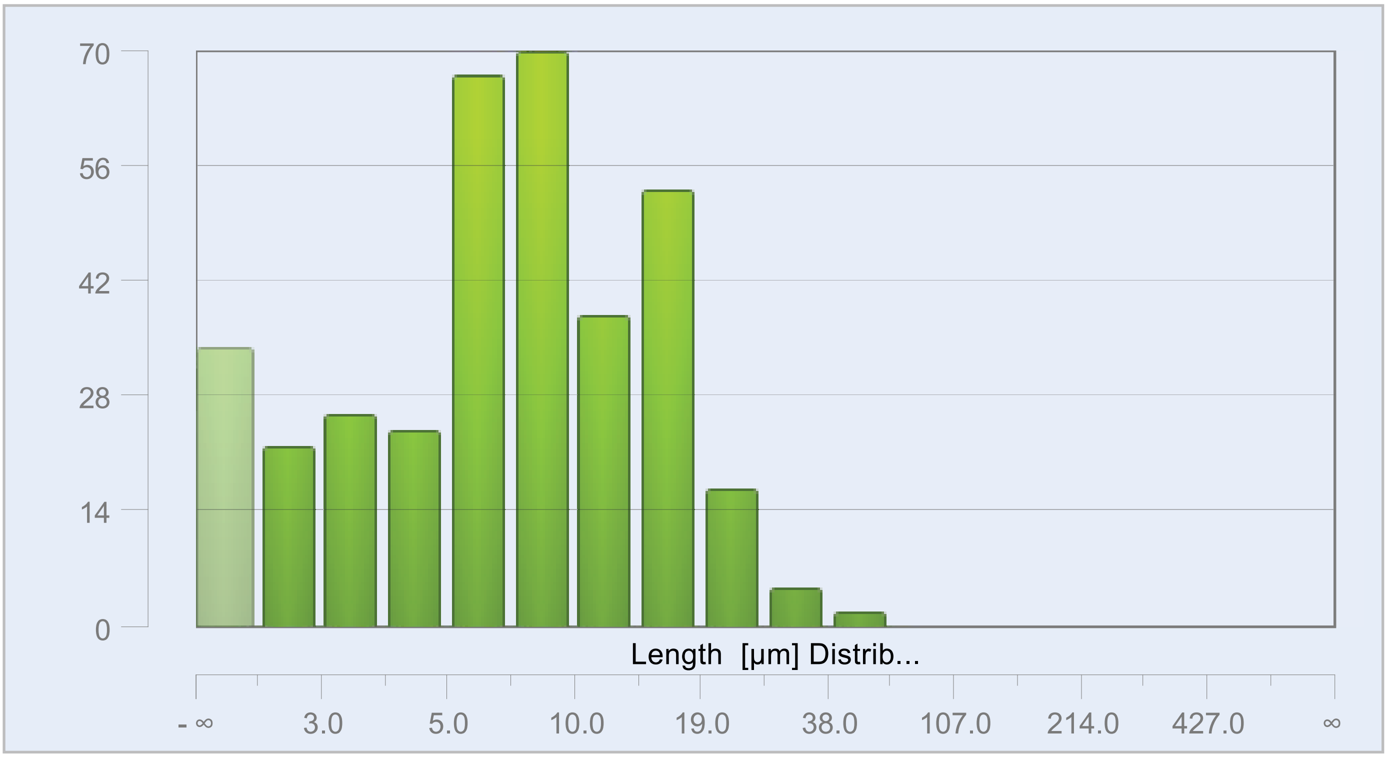

Start	End	Absolute Frequency	Absolute Frequency (accumulated)	Relative Frequency [%]	Relative Frequency (accumulated) [%]
	2.0 µm	34	34	9	9
2.0 µm	3.0 µm	22	56	6	16
3.0 µm	4.0 µm	26	82	7	23
4.0 µm	5.0 µm	24	106	7	30
5.0 µm	7.0 µm	67	173	19	48
7.0 µm	10.0 µm	70	243	20	68
10.0 µm	13.0 µm	38	281	11	78
13.0 µm	19.0 µm	53	334	15	93
19.0 µm	27.0 µm	17	351	5	98
27.0 µm	38.0 µm	5	356	1	99
38.0 µm	75.0 µm	2	358	1	100
75.0 µm	107.0 µm	0	358	0	100
107.0 µm	151.0 µm	0	358	0	100
151.0 µm	214.0 µm	0	358	0	100
214.0 µm	302.0 µm	0	358	0	100
302.0 µm	427.0 µm	0	358	0	100
427.0 µm	600.0 µm	0	358	0	100
600.0 µm		0	358	0	100

4. Single Result 3 (CrCoNi Twins grain size_ASTM 900C 1h_00209)

Mean chord length	8.8 µm
Grain size (ASTM)	10.4
Grain size (G643)	10.3
Grain stretching	93.4 %

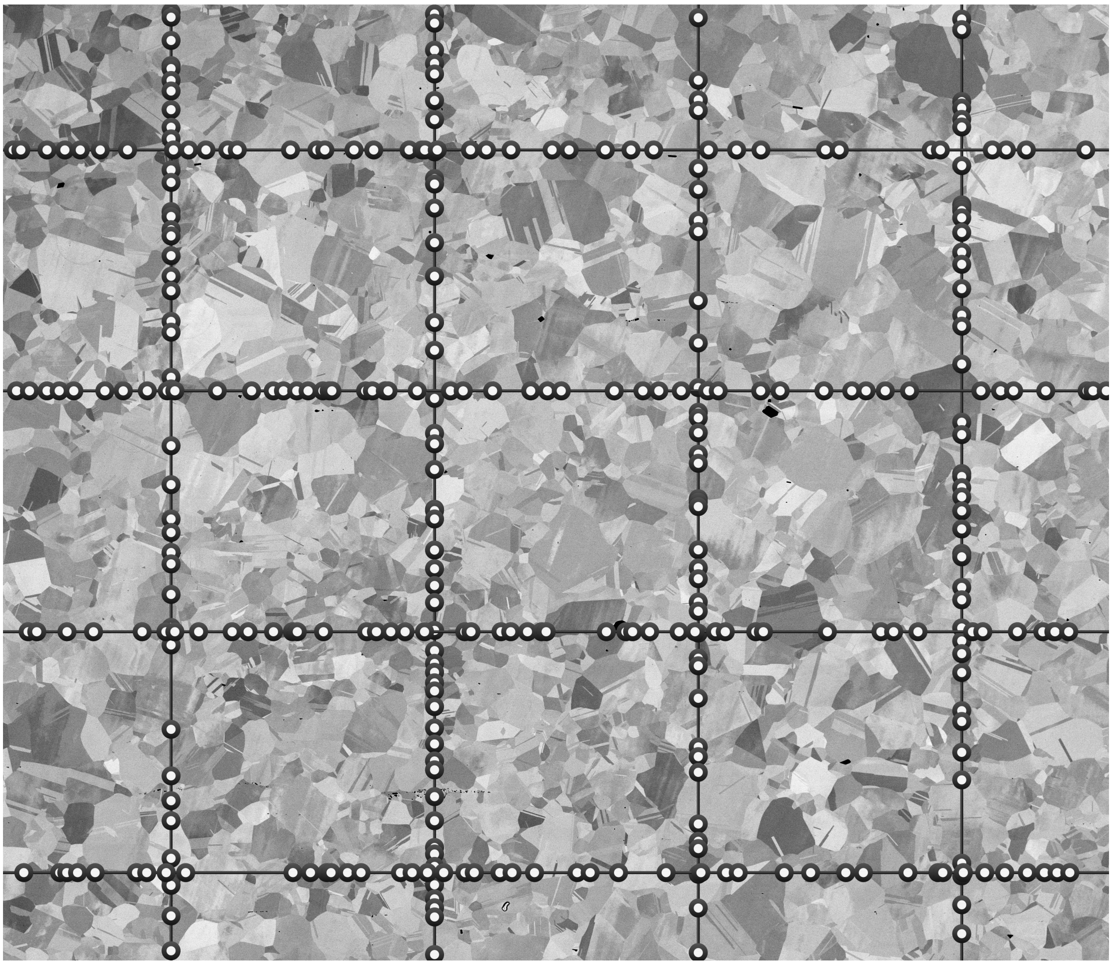

4.1. Statistical Analysis

Statistical Data	Length
Object Count	358
Minimum	0.4 µm
Maximum	41.0 µm
Average	8.8 µm
Standard deviation	6.0 µm
Skewness	0.0
Standard deviation (n-1)	6.1 µm
Variance	36.5 µm²
Variance (n-1)	36.6 µm²
Sum	3'151.7 µm
Sum of squares	40'820.9 µm²
Sum of cubes	714'447.5 µm³

4.1.1. Chord Lenght Distribution

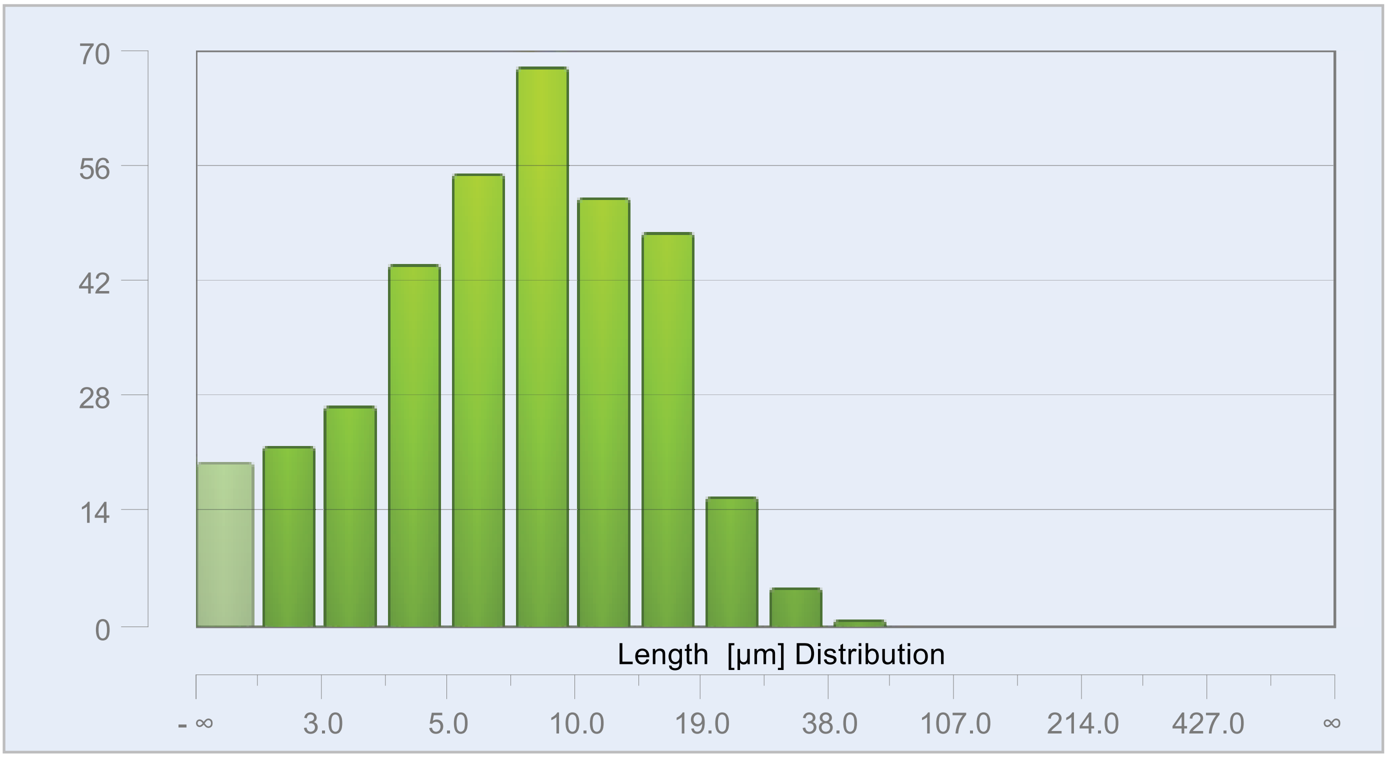

Start	End	Absolute Frequency	Absolute Frequency (accumulated)	Relative Frequency [%]	Relative Frequency (accumulated) [%]
	2.0 µm	20	20	6	6
2.0 µm	3.0 µm	22	42	6	12
3.0 µm	4.0 µm	27	69	8	19
4.0 µm	5.0 µm	44	113	12	32
5.0 µm	7.0 µm	55	168	15	47
7.0 µm	10.0 µm	68	236	19	66
10.0 µm	13.0 µm	52	288	15	80
13.0 µm	19.0 µm	48	336	13	94
19.0 µm	27.0 µm	16	352	4	98
27.0 µm	38.0 µm	5	357	1	100
38.0 µm	75.0 µm	1	358	0	100
75.0 µm	107.0 µm	0	358	0	100
107.0 µm	151.0 µm	0	358	0	100
151.0 µm	214.0 µm	0	358	0	100
214.0 µm	302.0 µm	0	358	0	100
302.0 µm	427.0 µm	0	358	0	100
427.0 µm	600.0 µm	0	358	0	100
600.0 µm		0	358	0	100

5. Single Result 4 (CrCoNi Twins grain size_ASTM 900C 1h_00210)

Mean chord length	9.1 µm
Grain size (ASTM)	10.3
Grain size (G643)	10.2
Grain stretching	95.7 %

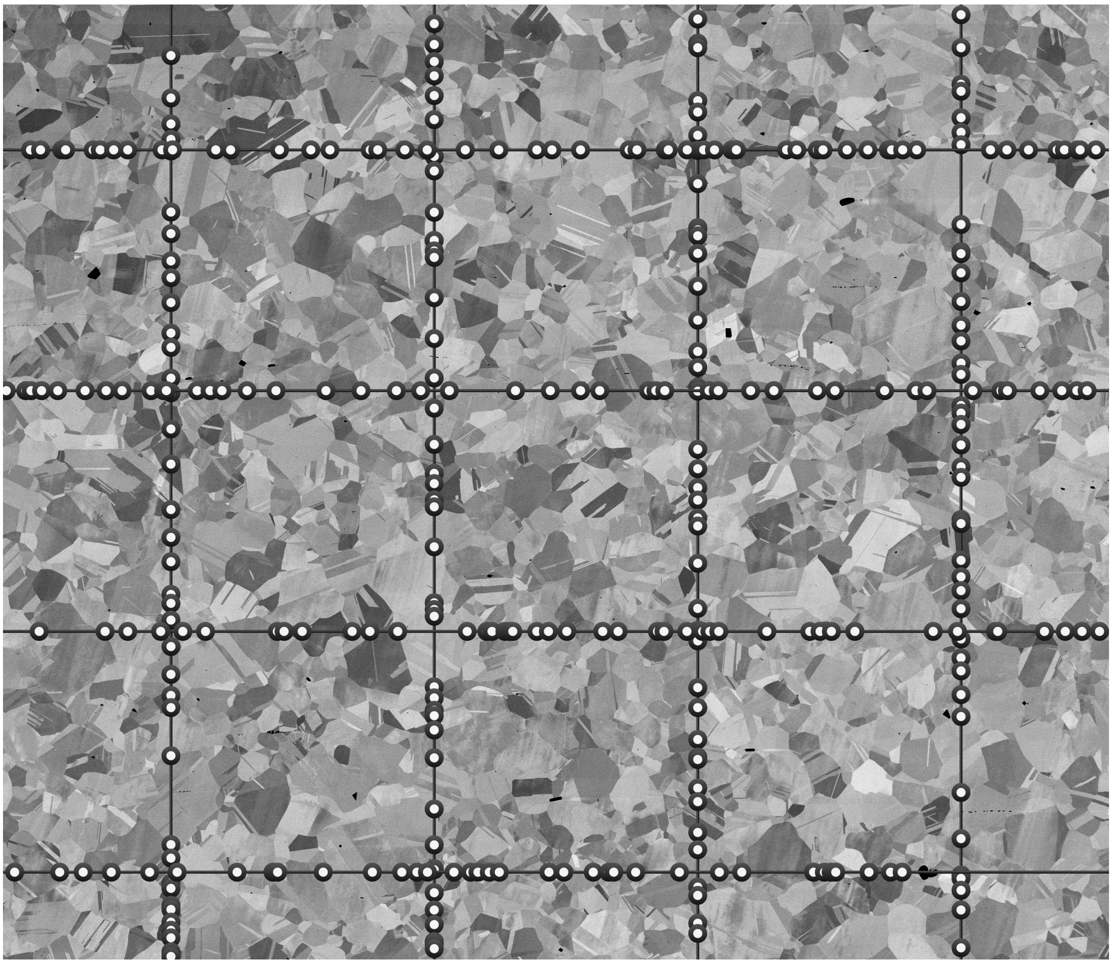

5.1. Statistical Analysis

Statistical Data	Length
Object Count	348
Minimum	0.4 µm
Maximum	34.0 µm
Average	9.1 µm
Standard deviation	6.1 µm
Skewness	0.0
Standard deviation (n-1)	6.1 µm
Variance	37.4 µm²
Variance (n-1)	37.5 µm²
Sum	3'152.9 µm
Sum of squares	41'591.5 µm²
Sum of cubes	704'486.4 µm³

5.1.1. Chord Lenght Distribution

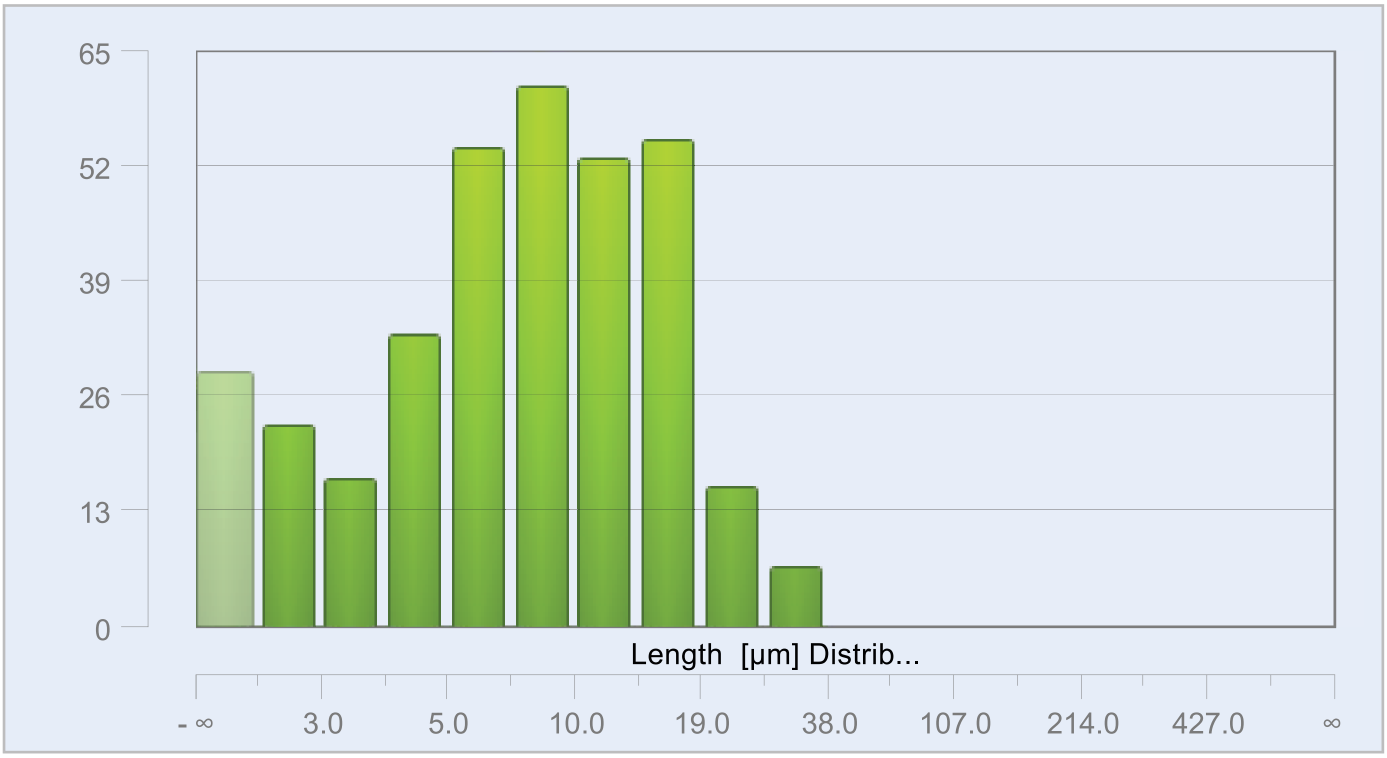

Start	End	Absolute Frequency	Absolute Frequency (accumulated)	Relative Frequency [%]	Relative Frequency (accumulated) [%]
	2.0 µm	29	29	8	8
2.0 µm	3.0 µm	23	52	7	15
3.0 µm	4.0 µm	17	69	5	20
4.0 µm	5.0 µm	33	102	9	29
5.0 µm	7.0 µm	54	156	16	45
7.0 µm	10.0 µm	61	217	18	62
10.0 µm	13.0 µm	53	270	15	78
13.0 µm	19.0 µm	55	325	16	93
19.0 µm	27.0 µm	16	341	5	98
27.0 µm	38.0 µm	7	348	2	100
38.0 µm	75.0 µm	0	348	0	100
75.0 µm	107.0 µm	0	348	0	100
107.0 µm	151.0 µm	0	348	0	100
151.0 µm	214.0 µm	0	348	0	100
214.0 µm	302.0 µm	0	348	0	100
302.0 µm	427.0 µm	0	348	0	100
427.0 µm	600.0 µm	0	348	0	100
600.0 µm		0	348	0	100
