# Supplementary material for: Benchmark dataset of the effect of grain size on strength in the single-phase FCC CrCoNi medium entropy alloy
Source: Data Brief. 2019 Oct 1;27:104592. doi: 10.1016/j.dib.2019.104592 (PMC6812030; doi:10.1016/j.dib.2019.104592)
Supplement: Multimedia component 1 [file mmc1.zip › CrCoNi_1273K_180min/CrCoNi_1273K_180min_c=18.5μm.pdf]

Analysebericht

Nov 2, 2017 2:28:43 PM

powered by imagic.ch

1. cumulative Result 1

Number of images	4
Grain size (ASTM)	8.2
Grain size (G643)	8.2
Grain stretching	89.8 %
Mean chord length	18.5 µm

2. Single Result 1 (CrCoNi - ASTM E 112_CrCoNi_homogenized_8.1mmSW_1000°C_180min_00080)

Mean chord length	18.8 µm
Grain size (ASTM)	8.2
Grain size (G643)	8.1
Grain stretching	98.4 %

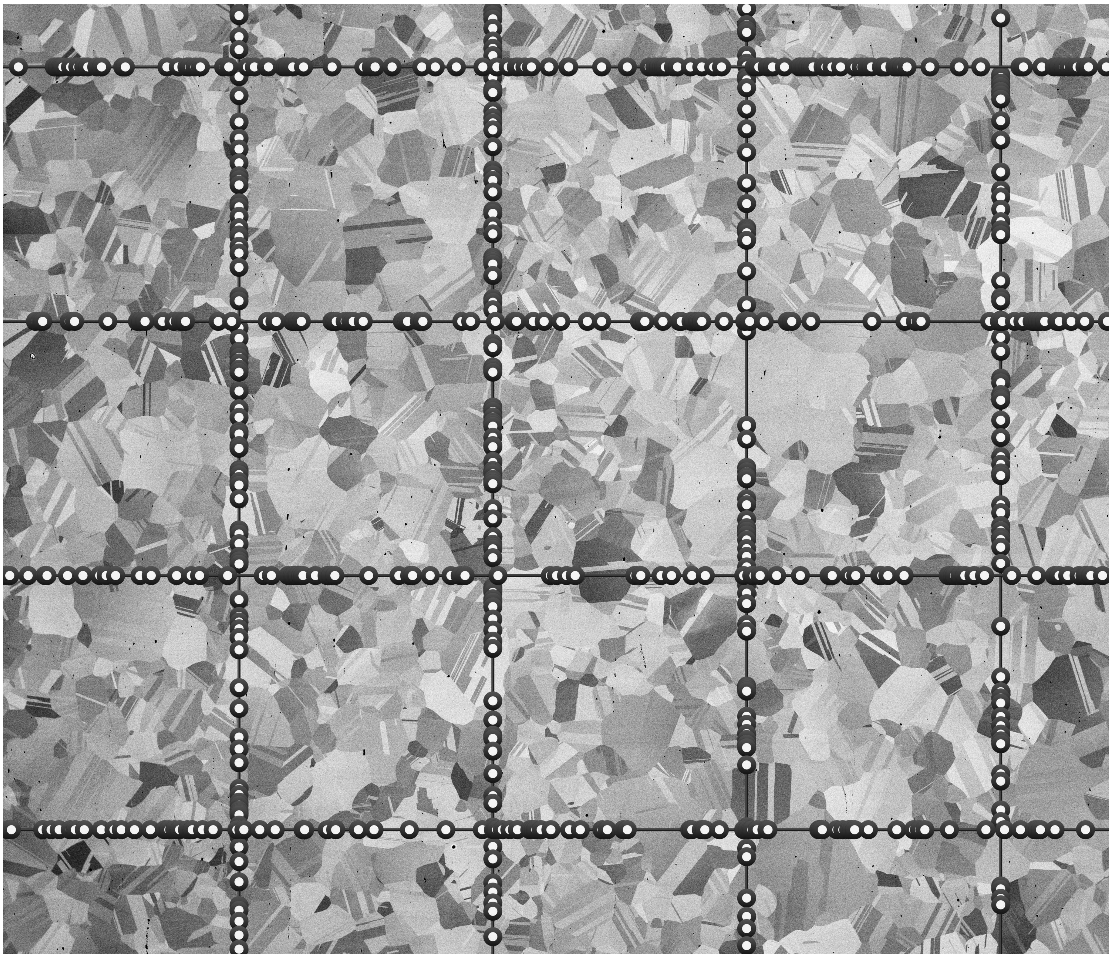

2.1. Statistical Analysis

Statistical Data	Length
Object Count	670
Minimum	0.8 µm
Maximum	144.2 µm
Average	18.8 µm
Standard deviation	18.2 µm
Skewness	0.0
Standard deviation (n-1)	18.2 µm
Variance	331.6 µm²
Variance (n-1)	332.1 µm²
Sum	12'583.5 µm
Sum of squares	458'519.9 µm²

Statistical Data	Length
Sum of cubes	25'446'995.2 µm³

2.1.1. Chord Lenght Distribution

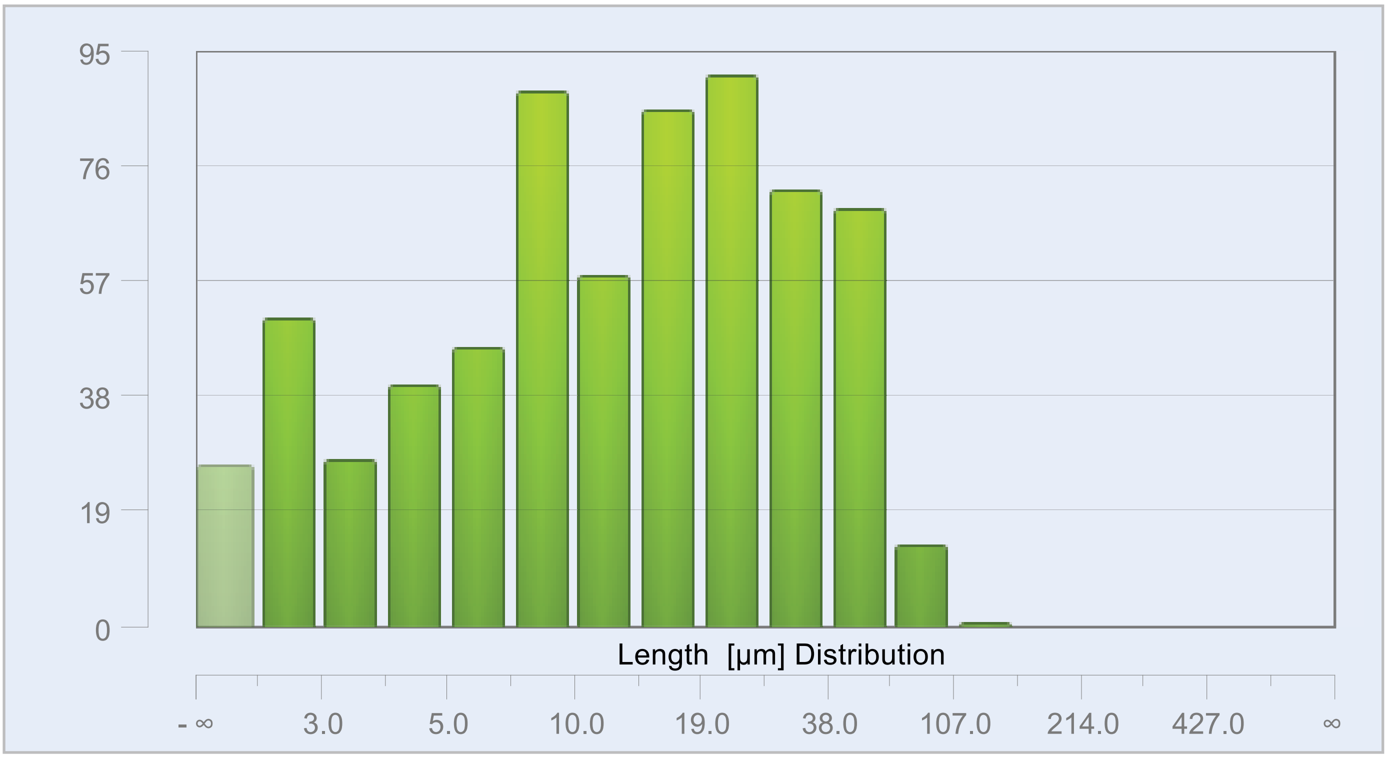

Start	End	Absolute Frequency	Absolute Frequency (accumulated)	Relative Frequency [%]	Relative Frequency (accumulated) [%]
	2.0 µm	27	27	4	4
2.0 µm	3.0 µm	51	78	8	12
3.0 µm	4.0 µm	28	106	4	16
4.0 µm	5.0 µm	40	146	6	22
5.0 µm	7.0 µm	46	192	7	29
7.0 µm	10.0 µm	88	280	13	42
10.0 µm	13.0 µm	58	338	9	50
13.0 µm	19.0 µm	85	423	13	63
19.0 µm	27.0 µm	91	514	14	77
27.0 µm	38.0 µm	72	586	11	87
38.0 µm	75.0 µm	69	655	10	98
75.0 µm	107.0 µm	14	669	2	100
107.0 µm	151.0 µm	1	670	0	100
151.0 µm	214.0 µm	0	670	0	100
214.0 µm	302.0 µm	0	670	0	100
302.0 µm	427.0 µm	0	670	0	100
427.0 µm	600.0 µm	0	670	0	100
600.0 µm		0	670	0	100

3. Single Result 2 (CrCoNi - ASTM E 112_CrCoNi_homogenized_8.1mmSW_1000°C_180min_00081)

Mean chord length	18.7 µm
Grain size (ASTM)	8.2
Grain size (G643)	8.1
Grain stretching	87.5 %

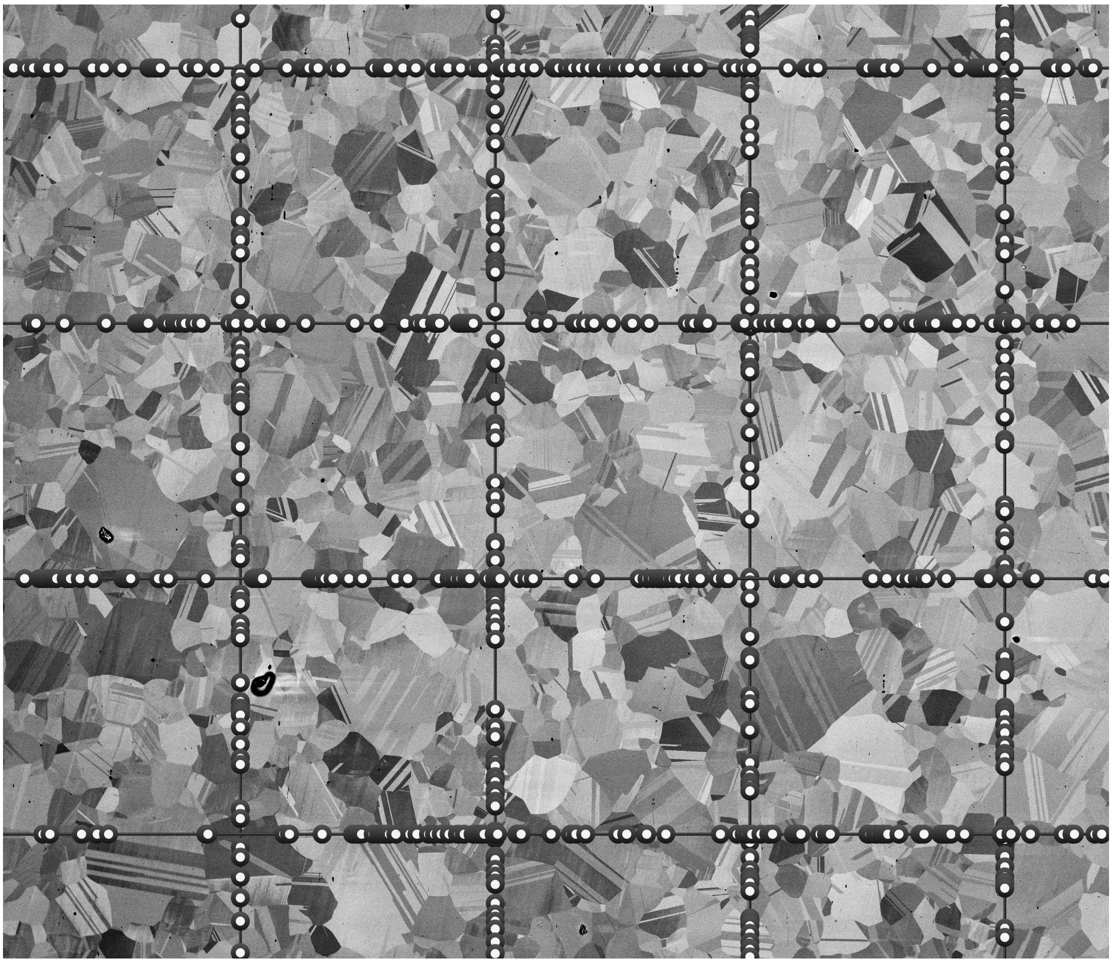

3.1. Statistical Analysis

Statistical Data	Length
Object Count	672
Minimum	0.8 µm
Maximum	148.6 µm
Average	18.7 µm
Standard deviation	19.5 µm
Skewness	0.0
Standard deviation (n-1)	19.5 µm
Variance	379.6 µm²
Variance (n-1)	380.2 µm²
Sum	12'596.2 µm
Sum of squares	491'193.8 µm²
Sum of cubes	28'978'242.7 µm³

3.1.1. Chord Lenght Distribution

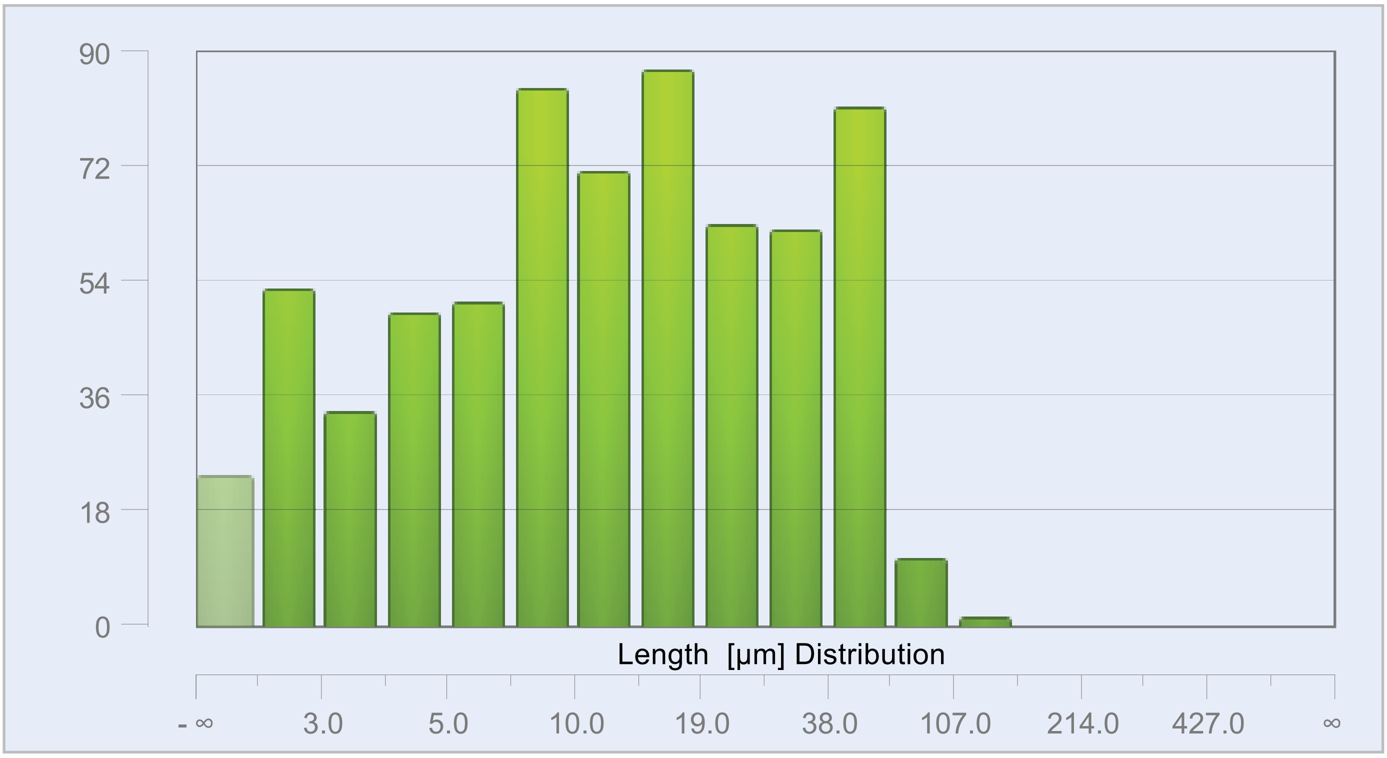

Start	End	Absolute Frequency	Absolute Frequency (accumulated)	Relative Frequency [%]	Relative Frequency (accumulated) [%]
	2.0 µm	24	24	4	4
2.0 µm	3.0 µm	53	77	8	11
3.0 µm	4.0 µm	34	111	5	17
4.0 µm	5.0 µm	49	160	7	24
5.0 µm	7.0 µm	51	211	8	31
7.0 µm	10.0 µm	84	295	12	44
10.0 µm	13.0 µm	71	366	11	54
13.0 µm	19.0 µm	87	453	13	67
19.0 µm	27.0 µm	63	516	9	77
27.0 µm	38.0 µm	62	578	9	86
38.0 µm	75.0 µm	81	659	12	98
75.0 µm	107.0 µm	11	670	2	100
107.0 µm	151.0 µm	2	672	0	100
151.0 µm	214.0 µm	0	672	0	100
214.0 µm	302.0 µm	0	672	0	100
302.0 µm	427.0 µm	0	672	0	100
427.0 µm	600.0 µm	0	672	0	100
600.0 µm		0	672	0	100

4. Single Result 3 (CrCoNi - ASTM E 112_CrCoNi_homogenized_8.1mmSW_1000°C_180min_00082)

Mean chord length	18.3 µm
Grain size (ASTM)	8.3
Grain size (G643)	8.2
Grain stretching	85.4 %

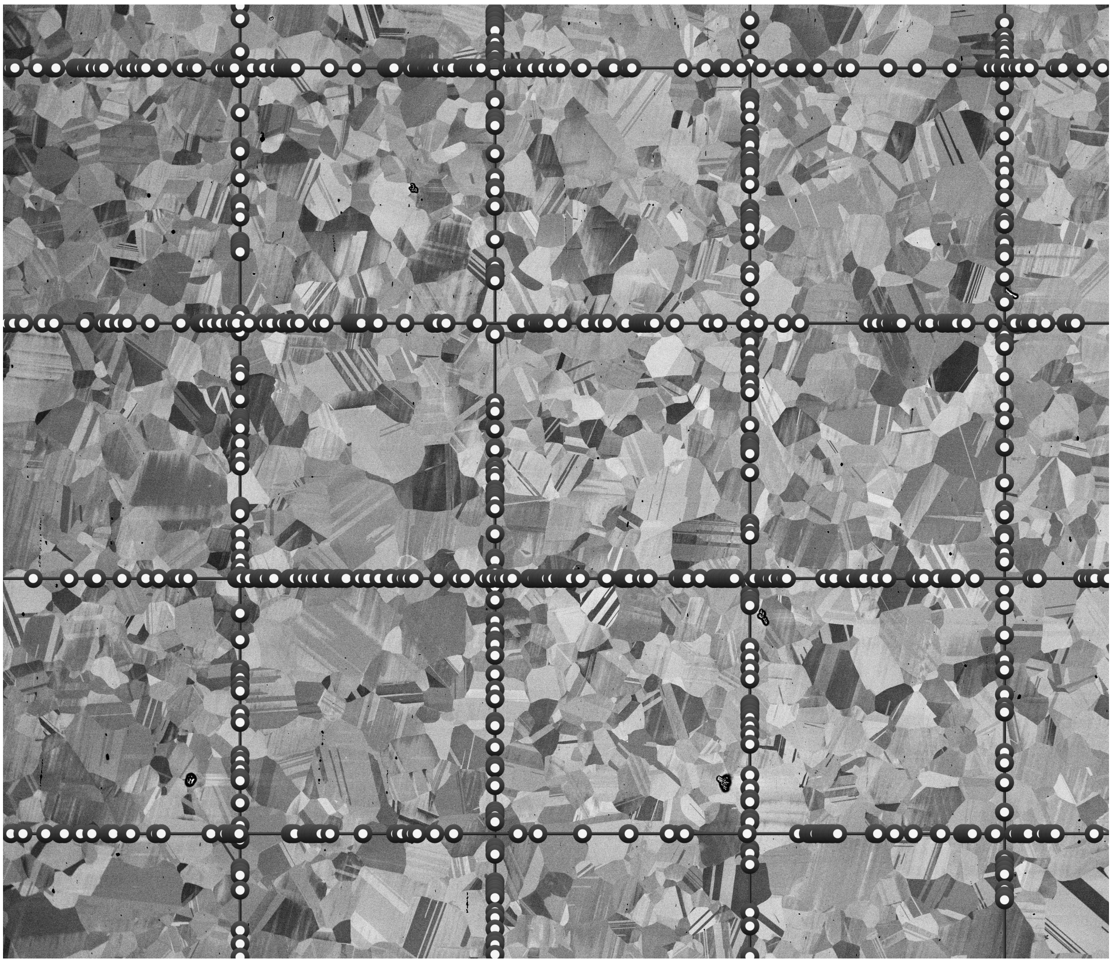

4.1. Statistical Analysis

Statistical Data	Length
Object Count	691
Minimum	0.4 µm
Maximum	104.9 µm
Average	18.3 µm
Standard deviation	18.2 µm
Skewness	0.0
Standard deviation (n-1)	18.2 µm
Variance	329.8 µm²
Variance (n-1)	330.2 µm²
Sum	12'646.8 µm
Sum of squares	459'324.8 µm²
Sum of cubes	24'099'752.7 µm³

4.1.1. Chord Lenght Distribution

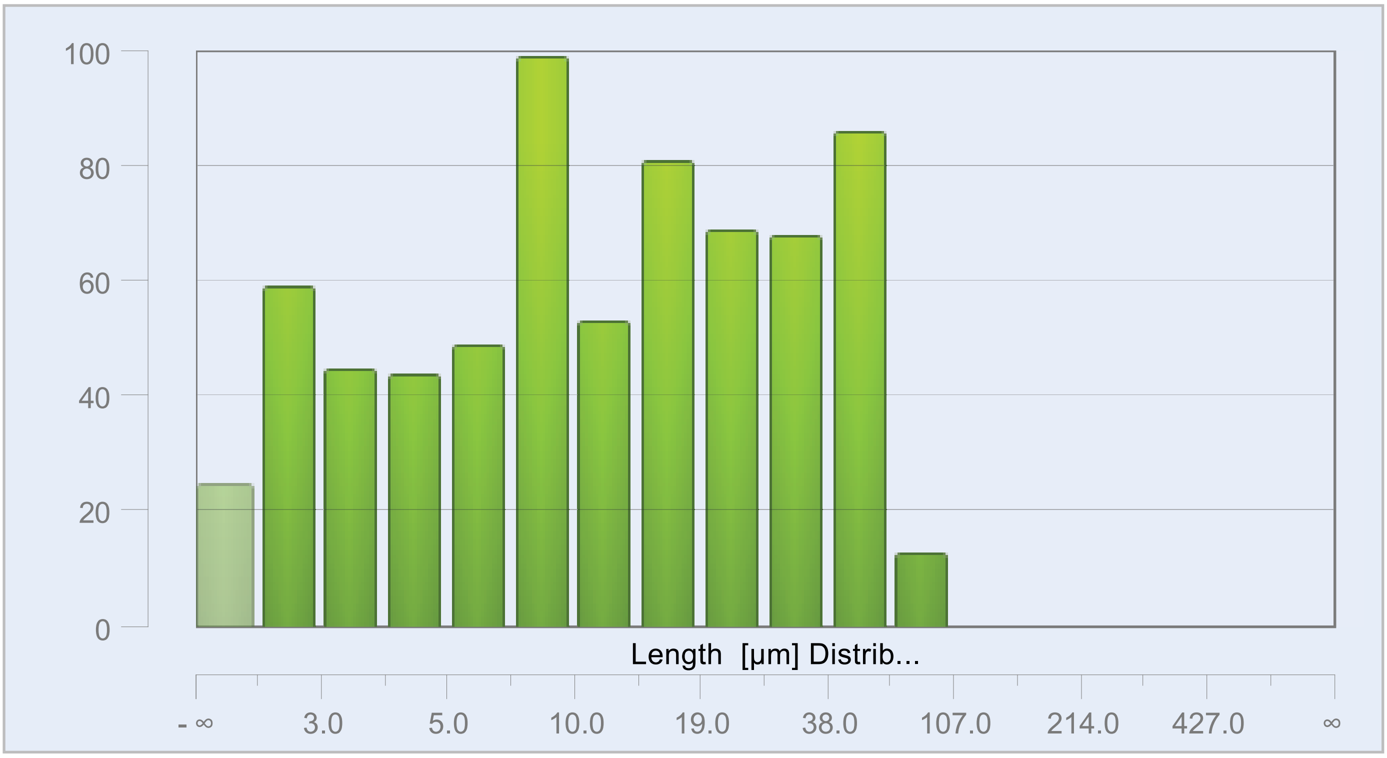

Start	End	Absolute Frequency	Absolute Frequency (accumulated)	Relative Frequency [%]	Relative Frequency (accumulated) [%]
	2.0 µm	25	25	4	4
2.0 µm	3.0 µm	59	84	9	12
3.0 µm	4.0 µm	45	129	7	19
4.0 µm	5.0 µm	44	173	6	25
5.0 µm	7.0 µm	49	222	7	32
7.0 µm	10.0 µm	99	321	14	46
10.0 µm	13.0 µm	53	374	8	54
13.0 µm	19.0 µm	81	455	12	66
19.0 µm	27.0 µm	69	524	10	76
27.0 µm	38.0 µm	68	592	10	86
38.0 µm	75.0 µm	86	678	12	98
75.0 µm	107.0 µm	13	691	2	100
107.0 µm	151.0 µm	0	691	0	100
151.0 µm	214.0 µm	0	691	0	100
214.0 µm	302.0 µm	0	691	0	100
302.0 µm	427.0 µm	0	691	0	100
427.0 µm	600.0 µm	0	691	0	100
600.0 µm		0	691	0	100

5. Single Result 4 (CrCoNi - ASTM E 112_CrCoNi_homogenized_8.1mmSW_1000°C_180min_00083)

Mean chord length	18.3 µm
Grain size (ASTM)	8.3
Grain size (G643)	8.2
Grain stretching	88.6 %

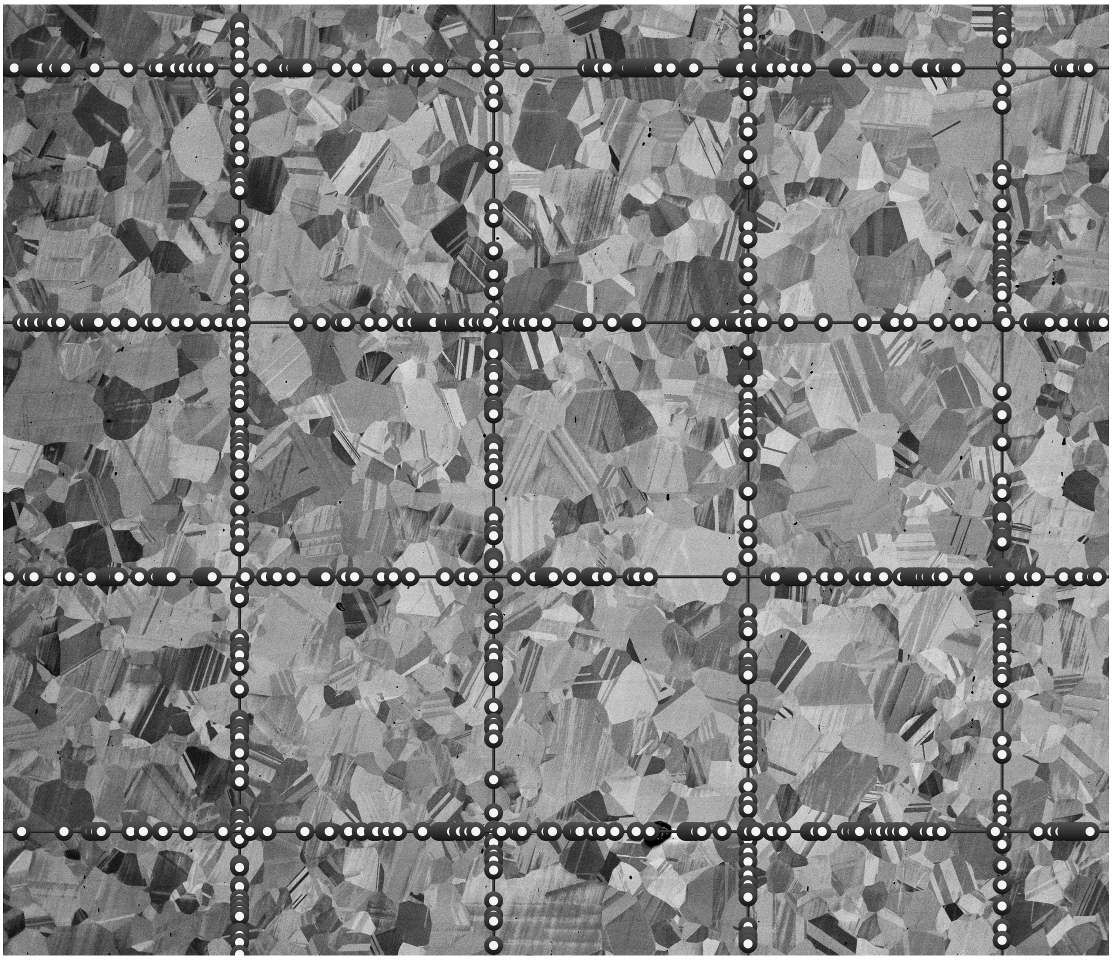

5.1. Statistical Analysis

Statistical Data	Length
Object Count	689
Minimum	0.8 μm
Maximum	127.2 μm
Average	18.3 μm
Standard deviation	17.4 μm
Skewness	0.0
Standard deviation (n-1)	17.4 μm
Variance	303.8 μm²
Variance (n-1)	304.2 μm²
Sum	12'621.4 μm
Sum of squares	440'497.3 μm²
Sum of cubes	22'869'249.8 μm³

5.1.1. Chord Lenght Distribution

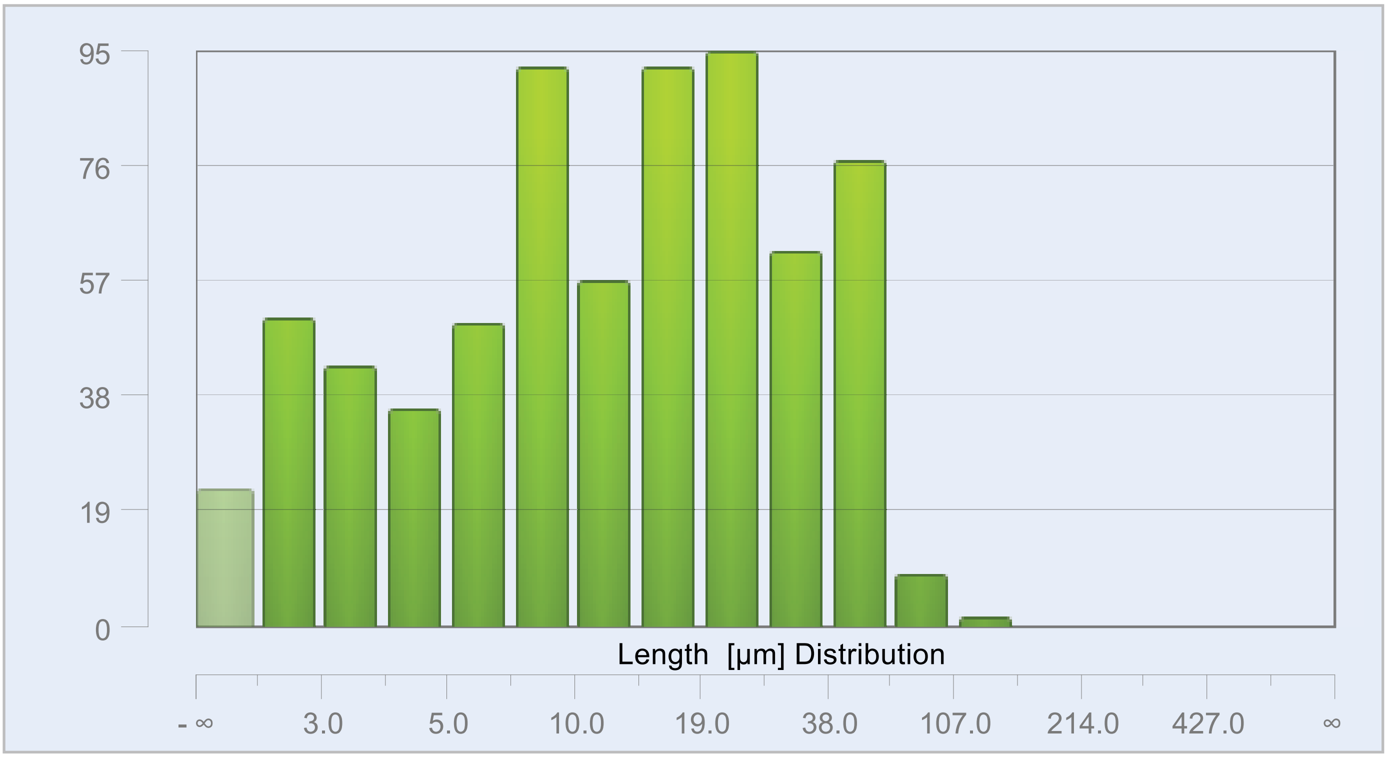

Start	End	Absolute Frequency	Absolute Frequency (accumulated)	Relative Frequency [%]	Relative Frequency (accumulated) [%]
	2.0 µm	23	23	3	3
2.0 µm	3.0 µm	51	74	7	11
3.0 µm	4.0 µm	43	117	6	17
4.0 µm	5.0 µm	36	153	5	22
5.0 µm	7.0 µm	50	203	7	29
7.0 µm	10.0 µm	92	295	13	43
10.0 µm	13.0 µm	57	352	8	51
13.0 µm	19.0 µm	92	444	13	64
19.0 µm	27.0 µm	95	539	14	78
27.0 µm	38.0 µm	62	601	9	87
38.0 µm	75.0 µm	77	678	11	98
75.0 µm	107.0 µm	9	687	1	100
107.0 µm	151.0 µm	2	689	0	100
151.0 µm	214.0 µm	0	689	0	100
214.0 µm	302.0 µm	0	689	0	100
302.0 µm	427.0 µm	0	689	0	100
427.0 µm	600.0 µm	0	689	0	100
600.0 µm		0	689	0	100
