# Supplementary material for: Benchmark dataset of the effect of grain size on strength in the single-phase FCC CrCoNi medium entropy alloy
Source: Data Brief. 2019 Oct 1;27:104592. doi: 10.1016/j.dib.2019.104592 (PMC6812030; doi:10.1016/j.dib.2019.104592)
Supplement: Multimedia component 1 [file mmc1.zip › CrCoNi_1273K_180min/CrCoNi_1273K_180min_d=42μm.pdf]

Analysebericht

27.08.2017 21:28:06

powered by imagic.ch

cumulative Result 1

Anzahl Bilder	4
Korngröße (ASTM)	5,9
Korngröße (G643)	5,9
Kornstreckung	99,2 %
Mittlere Sehnenlänge	41,4 µm

Single Result 1 (CrCoNi Twins grain size_ASTM 1000C 180min_00171)

Mittlere Sehnenlänge	41,9 µm
Korngröße (ASTM)	5,9
Korngröße (G643)	5,8
Kornstreckung	82,6 %

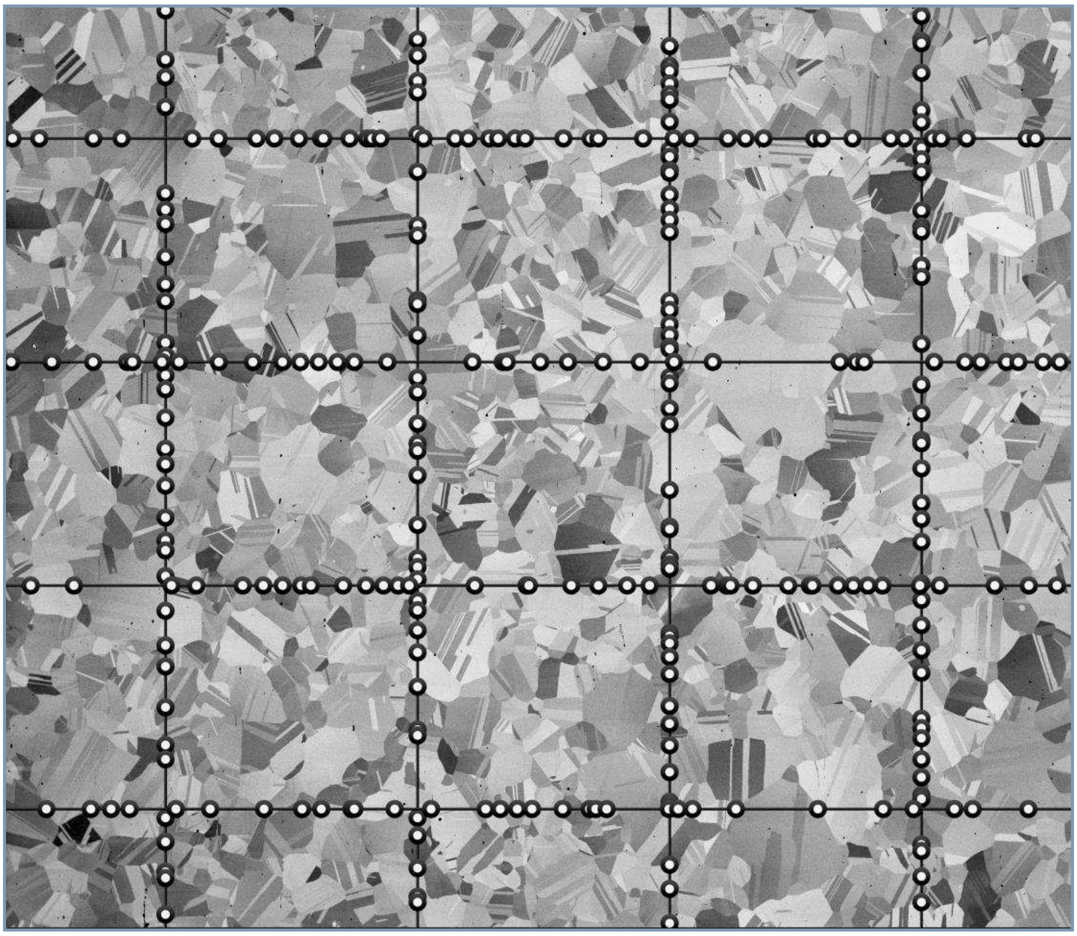

Statistische Analyse

Statistische Daten	Länge
Anzahl Objekte	301
Minimum	3,3 µm
Maximum	201,7 µm
Mittelwert	41,9 µm
Standardabweichung	28,8 µm
Schiefe	0,0
Standardabweichung (n-1)	28,8 µm
Varianz	827,5 µm²
Varianz (n-1)	830,3 µm²
Summe	12'623,1 µm

Quadratsumme	778'466,6 µm²
Kubiksumme	64'295'631,6 µm³

Chord Lenght Distribution

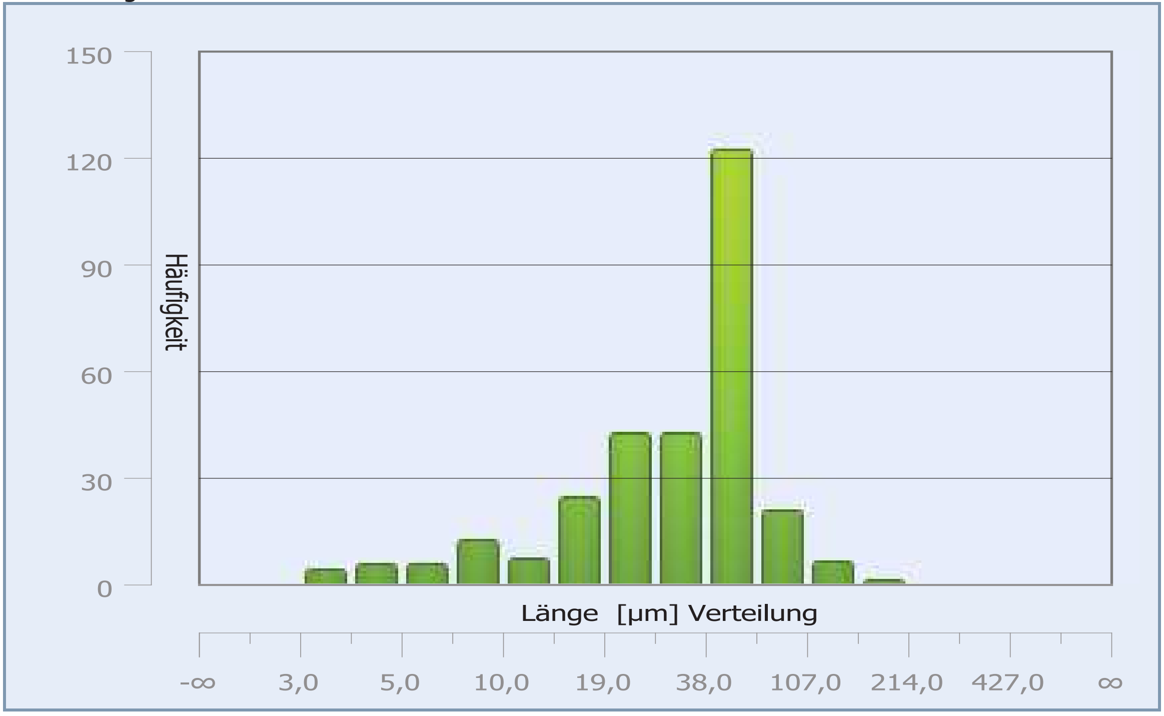

Start	Ende	Absolute Häufigkeit	Absolute Häufigkeit (kumuliert)	Relative Häufigkeit [%]	Relative Häufigkeit (kumuliert) [%]
	2,0 µm	0	0	0	0
2,0 µm	3,0 µm	0	0	0	0
3,0 µm	4,0 µm	5	5	2	2
4,0 µm	5,0 µm	6	11	2	4
5,0 µm	7,0 µm	6	17	2	6
7,0 µm	10,0 µm	13	30	4	10
10,0 µm	13,0 µm	8	38	3	13
13,0 µm	19,0 µm	25	63	8	21
19,0 µm	27,0 µm	43	106	14	35
27,0 µm	38,0 µm	43	149	14	50
38,0 µm	75,0 µm	122	271	41	90
75,0 µm	107,0 µm	21	292	7	97
107,0 µm	151,0 µm	7	299	2	99
151,0	214,0	2	301	1	100

µm	µm				
214,0 µm	302,0 µm	0	301	0	100
302,0 µm	427,0 µm	0	301	0	100
427,0 µm	600,0 µm	0	301	0	100
600,0 µm		0	301	0	100

Single Result 2 (CrCoNi Twins grain size_ASTM 1000C 180min_00172)

Mittlere Sehnenlänge	41,4 µm
Korngröße (ASTM)	5,9
Korngröße (G643)	5,9
Kornstreckung	98,1 %

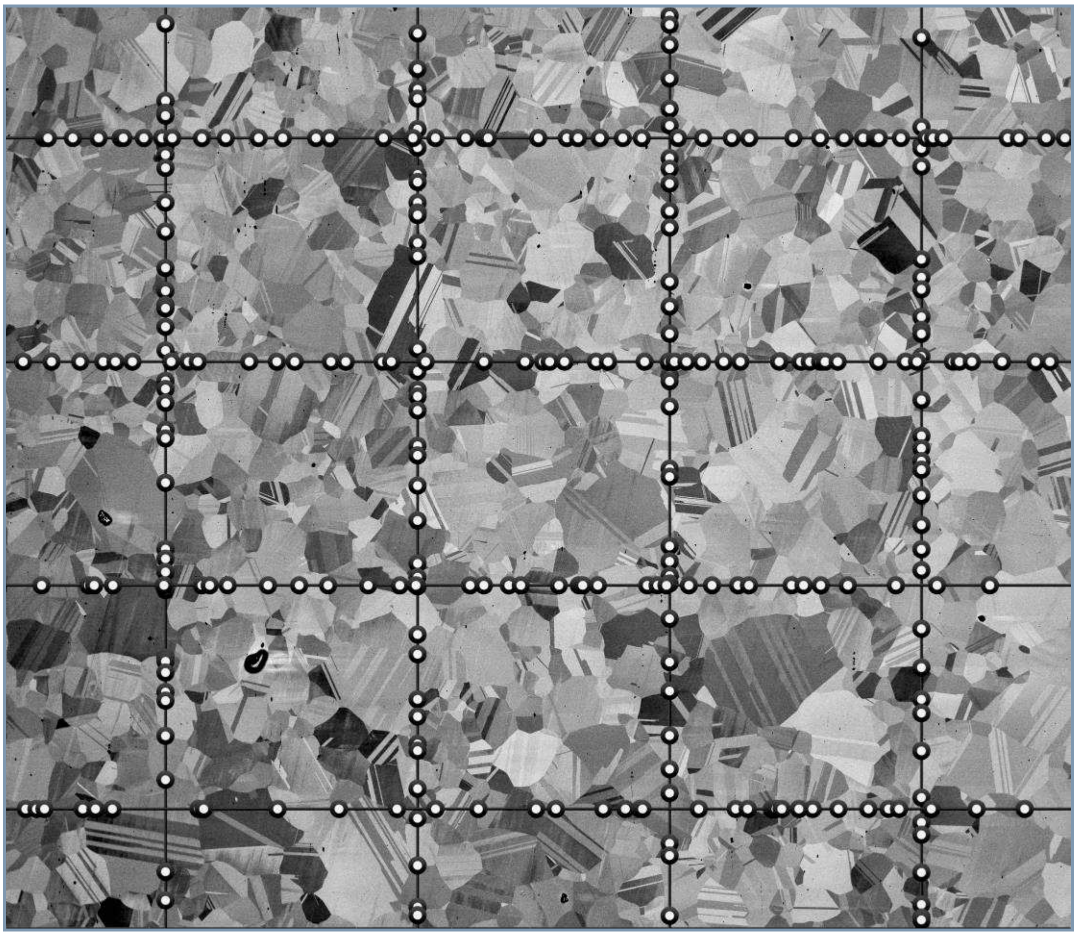

Statistische Analyse

Statistische Daten	Länge
Anzahl Objekte	305
Minimum	3,7 µm
Maximum	147,5 µm
Mittelwert	41,4 µm
Standardabweichung	27,9 µm
Schiefe	0,0
Standardabweichung (n-1)	27,9 µm
Varianz	776,0 µm²

Varianz (n-1)	778,6 µm²
Summe	12'623,1 µm
Quadratsumme	759'122,0 µm²
Kubiksumme	59'595'693,6 µm³

Chord Lenght Distribution

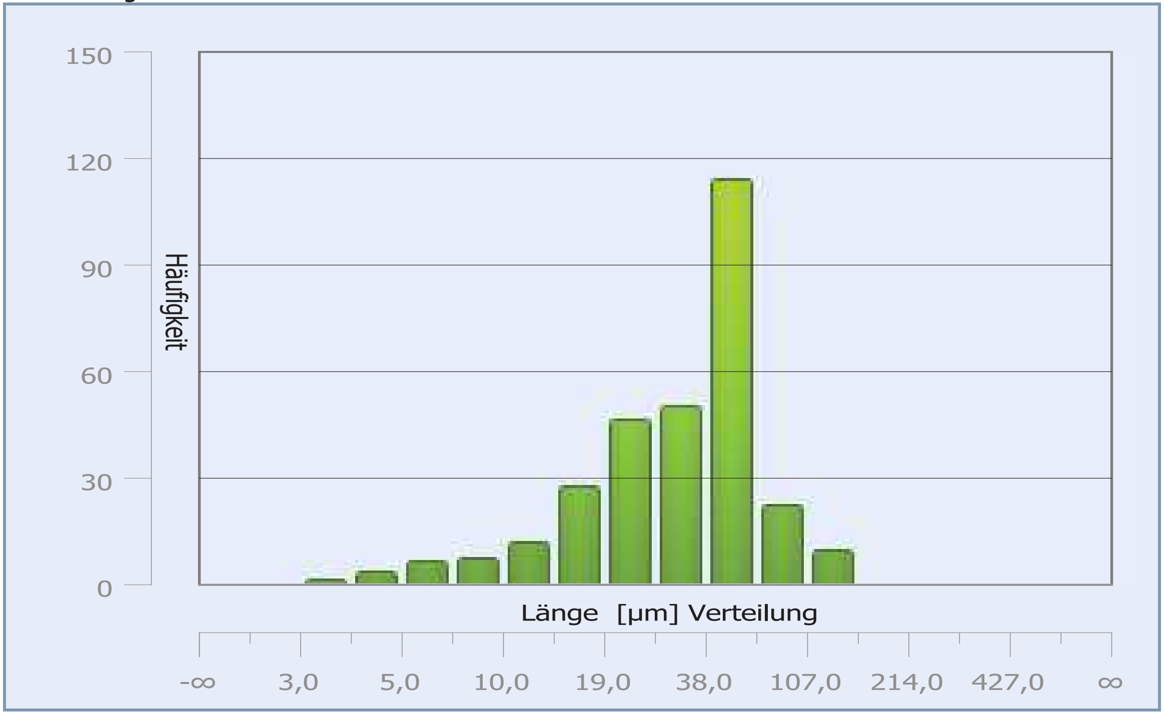

Start	Ende	Absolute Häufigkeit	Absolute Häufigkeit (kumuliert)	Relative Häufigkeit [%]	Relative Häufigkeit (kumuliert) [%]
	2,0 µm	0	0	0	0
2,0 µm	3,0 µm	0	0	0	0
3,0 µm	4,0 µm	2	2	1	1
4,0 µm	5,0 µm	4	6	1	2
5,0 µm	7,0 µm	7	13	2	4
7,0 µm	10,0 µm	8	21	3	7
10,0 µm	13,0 µm	12	33	4	11
13,0 µm	19,0 µm	28	61	9	20
19,0 µm	27,0 µm	47	108	15	35
27,0 µm	38,0 µm	50	158	16	52
38,0 µm	75,0 µm	114	272	37	89
75,0 µm	107,0 µm	23	295	8	97
107,0	151,0	10	305	3	100

µm	µm				
151,0 µm	214,0 µm	0	305	0	100
214,0 µm	302,0 µm	0	305	0	100
302,0 µm	427,0 µm	0	305	0	100
427,0 µm	600,0 µm	0	305	0	100
600,0 µm		0	305	0	100

Single Result 3 (CrCoNi Twins grain size_ASTM 1000C 180min_00173)

Mittlere Sehnenlänge	40,5 µm
Korngröße (ASTM)	6
Korngröße (G643)	5,9
Kornstreckung	91,6 %

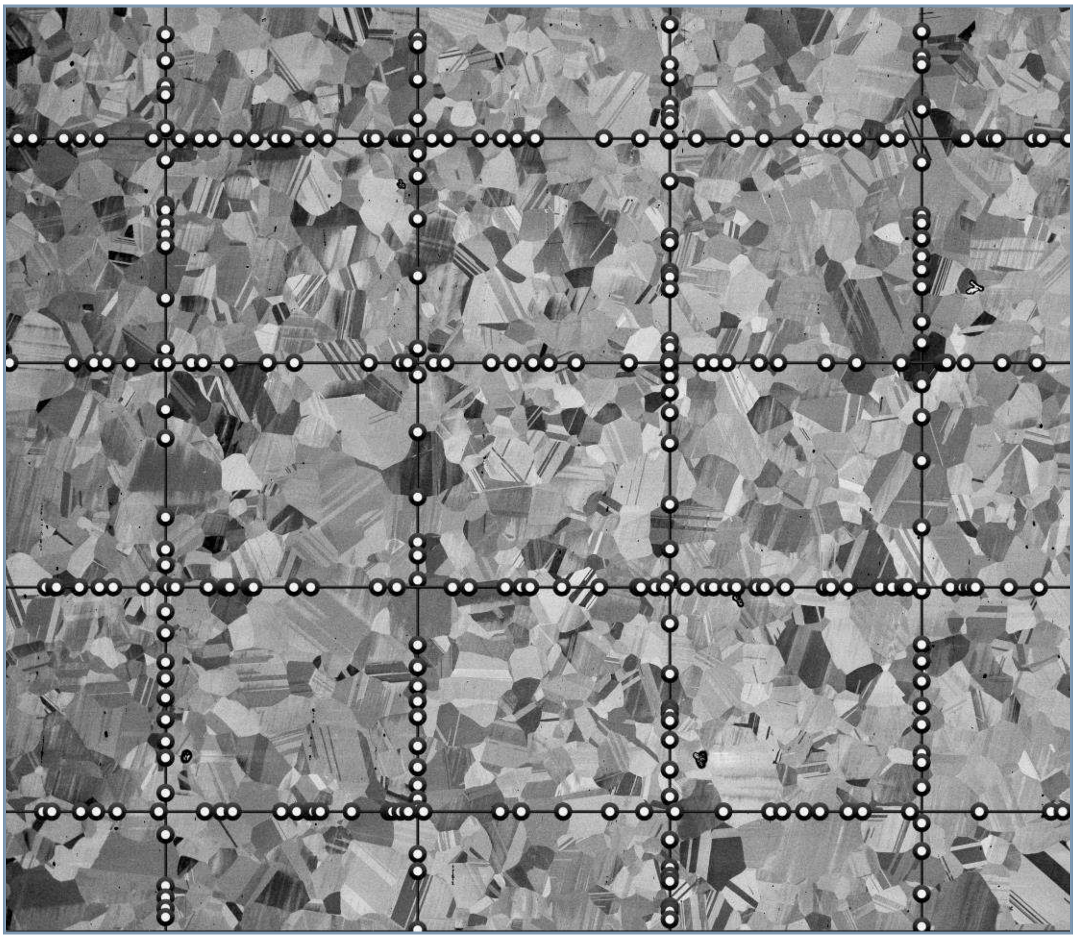

Statistische Analyse

Statistische Daten	Länge
Anzahl Objekte	312
Minimum	2,9 µm
Maximum	124,9 µm
Mittelwert	40,5 µm
Standardabweichung	26,5 µm
Schiefe	0,0

Standardabweichung (n-1)	26,5 µm
Varianz	700,5 µm²
Varianz (n-1)	702,7 µm²
Summe	12'628,6 µm
Quadratsumme	729'707,4 µm²
Kubiksumme	52'369'915,2 µm³

Chord Lenght Distribution

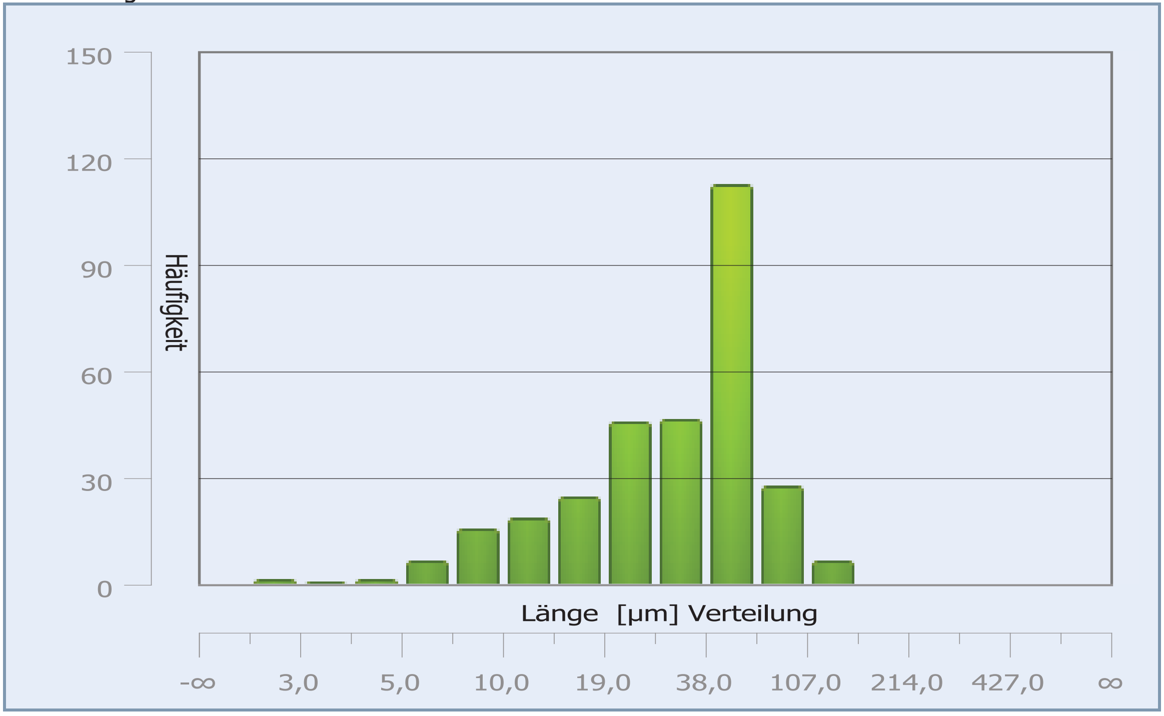

Start	Ende	Absolute Häufigkeit	Absolute Häufigkeit (kumuliert)	Relative Häufigkeit [%]	Relative Häufigkeit (kumuliert) [%]
	2,0 µm	0	0	0	0
2,0 µm	3,0 µm	2	2	1	1
3,0 µm	4,0 µm	1	3	0	1
4,0 µm	5,0 µm	2	5	1	2
5,0 µm	7,0 µm	7	12	2	4
7,0 µm	10,0 µm	16	28	5	9
10,0 µm	13,0 µm	19	47	6	15
13,0 µm	19,0 µm	25	72	8	23
19,0 µm	27,0 µm	46	118	15	38
27,0 µm	38,0 µm	47	165	15	53
38,0 µm	75,0 µm	112	277	36	89
75,0	107,0	28	305	9	98

µm	µm				
107,0 µm	151,0 µm	7	312	2	100
151,0 µm	214,0 µm	0	312	0	100
214,0 µm	302,0 µm	0	312	0	100
302,0 µm	427,0 µm	0	312	0	100
427,0 µm	600,0 µm	0	312	0	100
600,0 µm		0	312	0	100

Single Result 4 (CrCoNi Twins grain size_ASTM 1000C 180min_00174)

Mittlere Sehnenlänge	42 µm
Korngröße (ASTM)	5,9
Korngröße (G643)	5,8
Kornstreckung	95,3 %

Statistische Analyse

**Statistische Daten**	**Länge**
Anzahl Objekte	301
Minimum	2,9 µm
Maximum	167,2 µm
Mittelwert	42,0 µm
Standardabweichung	27,1 µm
Schiefe	0,0
Standardabweichung (n-1)	27,2 µm
Varianz	736,4 µm²
Varianz (n-1)	738,9 µm²
Summe	12'644,0 µm
Quadratsumme	752'794,6 µm²
Kubiksumme	56'492'384,6 µm³

Chord Lenght Distribution

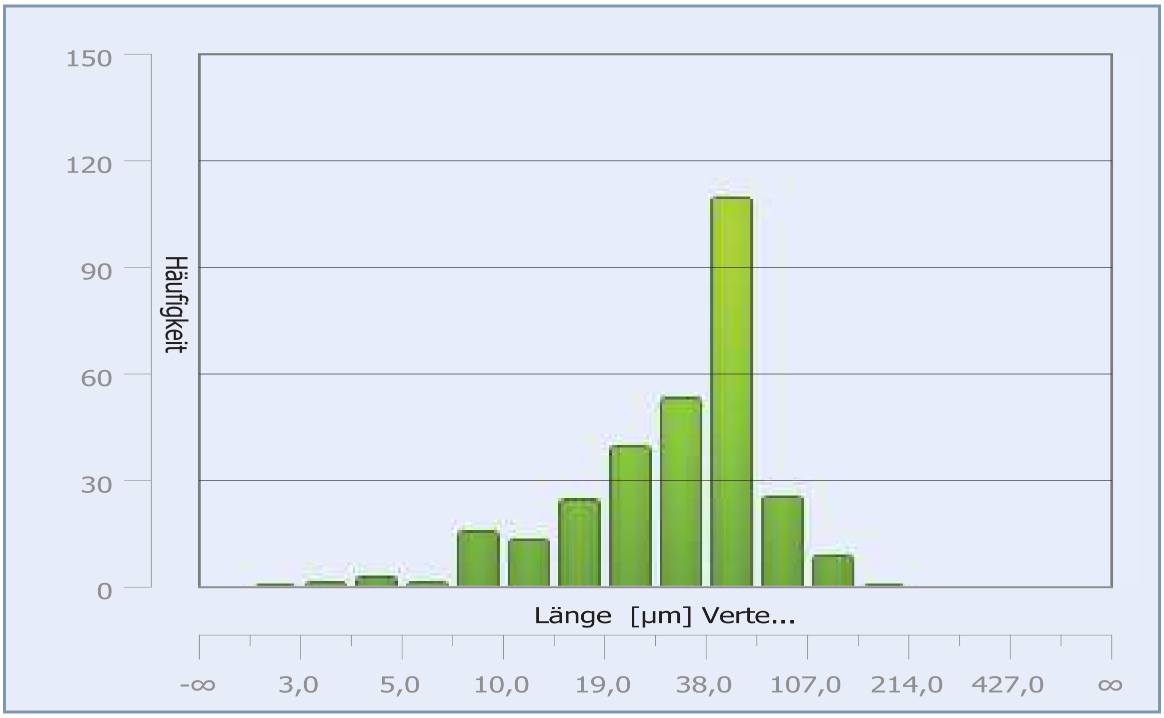

Start	Ende	Absolute Häufigkeit	Absolute Häufigkeit (kumuliert)	Relative Häufigkeit [%]	Relative Häufigkeit (kumuliert) [%]
	2,0 µm	0	0	0	0
2,0 µm	3,0 µm	1	1	0	0
3,0 µm	4,0 µm	2	3	1	1
4,0 µm	5,0 µm	3	6	1	2
5,0 µm	7,0 µm	2	8	1	3
7,0 µm	10,0 µm	16	24	5	8
10,0 µm	13,0 µm	14	38	5	13
13,0 µm	19,0 µm	25	63	8	21
19,0 µm	27,0 µm	40	103	13	34
27,0 µm	38,0 µm	53	156	18	52
38,0 µm	75,0 µm	109	265	36	88
75,0 µm	107,0 µm	26	291	9	97
107,0 µm	151,0 µm	9	300	3	100
151,0 µm	214,0 µm	1	301	0	100
214,0 µm	302,0 µm	0	301	0	100
302,0	427,0	0	301	0	100

µm	µm				
427,0 µm	600,0 µm	0	301	0	100
600,0 µm		0	301	0	100
