# Supplementary material for: Benchmark dataset of the effect of grain size on strength in the single-phase FCC CrCoNi medium entropy alloy
Source: Data Brief. 2019 Oct 1;27:104592. doi: 10.1016/j.dib.2019.104592 (PMC6812030; doi:10.1016/j.dib.2019.104592)
Supplement: Multimedia component 1 [file mmc1.zip › CrCoNi_1273K_60min/CrCoNi_1273K_60min_c=15.8μm.pdf]

Analysebericht

Nov 2, 2017 4:44:11 PM

powered by imagic.ch

1. cumulative Result 1

Number of images	4
Grain size (ASTM)	8.7
Grain size (G643)	8.6
Grain stretching	83.4 %
Mean chord length	15.8 µm

2. Single Result 1 (CrCoNi - ASTM E 112_CrCoNi_homogenized_8.1mmSW_1000°C_60min_00139)

Mean chord length	15.1 µm
Grain size (ASTM)	8.8
Grain size (G643)	8.8
Grain stretching	89.9 %

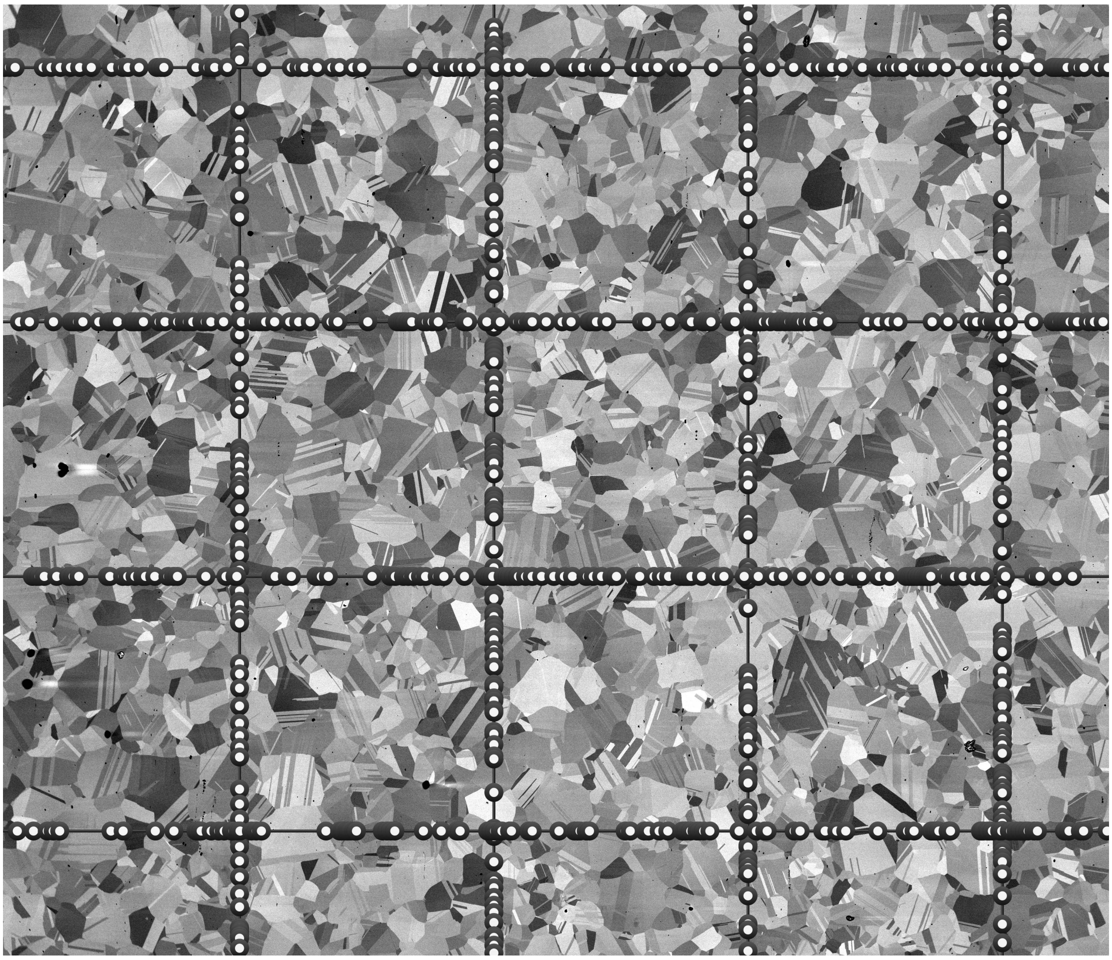

2.1. Statistical Analysis

Statistical Data	Length
Object Count	837
Minimum	0.8 µm
Maximum	98.8 µm
Average	15.1 µm
Standard deviation	14.4 µm
Skewness	0.0
Standard deviation (n-1)	14.4 µm
Variance	208.3 µm²
Variance (n-1)	208.6 µm²
Sum	12'602.2 µm

Statistical Data	Length
Sum of squares	364'127.3 µm²
Sum of cubes	15'631'757.6 µm³

2.1.1. Chord Lenght Distribution

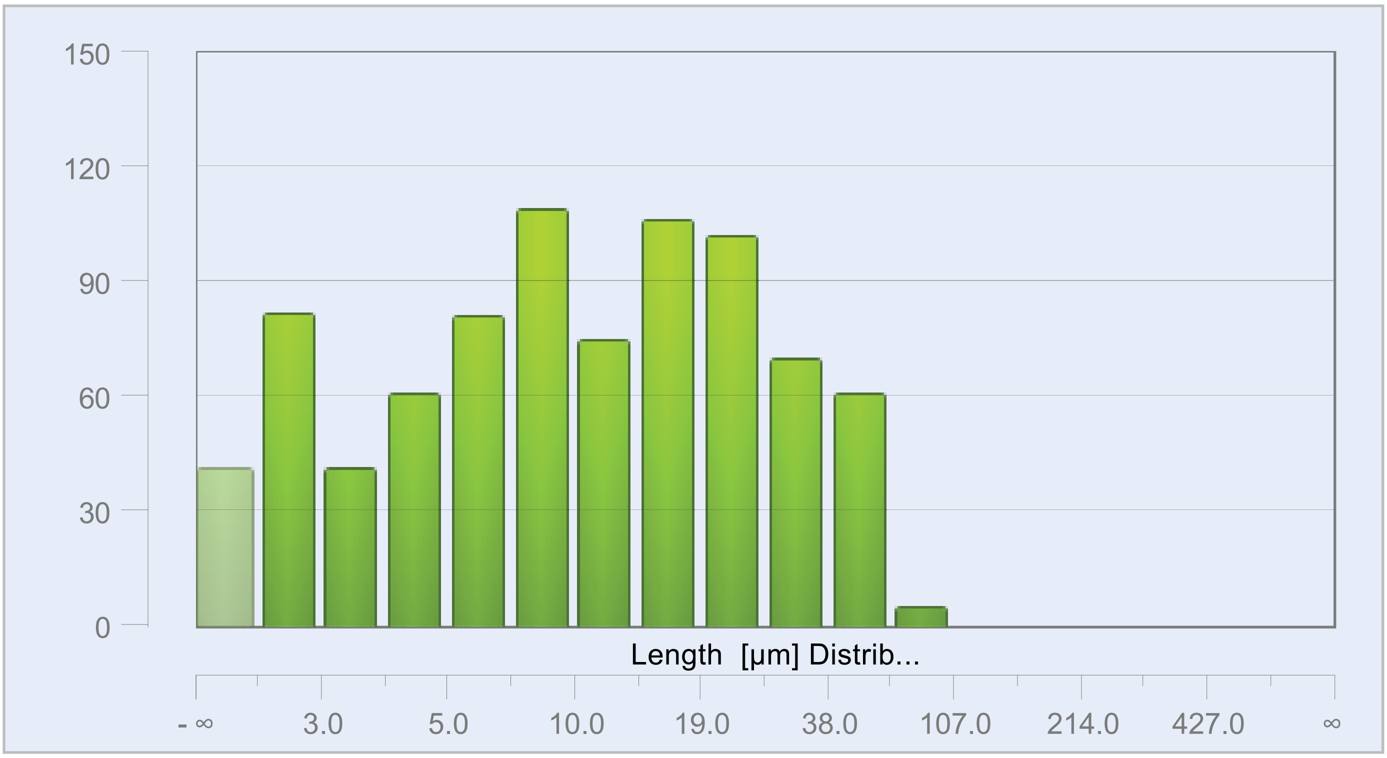

Start	End	Absolute Frequency	Absolute Frequency (accumulated)	Relative Frequency [%]	Relative Frequency (accumulated) [%]
	2.0 µm	42	42	5	5
2.0 µm	3.0 µm	82	124	10	15
3.0 µm	4.0 µm	42	166	5	20
4.0 µm	5.0 µm	61	227	7	27
5.0 µm	7.0 µm	81	308	10	37
7.0 µm	10.0 µm	109	417	13	50
10.0 µm	13.0 µm	75	492	9	59
13.0 µm	19.0 µm	106	598	13	71
19.0 µm	27.0 µm	102	700	12	84
27.0 µm	38.0 µm	70	770	8	92
38.0 µm	75.0 µm	61	831	7	99
75.0 µm	107.0 µm	6	837	1	100
107.0 µm	151.0 µm	0	837	0	100
151.0 µm	214.0 µm	0	837	0	100
214.0 µm	302.0 µm	0	837	0	100
302.0 µm	427.0 µm	0	837	0	100
427.0 µm	600.0 µm	0	837	0	100
600.0 µm		0	837	0	100

3. Single Result 2 (CrCoNi - ASTM E 112_CrCoNi_homogenized_8.1mmSW_1000°C_60min_00140)

Mean chord length	15.1 µm
Grain size (ASTM)	8.8
Grain size (G643)	8.8
Grain stretching	80.7 %

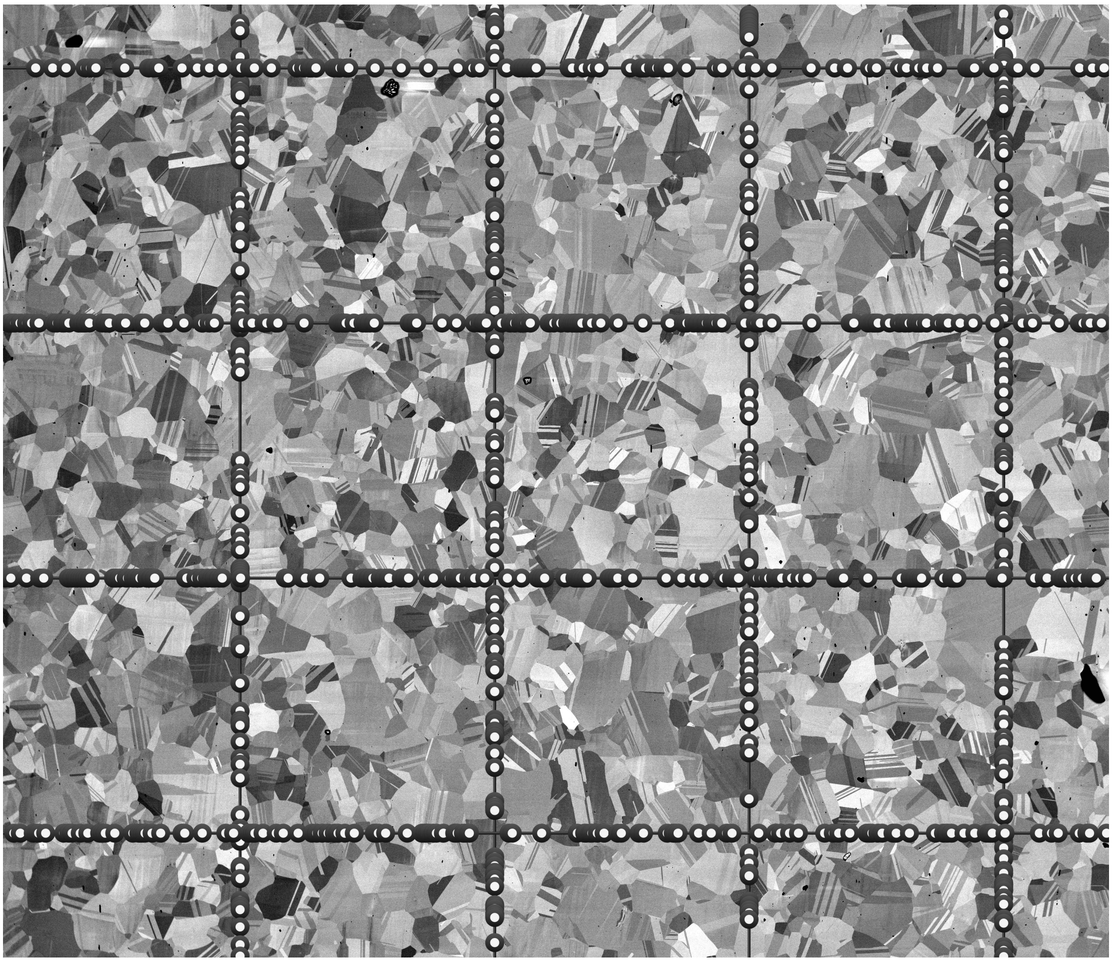

3.1. Statistical Analysis

Statistical Data	Length
Object Count	834
Minimum	0.8 µm
Maximum	135.3 µm
Average	15.1 µm
Standard deviation	15.2 µm
Skewness	0.0
Standard deviation (n-1)	15.2 µm
Variance	231.0 µm²
Variance (n-1)	231.3 µm²
Sum	12'620.9 µm
Sum of squares	383'640.0 µm²
Sum of cubes	17'801'151.2 µm³

3.1.1. Chord Lenght Distribution

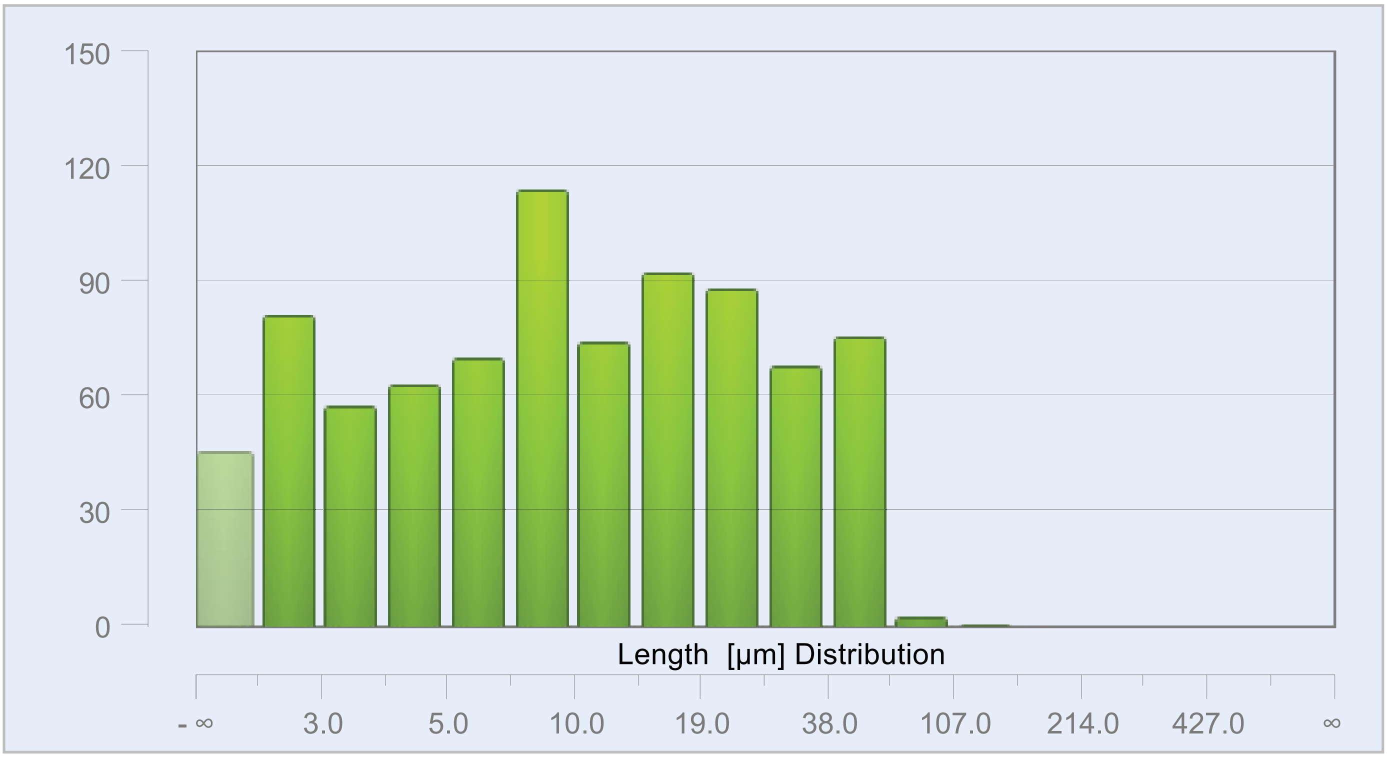

Start	End	Absolute Frequency	Absolute Frequency (accumulated)	Relative Frequency [%]	Relative Frequency (accumulated) [%]
	2.0 µm	46	46	6	6
2.0 µm	3.0 µm	81	127	10	15
3.0 µm	4.0 µm	58	185	7	22
4.0 µm	5.0 µm	63	248	8	30
5.0 µm	7.0 µm	70	318	8	38
7.0 µm	10.0 µm	114	432	14	52
10.0 µm	13.0 µm	74	506	9	61
13.0 µm	19.0 µm	92	598	11	72
19.0 µm	27.0 µm	88	686	11	82
27.0 µm	38.0 µm	68	754	8	90
38.0 µm	75.0 µm	76	830	9	100
75.0 µm	107.0 µm	3	833	0	100
107.0 µm	151.0 µm	1	834	0	100
151.0 µm	214.0 µm	0	834	0	100
214.0 µm	302.0 µm	0	834	0	100
302.0 µm	427.0 µm	0	834	0	100
427.0 µm	600.0 µm	0	834	0	100
600.0 µm		0	834	0	100

4. Single Result 3 (CrCoNi - ASTM E 112_CrCoNi_homogenized_8.1mmSW_1000°C_60min_00141)

Mean chord length	16.8 µm
Grain size (ASTM)	8.5
Grain size (G643)	8.5
Grain stretching	80.9 %

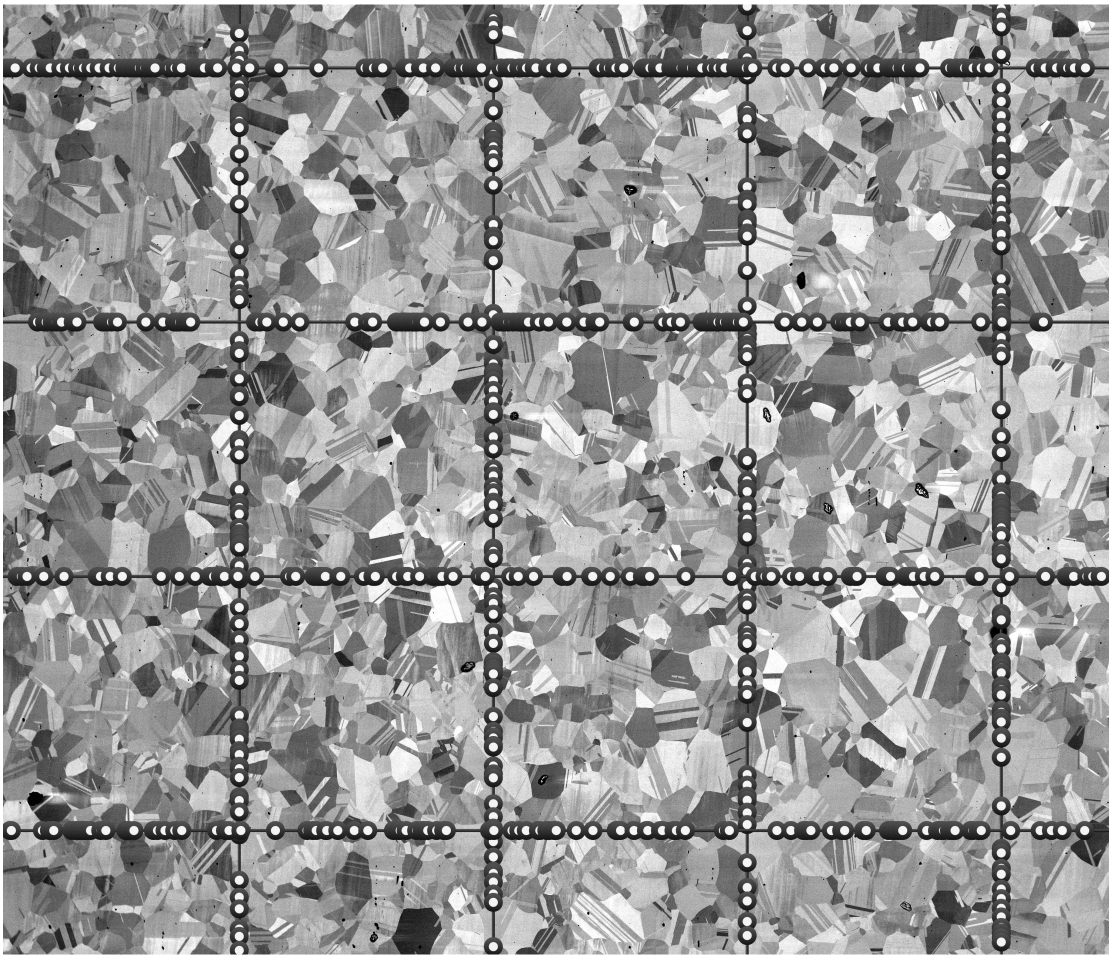

4.1. Statistical Analysis

Statistical Data	Length
Object Count	751
Minimum	0.8 µm
Maximum	100.2 µm
Average	16.8 µm
Standard deviation	16.6 µm
Skewness	0.0
Standard deviation (n-1)	16.6 µm
Variance	275.8 µm²
Variance (n-1)	276.2 µm²
Sum	12'606.0 µm
Sum of squares	418'755.6 µm²
Sum of cubes	20'438'196.8 µm³

4.1.1. Chord Lenght Distribution

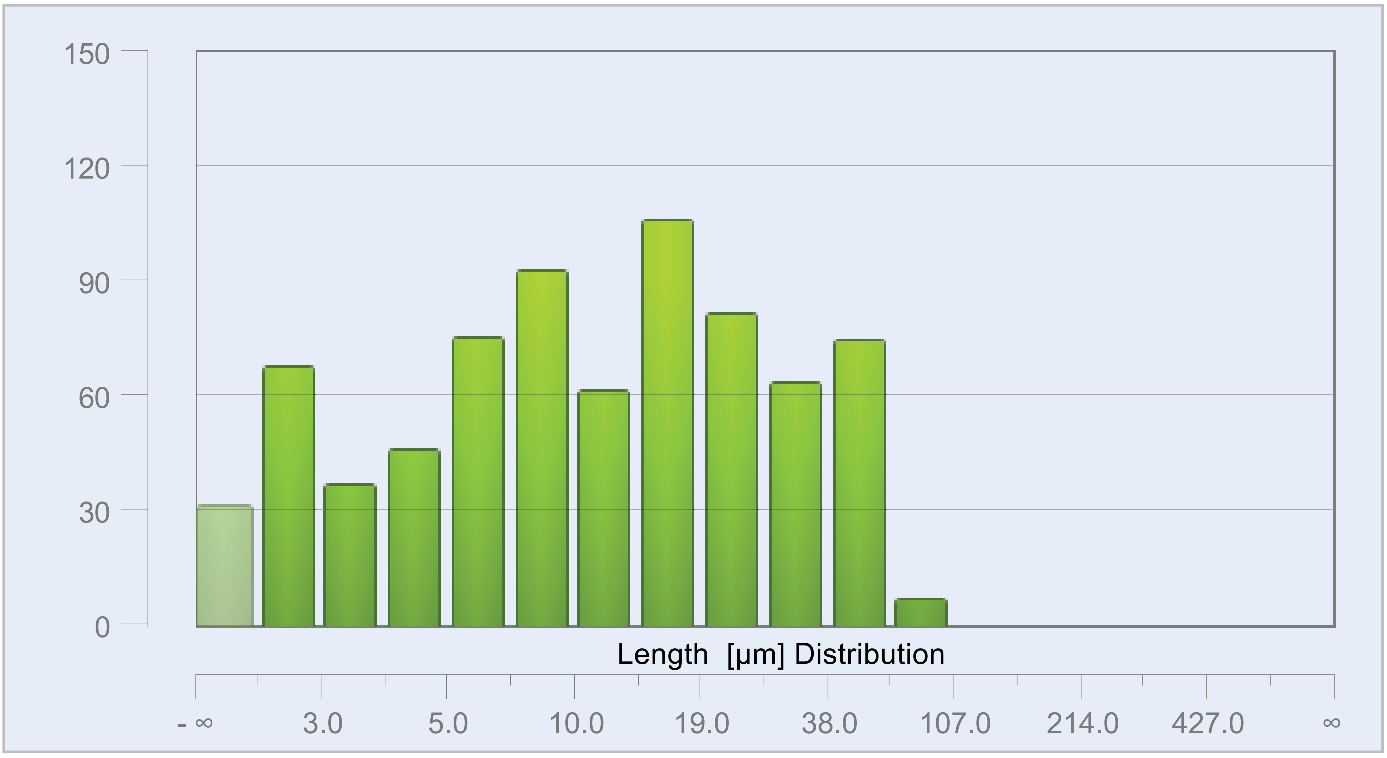

Start	End	Absolute Frequency	Absolute Frequency (accumulated)	Relative Frequency [%]	Relative Frequency (accumulated) [%]
	2.0 µm	32	32	4	4
2.0 µm	3.0 µm	68	100	9	13
3.0 µm	4.0 µm	38	138	5	18
4.0 µm	5.0 µm	47	185	6	25
5.0 µm	7.0 µm	76	261	10	35
7.0 µm	10.0 µm	93	354	12	47
10.0 µm	13.0 µm	62	416	8	55
13.0 µm	19.0 µm	106	522	14	70
19.0 µm	27.0 µm	82	604	11	80
27.0 µm	38.0 µm	64	668	9	89
38.0 µm	75.0 µm	75	743	10	99
75.0 µm	107.0 µm	8	751	1	100
107.0 µm	151.0 µm	0	751	0	100
151.0 µm	214.0 µm	0	751	0	100
214.0 µm	302.0 µm	0	751	0	100
302.0 µm	427.0 µm	0	751	0	100
427.0 µm	600.0 µm	0	751	0	100
600.0 µm		0	751	0	100

5. Single Result 4 (CrCoNi - ASTM E 112_CrCoNi_homogenized_8.1mmSW_1000°C_60min_00138)

Mean chord length	16.2 µm
Grain size (ASTM)	8.6
Grain size (G643)	8.6
Grain stretching	81.9 %

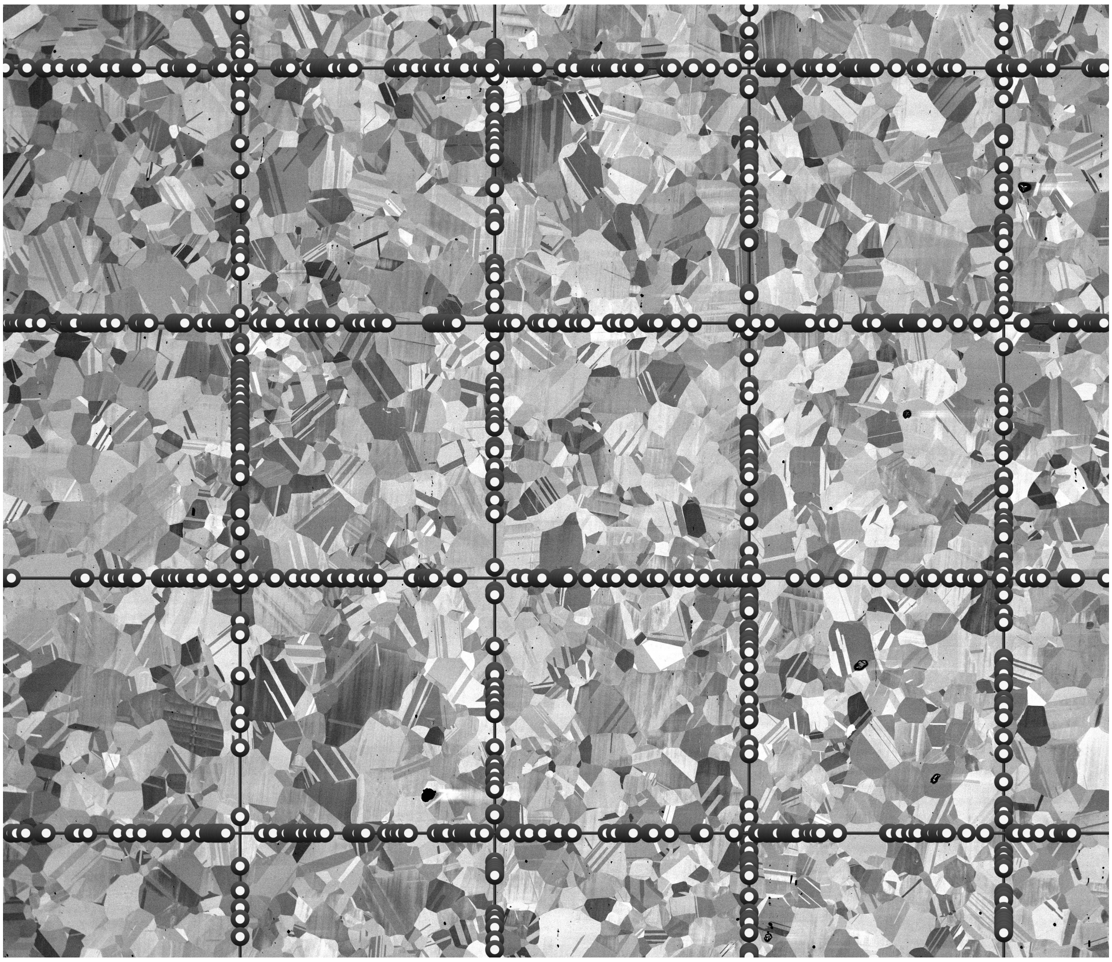

5.1. Statistical Analysis

Statistical Data	Length
Object Count	781
Minimum	0.8 µm
Maximum	105.5 µm
Average	16.2 µm
Standard deviation	15.8 µm
Skewness	0.0
Standard deviation (n-1)	15.9 µm
Variance	251.2 µm²
Variance (n-1)	251.5 µm²
Sum	12'620.9 µm
Sum of squares	400'139.7 µm²
Sum of cubes	18'865'429.5 µm³

5.1.1. Chord Lenght Distribution

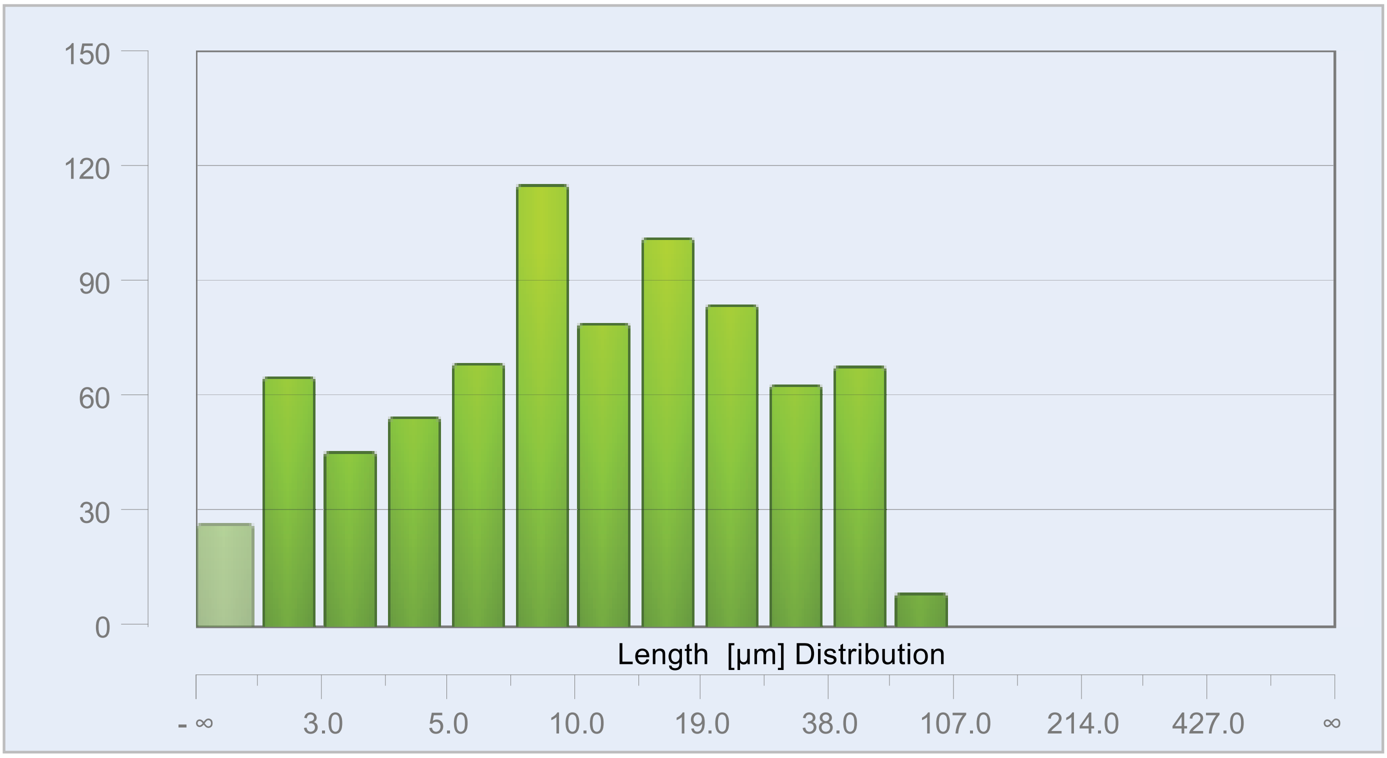

Start	End	Absolute Frequency	Absolute Frequency (accumulated)	Relative Frequency [%]	Relative Frequency (accumulated) [%]
	2.0 µm	27	27	3	3
2.0 µm	3.0 µm	65	92	8	12
3.0 µm	4.0 µm	46	138	6	18
4.0 µm	5.0 µm	55	193	7	25
5.0 µm	7.0 µm	69	262	9	34
7.0 µm	10.0 µm	115	377	15	48
10.0 µm	13.0 µm	79	456	10	58
13.0 µm	19.0 µm	101	557	13	71
19.0 µm	27.0 µm	84	641	11	82
27.0 µm	38.0 µm	63	704	8	90
38.0 µm	75.0 µm	68	772	9	99
75.0 µm	107.0 µm	9	781	1	100
107.0 µm	151.0 µm	0	781	0	100
151.0 µm	214.0 µm	0	781	0	100
214.0 µm	302.0 µm	0	781	0	100
302.0 µm	427.0 µm	0	781	0	100
427.0 µm	600.0 µm	0	781	0	100
600.0 µm		0	781	0	100
