# Supplementary material for: Benchmark dataset of the effect of grain size on strength in the single-phase FCC CrCoNi medium entropy alloy
Source: Data Brief. 2019 Oct 1;27:104592. doi: 10.1016/j.dib.2019.104592 (PMC6812030; doi:10.1016/j.dib.2019.104592)
Supplement: Multimedia component 1 [file mmc1.zip › CrCoNi_1273K_60min/CrCoNi_1273K_60min_d=33μm.pdf]

Analysebericht

Aug 29, 2017 11:07:58 AM

powered by imagic.ch

1. cumulative Result 1

Number of images	4
Grain size (ASTM)	6.5
Grain size (G643)	6.5
Grain stretching	98.1 %
Mean chord length	33.2 µm

2. Single Result 1 (CrCoNi Twins grain size_ASTM 1000C 60min_00187)

Mean chord length	34.1 µm
Grain size (ASTM)	6.5
Grain size (G643)	6.4
Grain stretching	92.1 %

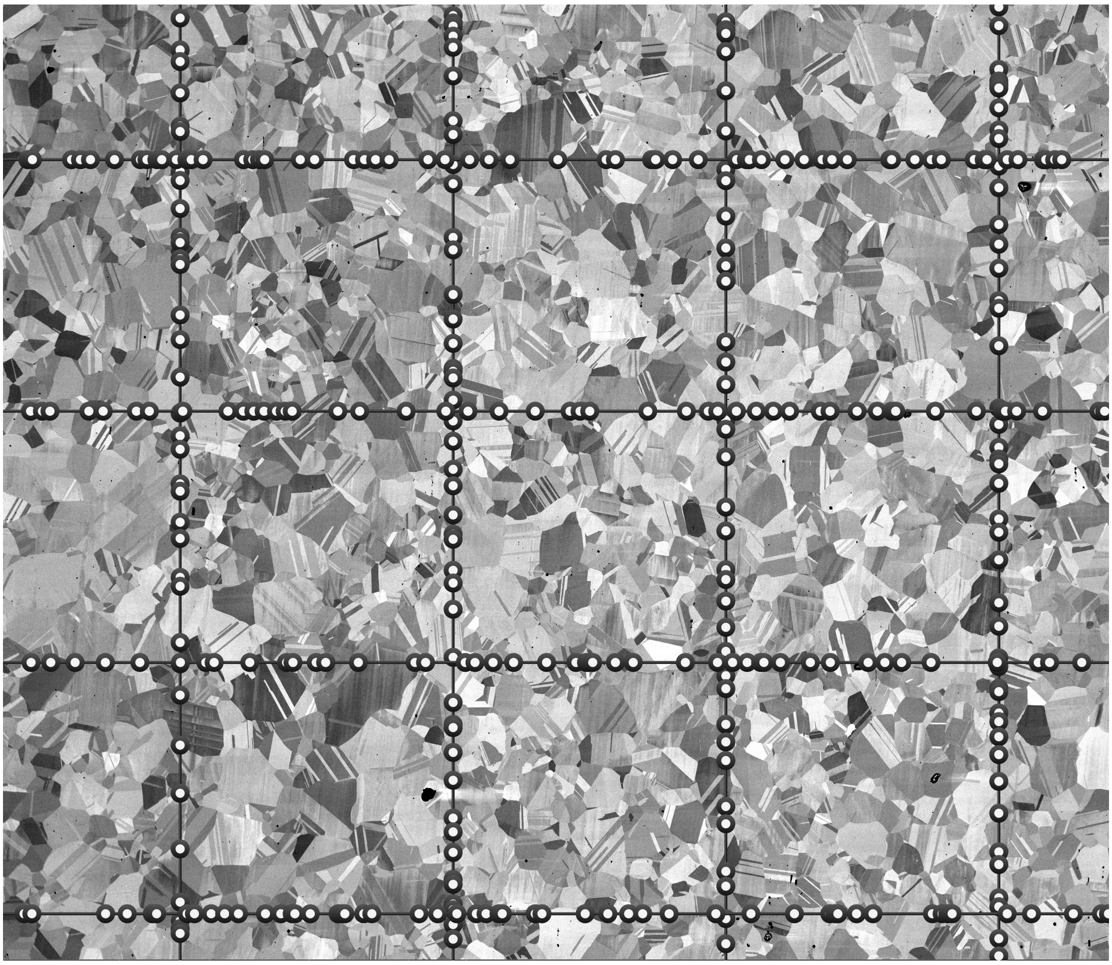

2.1. Statistical Analysis

Statistical Data	Length
Object Count	370
Minimum	0.4 µm
Maximum	112.8 µm
Average	34.1 µm
Standard deviation	21.7 µm
Skewness	0.0
Standard deviation (n-1)	21.7 µm
Variance	471.3 µm²
Variance (n-1)	472.5 µm²
Sum	12'618.2 µm

Statistical Data	Length
Sum of squares	604'683.4 μm²
Sum of cubes	35'323'435.8 μm³

2.1.1. Chord Lenght Distribution

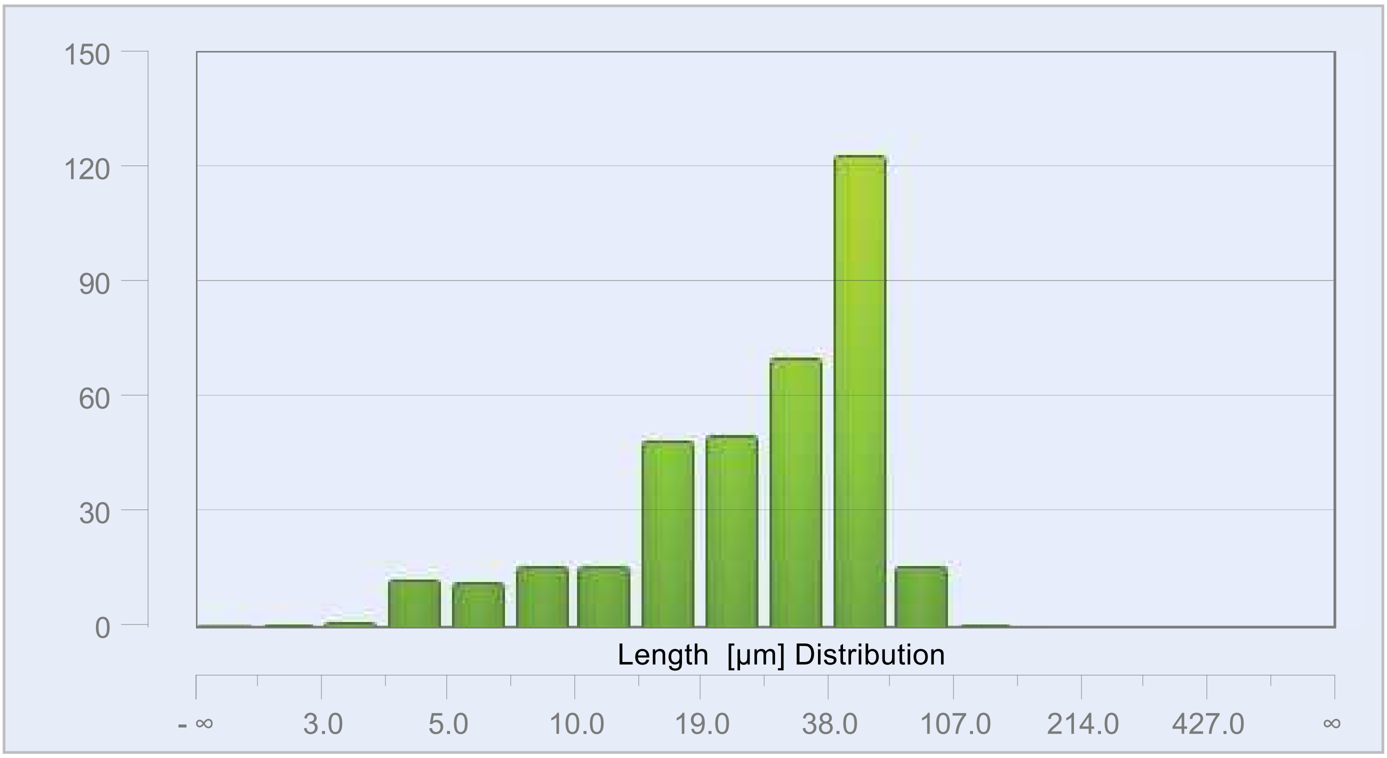

Start	End	Absolute Frequency	Absolute Frequency (accumulated)	Relative Frequency [%]	Relative Frequency (accumulated) [%]
	2.0 μm	1	1	0	0
2.0 μm	3.0 μm	1	2	0	1
3.0 μm	4.0 μm	2	4	1	1
4.0 μm	5.0 μm	13	17	4	5
5.0 μm	7.0 μm	12	29	3	8
7.0 μm	10.0 μm	16	45	4	12
10.0 μm	13.0 μm	16	61	4	16
13.0 μm	19.0 μm	49	110	13	30
19.0 μm	27.0 μm	50	160	14	43
27.0 μm	38.0 μm	70	230	19	62
38.0 μm	75.0 μm	123	353	33	95
75.0 μm	107.0 μm	16	369	4	100
107.0 μm	151.0 μm	1	370	0	100
151.0 μm	214.0 μm	0	370	0	100
214.0 μm	302.0 μm	0	370	0	100
302.0 μm	427.0 μm	0	370	0	100
427.0 μm	600.0 μm	0	370	0	100
600.0 μm		0	370	0	100

3. Single Result 2 (CrCoNi Twins grain size_ASTM 1000C 60min_00188)

Mean chord length	30.8 μm
Grain size (ASTM)	6.8
Grain size (G643)	6.7
Grain stretching	96.2 %

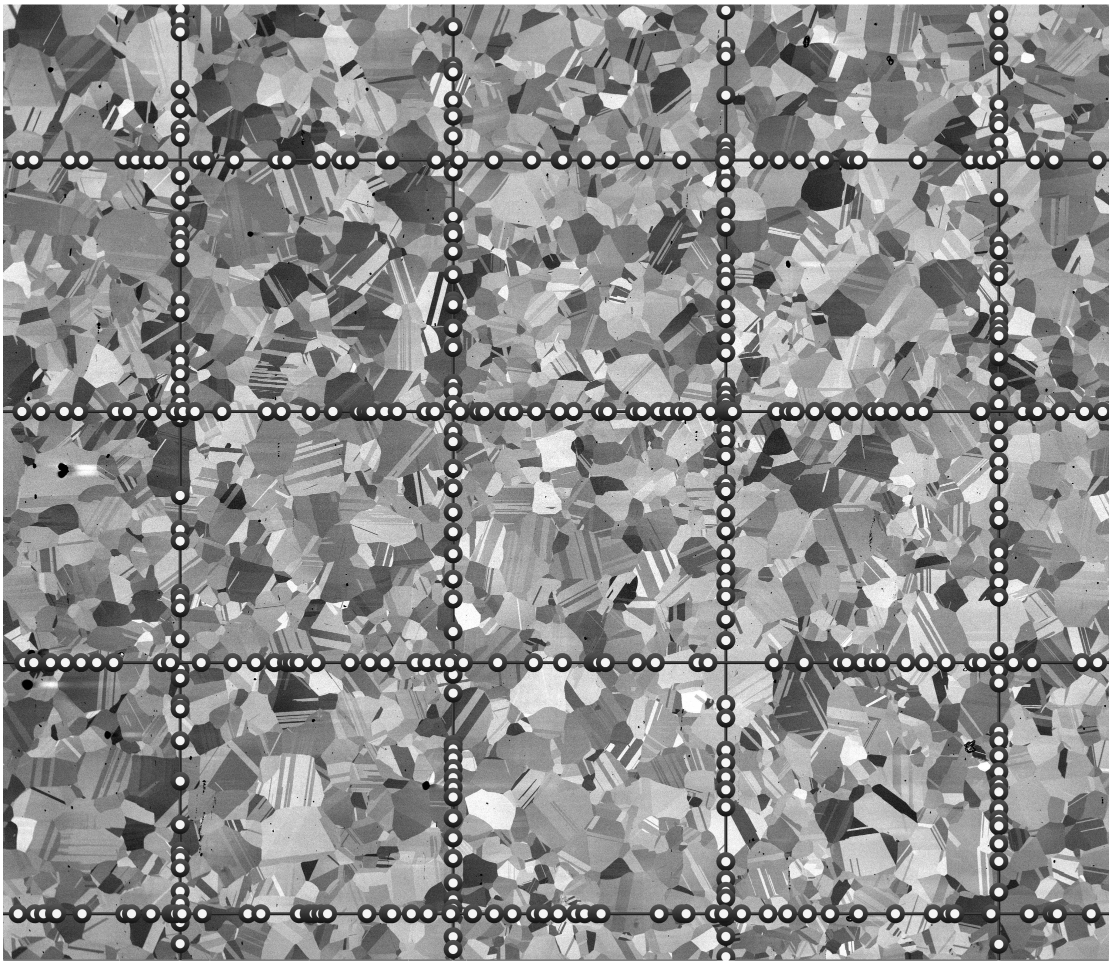

3.1. Statistical Analysis

Statistical Data	Length
Object Count	410
Minimum	2.9 µm
Maximum	118.5 µm
Average	30.8 µm
Standard deviation	19.5 µm
Skewness	0.0
Standard deviation (n-1)	19.5 µm
Variance	378.8 µm²
Variance (n-1)	379.7 µm²
Sum	12'607.8 µm
Sum of squares	542'998.7 µm²
Sum of cubes	29'852'140.5 µm³

3.1.1. Chord Lenght Distribution

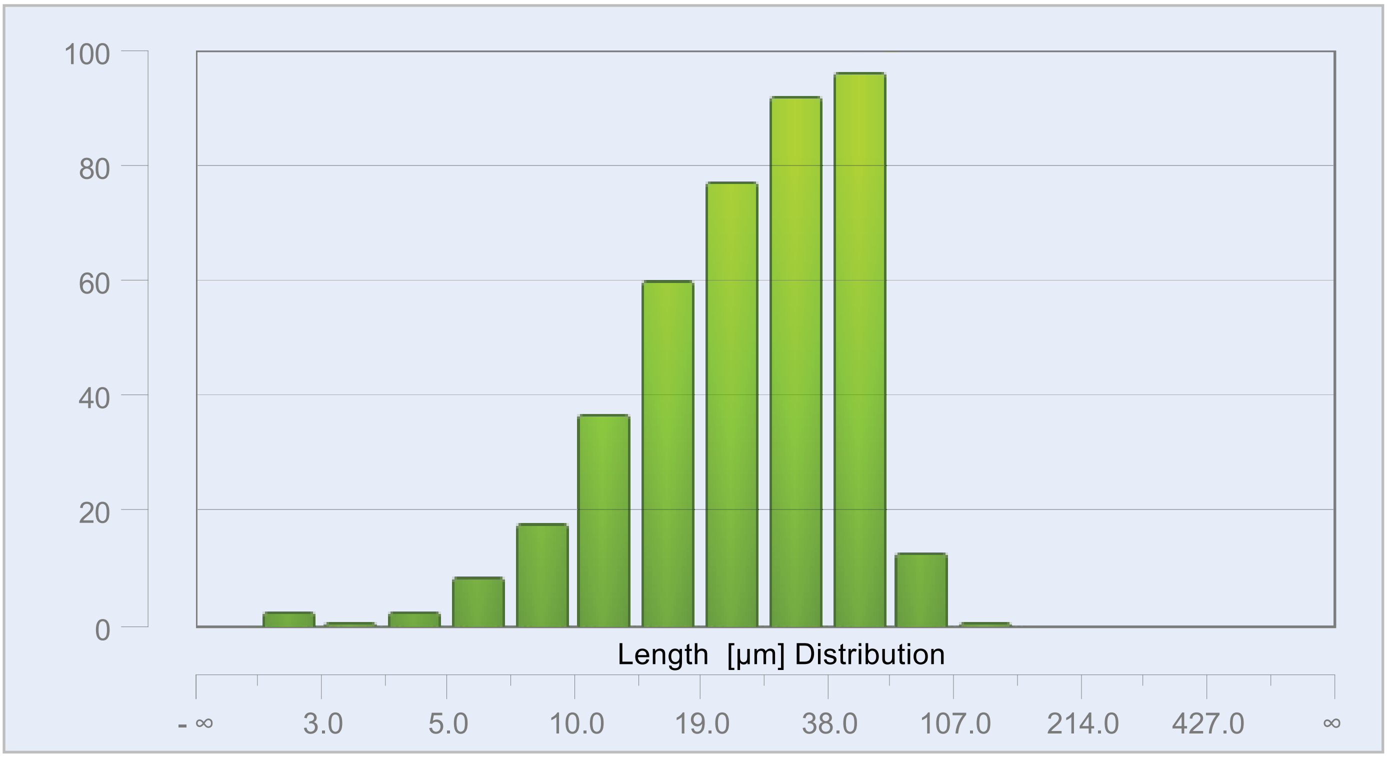

Start	End	Absolute Frequency	Absolute Frequency (accumulated)	Relative Frequency [%]	Relative Frequency (accumulated) [%]
	2.0 µm	0	0	0	0
2.0 µm	3.0 µm	3	3	1	1
3.0 µm	4.0 µm	1	4	0	1
4.0 µm	5.0 µm	3	7	1	2
5.0 µm	7.0 µm	9	16	2	4
7.0 µm	10.0 µm	18	34	4	8
10.0 µm	13.0 µm	37	71	9	17
13.0 µm	19.0 µm	60	131	15	32
19.0 µm	27.0 µm	77	208	19	51
27.0 µm	38.0 µm	92	300	22	73
38.0 µm	75.0 µm	96	396	23	97
75.0 µm	107.0 µm	13	409	3	100
107.0 µm	151.0 µm	1	410	0	100
151.0 µm	214.0 µm	0	410	0	100
214.0 µm	302.0 µm	0	410	0	100
302.0 µm	427.0 µm	0	410	0	100
427.0 µm	600.0 µm	0	410	0	100
600.0 µm		0	410	0	100

4. Single Result 3 (CrCoNi Twins grain size_ASTM 1000C 60min_00189)

Mean chord length	34.1 µm
Grain size (ASTM)	6.5
Grain size (G643)	6.4
Grain stretching	86.7 %

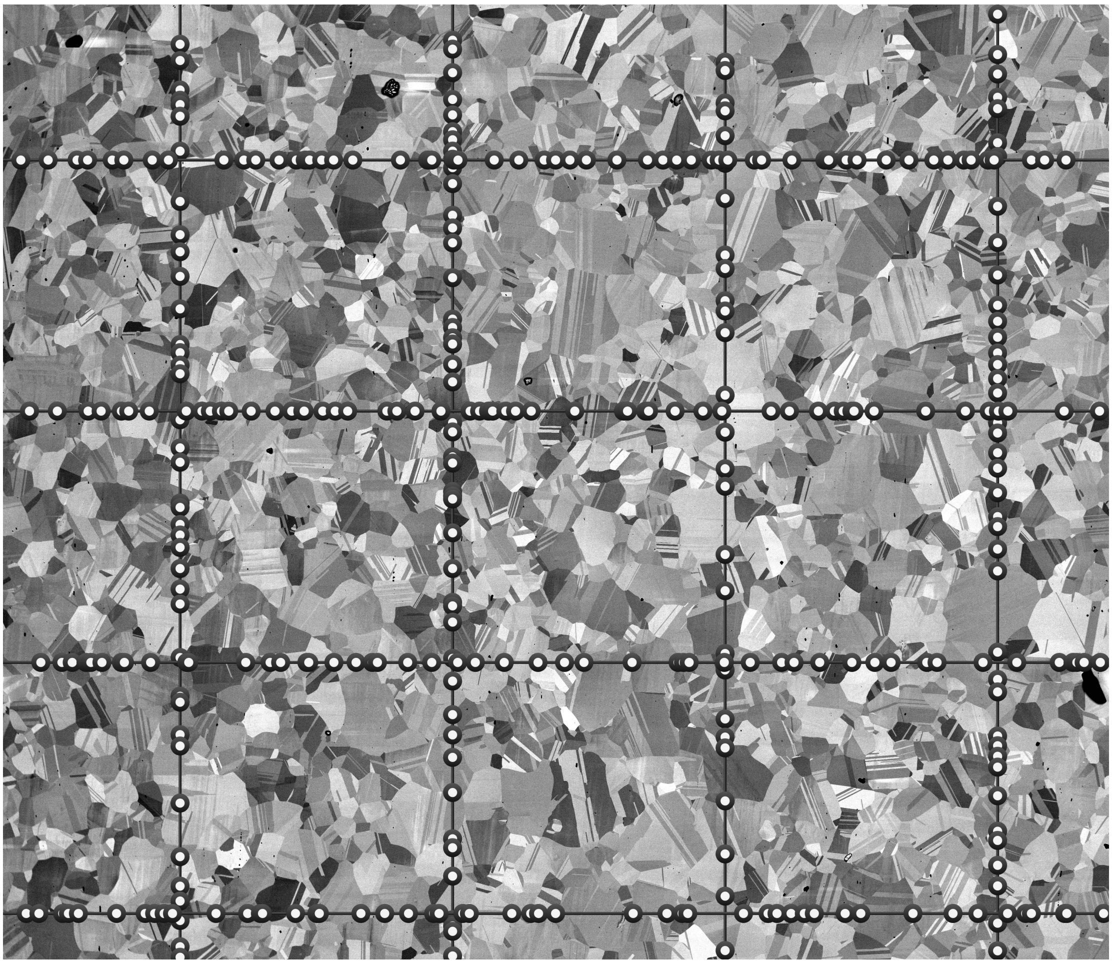

4.1. Statistical Analysis

Statistical Data	Length
Object Count	369
Minimum	2.1 µm
Maximum	124.0 µm
Average	34.1 µm
Standard deviation	22.1 µm
Skewness	0.0
Standard deviation (n-1)	22.2 µm
Variance	490.2 µm²
Variance (n-1)	491.6 µm²
Sum	12'596.7 µm
Sum of squares	610'917.3 µm²
Sum of cubes	37'022'349.1 µm³

4.1.1. Chord Lenght Distribution

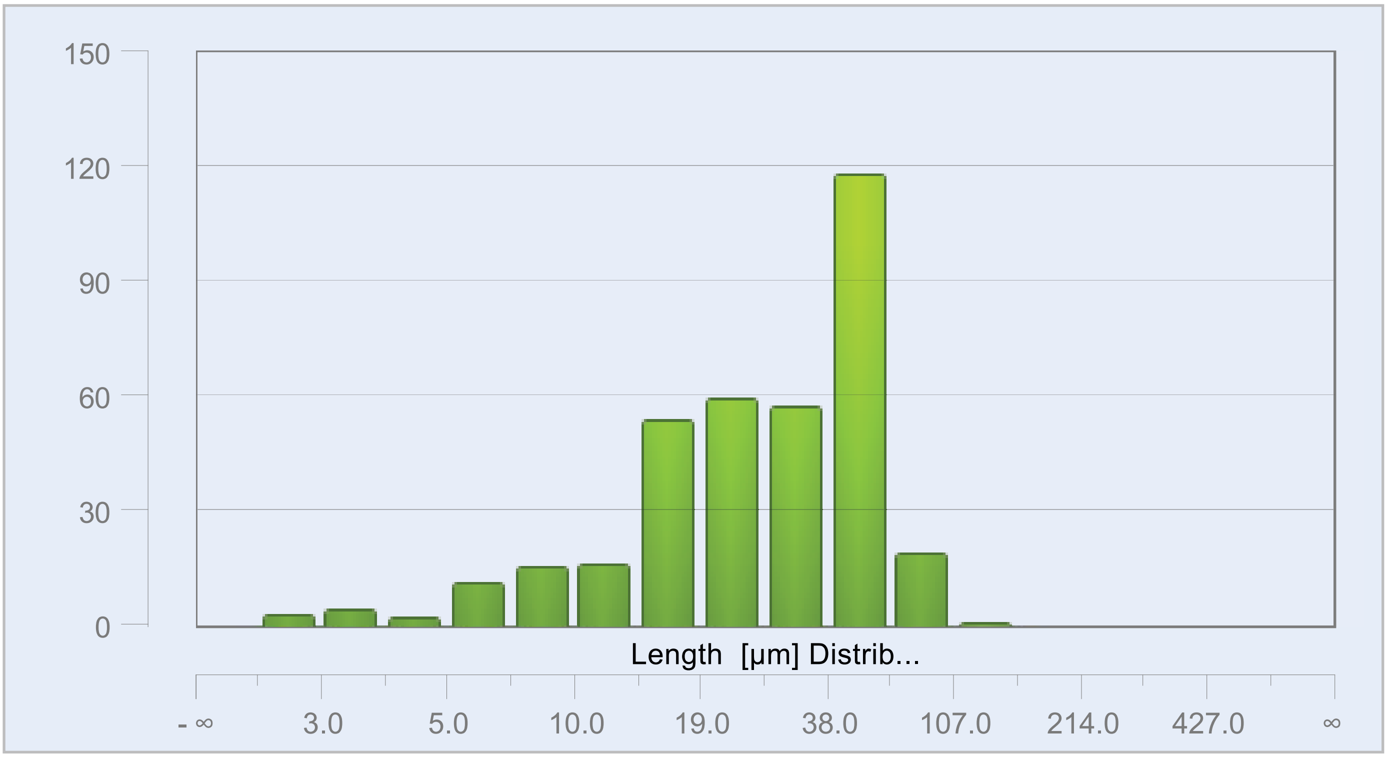

Start	End	Absolute Frequency	Absolute Frequency (accumulated)	Relative Frequency [%]	Relative Frequency (accumulated) [%]
	2.0 µm	0	0	0	0
2.0 µm	3.0 µm	4	4	1	1
3.0 µm	4.0 µm	5	9	1	2
4.0 µm	5.0 µm	3	12	1	3
5.0 µm	7.0 µm	12	24	3	7
7.0 µm	10.0 µm	16	40	4	11
10.0 µm	13.0 µm	17	57	5	15
13.0 µm	19.0 µm	54	111	15	30
19.0 µm	27.0 µm	60	171	16	46
27.0 µm	38.0 µm	58	229	16	62
38.0 µm	75.0 µm	118	347	32	94
75.0 µm	107.0 µm	20	367	5	99
107.0 µm	151.0 µm	2	369	1	100
151.0 µm	214.0 µm	0	369	0	100
214.0 µm	302.0 µm	0	369	0	100
302.0 µm	427.0 µm	0	369	0	100
427.0 µm	600.0 µm	0	369	0	100
600.0 µm		0	369	0	100

5. Single Result 4 (CrCoNi Twins grain size_ASTM 1000C 60min_00190)

Mean chord length	33.9 µm
Grain size (ASTM)	6.5
Grain size (G643)	6.4
Grain stretching	90.4 %

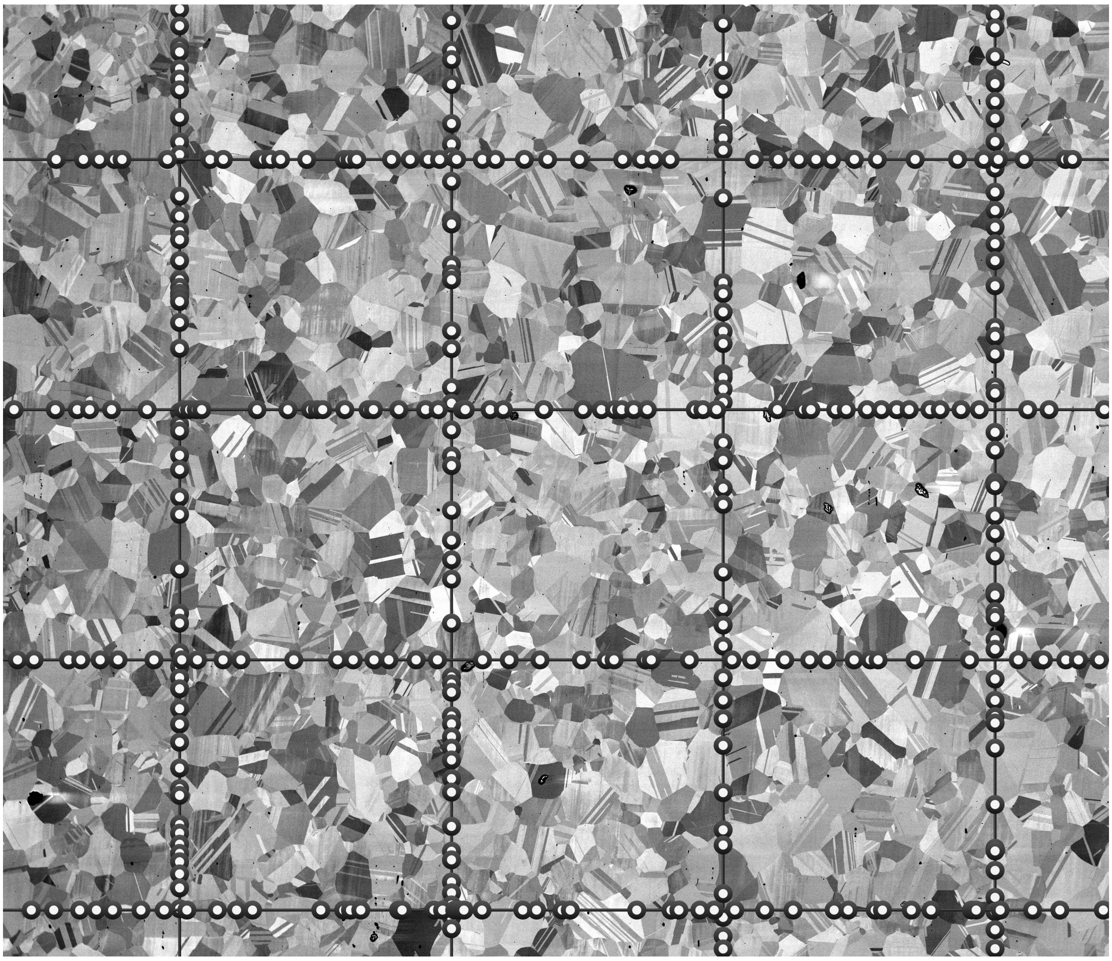

5.1. Statistical Analysis

Statistical Data	Length
Object Count	371
Minimum	2.1 µm
Maximum	129.6 µm
Average	33.9 µm
Standard deviation	21.6 µm
Skewness	0.0
Standard deviation (n-1)	21.6 µm
Variance	465.4 µm²
Variance (n-1)	466.6 µm²
Sum	12'586.3 µm
Sum of squares	599'649.4 µm²
Sum of cubes	36'521'563.7 µm³

5.1.1. Chord Lenght Distribution

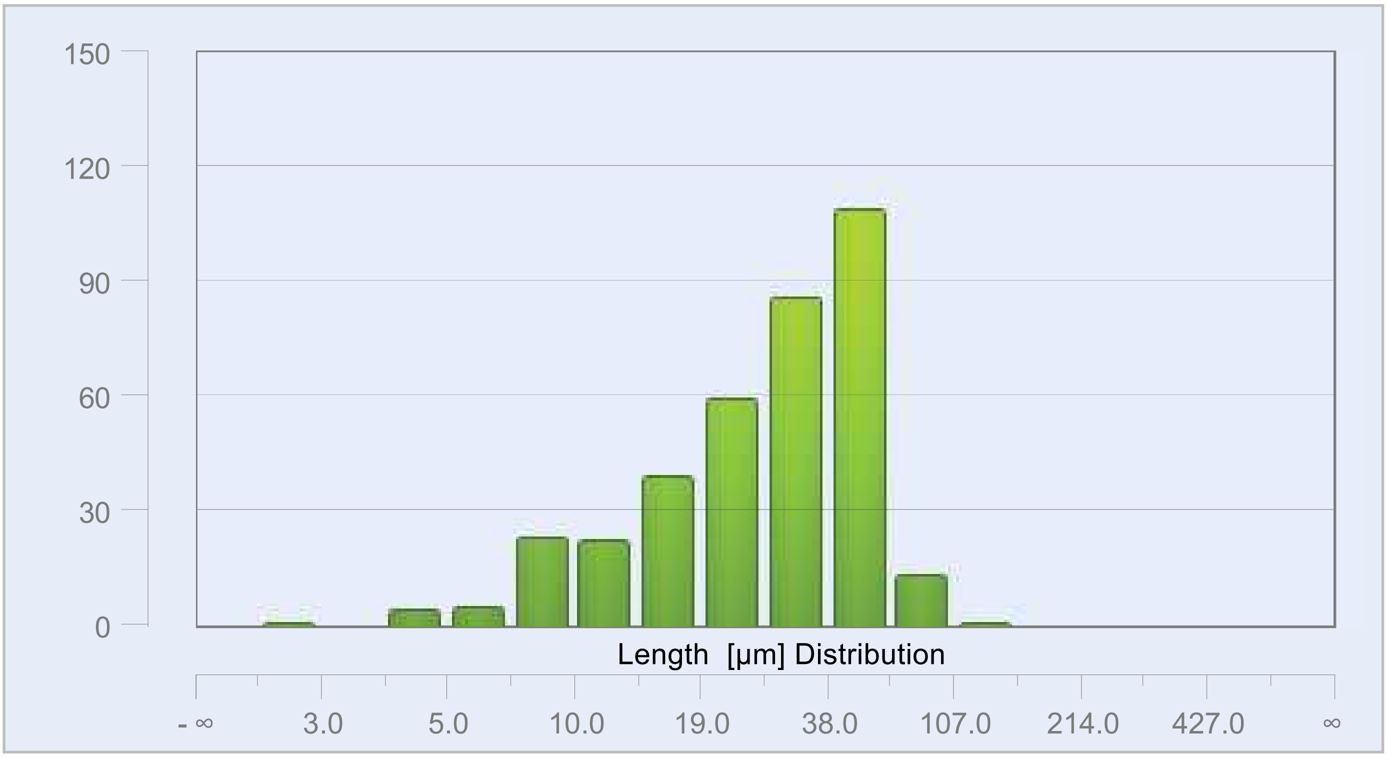

Start	End	Absolute Frequency	Absolute Frequency (accumulated)	Relative Frequency [%]	Relative Frequency (accumulated) [%]
	2.0 µm	0	0	0	0
2.0 µm	3.0 µm	2	2	1	1
3.0 µm	4.0 µm	0	2	0	1
4.0 µm	5.0 µm	5	7	1	2
5.0 µm	7.0 µm	6	13	2	4
7.0 µm	10.0 µm	24	37	6	10
10.0 µm	13.0 µm	23	60	6	16
13.0 µm	19.0 µm	40	100	11	27
19.0 µm	27.0 µm	60	160	16	43
27.0 µm	38.0 µm	86	246	23	66
38.0 µm	75.0 µm	109	355	29	96
75.0 µm	107.0 µm	14	369	4	99
107.0 µm	151.0 µm	2	371	1	100
151.0 µm	214.0 µm	0	371	0	100
214.0 µm	302.0 µm	0	371	0	100
302.0 µm	427.0 µm	0	371	0	100
427.0 µm	600.0 µm	0	371	0	100
600.0 µm		0	371	0	100
