# Supplementary material for: Benchmark dataset of the effect of grain size on strength in the single-phase FCC CrCoNi medium entropy alloy
Source: Data Brief. 2019 Oct 1;27:104592. doi: 10.1016/j.dib.2019.104592 (PMC6812030; doi:10.1016/j.dib.2019.104592)
Supplement: Multimedia component 1 [file mmc1.zip › CrCoNi_1373K_30min/CrCoNi_1373K_30min_c=23μm.pdf]

Analysebericht

02.11.2017 12:40:42

powered by imagic.ch

1. cumulative Result 1

Anzahl Bilder	1
Korngröße (ASTM)	7,7
Korngröße (G643)	7,6
Kornstreckung	89,4 %
Mittlere Sehnenlänge	22,5 µm

2. Single Result 1 (CrCoNi - ASTM E 112_CrCoNi_homogenized_8.1mmSW_1100C_30min_00005)

Mittlere Sehnenlänge	22,5 µm
Korngröße (ASTM)	7,7
Korngröße (G643)	7,6
Kornstreckung	89,4 %

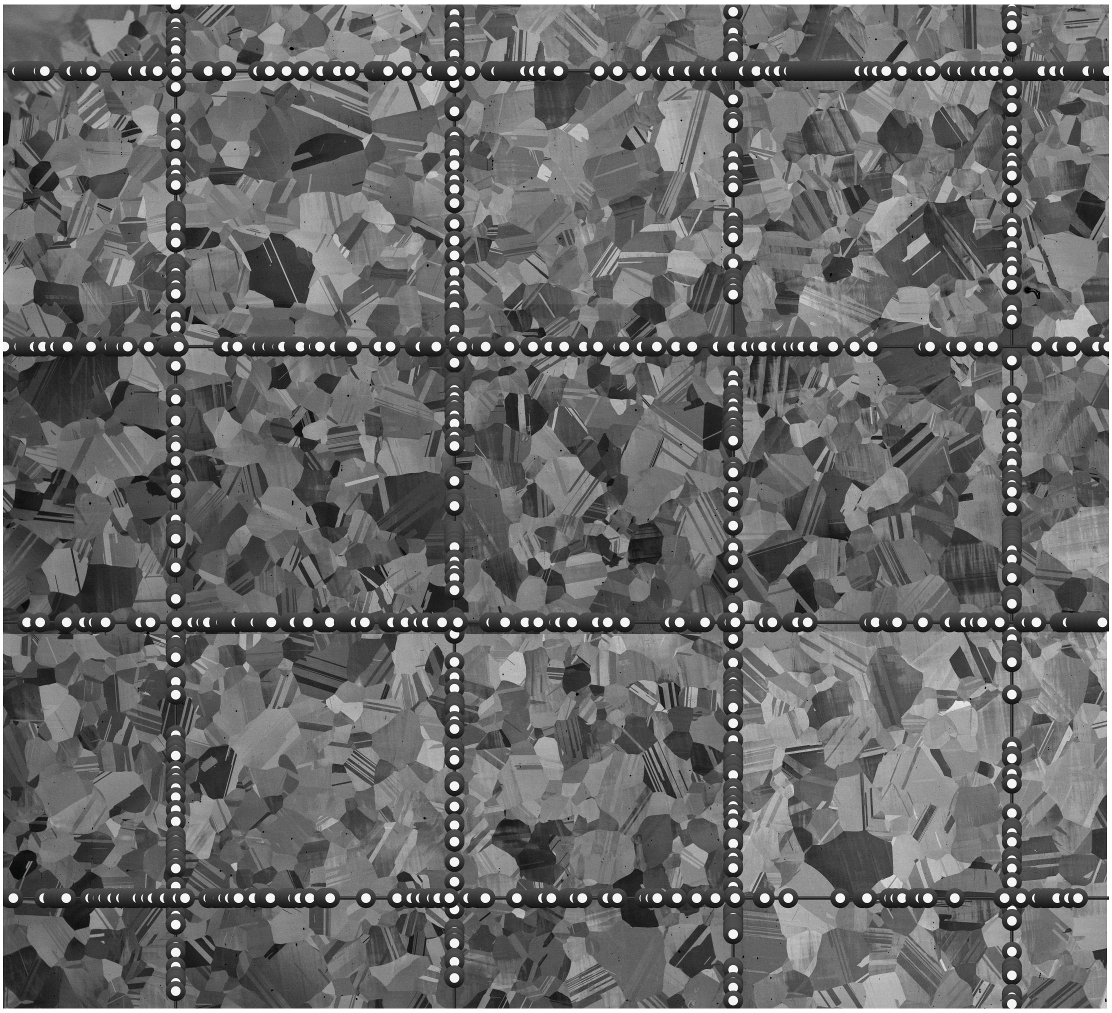

2.1. Statistische Analyse

Statistische Daten	Länge
Anzahl Objekte	1000
Minimum	1,2 µm
Maximum	164,1 µm
Mittelwert	22,5 µm
Standardabweichung	23,0 µm
Schiefe	0,0
Standardabweichung (n-1)	23,0 µm
Varianz	528,9 µm²
Varianz (n-1)	529,4 µm²

Statistische Daten	Länge
Summe	22'525,7 µm
Quadratsumme	1'036'289,4 µm²
Kubiksumme	70'842'542,9 µm³

2.1.1. Chord Lenght Distribution

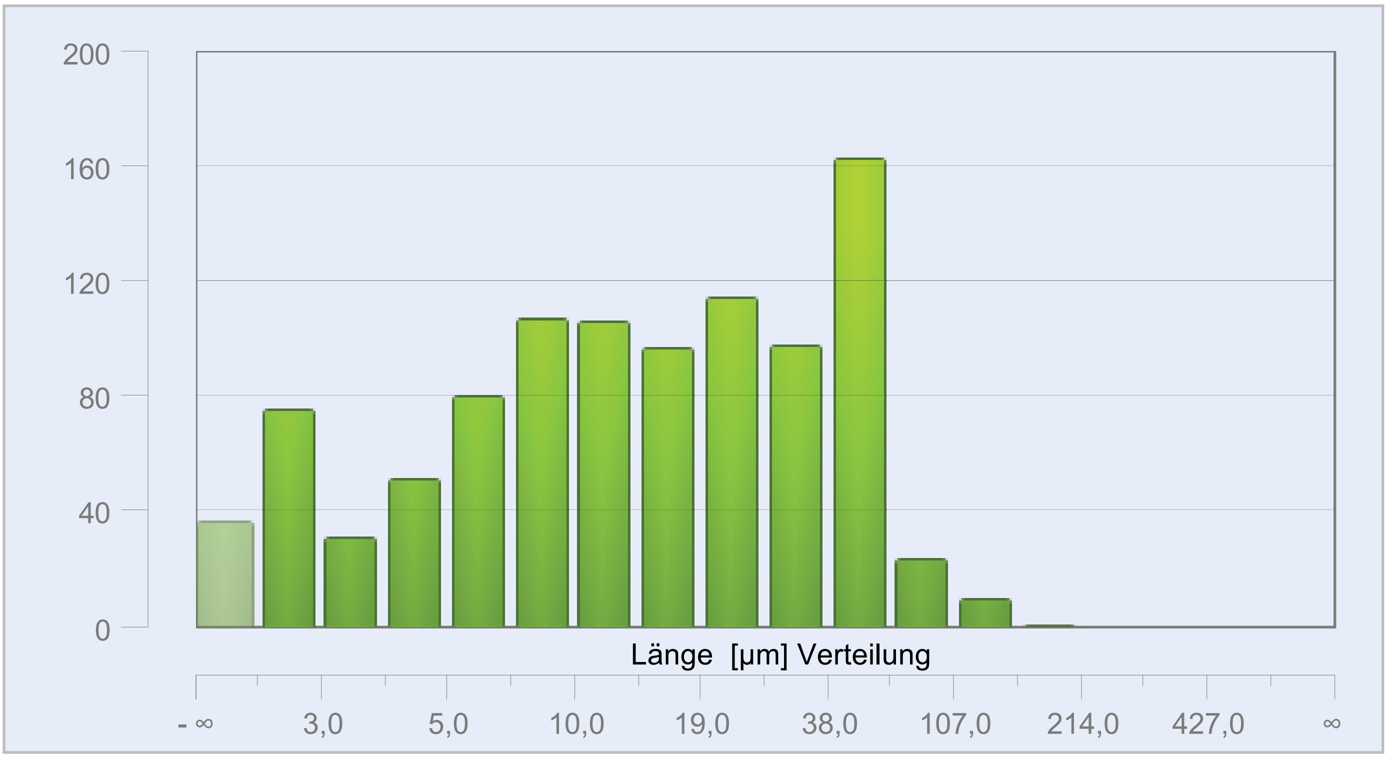

Start	Ende	Absolute Häufigkeit	Absolute Häufigkeit (kumuliert)	Relative Häufigkeit [%]	Relative Häufigkeit (kumuliert) [%]
	2,0 µm	37	37	4	4
2,0 µm	3,0 µm	76	113	8	11
3,0 µm	4,0 µm	32	145	3	14
4,0 µm	5,0 µm	52	197	5	20
5,0 µm	7,0 µm	81	278	8	28
7,0 µm	10,0 µm	107	385	11	38
10,0 µm	13,0 µm	106	491	11	49
13,0 µm	19,0 µm	97	588	10	59
19,0 µm	27,0 µm	115	703	12	70
27,0 µm	38,0 µm	98	801	10	80
38,0 µm	75,0 µm	163	964	16	96
75,0 µm	107,0 µm	24	988	2	99
107,0 µm	151,0 µm	11	999	1	100
151,0 µm	214,0 µm	1	1000	0	100
214,0 µm	302,0 µm	0	1000	0	100
302,0 µm	427,0 µm	0	1000	0	100
427,0 µm	600,0 µm	0	1000	0	100
600,0 µm		0	1000	0	100
