# Supplementary material for: Benchmark dataset of the effect of grain size on strength in the single-phase FCC CrCoNi medium entropy alloy
Source: Data Brief. 2019 Oct 1;27:104592. doi: 10.1016/j.dib.2019.104592 (PMC6812030; doi:10.1016/j.dib.2019.104592)
Supplement: Multimedia component 1 [file mmc1.zip › CrCoNi_1373K_30min/CrCoNi_1373K_30min_d=61μm.pdf]

Analysebericht

27.08.2017 21:57:20

powered by imagic.ch

1. cumulative Result 1

Anzahl Bilder	1
Korngröße (ASTM)	4,8
Korngröße (G643)	4,8
Kornstreckung	89,7 %
Mittlere Sehnenlänge	60,6 µm

2. Single Result 1 (CrCoNi - ASTM E 112_CrCoNi_homogenized_8.1mmSW_1100C_30min_00005)

Mittlere Sehnenlänge	60,6 µm
Korngröße (ASTM)	4,8
Korngröße (G643)	4,8
Kornstreckung	89,7 %

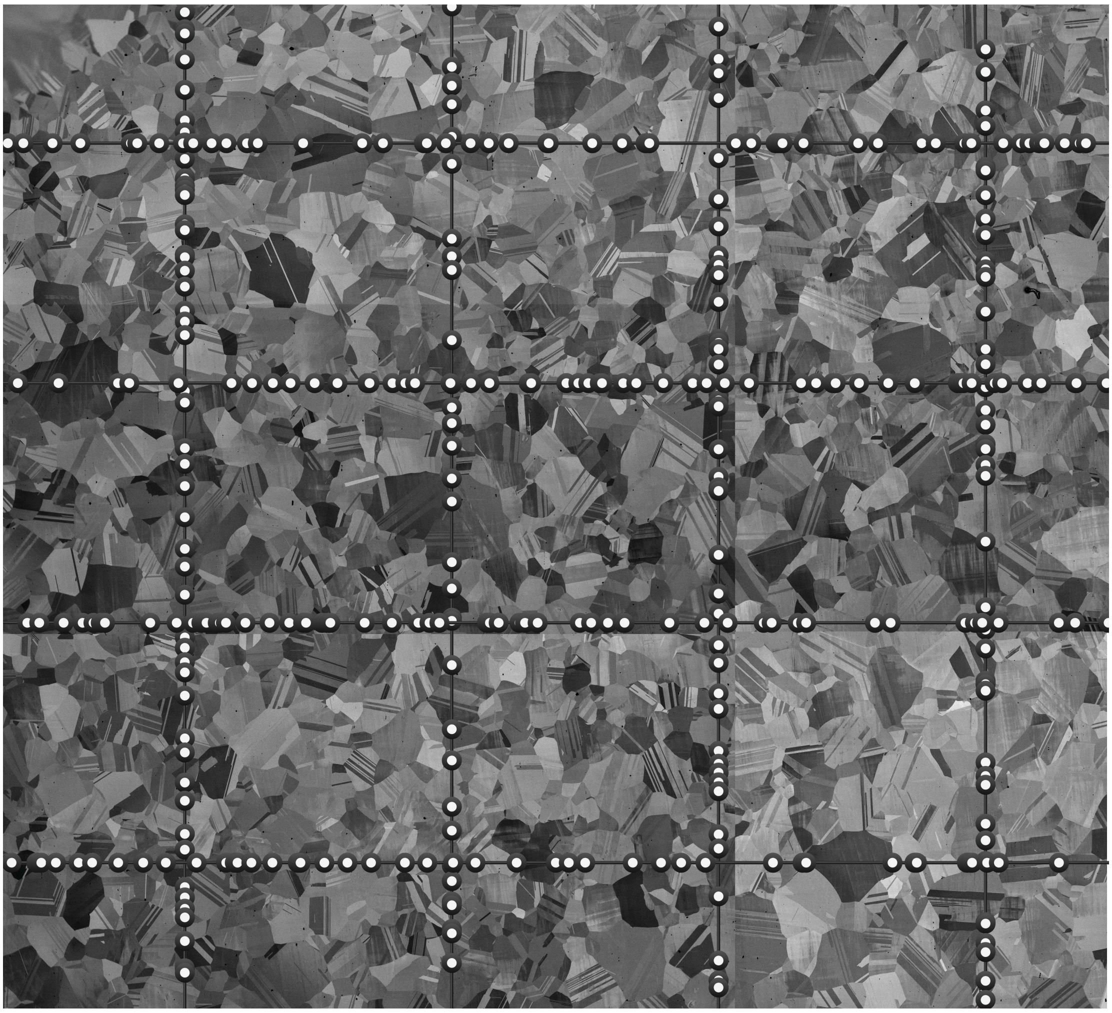

2.1. Statistische Analyse

Statistische Daten	Länge
Anzahl Objekte	372
Minimum	0,6 µm
Maximum	230,8 µm
Mittelwert	60,6 µm
Standardabweichung	44,1 µm
Schiefe	0,0
Standardabweichung (n-1)	44,2 µm
Varianz	1'947,3 µm²
Varianz (n-1)	1'952,6 µm²

Statistische Daten	Länge
Summe	22'525,7 µm
Quadratsumme	2'088'405,9 µm²
Kubiksumme	251'413'189,4 µm³

2.1.1. Chord Lenght Distribution

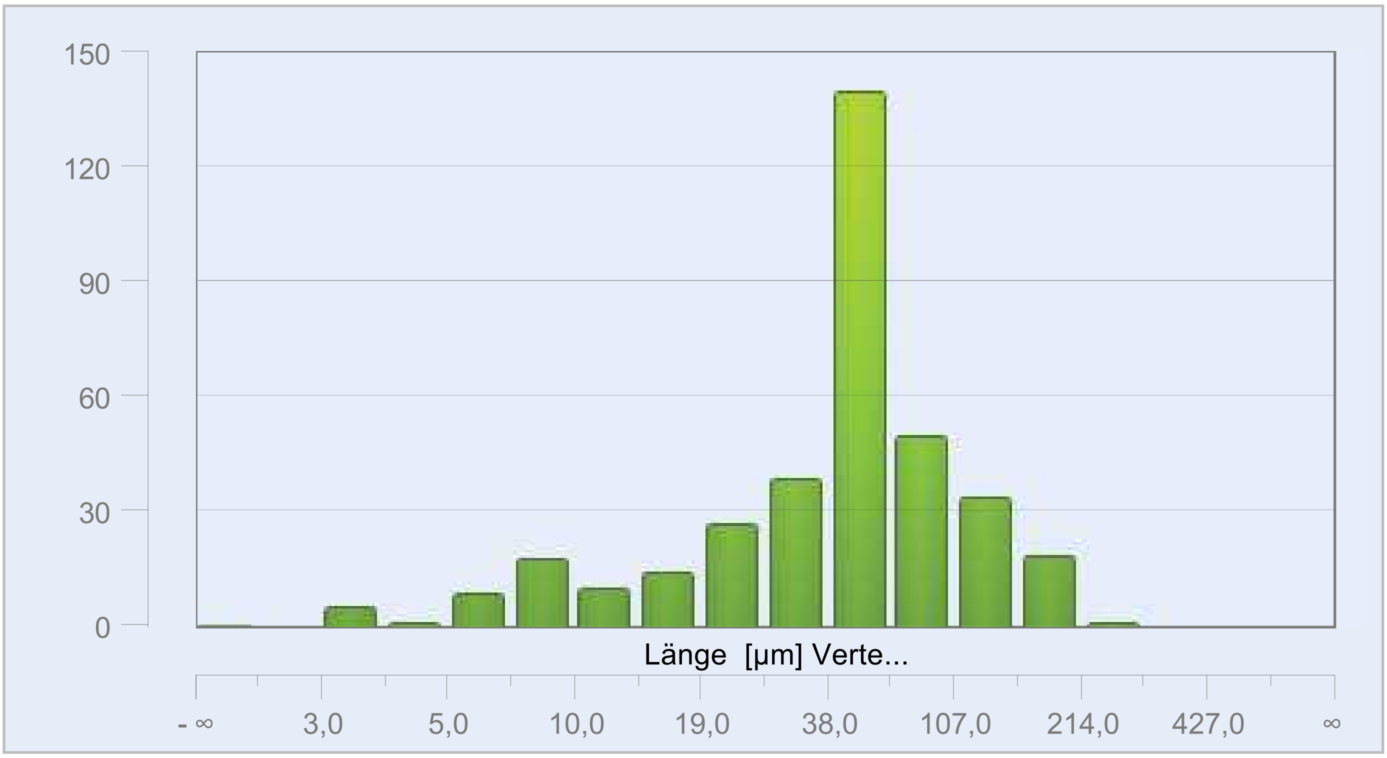

Start	Ende	Absolute Häufigkeit	Absolute Häufigkeit (kumuliert)	Relative Häufigkeit [%]	Relative Häufigkeit (kumuliert) [%]
	2,0 µm	1	1	0	0
2,0 µm	3,0 µm	0	1	0	0
3,0 µm	4,0 µm	6	7	2	2
4,0 µm	5,0 µm	2	9	1	2
5,0 µm	7,0 µm	9	18	2	5
7,0 µm	10,0 µm	18	36	5	10
10,0 µm	13,0 µm	11	47	3	13
13,0 µm	19,0 µm	15	62	4	17
19,0 µm	27,0 µm	27	89	7	24
27,0 µm	38,0 µm	39	128	10	34
38,0 µm	75,0 µm	139	267	37	72
75,0 µm	107,0 µm	50	317	13	85
107,0 µm	151,0 µm	34	351	9	94
151,0 µm	214,0 µm	19	370	5	99
214,0 µm	302,0 µm	2	372	1	100
302,0 µm	427,0 µm	0	372	0	100
427,0 µm	600,0 µm	0	372	0	100
600,0 µm		0	372	0	100
