# Supplementary material for: Benchmark dataset of the effect of grain size on strength in the single-phase FCC CrCoNi medium entropy alloy
Source: Data Brief. 2019 Oct 1;27:104592. doi: 10.1016/j.dib.2019.104592 (PMC6812030; doi:10.1016/j.dib.2019.104592)
Supplement: Multimedia component 1 [file mmc1.zip › CrCoNi_1473K_30240min/CrCoNi_1473K_30240min_c=50μm.pdf]

# Analysebericht

Aug 31, 2017 1:55:20 PM

powered by imagic.ch

## 1. cumulative Result 1

| | |
|---|---|
| Number of images | 1 |
| Grain size (ASTM) | 5.3 |
| Grain size (G643) | 5.3 |
| Grain stretching | 78.2 % |
| Mean chord length | 50.1 µm |

## 2. Single Result 1 (CrCoNi - ASTM E 112_CrCoNi_homogenized_8.1mmSW_1200_3Weeks_00026)

| | |
|---|---|
| Mean chord length | 50.1 µm |
| Grain size (ASTM) | 5.3 |
| Grain size (G643) | 5.3 |
| Grain stretching | 78.2 % |

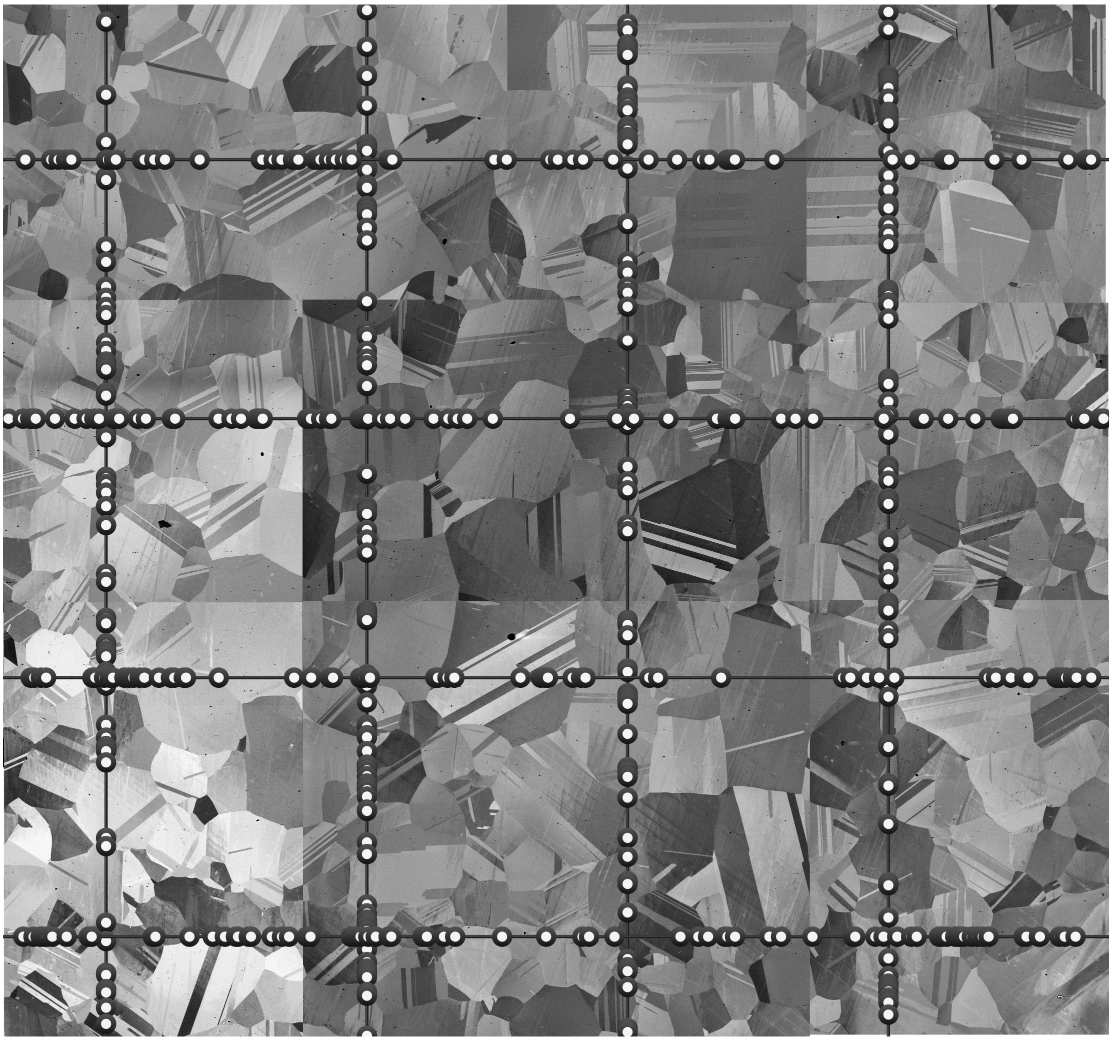

### 2.1. Statistical Analysis

| Statistical Data | Length |
|---|---|
| Object Count | 570 |
| Minimum | 1.5 µm |
| Maximum | 407.3 µm |
| Average | 50.1 µm |
| Standard deviation | 59.6 µm |
| Skewness | 0.0 |
| Standard deviation (n-1) | 59.7 µm |
| Variance | 3'556.9 µm² |

| Statistical Data | Length |
|---:|---:|
| Variance (n-1) | 3'563.2 µm² |
| Sum | 28'567.5 µm |
| Sum of squares | 3'459'193.3 µm² |
| Sum of cubes | 651'813'502.2 µm³ |

### 2.1.1. Chord Lenght Distribution

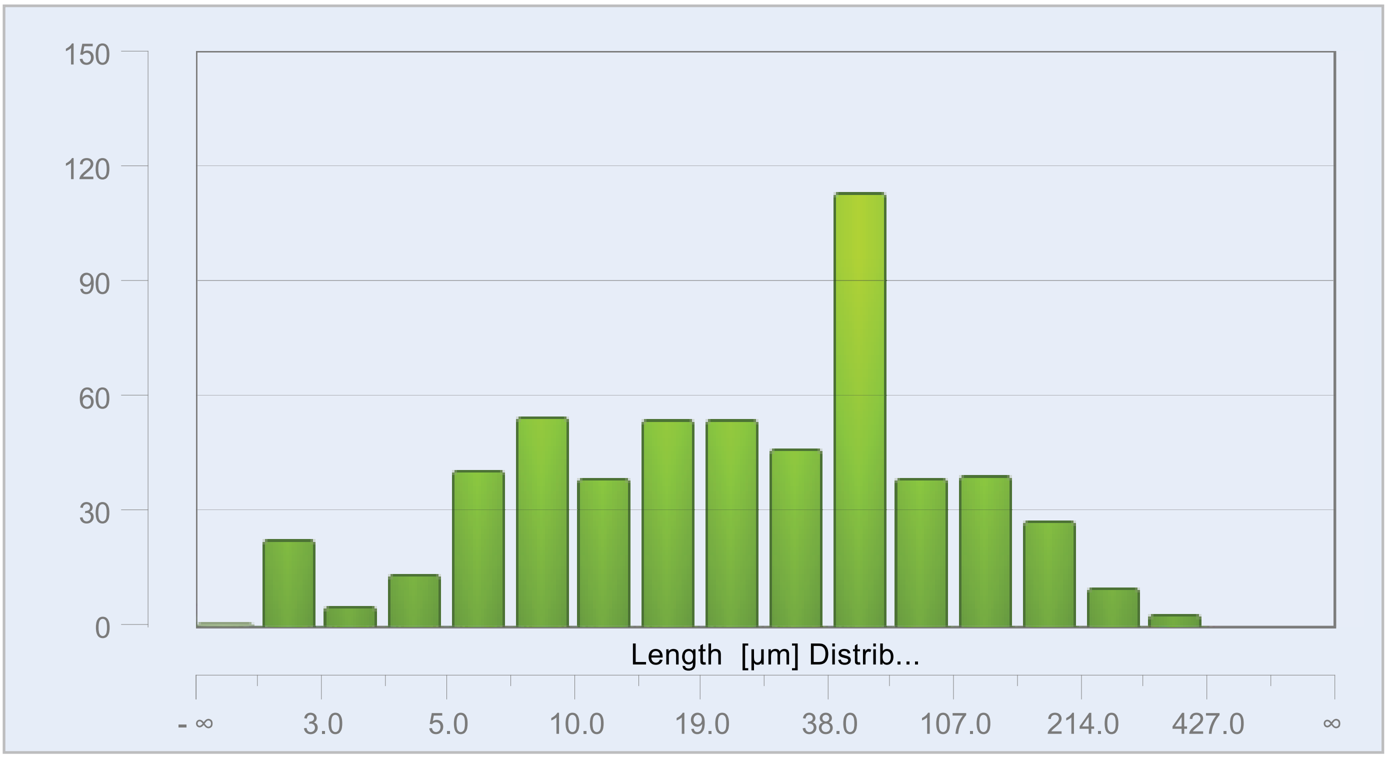


| Start | End | Absolute Frequency | Absolute Frequency (accumulated) | Relative Frequency [%] | Relative Frequency (accumulated) [%] |
|---:|---:|---:|---:|---:|---:|
| | 2.0 µm | 2 | 2 | 0 | 0 |
| 2.0 µm | 3.0 µm | 23 | 25 | 4 | 4 |
| 3.0 µm | 4.0 µm | 6 | 31 | 1 | 5 |
| 4.0 µm | 5.0 µm | 14 | 45 | 2 | 8 |
| 5.0 µm | 7.0 µm | 41 | 86 | 7 | 15 |
| 7.0 µm | 10.0 µm | 55 | 141 | 10 | 25 |
| 10.0 µm | 13.0 µm | 39 | 180 | 7 | 32 |
| 13.0 µm | 19.0 µm | 54 | 234 | 9 | 41 |
| 19.0 µm | 27.0 µm | 54 | 288 | 9 | 51 |
| 27.0 µm | 38.0 µm | 47 | 335 | 8 | 59 |
| 38.0 µm | 75.0 µm | 113 | 448 | 20 | 79 |
| 75.0 µm | 107.0 µm | 39 | 487 | 7 | 85 |
| 107.0 µm | 151.0 µm | 40 | 527 | 7 | 92 |
| 151.0 µm | 214.0 µm | 28 | 555 | 5 | 97 |
| 214.0 µm | 302.0 µm | 11 | 566 | 2 | 99 |
| 302.0 µm | 427.0 µm | 4 | 570 | 1 | 100 |
| 427.0 µm | 600.0 µm | 0 | 570 | 0 | 100 |
| 600.0 µm | | 0 | 570 | 0 | 100 |
