# Supplementary material for: Benchmark dataset of the effect of grain size on strength in the single-phase FCC CrCoNi medium entropy alloy
Source: Data Brief. 2019 Oct 1;27:104592. doi: 10.1016/j.dib.2019.104592 (PMC6812030; doi:10.1016/j.dib.2019.104592)
Supplement: Multimedia component 1 [file mmc1.zip › CrCoNi_1473K_30240min/CrCoNi_1473K_30240min_d=174μm.pdf]

Analysebericht

Aug 30, 2017 2:39:19 PM

powered by imagic.ch

1. cumulative Result 1

Number of images	1
Grain size (ASTM)	1.8
Grain size (G643)	1.7
Grain stretching	84.6 %
Mean chord length	174.2 µm

2. Single Result 1 (CrCoNi - ASTM E 112_CrCoNi_homogenized_8.1mmSW_1200_3Weeks_00026)

Mean chord length	174.2 µm
Grain size (ASTM)	1.8
Grain size (G643)	1.7
Grain stretching	84.6 %

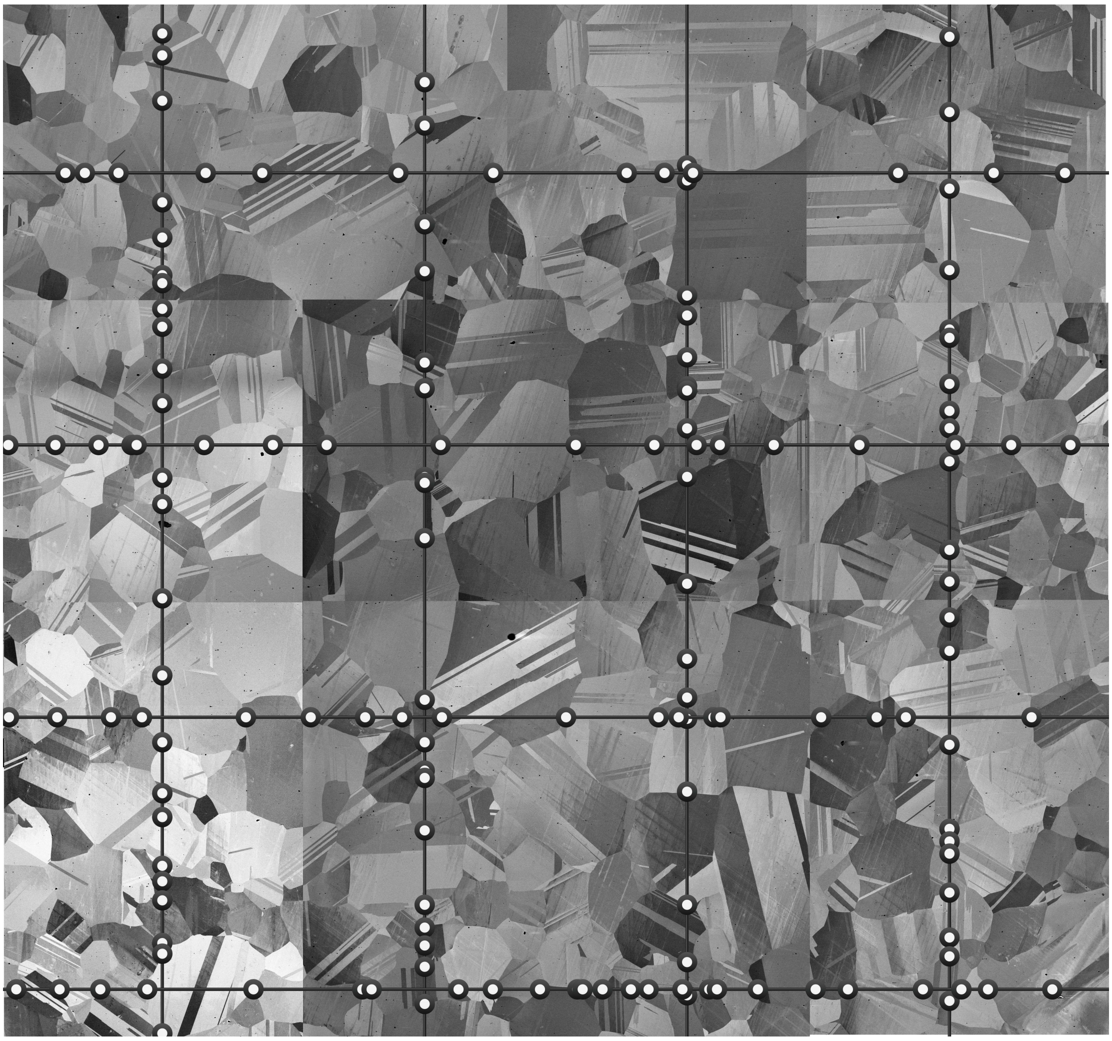

2.1. Statistical Analysis

Statistical Data	Length
Object Count	164
Minimum	9.6 µm
Maximum	685.3 µm
Average	174.2 µm
Standard deviation	118.9 µm
Skewness	0.0
Standard deviation (n-1)	119.2 µm
Variance	14'130.2 µm²

Statistical Data	Length
Variance (n-1)	14'216.9 μm²
Sum	28'567.5 μm
Sum of squares	7'293'564.5 μm²
Sum of cubes	2'408'505'342.4 μm³

2.1.1. Chord Lenght Distribution

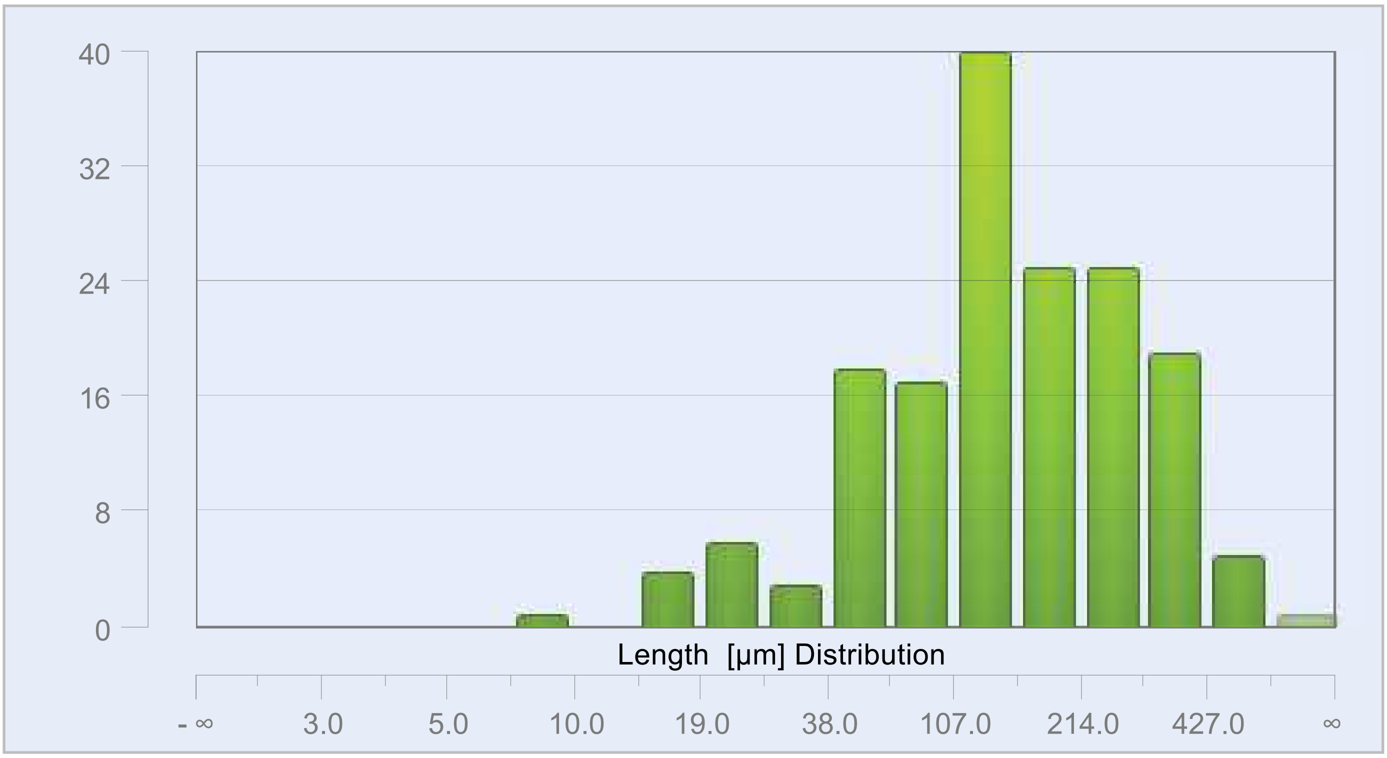

Start	End	Absolute Frequency	Absolute Frequency (accumulated)	Relative Frequency [%]	Relative Frequency (accumulated) [%]
	2.0 μm	0	0	0	0
2.0 μm	3.0 μm	0	0	0	0
3.0 μm	4.0 μm	0	0	0	0
4.0 μm	5.0 μm	0	0	0	0
5.0 μm	7.0 μm	0	0	0	0
7.0 μm	10.0 μm	1	1	1	1
10.0 μm	13.0 μm	0	1	0	1
13.0 μm	19.0 μm	4	5	2	3
19.0 μm	27.0 μm	6	11	4	7
27.0 μm	38.0 μm	3	14	2	9
38.0 μm	75.0 μm	18	32	11	20
75.0 μm	107.0 μm	17	49	10	30
107.0 μm	151.0 μm	40	89	24	54
151.0 μm	214.0 μm	25	114	15	70
214.0 μm	302.0 μm	25	139	15	85
302.0 μm	427.0 μm	19	158	12	96
427.0 μm	600.0 μm	5	163	3	99
600.0 μm		1	164	1	100
