# Supplementary material for: Benchmark dataset of the effect of grain size on strength in the single-phase FCC CrCoNi medium entropy alloy
Source: Data Brief. 2019 Oct 1;27:104592. doi: 10.1016/j.dib.2019.104592 (PMC6812030; doi:10.1016/j.dib.2019.104592)
Supplement: Multimedia component 1 [file mmc1.zip › CrCoNi_1473K_30min/CrCoNi_1473K_30min_c=42μm.pdf]

Analysebericht

Aug 31, 2017 6:20:28 PM

powered by imagic.ch

1. cumulative Result 1

Number of images	1
Grain size (ASTM)	5.9
Grain size (G643)	5.8
Grain stretching	98.9 %
Mean chord length	41.6 µm

2. Single Result 1 (CrCoNi - ASTM E 112_CrCoNi_homogenized_8.1mmSW_1200C_30min_00025)

Mean chord length	41.6 µm
Grain size (ASTM)	5.9
Grain size (G643)	5.8
Grain stretching	98.9 %

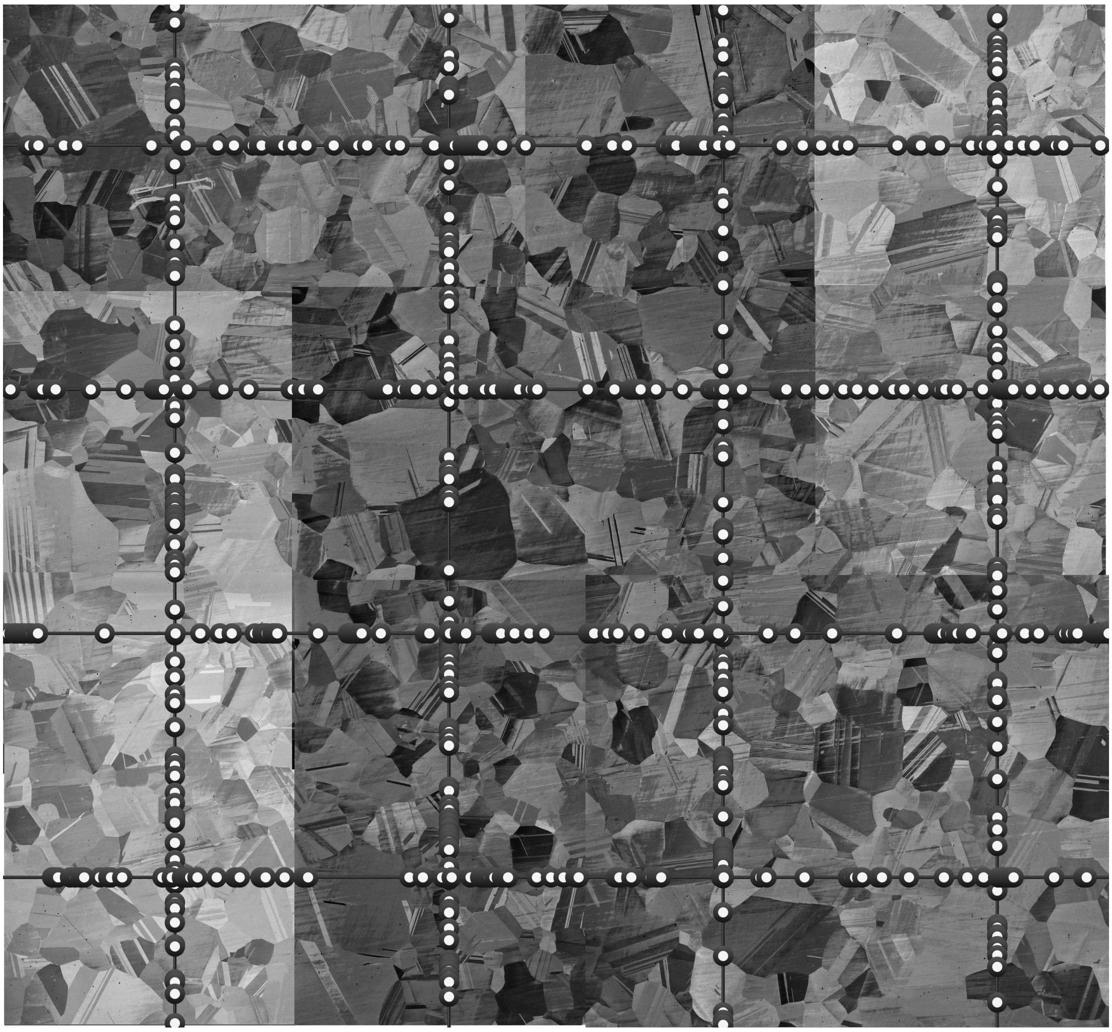

2.1. Statistical Analysis

Statistical Data	Length
Object Count	710
Minimum	0.8 µm
Maximum	351.2 µm
Average	41.6 µm
Standard deviation	45.9 µm
Skewness	0.0
Standard deviation (n-1)	45.9 µm
Variance	2'102.7 µm²
Variance (n-1)	2'105.7 µm²

Statistical Data	Length
Sum	29'509.7 µm
Sum of squares	2'719'416.8 µm²
Sum of cubes	372'849'017.0 µm³

2.1.1. Chord Lenght Distribution

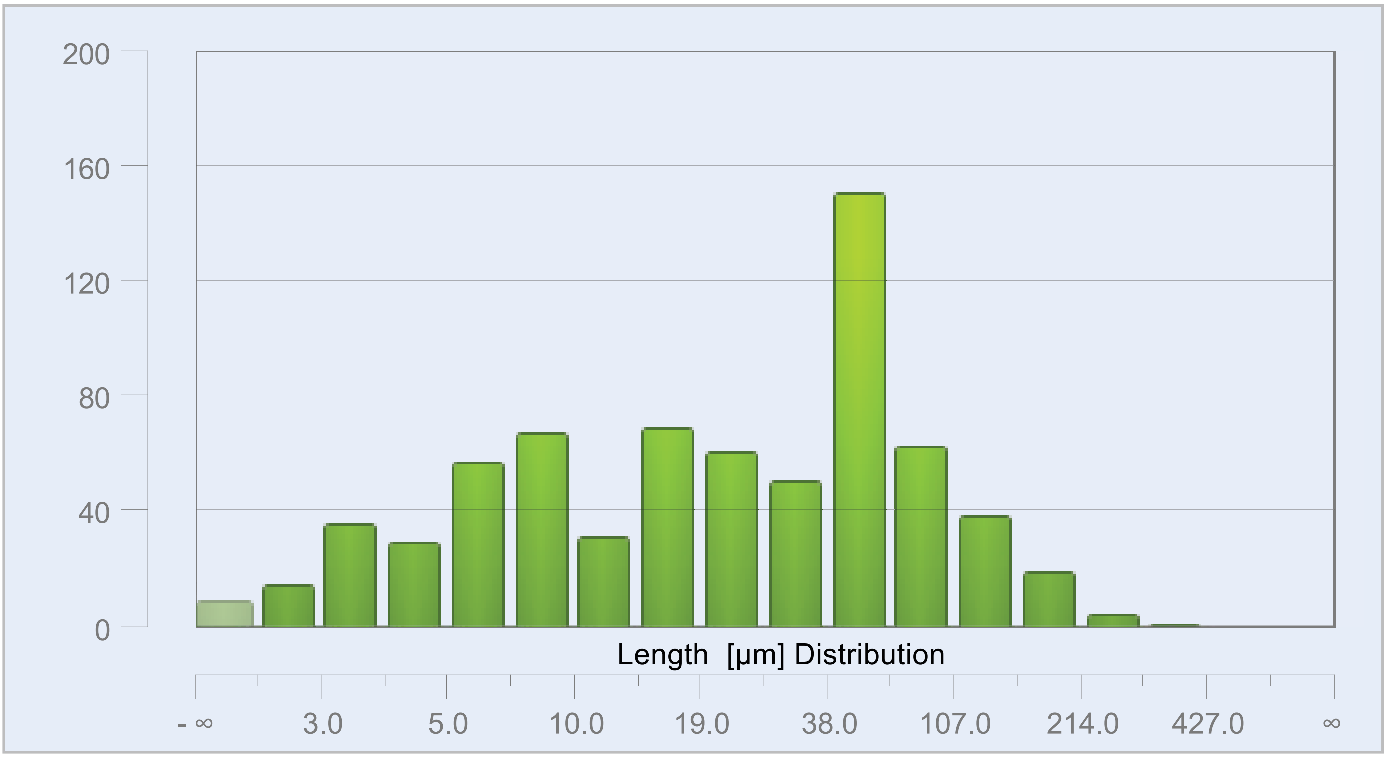

Start	End	Absolute Frequency	Absolute Frequency (accumulated)	Relative Frequency [%]	Relative Frequency (accumulated) [%]
	2.0 µm	10	10	1	1
2.0 µm	3.0 µm	15	25	2	4
3.0 µm	4.0 µm	36	61	5	9
4.0 µm	5.0 µm	30	91	4	13
5.0 µm	7.0 µm	58	149	8	21
7.0 µm	10.0 µm	68	217	10	31
10.0 µm	13.0 µm	32	249	5	35
13.0 µm	19.0 µm	70	319	10	45
19.0 µm	27.0 µm	61	380	9	54
27.0 µm	38.0 µm	51	431	7	61
38.0 µm	75.0 µm	151	582	21	82
75.0 µm	107.0 µm	63	645	9	91
107.0 µm	151.0 µm	39	684	5	96
151.0 µm	214.0 µm	20	704	3	99
214.0 µm	302.0 µm	5	709	1	100
302.0 µm	427.0 µm	1	710	0	100
427.0 µm	600.0 µm	0	710	0	100
600.0 µm		0	710	0	100
