# Supplementary material for: Benchmark dataset of the effect of grain size on strength in the single-phase FCC CrCoNi medium entropy alloy
Source: Data Brief. 2019 Oct 1;27:104592. doi: 10.1016/j.dib.2019.104592 (PMC6812030; doi:10.1016/j.dib.2019.104592)
Supplement: Multimedia component 1 [file mmc1.zip › CrCoNi_1473K_30min/CrCoNi_1473K_30min_d=115μm.pdf]

Analysebericht

Aug 29, 2017 4:48:30 PM

powered by imagic.ch

1. cumulative Result 1

Number of images	1
Grain size (ASTM)	2.9
Grain size (G643)	2.9
Grain stretching	95.4 %
Mean chord length	115.3 µm

2. Single Result 1 (CrCoNi - ASTM E 112_CrCoNi_homogenized_8.1mmSW_1200C_30min_00025)

Mean chord length	115.3 µm
Grain size (ASTM)	2.9
Grain size (G643)	2.9
Grain stretching	95.4 %

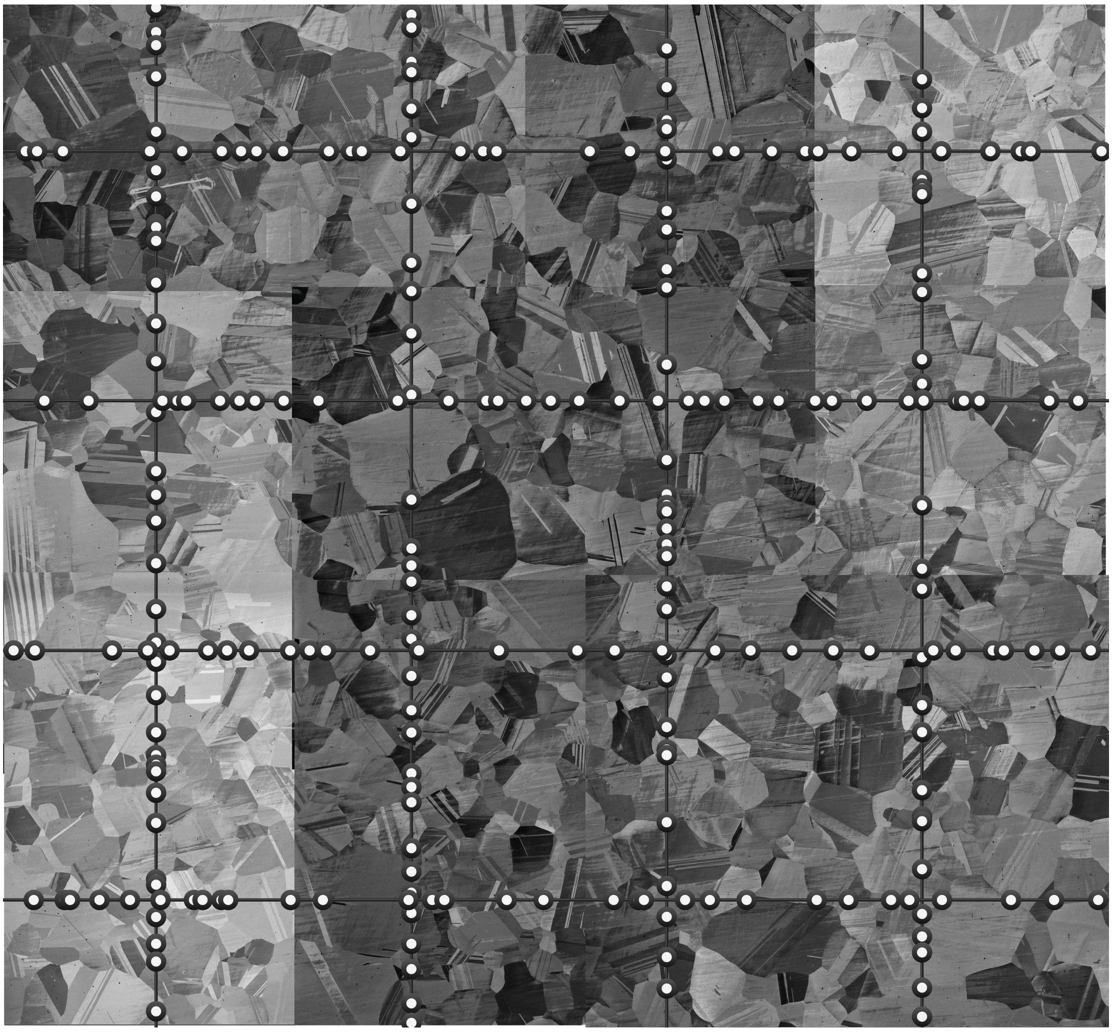

2.1. Statistical Analysis

Statistical Data	Length
Object Count	256
Minimum	10.7 µm
Maximum	366.6 µm
Average	115.3 µm
Standard deviation	69.7 µm
Skewness	0.0
Standard deviation (n-1)	69.8 µm
Variance	4'855.4 µm²
Variance (n-1)	4'874.5 µm²

Statistical Data	Length
Sum	29'509.7 µm
Sum of squares	4'644'624.7 µm²
Sum of cubes	910'842'518.9 µm³

2.1.1. Chord Lenght Distribution

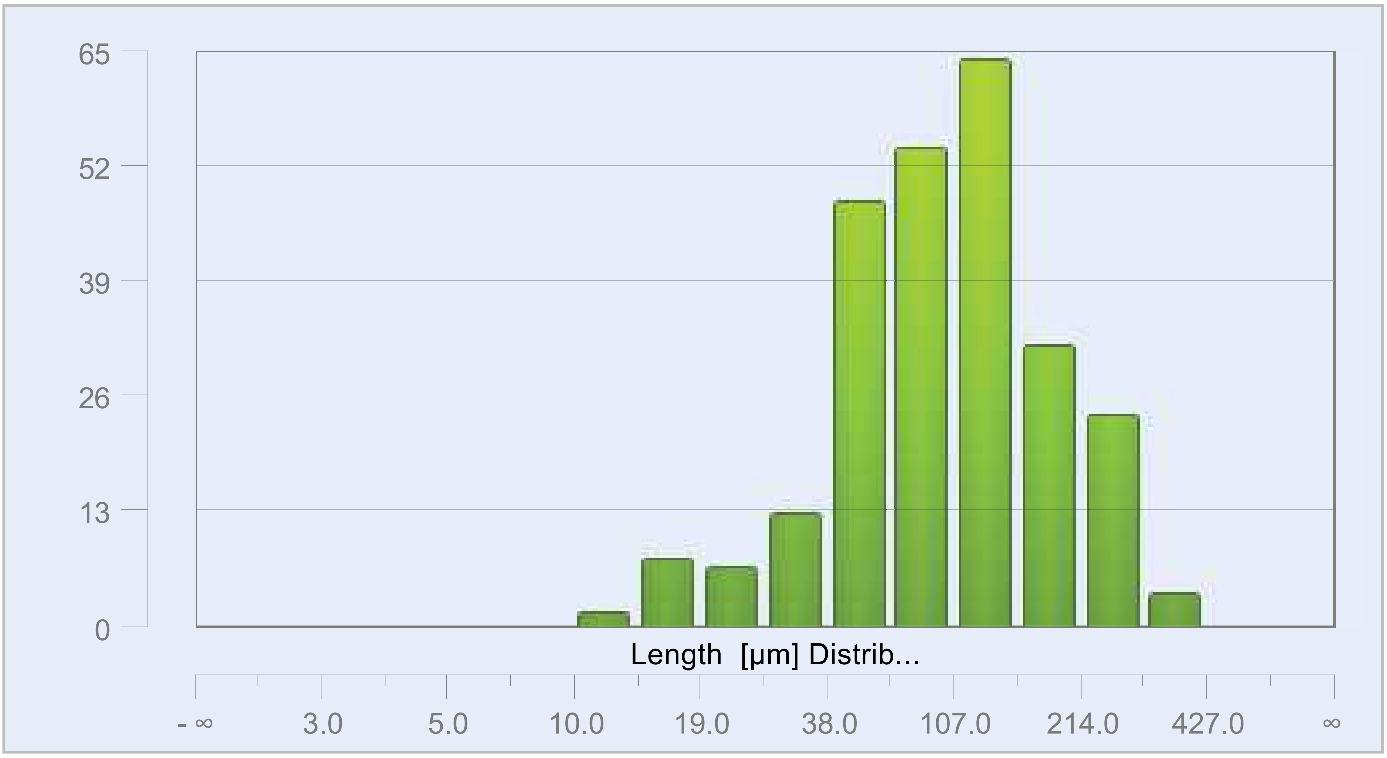

Start	End	Absolute Frequency	Absolute Frequency (accumulated)	Relative Frequency [%]	Relative Frequency (accumulated) [%]
	2.0 µm	0	0	0	0
2.0 µm	3.0 µm	0	0	0	0
3.0 µm	4.0 µm	0	0	0	0
4.0 µm	5.0 µm	0	0	0	0
5.0 µm	7.0 µm	0	0	0	0
7.0 µm	10.0 µm	0	0	0	0
10.0 µm	13.0 µm	2	2	1	1
13.0 µm	19.0 µm	8	10	3	4
19.0 µm	27.0 µm	7	17	3	7
27.0 µm	38.0 µm	13	30	5	12
38.0 µm	75.0 µm	48	78	19	30
75.0 µm	107.0 µm	54	132	21	52
107.0 µm	151.0 µm	64	196	25	77
151.0 µm	214.0 µm	32	228	12	89
214.0 µm	302.0 µm	24	252	9	98
302.0 µm	427.0 µm	4	256	2	100
427.0 µm	600.0 µm	0	256	0	100
600.0 µm		0	256	0	100
