# Supplementary figures and images for: Benchmark dataset of the effect of grain size on strength in the single-phase FCC CrCoNi medium entropy alloy
Source: Data Brief. 2019 Oct 1;27:104592. doi: 10.1016/j.dib.2019.104592 (PMC6812030; doi:10.1016/j.dib.2019.104592)

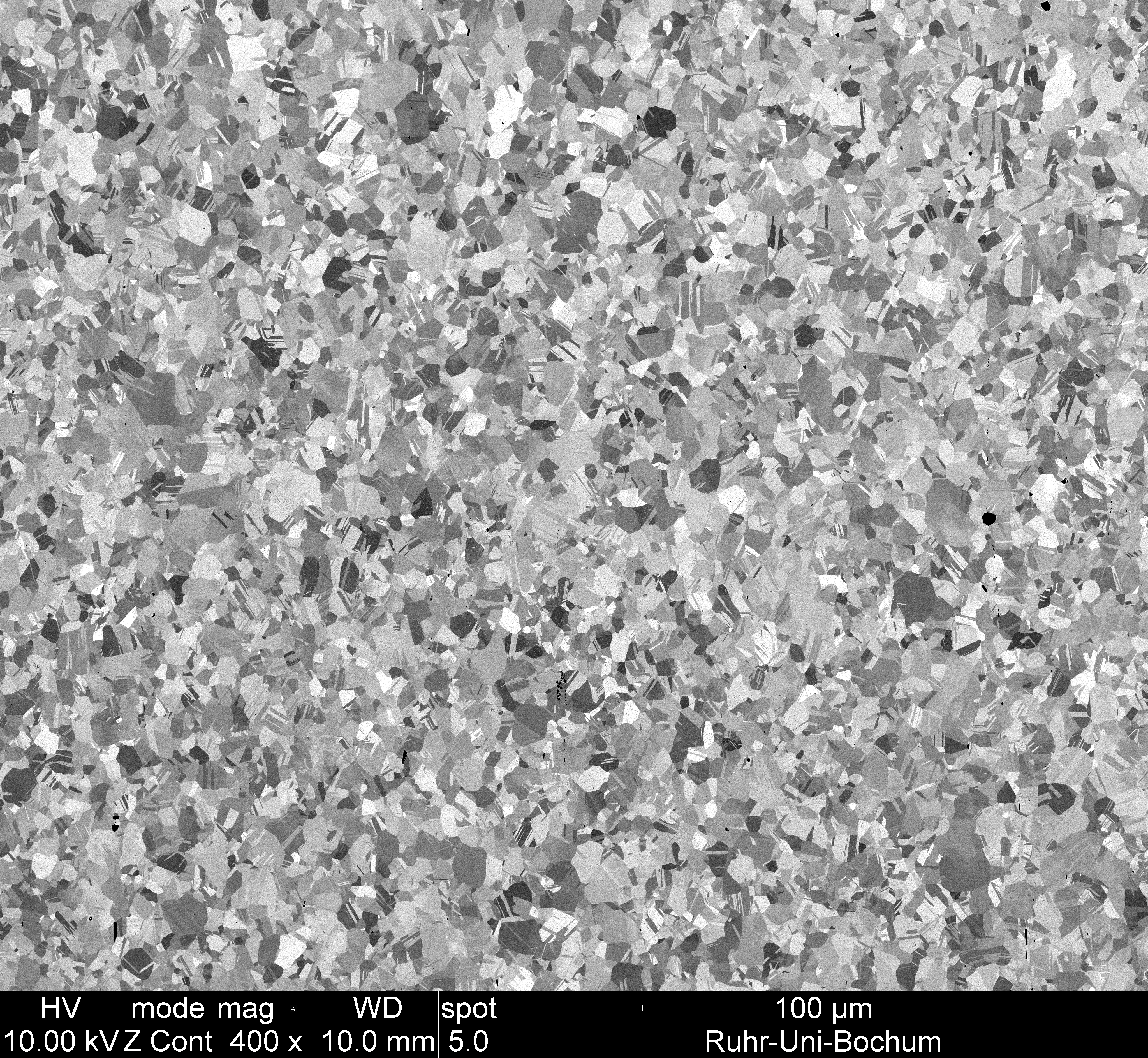

Supplement: Multimedia component 1 [file mmc1.zip › CrCoNi_1073K_120min/CrCoNi_1073K_120min_1.tif]

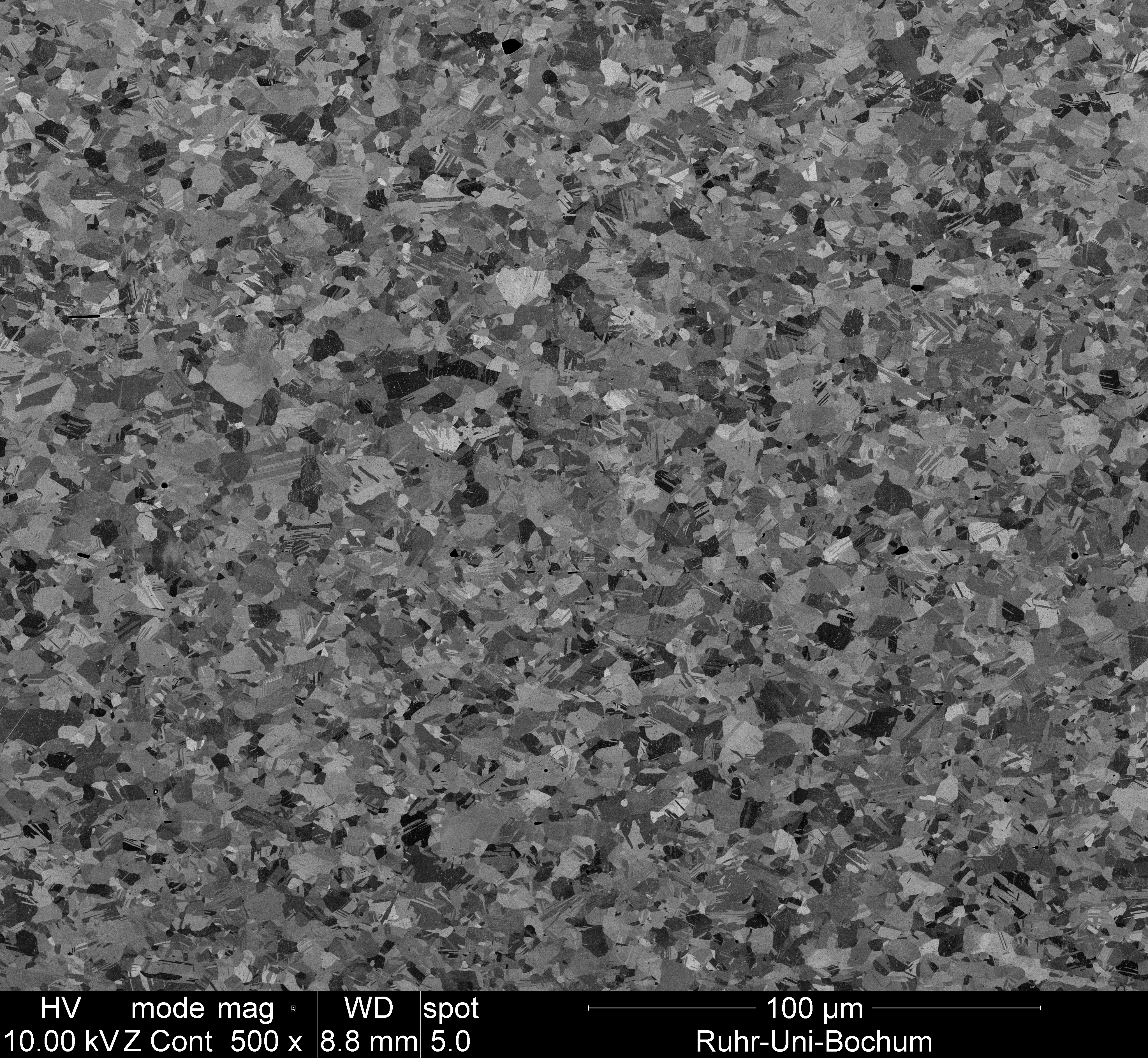

Supplement: Multimedia component 1 [file mmc1.zip › CrCoNi_1073K_15min/CrCoNi_1073K_15min_1.tif]

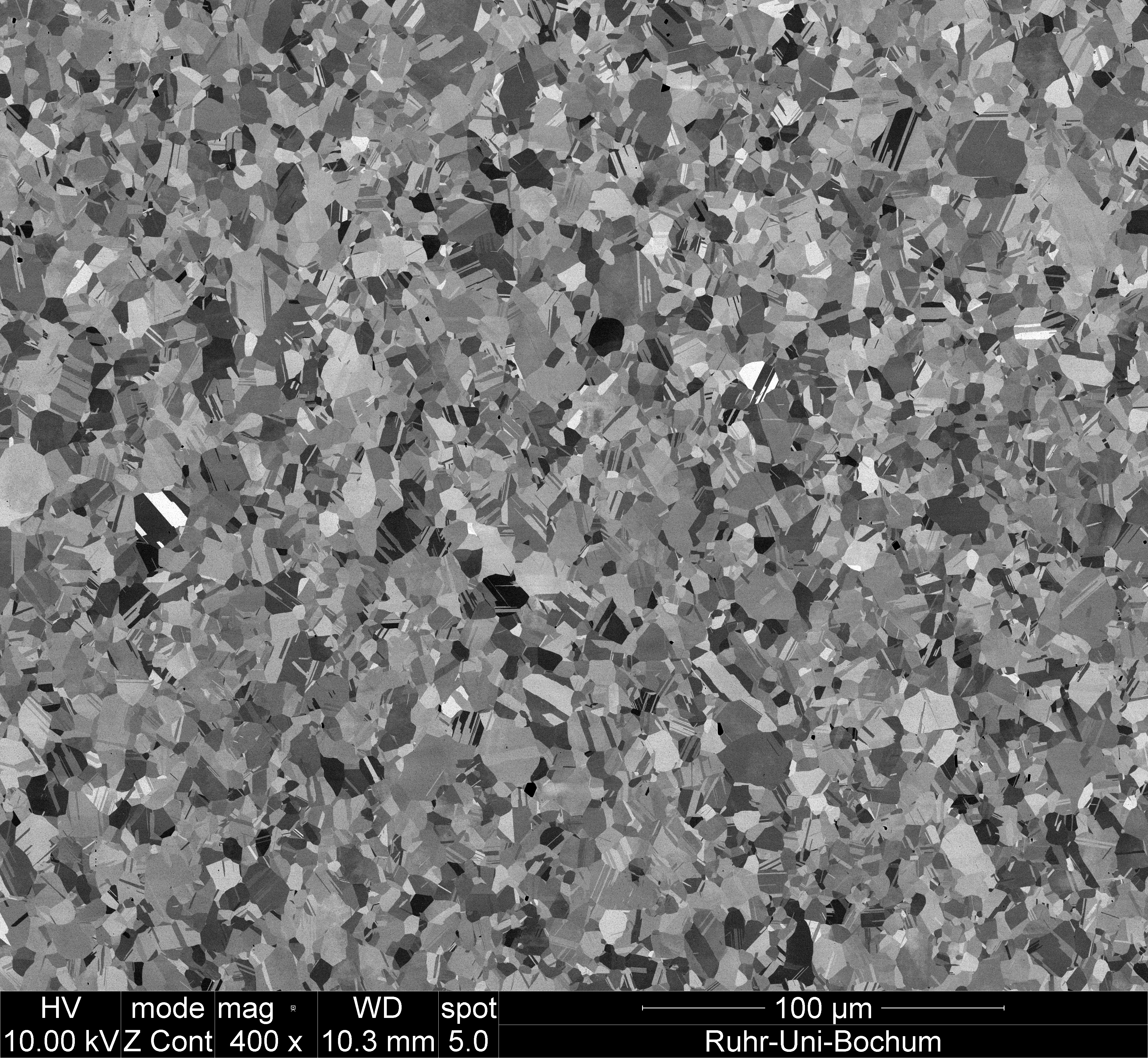

Supplement: Multimedia component 1 [file mmc1.zip › CrCoNi_1073K_180min/CrCoNi_1073K_180min_1.tif]

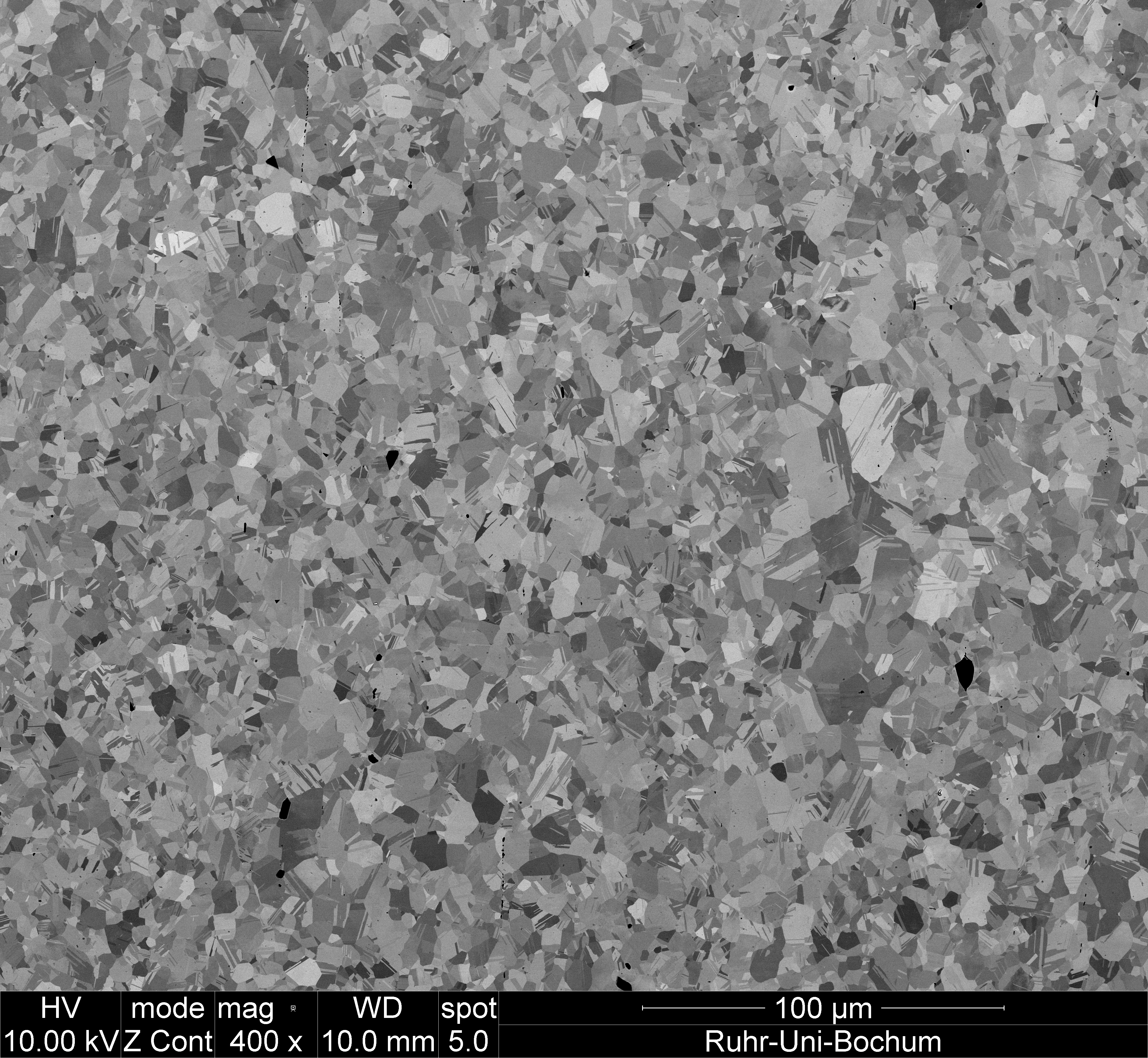

Supplement: Multimedia component 1 [file mmc1.zip › CrCoNi_1173K_10min/CrCoNi_1173K_10min_1.tif]

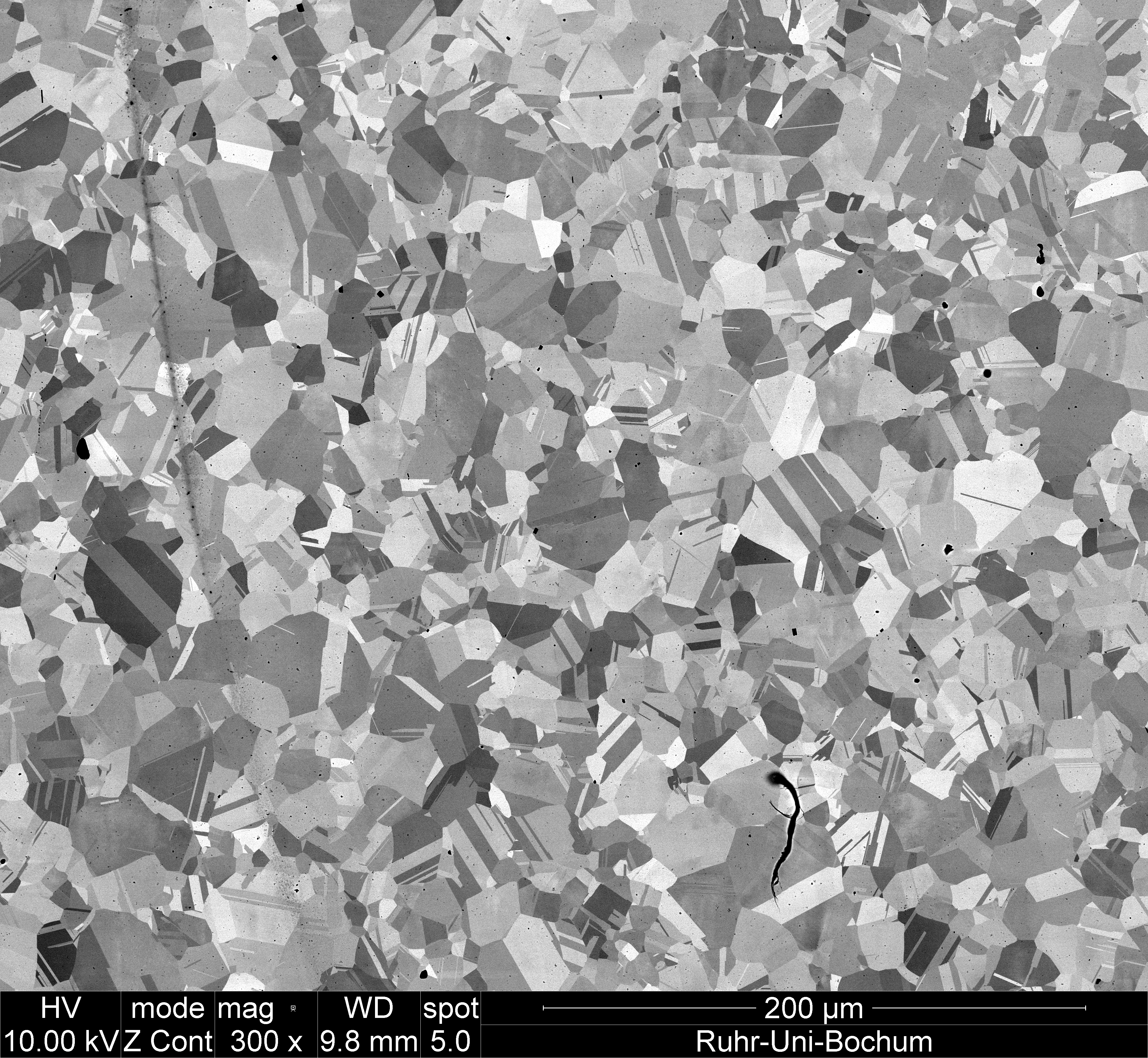

Supplement: Multimedia component 1 [file mmc1.zip › CrCoNi_1173K_120min/CrCoNi_1173K_120min_1.tif]

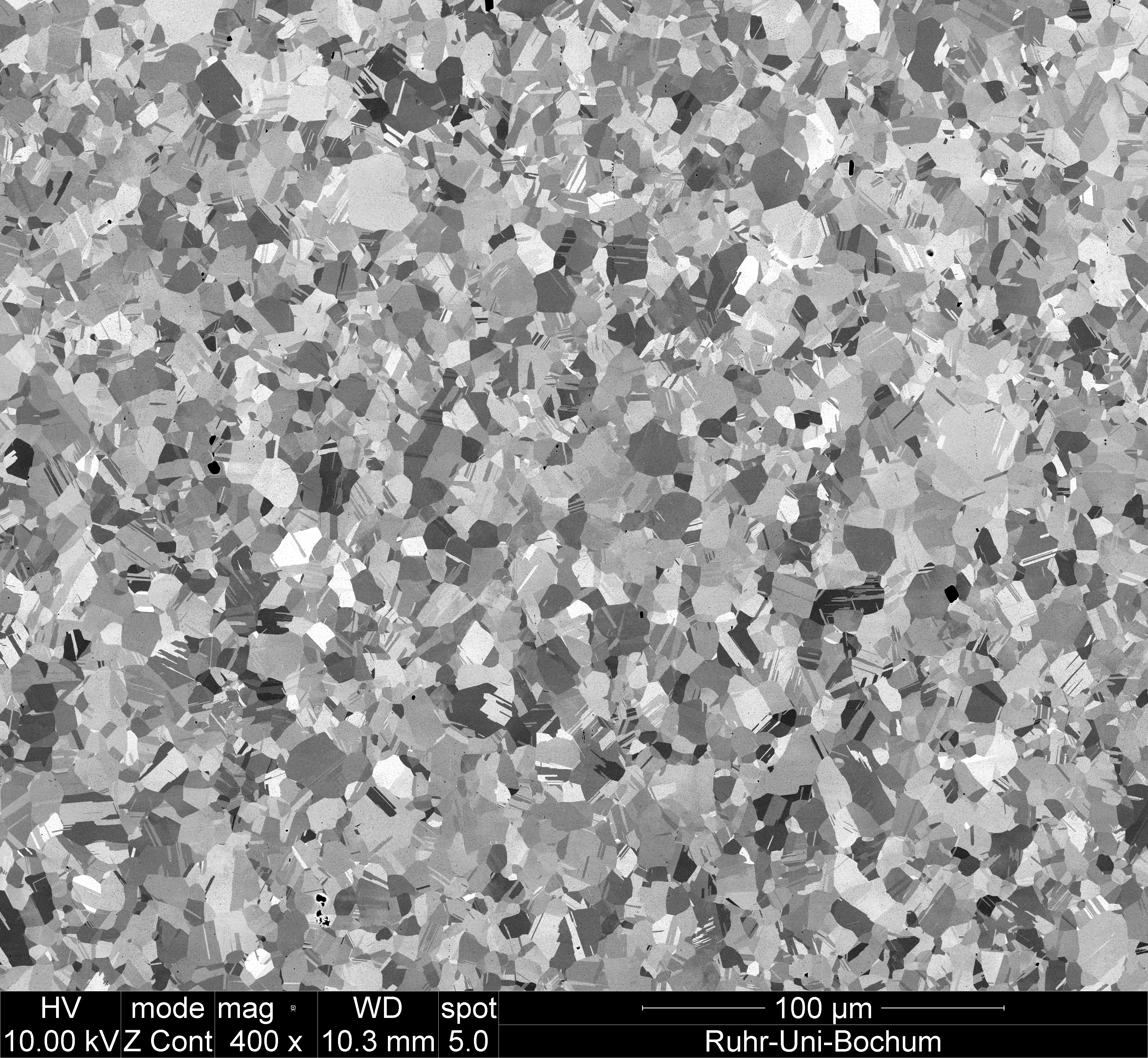

Supplement: Multimedia component 1 [file mmc1.zip › CrCoNi_1173K_15min/CrCoNi_1173K_15min_1.tif]

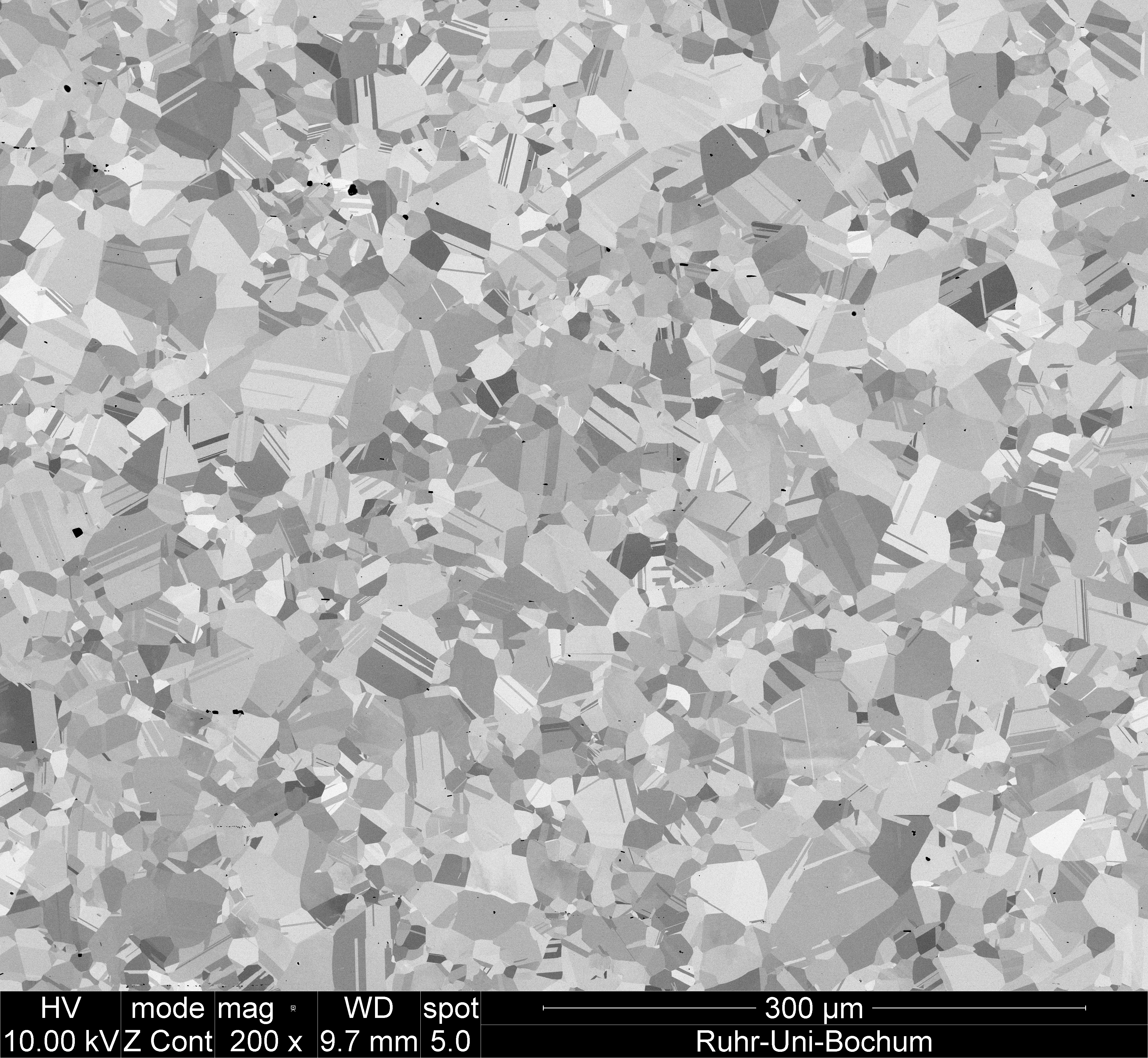

Supplement: Multimedia component 1 [file mmc1.zip › CrCoNi_1173K_180min/CrCoNi_1173K_180min_1.tif]

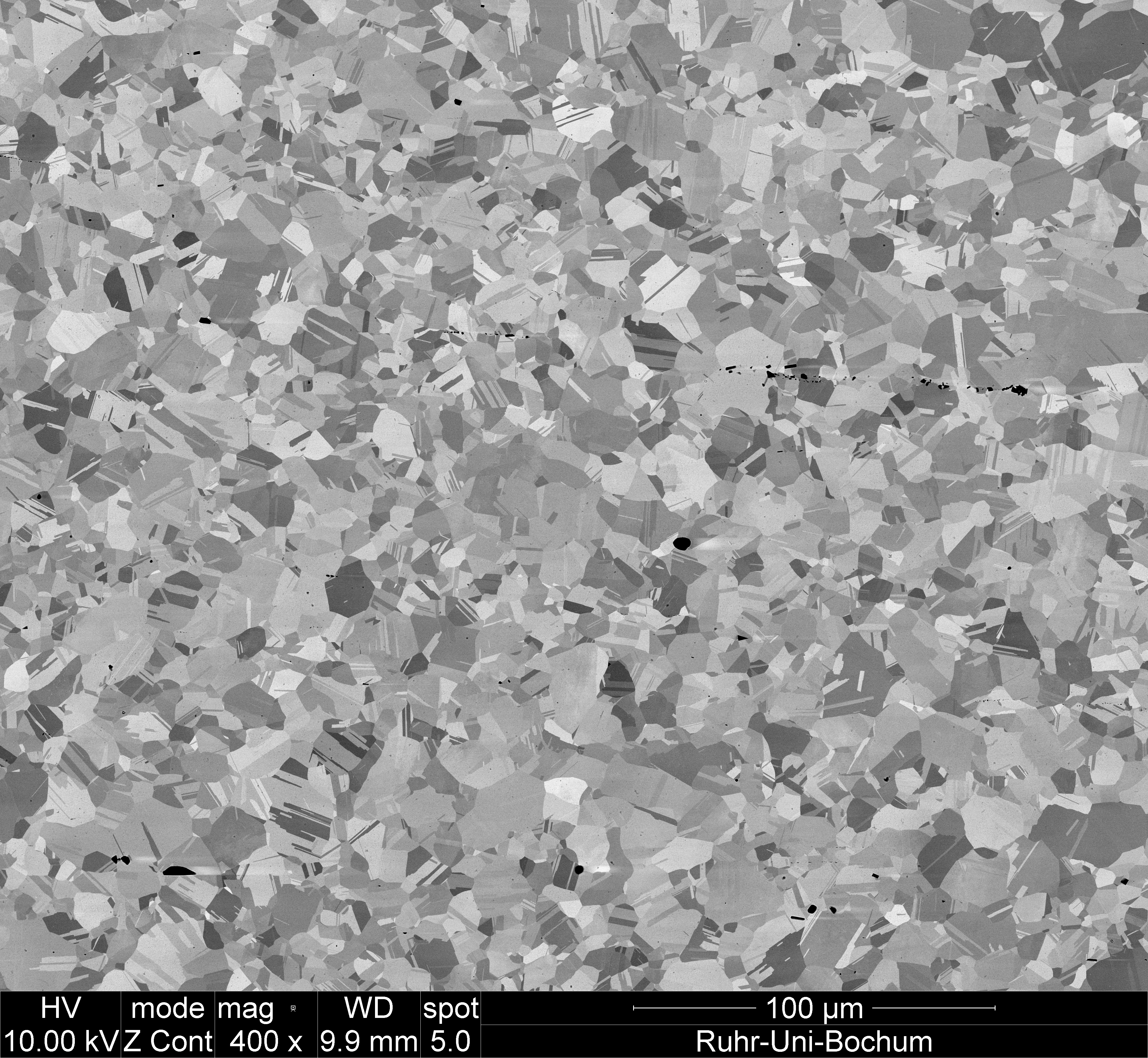

Supplement: Multimedia component 1 [file mmc1.zip › CrCoNi_1173K_20min/CrCoNi_1173K_20min_1.tif]

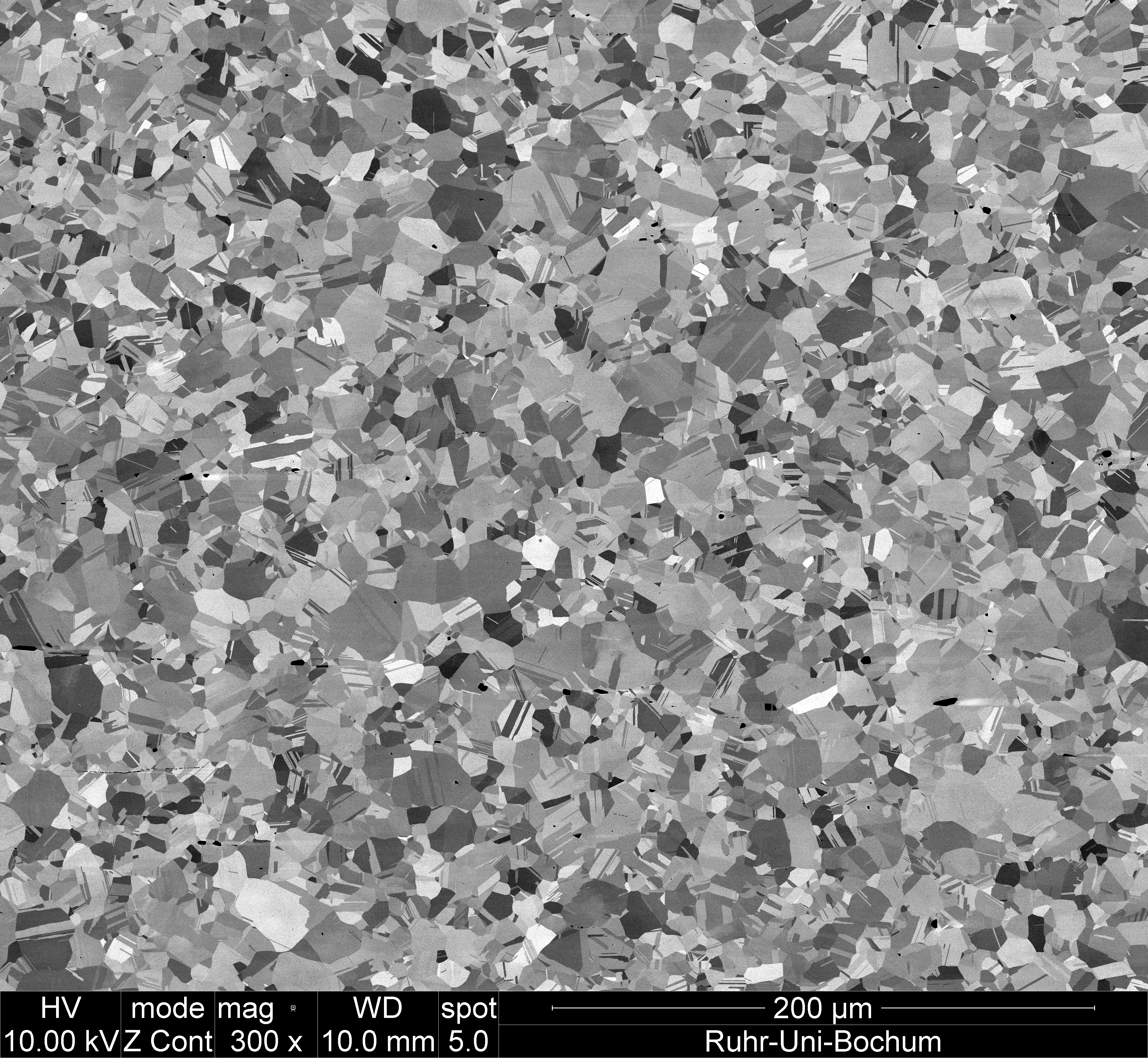

Supplement: Multimedia component 1 [file mmc1.zip › CrCoNi_1173K_30min/CrCoNi_1173K_30min_1.tif]

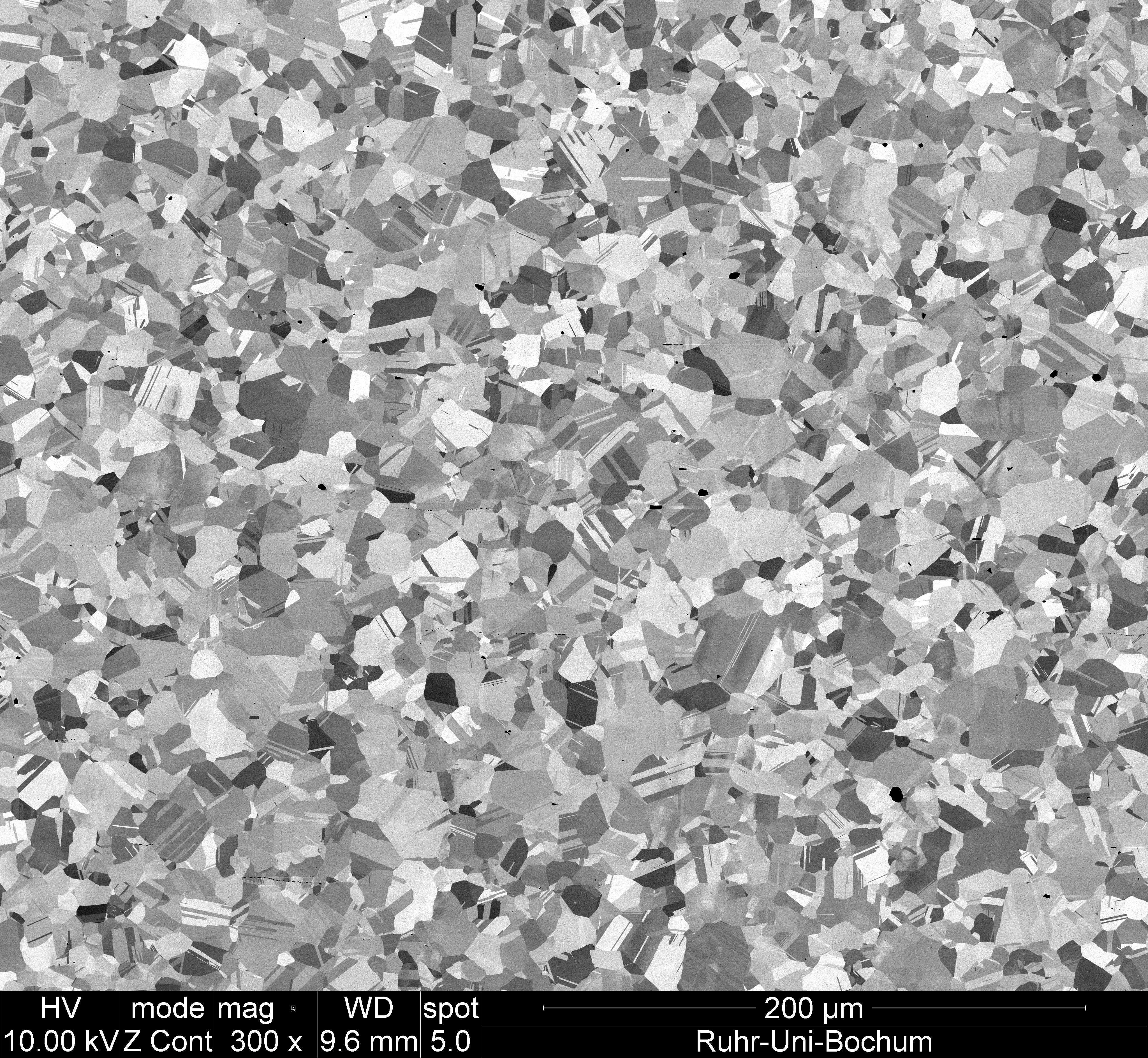

Supplement: Multimedia component 1 [file mmc1.zip › CrCoNi_1173K_45min/CrCoNi_1173K_45min_1.tif]

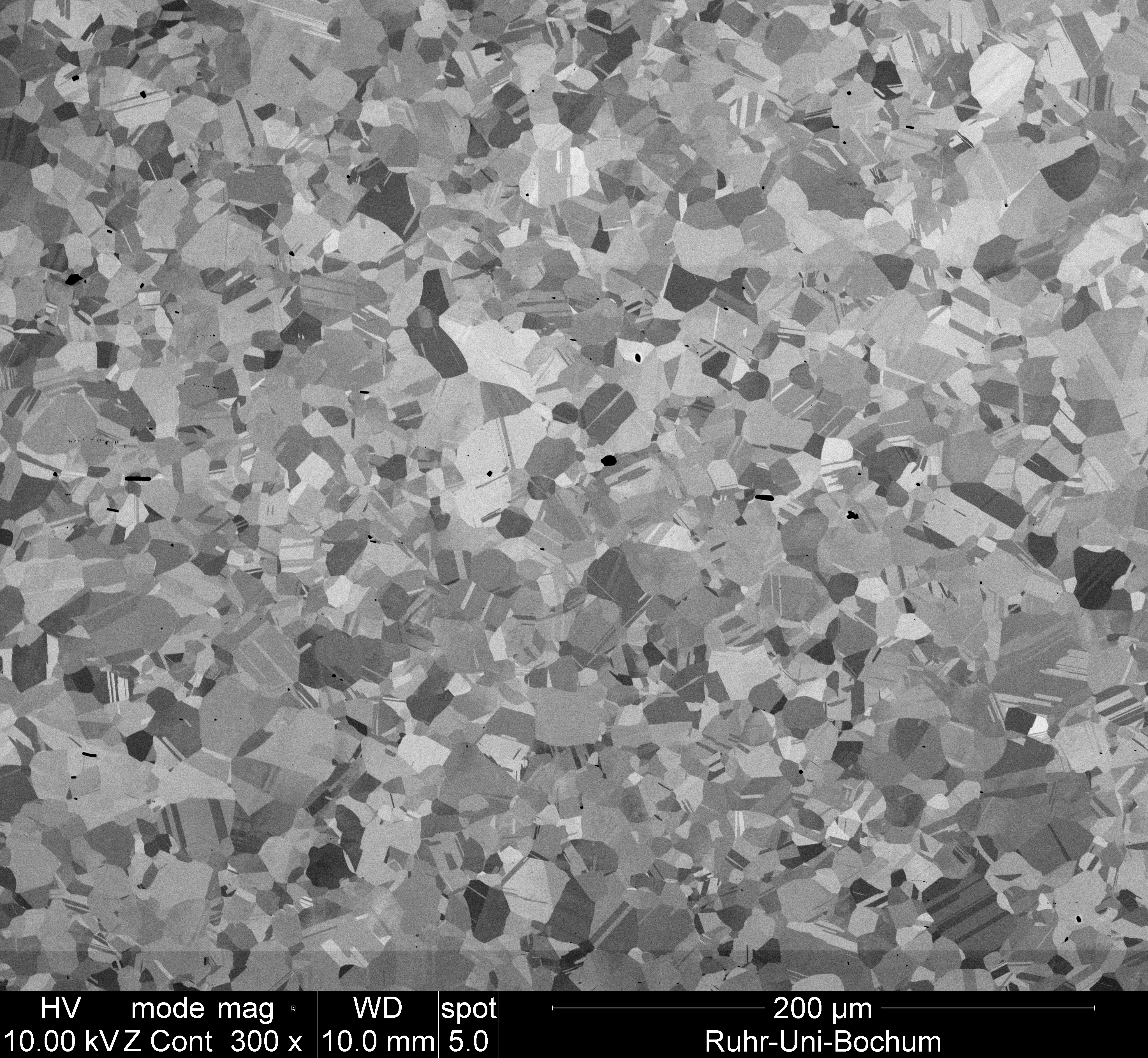

Supplement: Multimedia component 1 [file mmc1.zip › CrCoNi_1173K_60min/CrCoNi_1173K_60min_1.tif]

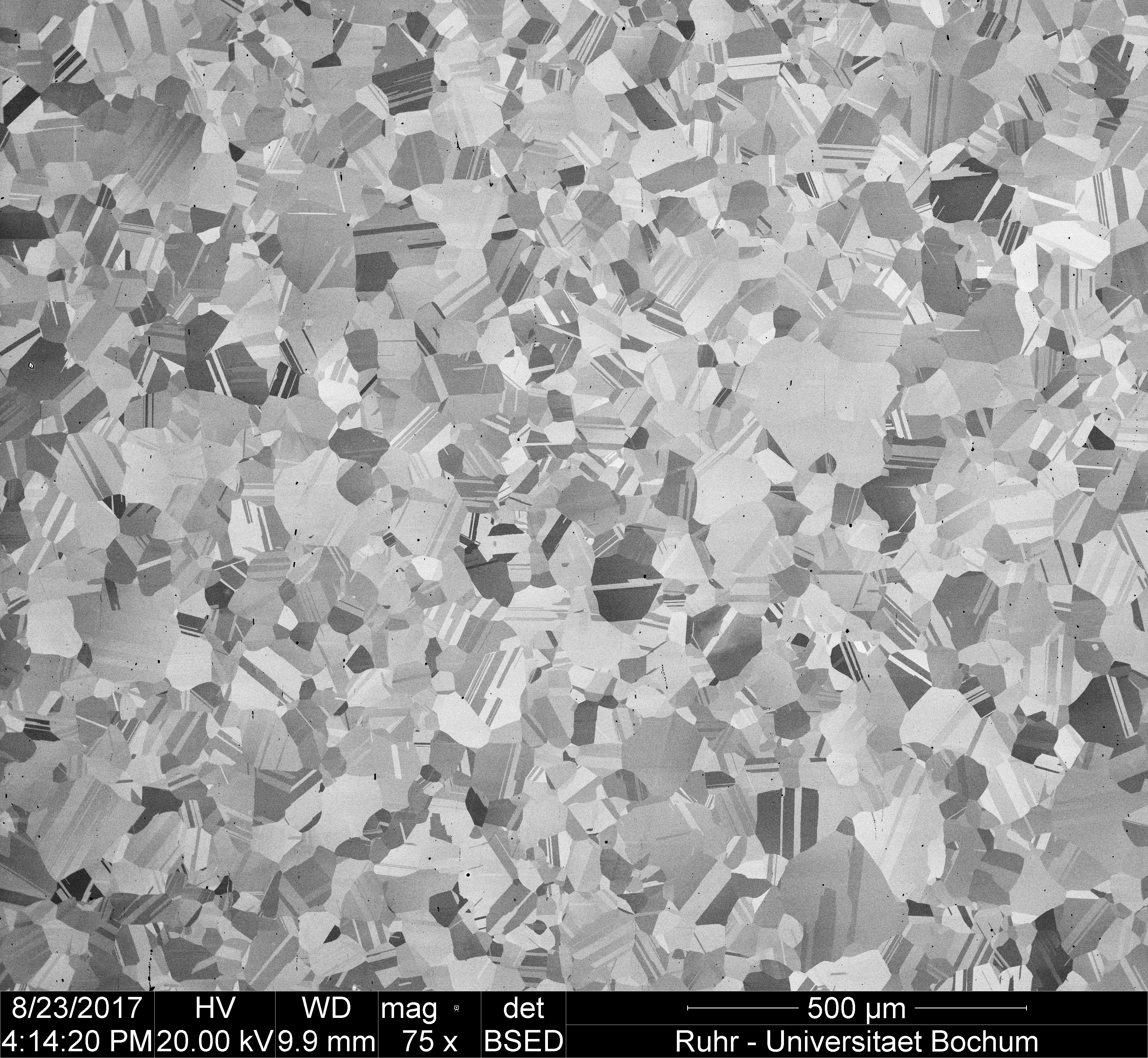

Supplement: Multimedia component 1 [file mmc1.zip › CrCoNi_1273K_180min/CrCoNi_1273K_180min_1.tif]

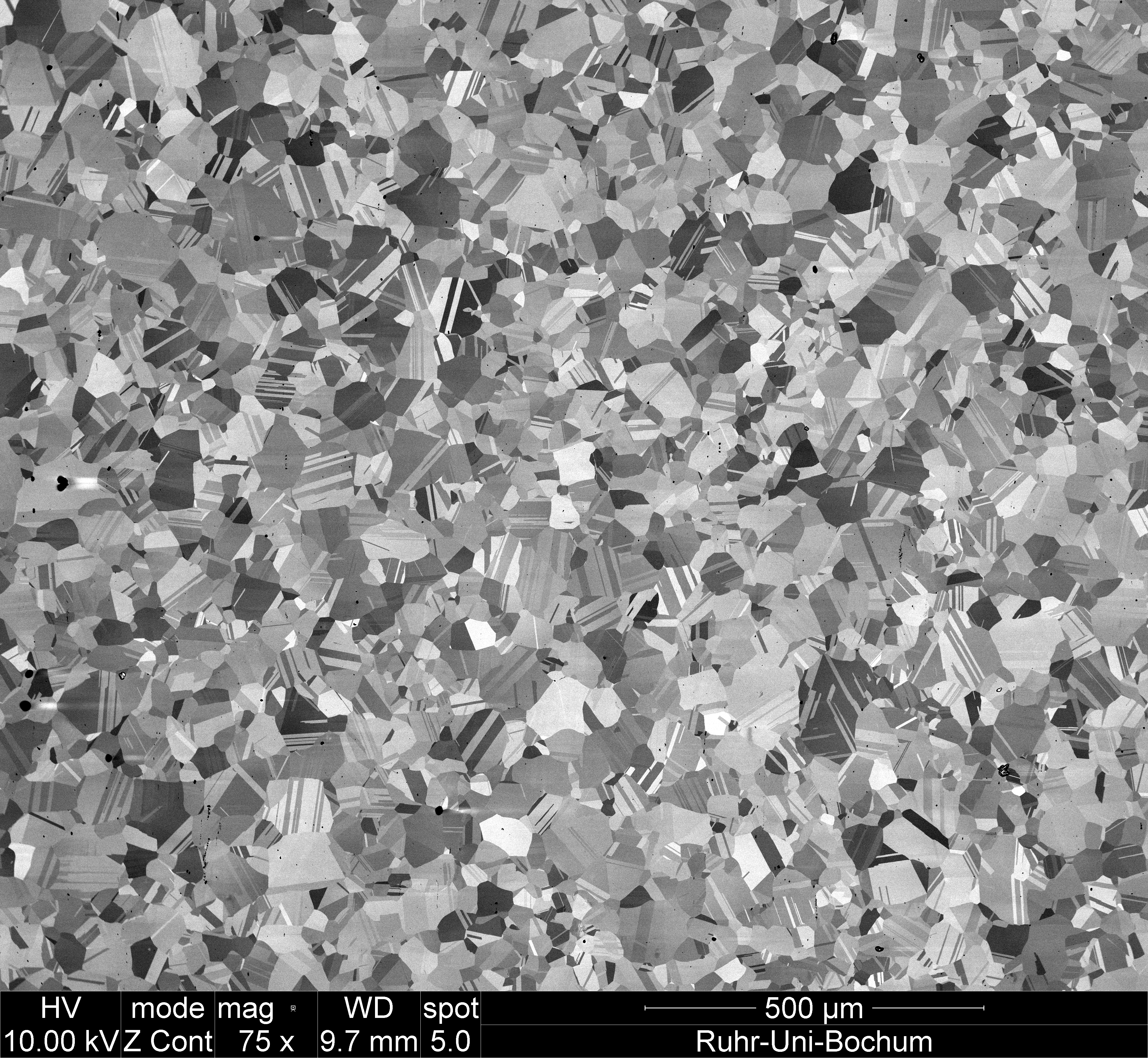

Supplement: Multimedia component 1 [file mmc1.zip › CrCoNi_1273K_60min/CrCoNi_1273K_60min_1.tif]

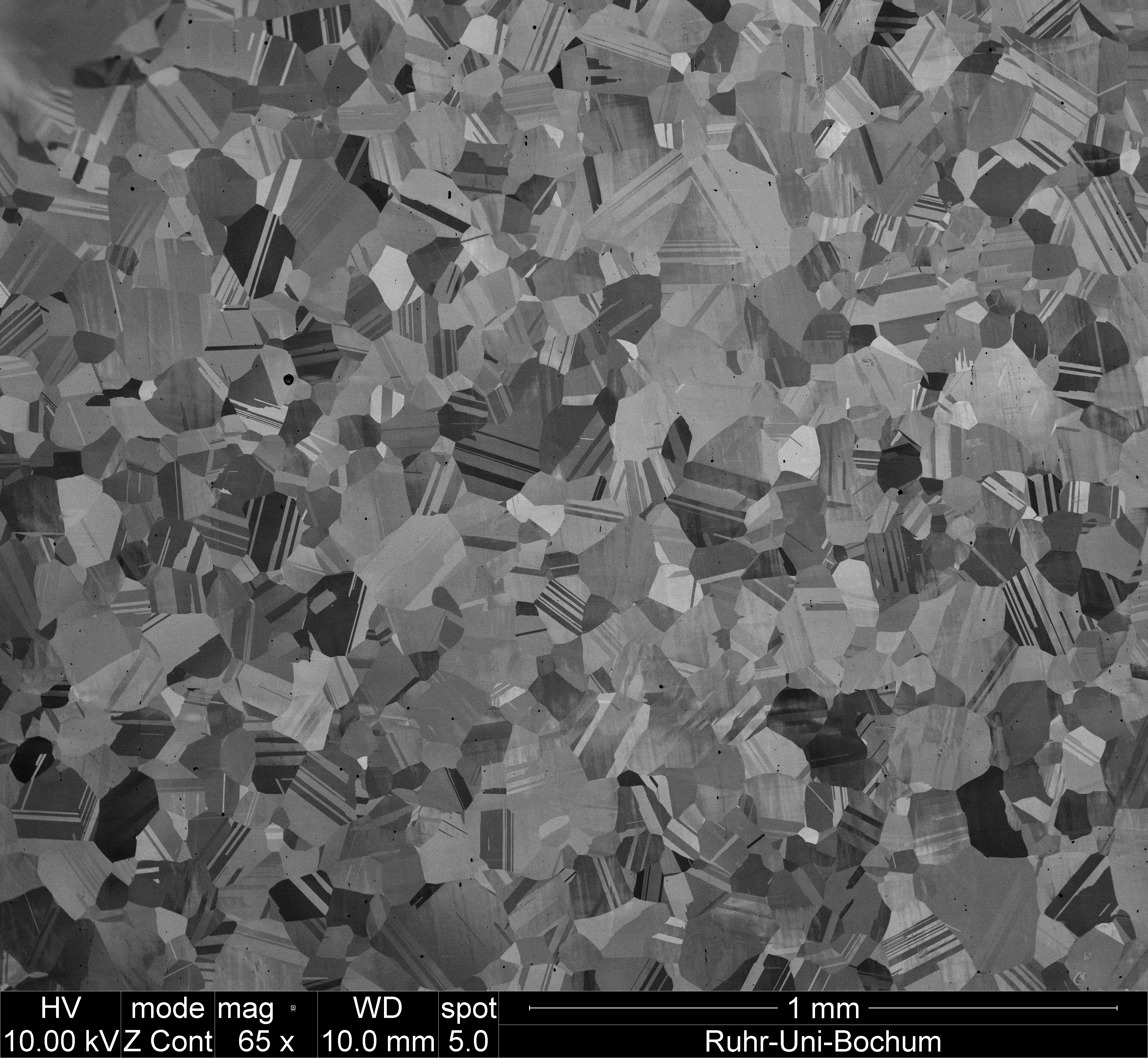

Supplement: Multimedia component 1 [file mmc1.zip › CrCoNi_1373K_30min/CrCoNi_1373K_30min_1.tif]

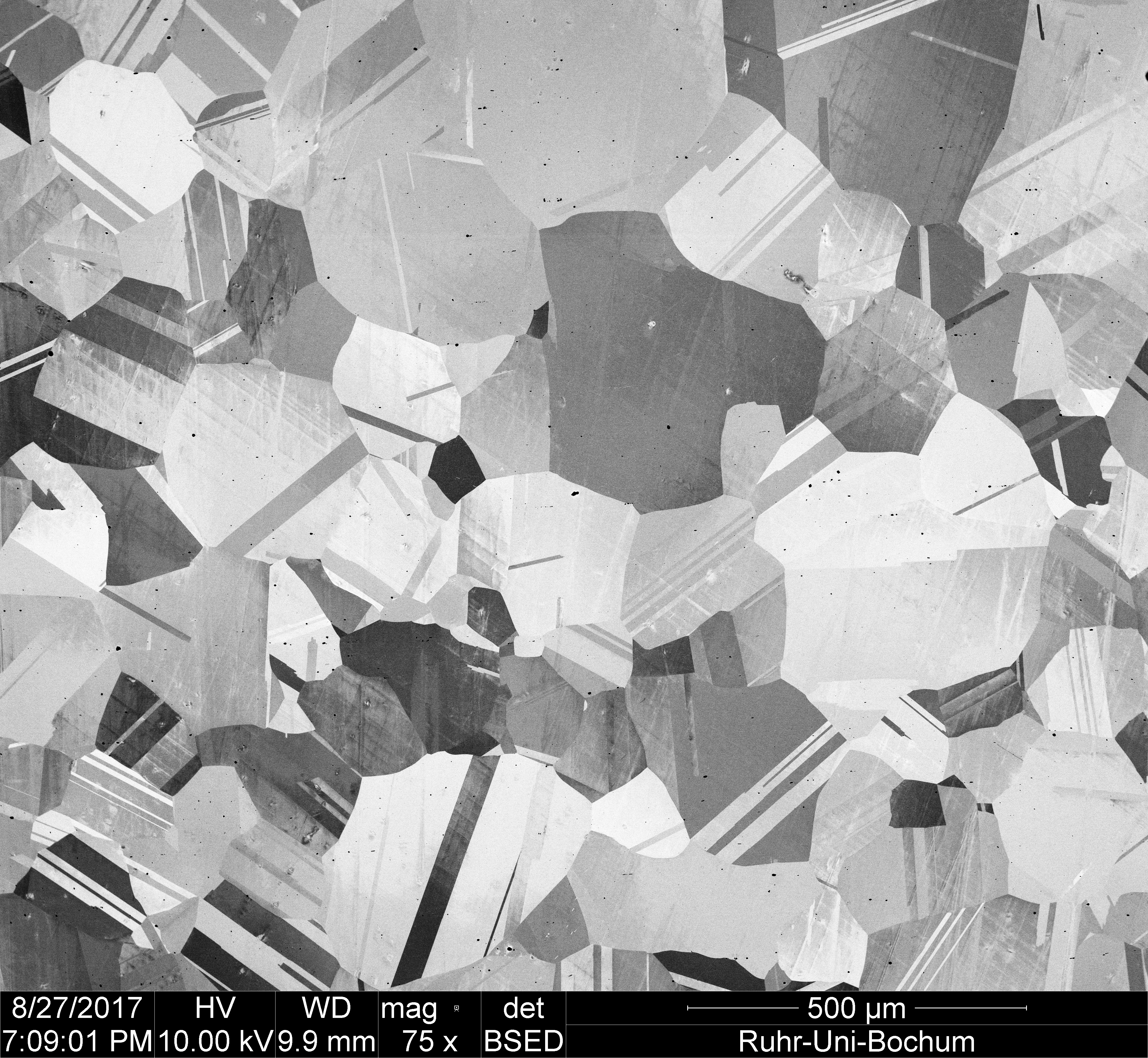

Supplement: Multimedia component 1 [file mmc1.zip › CrCoNi_1473K_30240min/CrCoNi_1473K_30240min_1.tif]

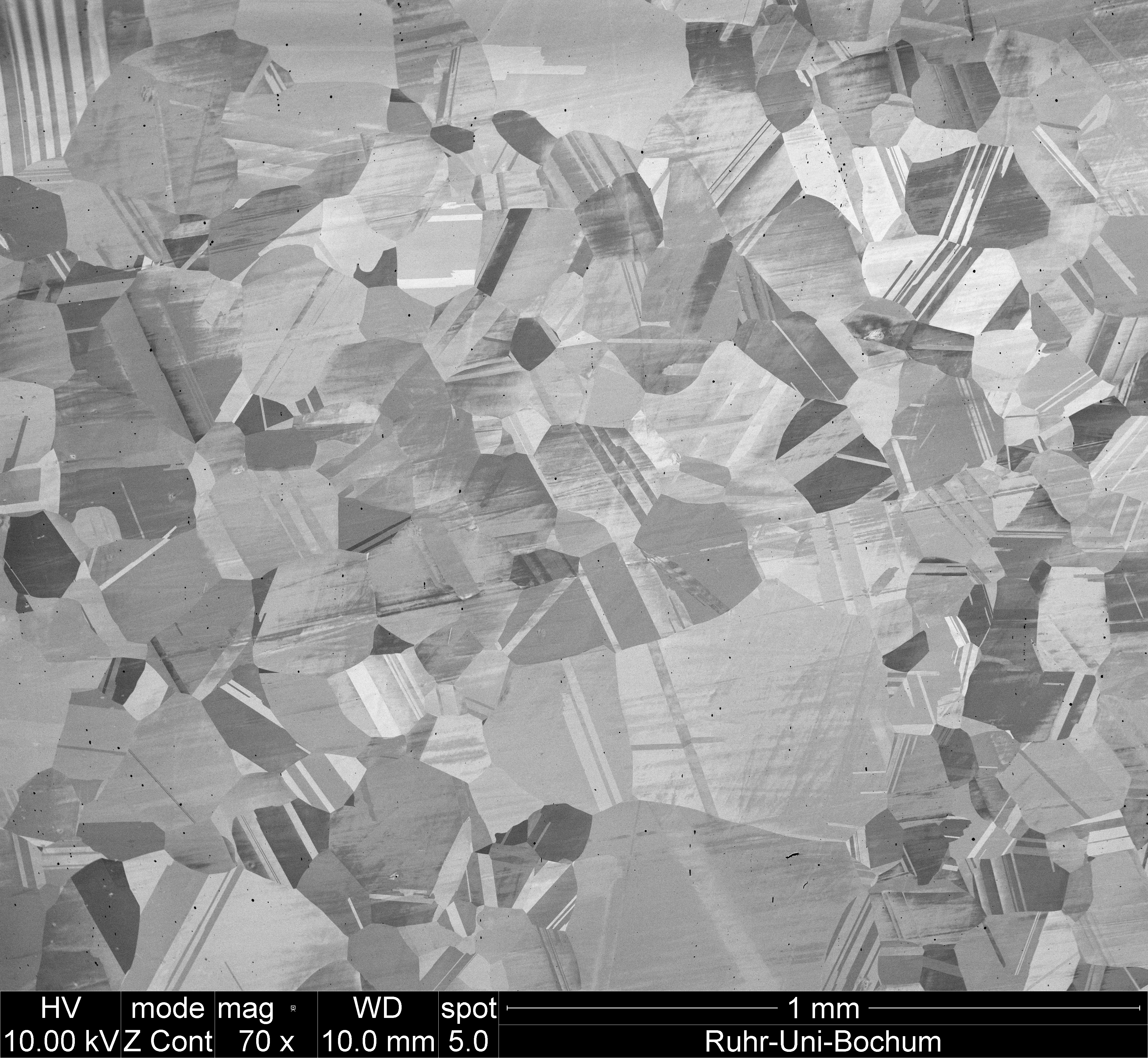

Supplement: Multimedia component 1 [file mmc1.zip › CrCoNi_1473K_30min/CrCoNi_1473K_30min_1.tif]
